# Highly Active Superbulky Alkaline Earth Metal Amide Catalysts for Hydrogenation of Challenging Alkenes and Aromatic Rings

**DOI:** 10.1002/anie.202001160

**Published:** 2020-03-27

**Authors:** Johannes Martin, Christian Knüpfer, Jonathan Eyselein, Christian Färber, Samuel Grams, Jens Langer, Katharina Thum, Michael Wiesinger, Sjoerd Harder

**Affiliations:** ^1^ Chair of Inorganic and Organometallic Chemistry Universität Erlangen-Nürnberg Egerlandstrasse 1 91058 Erlangen Germany

**Keywords:** Alkaline Earth Metals, Alkenes, Aromatic rings, DFT calculations, Hydrogenation

## Abstract

Two series of bulky alkaline earth (Ae) metal amide complexes have been prepared: Ae[N(TRIP)_2_]_2_ (**1**‐Ae) and Ae[N(TRIP)(DIPP)]_2_ (**2**‐Ae) (Ae=Mg, Ca, Sr, Ba; TRIP=Si*i*Pr_3_, DIPP=2,6‐diisopropylphenyl). While monomeric **1**‐Ca was already known, the new complexes have been structurally characterized. Monomers **1**‐Ae are highly linear while the monomers **2**‐Ae are slightly bent. The bulkier amide complexes **1**‐Ae are by far the most active catalysts in alkene hydrogenation with activities increasing from Mg to Ba. Catalyst **1**‐Ba can reduce internal alkenes like cyclohexene or 3‐hexene and highly challenging substrates like 1‐Me‐cyclohexene or tetraphenylethylene. It is also active in arene hydrogenation reducing anthracene and naphthalene (even when substituted with an alkyl) as well as biphenyl. Benzene could be reduced to cyclohexane but full conversion was not reached. The first step in catalytic hydrogenation is formation of an (amide)AeH species, which can form larger aggregates. Increasing the bulk of the amide ligand decreases aggregate size but it is unclear what the true catalyst(s) is (are). DFT calculations suggest that amide bulk also has a noticeable influence on the thermodynamics for formation of the (amide)AeH species. Complex **1**‐Ba is currently the most powerful Ae metal hydrogenation catalyst. Due to tremendously increased activities in comparison to those of previously reported catalysts, the substrate scope in hydrogenation catalysis could be extended to challenging multi‐substituted unactivated alkenes and even to arenes among which benzene.

## Graphical abstract

**Bulk=Hulk**: Superbulky alkaline earth metal amide complexes were found to be extremely active catalysts for alkene hydrogenation, clearly extending the substrate scope. Even various arenes, including benzene, can be reduced under relatively mild conditions.

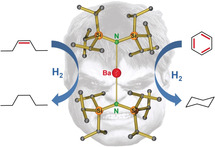

## Introduction

**Scheme 1:**
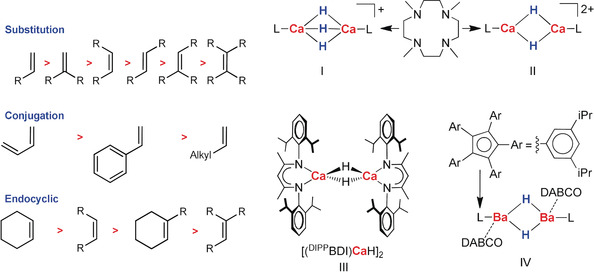
Reactivity order for alkene hydrogenation.

Addition of molecular hydrogen to multiple bonds may seem a simple reaction. In reality, however, this industrially important transformation has been subject of immense research.^1^ Since Sabatier's milestone discovery,^2^ alkene‐to‐alkane conversion has been fully dominated by transition metal catalysts. The last decade, however, saw the introduction of *s*‐block metal catalysts,^3^, ^4^, ^5^, ^6^, ^7^, ^8^, ^9^, ^10^, ^11^ or even metal‐free systems like Frustrated Lewis Pairs^12^ or boranes,^13^ showing that alkene activation by d→π* backbonding is not an absolute requirement.^14^, ^15^ Since the first report on alkaline earth (Ae) metal catalyzed alkene hydrogenation^3^ there has been a considerable improvement in catalyst performance, continuously shifting borders to milder reaction conditions and extending the substrate scope to more challenging substrates. In general, alkene hydrogenation becomes more difficult with the number of substituents while conjugation or incorporation in a ring system facilitate reduction (Scheme 1).

In 2008 we reported the hydrogenation of activated (conjugated) alkenes with K, Ca, and Sr benzyl catalysts or a β‐diketiminate Ca hydride complex.^3^ This transformation follows a simple mechanism in which addition of the alkene to a metal hydride intermediate is a key step. Since styrene or butadiene type substrates react to resonance‐stabilized benzylic or allylic intermediates, hydrogenation of conjugated double bonds is facile.

Okuda and co‐workers introduced cationic calcium hydride catalysts (**I**–**II**) and demonstrated hydrogenation of unactivated alkenes like 1‐hexene.^6^, ^7^, ^8^ As the dicationic catalyst (**II**) was found to be particularly active, it was concluded that the positive charge is critical in imparting sufficient electrophilicity to the metal center. The Hill group introduced a THF‐free analogue (**III**)^16^ of an earlier reported β‐diketiminate Ca hydride complex.^17^ Unstabilized by a Lewis base, this hydride complex is extremely reactive, however, at higher temperatures it is also prone to ligand exchange by the Schlenk equilibrium: [(^DIPP^BDI)CaH]_2_ ⇌ (^DIPP^BDI)_2_Ca + CaH_2_. Since precipitation of (CaH_2_)_∞_ results in catalyst deactivation, alkene hydrogenation was studied at 25 °C. Unactivated alkenes like 1‐hexene or norbornene could be reduced, however, conversion times of 2–3 weeks are needed.^9^

**Scheme 2:**
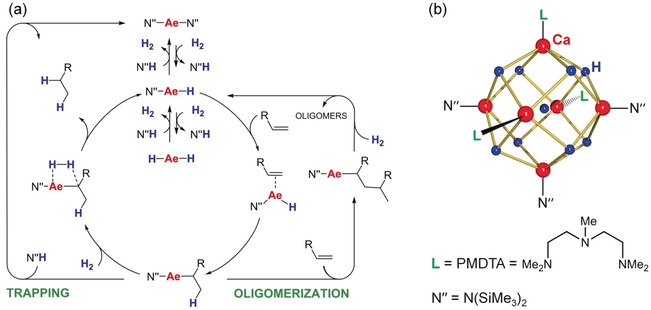
a) Catalytic cycle for alkene hydrogenation with AeN′′_2_ catalysts. b) Crystal structure of Ca_6_H_9_N′′_3_⋅(PMDTA)_3_.21a

At the same time, we reported the unexpected catalytic activity of well‐known Ae[N(SiMe_3_)_2_]_2_ complexes abbreviated as AeN′′_2_ (Ae=Ca, Sr, Ba).^4^ Metal hydride formation by deprotonation of H_2_ (*p*K_a_≈49)^18^ by this weak base (*p*K_a_ of N′′H=25.8)^19^ seemed illogical (Scheme 2) and was calculated to be endergonic by ca. 11 kcal mol^−1^. However, reaction of CaN′′_2_ with H_2_ in benzene gave N′′H and undefined clusters (N′′CaH)_*x*_(CaH_2_)_*y*_ with a MW up to 7500.^20^ The energy released in the aggregation process is the driving force for this reaction. Addition of PMDTA enabled interception of the smaller cluster (N′′CaH)_3_(CaH_2_)_3_⋅(PMDTA)_3_ (Scheme 2 b); also small Sr and Ba clusters have been isolated.^21^ Use of simple AeN′′_2_ catalysts turned out to be highly advantageous. First of all, these catalysts fully suppress alkene oligomerization. This undesired side‐reaction is generally observed for activated substrates like styrene for which oligomerization is competitive with the more difficult H_2_ deprotonation. However, for AeN′′_2_ catalysts the acidic amine N′′H (formed during catalyst initiation) rapidly traps the benzylic intermediate preventing oligomerization (Scheme 2 a). Noteworthy is also the very high thermal stability of these catalysts. At an operating temperature of 120 °C, decomposition and Schlenk equilibria are not an issue. The activities of AeN′′_2_ catalysts strongly increase with metal size: Ca < Sr < Ba. The superb activity of the Ba catalyst was independently confirmed by the Cheng group who introduced a highly active barium hydride catalyst (**IV**) that operates at 30 °C.^10^

Despite continuous breakthroughs in alkene hydrogenation, early main group metal catalysts are a far cry from the classical transition metal catalysts. While unactivated terminal alkenes like 1‐hexene can be reduced, internal alkenes or higher substituted alkenes pose a problem: semi‐activated norbornene could be hydrogenated^4^, ^9^ but cyclohexene is already fully inert.

By far more challenging than alkene hydrogenation is the reduction of highly stable aromatic arenes. The catalysts for breaking the ring's aromaticity are generally based on platinum group metals. Many homogeneous catalysts react in reality as heterogenous systems,^22^ that is, larger nanoparticles often operating under harsher conditions, and very few true homogeneous catalysts exist.^23^, ^24^ Stephan and co‐workers reported the metal‐free catalyst B(C_6_F_5_)_3_ for hydrogenation of N‐containing rings or arenes.^25^, ^26^ In these cases the substrate is part of the Frustrated Lewis Pair (FLP) catalyst but the FLP combination Ph_2_PC_6_F_5_/B(C_6_F_5_)_3_ was able to hydrogenate larger polycyclic aromatic hydrocarbons (PAH′s) like anthracene and tetracene. Although these extended π‐systems are generally easier reduced, harsh conditions were needed (80 °C, 100 bar H_2_).^27^ Stock demonstrated that LiN(*i*Pr)_2_ or KN(SiMe_3_)_2_ catalyze the reduction of activated PAH′s like anthracene or naphthalene under drastic conditions (25 mol % catalyst, 200 °C, 70 bar H_2_, 18 h).^28^ The Hill group very recently demonstrated the stoichiometric reduction of activated PAH′s with Ca hydride complex **IV** 29 but the catalytic hydrogenation of aromatic rings with Ae metal catalysts has hitherto not been reported.

We here introduce a concept that boosts the activity of Ae metal amide catalysts tremendously. We demonstrate the hydrogenation of most challenging alkenes and, for the first time, also of aromatic rings.

## Results and Discussion

**Scheme 3:**
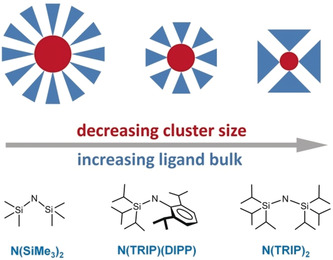
Influence of ligand bulk on particle aggregation and cluster size (red: cluster core, blue: capping ligand).

In contrast to the many advantages for hydrogenation with AeN′′_2_ catalysts, there is one major drawback: generally high catalyst loadings of 5–10 mol % are needed.^20^ We attribute this to the aforementioned aggregation to larger (N′′AeH)_*x*_(AeH_2_)_*y*_ clusters resulting in a drastic lowering of the catalyst concentration. Work by Jones, Ruhlandt–Senge and Mills has shown that superbulky amide ligands suppress aggregation.^30^, ^31^, ^32^, ^33^ It was reasoned that use of superbulky amides will decrease cluster size and increase catalyst concentration (Scheme 3).

Fine‐tuning the size of nanoparticles with capping ligands is a well‐known concept: bulkier ligands decrease core size while sharpening the particle size distribution.^34^ For Pd nanoparticles capped by dendritic ligands, the activity in alkene hydrogenation increased with dendrimer generation.^35^ The same principle also applies to smaller molecular clusters.^36^

### Synthesis and structures

**Figure 1:**
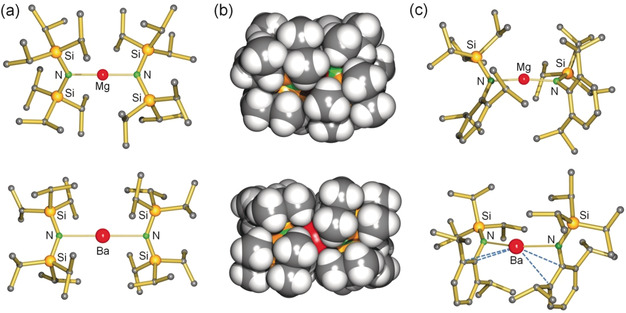
Selected crystal structures: a) **1**‐Mg and **1**‐Ba; H atoms omitted. b) Space‐filling models for **1**‐Mg (top) and **1**‐Ba (bottom). c) **2**‐Mg and **2**‐Ba; H atoms omitted.

We targeted the syntheses of the superbulky Ae metal amides Ae[N(TRIP)_2_]_2_ (**1**‐Ae) and Ae[N(TRIP)(DIPP)]_2_ (**2**‐Ae), see Scheme 3 for abbreviations. Complex **1**‐Ca has been reported but earlier attempts to isolate **1**‐Mg or **1**‐Sr failed.^33^ Amines HN(TRIP)_2_ and HN(TRIP)(DIPP)^33^, ^37^, ^38^ were converted to their potassium salts and subsequent reaction with AeI_2_ in aromatic solvents gave the bulky amide complexes **1**‐Ae (Ae=Mg, Ca, Sr, Ba) in crystallized yields of 57–67 %. Similarly, **2**‐Ae (Ae=Mg, Ca, Sr, Ba) complexes were isolated in yields of 40–46 %. The excellent solubility of these complexes in alkanes prevents high crystallized yields. All complexes were fully characterized by NMR, CHN analyses and X‐ray diffraction (Figure 1; Supporting Information, Table S2).

Complexes in the series **1**‐Ae are all monomeric featuring (nearly) linear two‐coordinated metal ions with N−Ae−N′ angles ranging from 171.9(1)° to 179.7(1)°. The Mg−N bond in **1**‐Mg (1.997(1) Å) is clearly longer than that in monomeric MgN′′_2_ (1.91(3) Å)^39^ illustrating the considerable steric stress in the **1**‐Ae series. Noteworthy is the linear nature of **1**‐Ba: N−Ba−N′=177.5(1)°. While Sarazin recently reported the first two‐coordinate Ba complex, a boryloxide complex that is strongly bent,^40^ **1**‐Ba is the first example of a near linear two‐coordinate barium compound. All complexes feature numerous short anagostic interactions with the *i*Pr groups (see Table S2). The Si−N−Si′ angles are very obtuse and increase with metal size from 132.4(1)° in **1**‐Mg to 141.3(1)° in **1**‐Ba. The *bis*‐silyl amide anion (R_3_Si)_2_N^−^ is isolobal to (R_3_Si)_2_O and Si−N−Si′ units have similar to Si−O−Si′ units the tendency to be linear.^41^ This originates from negative hyperconjugation, that is, partial charge delocalization of the N (or O) lone pairs in empty σ*(Si−R) orbitals,^42^ which increases along the series **1**‐Mg <**1**‐Ca <**1**‐Sr <**1**‐Ba (this is indicated by increasing Si‐N‐Si′ angles and decreasing Si−N bonds, Table S2).

Complexes **2**‐Ae are also monomeric but with N−Ae−N′ angles ranging from 143.8(1)° to 169.5(1)° they are clearly less linear than the **1**‐Ae series. The Ae−N distances in **2**‐Ae are consistently shorter than those in **1**‐Ae by 0.06–0.08 Å, showing that N(TRIP)_2_ is bulkier. The N(TRIP)(DIPP) ligand, however, should still be considered bulky: the Mg−N bonds in **2**‐Mg (1.934(2)–1.946(2) Å) are significantly longer than those in Mg[N(SiMe_3_)(DIPP)]_2_ (1.919(2) Å).32b The larger metal ions in the **2**‐Ae series have a strong tendency to interact with the DIPP π‐system (this is especially the case in **2**‐Ba). The Ae⋅⋅⋅DIPP interactions are clearly more important than anagostic Ae⋅⋅⋅TRIP interactions. This is evident from the very acute Ae−N−C angles (average: 89.1–90.5°) and rather obtuse Ae−N−Si angles (average: 131.8–138.7°).

### Alkene hydrogenation

Since deactivating Schlenk equilibria do not play a role for **1**‐Ae and **2**‐Ae, these very robust catalysts were tested at 120 °C but the H_2_ pressure was kept low at 6 bar (Table 1; see Table S3 for a more extensive list including additional substrates and different catalysts). As a first test case the hydrogenation of 1‐hexene, an unactivated alkene, was investigated. While the Mg catalysts **1**‐Mg and **2**‐Mg are essentially inactive, activities increased considerably down the group: Mg < Ca < Sr < Ba (entries 1–8). The catalysts with the bulkier N(TRIP)_2_ ligand (**1**‐Ae) clearly show the better performance. The less active **2**‐Ae catalysts gave much more isomerisation to internal alkenes which could not be reduced further. The Ba catalyst **1**‐Ba shows significant activity at 60 °C but is slow at room temperature (entries 9–10). Lowering the H_2_ pressure to 1 bar gave considerable isomerisation and incomplete reduction (entry 11) but lowering the catalyst loading to 1 mol % is unproblematic (entry 12). Compared to BaN′′_2_ the superbulky catalyst **1**‐Ba is extremely active (cf. entries 8 and 13). Sterically hindered alkenes like *t*BuC(H)=CH_2_ could be reduced within hours (entry 14) while hydrogenation of 1,5‐hexadiene led only to minor cyclization (entry 15). The very high activity of **1**‐Ba is further supported by rapid reduction of doubly substituted conjugated alkenes which were fully converted within 1–1.5 hours using only 1 mol % catalyst (entries 16–18); also diphenylacetylene was effectively doubly reduced (entry 19).

**Table 1:** Catalytic alkene hydrogenation.^[a]^

Entry	Catalyst	mol %	Substrate	H_2_ [bar]	*T* [°C]	*t* [h]	Product(s)	Conv.^[b]^ [%]
1	2‐Mg	10	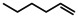	6	120	24	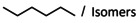	11/1^[c]^
2	1‐Mg	10		6	120	24		4/1^[c]^
3	2‐Ca	10		6	120	24		45/42^[c]^
4	1‐Ca	10		6	120	3		99
5	2‐Sr	10		6	120	24		78/19^[c]^
6	1‐Sr	10		6	120	0.5		99
7	2‐Ba	10		6	120	24		98^[c]^
8	1‐Ba	10		6	120	0.5		99
9	1‐Ba	10		6	60	6		99
10	1‐Ba	10		6	25	24		10/1^[c]^
11	1‐Ba	10		1	120	24		10/83^[c]^
12^[d]^	1‐Ba	1		6	120	4		99
13^[e]^	BaN′′_2_	10		6	120	24		42/58
								
14	1‐Ba	10	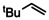	6	120	2	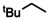	99
15	1‐Ba	10	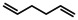	6	120	2	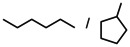	84/16^[c]^
16	1‐Ba	1	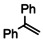	6	120	1.5	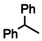	99^[c]^
17	1‐Ba	1	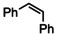	6	120	1	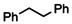	99
18	1‐Ba	1	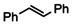	6	120	1	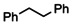	99^[c]^
19	1‐Ba	10	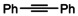	6	120	1	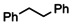	99^[c]^
								
20	1‐Ba	10	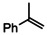	6	120	0.5	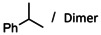	74/26^[c]^
21^[e]^	BaN′′_2_	10		6	120	0.25		99
22	1‐Ba	1		6	120	0.5		67/33^[c]^
23	1‐Ca	1		6	120	4		98/2^[c]^
								
24^[d]^	1‐Ba	1	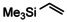	6	120	0.5	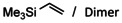	81/16^[c,f]^
25	1‐Ba	10		6	120	0.5		90/10^[c]^
26	1‐Ba	10		6	60	2		99^[c]^
								
27	1‐Ba	1	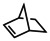	6	120	0.5	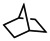	99
								
28	1‐Ca	10	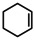	6	120	24	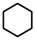	51
29	1‐Sr	10		6	120	10		99
30	1‐Ba	10		6	120	3		99
31	1‐Ba	5		6	120	24		26
								
32	1‐Ba	10	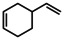	6	120	3.5	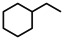	99
33	1‐Ba	10	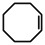	6	120	1.5	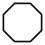	99
34	1‐Ba	10	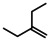	6	120	24	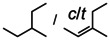	87/13^[c]^
35	1‐Ba	10	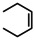	6	120	7	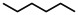	99
36	1‐Ba	10	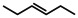	6	120	22	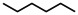	99
37	1‐Ba	10	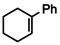	6	120	1	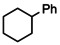	99
38^[g]^	1‐Ba	10	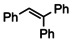	6	120	1	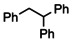	99^[c]^
39	1‐Ba	10	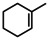	6	120	24	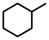	81^[c]^
40^[g]^	1‐Ba	10	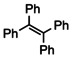	6	120	24	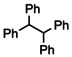	73^[c]^
41^[h]^	1‐Ba	20	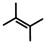	20	120	24	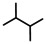	14^[c]^

[a] General catalytic reaction conditions: [substrate]_0_=0.5 m in C_6_D_6_ (1 mL); reaction times for essentially full conversion (99 %) have been optimized in 0.5 h‐steps; for non‐quantitative reactions, the conversion after 24 h is given. [b] Conversion was determined from a ^1^H NMR spectrum of the crude reaction mixture by integration of significant signals of alkane product and substrate, where applicable; GC/MS was used for further product identification (see ESI for experimental details). [c] Conversion and product distribution have been determined by GC/MS analysis. [d] Reaction conducted with [alkene]_0_=1 m in C_6_D_6_ (1 mL). [e] Data taken from Ref. 4. [f] The product mixture contained traces (<3 %) of unidentified species. [g] Reaction run at [alkene]_0_=0.25 m in C_6_H_6_ (1 mL). [h] C_6_H_6_ (1 mL) used as solvent in order to prevent D incorporation in the product.

One of the shortcomings of these superbulky catalysts became clear while reducing substrates sensitive towards oligomerization. α‐Methylstyrene is rapidly converted by **1**‐Ba but gave considerable quantities of dimer (entry 20); cf. BaN′′_2_ gave under similar conditions clean conversion (entry 21). This is explained by the fact that trapping of the benzylbarium intermediate by N′′H (Scheme 2 a) is much faster than by HN(TRIP)_2_ which due to steric hindrance is very hard to deprotonate. Especially at low catalyst concentrations, and thus low free amine concentrations, alkene dimerization is an issue (entry 22). However, using the less active Ca catalyst **1**‐Ca led to full conversion with only traces of dimer (entry 23). Also for hydrogenation of the semi‐activated alkene (Me_3_Si)C(H)=CH_2_ dimeric products were observed, especially at low catalyst loading. In this case dimerization could be fully prevented by lowering the temperature (entries 24–26).

Norbornene, a semi‐activated alkene, is converted twenty times faster to norbornane with **1**‐Ba than with BaN′′_2_ (entry 27). We demonstrate a first case for hydrogenation of the very challenging substrate cyclohexene with activities rapidly increasing down the group: **1**‐Ca <**1**‐Sr <**1**‐Ba (entries 28–30). For comparison, BaN′′_2_ 5 and the dicationic Ca hydride complex **II** 8 gave nill conversion. The catalyst loading for **1**‐Ba could be lowered to 5 mol % (entry 31). Also 4‐vinylcyclohexene was fully reduced (entry 32). Ring enlargement to cyclooctene increased the conversion rate (entry 33).

Alkenes with heteroatoms that potentially coordinate to the Ae metal posed problems. The most active Ba catalyst **1**‐Ba did not convert 3,4‐dihydropyran but instead formed a stable 1/1 complex (Figure S55) suggesting that Ba‐ether coordination blocks free coordination sites at Ba killing any further reactivity with H_2_.

Catalyst **1**‐Ba even converted acyclic, unactivated, internal alkenes (entries 34–36). As expected, reduction of *cis*‐3‐hexene is faster than *trans*‐3‐hexene but 1,1‐Et_2_C=CH_2_, which should be reduced more facile, gave partial isomerisation to a trisubstituted alkene which could only be slowly reduced further. We were, however, able to hydrogenate 1‐Ph‐cyclohexene (entries 37–38) and, most impressively, also the cyclic unactivated trisubstituted alkene: 1‐Me‐cyclohexene (entry 39). The superb performance of **1**‐Ba is further highlighted by the hydrogenation of tetraphenylethylene (entry 40), a most challenging substrate (catalyst **IV** gave stoichiometric conversion).^10^ The limitation of the highly active catalyst **1**‐Ba is set by the tetraalkylated alkene 2,3‐dimethyl‐2‐butene which was reduced in small quantities (entry 41). It should, however, be mentioned that reduction of this substrate is even for platinum group catalysts highly challenging.^43^

### Arene hydrogenation

While the dearomatization of arenes by hydrogenation is quite a challenge, first attempts in group 2 metal catalyzed arene hydrogenation focused on anthracene in which particularly the central ring is activated for reduction (Table 2; see Table S4 for a more extensive list including additional substrates and different catalysts). Even simple AeN′′_2_ complexes are able to reduce anthracene, the activity increasing from Ca to Ba (entries 1–3). Reduction of the central ring is preferred and only slight reduction in the terminal rings was observed. Again, the bulkier amides **1**‐Ae (Ca, Sr, Ba) are clearly more active (entries 4–6); the most active catalyst **1**‐Ba gave after 2.5 hours nearly quantitative yield. Complex BaN′′_2_ was also able to reduce the more challenging substrate naphthalene (entry 7). While bulky **1**‐Ca is inactive, the **1**‐Sr and **1**‐Ba catalysts quantitatively hydrogenated one of the rings in naphthalene with conversion times as short as two hours (entries 8–10). The most active catalyst **1**‐Ba was able to hydrogenate alkylated PAH′s like 9‐Me‐anthracene and 1‐Me‐naphthalene (entries 11–12), which are deactivated for reduction by electronic effects.^44^ The preference for the hydrogenation of non‐alkylated rings is evident from the 80/20 ratio observed for reduction of 1‐Me‐naphthalene. Hydrogenation of phenanthrene gave a mixture of four isomers but extending the reaction time reduced this to only two products (entry 13–14). Using the most active catalyst **1**‐Ba, biphenyl was hydrogenated within ten hours leaving the second ring intact. The activity increases with metal size Ca < Sr < Ba (entries 15–17). Hydrogenation of 1,3,5‐triphenylbenzene gave exclusively reduction of the highly activated central ring producing the two possible diastereomers (entry 18).

**Table 2:** Catalytic arene hydrogenation; [arene]_0_=0.5 mmol, Ae metal catalyst (10 mol %), C_6_H_6_ (1 mL), H_2_ (12 bar), 120 °C.

Entry	Catalyst	Substrate	*T* [h]	Product(s)	Conv.^[a]^ [%]
1^[b]^	CaN′′_2_	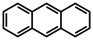	24	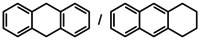	44/1
2^[b]^	SrN′′_2_		24		97/2
3^[b]^	BaN′′_2_		24		95/3
4^[b]^	1‐Ca		24		83/2
5^[b]^	1‐Sr		24		94/3
6^[b]^	1‐Ba		2.5		94/2
					
7	BaN′′_2_	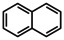	24	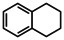	53
8	1‐Ca		24		7
9	1‐Sr		24		99
10	1‐Ba		2		99
					
11^[b]^	1‐Ba	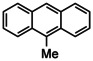	24	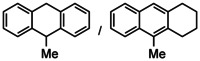	84/15
12	1‐Ba	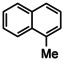	24	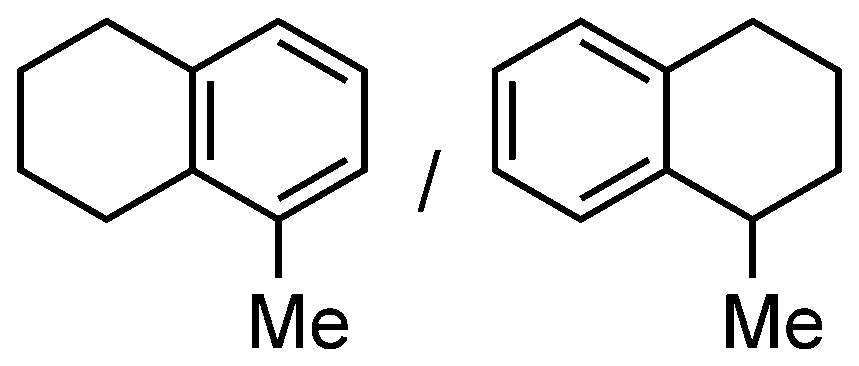	80/20
					
13	1‐Ba	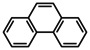	24	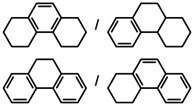	42/32 15/11
					
14^[c]^	1‐Ba	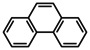	48	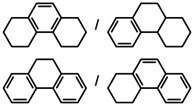	51/49 0/0
					
15	1‐Ca	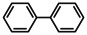	24	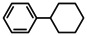	18
16	1‐Sr		24		68
17	1‐Ba		10		99
					
18^[b]^	1‐Ba	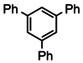	24	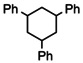	99
19^[d]^	1‐Ba	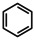	72	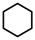	18^[e]^

[a] Conversion and product distribution were determined by GC/MS analysis; NMR spectroscopy was also used to corroborate the product identification. [b] Reaction conducted with [arene]_0_=0.25 m in C_6_H_6_ (1 mL). [c] Reaction at 20 bar H_2_. [d] Reaction performed with 2.5 mol % **1**‐Ba at 140 °C under 50 bar H_2_. [e] Conversion determined by quantitative ^13^C{^1^H} NMR spectroscopy.

These results demonstrate that group 2 metal catalysts, in particular **1**‐Ba, are well able to hydrogenate highly stable aromatic substrates provided the rings are conjugated. Although conversion of isolated aromatic rings was not observed, we found in longer runs with the most active hydrogenation catalyst **1**‐Ba always minor quantities of cyclohexane. The latter is formed by hydrogenation of the benzene solvent. Since benzene‐to‐cyclohexane conversion is an important industrial bulk process, we attempted benzene hydrogenation under more stringent conditions of 50 bar H_2_ and 140 °C, that is, conditions similar to the IFP process for large‐scale cyclohexane production.^45^ Using only 2.5 mol % **1**‐Ba we found that after three days 18 % of the benzene solvent was converted to cyclohexane (entry 19). Although the Ba catalyst is not as efficient as Ni/Al catalysts, the current result is a most striking demonstration that also unactivated (non‐conjugated) aromatic rings like benzene, which are generally very hard to reduce,^46^ can be hydrogenated with group 2 metal catalysts.

### Mechanistic considerations

In contrast to catalytic studies with well‐defined Ae metal hydride catalysts,^3^, ^9^, ^10^ it is extremely difficult to identify the “true” catalyst in Ae metal amide catalyzed hydrogenation.^20^ Borders between homogeneous molecular catalysis and heterogeneous nanoparticle catalysis are starting to disappear and it is often extremely hard, or even impossible, to establish the nature of the catalyst. It is even likely that numerous catalytically active species operate in concert.^47^ Building upon our previous work in alkene hydrogenation,^4^, ^5^ it is reasonable to propose the initial formation of monomeric (amide)AeH species.

In order to increase our understanding on possible catalytically active species, a solution of **1**‐Ae (Ca or Ba) in C_6_D_6_ was reacted with H_2_ (1 bar, 60–90 °C) and monitored by ^1^H NMR. The appearance of signals for HN(TRIP)_2_ and very broad resonances in the amide and hydride regions suggested the formation of undefined hydride clusters. Reaction of **1**‐Ca with H_2_ is much slower than hydrogenolysis of CaN′′_2_. At 1 bar H_2_ and 60 °C only 50 % conversion was reached in 50 hours. Hydrogenolysis of **1**‐Ba was considerably faster (1 bar H_2_, 90 °C, 100 % conversion, 5 h). As discussed previously, this contrathermodynamic reaction is possible due to aggregation which releases energy. All attempts to crystallize defined metal hydride clusters from reaction of **1**‐Ae with H_2_ failed. This is partially due to the extreme solubility of these species induced by the multiple *i*Pr substituents.

The effect of ligand size on cluster size was investigated by DOSY NMR using the external calibration method described by Stalke and co‐workers (see Supporting Information).^48^ For **1**‐Ca and **1**‐Ba in benzene we found in both cases molecular weights lower than calculated for the monomers (**1**‐Ca: calc. 697, found 515; **1**‐Ba: calc. 795, found 548). Using a different calibration method, Mills and co‐workers reported a molecular weight of 1000 for **1**‐Ca in benzene.^33^ It is possible that the nature of the ligand, which is very rich in H, may cause systematic errors. DOSY measurements on a solution of **1**‐Ca converted with H_2_ gave species with a molecular weight up to circa 1700 g mol^−1^. For reaction mixtures of **1**‐Ba with H_2_ clusters up to a molecular weight of circa 3300 g mol^−1^ have been observed. The higher aggregation for the Ba hydride species is in line with the fact that larger metal cations tend to form larger aggregates. However, both clusters are considerably smaller than those obtained from reaction of CaN′′_2_ with H_2_ (7500 g mol^−1^),^20^ supporting the idea that bulky amide ligands give rise to smaller catalytic species in higher concentrations.

Mechanistic understanding was improved by various DFT studies using the B3PW91/def2tzvpp method including correction for dispersion (GD3BJ) and solvent (PCM=benzene); Ba was described by pseudopotentials (see ESI for details).

In a first set of calculations we investigated the effect of amide bulk on aggregation by optimization of (R_2_NCaH)_*x*_ species (*x=*1, 2 or 4) with increasing amide size Me_2_N < (Me_3_Si)_2_N < (TRIP)_2_N (Table S9 and Scheme S4). Monomer→dimer conversion is independent on amide bulk and in all cases exothermic by circa Δ*H*=−30 kcal mol^−1^. However, the enthalpies for monomer→tetramer conversion decrease with increasing amide size; Δ*H* in kcal mol^−1^: Me_2_N −87.7, (Me_3_Si)_2_N −72.4 and (TRIP)_2_N −39.6. As aggregation causes considerable entropy loss the Δ*G* values are less negative (Me_2_N −55.8, (Me_3_Si)_2_N −21.6) while formation of [(TRIP)_2_NCaH]_4_ is even endergonic by Δ*G*=+8.2 kcal mol^−1^. This clearly supports the observation that bulkier amides reduce cluster size leading to increased catalyst concentration.

**Scheme 4:**
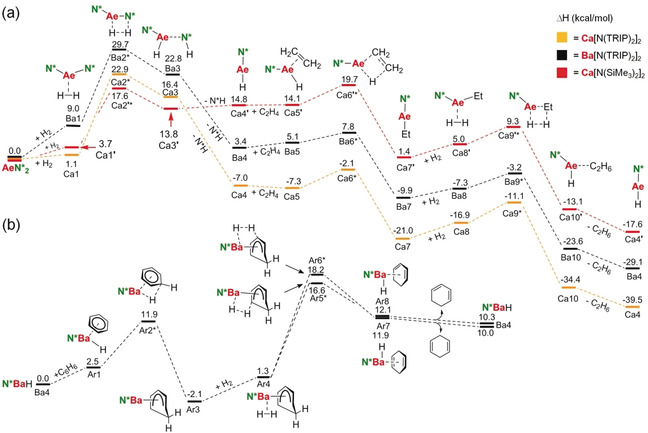
Energy profiles (Δ*H* in kcal mol^−1^) for a) the hydrogenation of ethylene by catalysts **1**‐Ca (orange), **1**‐Ba (black) and CaN′′_2_ (red), and b) benzene hydrogenation by **1**‐Ba; B3PW91/def2tzvpp including correction for dispersion (GD3BJ) and solvent (PCM=benzene).

The second set of calculations aimed at understanding the influence of amide bulk and the metal on alkene hydrogenation. The pathway for catalytic ethylene hydrogenation with **1**‐Ca and **1**‐Ba catalysts is shown in Scheme 4 a. For comparison, we also recalculated the previously reported pathway for CaN′′_2_ using this method. The barrier for hydride formation by reaction of **1**‐Ca with H_2_ is higher (Ca2*: 22.9 kcal mol^−1^) than that for the reaction of CaN′′_2_ with H_2_ (17.6 kcal mol^−1^). This is in agreement with experimental observation and likely related to unusually high steric stress in the superbulky amide complex. Steric stress in **1**‐Ca is also reflected by its exothermic conversion to (TRIP)_2_NCaH (Ca4: −7.0 kcal mol^−1^) while reaction of CaN′′_2_ to N′′CaH is endothermic (Ca4′: +14.8 kcal mol^−1^).^4^ The activation energy for formation of (TRIP)_2_NBaH from **1**‐Ba is higher and not in agreement with the faster reaction of **1**‐Ba with H_2_. This is likely due to insufficient modeling of solvent effects for the highly unsaturated species (TRIP)_2_NBaH by the PCM method.

The activation energy for alkene insertion for the Ba catalyst (Ba5 → Ba6*: +2.7 kcal mol^−1^) is lower than that for the Ca catalyst (Ca5 → Ca6*: +5.2 kcal mol^−1^). Also the reaction of the ethyl metal intermediate with H_2_ has a lower barrier for Ba (Ba7 → Ba9*: +6.7 kcal mol^−1^) than for Ca (Ca7 → Ca9*: +9.9 kcal mol^−1^). The very shallow energy profile for the Ba catalyst is in line with its much higher activity and excellent performance in alkene hydrogenation. Comparison of the profiles for both Ca catalysts, N′′CaH and (TRIP)_2_NCaH, shows that the bulk of the remaining amide ligand has only a minor influence on ethylene hydrogenation. However, for the less bulky catalyst N′′CaH deactivation by the reverse reaction with N′′H back to CaN′′_2_ (Ca4′ → Ca2′*: +2.8 kcal mol^−1^) is easier than ethylene insertion (Ca4′ → Ca6′*: +4.9 kcal mol^−1^). In contrast, reaction of bulky (TRIP)_2_NCaH with (TRIP)_2_NH is extremely difficult (Ca4 → Ca2*: +29.9 kcal mol^−1^) and ethylene insertion is facile. It is therefore unlikely that formation of the catalyst (TRIP)_2_NCaH is an equilibrium. This may also explain the much higher activity of **1**‐Ca compared to CaN′′_2_.

While the monomer R_2_NAeH may not be the best model system for larger aggregates, the hydride‐alkene insertion step was additionally calculated for a dimeric catalyst (Scheme S2). In line with the higher reactivity of monomeric metal hydrides, these dimeric catalysts show somewhat higher activation enthalpies for ethylene insertion (Δ*H* in kcal mol^−1^): [(TRIP)_2_NCaH]_2_ +9.9, [(TRIP)_2_NBaH]_2_ +9.9 and [N′′CaH]_2_ +14.6. In this case the Ca catalyst with the bulkier amide (TRIP)_2_N is more reactive than that with N′′.

In a third set of calculations the pathway for benzene hydrogenation with the most active (TRIP)_2_NBaH catalyst has been evaluated (Scheme 4 b). The cycle starts with benzene complexation followed by formation of a Meisenheimer anion which is surprisingly exothermic (Ar3: −3.5 kcal mol^−1^); breaking benzene's aromaticity by formation of the Meisenheimer anion is generally endothermic.^5^, ^29^, ^49^ Two possible transition states for the reaction with H_2_ have been located (Ar5* and Ar6*). The transition state for formation of 1,3‐cyclohexadiene (Ar5*) is 1.6 kcal mol^−1^ lower in energy than that for 1,4‐cyclohexadiene formation (A6*). The activation energies of +18.7 and +20.3 kcal mol^−1^ are considerably higher than those for alkene hydrogenation. This is in line with the experimentally very challenging reduction of aromatic substrates. Note that the first step, benzene‐to‐cyclohexadiene reduction, is endothermic by circa +10 kcal mol^−1^ but further hydrogenation to cyclohexane is overall exothermic^50^ and should be facile.

## Conclusion

We introduced two sets of bulky Ae metal amide complexes, **1**‐Ae and **2**‐Ae (Ae=Mg, Ca, Sr, Ba) that are monomeric in the solid state. All **1**‐Ae complexes with the bulkier N(TRIP)_2_ ligand show highly linear coordination geometries while **2**‐Ae complexes with the N(TRIP)(DIPP) ligand are bent and reveal shorter Ae−N bonds.

The bulk of the amide ligand has a tremendous effect on alkene hydrogenation which is likely best illustrated by entries 8 and 13 in Table 1. The catalyst activities increase with the size of the amide ligand along the series AeN′′_2_ <**2**‐Ae <**1**‐Ae and with metal size Mg < Ca < Sr < Ba. The most active catalyst **1**‐Ba clearly extended the substrate scope for *s*‐block metal catalyzed alkene hydrogenation. Highly active **1**‐Ba is able to reduce unactivated internal alkenes like cyclohexene or 3‐hexene but also hydrogenation of highly challenging substrates like 1‐Me‐cyclohexene (a trisubstituted double bond) or tetraphenylethylene could be achieved. A drawback for the bulky amide catalysts is the enhanced oligomerization found for activated alkenes like styrene. In this case less bulky AeN′′_2_ catalysts would be preferred.

The very high activity of bulky amide catalysts was further demonstrated by high activities in arene hydrogenation which hitherto was never achieved with group 2 metal catalysts. While PAH′s with extended π‐systems could be routinely reduced to products with at least one aromatic ring, hydrogenation of the remaining aromatic 6π‐electron system is difficult and also for transition metal catalysts a challenge.^43^ However, during hydrogenation catalysis using our most active catalyst **1**‐Ba in benzene, generally traces of cyclohexane were found. Under more forcing conditions benzene could be partially hydrogenated to cyclohexane. This first main group metal catalyzed benzene hydrogenation illustrates the enormous potential of the heavier Ba catalysts.

Since Ae metal hydride species have strong tendencies to aggregate to complicated mixtures of species, the nature of the catalyst remains unclear. DOSY NMR measurements show that the much higher activity of superbulky Ae amide catalysts may be explained by the lower aggregation numbers for these bulky systems. This is supported by DFT calculations which demonstrate that aggregation of R_2_NCaH species becomes less favorable with increasing amide size: Me_2_N < N′′ < (TRIP)_2_N. Calculations also show that catalyst generation may play a role: for smaller amides like N′′ the equilibrium, AeN′′_2_ + H_2_ ⇌ N′′AeH + N′′H, lies to the left. The reaction of **1**‐Ae with H_2_ to give (TRIP)_2_NAeH is much slower but once formed the reverse catalyst deactivation, that is, deprotonation of sterically hindered (TRIP)_2_NH, is unlikely.

The herein described catalytic reduction of highly challenging alkenes and arenes by superbulky Ae metal amides is unrivalled. These results demonstrate that current state‐of‐the‐art group 2 metal hydrogenation catalysis starts to reach a similar level as traditional transition metal catalysis. It should be considered highly remarkable that stable aromatic molecules like benzene submit to group 2 metal power.^51^ We currently aim to increase catalyst activities and applications even further.

## Conflict of interest

The authors declare no conflict of interest.

## Supporting information

As a service to our authors and readers, this journal provides supporting information supplied by the authors. Such materials are peer reviewed and may be re‐organized for online delivery, but are not copy‐edited or typeset. Technical support issues arising from supporting information (other than missing files) should be addressed to the authors.

Supplementary
