# Supplementary material for: Highly Active Superbulky Alkaline Earth Metal Amide Catalysts for Hydrogenation of Challenging Alkenes and Aromatic Rings
Source: Angew Chem Int Ed Engl. 2020 Mar 27;59(23):9102–12. doi: 10.1002/anie.202001160 (PMC7318605; doi:10.1002/anie.202001160)
Supplement: Supplementary file 1 — Supplementary [file ANIE-59-9102-s001.pdf]

Angewandte Chemie
Eine Zeitschrift der Gesellschaft Deutscher Chemiker

Supporting Information

Highly Active Superbulky Alkaline Earth Metal Amide Catalysts for Hydrogenation of Challenging Alkenes and Aromatic Rings

*Johannes Martin, Christian Knüpfer, Jonathan Eyselein, Christian Färber, Samuel Grams, Jens Langer, Katharina Thum, Michael Wiesinger, and Sjoerd Harder**

anie_202001160_sm_miscellaneous_information.pdf

Table of Contents

1	General Experimental Procedures	S1
2	Synthetic, Spectroscopic and Analytical Data for New Compounds	S4
3	Crystal Structure Determination	S48
4	Preparation of Pre-Catalysts	S74
5	Catalytic Hydrogenation of Alkenes and Alkynes	S82
6	Catalytic Hydrogenation of Arenes	S181
7	Catalytic Hydrogenation of Benzene	S252
8	Reactions of $Ae[N(Si^{i}Pr_3)_2]_2$ with H_2	S256
9	Diffusion Ordered Spectroscopy	S261
10	Theoretical Calculations	S266
11	References	S382

1 General Experimental Procedures

All air- and moisture-sensitive manipulations were carried out in dry glassware under an atmosphere of N_2 using standard Schlenk techniques or in a glovebox (MBraun, Labmaster SP). Solvents (benzene, toluene, *n*-pentane, *n*-hexane and diethyl ether) were degassed with N_2, dried over activated aluminum oxide (Solvent Purification System: Pure Solv 400-4-MD, Innovative Technology) and stored over molecular sieves (3 Å) under an inert atmosphere. Deuterated benzene (C_6D_6; 99.6+% D), [d_8]toluene (99.6+% D), [d_8]THF (99.5+% D) and [d_{12}]cyclohexane (99.5+% D) were purchased from Sigma Aldrich or Deutero GmbH and dried over molecular sieves (3 Å) before use.

The following compounds were synthesized according to previously reported procedures: potassium *bis*(tri*iso*propylsilyl)amide $[KN(Si^iPr_3)_2]_n$ **(1-K)**,[S1] HN(DIPP)(Si^iPr_3) **(1-H)**[S2] and *bis*(*para*-*tert*-butylbenzyl)calcium.[S3] Alkaline earth metal iodides AeI_2 (Ae = Mg, Ca, Sr, Ba) were prepared by reactions of the respective excessive metal with iodine in boiling THF followed by continuous extraction with hot THF, solvent evaporation and drying in high vacuum at 250 °C. Potassium hydride was obtained from Sigma Aldrich as a dispersion in mineral oil, filtered, washed several times with hexane and dried *in vacuo* prior to use. Other commercially available reagents were used as received.

Multinuclear NMR spectra were recorded on a Bruker Avance III HD 400 MHz or 600 MHz spectrometer at ambient temperature. Chemical shifts δ are reported in *parts per million* (ppm) relative to Me_4Si as external standard and referenced internally to the carbon nuclei ($^{13}C\{^1H\}$) or residual *protio*-solvent resonances (^1H) of the deuterated solvents. $^{29}Si\{^1H\}$ NMR spectra were referenced using Me_4Si as external standard. Assignments of resonance signals in the ^1H and $^{13}C\{^1H\}$ NMR spectra were made based on two-dimensional NMR correlation (HSQC, HMBC, COSY) and ATP experiments. Coupling constants J are given in Hertz (Hz). Signal multiplicities are abbreviated as follows: s (singlet), d (doublet), sept (septet), m (multiplet) and br (broad).

Infrared spectra were acquired on a Bruker Alpha II FT-IR spectrometer equipped with a Platinum ATR diamond from the neat compounds under inert conditions inside a glovebox. All spectra were recorded at room temperature in the range of 400 – 4000 cm^{-1} with a resolution of 4 cm^{-1} and baseline corrected. Wavenumbers are given in cm^{-1} and intensities of IR bands are described using the following terms: s = strong, m = medium and w = weak.

Crystal structures have been measured on a SuperNova (Agilent) diffractometer with dual Cu and Mo microfocus and an Atlas S2 detector. Elemental analyses were performed with a Hekatech Eurovector EA3000 analyzer. A Biotage Initiator+ microwave synthesizer equipped with a Robot Eight autosampler was used for microwave-heated reactions.

GC-MS measurements were performed on a Thermo Scientic™ Trace™ 1310 gas chromatography system (carrier gas: helium) with detection by a Thermo Scientic™ ISQ™ LT single quadrupole mass spectrometer. A Thermo Scientific™ TraceGOLD™ TG-5SilMS GC column or Phenomenex® ZebronTM ZB-5 GC column of the dimensions 0.25 mm x 30 m with a film thickness of 0.25 µm was used. The samples (1 µL) were injected with an instant connect-SSL module in the split mode (injector temperature: 280 °C). Temperature programs were started at 40 °C followed by heating ramps, optimized for the separation problem, until 280 °C. Baseline separation of each analyte was achieved by choosing the different temperature programs. The molecular identities were confirmed by comparison with entries in the NIST/EPA/NIH mass spectral library (v2.2, built June 10 2014).

All catalytic hydrogenation experiments were carried out on a high vacuum Schlenk line attached to a hydrogen gas tank in high-pressure miniature autoclaves (15 mL capacity) made of stainless steel by Amtech, which were previously dried by heating in an oven at 80 °C overnight. Research grade hydrogen gas (H_2; Air Liquide) used in catalytic reactions was of 99.999% purity and used without additional purification. The pre-catalysts $Ae[N(SiMe_3)_2]_2$ (Ae = Ca, Sr, Ba),[S4] $Sr[CH(SiMe_3)_2]_2(THF)_2$[S5] and $(DMAT)_2Sr(THF)_3$[S6] were prepared following literature known procedures.

The commercially available liquid unsaturated substrates 1,1-diphenylethylene (TCI Chemicals, >98%), α-methylstyrene (Acros Organics, 99%), vinyltrimethylsilane (Sigma Aldrich, 97%), 2,3-dimethyl-1,3-butadiene (Alfa Aesar, 98%), *cis*-stilbene (Merck, 97%), norbornene (Acros Organics, 99%), 1-hexene (Acros Organics, 97%), 3,3-dimethylbutene (Sigma Aldrich, >98%), 1,5-hexadiene (Fluka, 97%), cyclohexene (Fluka, 99%), 3,4-dihydro-2*H*-pyran (Alfa Aesar, 99%), *cis*-cyclooctene (ABCR, 95%), 1,5-cyclooctadiene (Acros Organics, 99%), 4-vinyl-1-cyclohexene (TCI Chemicals, >95%), 2-ethyl-1-butene (TCI Chemicals, >97%), *cis*-3-hexene (TCI Chemicals, 97%), 1-phenyl-1-cyclohexene (Sigma Aldrich, 95%), 1-methyl-1-cyclohexene (Alfa Aesar, 96%) and 2,3-dimethyl-2-butene (Alfa Aesar, 97%) were dried by stirring over freshly ground CaH_2 at room temperature overnight, distilled under reduced pressure and stored in an N_2-filled glovebox over activated molecular sieves (3 Å) before usage. 1-Pyrrolidino-1-cyclohexene was synthesized according to a literature procedure and dried over freshly ground CaH_2 before vacuum distilling and storing under an inert atmosphere over molecular sieves

(3 Å).[S7] *Trans*-3-hexene (Alfa Aesar, 98%) and 3-hexyne (Sigma Aldrich, 99%) were dried over molecular sieves (3 Å) in a glovebox. Triphenylethylene (>98%) and tetraphenylethylene (98%) were obtained from Alfa Aesar and used without further purification.

Biphenyl (Sigma Aldrich, >99%) and the polycyclic aromatic hydrocarbons anthracene (Sigma Aldrich, 97%), naphthalene (Sigma Aldrich, 99%), acenaphthylene (TCI Chemicals, >94%), phenanthrene (Alfa Aesar, 98%) and pyrene (Alfa Aesar, 98%) were obtained commercially, sublimed under reduced pressure and stored under an N_2 atmosphere prior to catalytic reactions. 1-Methylnaphthalene was purchased from Alfa Aesar (96%) and dried over freshly ground CaH_2 at room temperature overnight followed by dynamic vacuum distillation and storage over molecular sieves (3 Å) under an inert N_2 atmosphere. 9-Methylanthracene (TCI Chemicals, 98%) and 1,3,5-triphenylbenzene (Sigma Aldrich, 97%) were dried in high vacuum at 60 °C and then stored in an N_2-filled glovebox.

2 Synthetic, Spectroscopic and Analytical Data for New Compounds

Synthesis of Mg[N(Si^{i}Pr$_3$)$_2$]$_2$ (1-Mg)

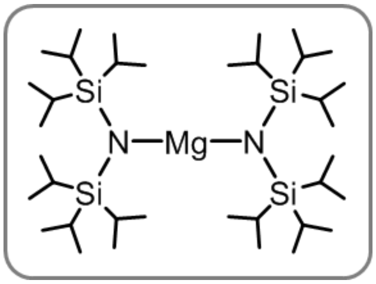

A suspension of [KN(Si^{i}Pr$_3$)$_2$]$_n$ (1.52 g, 4.13 mmol) and MgI_2 (597 mg, 2.15 mmol) in benzene (8 mL) was stirred at 60 °C for five days. After allowing the reaction mixture to cool to room temperature, it was freed from solvent in a dynamic vacuum. The remaining off-white residue was dried *in vacuo* at 60 °C for 30 minutes and then treated with hexane (12 mL). The hexane extract was filtered prior to removing the volatiles from the filtrate under reduced pressure. The resulting colorless solid was dried in vacuum at 60 °C for 30 minutes, dissolved in boiling hexane (4 mL), filtered and gradually cooled to 8 °C to give Mg[N(Si^{i}Pr$_3$)$_2$]$_2$ (986 mg, 1.45 mmol, 67%) as colorless block-like crystals. The mother liquor was decanted, and the crystals washed with cold pentane (–20 °C, 2 x 2 mL), dried in vacuum at room temperature and collected.

^{1}H NMR (600 MHz, C_6D_6, 25 °C): δ_H = 0.96 (sept, ^3J(H,H) = 7.4 Hz, 12H, Si(C*H*Me$_2$)$_3$), 1.29 (d, ^3J(H,H) = 7.5 Hz, 72H, Si[CH(C*H*$_3$)$_2$]$_3$) ppm; **^{13}C{^{1}H} NMR** (151 MHz, C_6D_6, 25 °C): δ_C = 19.8 (s, Si(*C*HMe$_2$)$_3$), 20.5 (s, Si[CH(*C*H$_3$)$_2$]$_3$) ppm; **^{29}Si{^{1}H} NMR** (119 MHz, C_6D_6, 25 °C): δ_{Si} = –1.0 (s) ppm; **FT-IR** (ATR, pure): $\tilde{\upsilon}$ = 2940 (m), 2860 (s), 2760 (w), 1457 (w), 1075 (m), 1051 (s), 1000 (m), 953 (w), 916 (w), 875 (s), 694 (s), 651 (s), 596 (m), 502 (m), 469 (m) cm^{-1}; **Elemental analysis:** calculated (%) for $C_{36}H_{84}MgN_2Si_4$ (681.73 g mol^{-1}): C 63.43, H 12.42, N 4.11; found: C 63.09, H 12.25, N 4.13.

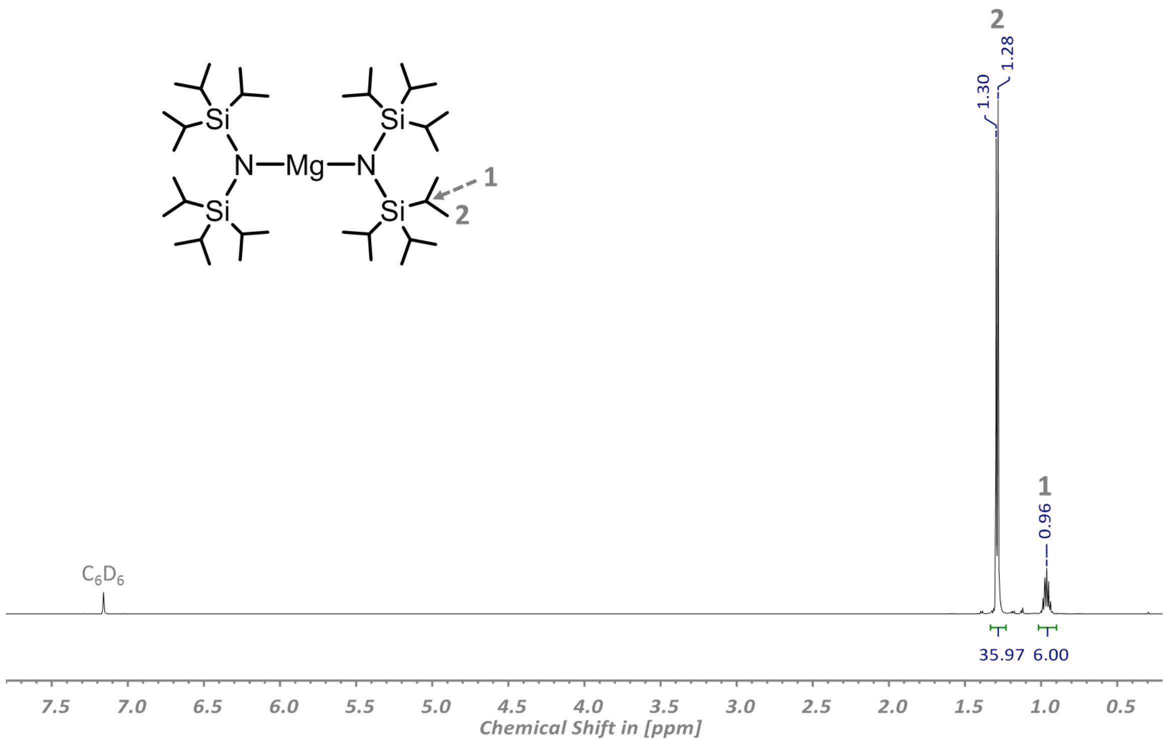

Figure S1. ^{1}H NMR spectrum (600 MHz, C_6D_6, 25 °C) of $Mg[N(Si^iPr_3)_2]_2$ **(1-Mg)**.

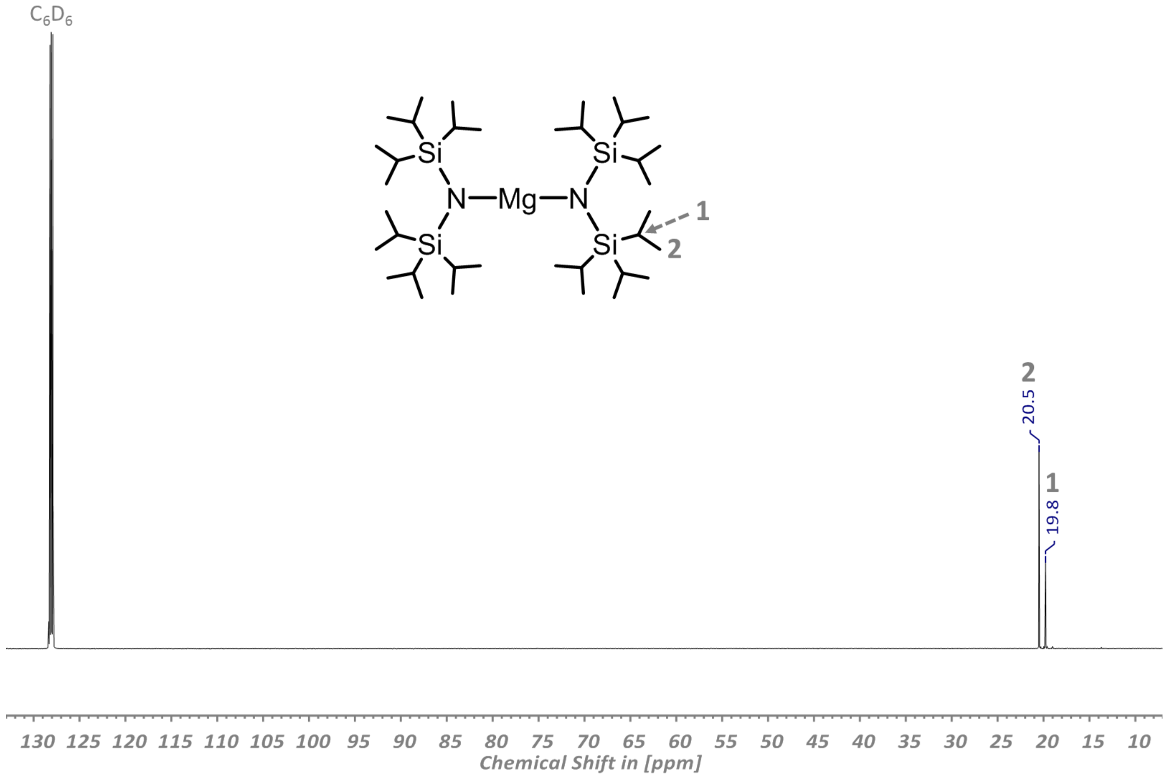

Figure S2. $^{13}C\{^1H\}$ NMR (151 MHz, C_6D_6, 25 °C) of $Mg[N(Si^iPr_3)_2]_2$ **(1-Mg)**.

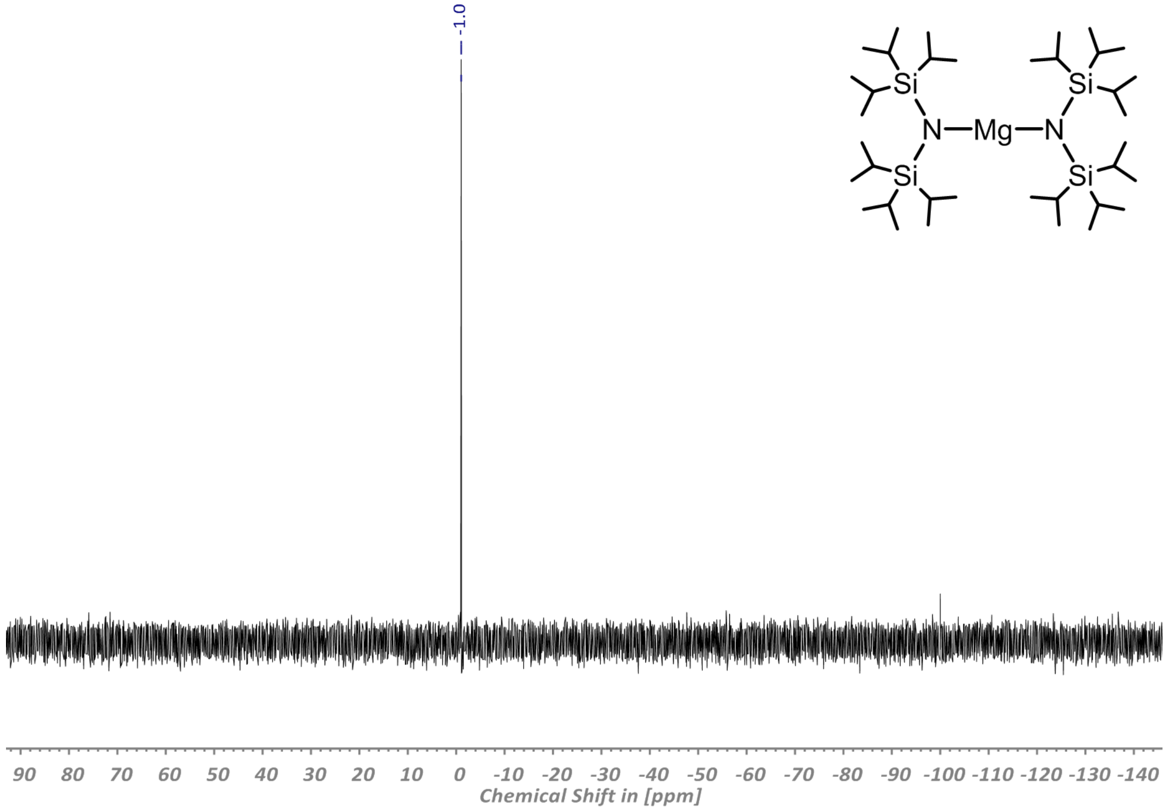

Figure S3. $^{29}Si\{^{1}H\}$ NMR spectrum (119 MHz, C_6D_6, 25 °C) of $Mg[N(Si^{i}Pr_3)_2]_2$ **(1-Mg)**.

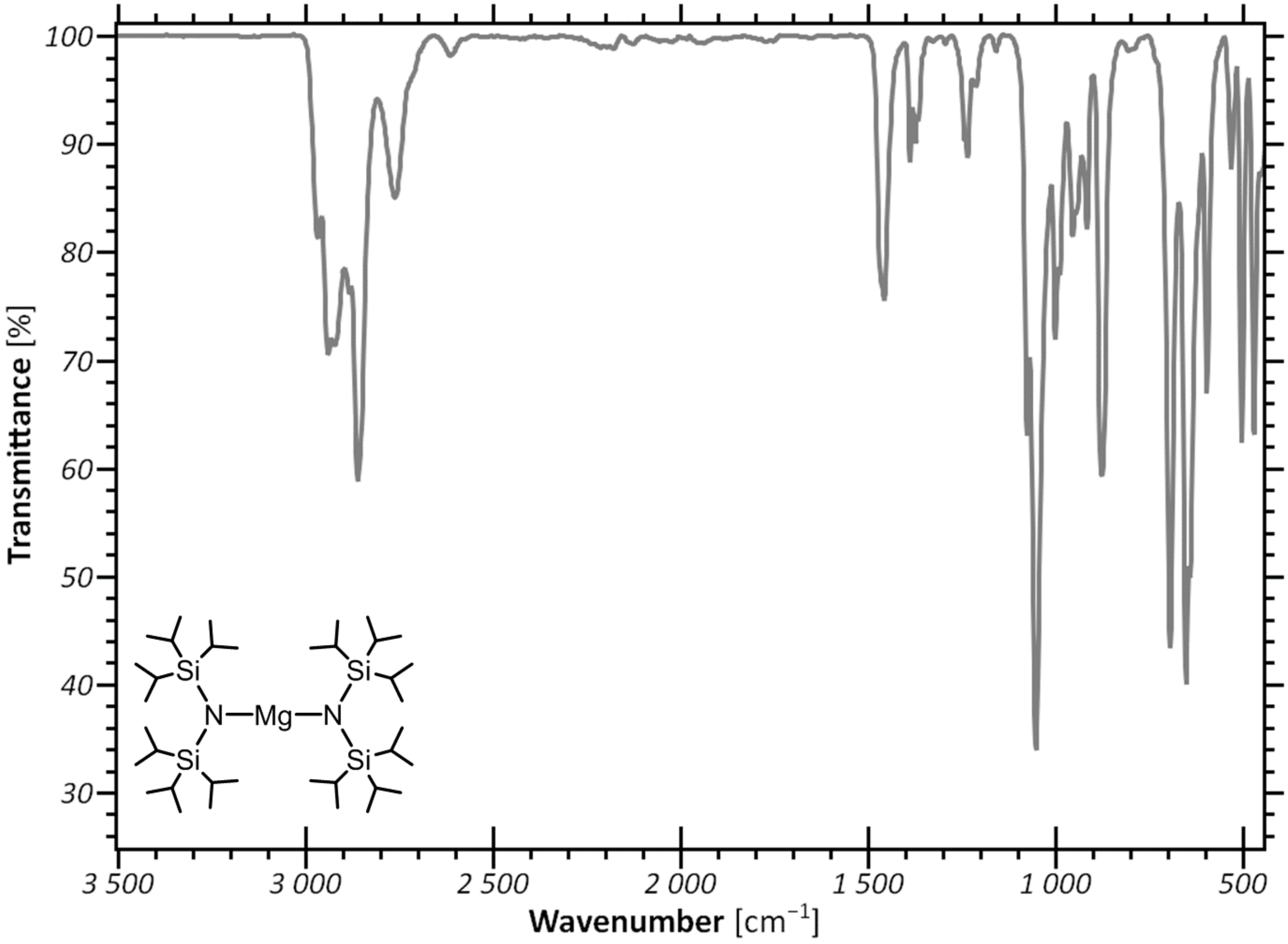

Figure S4. FT-IR ATR spectrum of Mg[N(Si^{i}Pr$_3$)$_2$]$_2$ **(1-Mg)**.

Synthesis of $Ca[N(Si^{i}Pr_3)_2]_2$ (1-Ca)

The synthesis of $Ca[N(Si^{i}Pr_3)_2]_2$ was conducted according to a modified literature procedure.[S8]

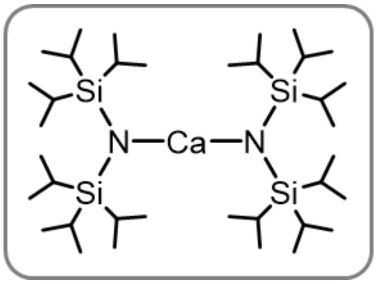

CaI_2 (1.29 g, 4.39 mmol) and $[KN(Si^{i}Pr_3)_2]_n$ (3.23 g, 8.78 mmol) were combined in a Schlenk tube and benzene (20 mL) was added at room temperature forming an off-white suspension. The reaction mixture was heated to 70 °C and stirred over the course of two days. Following solvent evaporation in a dynamic vacuum, the off-white residue was dried *in vacuo* at 60 °C for 30 minutes, extracted with hexane (30 mL) and filtered. Volatiles were removed from the filtrate under reduced pressure to give a colorless solid, which was further dried in vacuum (60 °C, 30 minutes). The residue was re-dissolved in boiling hexane (10 mL) and gradually cooled to 8 °C. Large, well-defined colorless crystals deposited and were collected after decanting the supernatant, washing with cold pentane (–20 °C, 2 x 2 mL) and drying *in vacuo* at room temperature. Further crops of crystals were obtained by repeated concentration of the mother liquor under reduced pressure and cooling to 8 °C to yield $Ca[N(Si^{i}Pr_3)_2]_2$ as colorless crystalline solid (1.74 g, 2.49 mmol) in an overall yield of 57%.

^{1}H NMR (600 MHz, C_6D_6, 25 °C): δ_H = 0.91 (sept, ^3J(H,H) = 7.3 Hz, 12H, Si(C*H*Me$_2$)$_3$), 1.26 (d, ^3J(H,H) = 7.6 Hz, 72H, Si[CH(C*H*$_3$)$_2$]$_3$) ppm; **^{13}C{^{1}H} NMR** (151 MHz, C_6D_6, 25 °C): δ_C = 18.2 (s, Si(*C*HMe$_2$)$_3$), 20.2 (s, Si[CH(*C*H$_3$)$_2$]$_3$) ppm; **^{29}Si{^{1}H} NMR** (119 MHz, C_6D_6, 25 °C): δ_{Si} = –7.7 (s) ppm; **FT-IR** (ATR, pure): $\tilde{\upsilon}$ = 2944 (w), 2861 (m), 1466 (w), 1035 (s), 1001 (w), 877 (m), 696 (s), 651 (s), 598 (w), 508 (m), 484 (m) cm^{-1}; **Elemental analysis:** calculated (%) for $C_{36}H_{84}CaN_2Si_4$ (697.50 g mol^{-1}): C 61.99, H 12.14, N 4.02; found: C 61.65, H 12.19, N 4.39. The spectroscopic and analytical data are in accordance with those previously reported for this compound.[S8]

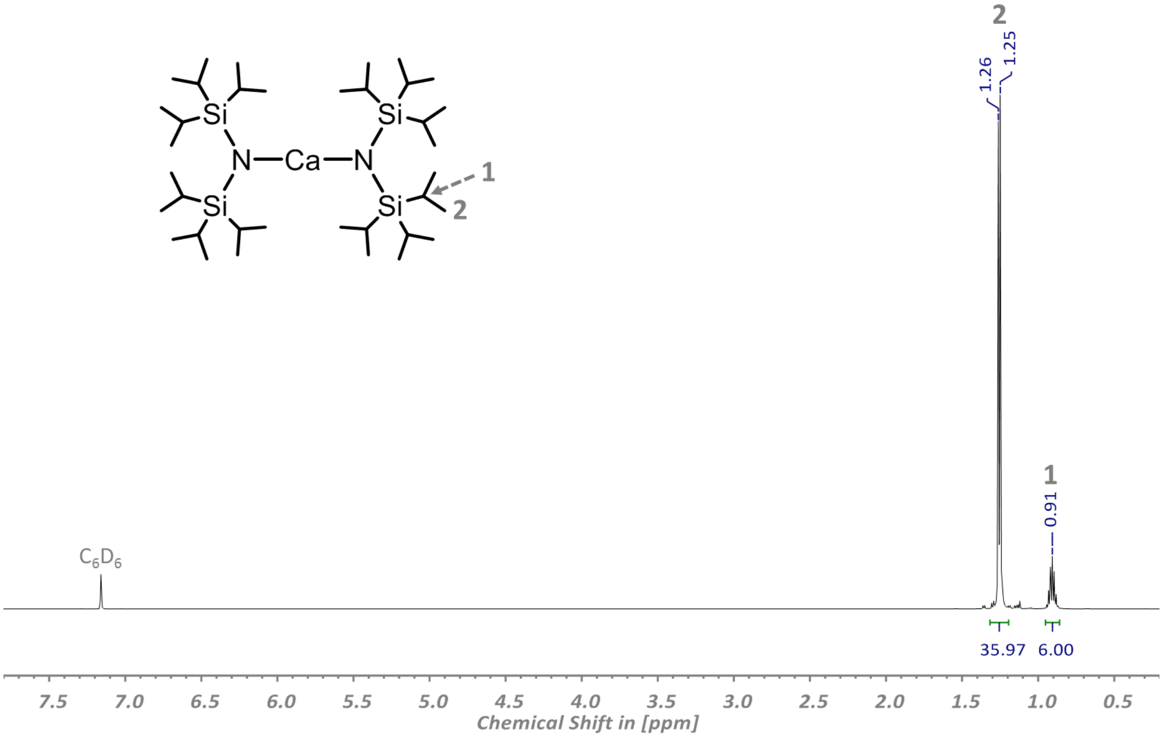

Figure S5. ^{1}H NMR spectrum (600 MHz, C_6D_6, 25 °C) of Ca[N(Si^{i}Pr$_3$)$_2$]$_2$ **(1-Ca)**.

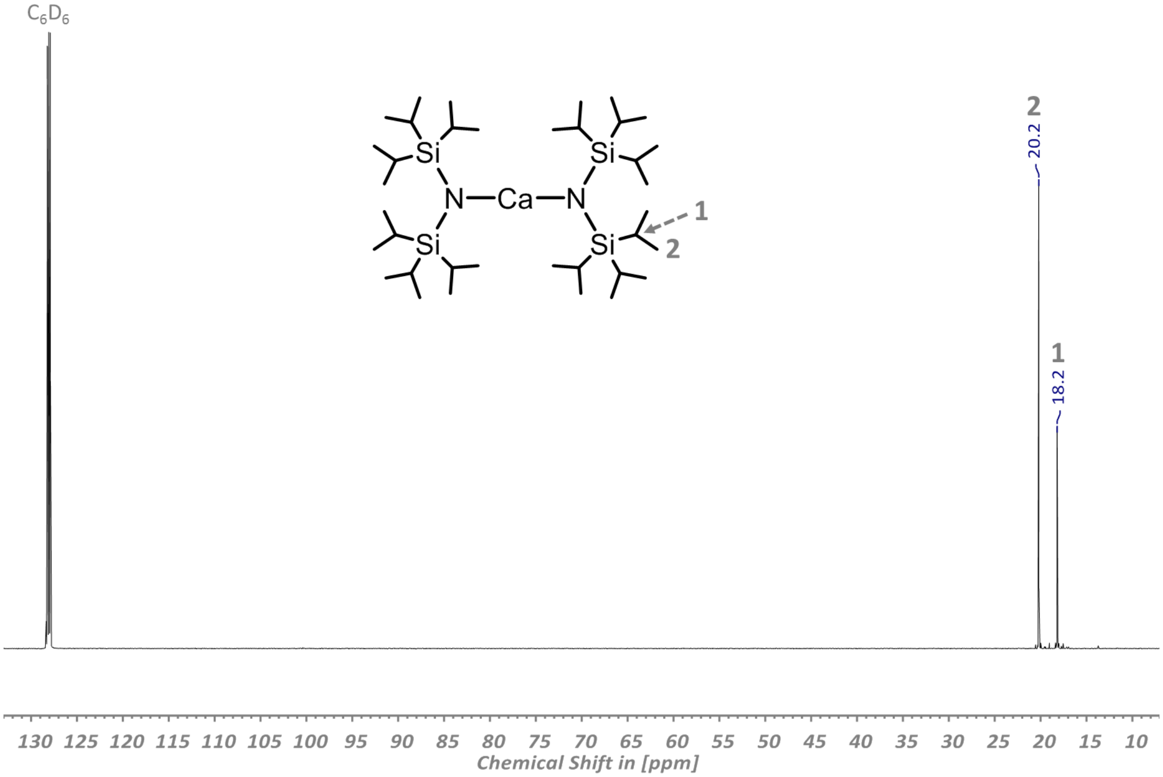

Figure S6. $^{13}C\{^{1}H\}$ NMR (151 MHz, C_6D_6, 25 °C) of Ca[N(Si^{i}Pr$_3$)$_2$]$_2$ **(1-Ca)**.

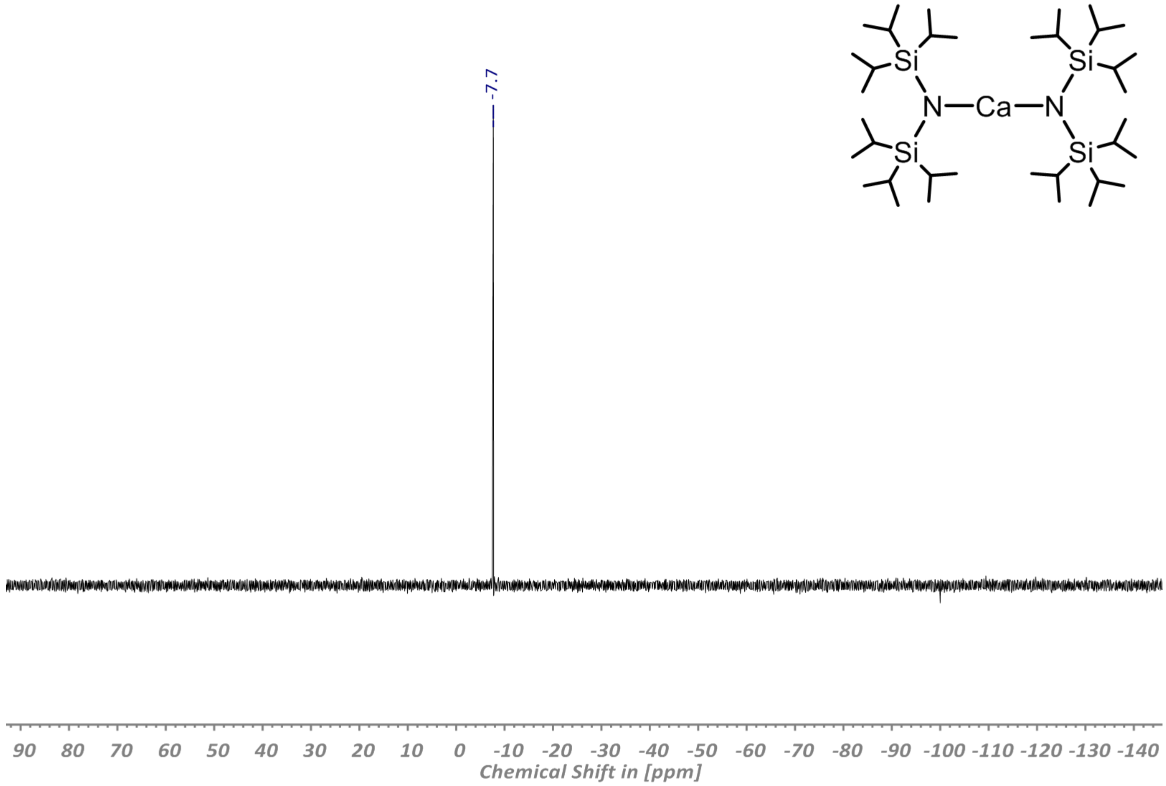

Figure S7. $^{29}Si\{^{1}H\}$ NMR spectrum (119 MHz, C_6D_6, 25 °C) of $Ca[N(Si^{i}Pr_3)_2]_2$ **(1-Ca)**.

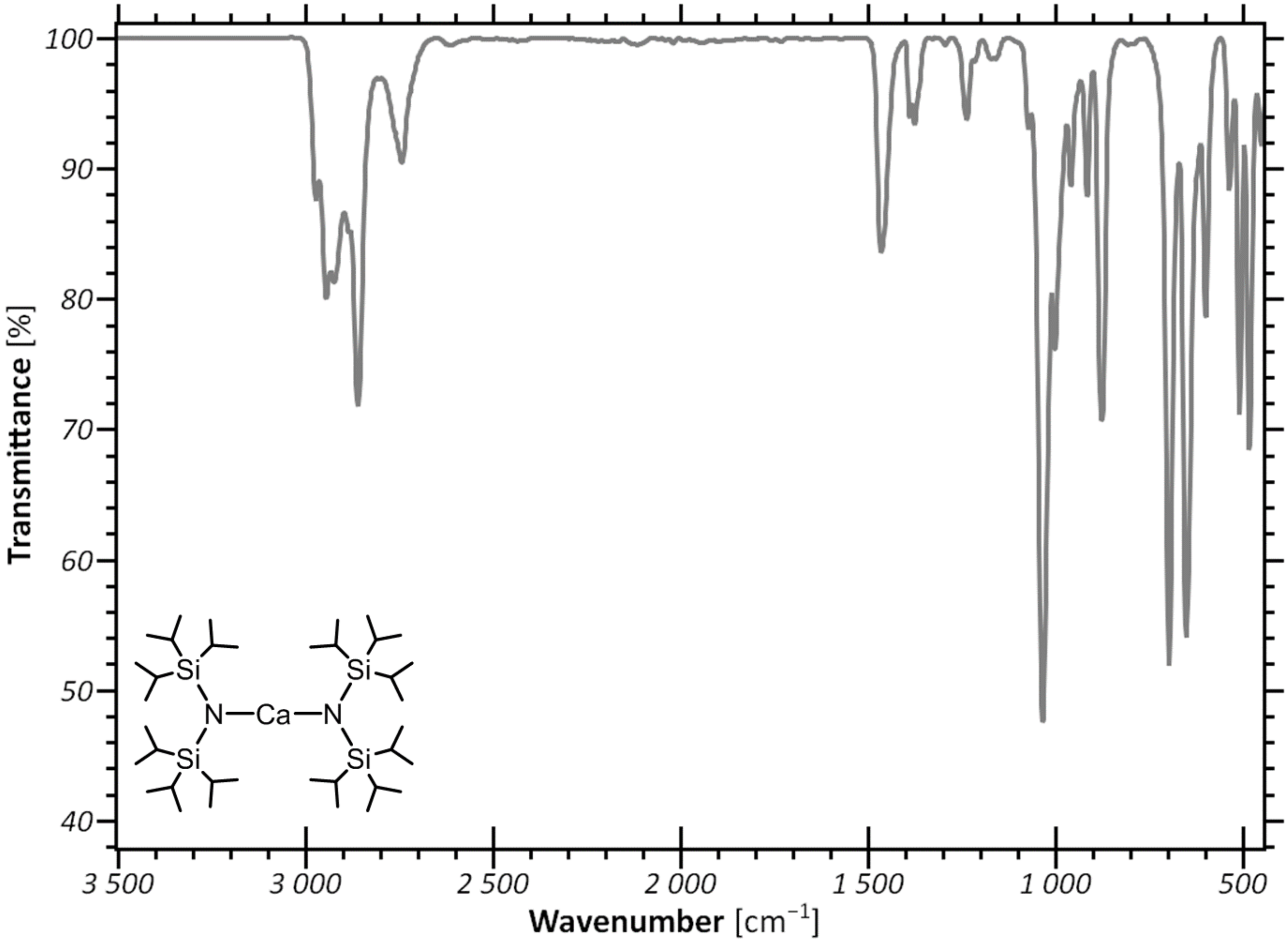

Figure S8. FT-IR ATR spectrum of $Ca[N(Si^{i}Pr_3)_2]_2$ **(1-Ca)**.

Synthesis of $Sr[N(Si^iPr_3)_2]_2$ (1-Sr)

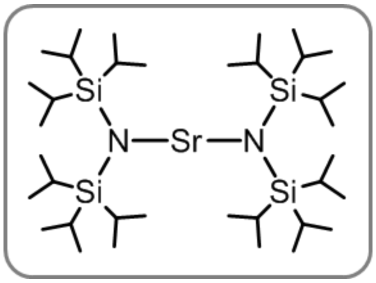

Benzene (30 mL) was added to a mixture of $[KN(Si^iPr_3)_2]_n$ (4.54 g, 12.34 mmol) and SrI_2 (2.14 g, 6.27 mmol) at room temperature. After stirring the resultant colorless suspension for two days at 70 °C, the solvent was removed under a dynamic vacuum. The crude off-white solid was then dried in high vacuum at 60 °C for 30 minutes, extracted with hexane (50 mL) and filtered. Evaporation of the solvent from the colorless hexane extract and drying under reduced pressure (60 °C, 30 minutes) resulted in a colorless powder, which was dissolved in boiling hexane (9 mL) and filtered. Slow cooling to 8 °C yielded large well-defined colorless block-like crystals of $Sr[N(Si^iPr_3)_2]_2$, which were separated from the supernatant by decantation, washed with cold pentane (–20 °C, 2 x 3 mL) and dried *in vacuo* at room temperature. A further crop of $Sr[N(Si^iPr_3)_2]_2$ (combined yield: 3.07 g, 4.12 mmol, 66%) was obtained after concentration of the mother liquor under a dynamic vacuum to approximately one third of its initial volume, gradual cooling to –20 °C overnight and isolation of the formed crystals.

^{1}H NMR (600 MHz, C_6D_6, 25 °C): δ_H = 0.89 (sept, ^3J(H,H) = 7.4 Hz, 12H, Si(C*H*Me$_2$)$_3$), 1.24 (d, ^3J(H,H) = 7.5 Hz, 72H, Si[CH(C*H*$_3$)$_2$]$_3$) ppm; **^{13}C{^{1}H} NMR** (151 MHz, C_6D_6, 25 °C): δ_C = 17.7 (s, Si(*C*HMe$_2$)$_3$), 20.1 (s, Si[CH(*C*H$_3$)$_2$]$_3$) ppm; **^{29}Si{^{1}H} NMR** (119 MHz, C_6D_6, 25 °C): δ_{Si} = –9.4 (s) ppm; **FT-IR** (ATR, pure): $\tilde{\upsilon}$ = 2940 (w), 2860 (m), 1457 (w), 1075 (m), 1053 (s), 1000 (w), 875 (m), 694 (s), 651 (s), 596 (w), 504 (m), 471 (m) cm^{-1}; **Elemental analysis:** calculated (%) for $C_{36}H_{84}N_2Si_4Sr$ (745.04 g mol^{-1}): C 58.04, H 11.36, N 3.76; found: C 57.67, H 11.48, N 3.70.

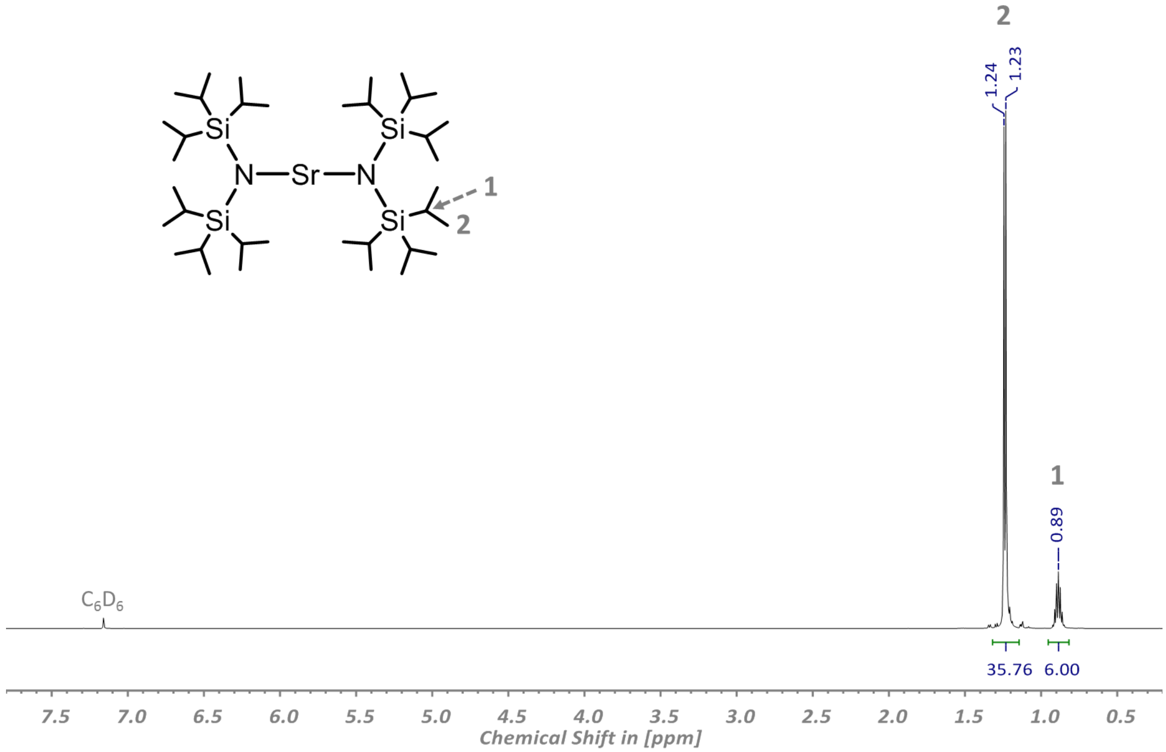

Figure S9. ^{1}H NMR spectrum (600 MHz, C_6D_6, 25 °C) of $Sr[N(Si^iPr_3)_2]_2$ **(1-Sr)**.

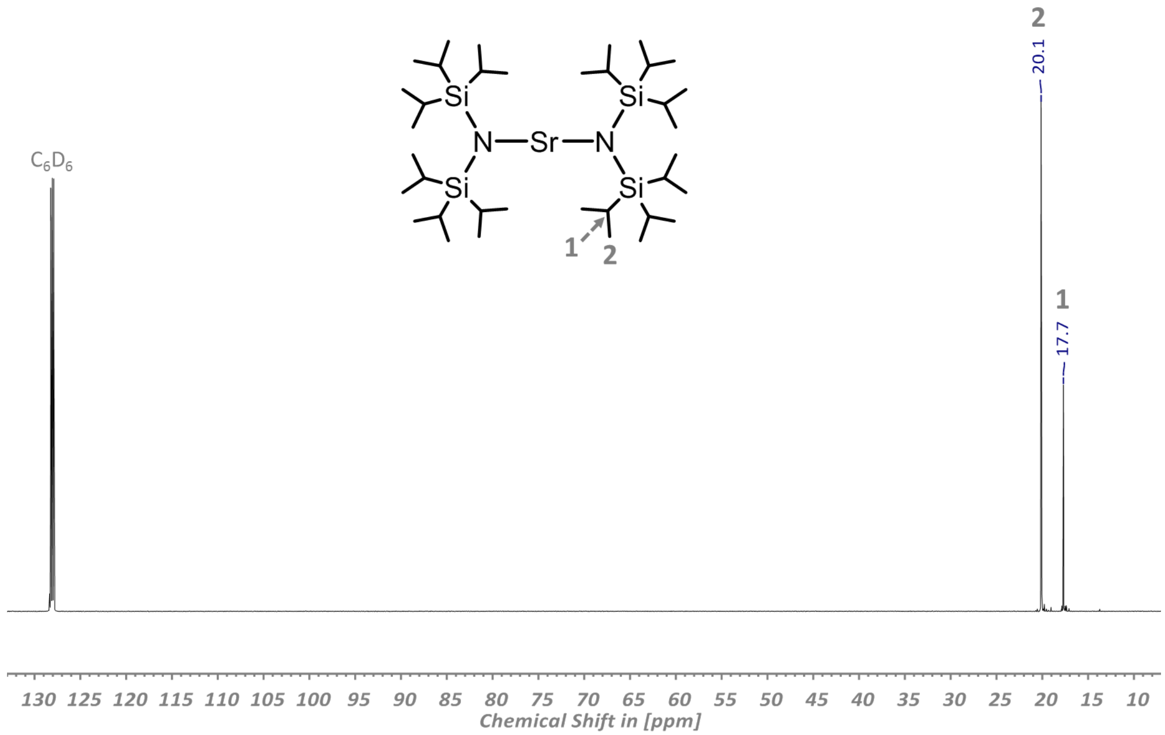

Figure S10. $^{13}C\{^{1}H\}$ NMR (151 MHz, C_6D_6, 25 °C) of $Sr[N(Si^iPr_3)_2]_2$ **(1-Sr)**.

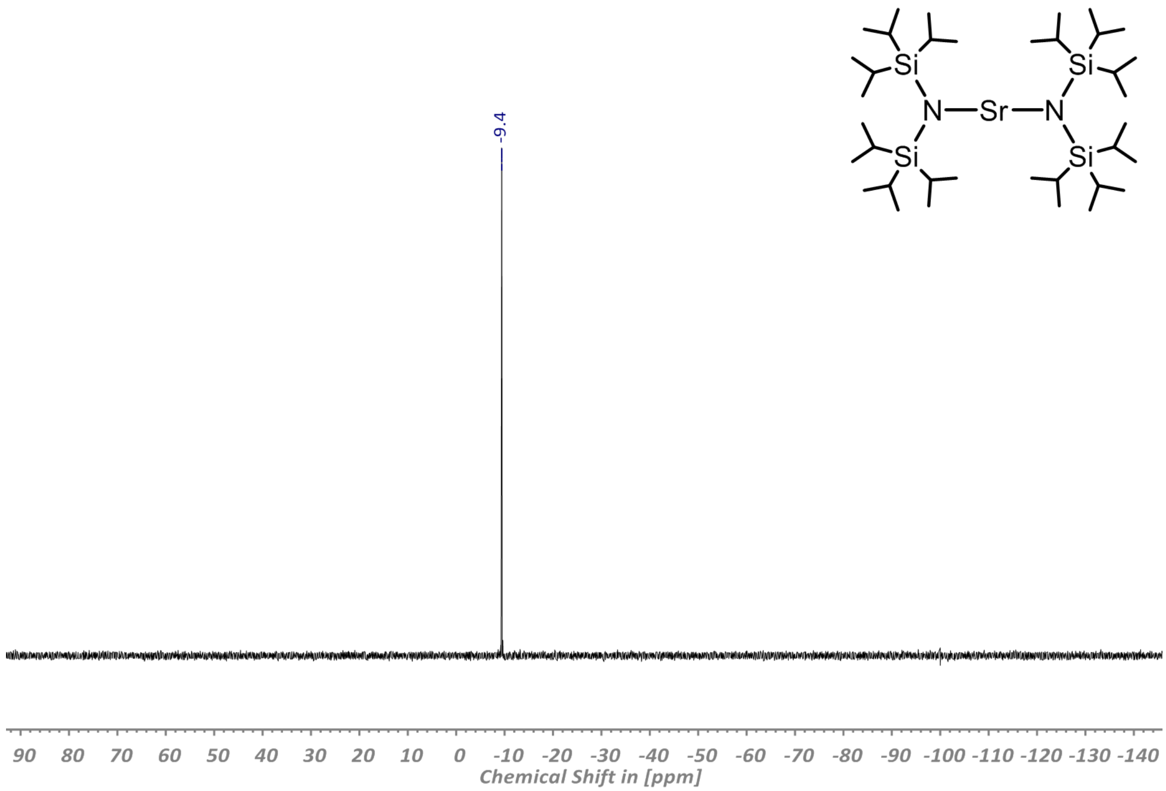

Figure S11. $^{29}Si\{^1H\}$ NMR spectrum (119 MHz, C_6D_6, 25 °C) of $Sr[N(Si^iPr_3)_2]_2$ **(1-Sr)**.

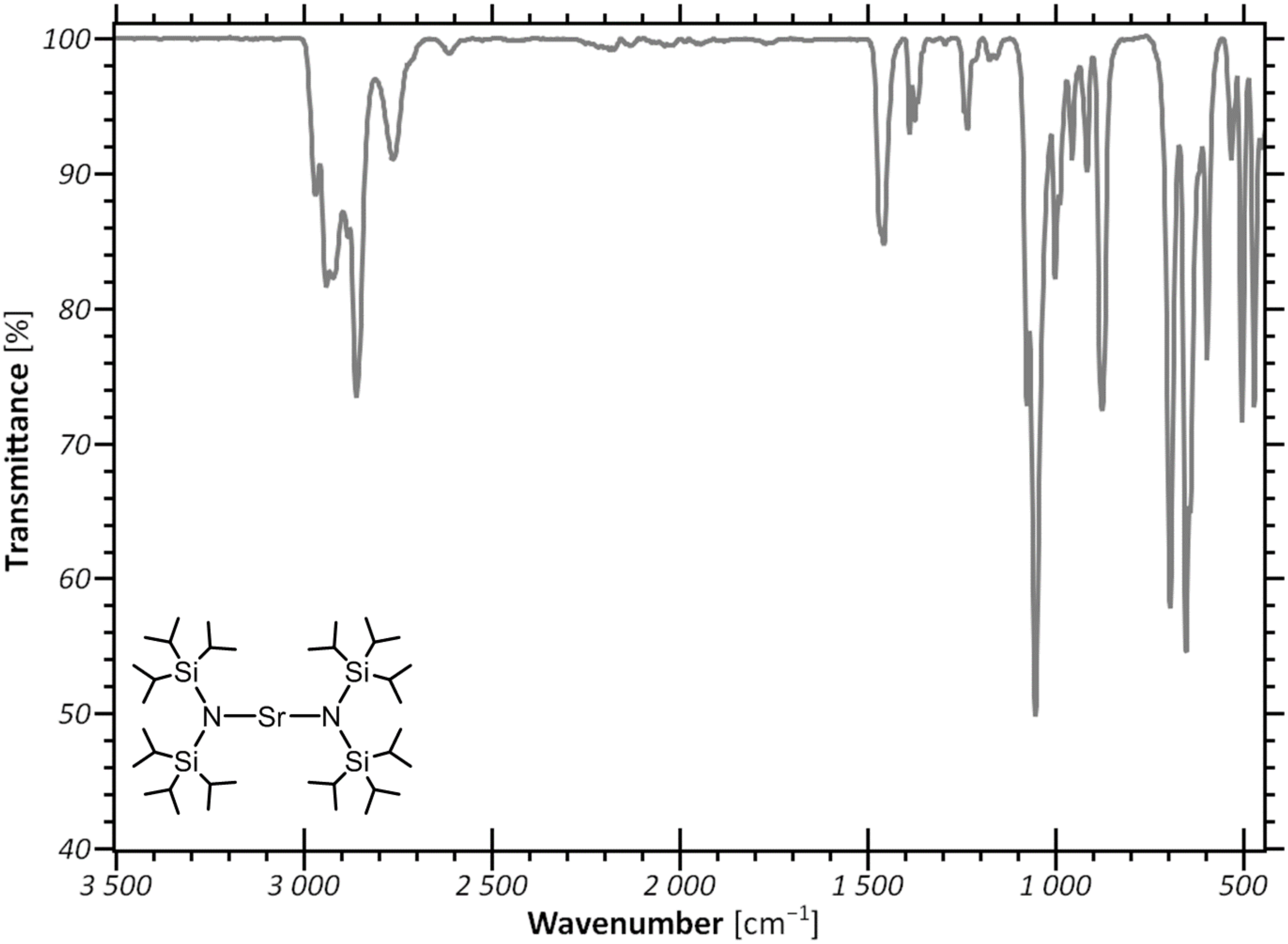

Figure S12. FT-IR ATR spectrum of Sr[N(Si^{i}Pr$_3$)$_2$]$_2$ **(1-Sr)**.

Synthesis of Ba[N(Si^{i}Pr$_3$)$_2$]$_2$ (1-Ba)

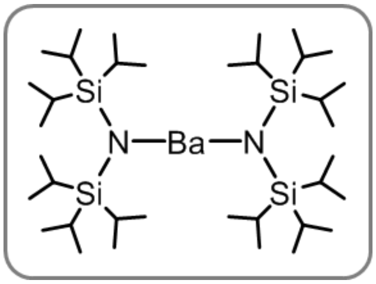

A colorless suspension of [KN(Si^{i}Pr$_3$)$_2$]$_n$ (6.61 g, 17.97 mmol) and BaI_2 (3.52 g, 9.00 mmol) in benzene (40 mL) was stirred at 70 °C over a period of four days, whereupon the solvent was removed under a dynamic vacuum and the solid residue dried *in vacuo* (60 °C, 30 minutes). The off-white solid was extracted with hexane (55 mL) once, filtered and the resultant colorless solution concentrated under reduced pressure. The residue, a colorless powder, was dried in high vacuum at 60 °C for 30 minutes, dissolved in a minimum volume of boiling hexane (15 mL), centrifuged and left to slowly cool to room temperature. A uniform batch of large, colorless block-like crystals of Ba[N(Si^{i}Pr$_3$)$_2$]$_2$ deposited overnight. These crystals were separated by decantation, washed with cold pentane (–20 °C, 2 x 3 mL) and dried in high vacuum at room temperature. The supernatant was collected and evaporated to dryness under a dynamic vacuum. A second crop of crystals was obtained in a similar fashion after dissolving the resultant colorless solid residue in a minimum amount of boiling hexane (4 mL) and gradual cooling to –20 °C. Ba[N(Si^{i}Pr$_3$)$_2$]$_2$ (combined yield: 4.82 g, 6.06 mmol) was isolated as colorless crystalline solid in an overall yield of 67%.

^{1}H NMR (600 MHz, C_6D_6, 25 °C): δ_H = 0.93 (sept, ^3J(H,H) = 7.4 Hz, 12H, Si(C*H*Me$_2$)$_3$), 1.23 (d, ^3J(H,H) = 7.5 Hz, 72H, Si[CH(C*H*$_3$)$_2$]$_3$) ppm; **^{13}C{^{1}H} NMR** (151 MHz, C_6D_6, 25 °C): δ_C = 17.2 (s, Si(*C*HMe$_2$)$_3$), 20.2 (s, Si[CH(*C*H$_3$)$_2$]$_3$) ppm; **^{29}Si{^{1}H} NMR** (119 MHz, C_6D_6, 25 °C): δ_{Si} = –11.1 (s) ppm; **^{1}H NMR** (600 MHz, [*d*$_8$]toluene, 25 °C): δ_H = 0.89 (sept, ^3J(H,H) = 7.4 Hz, 12H, Si(C*H*Me$_2$)$_3$), 1.20 (d, ^3J(H,H) = 7.5 Hz, 72H, Si[CH(C*H*$_3$)$_2$]$_3$) ppm; **^{13}C{^{1}H} NMR** (151 MHz, [*d*$_8$]toluene, 25 °C): δ_C = 17.2 (s, Si(*C*HMe$_2$)$_3$), 20.2 (s, Si[CH(*C*H$_3$)$_2$]$_3$) ppm; **^{29}Si{^{1}H} NMR** (119 MHz, [*d*$_8$]toluene, 25 °C): δ_{Si} = –11.1 (s) ppm; **FT-IR** (ATR, pure): $\tilde{\upsilon}$ = 2962 (w), 2938 (w), 2862 (m), 2799 (w), 1461 (m), 1089 (s), 1065 (s), 994 (m), 981 (w), 877 (s), 698 (s), 659 (s), 612 (s), 539 (w), 504 (s), 469 (w) cm^{-1}; **Elemental analysis:** calculated (%) for $C_{36}H_{84}BaN_2Si_4$ (794.75 g mol^{-1}): C 54.41, H 10.65, N 3.52; found: C 54.24, H 10.75, N 3.57.

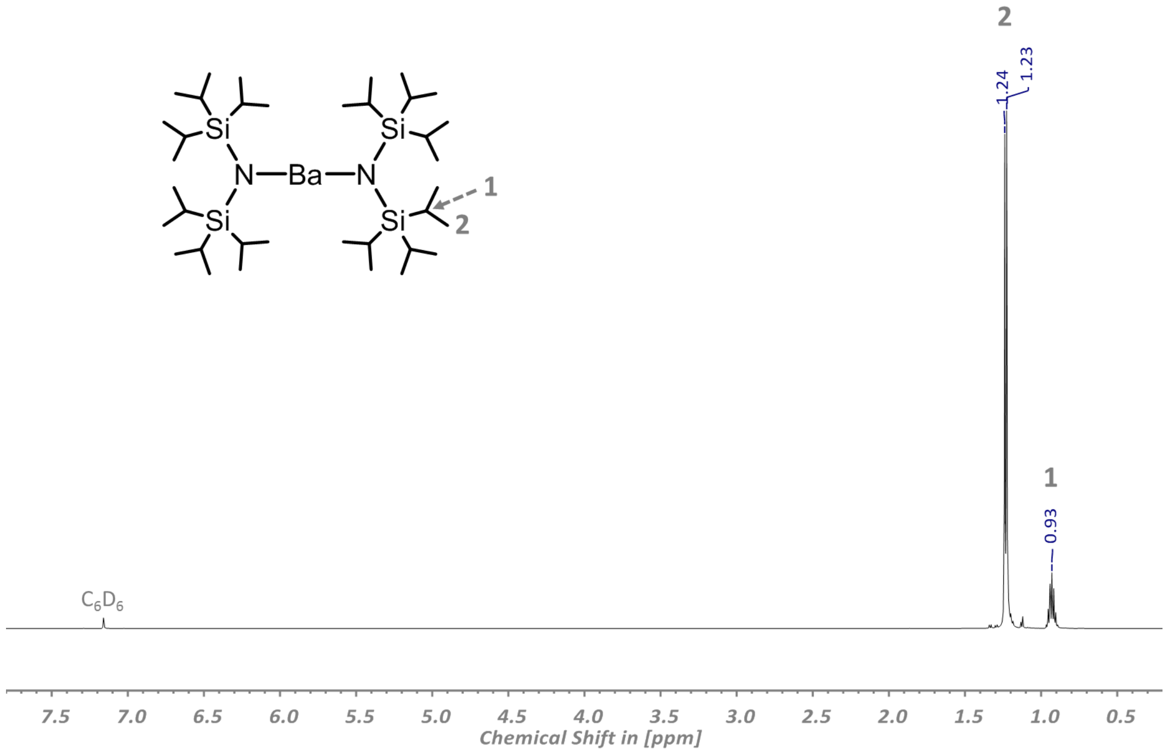

Figure S13. ^{1}H NMR spectrum (600 MHz, C_6D_6, 25 °C) of Ba[N(Si^{i}Pr$_3$)$_2$]$_2$ **(1-Ba)**.

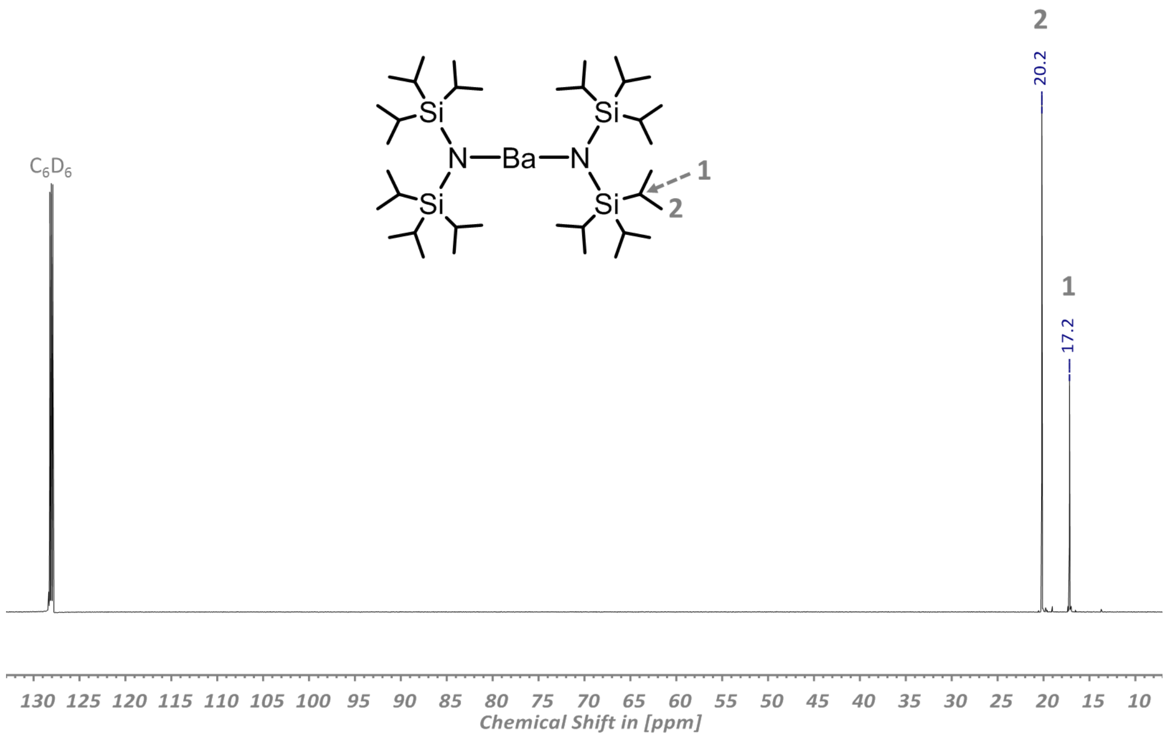

Figure S14. ^{13}C{^{1}H} NMR (151 MHz, C_6D_6, 25 °C) of Ba[N(Si^{i}Pr$_3$)$_2$]$_2$ **(1-Ba)**.

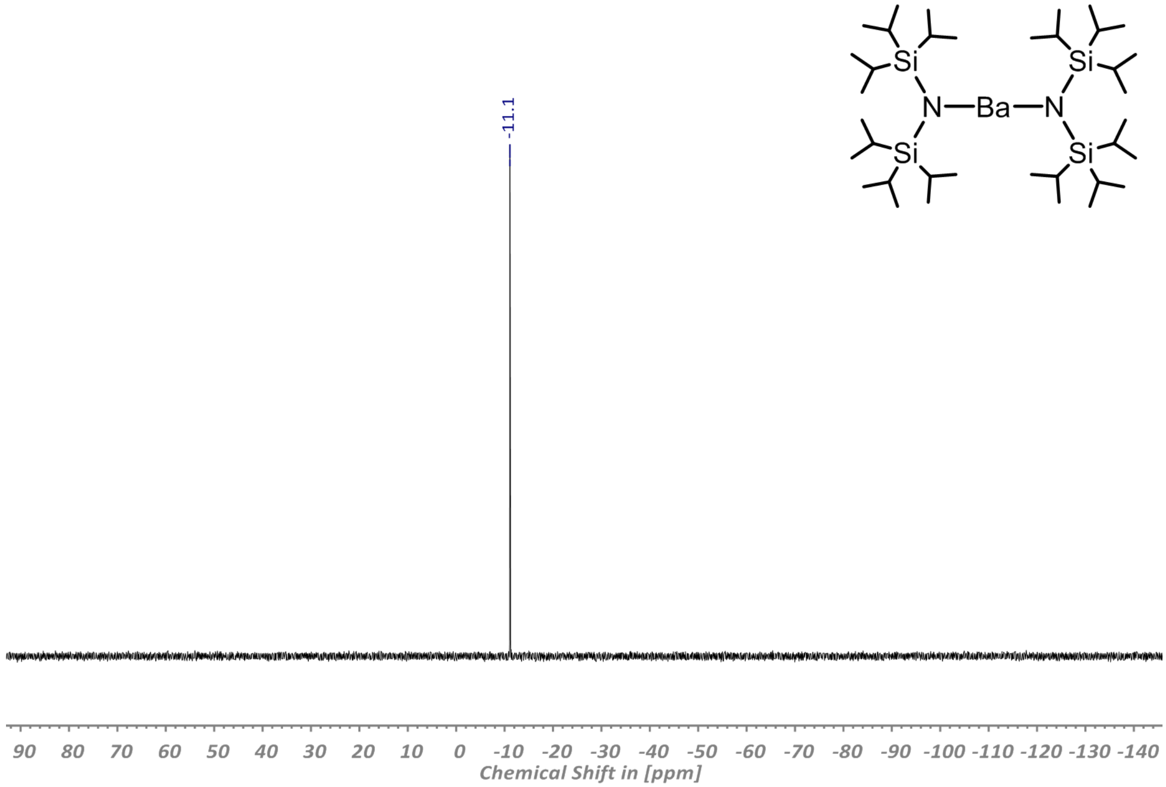

Figure S15. $^{29}Si\{^{1}H\}$ NMR spectrum (119 MHz, C_6D_6, 25 °C) of $Ba[N(Si^iPr_3)_2]_2$ **(1-Ba)**.

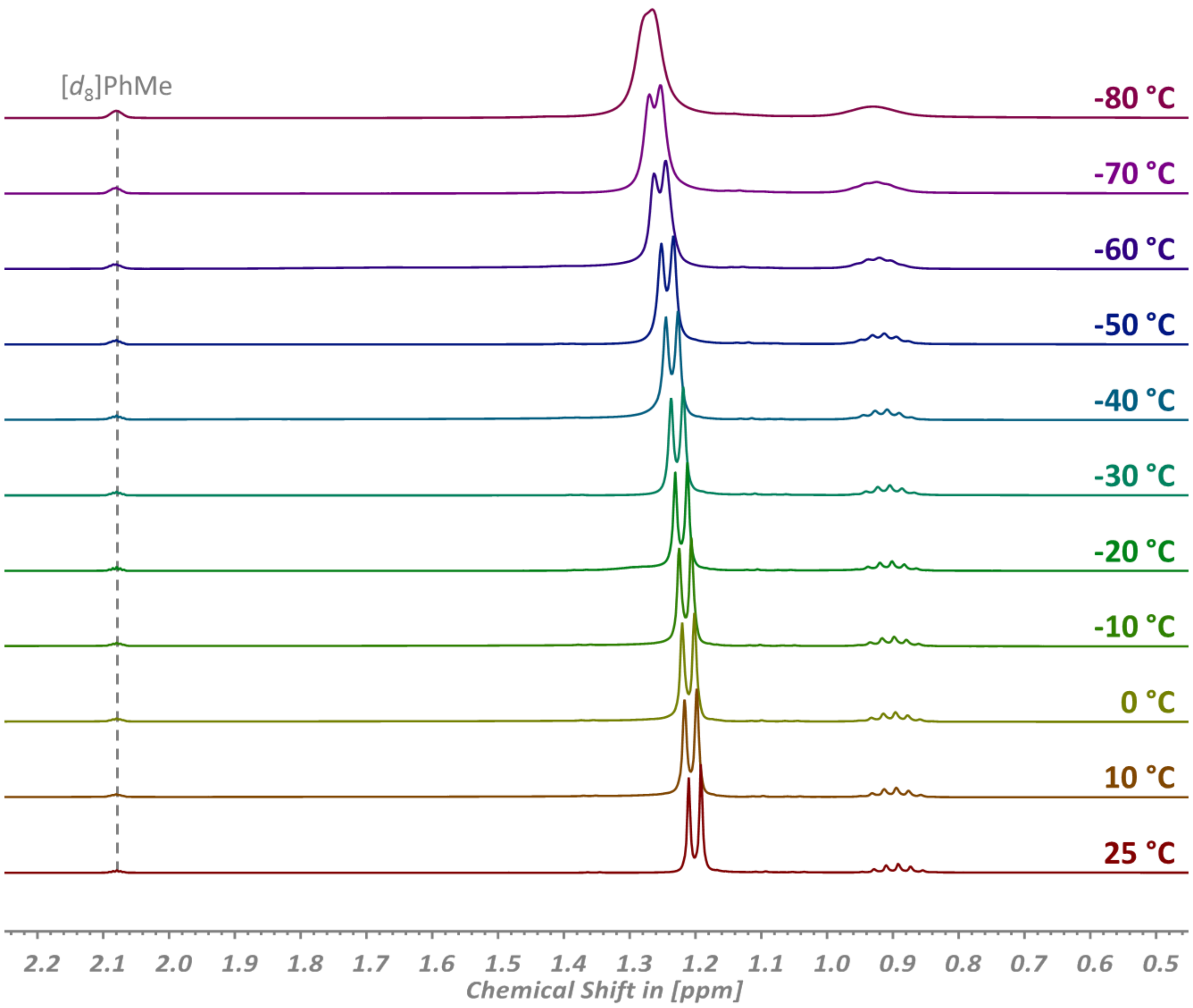

Figure S16. Temperature depended ^{1}H NMR spectra (400 MHz, [d_8]toluene) of $Ba[N(Si^iPr_3)_2]_2$ **(1-Ba)** over the temperature range from 25 °C to -80 °C.

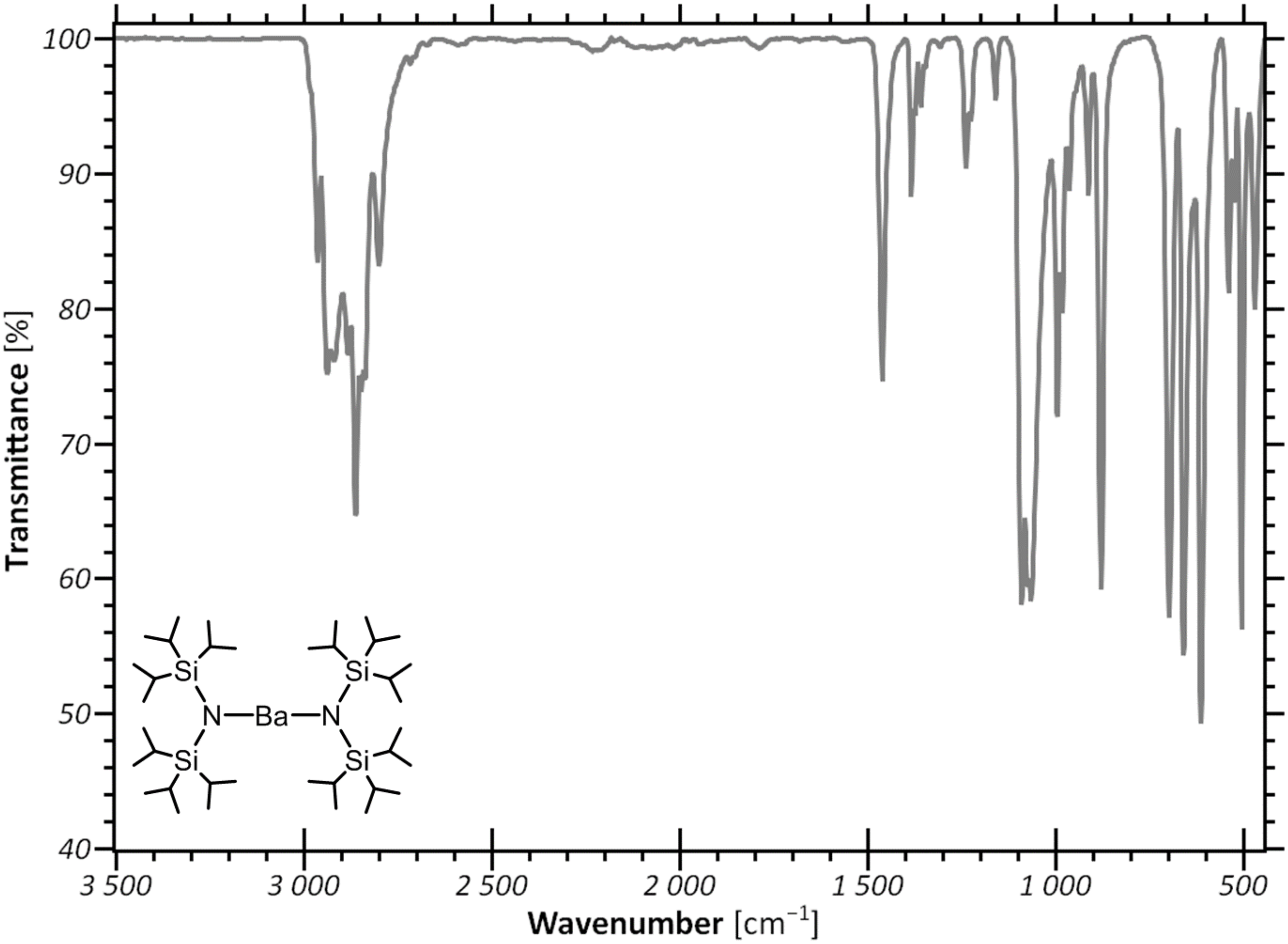

Figure S17. FT-IR ATR spectrum of Ba[N(Si^{i}Pr$_3$)$_2$]$_2$ **(1-Ba)**.

Synthesis of $\{Ba[N(Si^iPr_3)_2]_2(3,4\text{-dihydro-}2H\text{-pyran})\}$

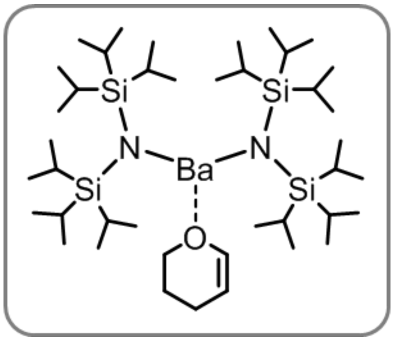

$Ba[N(Si^iPr_3)_2]_2$ (53 mg, 0.07 mmol) was dissolved in 3,4-dihydro-2*H*-pyran (300 µL, 277 mg, 3.29 mmol) and the resulting bright-yellow solution transferred into a J. Young NMR tube. The reaction mixture was then heated at 60 °C in a heating block for two hours. The pale orange solution was left to cool to room temperature and filtered. Storage at –20 °C for three days repeatedly resulted in the separation of $\{Ba[N(Si^iPr_3)_2]_2(3,4\text{-dihydro-}2H\text{-pyran})\}$ as colorless block-like crystals suitable for X-ray diffraction.

Note: No further spectroscopic and analytical characterization was possible due to the hemilability of the bound 3,4-dihydro-2*H*-pyran. In solution most likely an equilibrium exists between free $BaN[Si^iPr_3)_2]_2$ and the pyran adduct. In the following scheme, VT-NMR spectra of an equimolar mixture of 3,4-dihydro-2*H*-pyran and $BaN[Si^iPr_3)_2]_2$ in [d_8]toluene is depicted.

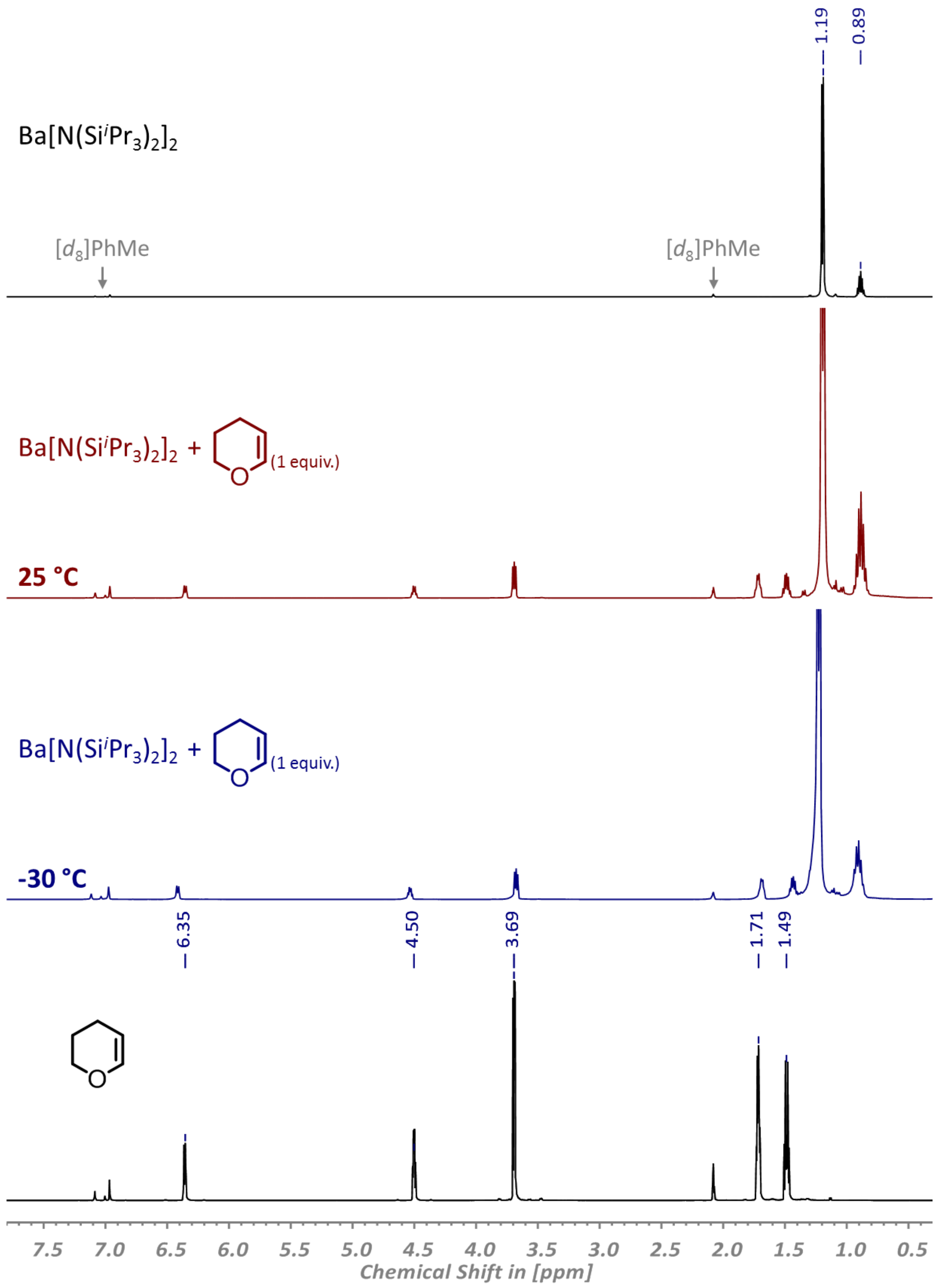

Figure S18. Temperature dependent ^{1}H NMR spectra (400 MHz, [d_8]toluene) of a 1/1 mixture of Ba[N(Si^{i}Pr$_3$)$_2$]$_2$ and 3,4-dihydro-2*H*-pyran at 25 °C (*red*) and –30 °C (*blue*). For reference, the respective ^{1}H NMR spectra of Ba[N(Si^{i}Pr$_3$)$_2$]$_2$ (*top*) and 3,4-dihydro-2*H*-pyran (*bottom*) in [d_8]toluene at 25 °C are shown.

Synthesis of [KN(DIPP)(Si^{i}Pr$_3$)]$_n$ (1-K)

This compound was prepared according to a modified literature procedure.[S9]

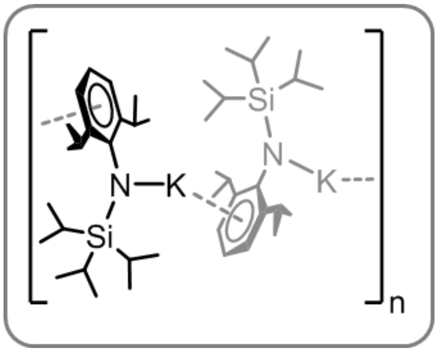

Toluene (37 mL) was added to a mixture of HN(DIPP)(Si^{i}Pr$_3$) (2.16 g, 6.47 mmol) and potassium hydride (320 mg, 7.98 mmol) at room temperature. The resulting stirred greyish suspension was heated to reflux for 18 hours, after which it was allowed to cool to room temperature. The solvent was removed from the ochre suspension under reduced pressure to give a crude pale yellow solid, which was treated with diethyl ether (50 mL) once. After filtration through a filter cannula and concentration of the yellow filtrate under a dynamic vacuum, a crude beige-colored solid was obtained, which was dried *in vacuo* at 60 °C for 30 minutes. Washing with hexane (4 x 20 mL) and subsequent drying in vacuum (60 °C, 1 hour) gave [KN(DIPP)(Si^{i}Pr$_3$)]$_n$ (1.86 g, 5.00 mmol, 77%) as an off-white powder, which was used without further purification. Single needle-like crystals of [KN(DIPP)(Si^{i}Pr$_3$)]$_n$ suitable for X-ray diffraction were obtained by slowly cooling a warm saturated hexane / toluene solution (70 °C, 2:1) to room temperature overnight.

^{1}H NMR (600 MHz, [d_8]THF, 25 °C): δ_H = 0.99 – 1.09 (m, 33H, Si(C*H*Me$_2$)$_3$ and Si[CH(CH_3)$_2$]$_3$ and CH(CH_3)$_2$), 4.02 (d, ^3J(H,H) = 7.0 Hz, 2H, C*H*Me$_2$), 6.07 (t, ^3J(H,H) = 7.3 Hz, 1H, *para*-CH_{arom}), 6.68 (d, ^3J(H,H) = 7.3 Hz, 2H, *meta*-CH_{arom}) ppm; **^{13}C{^{1}H} NMR** (151 MHz, [d_8]THF, 25 °C): δ_C = 17.2 (s, Si(*C*HMe$_2$)$_3$), 20.6 (s, Si[CH(CH$_3$)$_2$]$_3$), 24.9 (s, CH(CH$_3$)$_2$), 26.5 (*C*HMe$_2$), 109.7 (s, *para*-C_{arom}), 122.7 (s, *meta*-C_{arom}), 140.2 (s, *ortho*-C_{arom}), 157.1 (s, *ipso*-C_{arom}) ppm; **^{29}Si{^{1}H} NMR** (119 MHz, [d_8]THF, 25 °C): δ_{Si} = –28.3 (s) ppm; **FT-IR** (ATR, pure): $\tilde{\upsilon}$ = 2954 (w), 2922 (w), 2854 (w), 1418 (m), 1369 (w), 1285 (w), 963 (w), 877 (w), 759 (m), 745 (w), 645 (m), 626 (m) cm^{-1}; **Elemental analysis:** calculated (%) for $C_{21}H_{38}KNSi$ (371.73 g mol^{-1}): C 67.85, H 10.30, N 3.77; found: C 68.13, H 10.18, N 3.81.

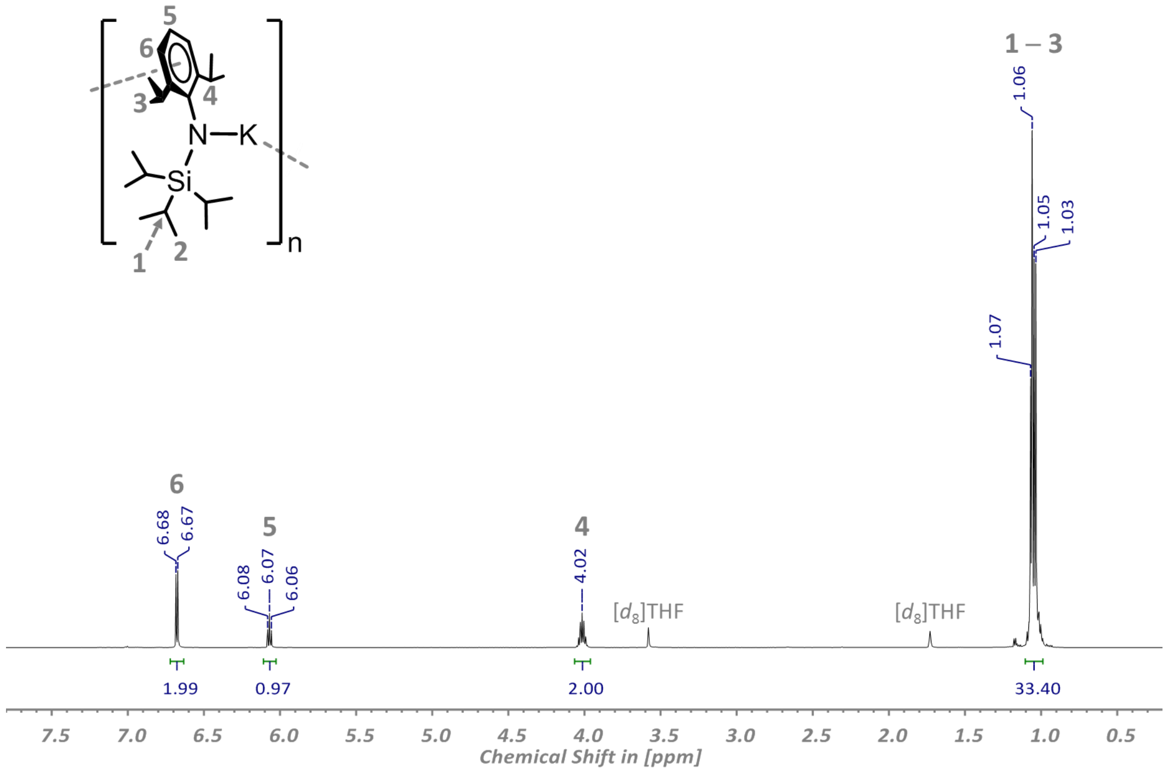

Figure S19. ^{1}H NMR spectrum (600 MHz, [d_8]THF, 25 °C) of [KN(DIPP)(Si^{i}Pr$_3$)]$_n$ **(2-K)**.

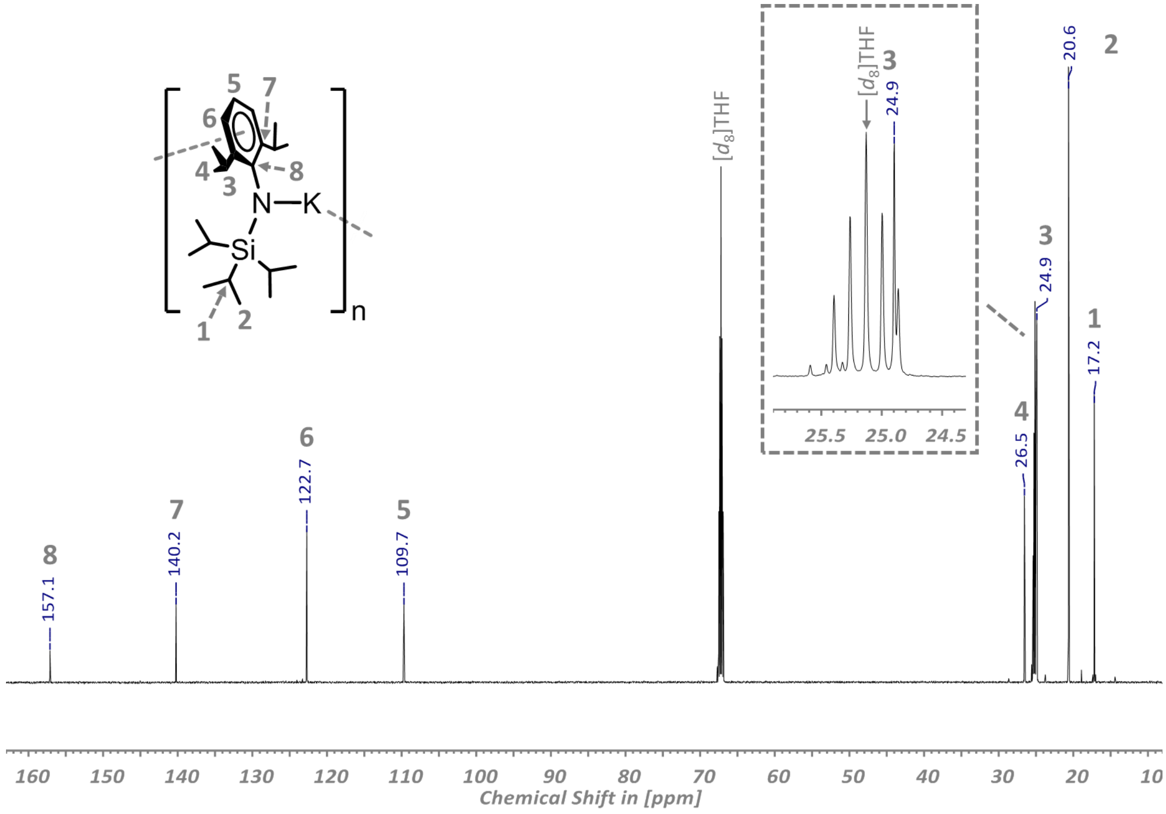

Figure S20. ^{13}C{^{1}H} NMR (151 MHz, [d_8]THF, 25 °C) of [KN(DIPP)(Si^{i}Pr$_3$)]$_n$ **(2-K)**.

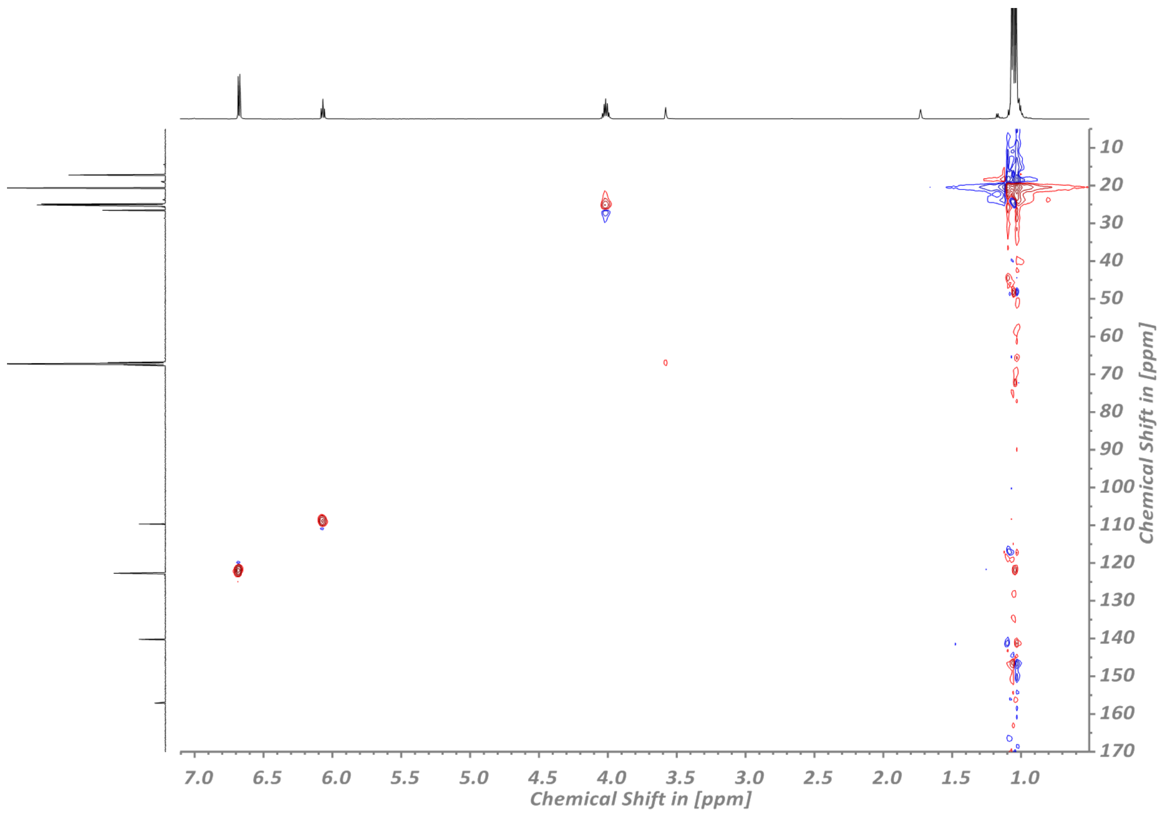

Figure S21. ^{1}H, ^{13}C HSQC NMR spectrum (600 MHz / 151 MHz, [d_8]THF, 25 °C) of [KN(DIPP)(Si^{i}Pr$_3$)]$_n$.

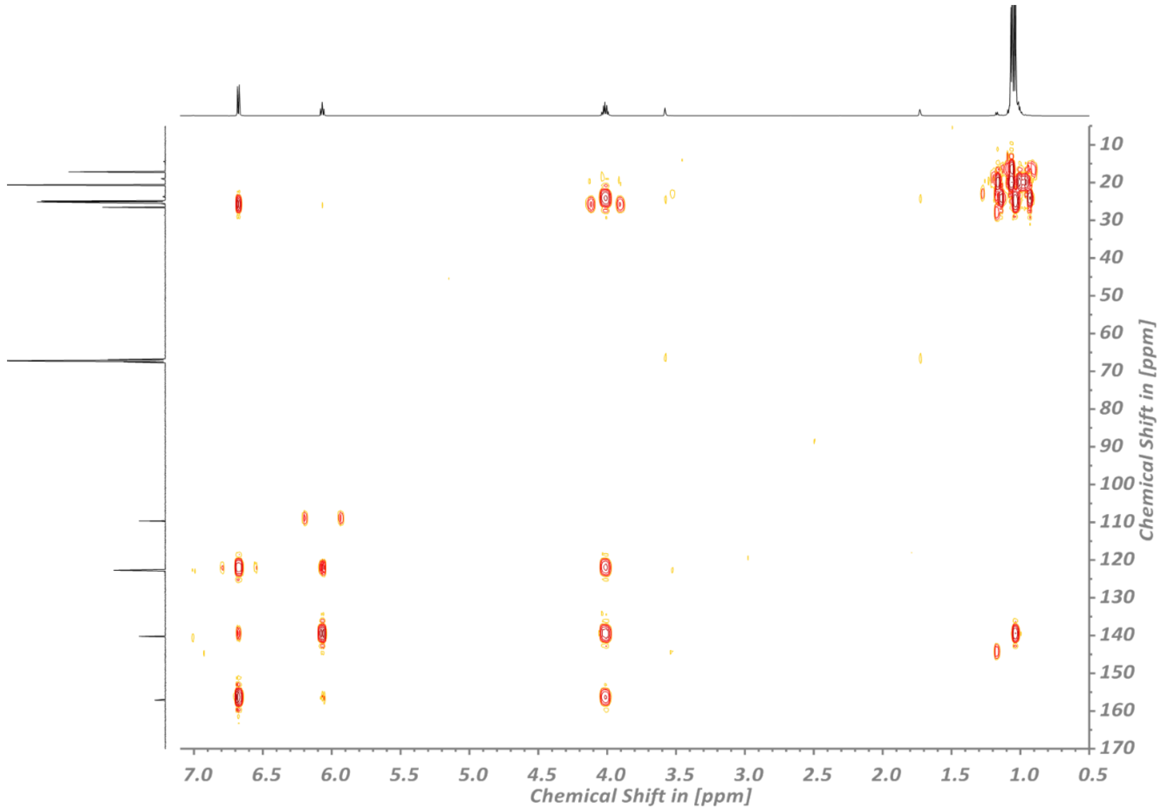

Figure S22. ^{1}H, ^{13}C HMBC NMR spectrum (600 MHz / 151 MHz, [d_8]THF, 25 °C) of [KN(DIPP)(Si^{i}Pr$_3$)]$_n$.

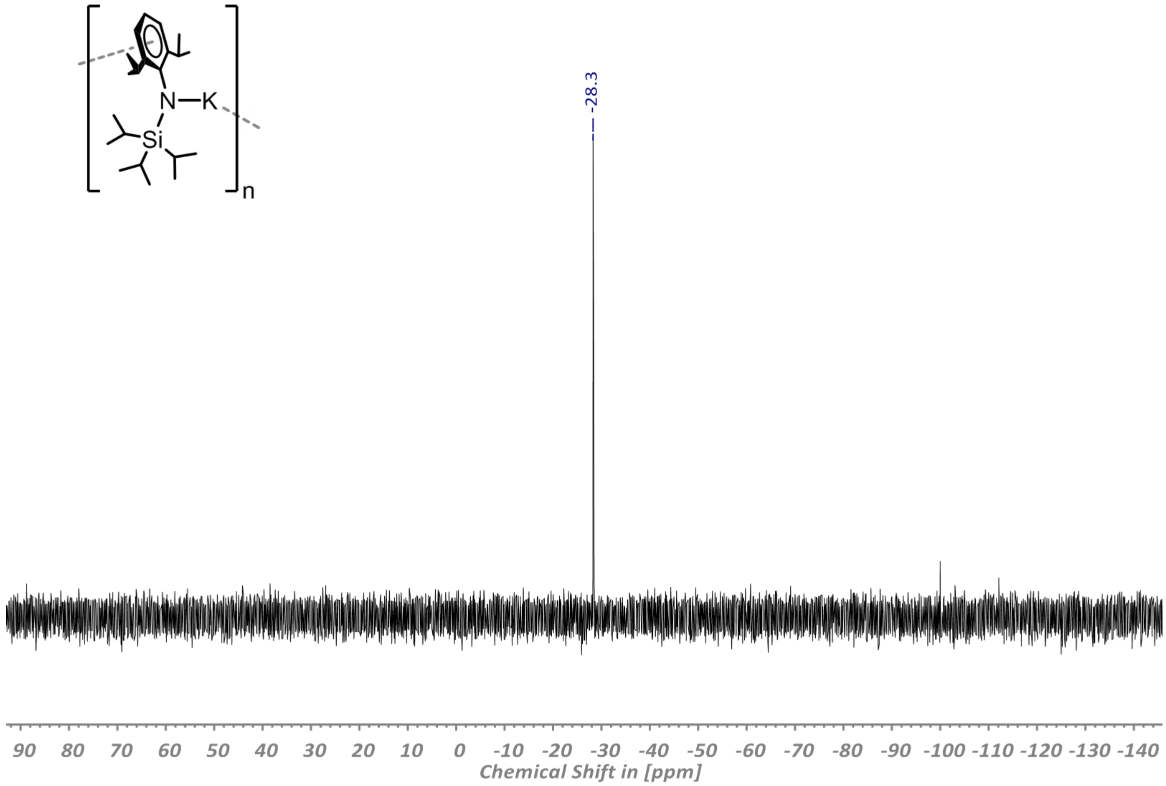

Figure S23. $^{29}Si\{^{1}H\}$ NMR spectrum (119 MHz, [d_8]THF, 25 °C) of [KN(DIPP)(Si^{i}Pr$_3$)]$_n$ **(2-K)**.

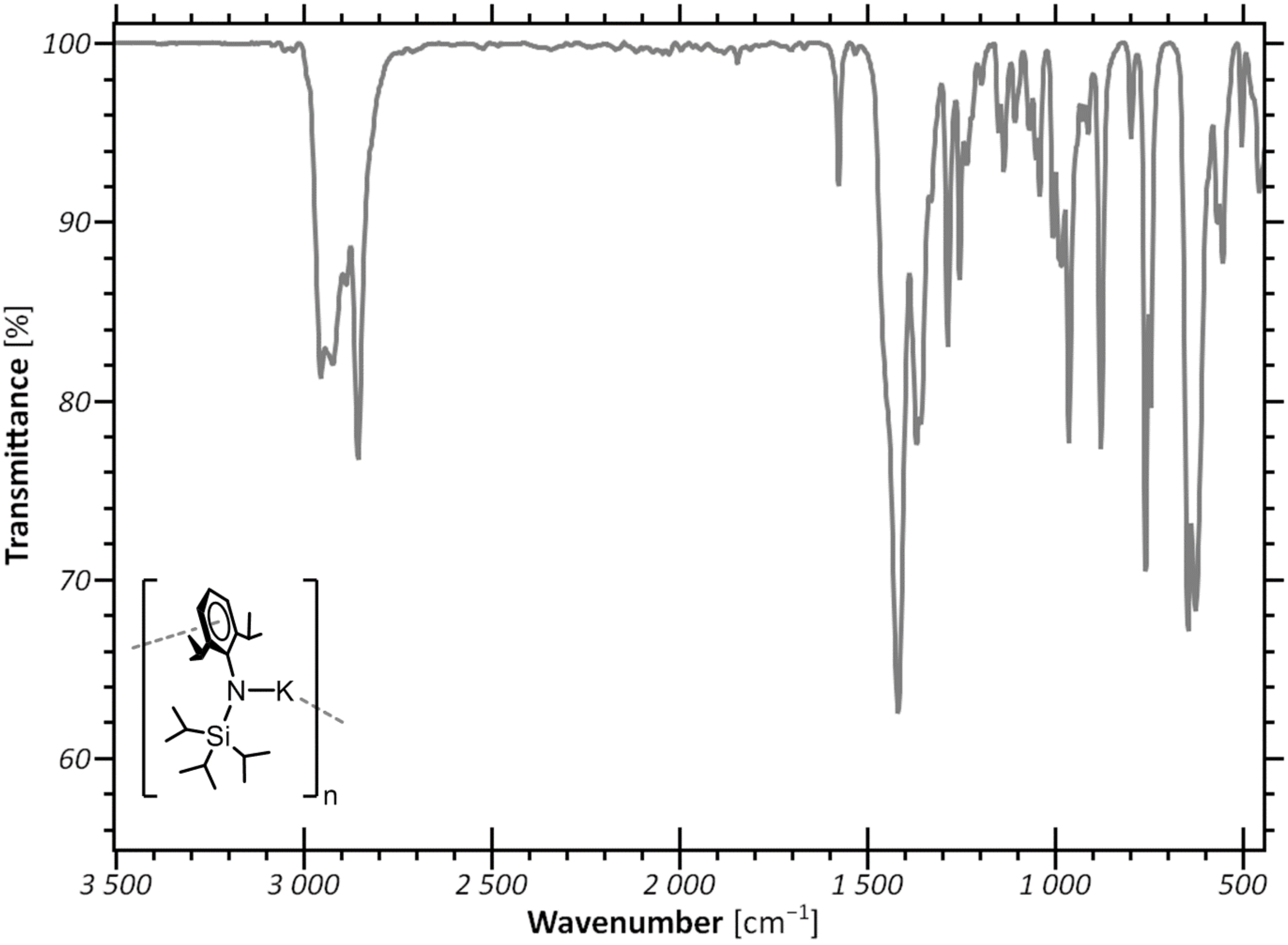

Figure S24. FT-IR ATR spectrum of [KN(DIPP)(Si^{i}Pr$_3$)]$_n$ **(2-K)**.

Synthesis of Mg[N(DIPP)(Si^{i}Pr$_3$)]$_2$ (2-Mg)

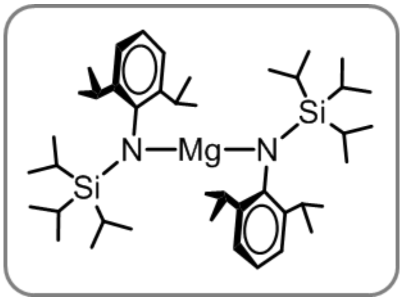

This complex was prepared by two different methods:

Method A: [KN(DIPP)(Si^{i}Pr$_3$)]$_n$ (1.04 g, 2.80 mmol) and MgI_2 (407 mg, 1.46 mmol) were combined in a dried Schlenk tube and benzene (10 mL) was added at room temperature. The resulting mixture was stirred for four days at 70 °C. Subsequent evaporation of the solvent from the ochre suspension gave a solid residue, which was extracted with hexane (10 mL). The hexane extract was filtered and the clear pale yellow solution concentrated *in vacuo*. The resultant beige-colored solid was then dried in high vacuum. Large, uniform colorless block-like crystals of Mg[N(DIPP)(Si^{i}Pr$_3$)]$_2$ (399 mg, 0.58 mmol, 40%) were obtained after cooling a saturated solution in hexane (3.5 mL) to –20 °C overnight. These extremely air- and moisture-sensitive crystals were isolated after decanting the supernatant and removing residual volatile compounds briefly under reduced pressure.

Method B: To a solution of HN(DIPP)(Si^{i}Pr$_3$) (889 mg, 2.66 mmol) in benzene (3.5 mL) was added di-*n*-butylmagnesium (1 M in heptane, 1.35 mL, 1.35 mmol) at room temperature and the resulting solution was transferred into an oven-dried microwave vial. The vial was sealed with a septum and the stirred reaction mixture heated at 160 °C (5 bar) in a laboratory microwave reactor for three hours. All volatiles were then removed under a dynamic vacuum. The beige-colored solid residue was dissolved in hexane (4 mL), filtered and the resultant brown solution kept at –20 °C overnight. Mg[N(DIPP)(Si^{i}Pr$_3$)$_2$ (374 mg, 0.54 mmol, 40%) crystallized in the form of large well-defined colorless blocks, which were separated from the mother liquor, briefly dried *in vacuo* at room temperature and collected.

^{1}H NMR (600 MHz, C_6D_6, 25 °C): δ_H = 0.80 (d, ^3J(H,H) = 6.9 Hz, 12H, CH(C*H*$_3$)$_2$), 1.10 – 1.19 (m, 42H, Si(C*H*Me$_2$)$_3$ and Si[CH(C*H*$_3$)$_2$]$_3$), 1.26 (d, ^3J(H,H) = 6.9 Hz, 12H, CH(C*H*$_3$)$_2$), 3.80 (sept, ^3J(H,H) = 6.8 Hz, 4H, C*H*Me$_2$), 6.92 – 6.98 (m, 6H, C*H*$_{arom}$) ppm; **^{13}C{^{1}H} NMR** (151 MHz, C_6D_6, 24 °C): δ_C = 15.9 (s, Si(*C*HMe$_2$)$_3$), 19.8 (s, Si[CH(*C*H$_3$)$_2$]$_3$), 24.3 (s, CH(*C*H$_3$)$_2$), 25.8 (s, CH(*C*H$_3$)$_2$), 27.2 (*C*HMe$_2$), 122.1 (s, *para*-*C*$_{arom}$), 124.1 (s, *meta*-*C*$_{arom}$), 144.7 (s, *ortho*-*C*$_{arom}$), 147.3 (s, *ipso*-*C*$_{arom}$) ppm; **^{29}Si{^{1}H} NMR** (119 MHz, C_6D_6, 22 °C): δ_{Si} = –3.6 (s) ppm; **FT-IR** (ATR, pure): $\tilde{\nu}$ = 2950 (w), 2864 (w), 1424 (w), 1236 (w), 1183 (w), 910 (m), 875 (w), 828 (m), 785 (m), 651 (m) cm^{-1}; **Elemental analysis:** calculated (%) for $C_{42}H_{76}MgN_2Si_2$ (689.56 g mol^{-1}): C 73.16, H 11.11, N 4.06; found: C 73.30, H 11.13, N 4.03.

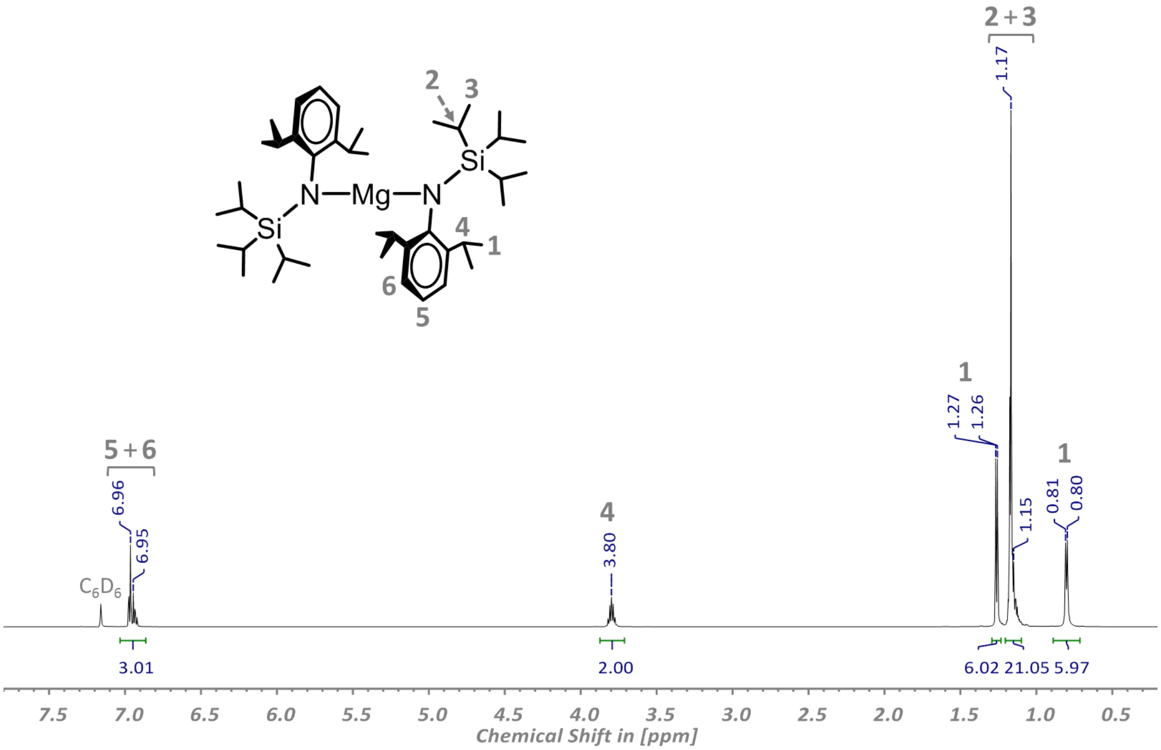

Figure S25. ^{1}H NMR spectrum (600 MHz, C_6D_6, 25 °C) of Mg[N(DIPP)(Si^{i}Pr$_3$)]$_2$ **(2-Mg)**.

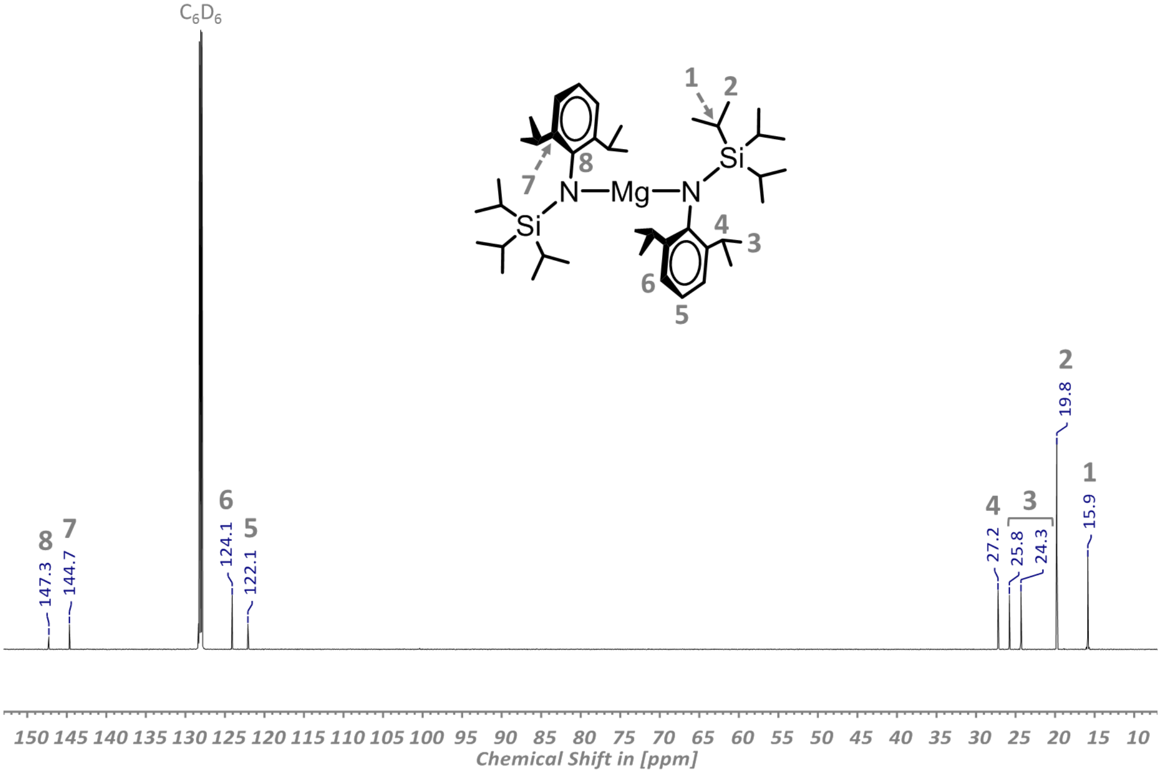

Figure S26. $^{13}C\{^{1}H\}$ NMR (151 MHz, C_6D_6, 24 °C) of Mg[N(DIPP)(Si^{i}Pr$_3$)]$_2$ **(2-Mg)**.

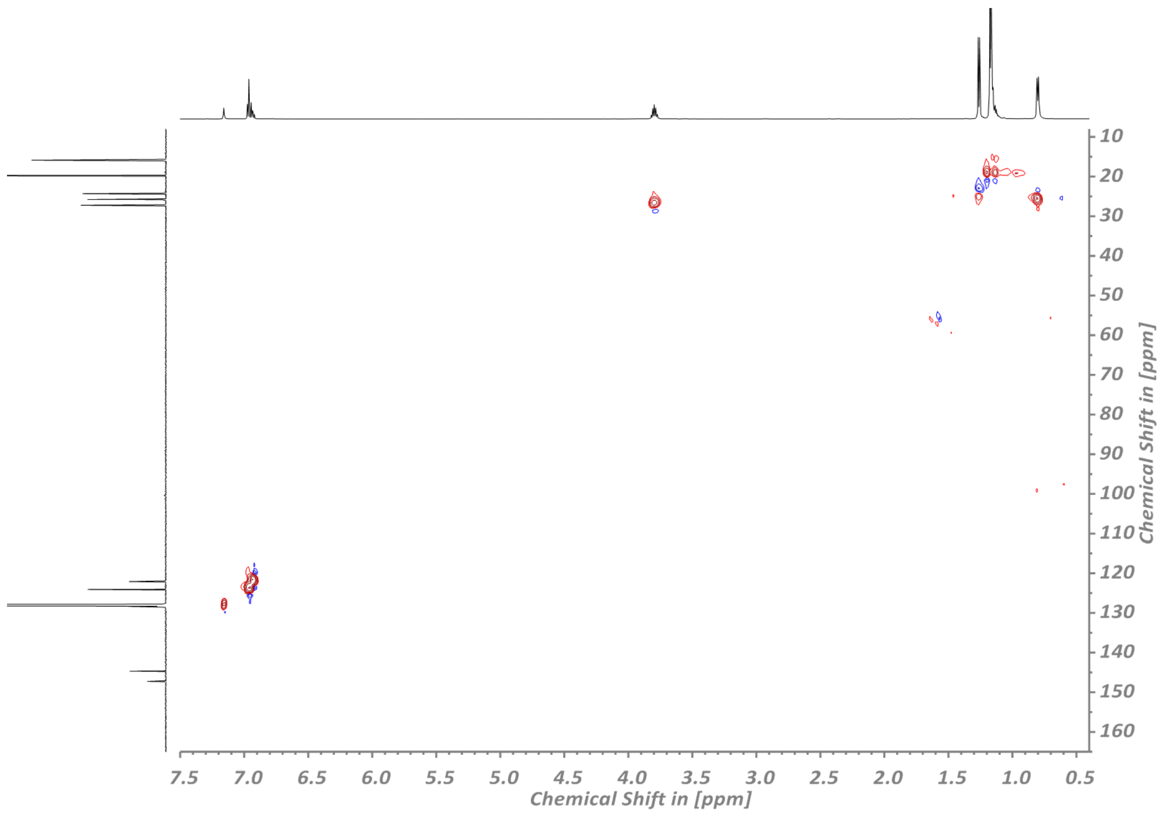

Figure S27. ^{1}H, ^{13}C HSQC NMR spectrum (600 MHz / 151 MHz, C_6D_6, 22 °C) of Mg[N(DIPP)(Si^{i}Pr$_3$)]$_2$.

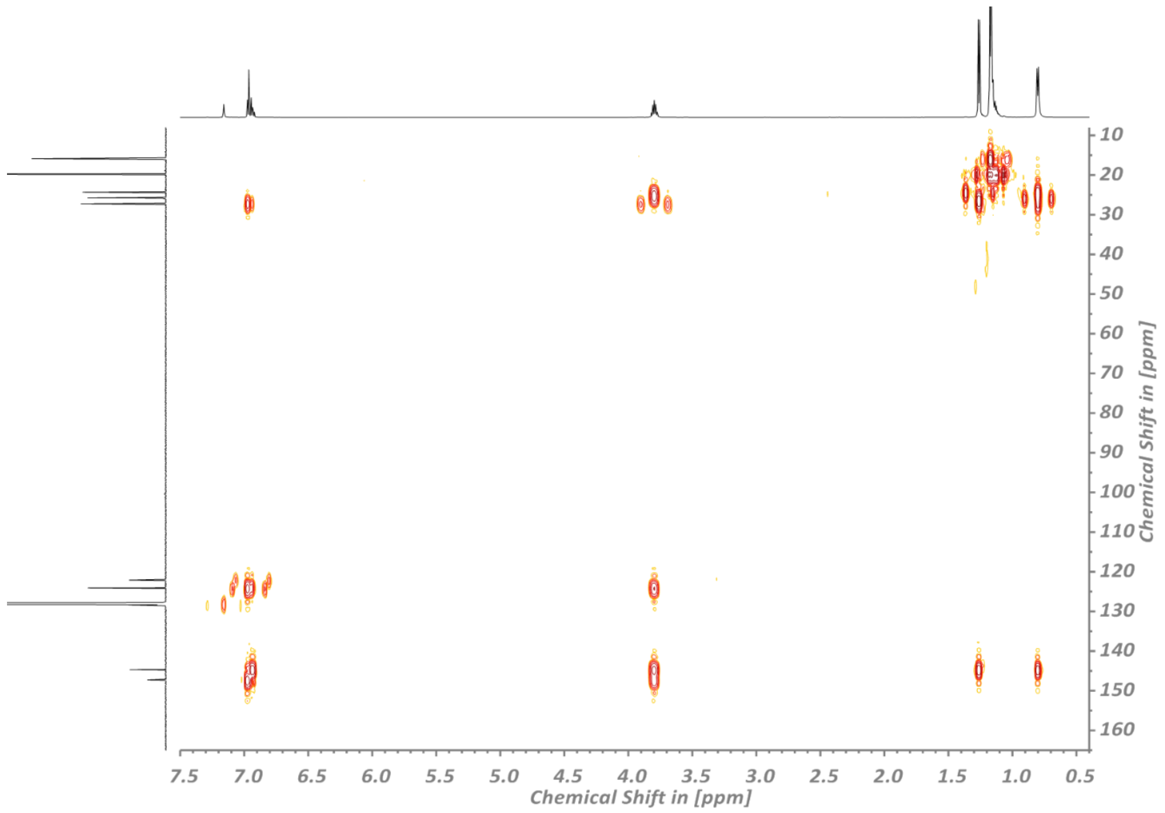

Figure S28. ^{1}H, ^{13}C HMBC NMR spectrum (600 MHz / 151 MHz, C_6D_6, 23 °C) of Mg[N(DIPP)(Si^{i}Pr$_3$)]$_2$.

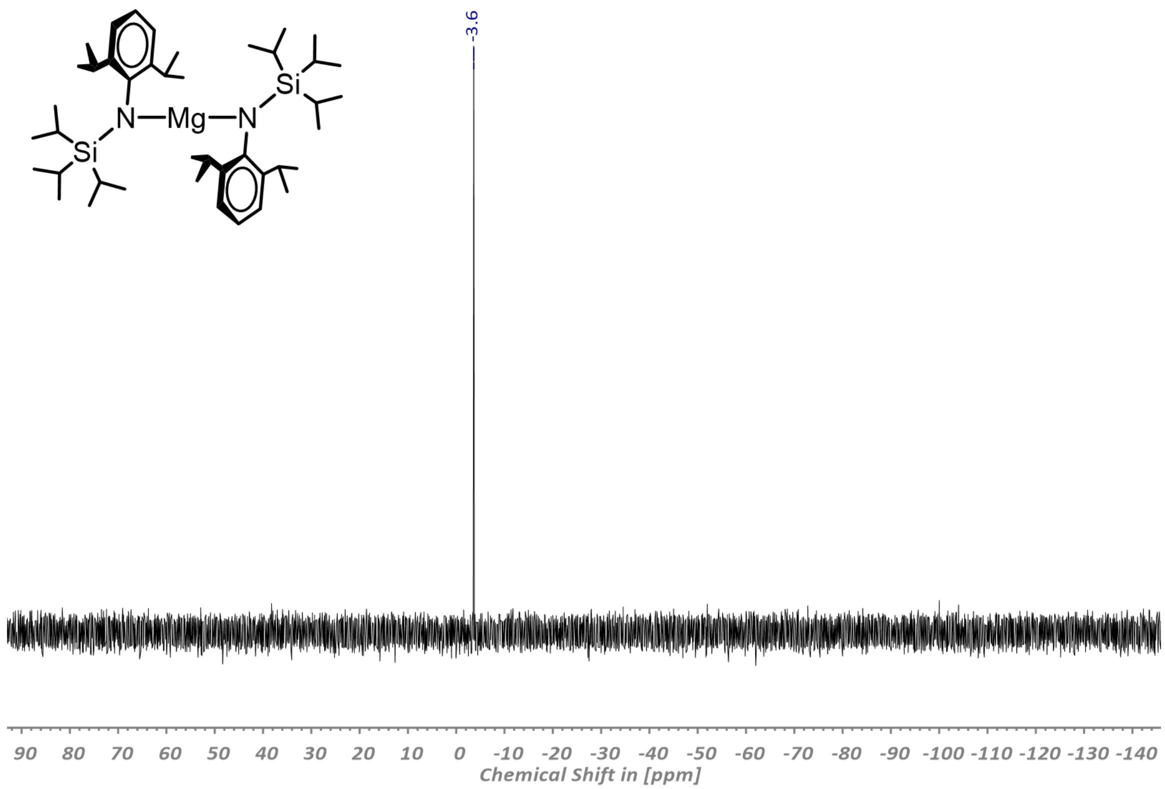

Figure S29. $^{29}Si\{^1H\}$ NMR spectrum (119 MHz, C_6D_6, 22 °C) of Mg[N(DIPP)(Si^{i}Pr$_3$)]$_2$ **(2-Mg)**.

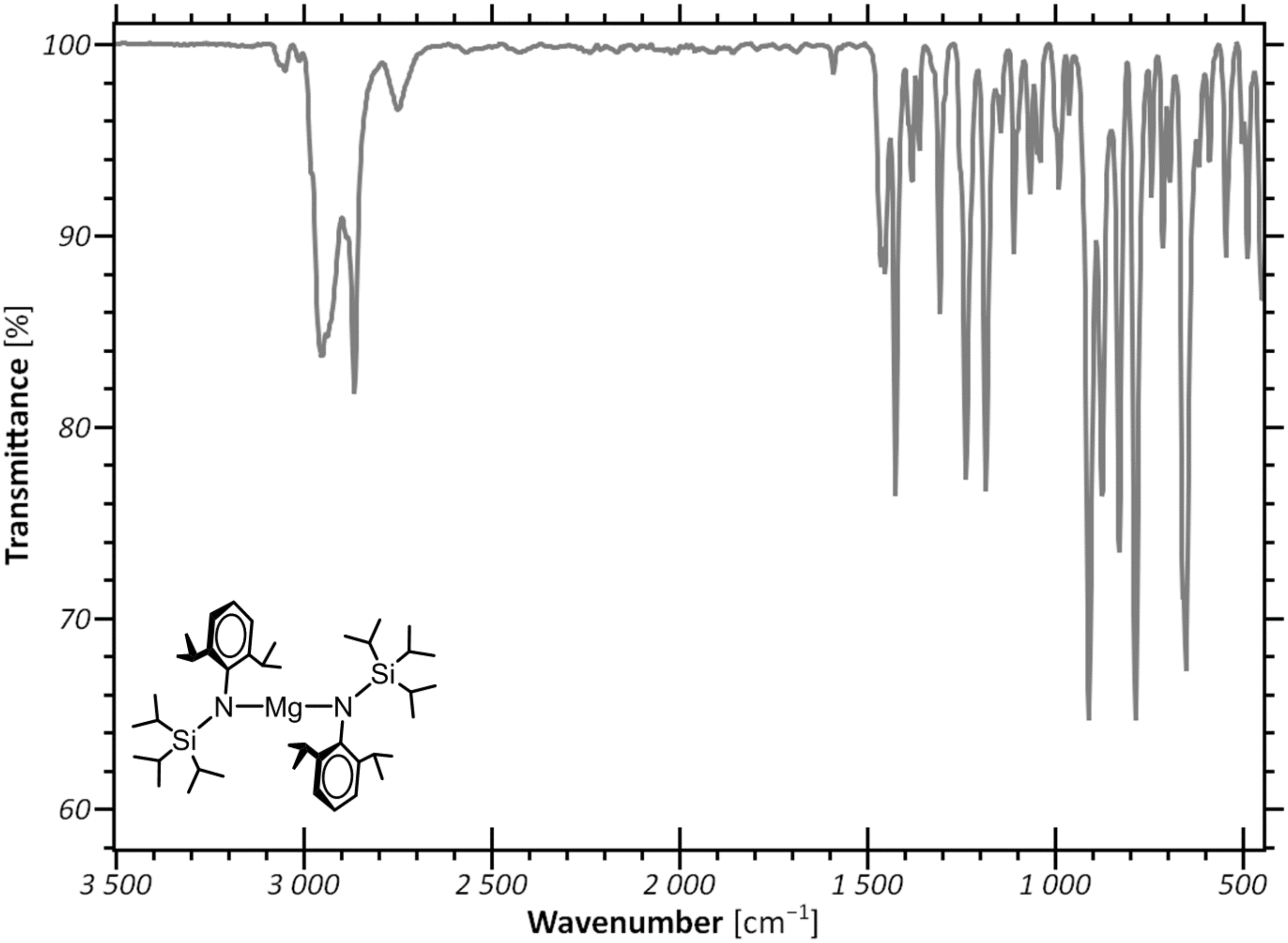

Figure S30. ATR-IR spectrum of Mg[N(DIPP)(Si^{i}Pr$_3$)]$_2$ **(2-Mg)**.

Synthesis of $Ca[N(DIPP)(Si^iPr_3)]_2$ (2-Ca)

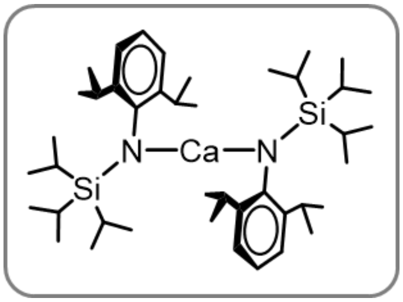

This compound was prepared by two different methods:

Method 1: A suspension of $[KN(DIPP)(Si^iPr_3)]_n$ (1.33 g, 3.58 mmol) and CaI_2 (536 mg, 1.82 mmol) in benzene (8 mL) was stirred at 70 °C for three days. After removing the solvent under a dynamic vacuum, an off-white solid remained, which was dried *in vacuo* at 60 °C for 30 minutes. The residue was extracted with hexane (12 mL), filtered and concentrated to dryness under reduced pressure. The pale yellow crude product was dissolved in hexane (3 mL), filtered and cooled to –20 °C. A uniform batch of block-like crystals of $Ca[N(DIPP)(Si^iPr_3)]_2$ (582 mg, 0.83 mmol, 46%) formed overnight. The supernatant was decanted and the extremely air- and moisture-sensitive crystals dried in vacuum at ambient temperature briefly.

Method 2: Benzene (5 mL) was added to a mixture of $HN(DIPP)(Si^iPr_3)$ (502 mg, 1.50 mmol) and $(p\text{-}^tBuBn)_2Ca$ (268 mg, 0.80 mmol) at room temperature and the resulting orange solution was stirred at 70°C for three days. All volatiles were removed under reduced pressure, and the brown oily residue was dried *in vacuo* at room temperature, dissolved in hexane (0.8 mL) and filtered giving a brown solution. Storing at –20 °C for two weeks afforded pale yellow crystals of $Ca[N(DIPP)(Si^iPr_3)]_2$ (139 mg, 0.20 mmol, 25%), which were collected following decanting the supernatant and drying in high vacuum briefly.

^{1}H NMR (600 MHz, C_6D_6, 25 °C): δ_H = 0.74 (d, ^3J(H,H) = 7.1 Hz, 12H, CH(C*H*$_3$)$_2$), 1.15 (d, ^3J(H,H) = 6.8 Hz, 12H, CH(C*H*$_3$)$_2$), 1.17 – 1.22 (m, 42H, Si(C*H*Me$_2$)$_3$ and Si[CH(C*H*$_3$)$_2$]$_3$), 3.73 (sept, ^3J(H,H) = 6.9 Hz, 4H, C*H*Me$_2$), 6.75 (t, ^3J(H,H) = 7.6 Hz, 2H, *para*-C*H*$_{arom}$), 6.92 (d, ^3J(H,H) = 7.6 Hz, 4H, *meta*-C*H*$_{arom}$) ppm; **^{13}C{^{1}H} NMR** (151 MHz, C_6D_6, 25 °C): δ_C = 15.7 (s, Si(*C*HMe$_2$)$_3$), 20.4 (s, Si[CH(*C*H$_3$)$_2$]$_3$), 24.5 (s, CH(*C*H$_3$)$_2$), 25.7 (s, CH(*C*H$_3$)$_2$), 26.4 (*C*HMe$_2$), 120.6 (s, *para*-*C*$_{arom}$), 126.0 (s, *meta*-*C*$_{arom}$), 143.8 (s, *ortho*-*C*$_{arom}$), 151.7 (s, *ipso*-*C*$_{arom}$) ppm; **^{29}Si{^{1}H} NMR** (119 MHz, C_6D_6, 25 °C): δ_{Si} = –10.3 (s) ppm; **FT-IR** (ATR, pure): $\tilde{\upsilon}$ = 2930 (w), 2860 (w), 1414 (m), 1312 (w), 1240 (m), 1198 (w), 926 (m), 879 (m), 767 (m), 645 (s), 620 (w), 502 (w) cm^{-1}; **Elemental analysis:** calculated (%) for $C_{42}H_{76}CaN_2Si_2$ (705.33 g mol^{-1}): C 71.52, H 10.86, N 3.97; found: C 71.55, H 10.84, N 3.64.

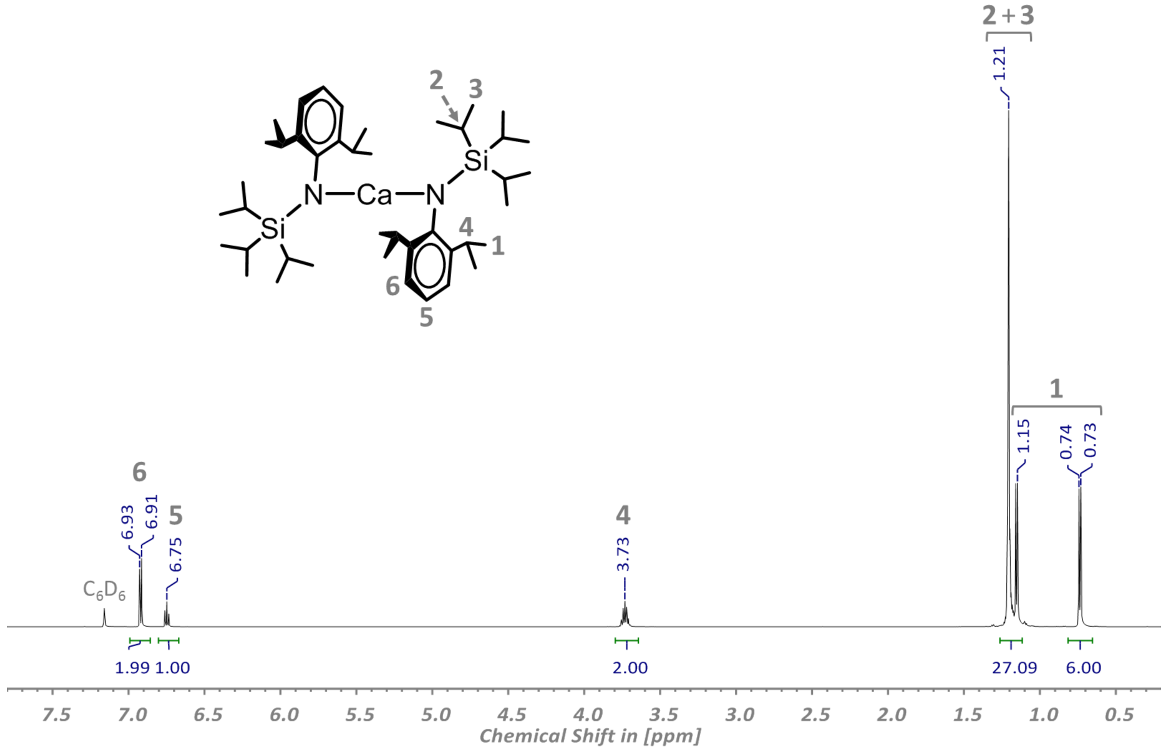

Figure S31. ^{1}H NMR spectrum (600 MHz, C_6D_6, 25 °C) of Ca[N(DIPP)(Si^{i}Pr$_3$)]$_2$ **(2-Ca)**.

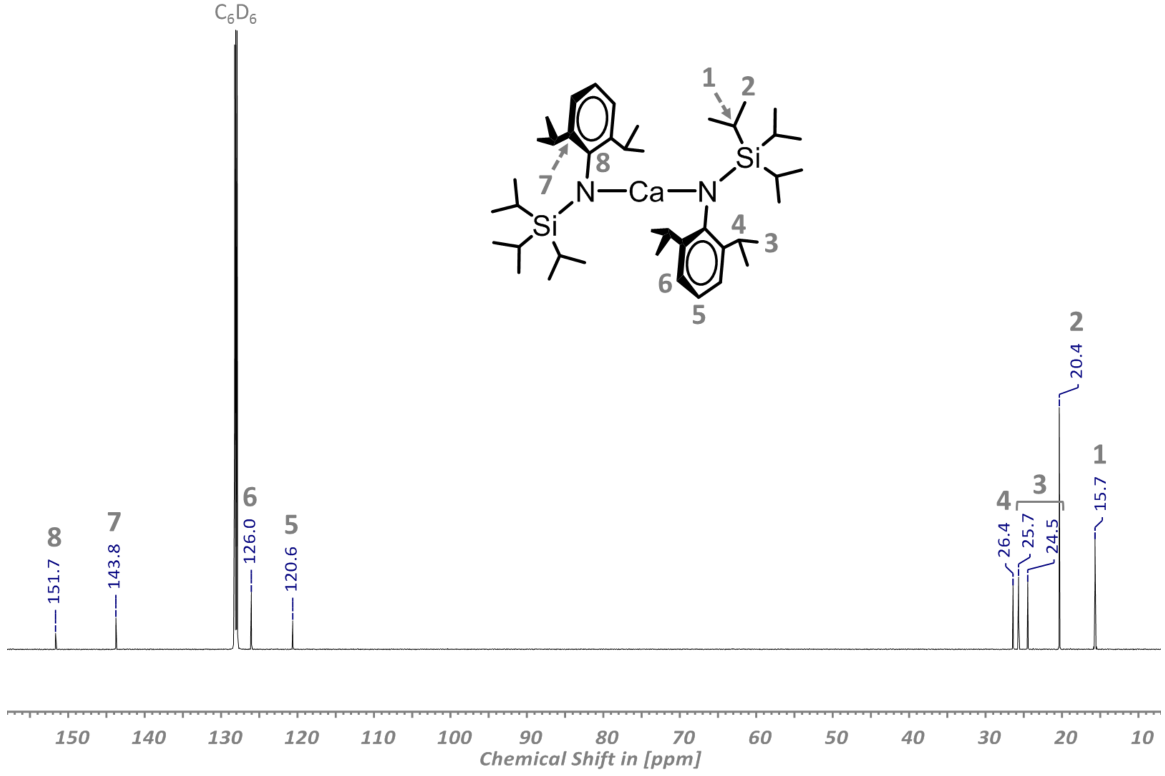

Figure S32. $^{13}C\{^{1}H\}$ NMR (151 MHz, C_6D_6, 25 °C) of Ca[N(DIPP)(Si^{i}Pr$_3$)]$_2$ **(2-Ca)**.

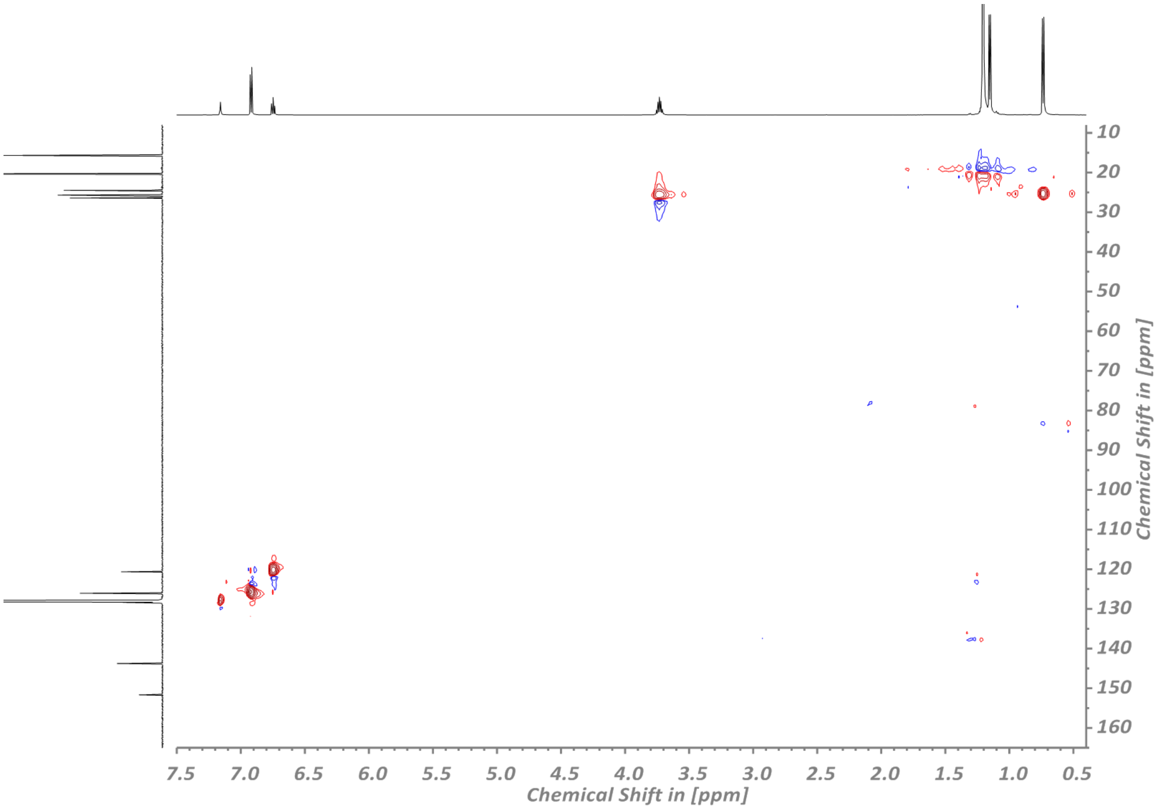

Figure S33. ^{1}H, ^{13}C HSQC NMR spectrum (600 MHz / 151 MHz, C_6D_6, 25 °C) of Ca[N(DIPP)(Si^{i}Pr$_3$)]$_2$ **(2-Ca)**

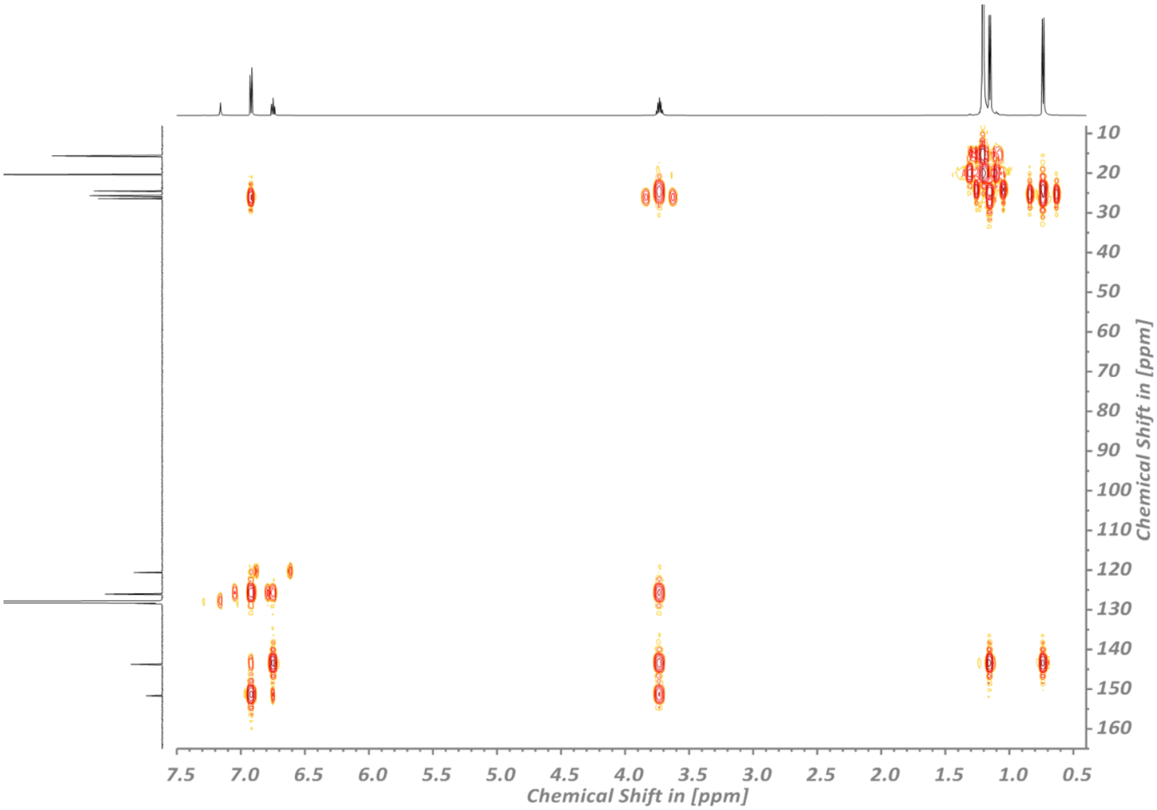

Figure S34. ^{1}H, ^{13}C HMBC NMR spectrum (600 MHz / 151 MHz, C_6D_6, 25 °C) of Ca[N(DIPP)(Si^{i}Pr$_3$)]$_2$ **(2-Ca)**

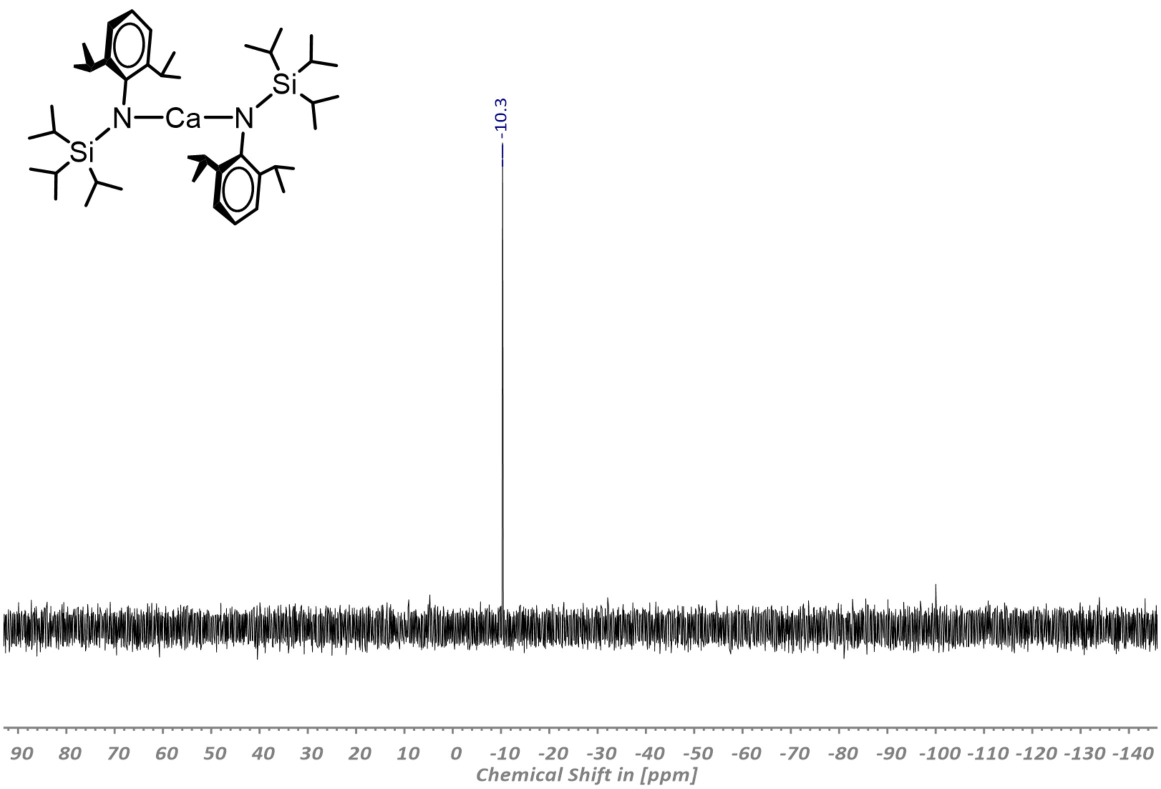

Figure S35. $^{29}Si\{^1H\}$ NMR spectrum (119 MHz, C_6D_6, 25 °C) of $Ca[N(DIPP)(Si^iPr_3)_2]_2$ **(2-Ca)**.

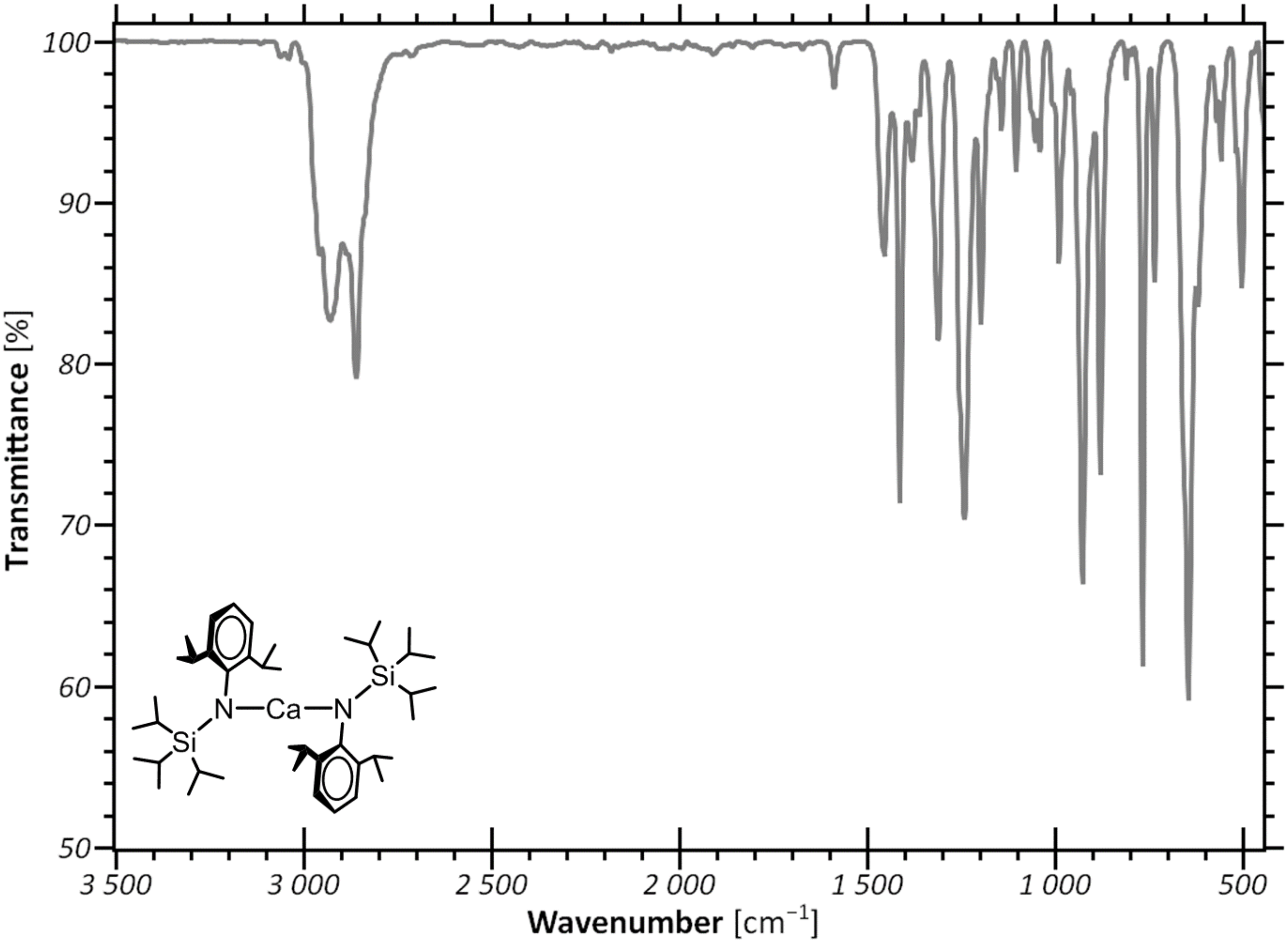

Figure S36. FT-IR ATR spectrum of Ca[N(DIPP)(Si^{i}Pr$_3$)]$_2$ **(2-Ca)**.

Synthesis of Sr[N(DIPP)(Si^{i}Pr$_3$)]$_2$ (2-Sr)

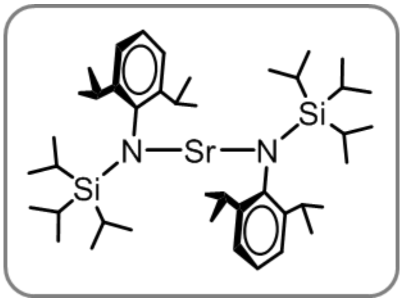

[KN(DIPP)(Si^{i}Pr$_3$)]$_n$ (1.02 g, 2.74 mmol) and SrI_2 (0.47 g, 1.38 mmol) were combined in a dried Schlenk tube and benzene (10 mL) was added yielding a pale yellow suspension. The stirred reaction mixture was then heated at 70 °C for two days, whereupon the solvent was evaporated in a dynamic vacuum. The resultant solid residue was dried in high vacuum (60 °C, 30 minutes), extracted with hexane (12 mL) once and filtered. All volatiles were removed from the filtrate under reduced pressure to give a beige-colored solid, which was dried *in vacuo* and recrystallized from a concentrated hexane solution (1.4 mL) at –20 °C to afford a crop of well-defined colorless blocks. After decantation of the mother liquor and briefly drying *in vacuo* at room temperature, Sr[N(DIPP)(Si^{i}Pr$_3$)]$_2$ (0.45 g, 0.60 mmol) was obtained in 43% yield as ochre crystalline solid.

^{1}H NMR (600 MHz, C_6D_6, 25 °C): δ_H = 0.77 (d, ^3J(H,H) = 7.0 Hz, 12H, CH(CH_3)$_2$), 1.15 (d, ^3J(H,H) = 6.8 Hz, 12H, CH(CH_3)$_2$), 1.16 – 1.19 (m, 42H, Si(CHMe$_2$)$_3$ and Si[CH(CH_3)$_2$]$_3$), 3.66 (sept, ^3J(H,H) = 6.9 Hz, 4H, CHMe$_2$), 6.71 (t, ^3J(H,H) = 7.6 Hz, 2H, *para*-CH_{arom}), 6.94 (d, ^3J(H,H) = 7.6 Hz, 4H, *meta*-CH_{arom}) ppm; **^{13}C{^{1}H} NMR** (151 MHz, C_6D_6, 25 °C): δ_C = 15.9 (s, Si(CHMe$_2$)$_3$), 20.6 (s, Si[CH(CH$_3$)$_2$]$_3$), 24.5 (s, CH(CH$_3$)$_2$), 25.6 (s, CH(CH$_3$)$_2$), 26.5 (CHMe$_2$), 119.3 (s, *para*-C_{arom}), 126.1 (s, *meta*-C_{arom}), 142.9 (s, *ortho*-C_{arom}), 153.0 (s, *ipso*-C_{arom}) ppm; **^{29}Si{^{1}H} NMR** (119 MHz, C_6D_6, 25 °C): δ_{Si} = –12.6 (s) ppm; **FT-IR** (ATR, pure): $\tilde{\upsilon}$ = 2936 (w), 2860 (m), 1410 (m), 1328 (w), 1312 (m), 1259 (m), 1249 (m), 941 (m), 877 (m), 761 (m), 739 (w), 647 (m), 618 (m) cm^{-1}; **Elemental analysis:** calculated (%) for $C_{42}H_{76}N_2Si_2Sr$ (752.87 g mol^{-1}): C 67.00, H 10.18, N 3.72; found: C 66.94, H 10.55, N 3.51.

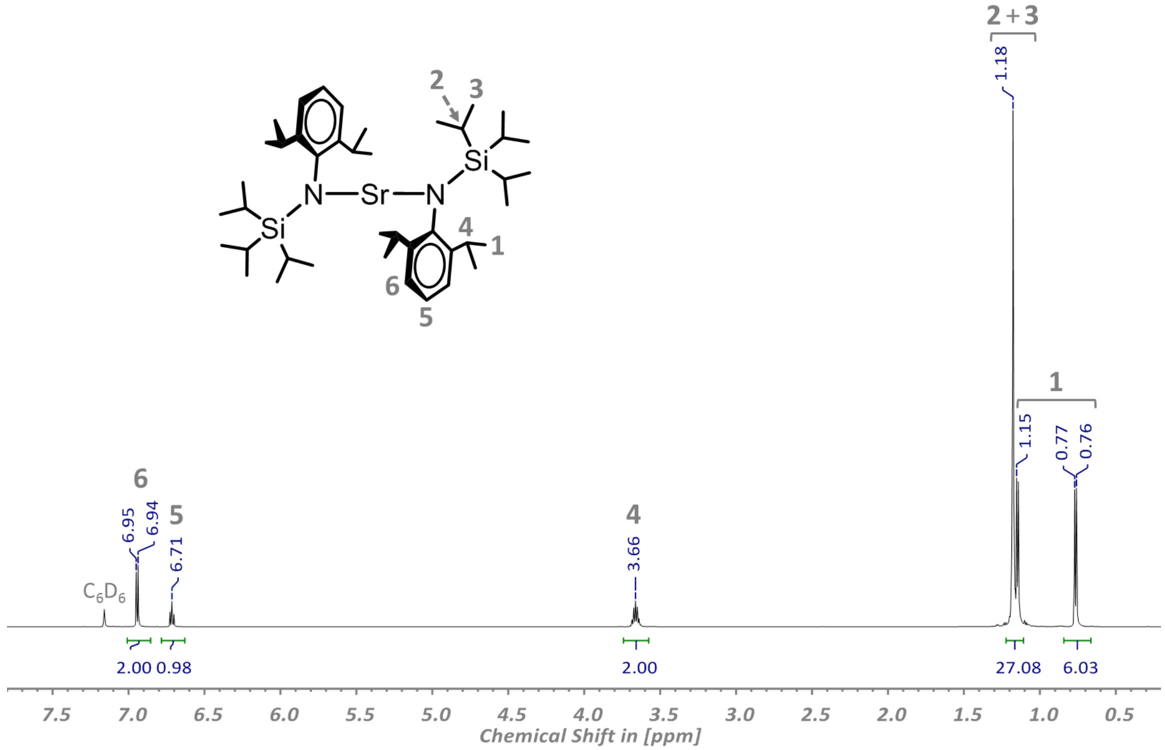

Figure S37. ^{1}H NMR spectrum (600 MHz, C_6D_6, 25 °C) of Sr[N(DIPP)(Si^{i}Pr$_3$)]$_2$ **(2-Sr)**.

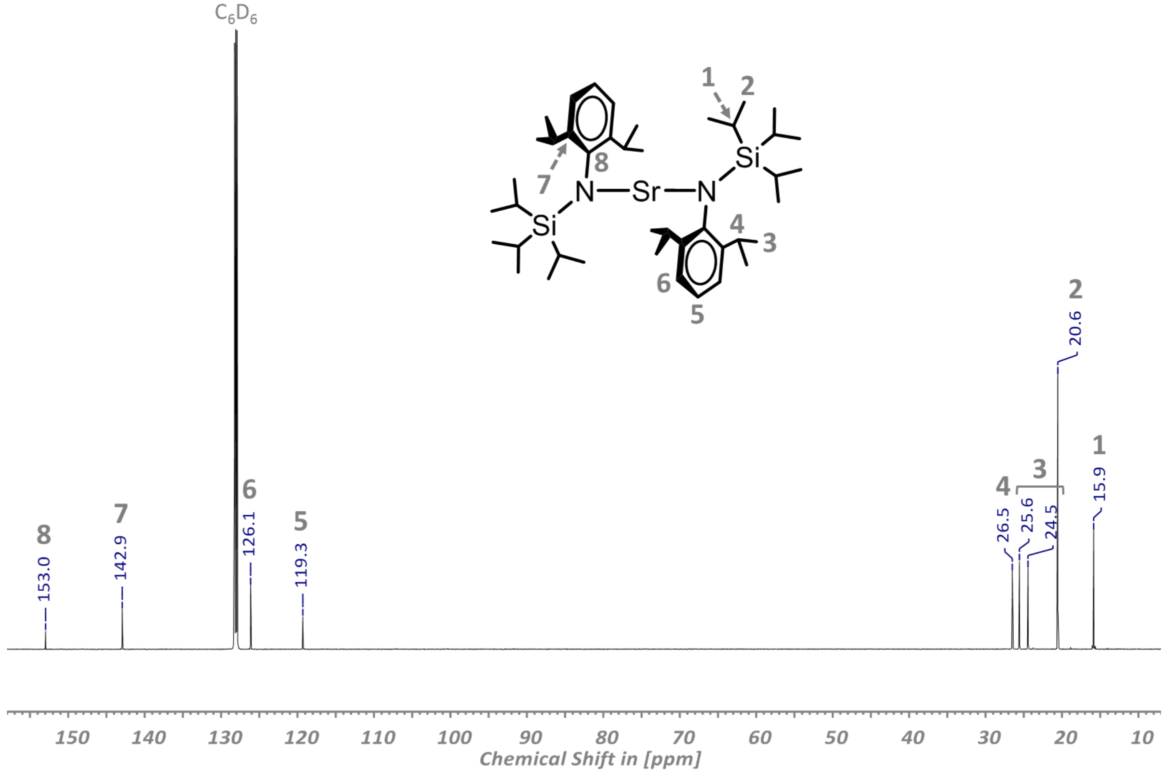

Figure S38. $^{13}C\{^{1}H\}$ NMR (151 MHz, C_6D_6, 25 °C) of Sr[N(DIPP)(Si^{i}Pr$_3$)]$_2$ **(2-Sr)**.

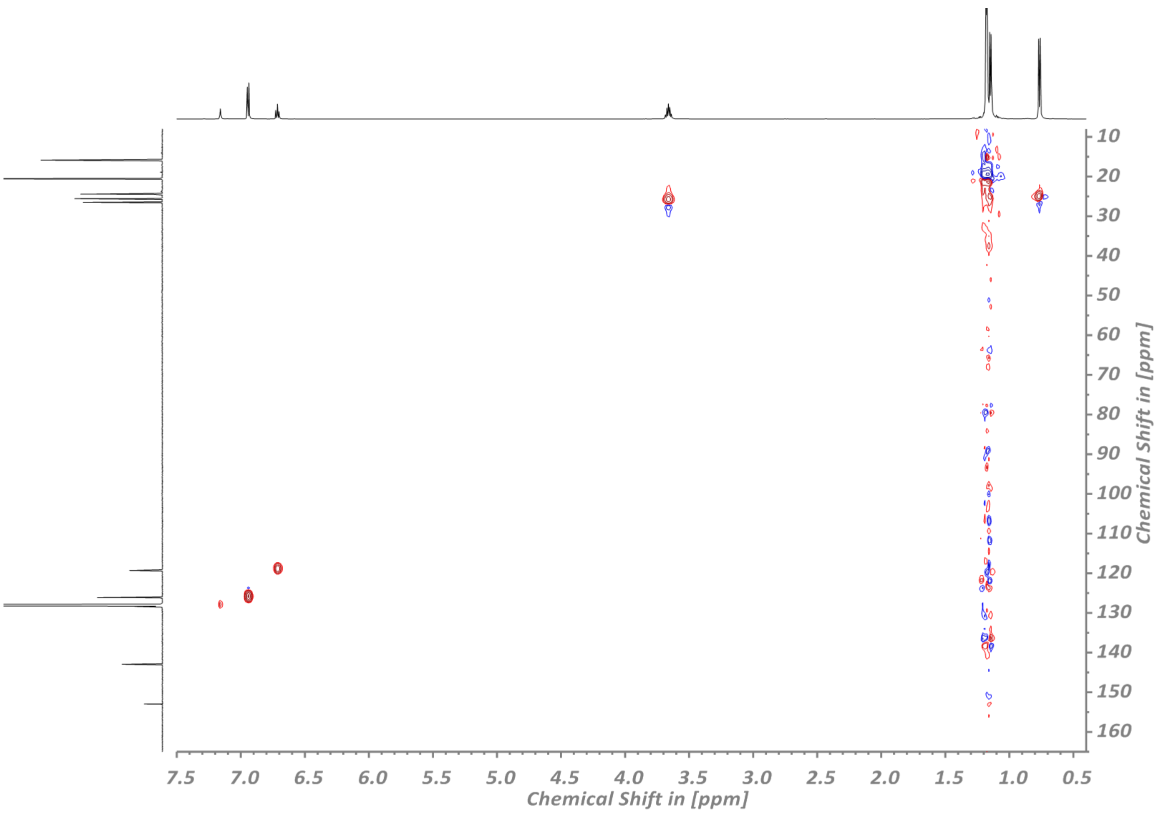

Figure S39. ^{1}H, ^{13}C HSQC NMR spectrum (600 MHz / 151 MHz, C_6D_6, 25 °C) of Sr[N(DIPP)(Si^{i}Pr$_3$)]$_2$ **(2-Sr)**.

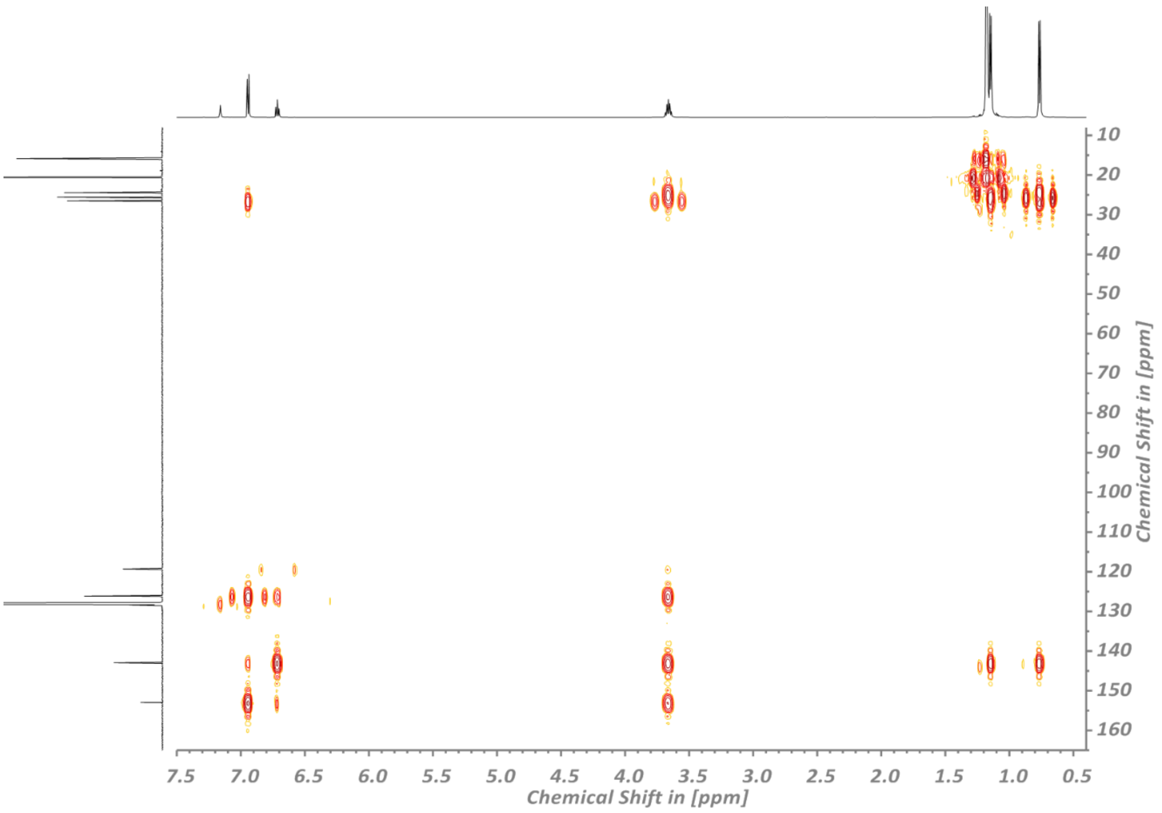

Figure S40. ^{1}H, ^{13}C HMBC NMR spectrum (600 MHz / 151 MHz, C_6D_6, 25 °C) of Sr[N(DIPP)(Si^{i}Pr$_3$)]$_2$ **(2-Sr)**.

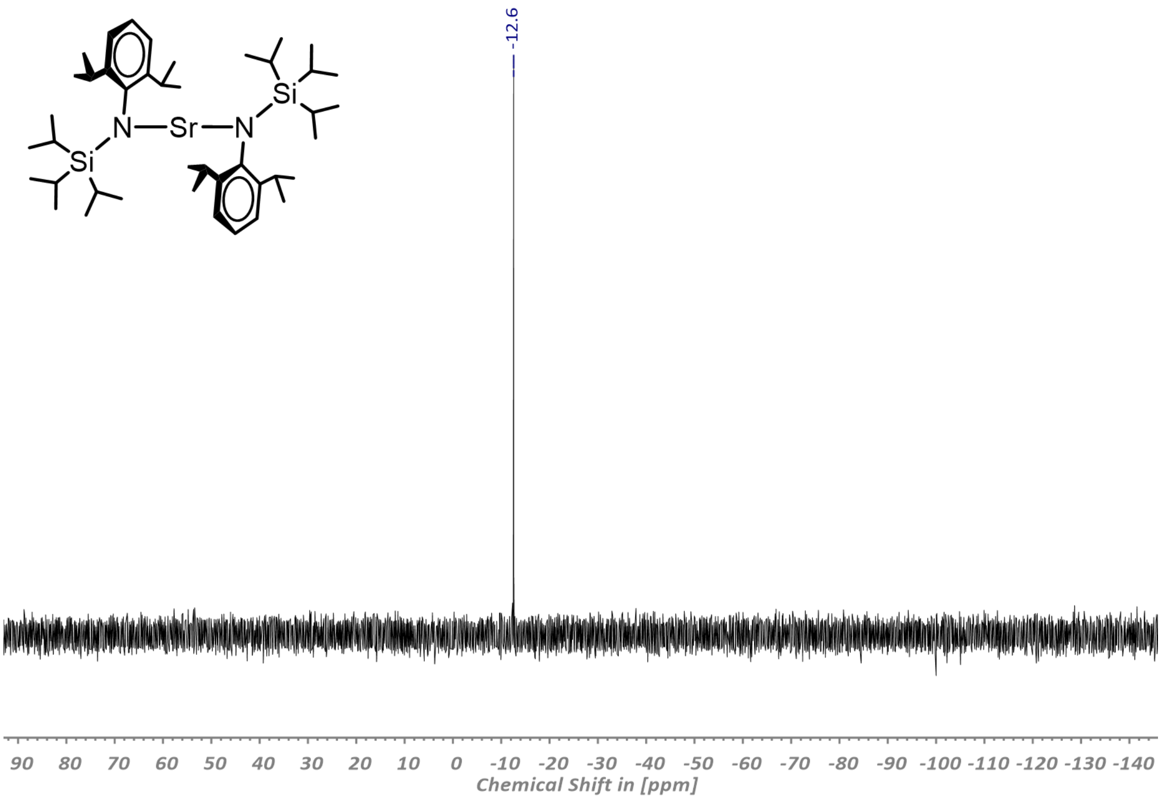

Figure S41. $^{29}Si\{^{1}H\}$ NMR spectrum (119 MHz, C_6D_6, 25 °C) of $Sr[N(DIPP)(Si^{i}Pr_3)_2]_2$ **(2-Sr)**.

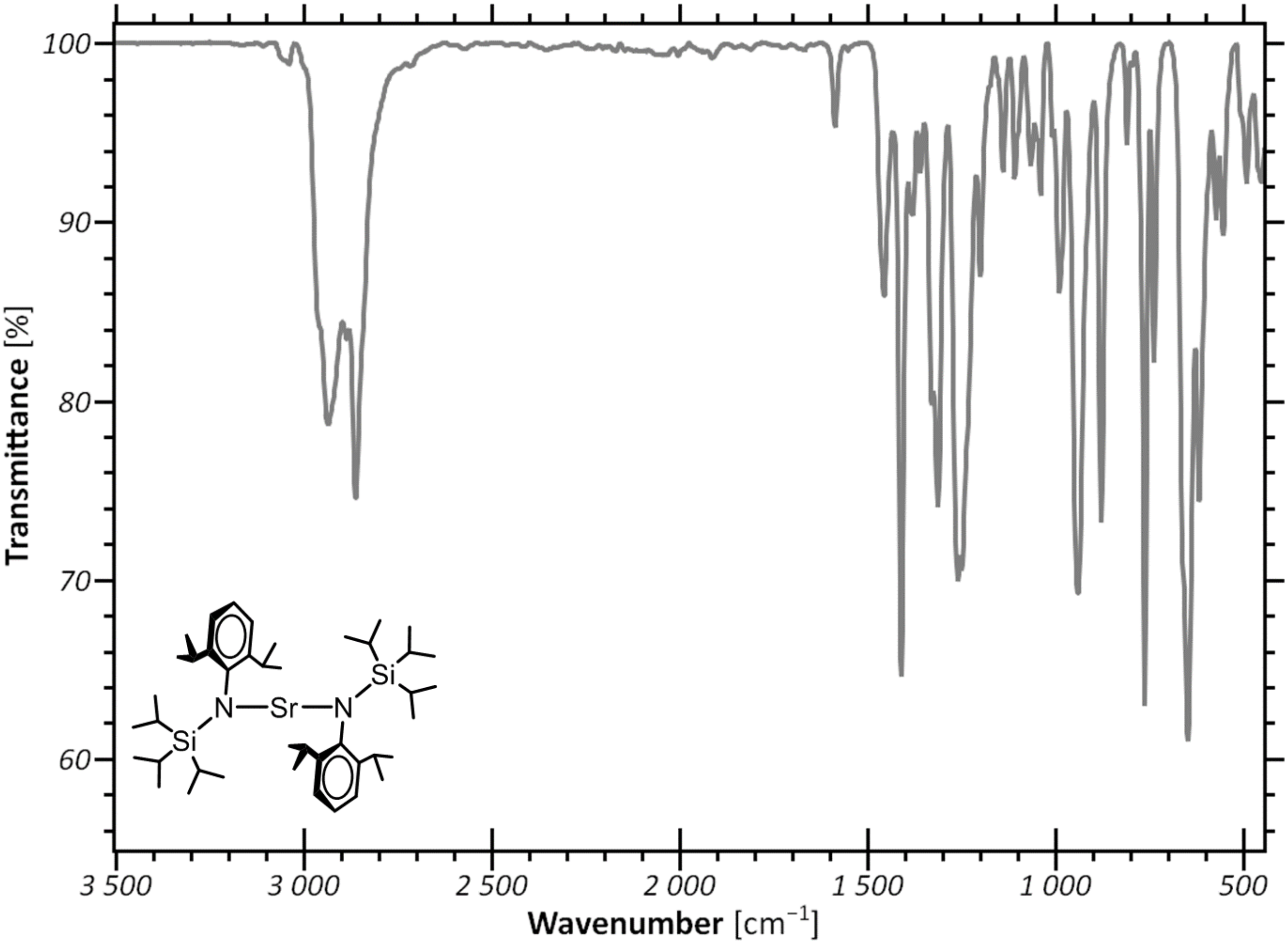

Figure S42. FT-IR ATR spectrum of $Sr[N(DIPP)(Si^{i}Pr_3)]_2$ **(2-Sr)**.

Synthesis of Ba[N(DIPP)(Si^{i}Pr$_3$)]$_2$ (2-Ba)

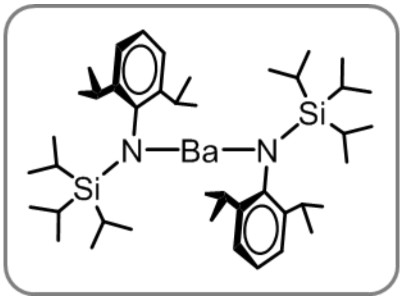

A stirred pale yellow suspension of [KN(DIPP)(Si^{i}Pr$_3$)]$_n$ (1.25 g, 3.36 mmol) and BaI_2 (0.66 g, 1.69 mmol) in benzene (10 mL) was heated at 70 °C over a period of four days. The solvent was removed under reduced pressure to leave a yellowish powder, which was subsequently dried *in vacuo* at 60 °C for 30 minutes. Following extraction with hexane (10 mL) and filtration, the resulting brown solution was concentrated to dryness under a dynamic vacuum. Storage of a concentrated hexane solution (2 mL) at –20 °C overnight resulted in the formation of Ba[N(DIPP)(Si^{i}Pr$_3$)]$_2$ (545 mg, 0.68 mmol, 40%) as extremely air- and moisture-sensitive pale yellow block-like crystals, which were separated from the supernatant by decantation, briefly dried in high vacuum at room temperature and collected.

^{1}H NMR (600 MHz, C_6D_6, 25 °C): δ_H = 0.86 (d, ^3J(H,H) = 7.0 Hz, 12H, CH(C*H*$_3$)$_2$), 1.13 (d, ^3J(H,H) = 6.8 Hz, 12H, CH(C*H*$_3$)$_2$), 1.15 – 1.19 (m, 42H, Si(C*H*Me$_2$)$_3$ and Si[CH(C*H*$_3$)$_2$]$_3$), 3.52 (sept, ^3J(H,H) = 6.9 Hz, 4H, C*H*Me$_2$), 6.68 (t, ^3J(H,H) = 7.5 Hz, 2H, *para*-C*H*$_{arom}$), 6.98 (d, ^3J(H,H) = 7.5 Hz, 4H, *meta*-C*H*$_{arom}$) ppm; **^{13}C{^{1}H} NMR** (151 MHz, C_6D_6, 25 °C): δ_C = 16.2 (s, Si(*C*HMe$_2$)$_3$), 20.7 (s, Si[CH(*C*H$_3$)$_2$]$_3$), 24.6 (s, CH(*C*H$_3$)$_2$), 25.5 (s, CH(*C*H$_3$)$_2$), 26.7 (*C*HMe$_2$), 118.2 (s, *para*-*C*$_{arom}$), 126.2 (s, *meta*-*C*$_{arom}$), 143.1 (s, *ortho*-*C*$_{arom}$), 153.6 (s, *ipso*-*C*$_{arom}$) ppm; **^{29}Si{^{1}H} NMR** (119 MHz, C_6D_6, 25 °C): δ_{Si} = –14.0 (s) ppm; **FT-IR** (ATR, pure): $\tilde{\nu}$ = 2936 (m), 2860 (m), 1455 (w), 1412 (s), 1334 (m), 1316 (m), 1269 (m), 987 (w), 947 (m), 877 (m), 759 (s), 739 (m), 647 (s), 616 (m) cm^{-1}; **Elemental analysis:** calculated (%) for $C_{42}H_{76}BaN_2Si_2$ (802.58 g mol^{-1}): C 62.85, H 9.55, N 3.49; found: C 62.86, H 9.60, N 3.34.

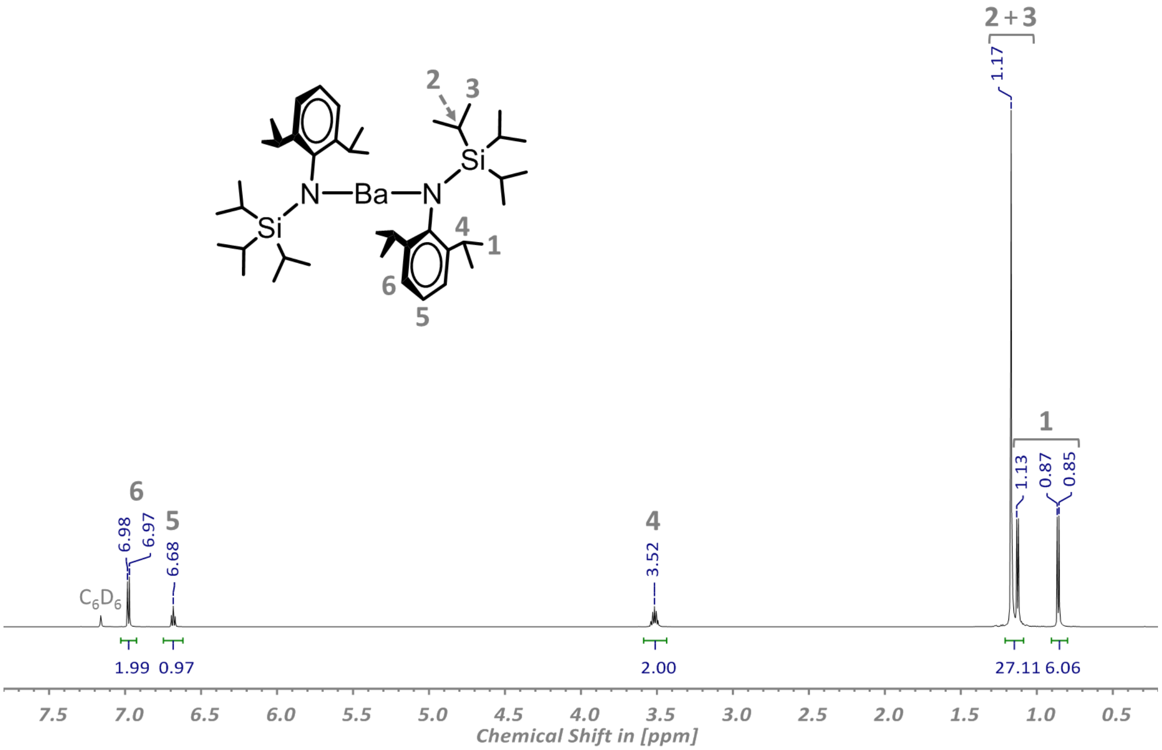

Figure S43. ^{1}H NMR spectrum (600 MHz, C_6D_6, 25 °C) of Ba[N(DIPP)(Si^{i}Pr$_3$)]$_2$ **(2-Ba)**.

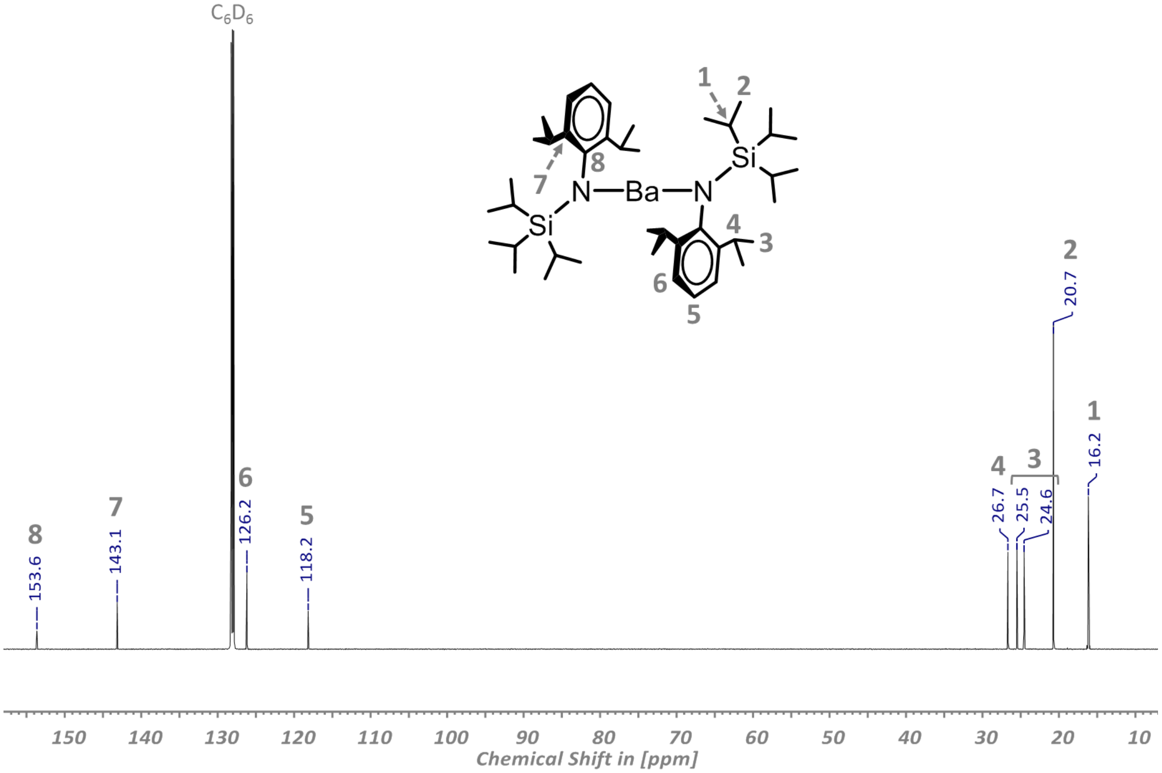

Figure S44. ^{13}C{^{1}H} NMR (151 MHz, C_6D_6, 25 °C) of Ba[N(DIPP)(Si^{i}Pr$_3$)]$_2$ **(2-Ba)**.

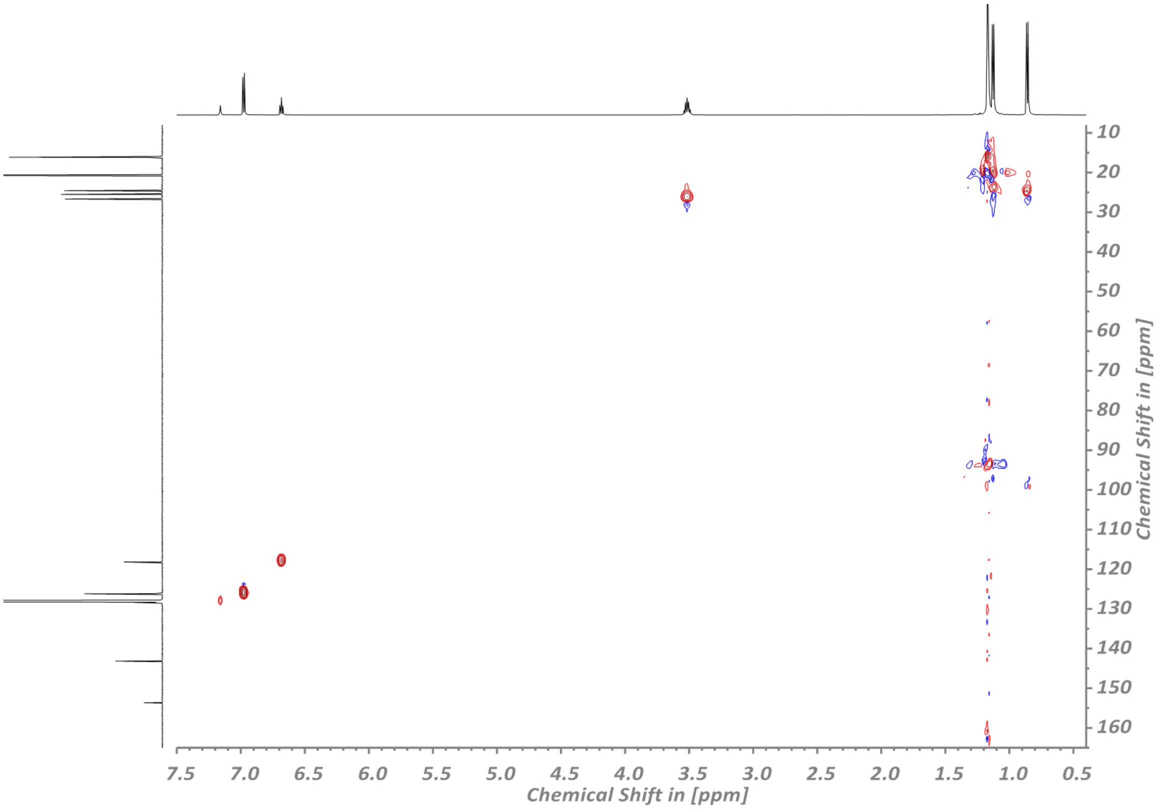

Figure S45. ^{1}H, ^{13}C HSQC NMR spectrum (600 MHz / 151 MHz, C_6D_6, 25 °C) of Ba[N(DIPP)(Si^{i}Pr$_3$)]$_2$ **(2-Ba)**

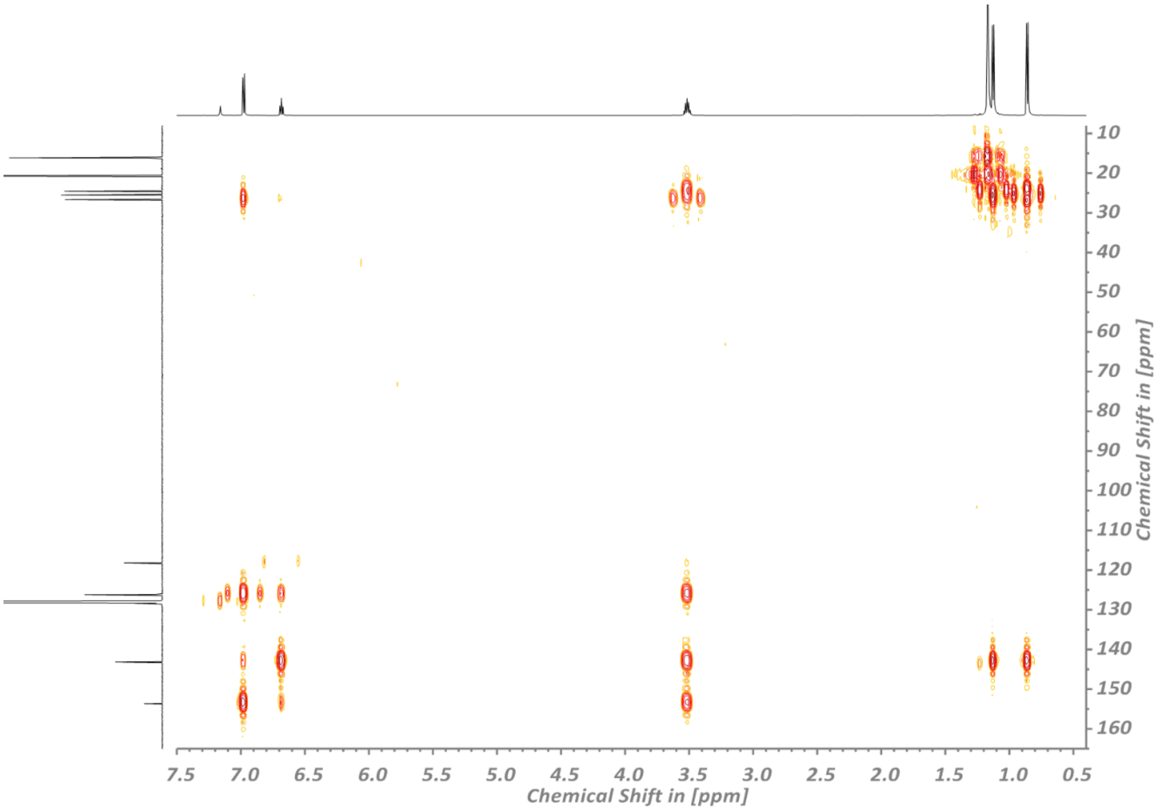

Figure S46. ^{1}H, ^{13}C HMBC NMR spectrum (600 MHz / 151 MHz, C_6D_6, 25 °C) of Ba[N(DIPP)(Si^{i}Pr$_3$)]$_2$ **(2-Ba)**

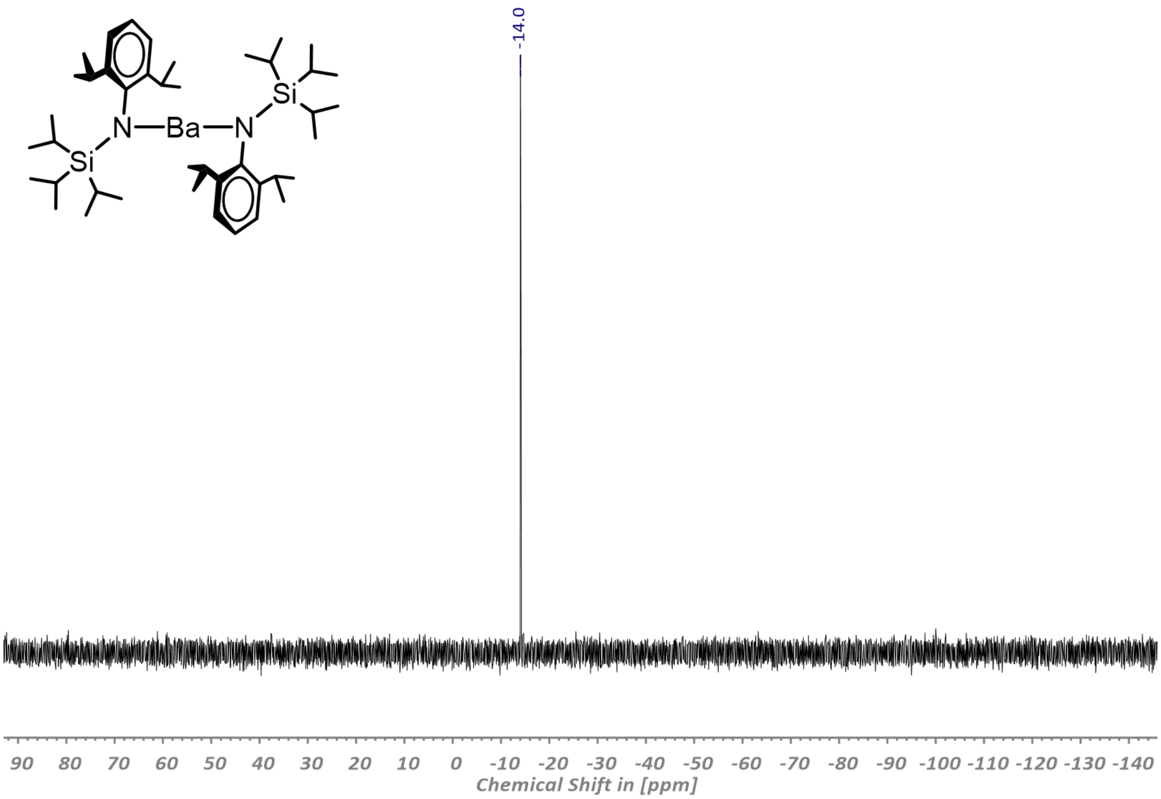

Figure S47. $^{29}Si\{^1H\}$ NMR spectrum (119 MHz, C_6D_6, 25 °C) of Ba[N(DIPP)(Si^{i}Pr$_3$)$_2$]$_2$ **(2-Ba)**.

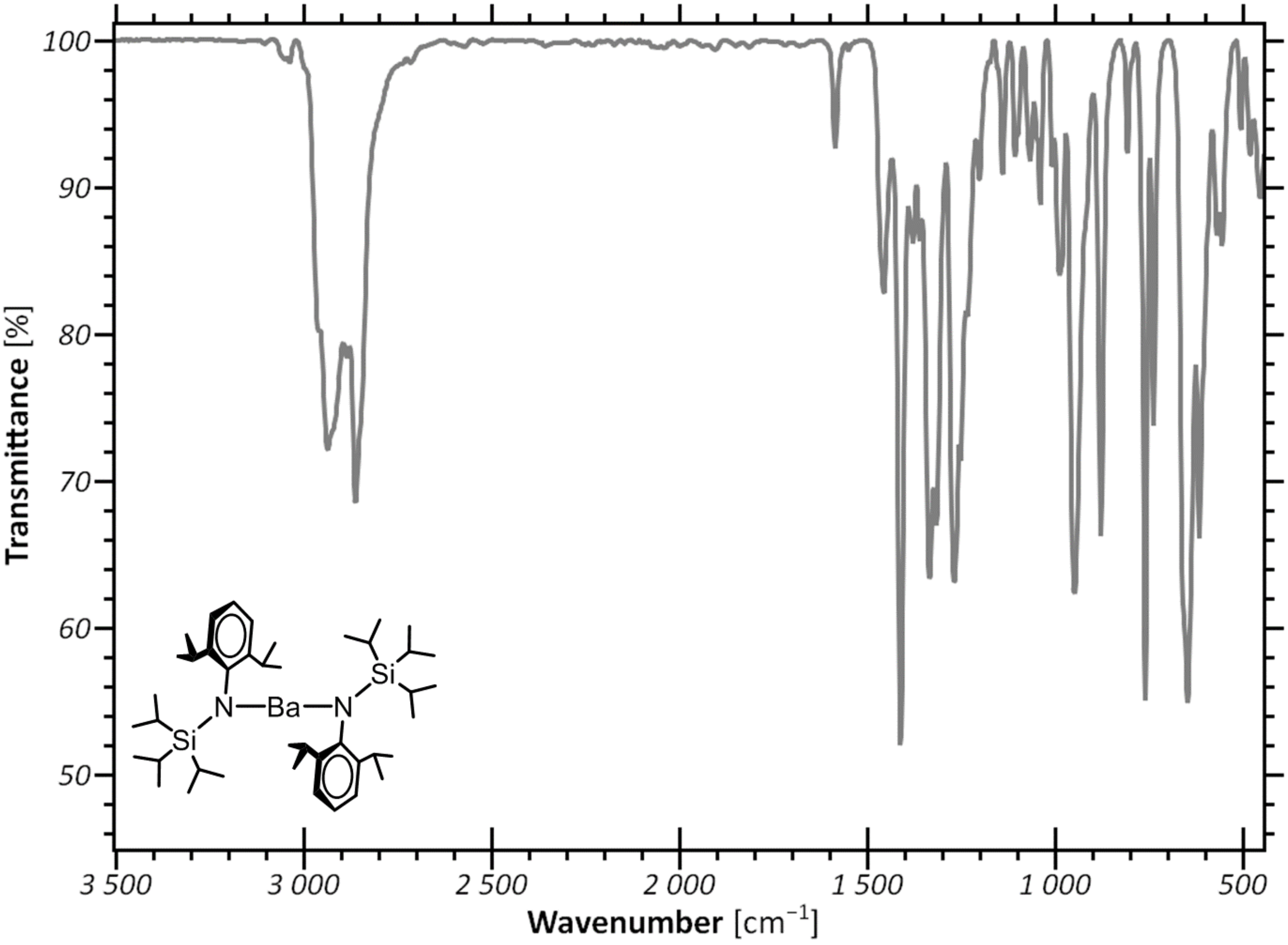

Figure S48. FT-IR ATR spectrum of Ba[N(DIPP)(Si^{i}Pr$_3$)]$_2$ **(2-Ba)**.

3 Crystal Structure Determination

Structure determination of $Mg[N(Si^iPr_3)_2]_2$ (1-Mg)

A colorless crystal of compound $Mg[N(Si^iPr_3)_2]_2$ was embedded in inert perfluoropolyalkylether (viscosity 1800 cSt; ABCR GmbH) and mounted using a Hampton Research CryoLoop. The crystal was then flash cooled to 100.0(2) K in a nitrogen gas stream and kept at this temperature during the experiment. The crystal structure was measured on a SuperNova diffractometer with Atlas detector using a CuKα microfocus source. The measured data was processed with the CrysAlisPro (v39.46) software package.[S10] Using Olex2,[S11] the structure was solved with the ShelXT[S12] structure solution program using Intrinsic Phasing and refined with the ShelXL[S13] refinement package using Least Squares minimization. All non-hydrogen atoms were refined anisotropically. All hydrogen atoms were placed in ideal positions and refined as riding atoms with relative isotropic displacement parameters.

The crystal structure data has been deposited with the Cambridge Crystallographic Data Centre. CCDC 1970927 contains the supplementary crystallographic data for complex $Mg[N(Si^iPr_3)_2]_2$. This data can be obtained free of charge from the Cambridge Crystallographic Data Centre *via* www.ccdc.cam.ac.uk/data_request/cif.

Crystallographic and refinement data are summarized in Table S1.

Structure determination of $Sr[N(Si^iPr_3)_2]_2$ (1-Sr)

A colorless crystal of compound $Sr[N(Si^iPr_3)_2]_2$ was embedded in inert perfluoropolyalkylether (viscosity 1800 cSt; ABCR GmbH) and mounted using a Hampton Research CryoLoop. The crystal was then flash cooled to 100.0(1) K in a nitrogen gas stream and kept at this temperature during the experiment. The crystal structure was measured on a SuperNova diffractometer with Atlas detector using a CuKα microfocus source. The measured data was processed with the CrysAlisPro (v40.18b) software package.[S10] Using Olex2,[S11] the structure was solved with the ShelXT[S12] structure solution program using Intrinsic Phasing and refined with the ShelXL[S13] refinement package using Least Squares minimization. All non-hydrogen atoms were refined anisotropically. All hydrogen atoms were placed in ideal positions and refined as riding atoms with relative isotropic displacement parameters.

Disorder of the Sr atom was observed and modeled. The relative occupancies of the two alternative positions were refined to 0.861(13) and 0.139(13).

The crystal structure data has been deposited with the Cambridge Crystallographic Data Centre. CCDC 1970928 contains the supplementary crystallographic data for complex $Sr[N(Si^{i}Pr_3)_2]_2$. This data can be obtained free of charge from the Cambridge Crystallographic Data Centre *via* www.ccdc.cam.ac.uk/data_request/cif.

Crystallographic and refinement data are summarized in Table S1.

Structure determination of $Ba[N(Si^{i}Pr_3)_2]_2$ (1Ba)

A colorless crystal of compound $Ba[N(Si^{i}Pr_3)_2]_2$ was embedded in inert perfluoropolyalkylether (viscosity 1800 cSt; ABCR GmbH) and mounted using a Hampton Research CryoLoop. The crystal was then flash cooled to 100.0(1) K in a nitrogen gas stream and kept at this temperature during the experiment. The crystal structure was measured on a SuperNova diffractometer with Atlas detector using a CuKα microfocus source. The measured data was processed with the CrysAlisPro (v40.18b) software package.[S10] Using Olex2,[S11] the structure was solved with the ShelXT[S12] structure solution program using Intrinsic Phasing and refined with the ShelXL[S13] refinement package using Least Squares minimization. All non-hydrogen atoms were refined anisotropically. All hydrogen atoms were placed in ideal positions and refined as riding atoms with relative isotropic displacement parameters.

The crystal structure data has been deposited with the Cambridge Crystallographic Data Centre. CCDC 1970929 contains the supplementary crystallographic data for complex $Ba[N(Si^{i}Pr_3)_2]_2$. This data can be obtained free of charge from the Cambridge Crystallographic Data Centre *via* www.ccdc.cam.ac.uk/data_request/cif.

Crystallographic and refinement data are summarized in Table S1.

Structure determination of {$Ba[N(Si^{i}Pr_3)_2]_2$(3,4-dihydro-*2H*-pyran)}

A colorless crystal of compound {$Ba[N(Si^{i}Pr_3)_2]_2$(3,4-dihydro-*2H*-pyran)} was embedded in inert perfluoropolyalkylether (viscosity 1800 cSt; ABCR GmbH) and mounted using a Hampton Research CryoLoop. The crystal was then flash cooled to 100.0(2) K in a nitrogen gas stream and kept at this

temperature during the experiment. The crystal structure was measured on a SuperNova diffractometer with Atlas detector using a CuKα microfocus source. The measured data was processed with the CrysAlisPro (v40.18b) software package.[S10] Using Olex2,[S11] the structure was solved with the ShelXT[S12] structure solution program using Intrinsic Phasing and refined with the ShelXL[S13] refinement package using Least Squares minimization. All non-hydrogen atoms were refined anisotropically. All hydrogen atoms were placed in ideal positions and refined as riding atoms with relative isotropic displacement parameters.

The unit cell contains three half molecules, which are located on two-fold rotational axes. While the O atoms are located directly on these axes, the rest the 3,4-dihydro-*2H*-pyran moieties are disordered around these symmetry elements. A suitable disorder model was built with the help of similarity restraints (SIMU, SADI) and rigid bond restraints (RIGU).[S14]

The crystal structure data has been deposited with the Cambridge Crystallographic Data Centre. CCDC 1970937 contains the supplementary crystallographic data for complex {$Ba[N(Si^iPr_3)_2]_2$(3,4-dihydro-*2H*-pyran)}. This data can be obtained free of charge from the Cambridge Crystallographic Data Centre *via* www.ccdc.cam.ac.uk/data_request/cif.

Crystallographic and refinement data are summarized in Table S1.

Structure determination of $[KN(DIPP)(Si^iPr_3)]_n$ (2-K)

A yellow crystal of compound $[KN(DIPP)(Si^iPr_3)]_n$ was embedded in inert perfluoropolyalkylether (viscosity 1800 cSt; ABCR GmbH) and mounted using a Hampton Research CryoLoop. The crystal was then flash cooled to 100.0(1) K in a nitrogen gas stream and kept at this temperature during the experiment. The crystal structure was measured on a SuperNova diffractometer with Atlas detector using a CuKα microfocus source. The measured data was processed with the CrysAlisPro (v37.35) software package.[S10] Using Olex2,[S11] the structure was solved with the ShelXT[S12] structure solution program using Intrinsic Phasing and refined with the ShelXL[S13] refinement package using Least Squares minimization. All non-hydrogen atoms were refined anisotropically. All hydrogen atoms were placed in ideal positions and refined as riding atoms with relative isotropic displacement parameters.

Disorder of the *i*Pr groups was observed, and was modeled with the help of similarity restraints (SIMU, SADI) and rigid bond restraints (RIGU).[S14] The relative occupancies of the two alternative orientations were refined to 0.917(2) and 0.083(2).

The crystal structure data has been deposited with the Cambridge Crystallographic Data Centre. CCDC 1970935 contains the supplementary crystallographic data for complex $[KN(DIPP)(Si^iPr_3)]_n$. This data can be obtained free of charge from the Cambridge Crystallographic Data Centre *via* www.ccdc.cam.ac.uk/data_request/cif.

Crystallographic and refinement data are summarized in Table S1.

Structure determination of $Mg[N(DIPP)(Si^iPr_3)]_2$ (2-Mg)

A colorless crystal of compound $Mg[N(DIPP)(Si^iPr_3)]_2$ was embedded in inert perfluoropolyalkylether (viscosity 1800 cSt; ABCR GmbH) and mounted using a Hampton Research CryoLoop. The crystal was then flash cooled to 100.0(1) K in a nitrogen gas stream and kept at this temperature during the experiment. The crystal structure was measured on a SuperNova diffractometer with Atlas detector using a CuKα microfocus source. The measured data was processed with the CrysAlisPro (v40.18b) software package.[S10] Using Olex2,[S11] the structure was solved with the ShelXT[S12] structure solution program using Intrinsic Phasing and refined with the ShelXL[S13] refinement package using Least Squares minimization. All non-hydrogen atoms were refined anisotropically. All hydrogen atoms were placed in ideal positions and refined as riding atoms with relative isotropic displacement parameters.

The crystal under investigation was a racemic twin. The fractional contribution of the two twin domains were refined to 0.596(17) and 0.404(17), respectively.

The crystal structure data has been deposited with the Cambridge Crystallographic Data Centre. CCDC 1970930 contains the supplementary crystallographic data for complex $Mg[N(DIPP)(Si^iPr_3)]_2$. This data can be obtained free of charge from the Cambridge Crystallographic Data Centre *via* www.ccdc.cam.ac.uk/data_request/cif.

Crystallographic and refinement data are summarized in Table S1.

Structure determination of Ca[N(DIPP)(Si^{i}Pr$_3$)]$_2$ (2-Ca)

A pale yellow crystal of compound Ca[N(DIPP)(Si^{i}Pr$_3$)]$_2$ was embedded in inert perfluoropolyalkylether (viscosity 1800 cSt; ABCR GmbH) and mounted using a Hampton Research CryoLoop. The crystal was then flash cooled to 100.0(1) K in a nitrogen gas stream and kept at this temperature during the experiment. The crystal structure was measured on a SuperNova diffractometer with Atlas detector using a CuKα microfocus source. The measured data was processed with the CrysAlisPro (v40.18b) software package.[S10] Using Olex2, [S11] the structure was solved with the ShelXT[S12] structure solution program using Intrinsic Phasing and refined with the ShelXL[S13] refinement package using Least Squares minimization. All non-hydrogen atoms were refined anisotropically. All hydrogen atoms were placed in ideal positions and refined as riding atoms with relative isotropic displacement parameters.

Disorder of the Ca atom was observed and was modeled with the help of similarity restraints (SIMU). The relative occupancies of the two alternative positions were refined to 0.913(11) and 0.087(11).

The crystal structure data has been deposited with the Cambridge Crystallographic Data Centre. CCDC 1970931 contains the supplementary crystallographic data for complex Ca[N(DIPP)(Si^{i}Pr$_3$)]$_2$. This data can be obtained free of charge from the Cambridge Crystallographic Data Centre *via* www.ccdc.cam.ac.uk/data_request/cif.

Crystallographic and refinement data are summarized in Table S1.

Structure determination of Sr[N(DIPP)(Si^{i}Pr$_3$)]$_2$ (2-Sr)

A colorless crystal of compound Sr[N(DIPP)(Si^{i}Pr$_3$)]$_2$ was embedded in inert perfluoropolyalkylether (viscosity 1800 cSt; ABCR GmbH) and mounted using a Hampton Research CryoLoop. The crystal was then flash cooled to 100.0(2) K in a nitrogen gas stream and kept at this temperature during the experiment. The crystal structure was measured on a SuperNova diffractometer with Atlas detector using a CuKα microfocus source. The measured data was processed with the CrysAlisPro (v40.53) software package.[S10] Using Olex2,[S11] the structure was solved with the ShelXT[S12] structure solution program using Intrinsic Phasing and refined with the ShelXL[S13] refinement package using Least Squares minimization. All non-hydrogen atoms were refined anisotropically. All hydrogen atoms were placed in ideal positions and refined as riding atoms with relative isotropic displacement parameters.

The crystal structure data has been deposited with the Cambridge Crystallographic Data Centre. CCDC 1970932 contains the supplementary crystallographic data for complex Sr[N(DIPP)(Si^{i}Pr$_3$)]$_2$. This data can be obtained free of charge from the Cambridge Crystallographic Data Centre *via* www.ccdc.cam.ac.uk/data_request/cif.

Crystallographic and refinement data are summarized in Table S1.

Structure determination of Ba[N(DIPP)(Si^{i}Pr$_3$)]$_2$ (2-Ba)

A pale yellow crystal of compound Ba[N(DIPP)(Si^{i}Pr$_3$)]$_2$ was embedded in inert perfluoropolyalkylether (viscosity 1800 cSt; ABCR GmbH) and mounted using a Hampton Research CryoLoop. The crystal was then flash cooled to 100.0(1) K in a nitrogen gas stream and kept at this temperature during the experiment. The crystal structure was measured on a SuperNova diffractometer with Atlas detector using a CuKα microfocus source. The measured data was processed with the CrysAlisPro (v40.53) software package.[S10] Using Olex2,[S11] the structure was solved with the ShelXT[S12] structure solution program using Intrinsic Phasing and refined with the ShelXL[S13] refinement package using Least Squares minimization. The unit cell contained eight symmetry independent molecules of compound Ba[N(DIPP)(Si^{i}Pr$_3$)]$_2$. All non-hydrogen atoms were refined anisotropically. All hydrogen atoms were placed in ideal positions and refined as riding atoms with relative isotropic displacement parameters.

The crystal structure data has been deposited with the Cambridge Crystallographic Data Centre. CCDC 1970933 contains the supplementary crystallographic data for complex Ba[N(DIPP)(Si^{i}Pr$_3$)]$_2$. This data can be obtained free of charge from The Cambridge Crystallographic Data Centre *via* www.ccdc.cam.ac.uk/data_request/cif.

Crystallographic and refinement data are summarized in Table S1.

Table S1. Crystallographic data and structure refinement for **(1-Ae)** (Ae = Mg, Sr, Ba) and **(2-Ba) (3,4-dihydro-2*H*-pyran)**.

**Compound**	**Mg[N(Si^{i}Pr$_3$)$_2$]$_2$ (1-Mg)**	**Sr[N(Si^{i}Pr$_3$)$_2$]$_2$ (1-Sr)**	**Ba[N(Si^{i}Pr$_3$)$_2$]$_2$ (1-Ba)**	**Ba[N(Si^{i}Pr$_3$)$_2$]$_2$ x 3,4-Dihydro-2*H*-pyran**
**Empirical formula**	$C_{36}H_{84}MgN_2Si_4$	$C_{36}H_{84}SrN_2Si_4$	$C_{36}H_{84}BaN_2Si_4$	$C_{41}H_{92}BaN_2OSi_4$
**Formula weight** [g mol^{-1}]	681.72	745.03	794.75	878.86
**Temperature** [K]	100.0(1)	100.0(1)	100.0(1)	100.0(2)
**Crystal system**	monoclinic	orthorhombic	monoclinic	monoclinic
**Space group**	$C2/c$	$Pbca$	$C2/c$	$C2/c$
$\boldsymbol{a}$ [Å]	21.1356(2)	20.5063(3)	25.1493(6)	22.9869(3)
$\boldsymbol{b}$ [Å]	10.48840(10)	16.0133(2)	8.28520(19)	36.7901(3)
$\boldsymbol{c}$ [Å]	19.76480(10)	26.4121(3)	22.2957(5)	17.06968(19)
$\boldsymbol{\alpha}$ [°]	90	90	90	90
$\boldsymbol{\beta}$ [°]	105.4880(10)	90	109.923(3)	90.4702(10)
$\boldsymbol{\gamma}$ [°]	90	90	90	90
**Volume** [Å^3]	4222.33(6)	8673.03(19)	4367.66(19)	14435.1(3)
**Z**	4	8	4	12
$\boldsymbol{\rho_{Cald}}$ [g cm^{-3}]	1.072	1.141	1.209	1.213
$\boldsymbol{\mu}$ [mm^{-1}]	1.623	2.953	1.044	0.955
**F(000)**	1528.0	3264.0	1704.0	5664.0
**Crystal size** [mm^3]	0.344 × 0.243 × 0.143	0.174 x 0.127 x 0.114	0.388 x 0.195 x 0.103	0.427 x 0.198 x 0.096
**Radiation**	Cu Kα (λ = 1.54184)	CuKα (λ = 1.54184)	MoKα (λ = 0.71073)	MoKα (λ = 0.71073)
**2Θ range for data collection** [°]	8.682 to 147.362	6.694 to 145.264	5.21 to 59.04	4.178 to 58.956
**Index ranges**	-25 ≤ h ≤ 26<br>-9 ≤ k ≤ 12<br>-24 ≤ l ≤ 24	-24 ≤ h ≤ 24<br>-19 ≤ k ≤ 18<br>-30 ≤ l ≤ 32	-31 ≤ h ≤ 34<br>-11 ≤ k ≤ 10<br>-28 ≤ l ≤ 29	-30 ≤ h ≤ 26<br>-50 ≤ k ≤ 48<br>-23 ≤ l ≤ 23
**Reflections collected**	16404	33995	20213	66851
**Independent reflections**	4170	8443	5504	17959
$\mathbf{R_{int}}$	0.0233	0.0305	0.0254	0.0254
**Data / restraints / parameters**	4170 / 0 / 207	8443 / 0 / 422	5504 / 0 / 207	17959 / 389 / 799
**Goodness-of-fit on F^2**	1.054	1.047	1.052	1.024
**Final R indexes** [$I \geq 2\sigma\ (I)$]	R_1 = 0.0307<br>wR_2 = 0.0828	R_1 = 0.0246<br>wR_2 = 0.0600	R_1 = 0.0215<br>wR_2 = 0.0491	R_1 = 0.0270<br>wR_2 = 0.0608
**Final R indexes** [all data]	R_1 = 0.0316<br>wR_2 = 0.0835	R_1 = 0.0291<br>wR_2 = 0.0622	R_1 = 0.0246<br>wR_2 = 0.0506	R_1 = 0.0378<br>wR_2 = 0.0657
**Largest diff. peak / hole** [e Å^{-3}]	0.35 / -0.32	0.31 / -0.24	0.42 / -0.24	0.59 / -0.37
**CCDC number**	1970927	1970928	1970929	1970937

Table S1 (contd.). Crystallographic data and structure refinement for **(2-K)** and **(2-Ae)** (Ae = Mg, Ca, Sr, Ba).

Compound	[KN(DIPP)(Si^{i}Pr$_3$)]$_n$ (2-K)	Mg[N(DIPP)(Si^{i}Pr$_3$)]$_2$ (2-Mg)	Ca[N(DIPP)(Si^{i}Pr$_3$)]$_2$ (2-Ca)	Sr[N(DIPP)(Si^{i}Pr$_3$)]$_2$ (2-Sr)	Ba[N(DIPP)(Si^{i}Pr$_3$)]$_2$ (2-Ba)
**Empirical formula**	$C_{21}H_{38}KNSi$	$C_{42}H_{76}MgN_2Si_2$	$C_{42}H_{76}CaN_2Si_2$	$C_{42}H_{76}SrN_2Si_2$	$C_{42}H_{76}BaN_2Si_2$
**Formula weight** [g mol^{-1}]	371.71	689.53	705.30	752.84	802.56
**Temperature** [K]	100.0(1)	100.0(1)	100.0(2)	100.0(2)	100.0(1)
**Crystal system**	monoclinic	monoclinic	monoclinic	monoclinic	triclinic
**Space group**	$P2_1/c$	$P2_1$	$P2_1/c$	$P2_1/c$	$P\bar{1}$
$\boldsymbol{a}$ [Å]	10.4673(2)	10.80493(14)	17.8373(3)	18.5610(2)	18.5515(2)
$\boldsymbol{b}$ [Å]	18.2471(3)	23.0314(3)	11.6800(2)	13.73988(14)	27.7418(3)
$\boldsymbol{c}$ [Å]	12.2282(2)	17.5671(2)	20.8572(4)	18.2935(2)	37.3763(5)
$\boldsymbol{\alpha}$ [°]	90	90	90	90°	111.5006(9)
$\boldsymbol{\beta}$ [°]	103.204(2)	99.0901(12)	91.9160(18)	111.6464(15)	100.2002(9)
$\boldsymbol{\gamma}$ [°]	90	90	90	90°	90.6524(9)
**Volume** [Å^3]	2273.81(7)	4316.73(9)	4342.93(15)	4336.30(10)	17553.1(3)
**Z**	4	4	4	4	16
$\boldsymbol{\rho_{Cald}}$ [g cm^{-3}]	1.086	1.061	1.079	1.153	1.215
$\boldsymbol{\mu}$ [mm^{-1}]	2.543	1.086	1.972	2.455	7.758
**F(000)**	816.0	1528.0	1560.0	1632.0	6816.0
**Crystal size** [mm^3]	0.1252 x 0.06 x 0.0424	0.211 x 0.139 x 0.063	0.181 x 0.106 x 0.069	0.283 x 0.245 x 0.202	0.162 x 0.114 x 0.041
**Radiation**	CuKα (λ = 1.54184)	CuKα (λ = 1.54184)	CuKα (λ = 1.54184)	CuKα (λ = 1.54184)	CuKα (λ = 1.54184)
**2Θ range for data collection** [°]	8.678 to 145.194	6.378 to 145.804	8.484 to 145.3	8.226 to 147.358	5.712 to 145.488
**Index ranges**	-12 ≤ h ≤ 12<br>-22 ≤ k ≤ 15<br>-14 ≤ l ≤ 15	-13 ≤ h ≤ 13<br>-28 ≤ k ≤ 28<br>-21 ≤ l ≤ 21	-21 ≤ h ≤ 21<br>-12 ≤ k ≤ 14<br>-21 ≤ l ≤ 25	-23 ≤ h ≤ 23<br>-16 ≤ k ≤ 15<br>-22 ≤ l ≤ 22	-22 ≤ h ≤ 22<br>-29 ≤ k ≤ 33<br>-46 ≤ l ≤ 45
**Reflections collected**	12921	62028	19908	33791	256122
**Independent reflections**	4407	16737	8397	8592	67836
$\mathbf{R_{int}}$	0.03364	0.0506	0.0421	0.0308	0.0600
**Data / restraints / parameters**	4407 / 234 / 295	16737 / 1 / 888	84397 / 6 / 454	8592 / 0 / 444	67836 / 0 / 3545
**Goodness-of-fit on F²**	1.024	1.037	1.028	1.054	1.077
**Final R indexes** [$I \geq 2\sigma(I)$]	R_1 = 0.0372<br>wR_2 = 0.0960	R_1 = 0.0359<br>wR_2 = 0.0916	R_1 = 0.0455<br>wR_2 = 0.1076	R_1 = 0.0246<br>wR_2 = 0.0642	R_1 = 0.0498<br>wR_2 = 0.1207
**Final R indexes** [all data]	R_1 = 0.0440<br>wR_2 = 0.1017	R_1 = 0.0383<br>wR_2 = 0.0933	R_1 = 0.0620<br>wR_2 = 0.1180	R_1 = 0.0269<br>wR_2 = 0.0658	R_1 = 0.0717<br>wR_2 = 0.1316
**Largest diff. peak / hole** [e Å^{-3}]	0.34 / -0.38	0.26 / -0.25	0.38 / -0.30	0.38 / -0.50	1.42 / -1.47
**Flack parameter**	/	0.404(17)	/	/	/
**CCDC number**	1970935	1970930	1970931	1970932	1970933

Table S2. Selected bond lengths (Å) and angles (°) for the complex series $Ae[N(TRIP)_2]_2$ (**1**-Ae) and $Ae[N(TRIP)(DIPP)]_2$ (**2**-Ae); average values shown between <>.

Complexes **1**-Ae				
Ae	Mg	Ca[28]	Sr	Ba
Ae-N	1.997(1)	2.329(2)-2.337(2)<br><2.333>	2.492(2)-2.496(2)<br><2.494>	2.683(1)
Si-N	1.724(1)-1.733(1)<br><1.729>	1.702(2)-1.711(3)<br><1.707>	1.694(1)-1.699(1)<br><1.697>	1.687(1)-1.689(1)<br><1.688>
N-Ae-N’	179.7(1)	172.6(1)	171.9(1)	177.5(1)
Si-N-Si’	132.4(1)	136.7(2)-138.2(2)<br><137.5>	139.0(1)-139.5(1)<br><139.3>	141.3(1)
Ae···HC[a]	2.34-2.83	2.42-2.96	2.52-3.12	2.83-3.10
Complexes **2**-Ae				
Ae	Mg	Ca	Sr	Ba
Ae-N	1.934(2)-1.946(2)<br><1.938>	2.253(2)-2.283(2)<br><2.268>	2.423(1)-2.423(1)<br><2.423>	2.551(4)-2.653(4)<br><2.607>
Si-N	1.724(2)-1.733(2)<br><1.728>	1.703(2)-1.713(2)<br><1.708>	1.702(1)-1.697(1)<br><1.700>	1.681(4)-1.704(4)<br><1.693>
N-Ae-N’	166.8(1)-169.5(1)<br><168.1>	145.0(1)	143.8(1)	148.6(1)-162.0(1)<br><155.2>
Si-N-C	124.0(2)-129.0(2)<br><126.3>	126.2(1)-136.4(1)<br><131.3>	135.0(1)-135.8(1)<br><135.4>	130.8(3)-1420.(3)<br><137.6>
Ae-N-Si	112.9(1)-116.2(1)<br><114.2>	128.9(1)-148.6(1)<br><138.7>	132.6(1)-135.6(1)<br><134.1>	127.5(2)-139.9(2)<br><131.8>
Ae-N-C	116.1(2)-121.9(2)<br><118.7>	85.2(1)-92.9(1)<br><89.1>	89.3(1)-91.7(1)<br><90.5>	87.9(3)-91.5(3)<br><89.3>
Ae···C_{arom}[b]	-	2.578(2)-2.914(2)	2.781(1)-3.100(1)	2.881(5)-3.273(4)
Ae···HC[a]	2.34-2.84	2.52-2.87	2.70-2.92	2.69-3.20

[a] Interactions between the Ae metal and aromatic ring carbon atoms

[b] Anagostic Ae···HC interactions are contacts that are shorter than the sum of the Ae and H van der Waals radii: Mg···H < 2.93 Å, Ca···H < 2.99 Å, Sr···H < 3.12 Å, Ba···H < 3.34 Å.

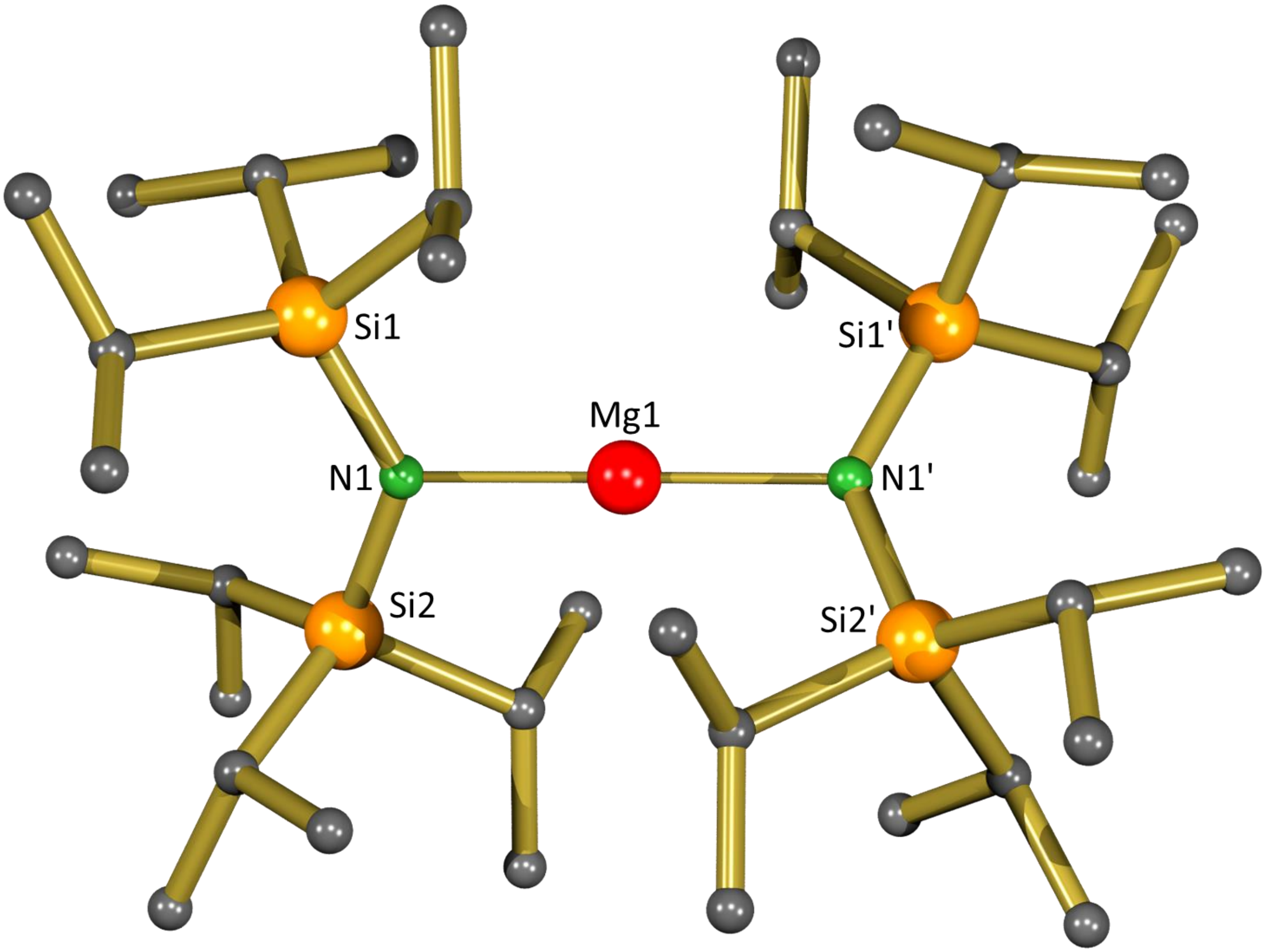

Figure S49. Solid state structure of Mg[N(Si^{i}Pr$_3$)$_2$]$_2$ (**1-Mg**) as determined by single-crystal X-ray diffraction. Hydrogen atoms are omitted for clarity. Selected interatomic distances [Å] and angles [°]: Mg1-N1 1.9971(9); Si1-N1 1.7240(10); Si2-N1 1.7328(9); N1-Mg-N1' 179.74(6); Si1-N1-Si2 132.44(6).

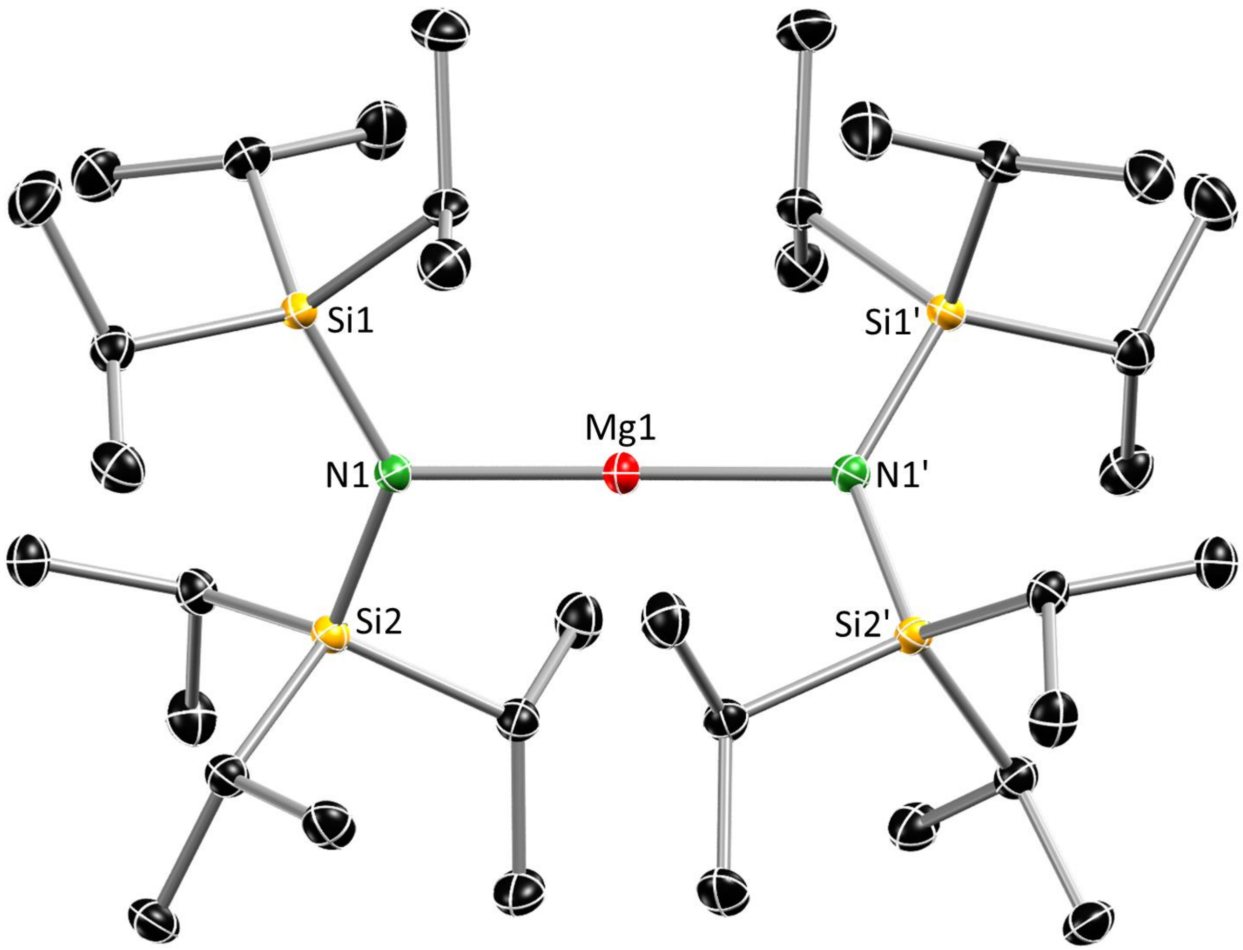

Figure S50. ORTEP representation of $Mg[N(Si^iPr_3)_2]_2$ (**1-Mg**) with atomic displacement ellipsoids set at 50% probability. Hydrogen atoms are omitted for clarity.

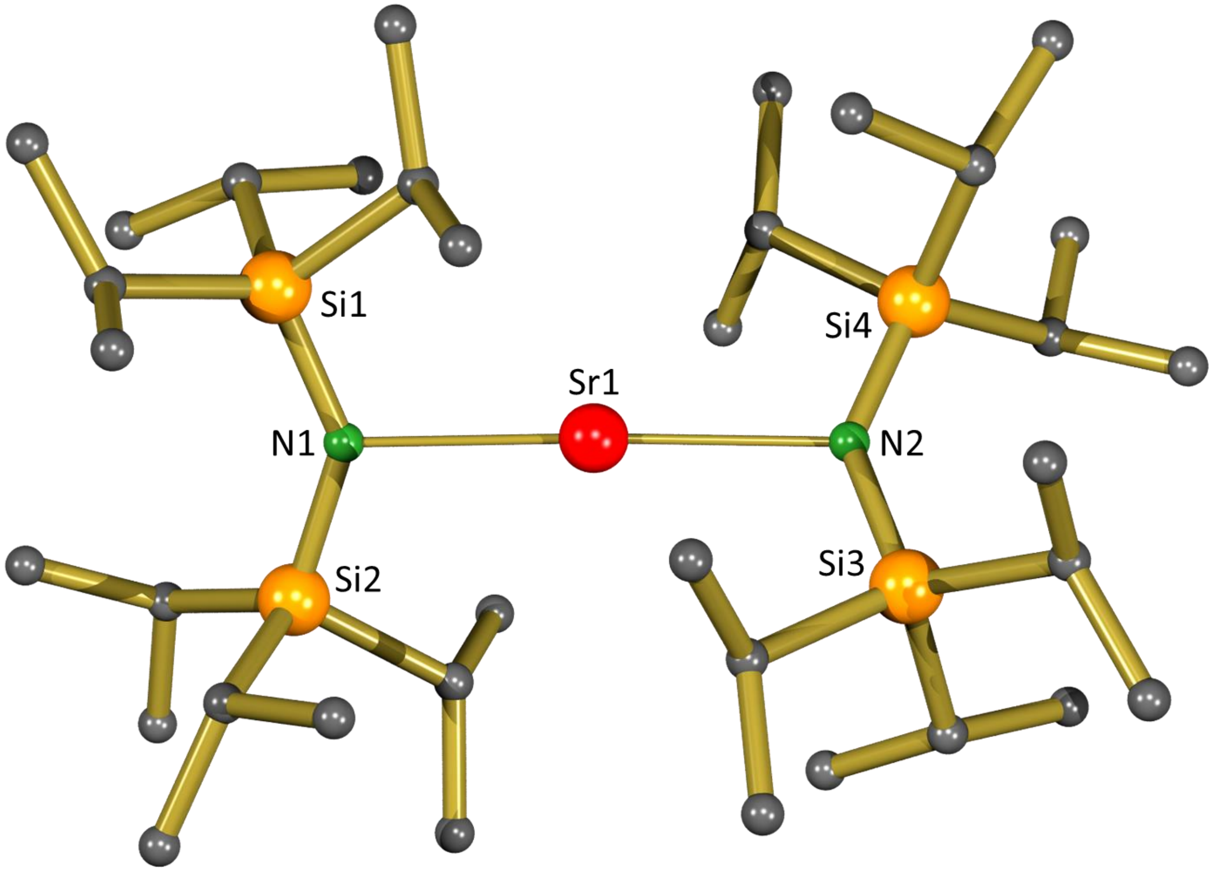

Figure S51. Solid state structure of Sr[N(Si^{i}Pr$_3$)$_2$]$_2$ (**1-Sr**) as determined by single-crystal X-ray diffraction. The disordered strontium atom is excluded, and hydrogen atoms are omitted for clarity. Selected interatomic distances [Å] and angles [°]: Sr1-N1 2.4915(17); Sr1-N2 2.4959(17); Si1-N1 1.6938(12); Si2-N1 1.6992(12); Si3-N2 1.6958(12); Si4-N2 1.6978(12); N1-Sr1-N2 171.94(13); Si1-N1-Si2 139.03(8); Si3-N2-Si4 139.53(8).

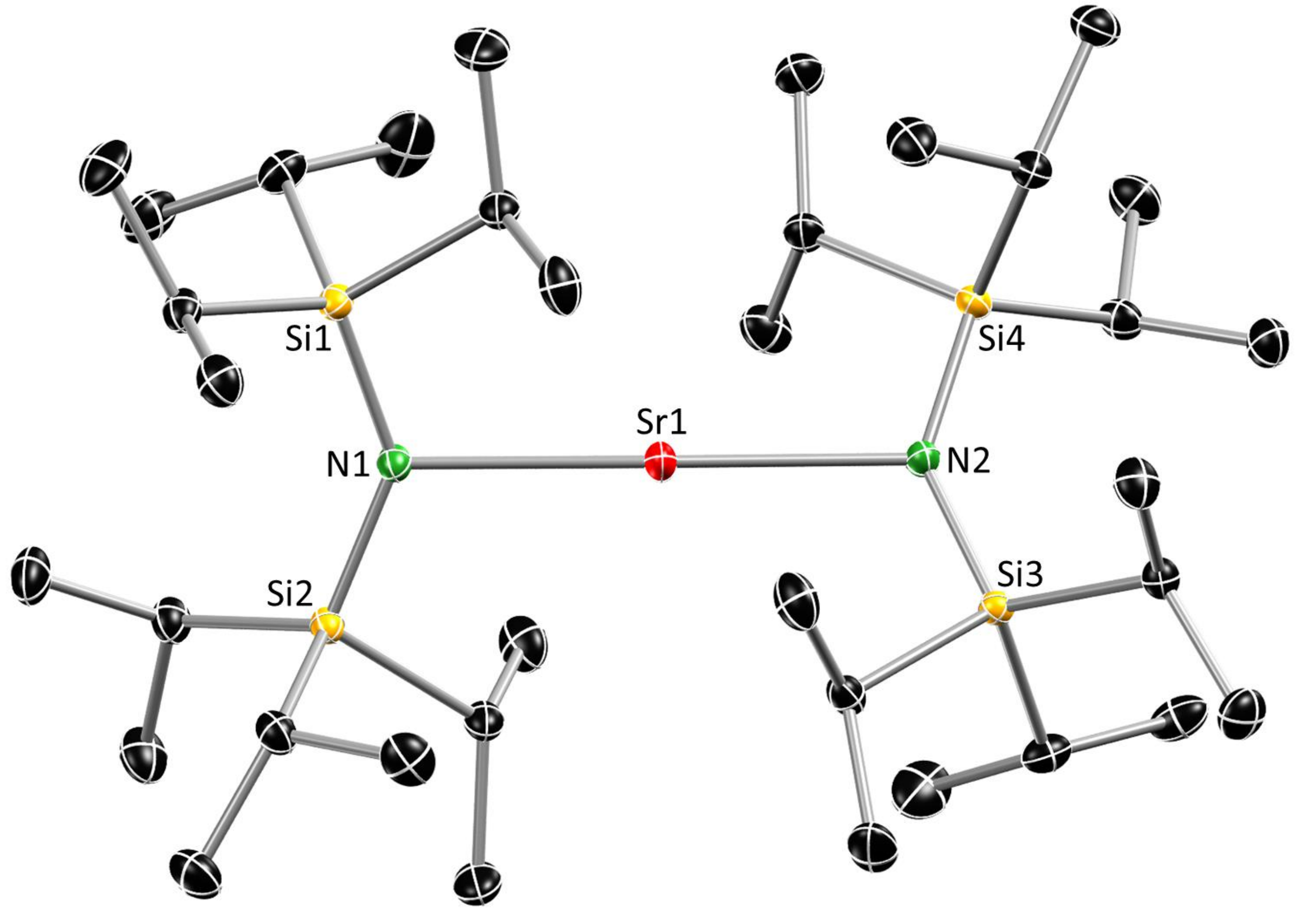

Figure S52. ORTEP representation of $Sr[N(Si^{i}Pr_3)_2]_2$ (**1-Sr**) with atomic displacement ellipsoids set at 50% probability. The disordered strontium atom is excluded, and hydrogen atoms are omitted for clarity.

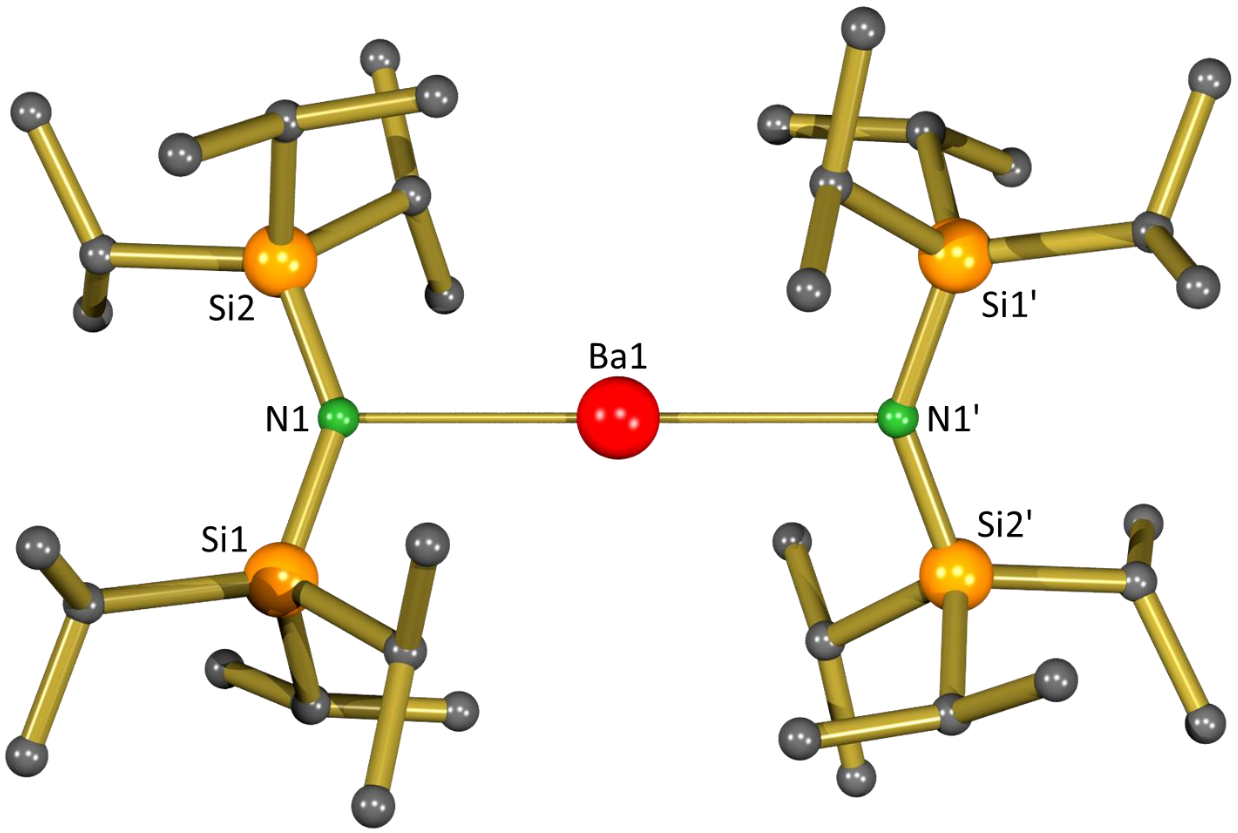

Figure S53. Solid state structure of Ba[N(Si^{i}Pr$_3$)$_2$]$_2$ (**1-Ba**) as determined by single-crystal X-ray diffraction. Hydrogen atoms are omitted for clarity. Selected interatomic distances [Å] and angles [°]: Ba1-N1 2.6831(11); Si1-N1 1.6888(11); Si2-N1 1.6870(12); N1-Ba1-N1' 177.45(5); Si1-N1-Si2 141.27(7).

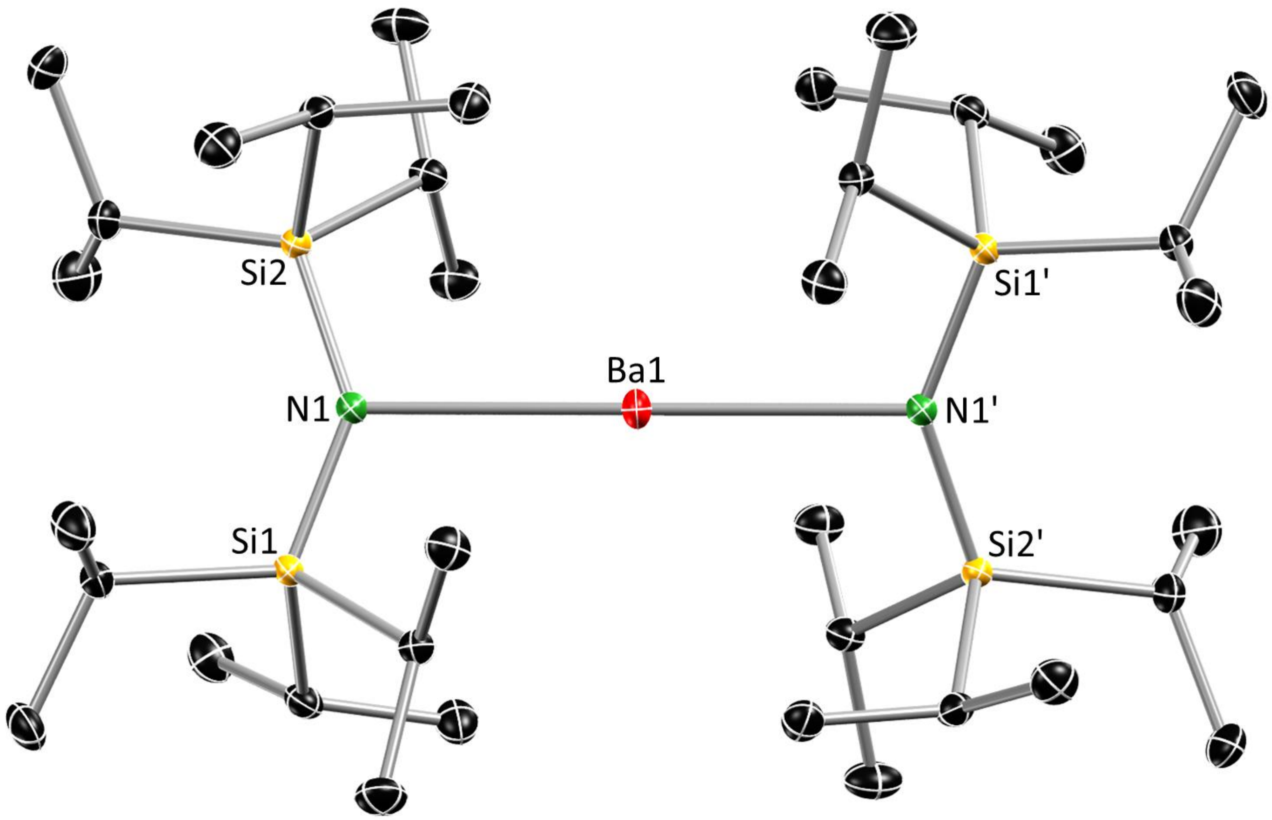

Figure S54. ORTEP representation of Ba[N(Si^{i}Pr$_3$)$_2$]$_2$ (**1-Ba**) with atomic displacement ellipsoids set at 50% probability. Hydrogen atoms are omitted for clarity.

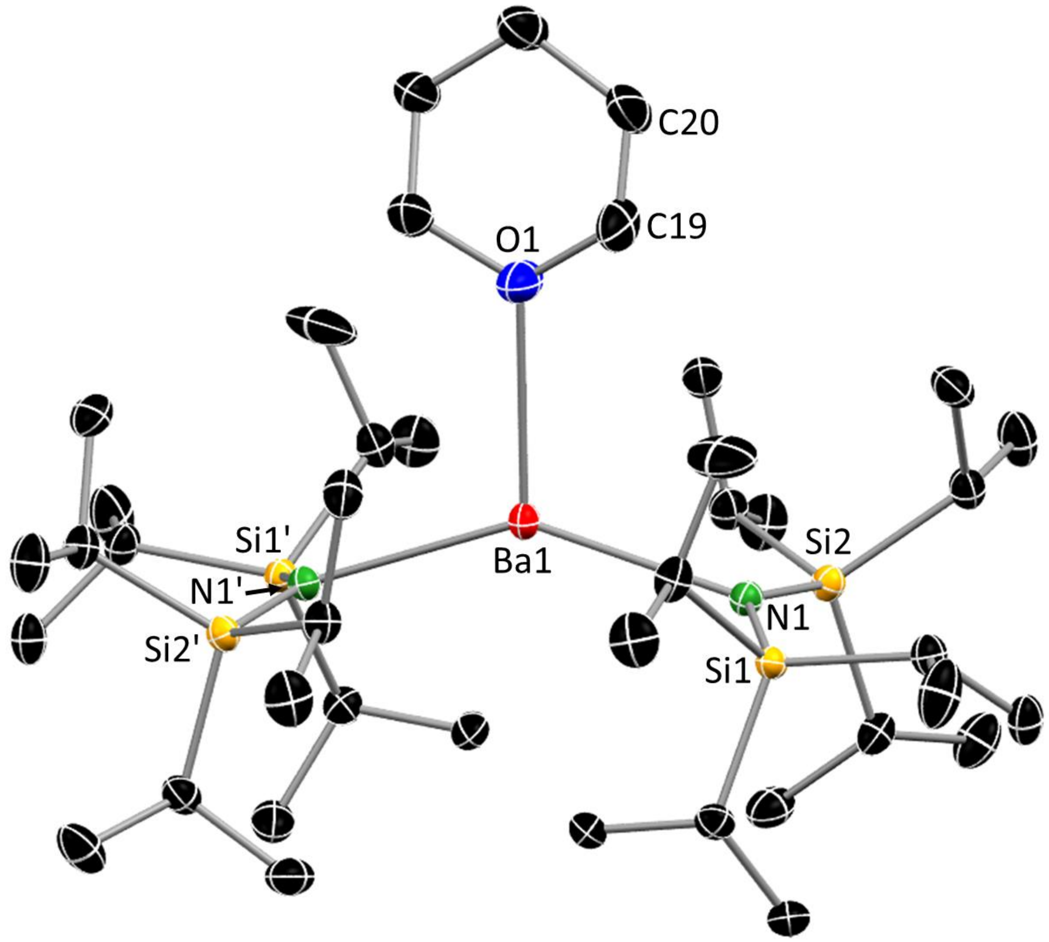

Figure S55. ORTEP representation of {Ba[N(Si^{i}Pr$_3$)$_2$]$_2$(3,4-dihydro-2*H*-pyran)} with atomic displacement ellipsoids set at 50% propability. Hydrogen atoms are omitted for clarity. Selected interatomic distances [Å] and angles [°]: Ba1-N1 2.6781(13); Ba1-O1 2.8011(17); Si1-N1 1.6982(14); Si2-N1 1.6961(14); C19-C20 1.334(9); N1-Ba1-N1' 144.41(5); N1-Ba1-O1 107.80(3).

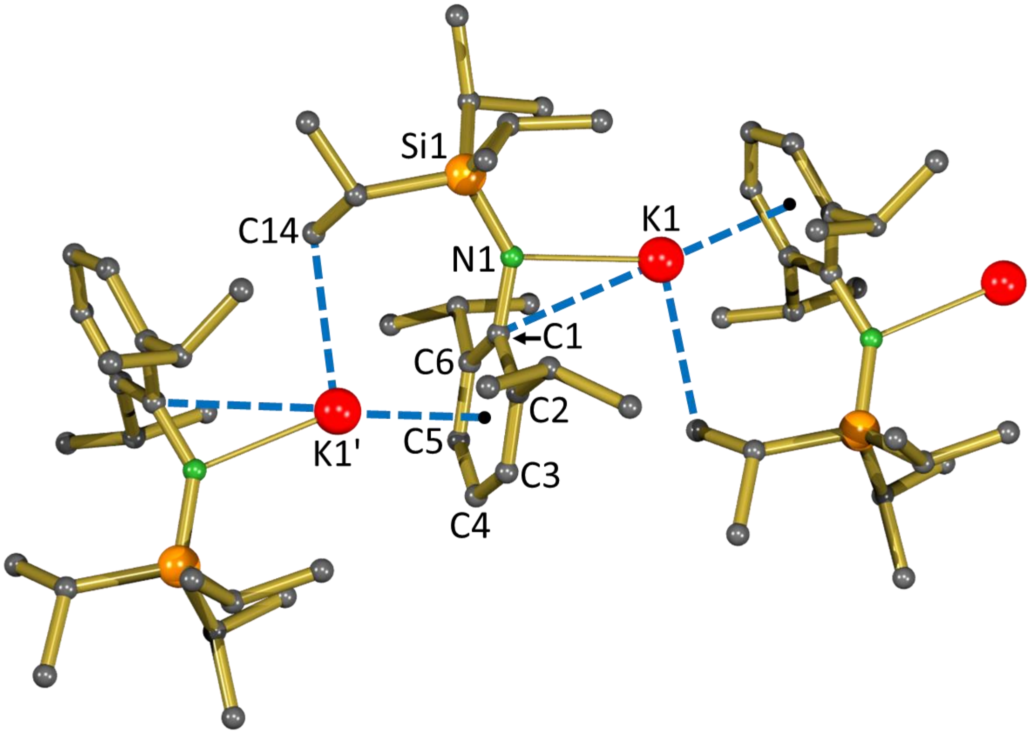

Figure S56. Infinite zig-zag chain structure of [KN(DIPP)(Si^{i}Pr$_3$)]$_n$ (**2-K**) as determined by single-crystal X-ray diffraction. Hydrogen atoms are omitted for clarity. Selected interatomic distances [Å] and angles [°]: K1-N1 2.6639(16); K1'···C1 3.2087(13); K1'···C2 3.0722(18); K1'···C3 3.068(2); K1'···C4 3.173(2); K1'···C5 3.2783(19); K1'···C6 3.3200(18); K1···C1 3.121(2); K1'···C14 3.286(3); Si1-N1 1.6656(16); K1-N1-Si1 122.88(8); K1-N1-C1 96.31(10).

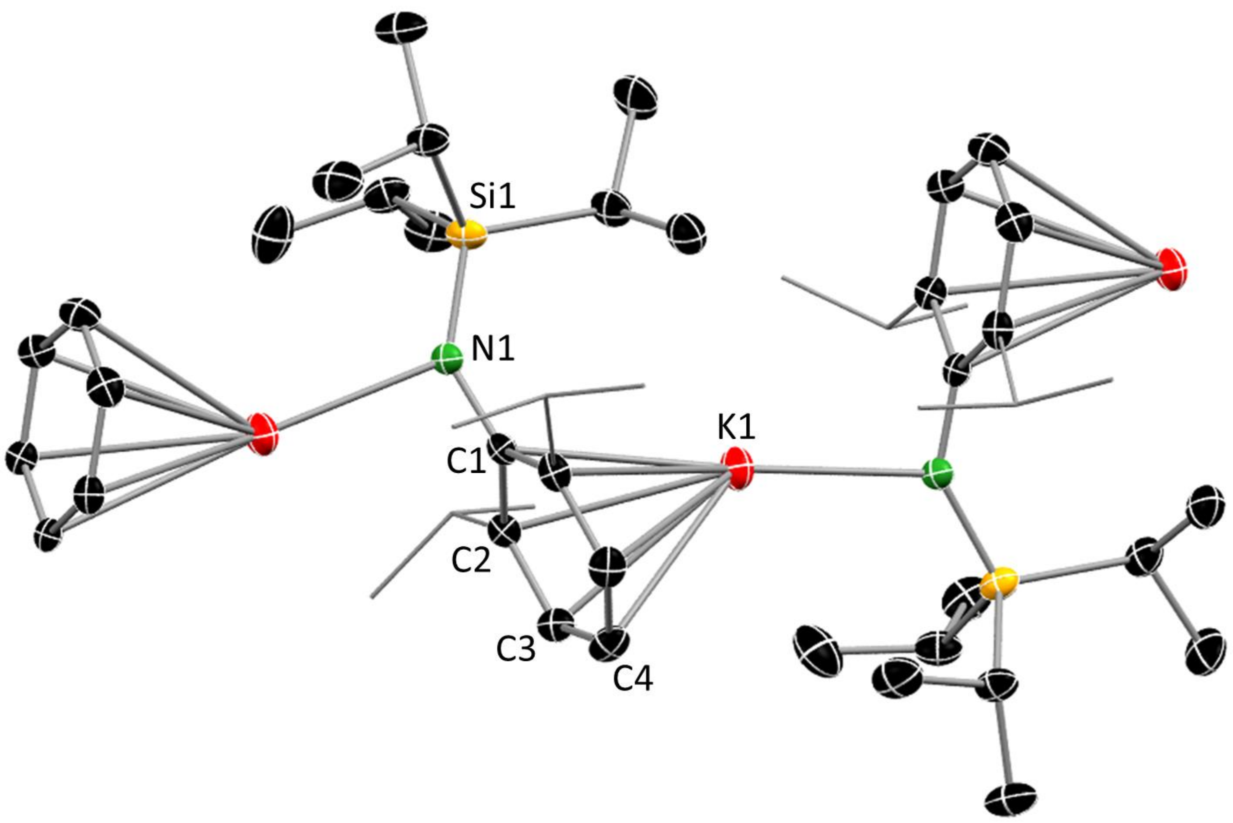

Figure S57. Infinite zig-zag chain structure [KN(DIPP)(Si^{i}Pr$_3$)]$_n$ (**2-K**). Thermal ellipsoids are displayed at 50% probability. Hydrogen atoms are omitted, and selected carbon atoms are shown in wireframe format for clarity.

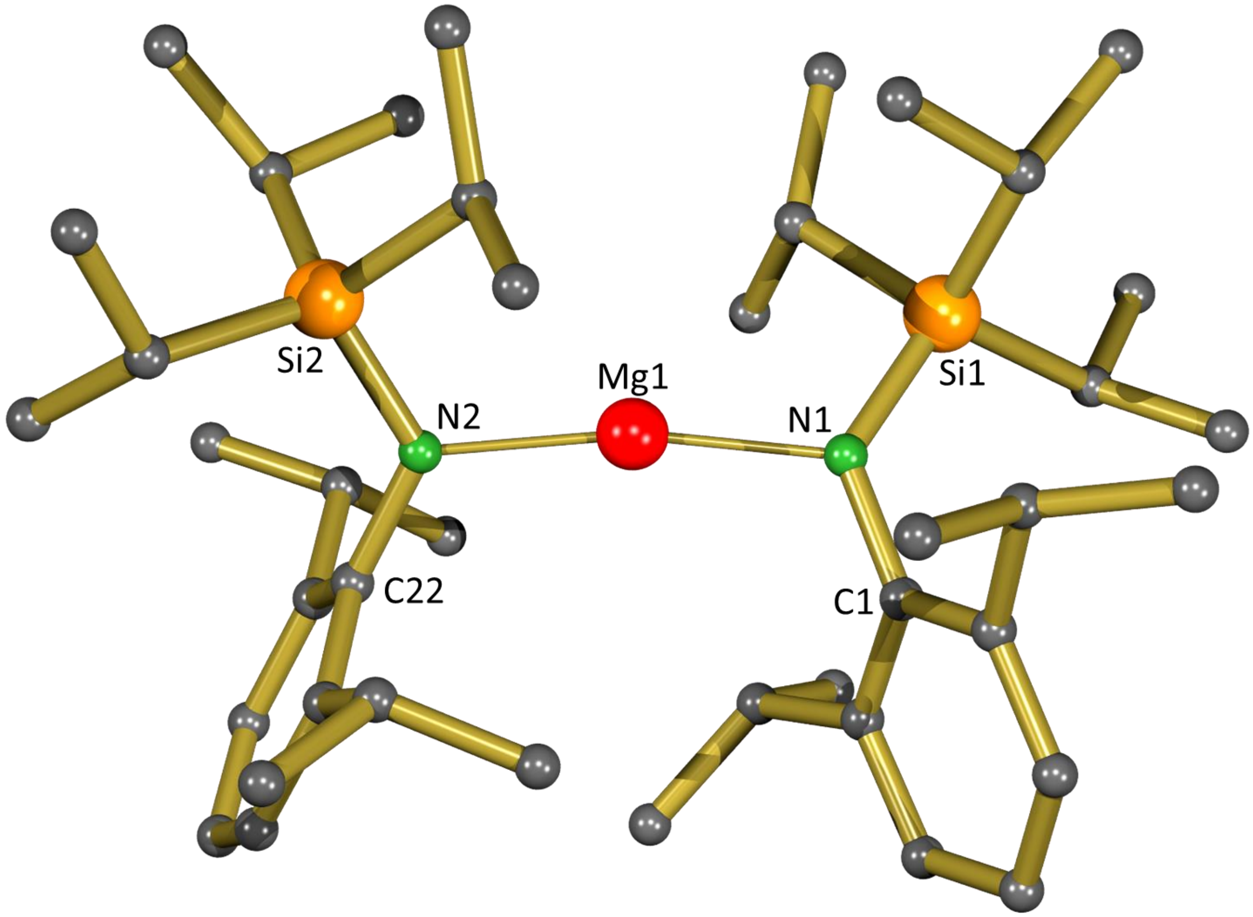

Figure S58. Solid state structure of Mg[N(DIPP)(Si^{i}Pr$_3$)]$_2$ (**2-Mg**) as determined by single-crystal X-ray diffraction. Hydrogen atoms are omitted for clarity. Selected interatomic distances [Å] and angles [°]: Mg1-N1 1.946(2); Mg1-N2 1.937(2); Si1-N1 1.729(2); Si2-N2 1.727(2); N1-Mg1-N2 169.49(11); Mg1-N1-Si1 116.16(12); Mg1-N2-Si2 113.67(12); Mg1-N1-C1 116.12(17); Mg1-N2-C22 121.90(17).

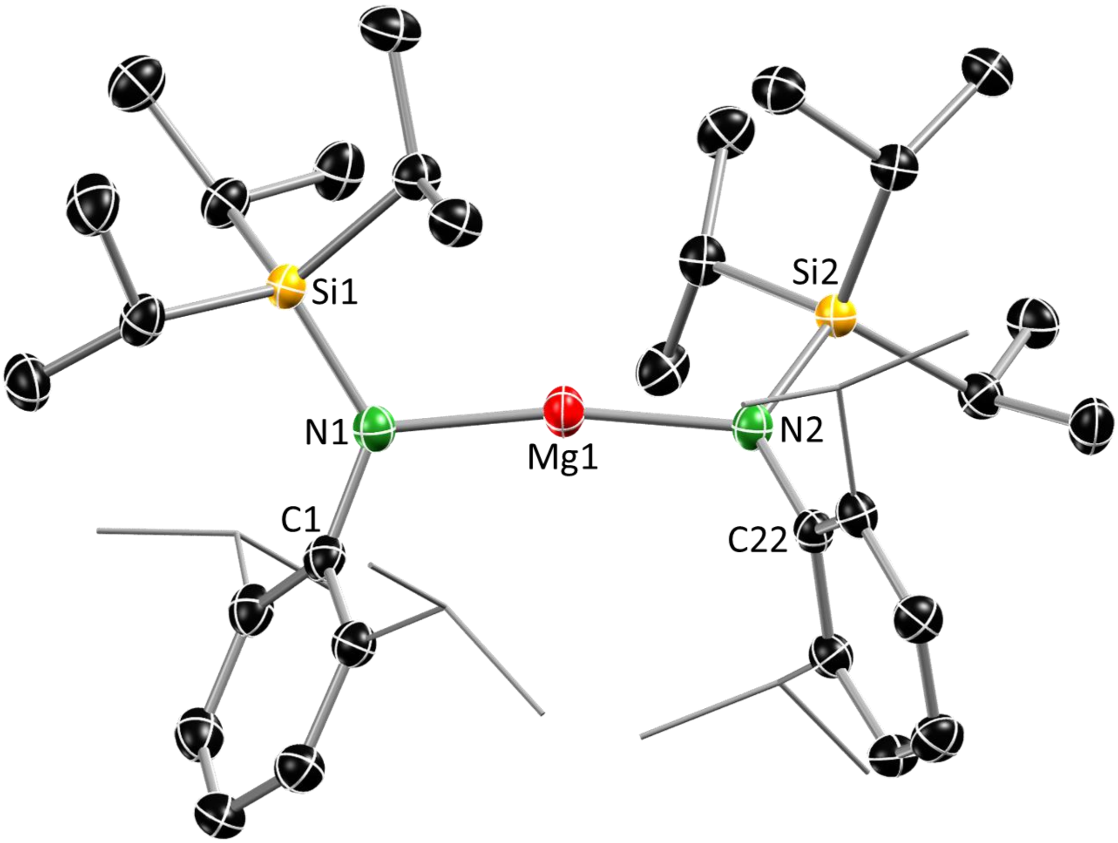

Figure S59. ORTEP representation of Mg[N(DIPP)(Si^{i}Pr$_3$)]$_2$ (**2-Mg**) with atomic displacement ellipsoids set at 50% probability. Hydrogen atoms are omitted, and selected carbon atoms shown in wireframe format for clarity.

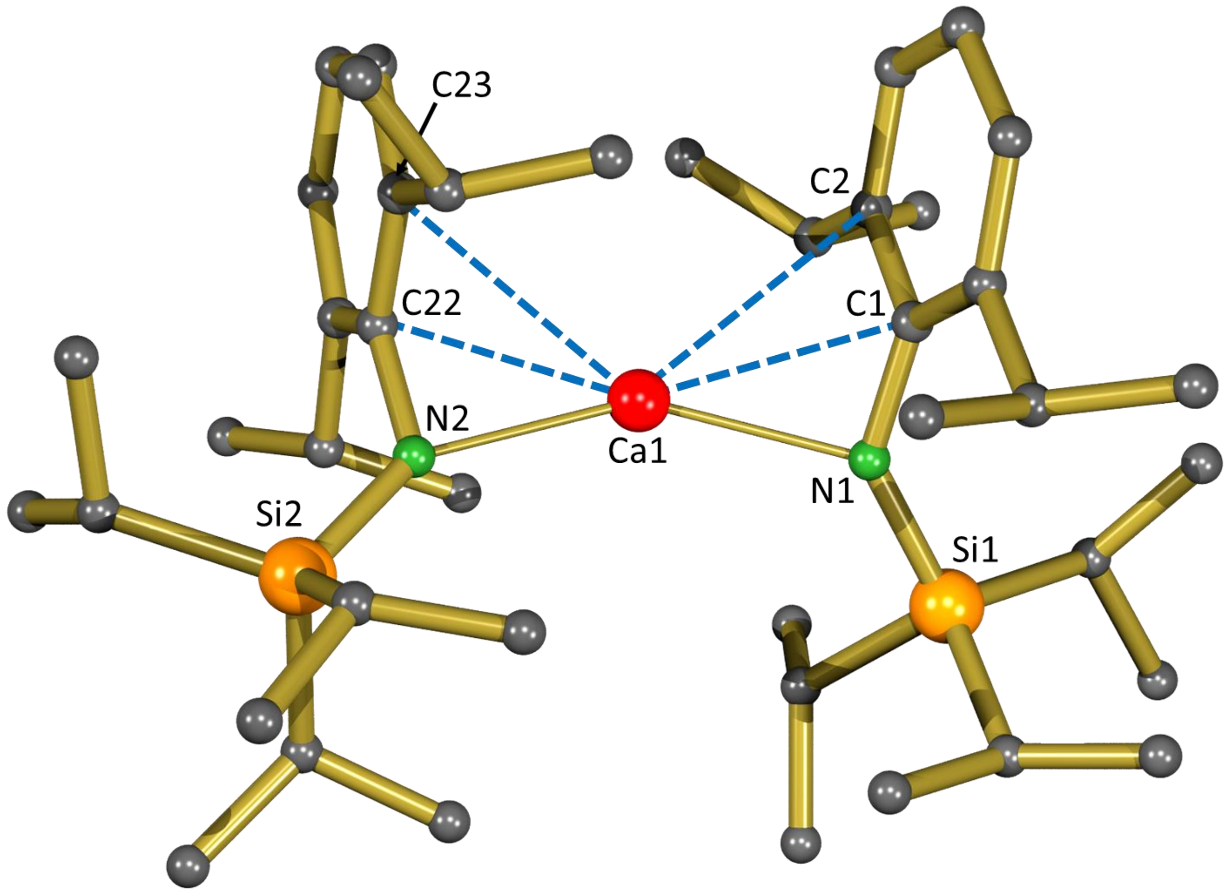

Figure S60. Solid state structure of Ca[N(DIPP)(Si^{i}Pr$_3$)]$_2$ (**2-Ca**) as determined by single-crystal X-ray diffraction. Hydrogen atoms are omitted for clarity. Selected interatomic distances [Å] and angles [°]: Ca1-N1 2.2534(19); Ca1-N2 2.283(2); Si1-N1 1.7034(17); Si2-N2 1.7125(16); Ca1···C1 2.709(2); Ca1···C2 2.914(2); Ca1···C22 2.578(2); Ca1···C23 3.082(3); N1-Ca1-N2 145.02(9); Ca1-N1-Si1 128.85(10); Ca1-N2-Si2 148.59(9); Ca1-N1-C1 92.94(12); Ca1-N2-C22 85.21(11).

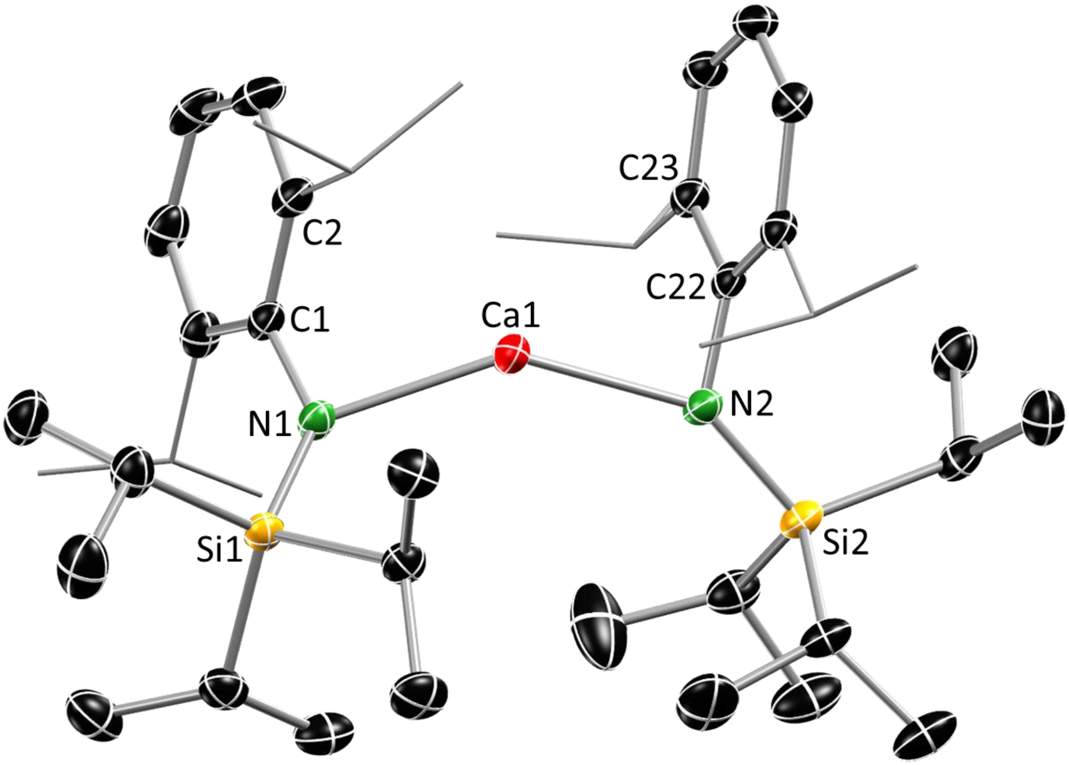

Figure S61. ORTEP representation of Ca[N(DIPP)(Si^{i}Pr$_3$)]$_2$ (**2-Ca**) with atomic displacement ellipsoids set at 50% probability. Hydrogen atoms are omitted, and selected carbon atoms shown in wireframe format for clarity.

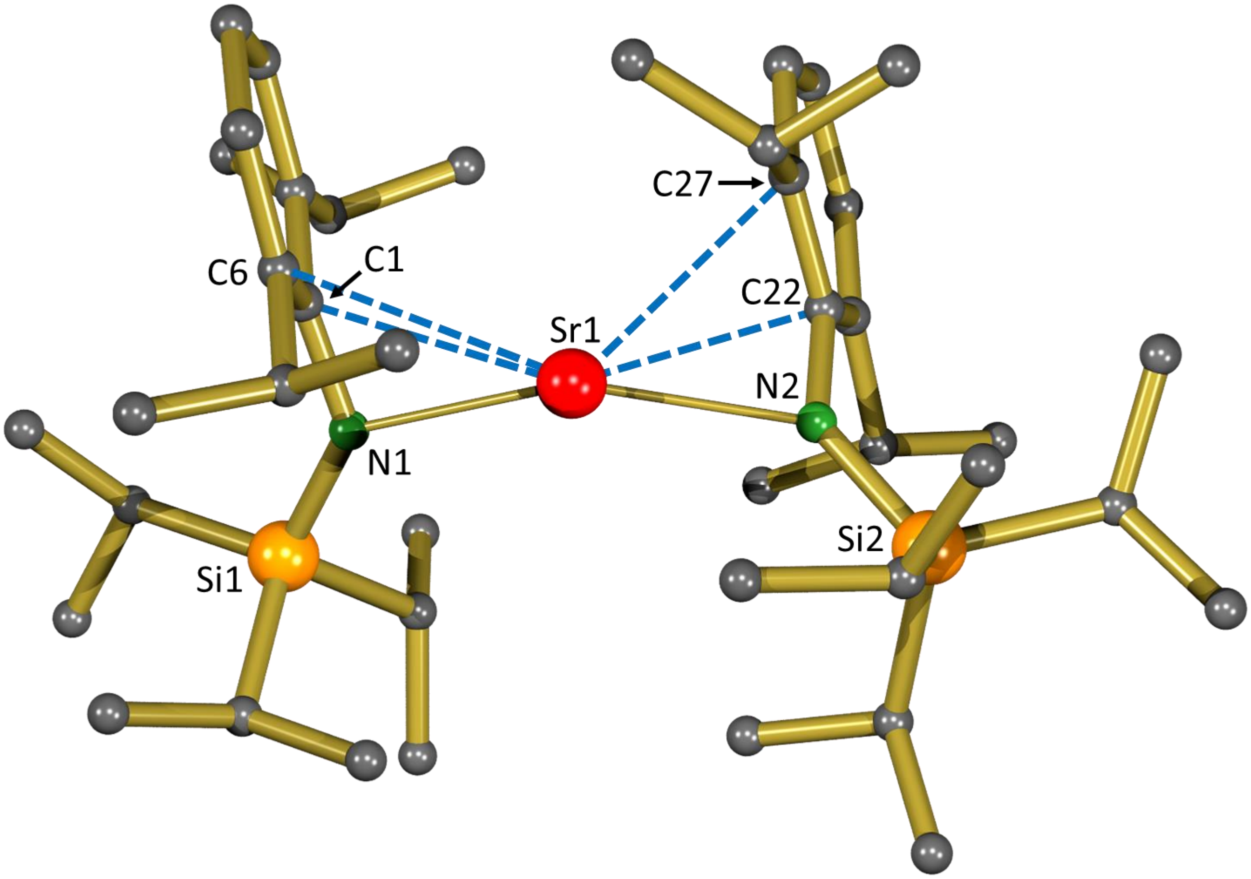

Figure S62. Solid state structure of Sr[N(DIPP)(Si^{i}Pr$_3$)]$_2$ (**2-Sr**) as determined by single-crystal X-ray diffraction. Hydrogen atoms are omitted for clarity. Selected interatomic distances [Å] and angles [°]: Sr1-N1 2.4228(11); Sr1-N2 2.4228(11); Si1-N1 1.7024(11); Si2-N2 1.6965(11); Sr1···C1 2.7810(12); Sr1···C6 3.0997(13); Sr1···C22 2.8259(13); Sr1···C27 2.9940(13); N1-Sr1-N2 143.82(4); Sr1-N1-Si1 135.59(6); Sr1-N2-Si2 132.57(6); Sr1-N1-C1 89.32(7); Sr1-N2-C22 91.66(7).

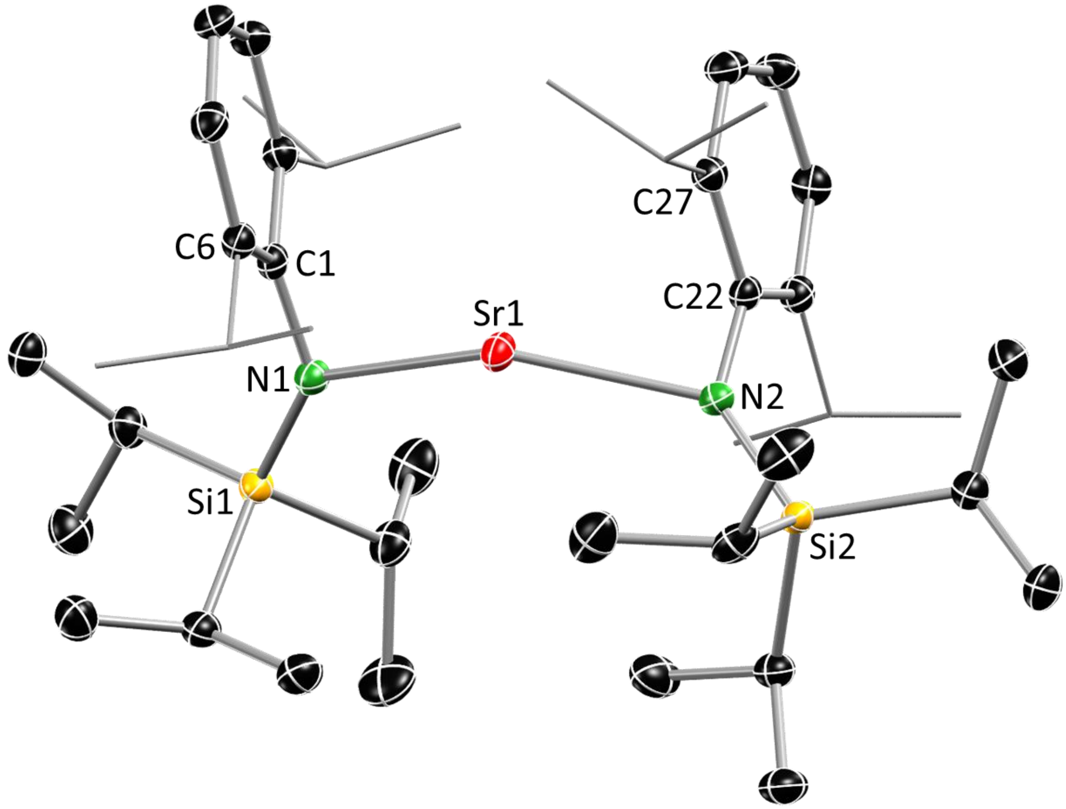

Figure S63. ORTEP representation of Sr[N(DIPP)(Si^{i}Pr$_3$)]$_2$ (**2-Sr**) with atomic displacement ellipsoids set at 50% probability. Hydrogen atoms are omitted, and selected carbon atoms shown in wireframe format for clarity.

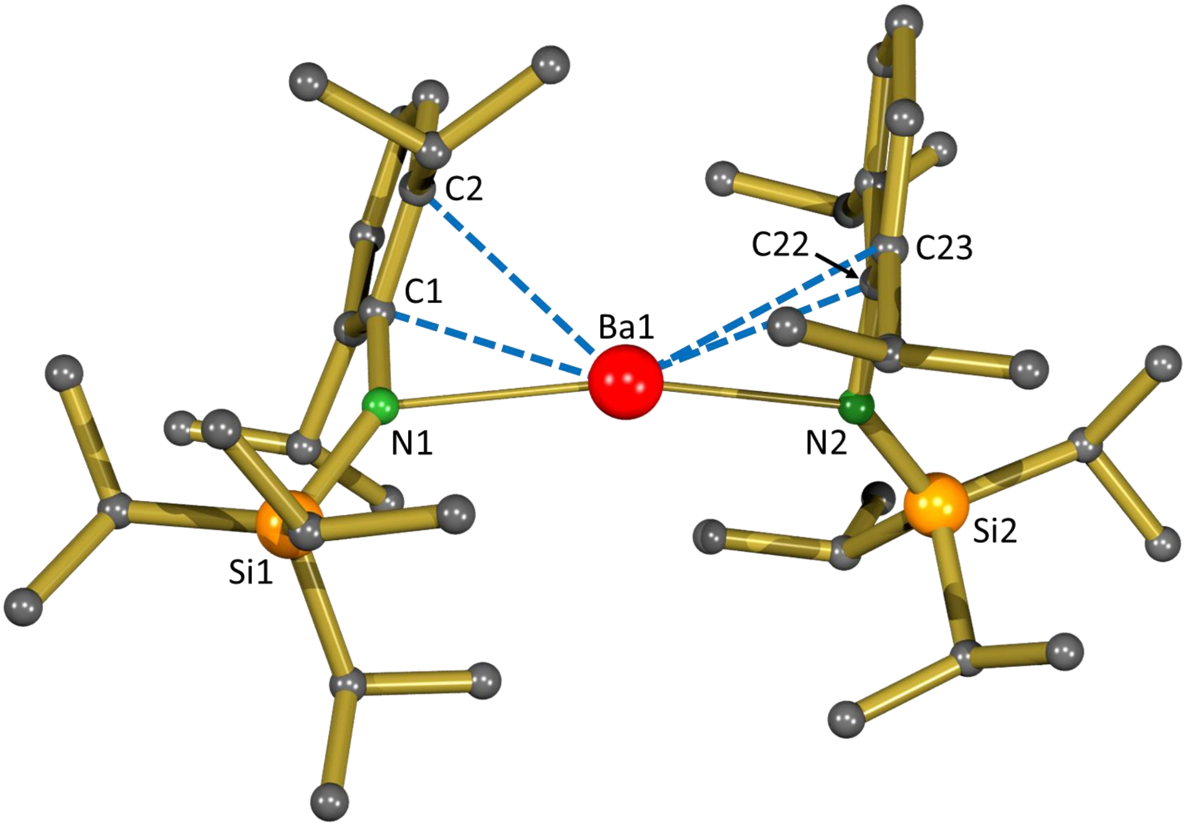

Figure S64. Solid state structure of Ba[N(DIPP)(Si^{i}Pr$_3$)]$_2$ (**2-Ba**) as determined by single-crystal X-ray diffraction. Hydrogen atoms are omitted for clarity. Selected interatomic distances [Å] and angles [°]: Ba1-N1 2.613(4); Ba1-N2 2.604(4); Si1-N1 1.684(4); Si2-N2 1.704(4); Ba1···C1 2.938(4); Ba1···C2 3.095(4); Ba1···C22 2.916(4); Ba1···C23 3.236(4); N1-Ba1-N2 159.51(11); Ba1-N1-Si1 127.49(18); Ba1-N2-Si2 137.52(19); Ba1-N1-C1 89.5(3); Ba1-N2-C22 88.3(3).

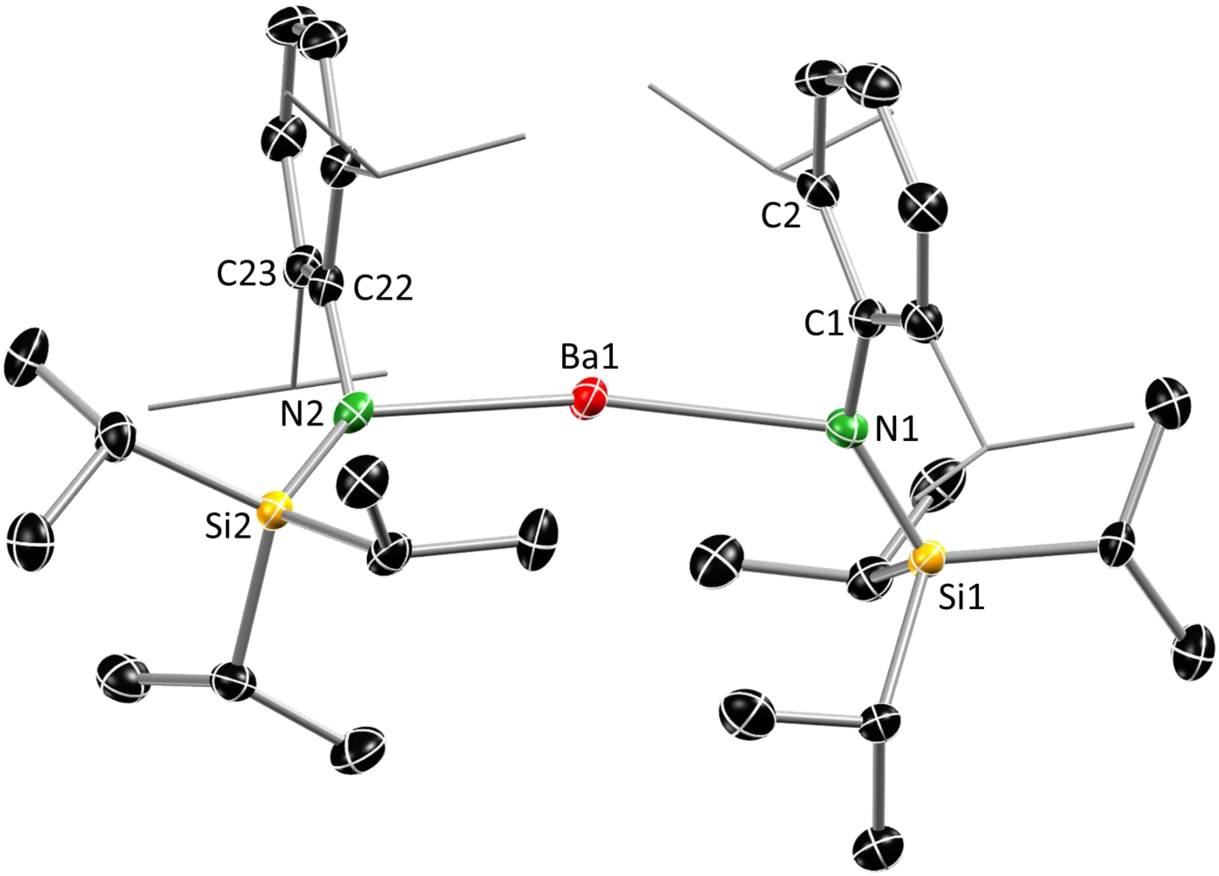

Figure S65. ORTEP representation of Ba[N(DIPP)(Si^{i}Pr$_3$)]$_2$ (**2-Ba**) with atomic displacement ellipsoids set at 50% probability. Hydrogen atoms are omitted, and selected carbon atoms shown in wireframe format for clarity.

4 Preparation of Pre-Catalysts

Pre-catalysts $Ae[N(SiMe_3)_2]_2$ (Ae = Ca, Sr, Ba),[S4] $Sr[CH(SiMe_3)_2]_2(THF)_2$[S5] and $(DMAT)_2Sr(THF)_3$[S6] were prepared according to literature procedures.

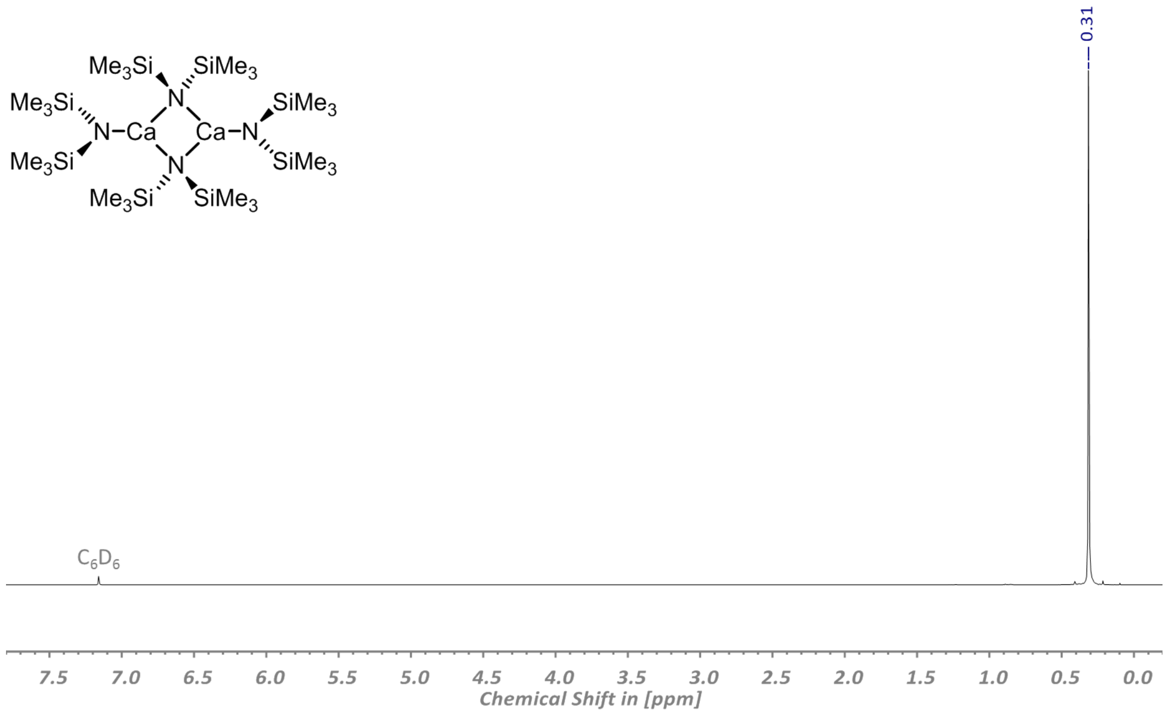

Figure S66. ^{1}H NMR spectrum (600 MHz, C_6D_6, 25 °C) of $Ca[N(SiMe_3)_2]_2$.

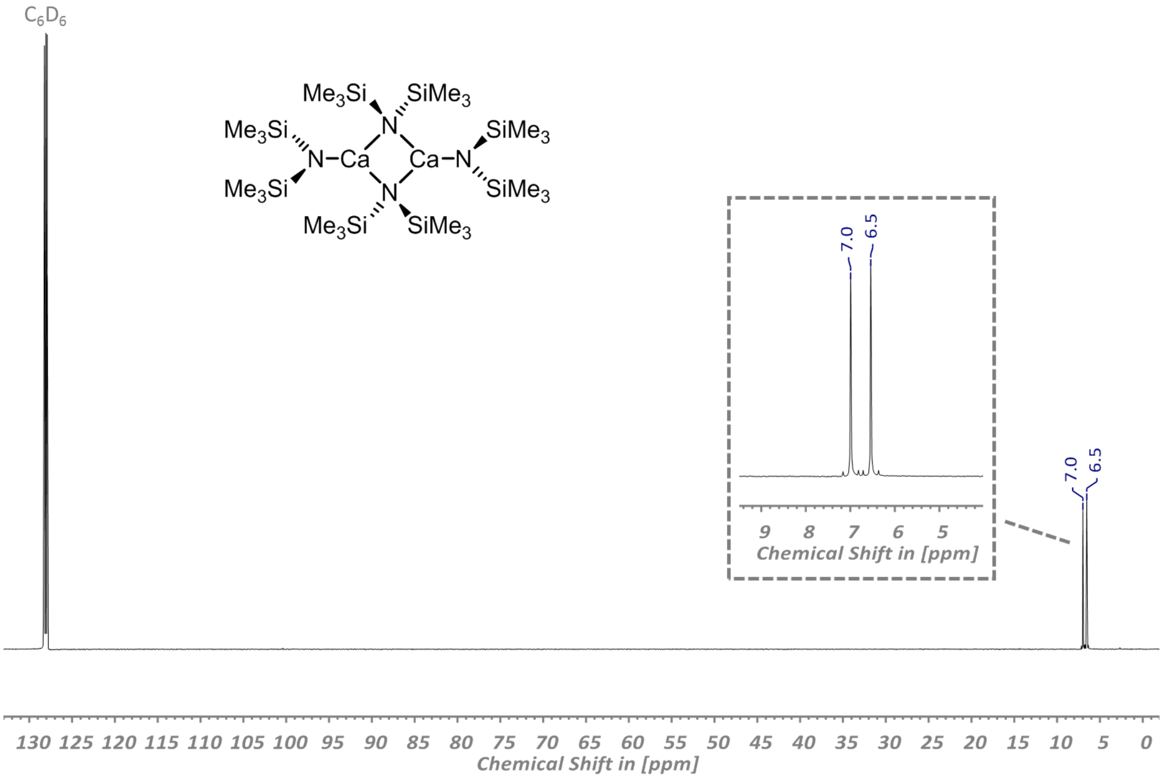

Figure S67. $^{13}C\{^{1}H\}$ NMR (151 MHz, C_6D_6, 25 °C) of $Ca[N(SiMe_3)_2]_2$.

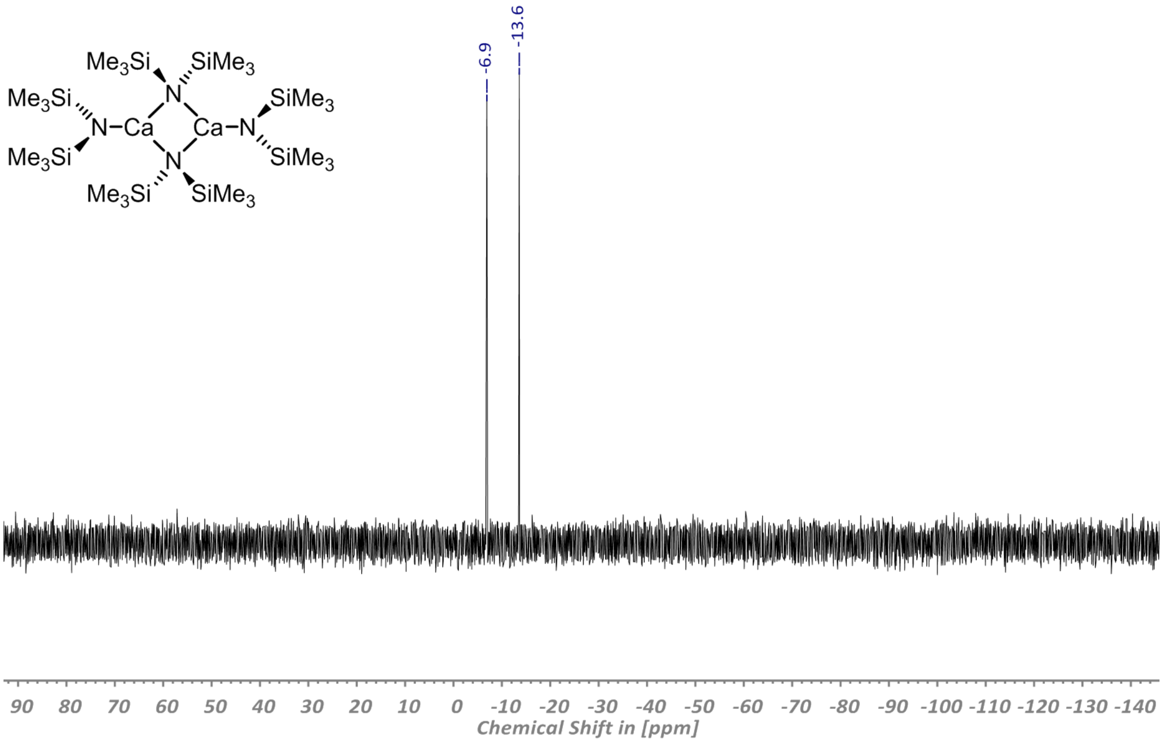

Figure S68. $^{29}Si\{^{1}H\}$ NMR spectrum (119 MHz, C_6D_6, 25 °C) of $Ca[N(SiMe_3)_2]_2$.

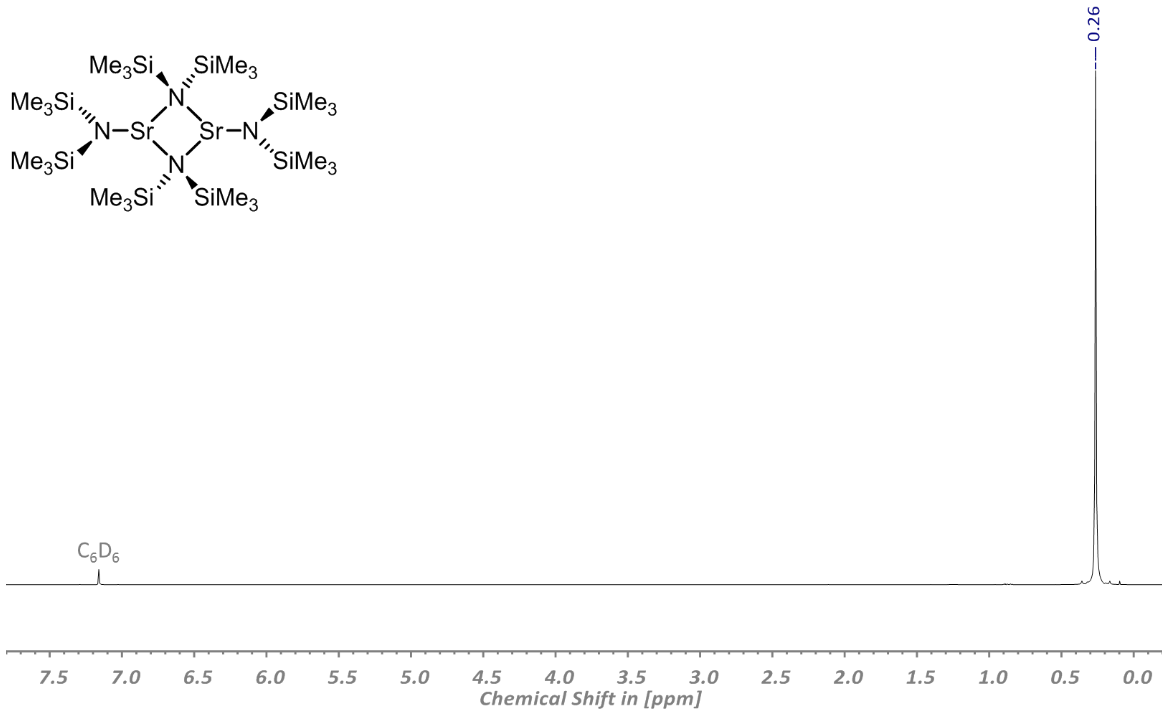

Figure S69. ^{1}H NMR spectrum (600 MHz, C_6D_6, 25 °C) of $Sr[N(SiMe_3)_2]_2$.

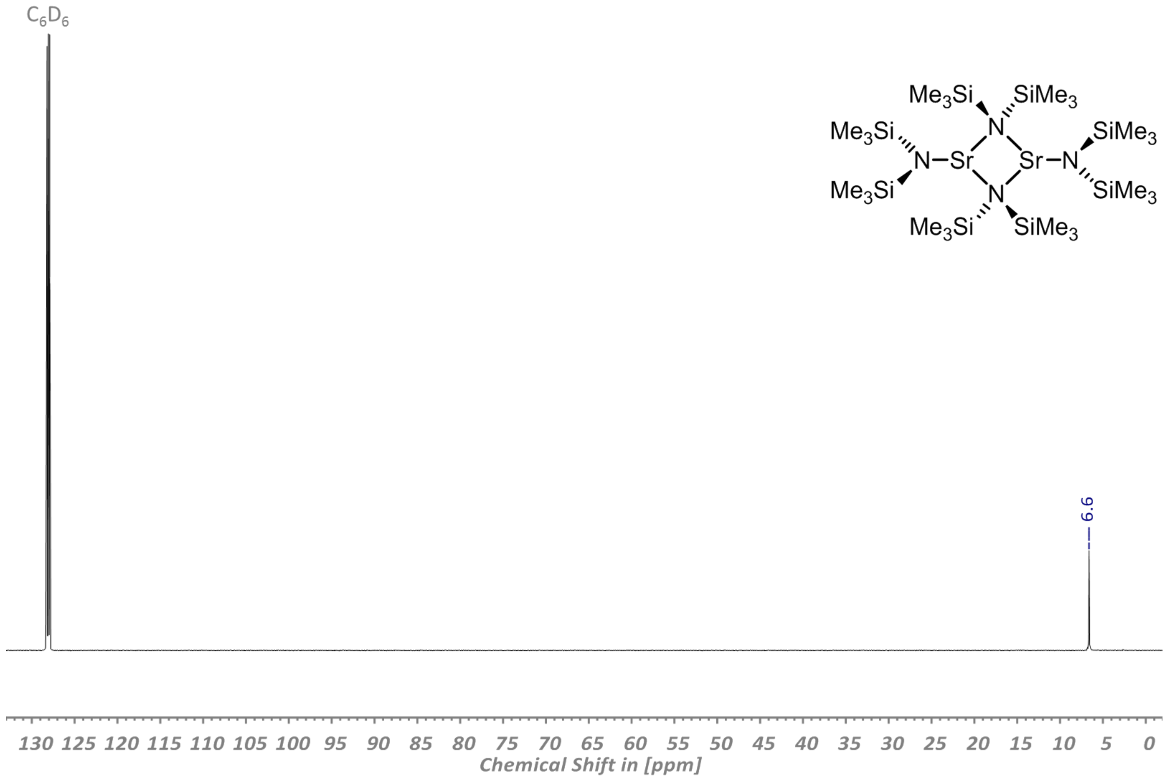

Figure S70. $^{13}C\{^{1}H\}$ NMR (151 MHz, C_6D_6, 25 °C) of $Sr[N(SiMe_3)_2]_2$.

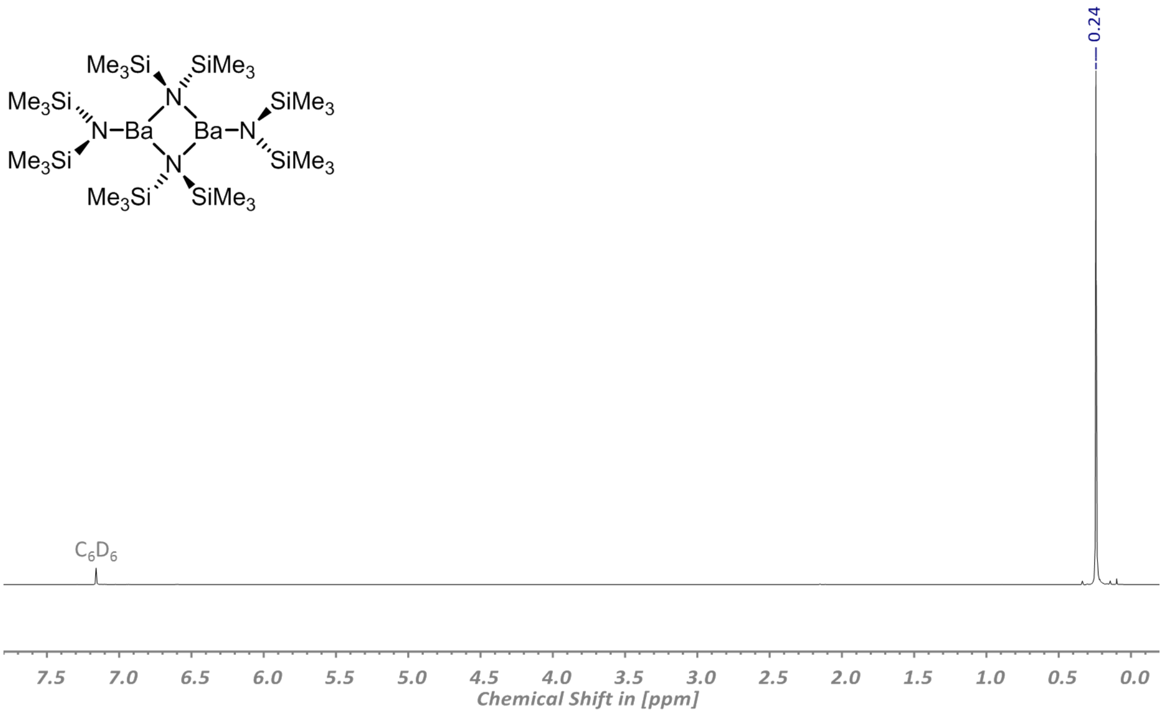

Figure S71. ^{1}H NMR spectrum (600 MHz, C_6D_6, 25 °C) of $Ba[N(SiMe_3)_2]_2$.

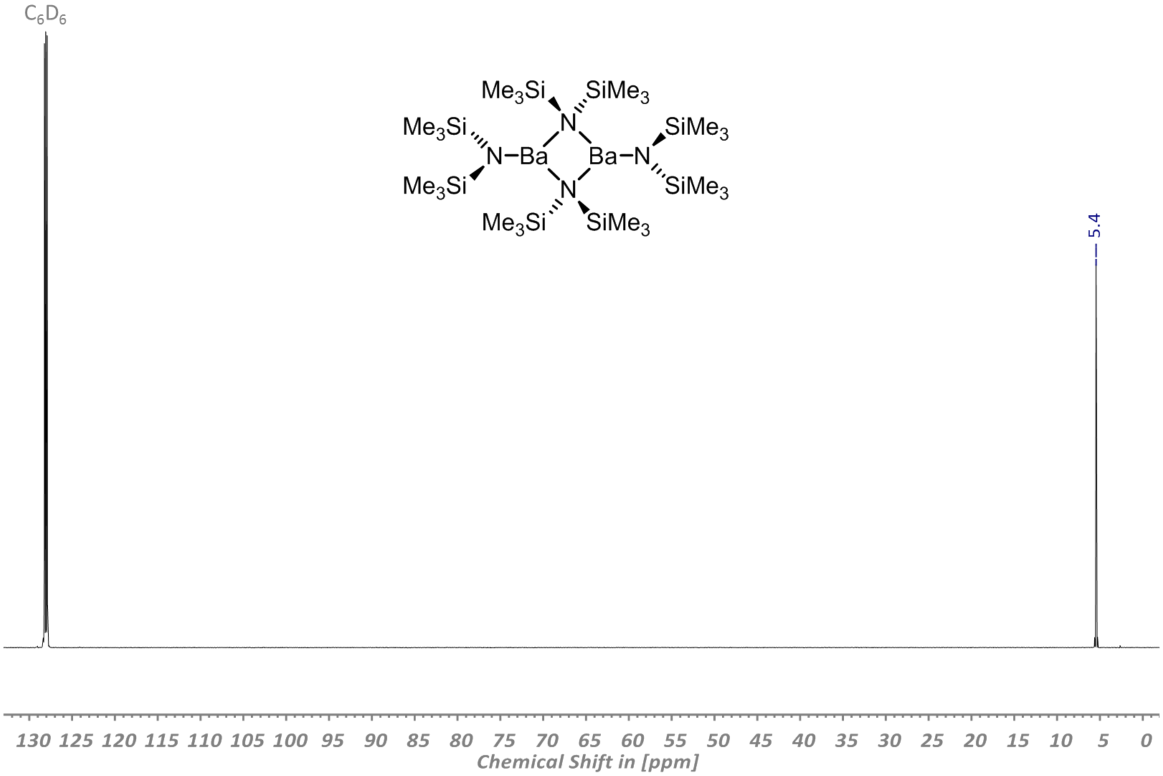

Figure S72. $^{13}C\{^{1}H\}$ NMR (151 MHz, C_6D_6, 25 °C) of $Ba[N(SiMe_3)_2]_2$.

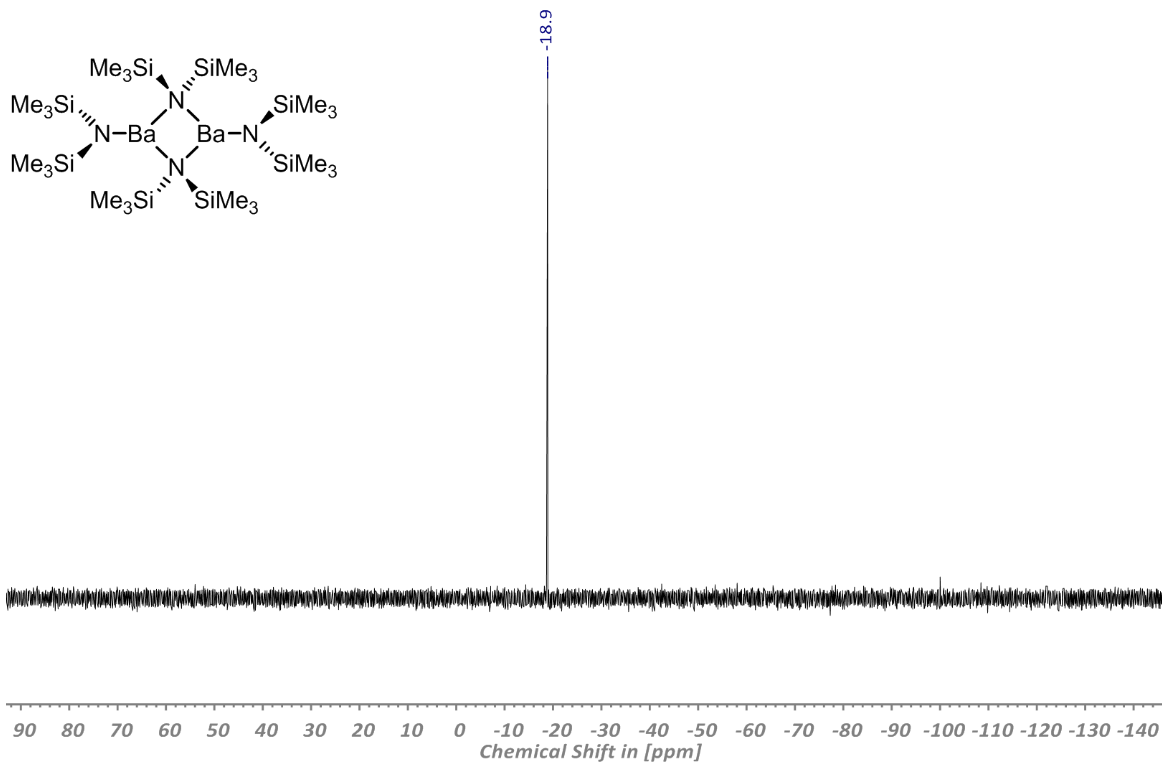

Figure S73. $^{29}Si\{^{1}H\}$ NMR spectrum (119 MHz, C_6D_6, 25 °C) of $Ba[N(SiMe_3)_2]_2$.

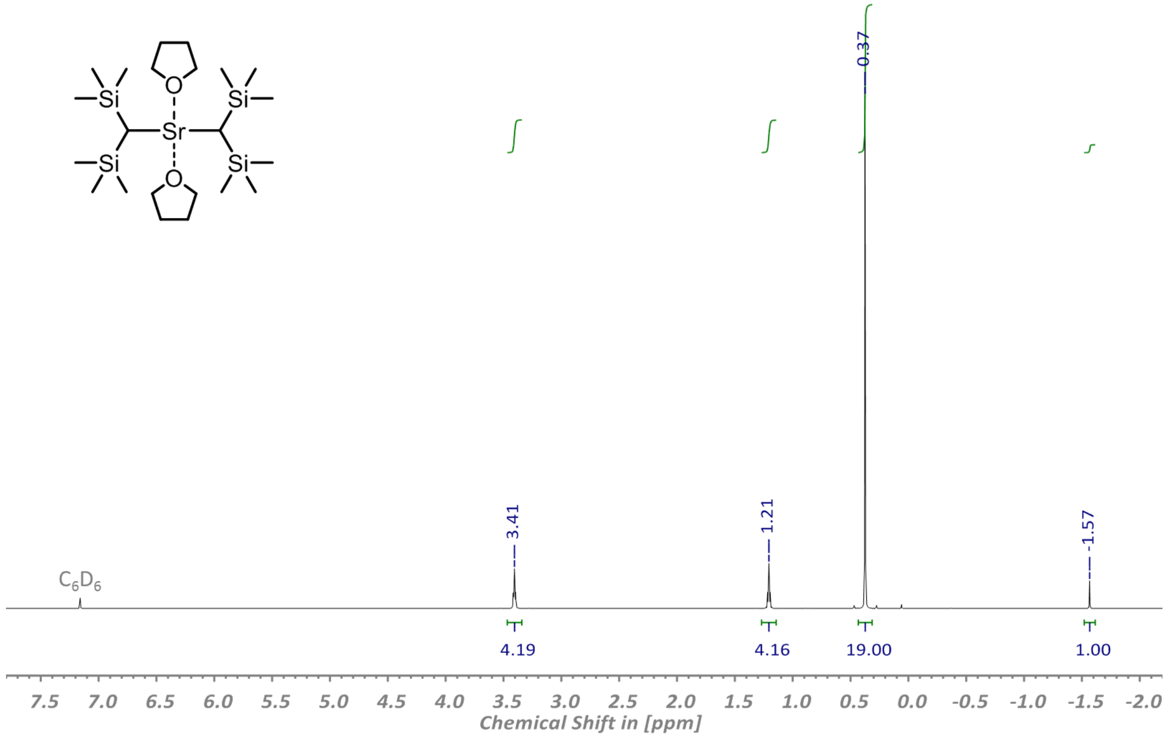

Figure S74. ^{1}H NMR spectrum (600 MHz, C_6D_6, 25 °C) of $Sr[CH(SiMe_3)_2]_2(THF)_2$.

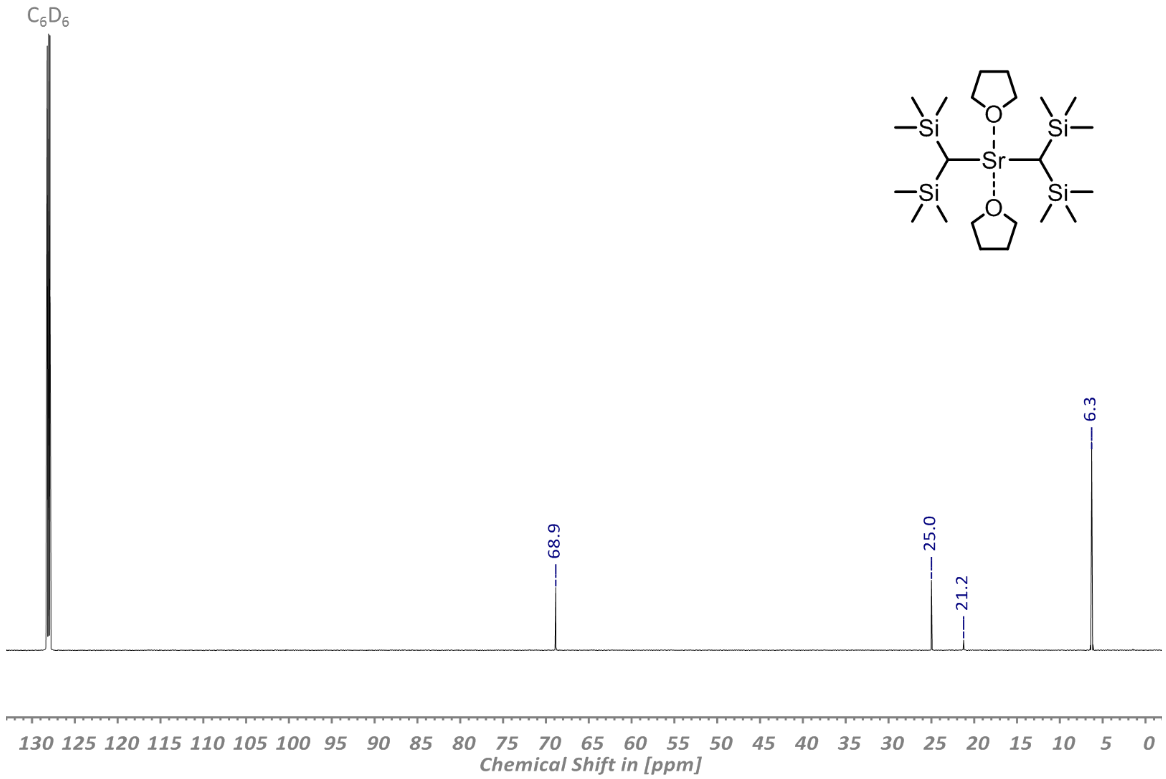

Figure S75. $^{13}C\{^{1}H\}$ NMR (151 MHz, C_6D_6, 25 °C) of $Sr[CH(SiMe_3)_2]_2(THF)_2$.

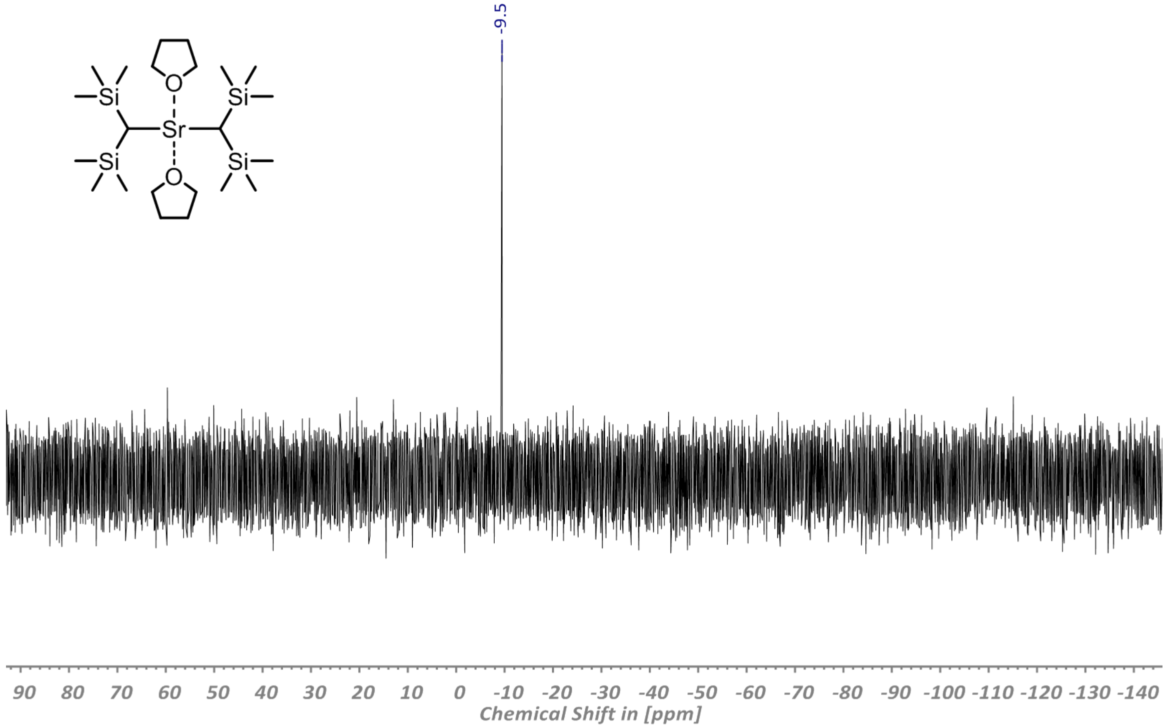

Figure S76. $^{29}Si\{^{1}H\}$ NMR spectrum (119 MHz, C_6D_6, 25 °C) of $Sr[CH(SiMe_3)_2]_2(THF)_2$.

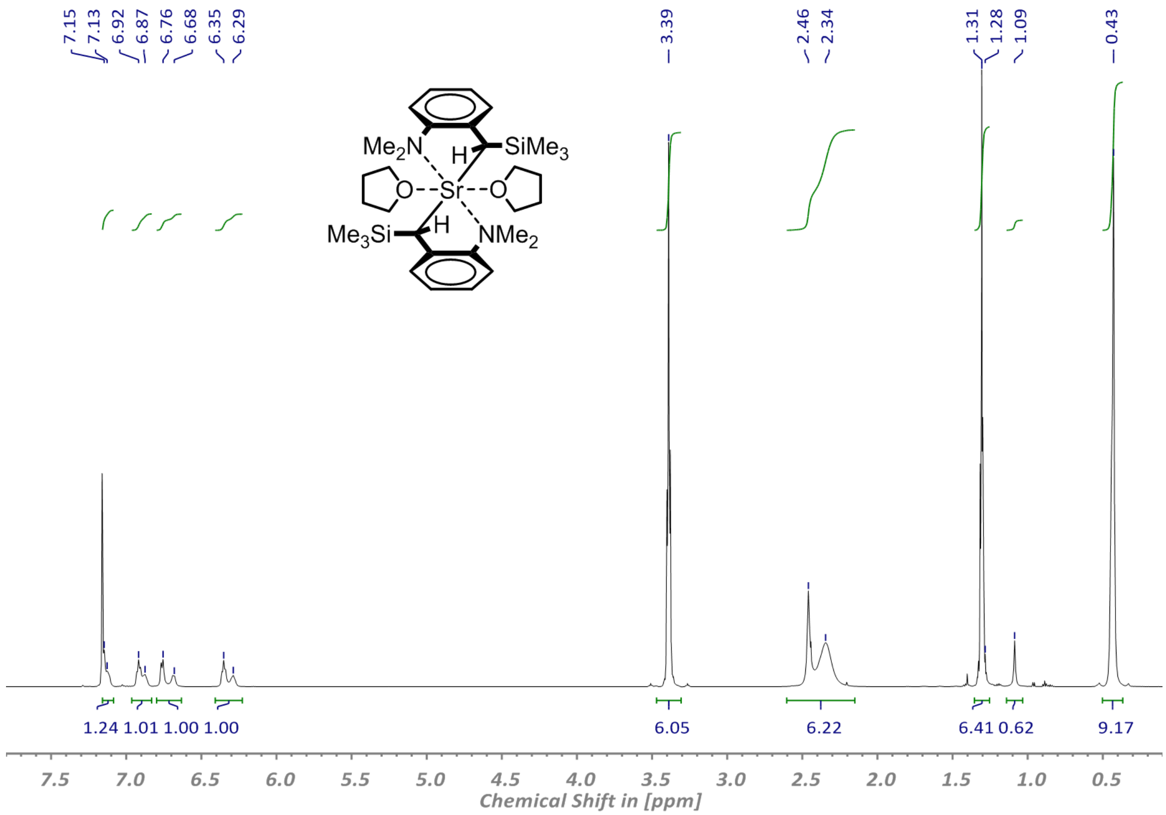

Figure S77. ^{1}H NMR spectrum (600 MHz, C_6D_6, 25 °C) of $[(DMAT)_2Sr(THF)_2](THF)$. *Note:* Two diastereoisomers are present.

5 Catalytic Hydrogenation of Alkenes and Alkynes

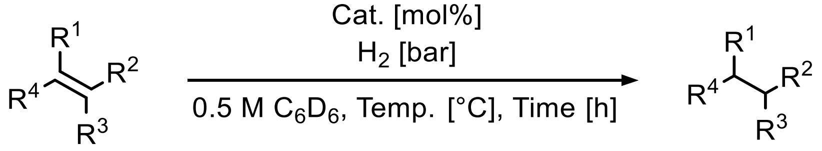

General Catalytic Procedure for Hydrogenation of Alkenes and Alkynes and Product Analysis:

Unless otherwise specified, all hydrogenation reactions of alkenes and alkynes were performed as follows: In an N_2-filled glovebox, an oven-dried miniature stainless steel autoclave (15 mL) containing a magnetic stir bar was charged with the specified crystalline alkaline-earth metal pre-catalyst (0.05 mmol, 10 mol% or 0.025 mmol, 5 mol%), C_6D_6 (1 mL) and the appropriate unsaturated substrate (0.5 mmol). The tightly sealed pressure vessel was transferred out of the glovebox, connected to a H_2 gas manifold and the supply line was evacuated and purged several times with N_2, followed by three times with H_2. The reactor was then pressurized with H_2 (6 or 20 bar), re-sealed and heated to the desired temperature (60 or 120 °C) in a pre-heated aluminum metal block. After stirring the reaction mixture for the indicated amount of time, the autoclave was allowed to cool to room temperature and vented. The reaction mixture was filtered through a glass microfiber filter in a Pasteur pipette and the crude filtrate was analyzed using ^{1}H NMR spectroscopy without additional purification. The identities of the hydrocarbon products were verified by comparison of the obtained ^{1}H NMR spectra with corresponding data either reported in the literature or of authentic samples. The conversion of substrates was estimated by integration of characteristic isolated ^{1}H NMR resonances of the desired saturated product and their residual olefinic counterparts. For cases in which characteristic ^{1}H NMR signals of the alkane product and unsaturated substrate did not separate, a significant degree of deuterium incorporation in the hydrogenated products was observed (as consequence of H/D isotope exchange in the deuterated C_6D_6 solvent) and by-products were formed, respectively, the product composition was analyzed *via* GC/MS. GC yields were determined by integrating the peaks of the hydrocarbon products against their unsaturated analogue. Reactions times for essentially full conversion were optimized in 0.5-hour intervals.

Table S3. Catalytic hydrogenation of alkenes and alkynes with alkaline-earth metal amide pre-catalysts.[a]

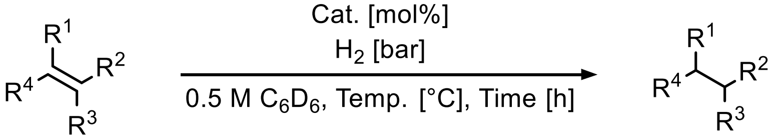

Entry	Catalyst	mol%	Substrate	H_2 [bar]	T [°C]	t [h]	Product(s)	Conv.[b] [%]
1	Ba[N(Si^{i}Pr$_3$)$_2$]$_2$	1	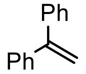	6	120	1.5	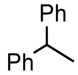	99[c]
2	Ca[N(Si^{i}Pr$_3$)$_2$]$_2$	10		6	120	0.5		98 / 2[c]
3[d]	Ca[N(Si^{i}Pr$_3$)$_2$]$_2$	1		6	120	4		98 / 2[c]
4[d]	Ba[N(SiMe$_3$)$_2$]$_2$	1		6	120	1		50 / 48[c][e]
5	Ba[N(DIPP)(Si^{i}Pr$_3$)]$_2$	10		6	120	0.5		69 / 31[c]
6[d]	Ba[N(DIPP)(Si^{i}Pr$_3$)]$_2$	1	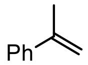	6	120	0.5	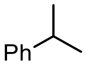 / Dimer	51 / 49[c]
7	Ba[N(Si^{i}Pr$_3$)$_2$]$_2$	10		6	120	0.5		74 / 26[c]
8	Ba[N(Si^{i}Pr$_3$)$_2$]$_2$	10		6	60	2		74 / 26[c]
9[d]	Ba[N(Si^{i}Pr$_3$)$_2$]$_2$	1		6	120	0.5		53 / 44[c][e]
10	Ba[N(Si^{i}Pr$_3$)$_2$]$_2$	1		6	120	0.5		67 / 33[c]
11	Ca[N(Si^{i}Pr$_3$)$_2$]$_2$	10		6	120	0.5		74 / 24[c][e]
12	Ba[N(Si^{i}Pr$_3$)$_2$]$_2$	10	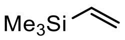	6	120	0.5	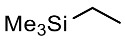 / Dimer	90 / 10[c]
13	Ba[N(Si^{i}Pr$_3$)$_2$]$_2$	10		6	60	2		99 / 0[c]
14[d]	Ba[N(Si^{i}Pr$_3$)$_2$]$_2$	1		6	120	0.5		81 / 16[c][e]
15	Ba[N(Si^{i}Pr$_3$)$_2$]$_2$	10	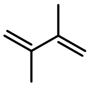	6	120	1	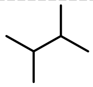 / 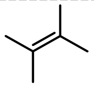	14 / 83[c][e]
16	Ba[N(Si^{i}Pr$_3$)$_2$]$_2$	1	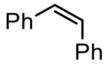	6	120	1	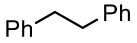	99
17	Ba[N(Si^{i}Pr$_3$)$_2$]$_2$	1	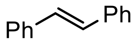	6	120	1	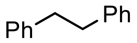	99[c]
18	Ba[N(Si^{i}Pr$_3$)$_2$]$_2$	10	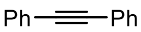	6	120	1	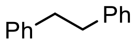	99[c]
19[d]	Ba[N(Si^{i}Pr$_3$)$_2$]$_2$	1	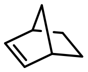	6	120	0.5	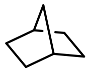	99
20	Mg[N(DIPP)(Si^{i}Pr$_3$)]$_2$	10		6	120	24		11 / 1[c]
21	Mg[N(Si^{i}Pr$_3$)$_2$]$_2$	10		6	120	24		4 / 1[c]
22	Ca[N(DIPP)(Si^{i}Pr$_3$)]$_2$	10	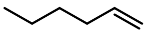	6	120	24	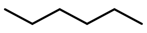 / Isomers	45 / 42[c]
23	Ca[N(Si^{i}Pr$_3$)$_2$]$_2$	10		6	120	3		99[c]
24	Sr[N(DIPP)(Si^{i}Pr$_3$)]$_2$	10		6	120	24		78 / 19[c]
25	Sr[N(Si^{i}Pr$_3$)$_2$]$_2$	10		6	120	0.5		99

Table S3 (contd.). Catalytic hydrogenation of alkenes and alkynes with alkaline-earth metal amide pre-catalysts.[a]

Entry	Catalyst	mol%	Substrate	H_2 [bar]	T [°C]	t [h]	**Product**(s)	Conv.[b] [%]
26	$Ba[N(DIPP)(Si^{i}Pr_3)]_2$	10	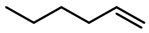	6	120	24	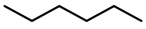 / Isomers	98[c]
27	$Ba[N(Si^{i}Pr_3)_2]_2$	10		6	120	0.5		99
28	$Ba[N(Si^{i}Pr_3)_2]_2$	10		6	60	6		99
29	$Ba[N(Si^{i}Pr_3)_2]_2$	10		6	25	24		10 / 1[c]
30	$Ba[N(Si^{i}Pr_3)_2]_2$	10		1	120	24		10 / 83[c]
31[d]	$Ba[N(Si^{i}Pr_3)_2]_2$	1		6	120	4		99
32	$Ba[N(Si^{i}Pr_3)_2]_2$	10	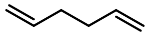	6	120	2	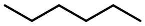 / 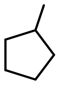	84 / 16[c]
33	$Ba[N(Si^{i}Pr_3)_2]_2$	10	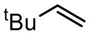	6	120	2	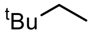	99
34	$Ca[N(Si^{i}Pr_3)_2]_2$	10	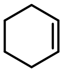	6	120	24	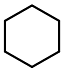	51
35	$Sr[N(Si^{i}Pr_3)_2]_2$	10		6	120	10		99
36	$Ba[N(SiMe_3)_2]_2$	10		6	120	24		3
37	$Ba[N(DIPP)(Si^{i}Pr_3)]_2$	10		6	120	24		36
38	$Ba[N(Si^{i}Pr_3)_2]_2$	10		6	120	3		99
39	$Ba[N(Si^{i}Pr_3)_2]_2$	5		6	120	24		26
40	$Ba[N(Si^{i}Pr_3)_2]_2$	10	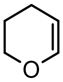	6	120	24	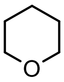	0
41	$Ba[N(Si^{i}Pr_3)_2]_2$	10	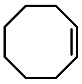	6	120	1.5	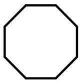	99
42	$Ba[N(Si^{i}Pr_3)_2]_2$	10	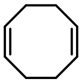	6	120	2.5	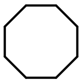 / 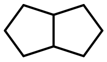	71 / 23[c][e]
43	$Sr[N(Si^{i}Pr_3)_2]_2$	10	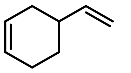	6	120	16	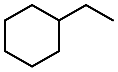	99
44	$Ba[N(Si^{i}Pr_3)_2]_2$	10		6	120	3.5		99
45	$Sr[N(Si^{i}Pr_3)_2]_2$	10	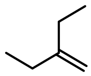	6	120	24	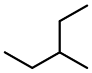 / 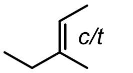	76 / 24[c]
46	$Ba[N(Si^{i}Pr_3)_2]_2$	10		6	120	24		87 / 13[c]
47	$Ba[N(Si^{i}Pr_3)_2]_2$	10	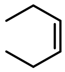	6	120	7	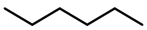	99
48	$Sr[N(Si^{i}Pr_3)_2]_2$	10	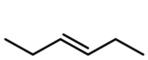	6	120	24	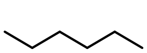	54[c]
49	$Ba[N(Si^{i}Pr_3)_2]_2$	10		6	120	22		99
50	$Ba[N(Si^{i}Pr_3)_2]_2$	10	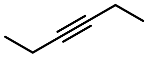	6	120	24	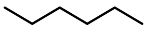	99
51	$Ba[N(Si^{i}Pr_3)_2]_2$	10	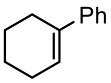	6	120	1	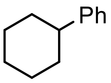	99

Table S3 (contd.). Catalytic hydrogenation of alkenes and alkynes with alkaline-earth metal amide pre-catalysts.[a]

Entry	Catalyst	mol%	Substrate	H_2 [bar]	T [°C]	t [h]	Product(s)	Conv.[b] [%]
52	$Ba[N(Si^iPr_3)_2]_2$	10	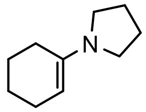	6	120	24	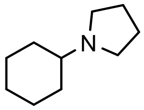	0
53	$Sr[N(Si^iPr_3)_2]_2$	10	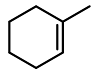	6	120	24	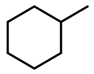	31[c]
54	$Ba[N(Si^iPr_3)_2]_2$	10		6	120	24		81[c]
55[f]	$Ca[N(Si^iPr_3)_2]_2$	10	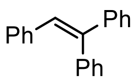	6	120	18	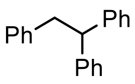	99[c]
56[f]	$Ba[N(Si^iPr_3)_2]_2$	10		6	120	1		99[c]
57[f]	$Ba[N(Si^iPr_3)_2]_2$	10	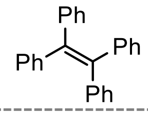	6	120	24	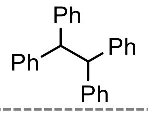	73[c]
58[g]	$Ba[N(Si^iPr_3)_2]_2$	20	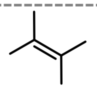	20	120	24	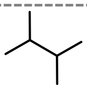	14[c]

[a] General catalytic reaction conditions: [substrate]$_0$ = 0.5 M in C_6D_6 (1 mL); the reaction times for essentially full conversion (> 99%) have been optimized in 0.5 h-intervals; for non-quantitative reactions, the conversion after 24 h is given. [b] Conversion was determined by ^{1}H NMR spectroscopy of the crude reaction mixture by integration of characteristic isolated ^{1}H NMR resonances of the hydrocarbon products and their remaining olefinic counterparts, if procurable; GC/MS analysis was also used to corroborate the product identification and composition. [c] Conversion and product distribution have been determined by GC/MS analysis. [d] Reaction performed with [alkene]$_0$ = 1 M in C_6D_6 (1 mL). [e] The product mixture contained traces (≤3%) of unidentified species. [f] Reaction run at [alkene]$_0$ = 0.25 M in C_6D_6 (1 mL). [g] C_6H_6 (1 mL) used as solvent.

The hydrogenation reactions of the specified alkene substrates described below were performed according to the general catalytic procedure with the following modifications:

Catalytic reactions with catalyst loadings of 1 mol% (Table S3, entries 1, 10, 17 and 18):

Hydrogenation reactions with 1 mol% catalyst loadings were carried out using the general catalytic procedure (*vide supra*), with the specified alkaline-earth metal pre-catalyst (0.01 mmol, 1 mol%), the appropriate unsaturated substrate (1 mmol, 0.5 M) and C_6D_6 (2 mL) as solvent.

1-Hexene (Table S3, entry 30):

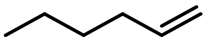

Following the general catalytic procedure described above, the hydrogenation reaction was carried out using 1-hexene (93 µL, 63 mg, 0.75 mmol) as olefinic substrate, $Ba[N(Si^iPr_3)_2]_2$ (60 mg, 0.075 mmol, 10 mol%) and C_6D_6 (1.5 mL). The reactor was connected to a gas manifold and a H_2 gas cylinder. The supply line was then evacuated and flushed several times with N_2, followed by three times with H_2. Upon evacuation of the vessel headspace, H_2 (1 bar) was admitted and the autoclave placed in a preheated aluminum metal block set to 120 °C. The reaction mixture was left to stir for 24 hours, while keeping the H_2 pressure constant in order to provide a sufficient amount of H_2 for the reaction. At the end of the reaction, the reactor was disconnected from the H_2 source and opened to air. The product mixture was filtered through a glass microfiber filter in a Pasteur pipette and analyzed by ^{1}H NMR spectroscopy without further purification. Under these reaction conditions, only a stoichiometric amount of hexane (10%) was formed and the main products were identified as internal hexene isomers (84%) resulting from isomerization of the starting material (7%) by the catalyst, as determined by GC/MS analysis.

Tetraphenylethylene (Table S3, entry 57):

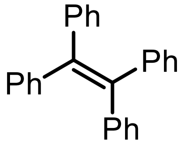

Using the general procedure, the catalytic hydrogenation of tetraphenylethylene (83 mg, 0.25 mmol) with $Ba[N(Si^iPr_3)_2]_2$ (20 mg, 0.025 mmol, 10 mol%) and H_2 (6 bar) was performed with benzene (1 mL) as solvent. At the end of the reaction (24 h), the reaction mixture was diluted with $CHCl_3$ (2 mL) in order to improve solubility and filtered through a glass microfiber filter in a Pasteur pipette for removal of barium contents. The

quantification of the formed product 1,1,2,2-tetraphenylethane was performed *via* GC/MS analysis of the product mixture to yield 73%. Following solvent evaporation under reduced pressure, the residue was dissolved in $CDCl_3$ (700 µL) and further analyzed by ^1H and $^{13}C\{^1H\}$ NMR spectroscopy without additional purification.

Tetramethylethylene (Table S3, entry 58):

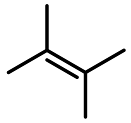

The reaction was carried out following the general procedure for catalytic alkene hydrogenation, using 2,3-dimethyl-2-butene (60 µL, 42.5 mg, 0.5 mmol) as substrate, $Ba[N(Si^iPr_3)_2]_2$ (81 mg, 0.1 mmol, 20 mol%) and benzene (1 mL) as solvent. After stirring the reaction mixture at 120 °C for 24 hours under an atmosphere of H_2 (20 bar), the autoclave was left to cool to ambient temperature and carefully vented in a fume hood. The reaction mixture was then filtered through a glass microfiber filter in a Pasteur pipette. GC/MS analysis of the product mixture revealed that the yield of 2,3-dimethylbutane was 14%. The product mixture was further analyzed using ^1H and $^{13}C\{^1H\}$ NMR spectroscopy following diluting an aliquot of the crude filtrate (150 µL) with C_6D_6 (550 µL).

As described in the general procedure for catalytic alkene hydrogenation above, after the indicated reaction time, the crude reaction mixture was filtered and then analyzed using NMR spectroscopy without additional purification. Consequently, the corresponding parent secondary amine HN(Si^{i}Pr$_3$)$_2$ **(1-H)** and HN(DIPP)(Si^{i}Pr$_3$) **(2-H)**, respectively, as product of catalyst initiation and decomposition is consistently observed in the depicted NMR spectra of the product mixtures. The ^{1}H and ^{13}C{^{1}H} NMR data for both pro-ligands are reported for reference in the following:

Hexa*iso*propyldisilazane HN(Si^{i}Pr$_3$)$_2$ (1-H)[S1]

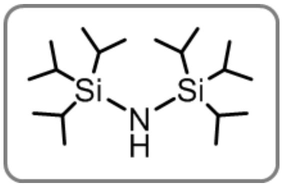

^{1}H NMR (600 MHz, C_6D_6, 25 °C): δ_H = –0.37 (br. s, 1H, N*H*), 1.01 – 1.09 (m, 6H, Si(C*H*Me$_2$)$_3$), 1.13 (d, ^{3}J(H,H) = 6.5 Hz, 36H, Si[CH(C*H*$_3$)$_2$]$_2$) ppm; **^{13}C{^{1}H} NMR** (151 MHz, C_6D_6, 25 °C): δ_C = 13.7 (s, Si(*C*HMe$_2$)$_3$), 19.0 (s, Si[CH(*C*H$_3$)$_2$]$_3$) ppm; **^{29}Si{^{1}H} NMR** (119 MHz, C_6D_6, 25 °C): δ_{Si} = 6.1 (s) ppm. **^{1}H NMR** (600 MHz, CDCl$_3$, 25 °C): δ_H = –0.42 (br. s, 1H, N*H*), 1.01 – 1.07 (m, 42H, Si(C*H*Me$_2$)$_3$ and Si[CH(C*H*$_3$)$_2$]$_3$) ppm; **^{13}C{^{1}H} NMR** (151 MHz, CDCl$_3$, 25 °C): δ_C = 13.5 (s, Si(*C*HMe$_2$)$_3$), 18.9 (s, Si[CH(*C*H$_3$)$_2$]$_3$) ppm; **^{29}Si{^{1}H} NMR** (119 MHz, CDCl$_3$, 25 °C): δ_{Si} = 6.0 (s) ppm.

HN(DIPP)(Si^{i}Pr$_3$) (2-H)[S2]

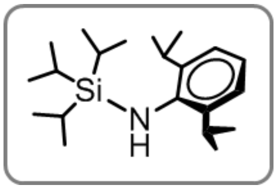

^{1}H NMR (600 MHz, C_6D_6, 25 °C): δ_H = 1.10 (d, ^{3}*J*(H,H) = 7.5 Hz, 18H, Si[CH(C*H*$_3$)$_2$]$_3$), 1.18 – 1.24 (m, 3H, Si(C*H*Me$_2$)$_3$), 1.23 (d, ^{3}*J*(H,H) = 7.0 Hz, 12H, CH(C*H*$_3$)$_2$), 2.42 (br. s., 1H, N*H*), 3.55 (sept, ^{3}*J*(H,H) = 6.8 Hz, 2H, C*H*Me$_2$), 7.04 – 7.07 (m, 1H, *para*-C*H*$_{arom}$), 7.10 – 7.11 (m, 2H, *meta*-C*H*$_{arom}$) ppm; **^{13}C{^{1}H} NMR** (151 MHz, C_6D_6, 25 °C): δ_C = 14.1 (s, Si(*C*HMe$_2$)$_3$), 18.9 (s, Si[CH(*C*H$_3$)$_2$]$_3$), 23.8 (CH(*C*H$_3$)$_2$), 28.5 (*C*HMe$_2$), 123.4 (s, *meta-C*$_{arom}$), 123.9 (s, *para-C*$_{arom}$), 140.9 (s, *ipso-C*$_{arom}$), 143.8 (s, *ortho-C*$_{arom}$) ppm; **^{29}Si{^{1}H} NMR** (119 MHz, C_6D_6, 25 °C): δ_{Si} = 2.9 (s) ppm. The spectroscopic NMR data are in accordance with those previously reported for this compound.[S2]

NMR spectra

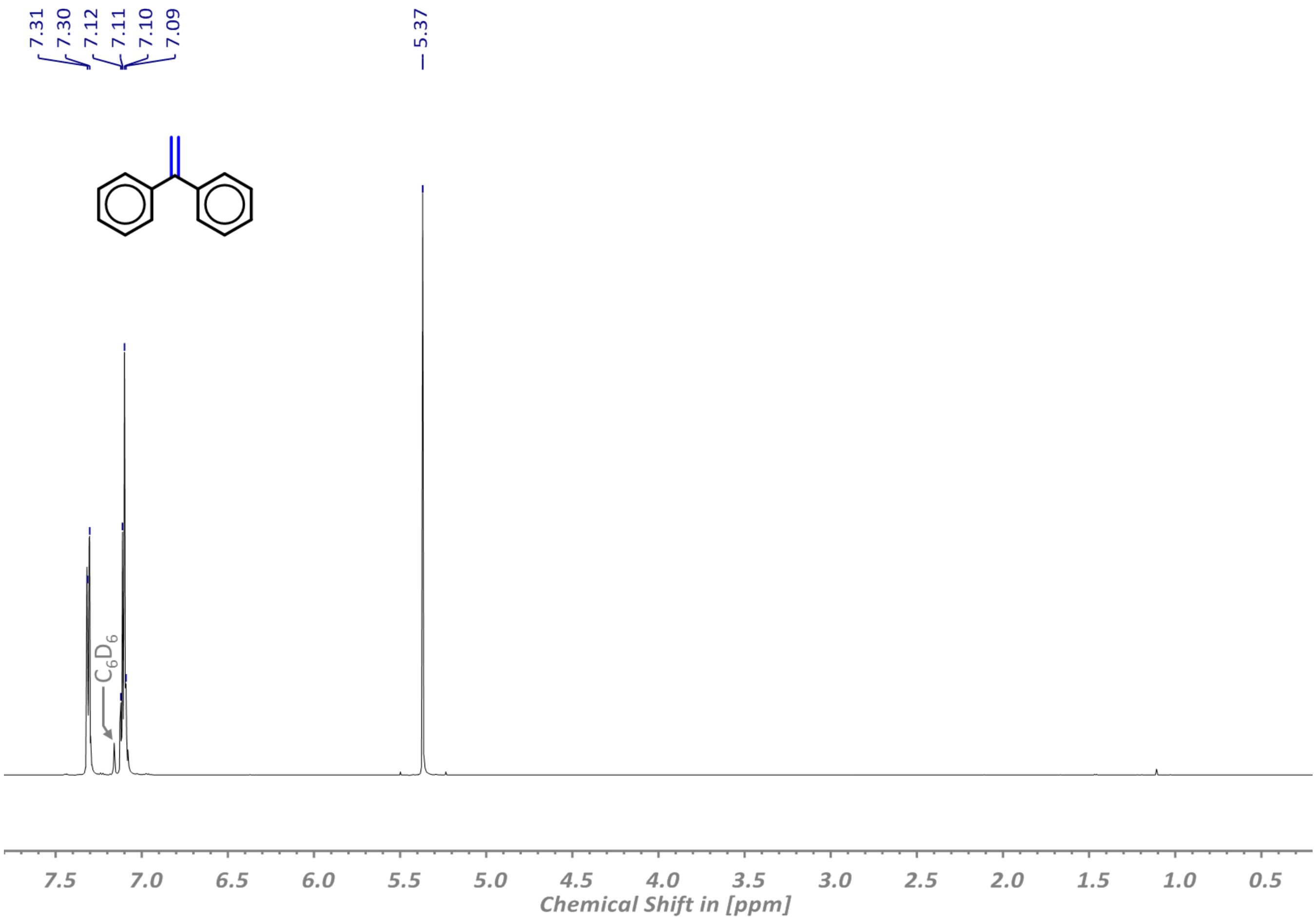

Figure S78. ^{1}H NMR spectrum (600 MHz, C_6D_6, 25 °C) of 1,1-diphenylethylene.

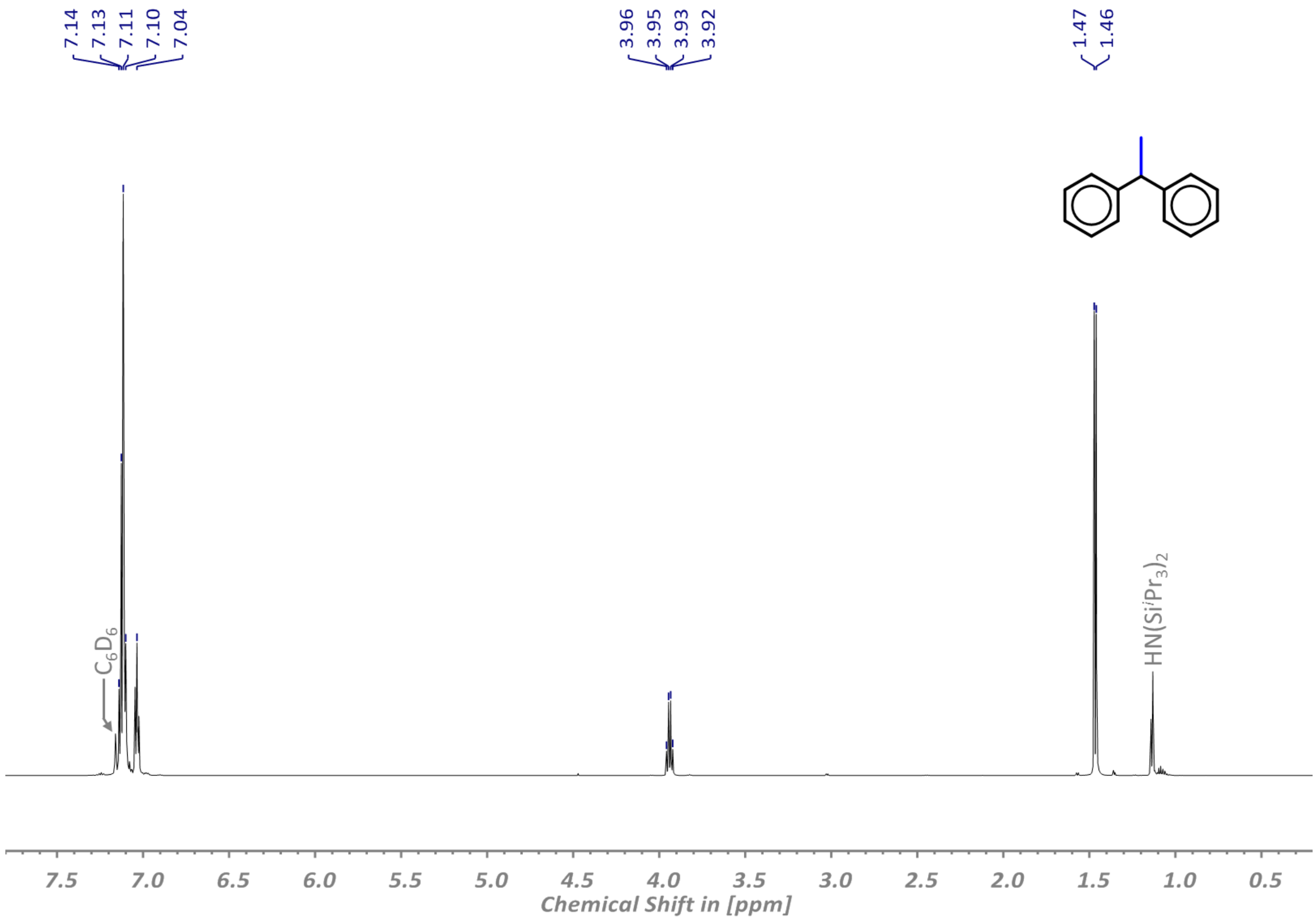

Figure S79. ^{1}H NMR spectrum (600 MHz, C_6D_6, 25 °C) of 1,1-diphenylethane after catalytic hydrogenation (1.5 h) of 1,1-diphenylethylene with $Ba[N(Si^{i}Pr_3)_2]_2$ (**1-Ba**) (1 mol%) and H_2 (6 bar) at 120 °C (Table S3, entry 1).

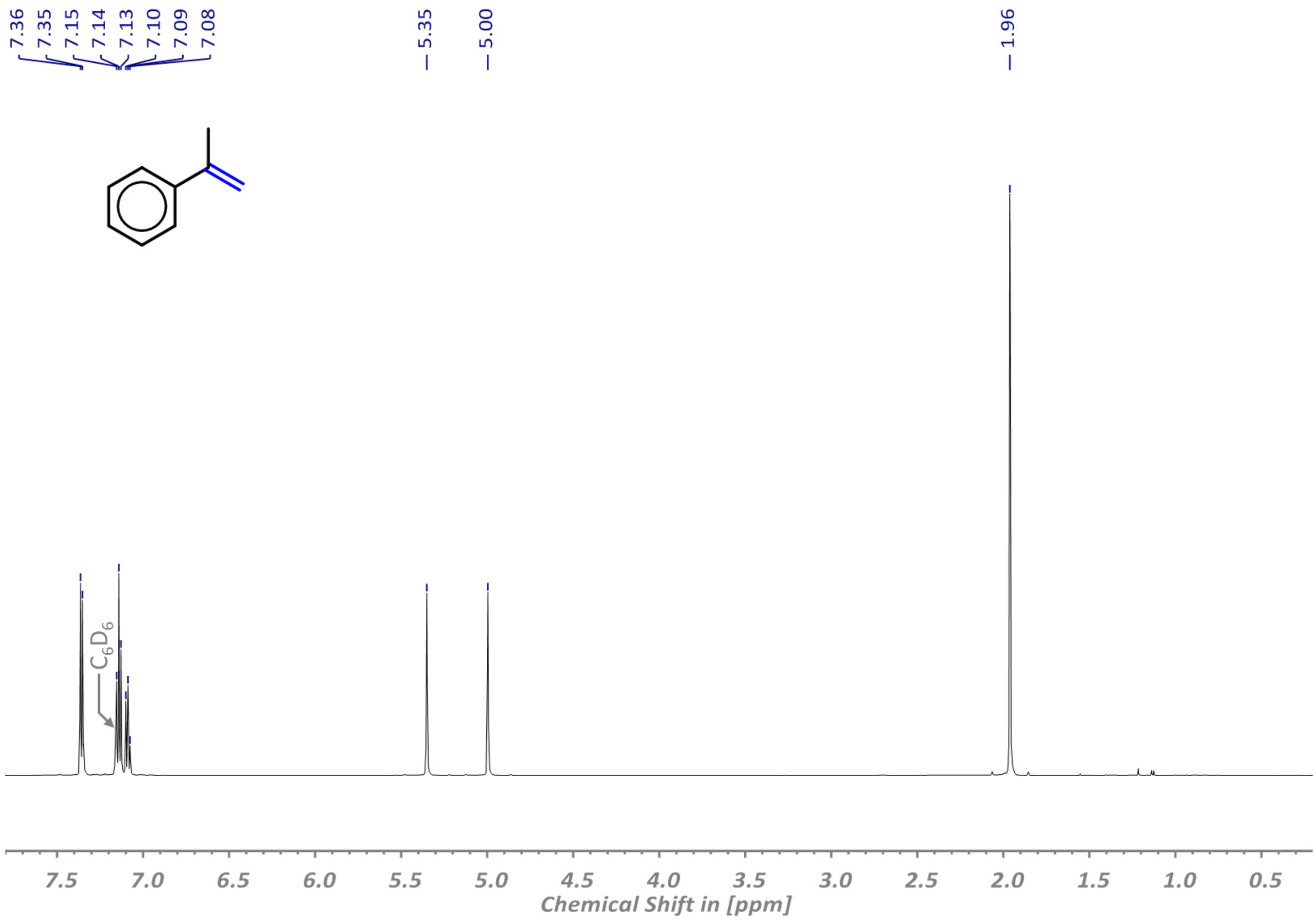

Figure S80. ^{1}H NMR spectrum (600 MHz, C_6D_6, 25 °C) of α-methylstyrene.

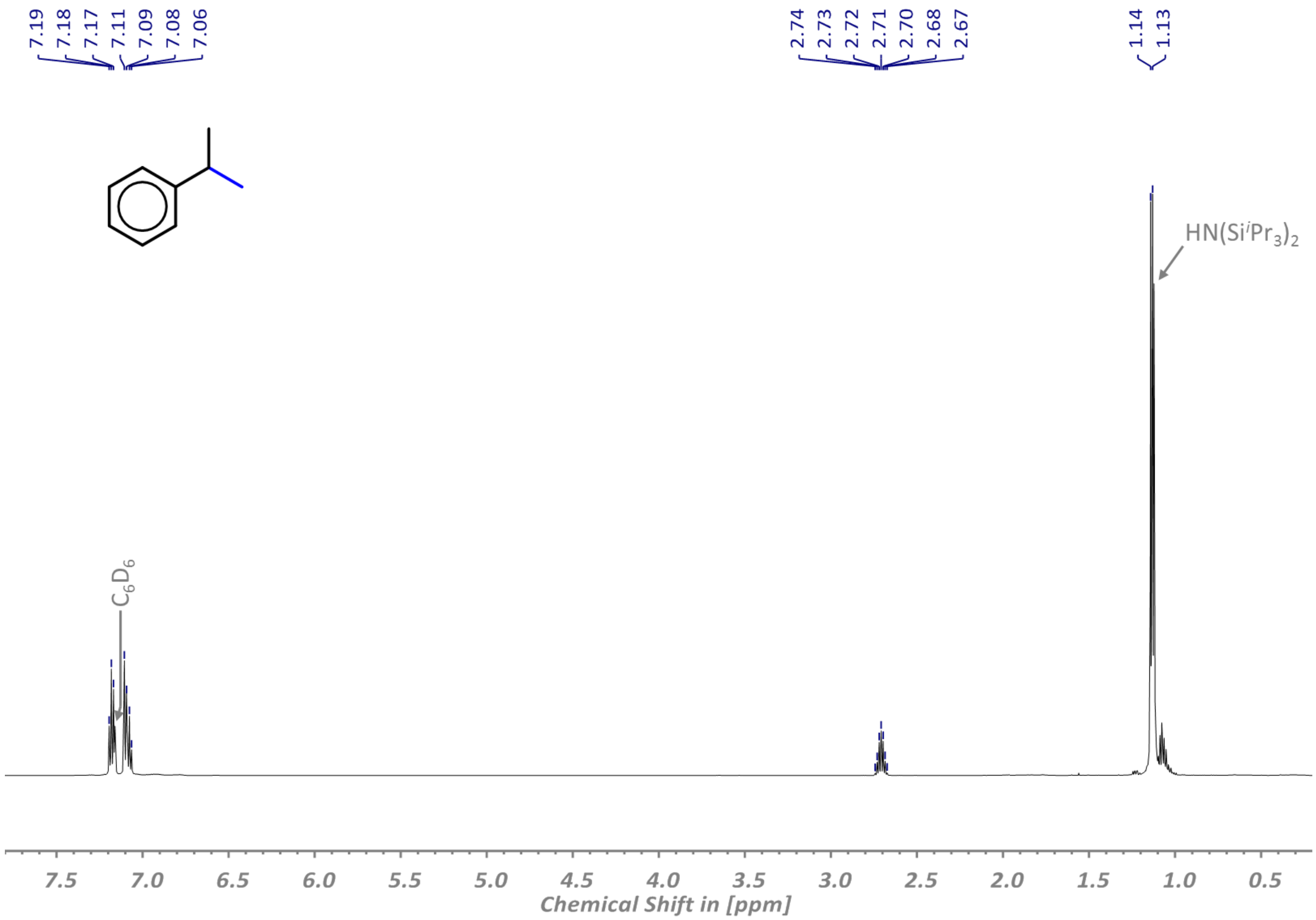

Figure S81. ^{1}H NMR spectrum (600 MHz, C_6D_6, 25 °C) of *iso*propylbenzene after catalytic hydrogenation (0.5 h) of α-methylstyrene with Ca[N(Si^{i}Pr$_3$)$_2$]$_2$ (**1-Ca**) (10 mol%) and H_2 (6 bar) at 120 °C (Table S3, entry 2).

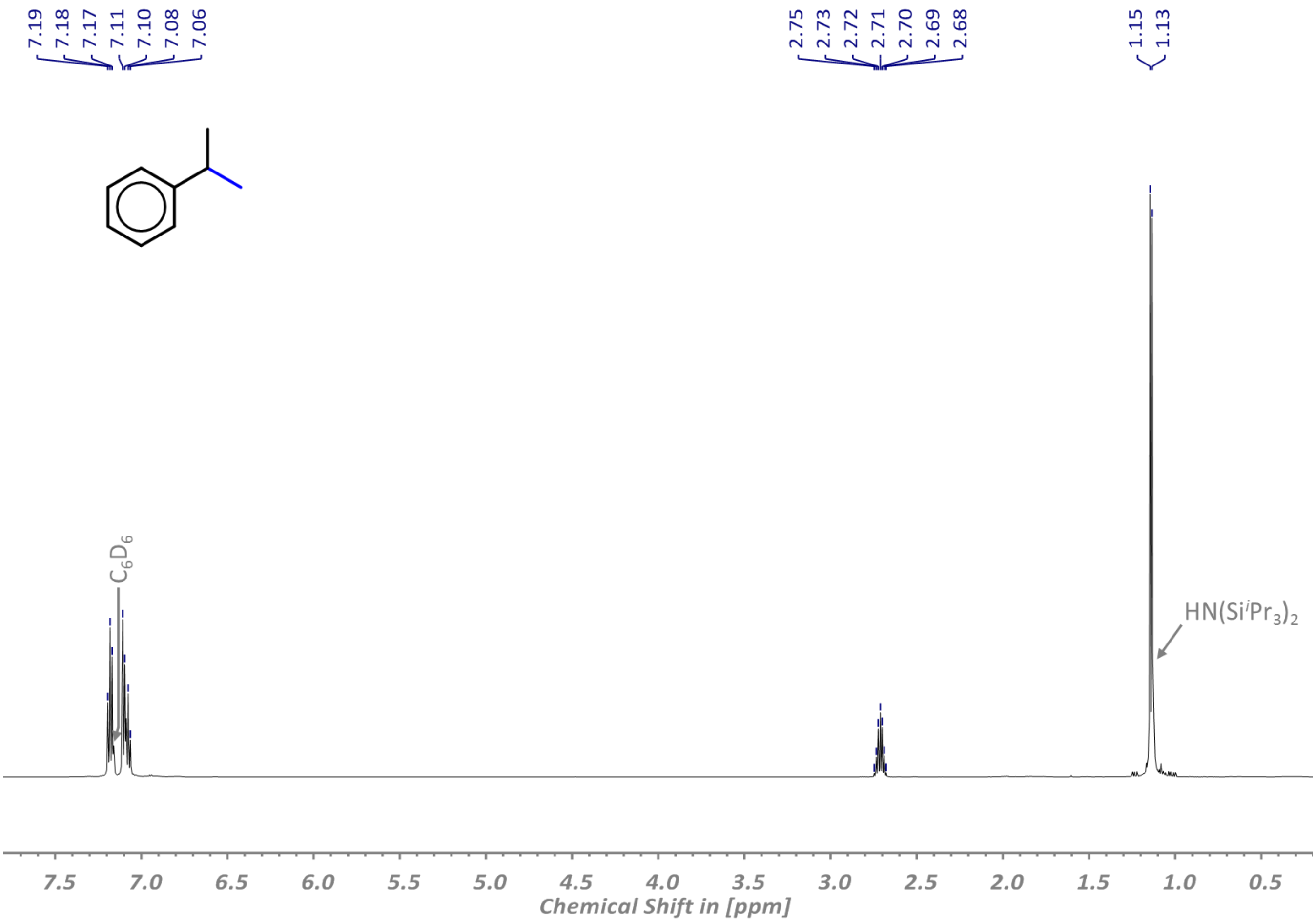

Figure S82. ^{1}H NMR spectrum (600 MHz, C_6D_6, 25 °C) of *iso*propylbenzene after catalytic hydrogenation (4 h) of α-methylstyrene (1 M) with $Ca[N(Si^iPr_3)_2]_2$ (**1-Ca**) (1 mol%) and H_2 (6 bar) at 120 °C (Table S3, entry 3).

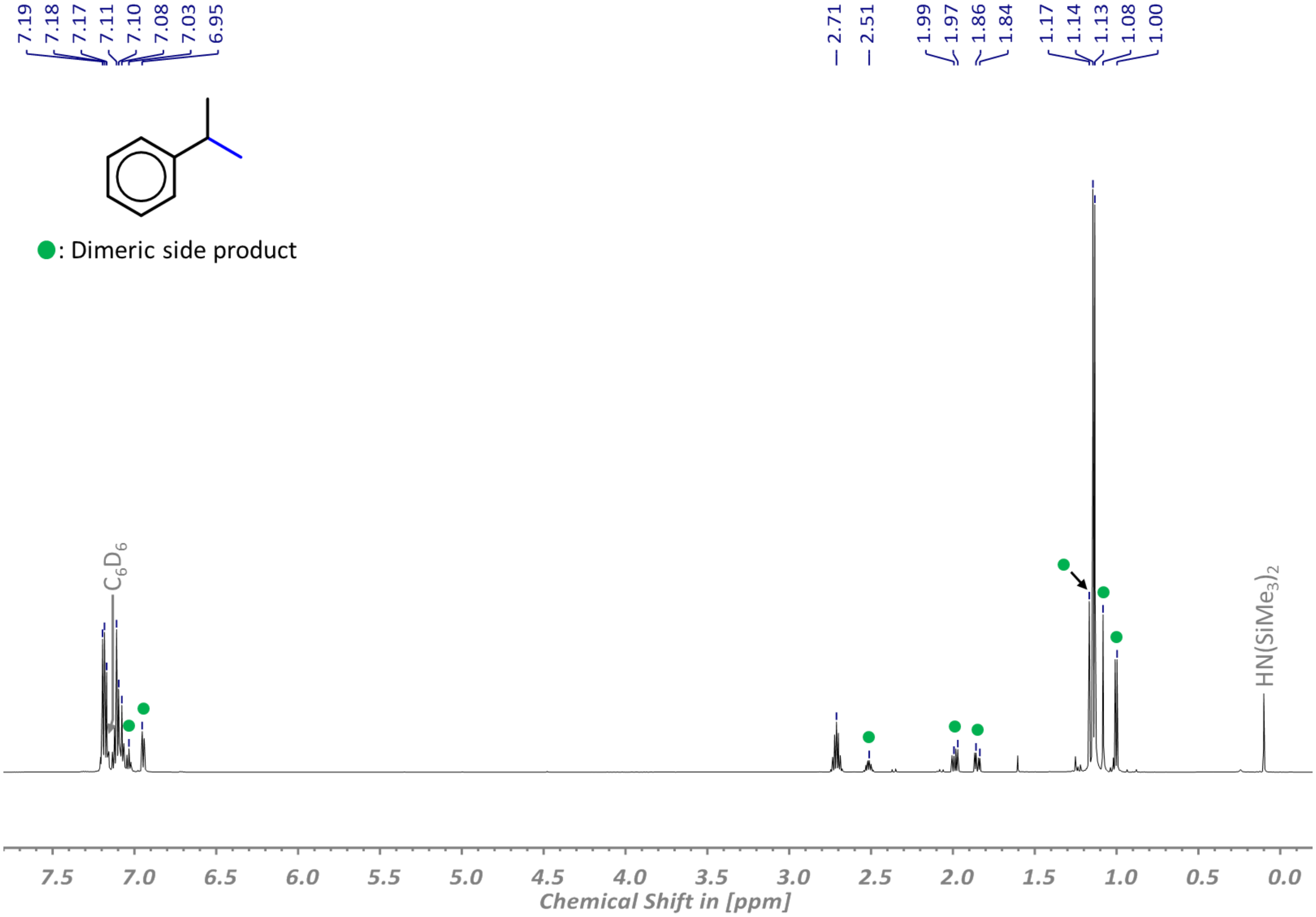

Figure S83. ^{1}H NMR spectrum (600 MHz, C_6D_6, 25 °C) after catalytic hydrogenation (1 h) of α-methylstyrene (1 M) with $Ba[N(SiMe_3)_2]_2$ (1 mol%) and H_2 (6 bar) at 120 °C showing the formation of *iso*propylbenzene and a dimeric side product (●). *Note:* The latter product was tentatively identified as 2-diphenyl-2-methylpentane *via* GC/MS analysis and comparison of the obtained ^{1}H NMR data with those previously reported in the literature.[S15] This compound is probably formed by addition of $Ph(Me)_2CH^-$ to α-methylstyrene followed by hydrogenolysis. A small quantity (2%) of the cyclodimerization product was also observed (Table S3, entry 4).

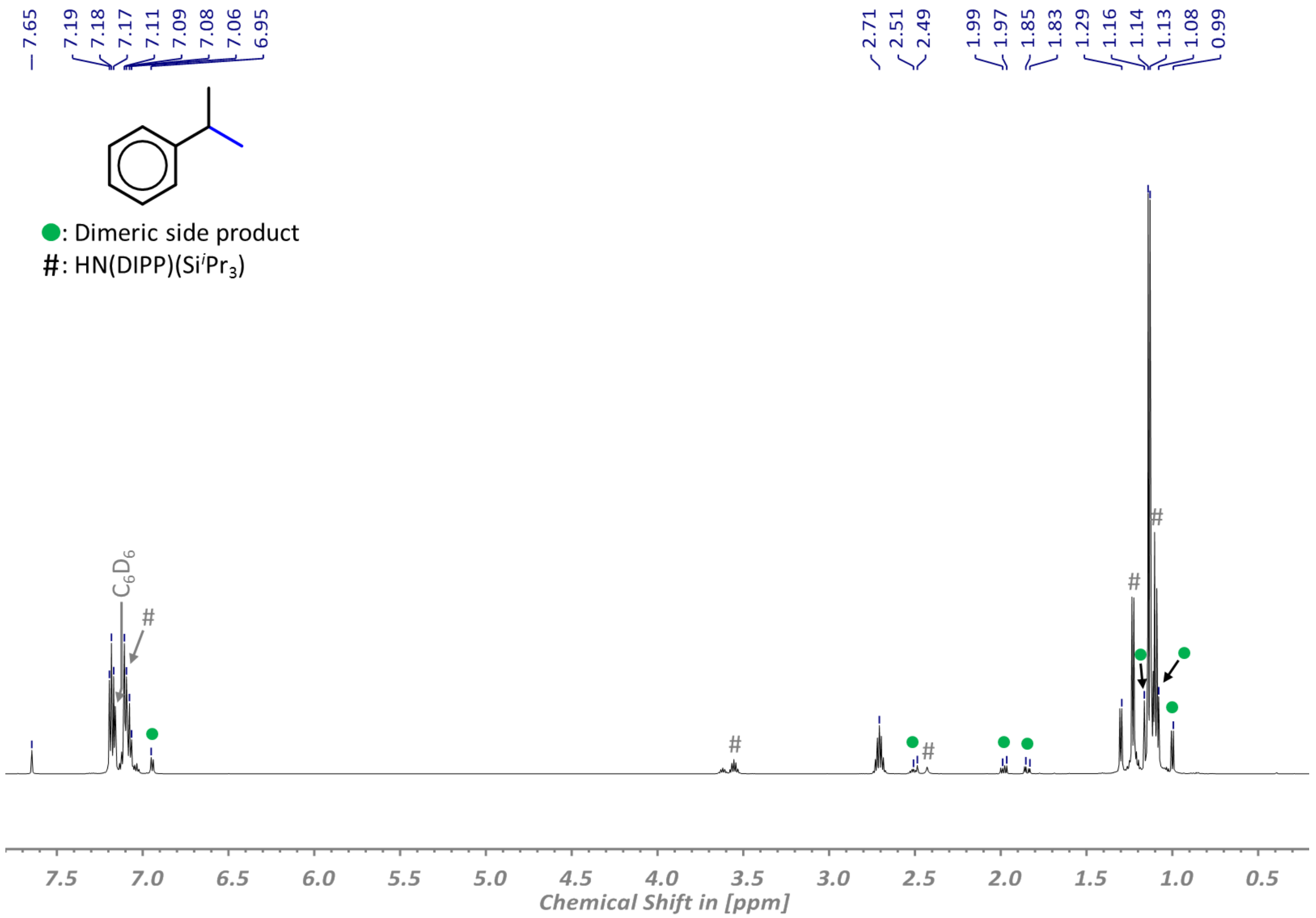

Figure S84. ^{1}H NMR spectrum (600 MHz, C_6D_6, 25 °C) after catalytic hydrogenation (0.5 h) of α-methylstyrene with Ba[N(DIPP)(Si^{i}Pr$_3$)]$_2$ (**2-Ba**) (10 mol%) and H_2 (6 bar) at 120 °C showing the formation of *iso*propylbenzene and a dimeric side product (●). *Note:* # denotes HN(DIPP)(Si^{i}Pr$_3$) (Table S3, entry 5).

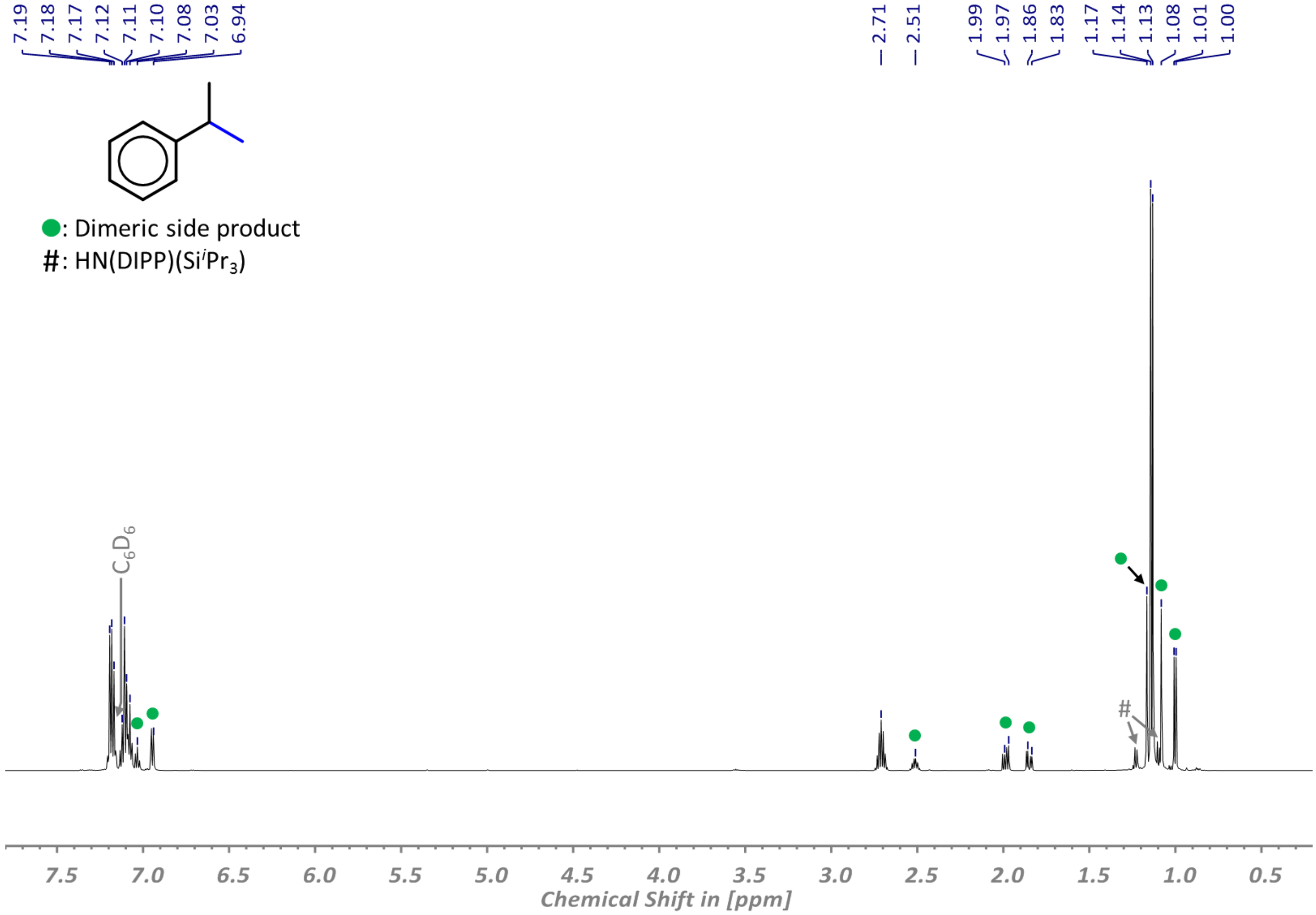

Figure S85. ^{1}H NMR spectrum (600 MHz, C_6D_6, 25 °C) after catalytic hydrogenation (0.5 h) of α-methylstyrene (1 M) with Ba[N(DIPP)(Si^{i}Pr$_3$)]$_2$ (**2-Ba**) (1 mol%) and H_2 (6 bar) at 120 °C showing the formation of *iso*propylbenzene and a dimeric side product (●). *Note:* # denotes HN(DIPP)(Si^{i}Pr$_3$) (Table S3, entry 6).

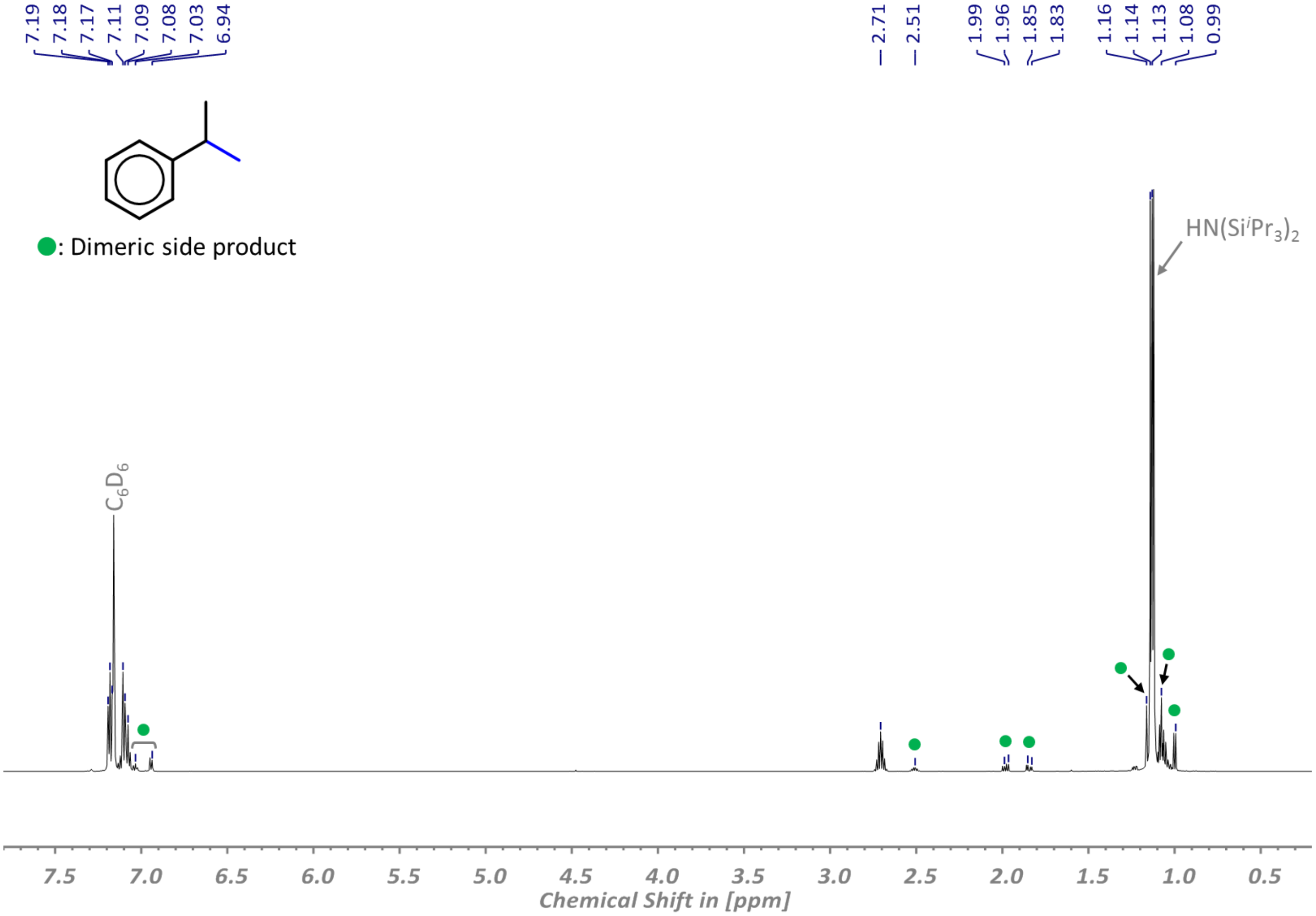

Figure S86. ^{1}H NMR spectrum (600 MHz, C_6D_6, 25 °C) after catalytic hydrogenation (0.5 h) of α-methylstyrene with Ba[N(Si^{i}Pr$_3$)$_2$]$_2$ (**1-Ba**) (10 mol%) and H_2 (6 bar) at 120 °C showing the formation of *iso*propylbenzene and a dimeric side product (●) (Table S3, entry 7).

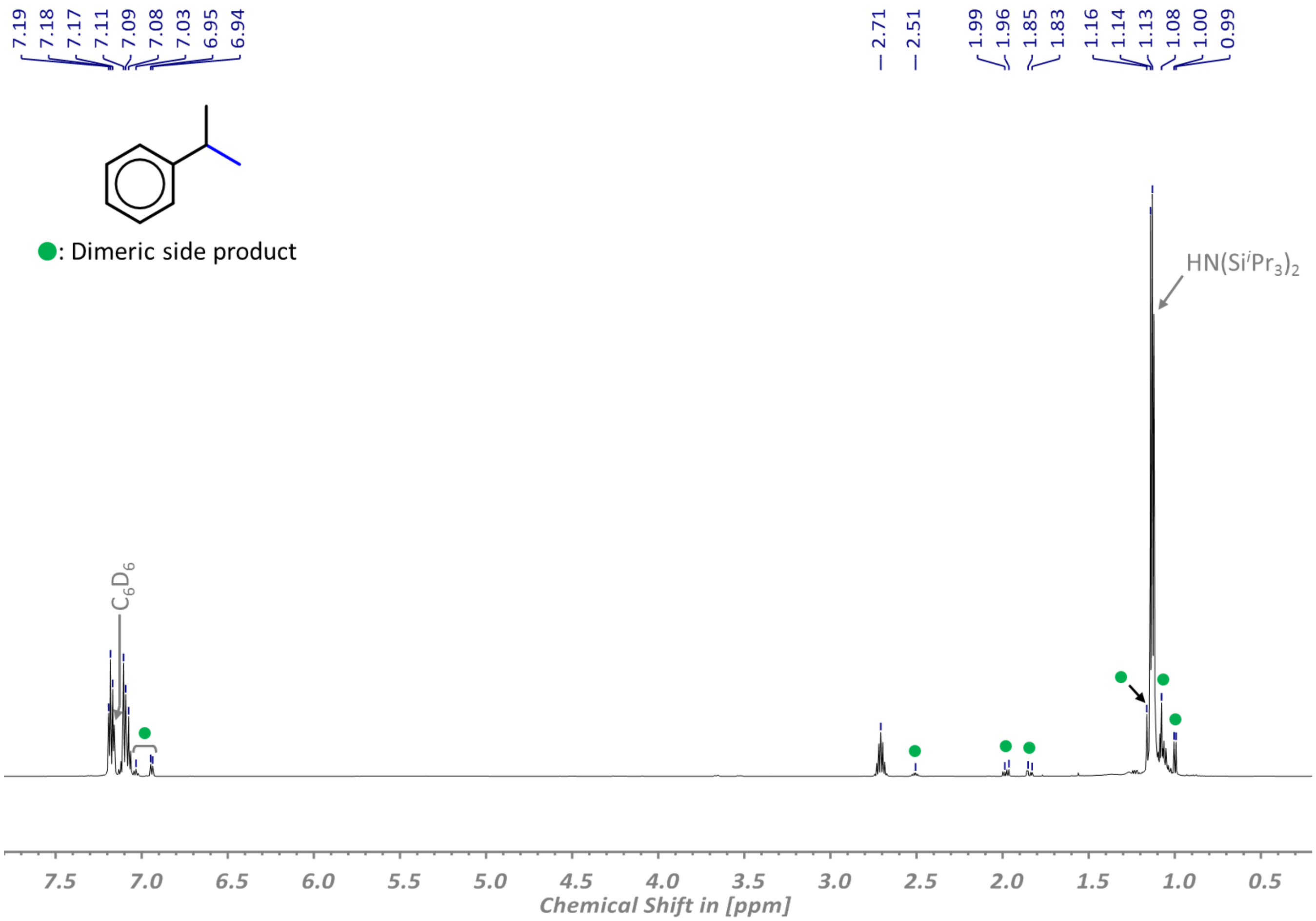

Figure S87. ^{1}H NMR spectrum (600 MHz, C_6D_6, 25 °C) after catalytic hydrogenation (2 h) of α-methylstyrene with $Ba[N(Si^{i}Pr_3)_2]_2$ (**1-Ba**) (10 mol%) and H_2 (6 bar) at 60 °C showing the formation of *iso*propylbenzene and a dimerization side product (●) (Table S3, entry 8).

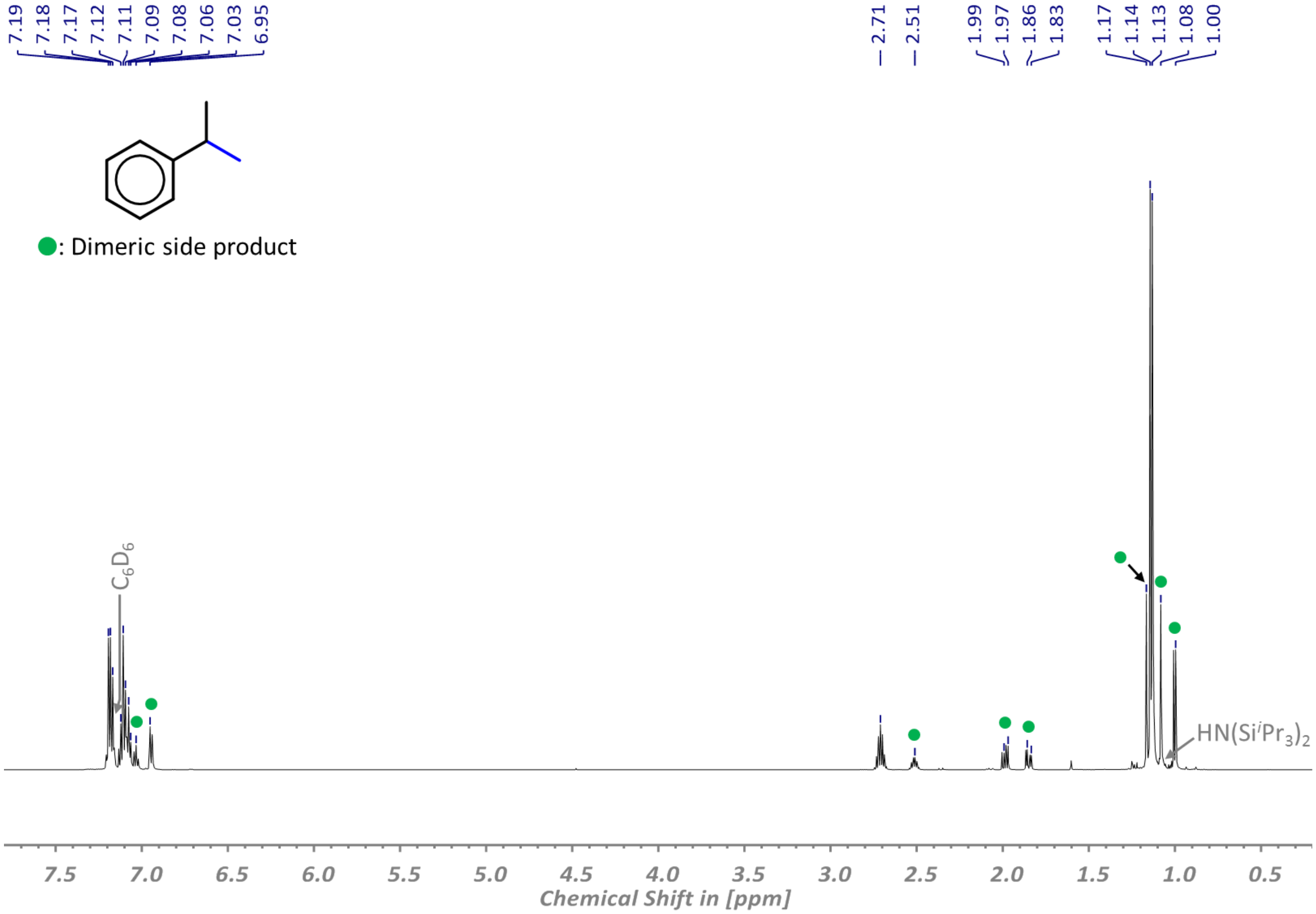

Figure S88. ^{1}H NMR spectrum (600 MHz, C_6D_6, 25 °C) after catalytic hydrogenation (0.5 h) of α-methylstyrene (1 M) with $Ba[N(Si^iPr_3)_2]_2$ (**1-Ba**) (1 mol%) and H_2 (6 bar) at 120 °C showing the formation of *iso*propylbenzene and a dimeric side product (●). *Note:* GC/MS analysis revealed the presence of small quantities (3%) of unidentified products formed during the reaction (Table S3, entry 9).

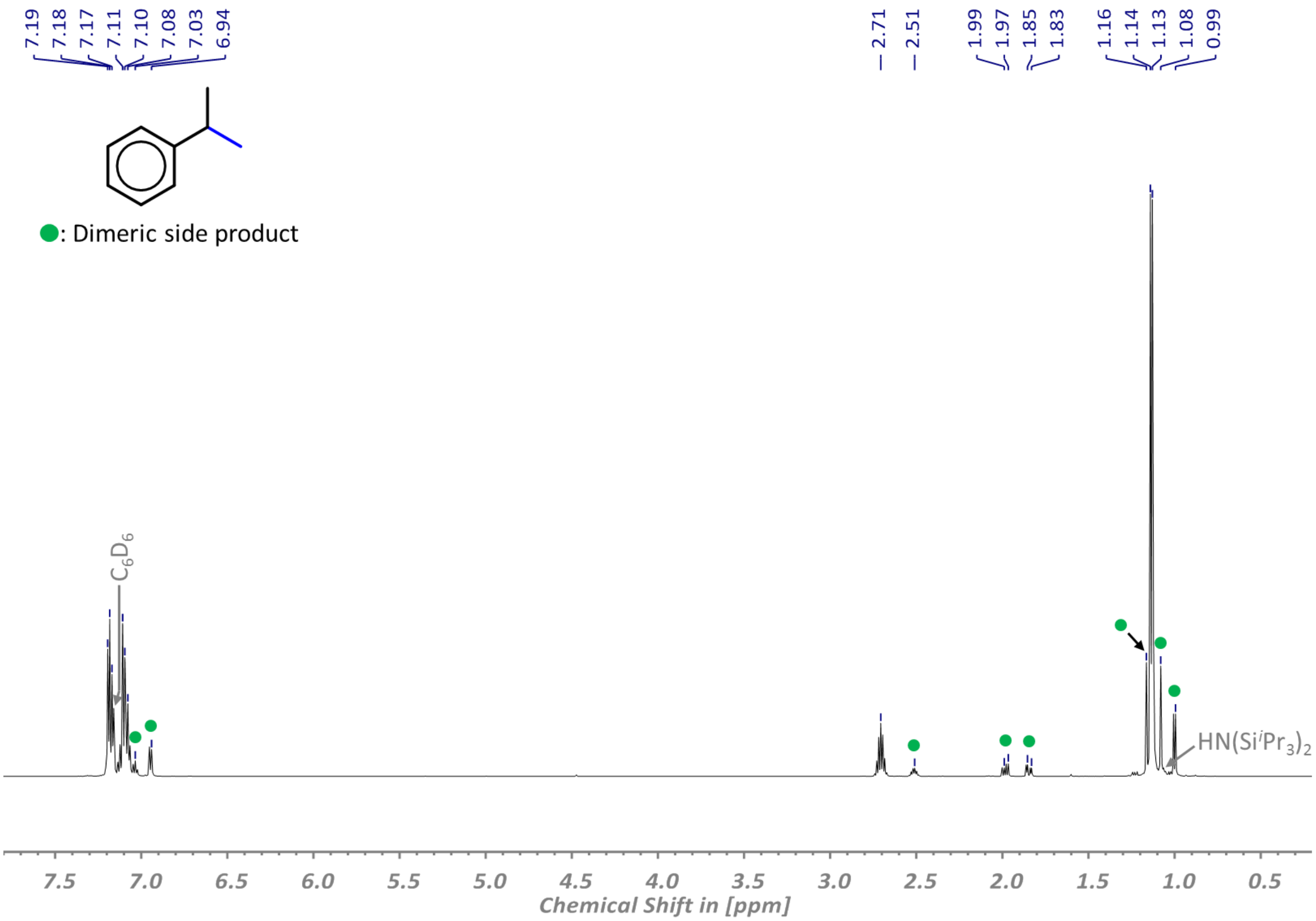

Figure S89. ^{1}H NMR spectrum (600 MHz, C_6D_6, 25 °C) after catalytic hydrogenation (0.5 h) of α-methylstyrene (0.5 M) with $Ba[N(Si^iPr_3)_2]_2$ (**1-Ba**) (1 mol%) and H_2 (6 bar) at 120 °C showing the formation of *iso*propylbenzene and a dimeric side product (●) (Table S3, entry 10).

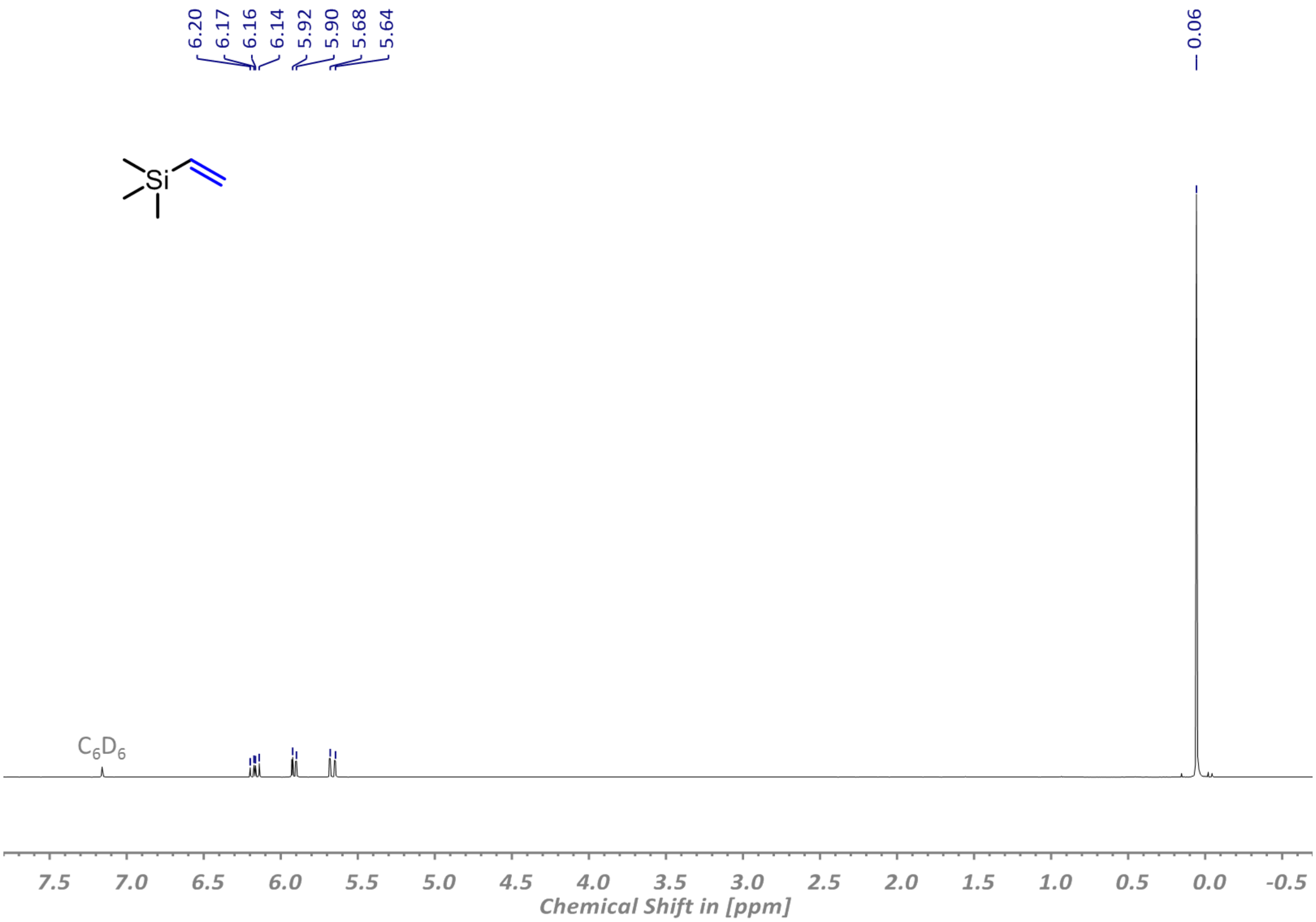

Figure S90. ^{1}H NMR spectrum (600 MHz, C_6D_6, 25 °C) of vinyltrimethylsilane.

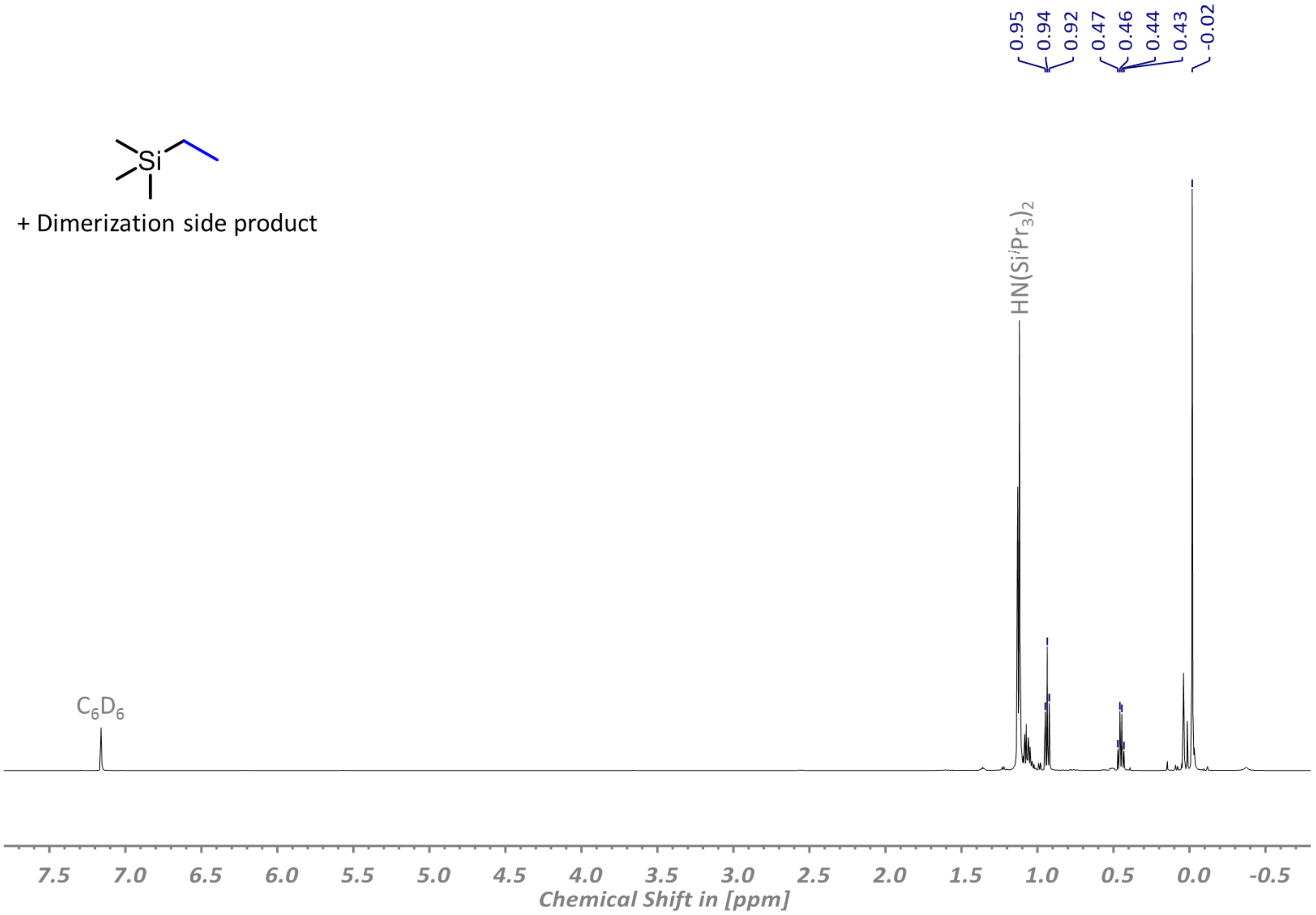

Figure S91. ^{1}H NMR spectrum (600 MHz, C_6D_6, 25 °C) after catalytic hydrogenation (0.5 h) of vinyltrimethylsilane with $Ca[N(Si^{i}Pr_3)_2]_2$ (**1-Ca**) (10 mol%) and H_2 (6 bar) at 120 °C showing the formation of trimethylsilylethane. *Note:* The presence of a dimerization side product,[S16] which most likely formed by addition of $Me_3Si(Me)CH_2^{-}$ to vinyltrimethylsilane followed by hydrogenation, was evidenced by GC/MS analysis. Trace amounts (2%) of unknown products are also observed (Table S3, entry 11).

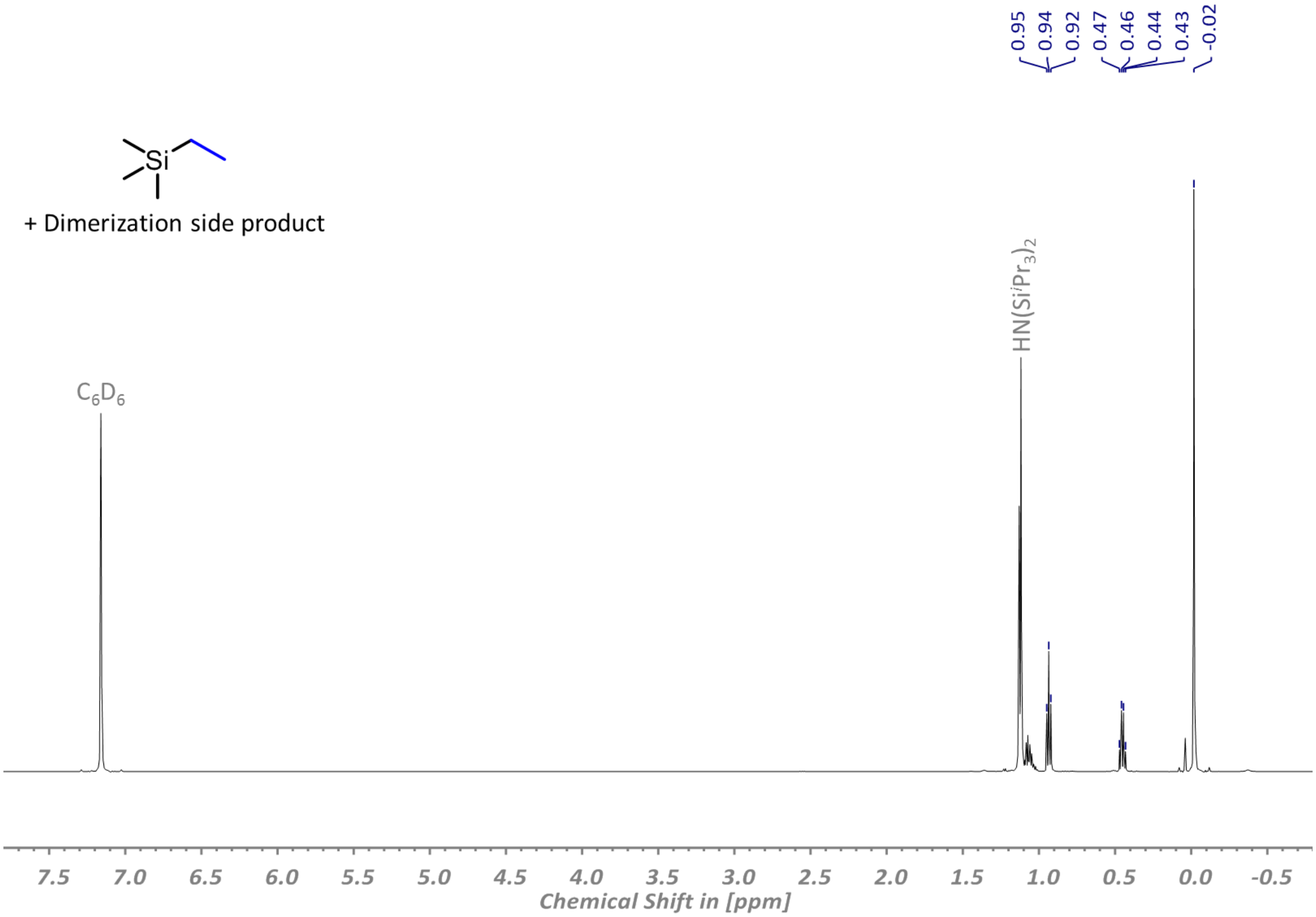

Figure S92. ^{1}H NMR spectrum (600 MHz, C_6D_6, 25 °C) after catalytic hydrogenation (0.5 h) of vinyltrimethylsilane with Ba[N(Si^{i}Pr$_3$)$_2$]$_2$ (**1-Ba**) (10 mol%) and H_2 (6 bar) at 120 °C showing the formation of trimethylsilylethane and a dimerization side product (Table S3, entry 12).

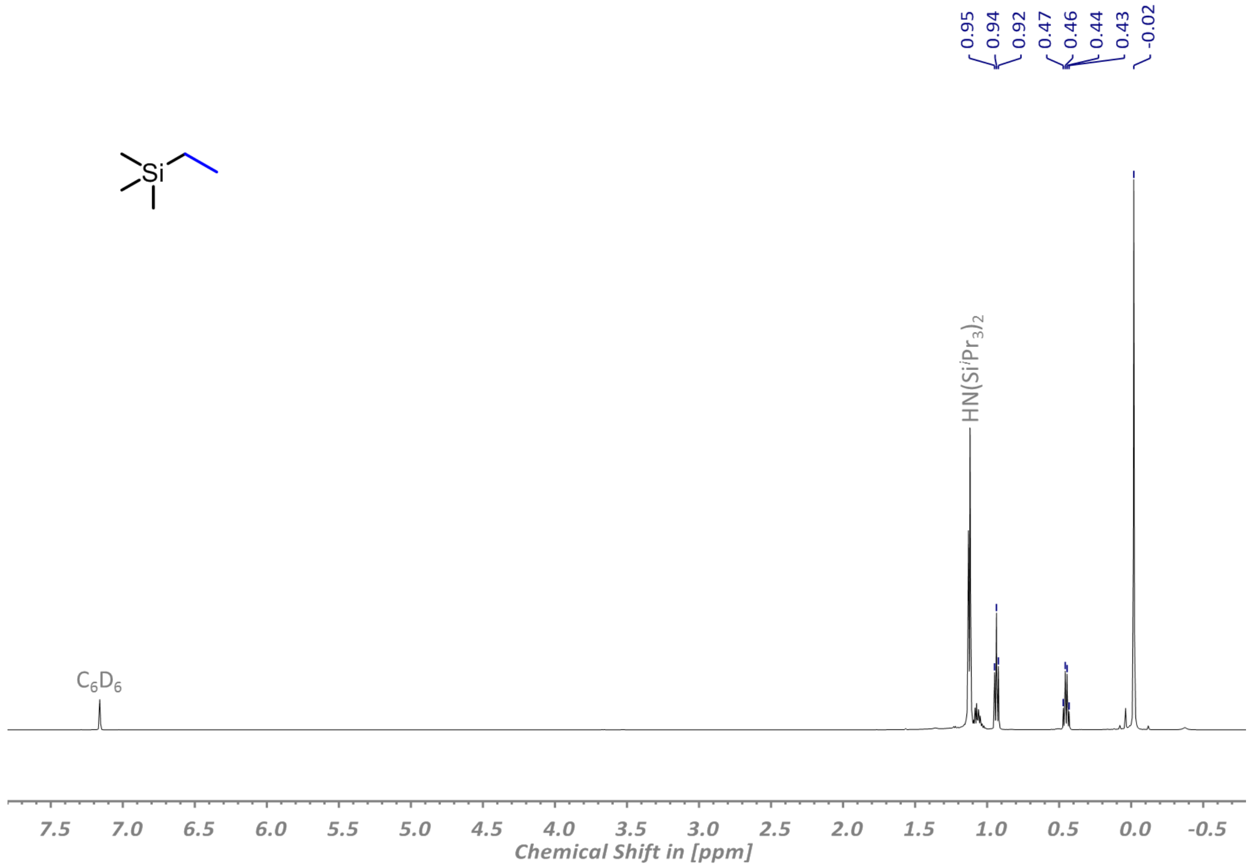

Figure S93. ^{1}H NMR spectrum (600 MHz, C_6D_6, 25 °C) of trimethylsilylethane after catalytic hydrogenation (2 h) of vinyltrimethylsilane with Ba[N(Si^{i}Pr$_3$)$_2$]$_2$ (**1-Ba**) (10 mol%) and H_2 (6 bar) at 60 °C (Table S3, entry 13).

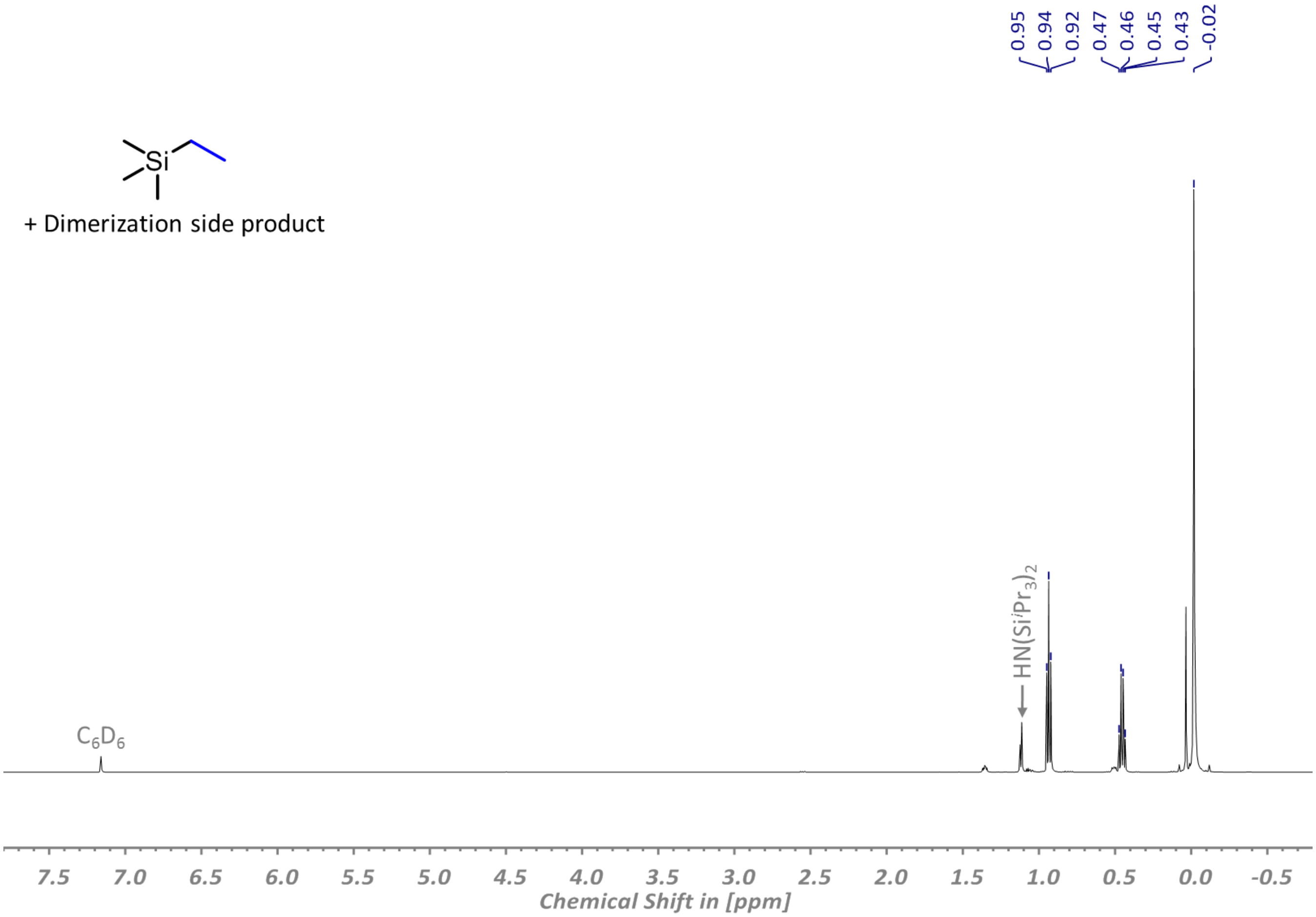

Figure S94. ^{1}H NMR spectrum (600 MHz, C_6D_6, 25 °C) after catalytic hydrogenation (0.5 h) of (trimethylsilyl)ethylene (1 M) with Ba[N(Si^{i}Pr$_3$)$_2$]$_2$ (**1-Ba**) (1 mol%) and H_2 (6 bar) at 120 °C showing the formation of trimethylsilylethane and a dimerization side product. *Note:* The product mixture contained traces (3%) of unidentified products as confirmed by GC/MS analysis (Table S3, entry 14).

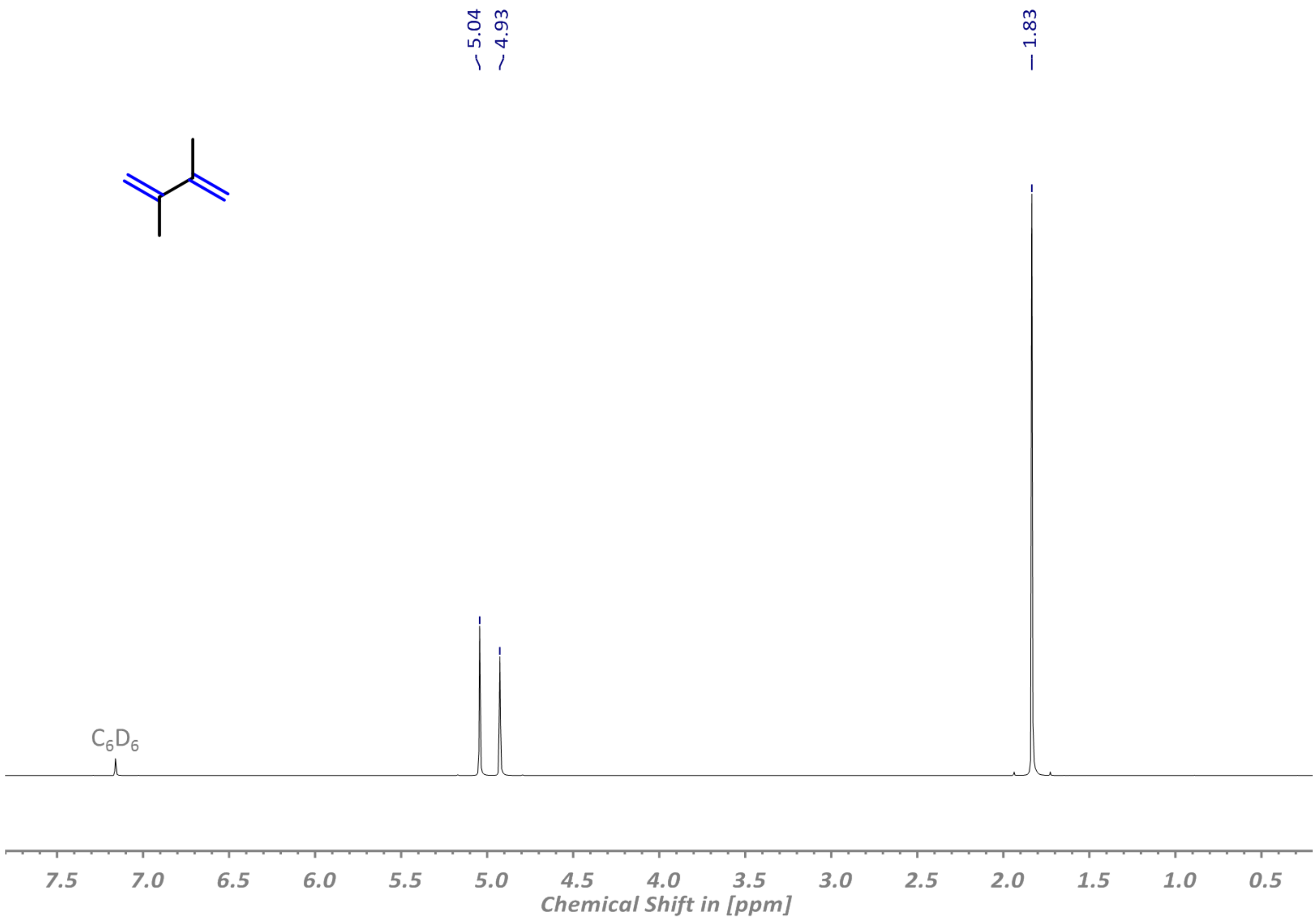

Figure S95. ^{1}H NMR spectrum (600 MHz, C_6D_6, 25 °C) of 2,3-dimethyl-1,3-butadiene.

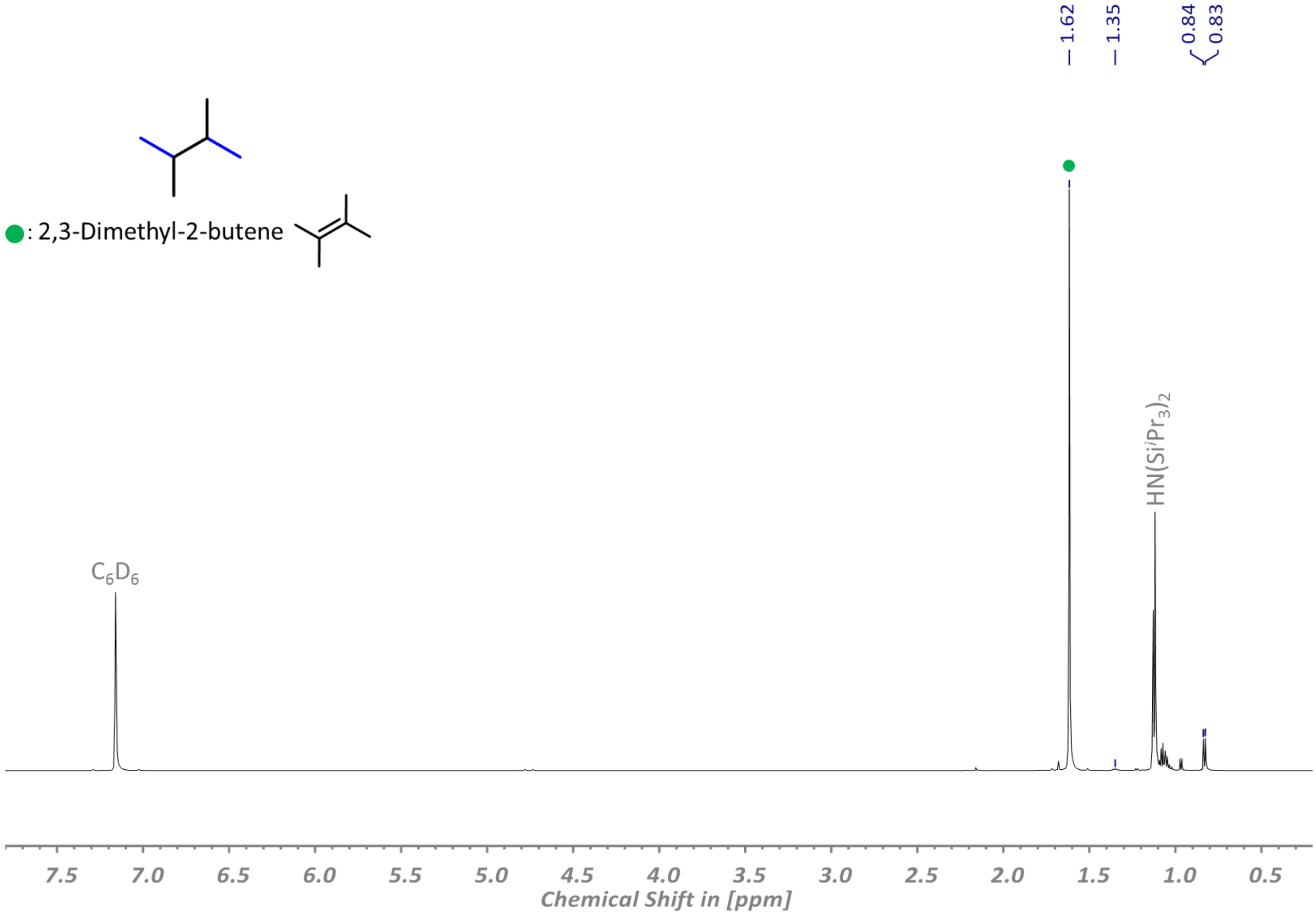

Figure S96. ^1H NMR spectrum (600 MHz, C_6D_6, 25 °C) after catalytic hydrogenation (1 h) of 2,3-dimethyl-1,3-butadiene with $Ba[N(Si^iPr_3)_2]_2$ (**1-Ba**) (10 mol%) and H_2 (6 bar) at 120 °C showing the formation of 2,3-dimethylbutane and mainly 2,3-dimethyl-2-butene (●). *Note:* GC/MS analysis of the product mixture revealed the formation of small amounts of unidentified species (3%) in this reaction (Table S3, entry 15).

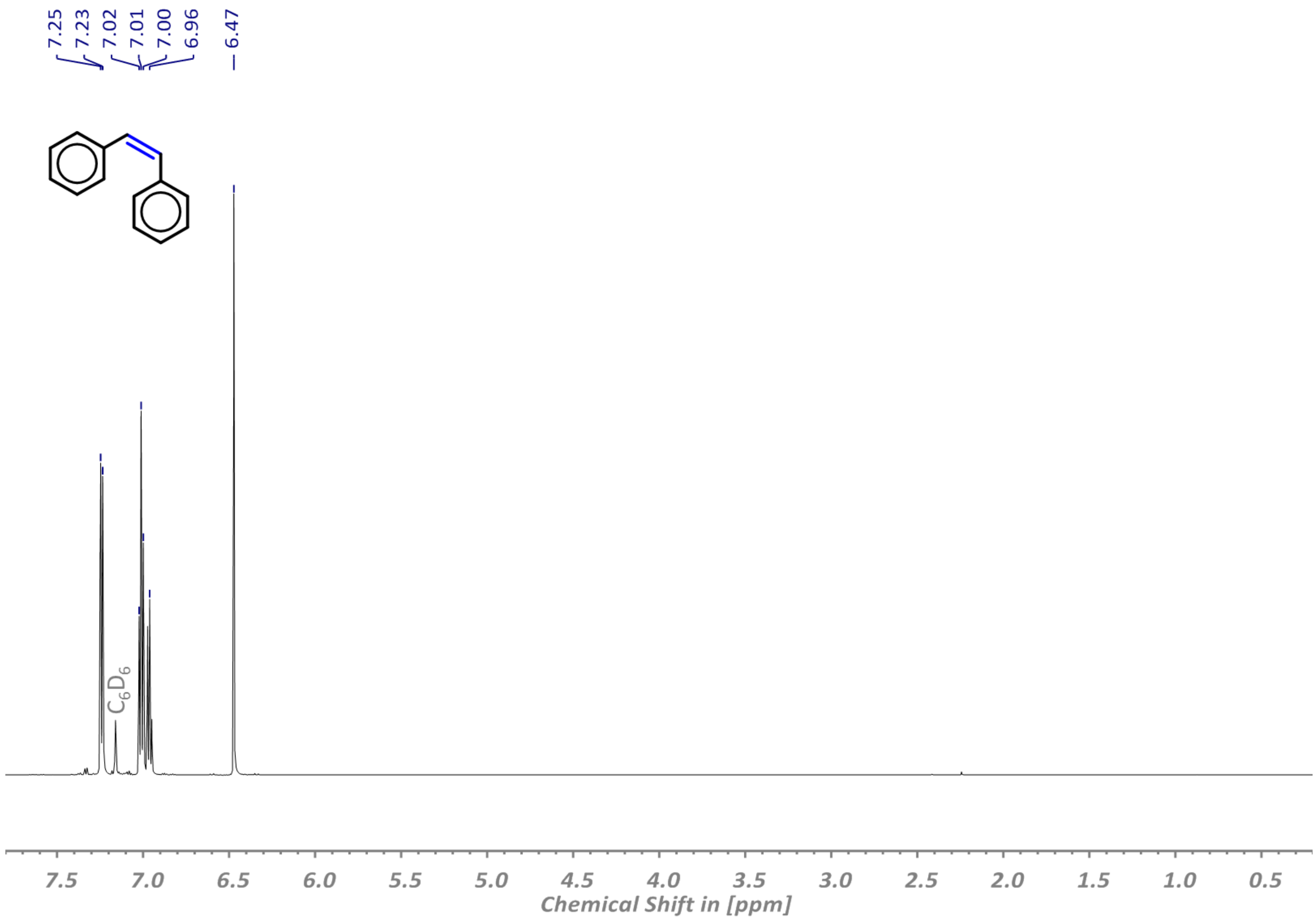

Figure S97. ^{1}H NMR spectrum (600 MHz, C_6D_6, 25 °C) of *cis*-stilbene.

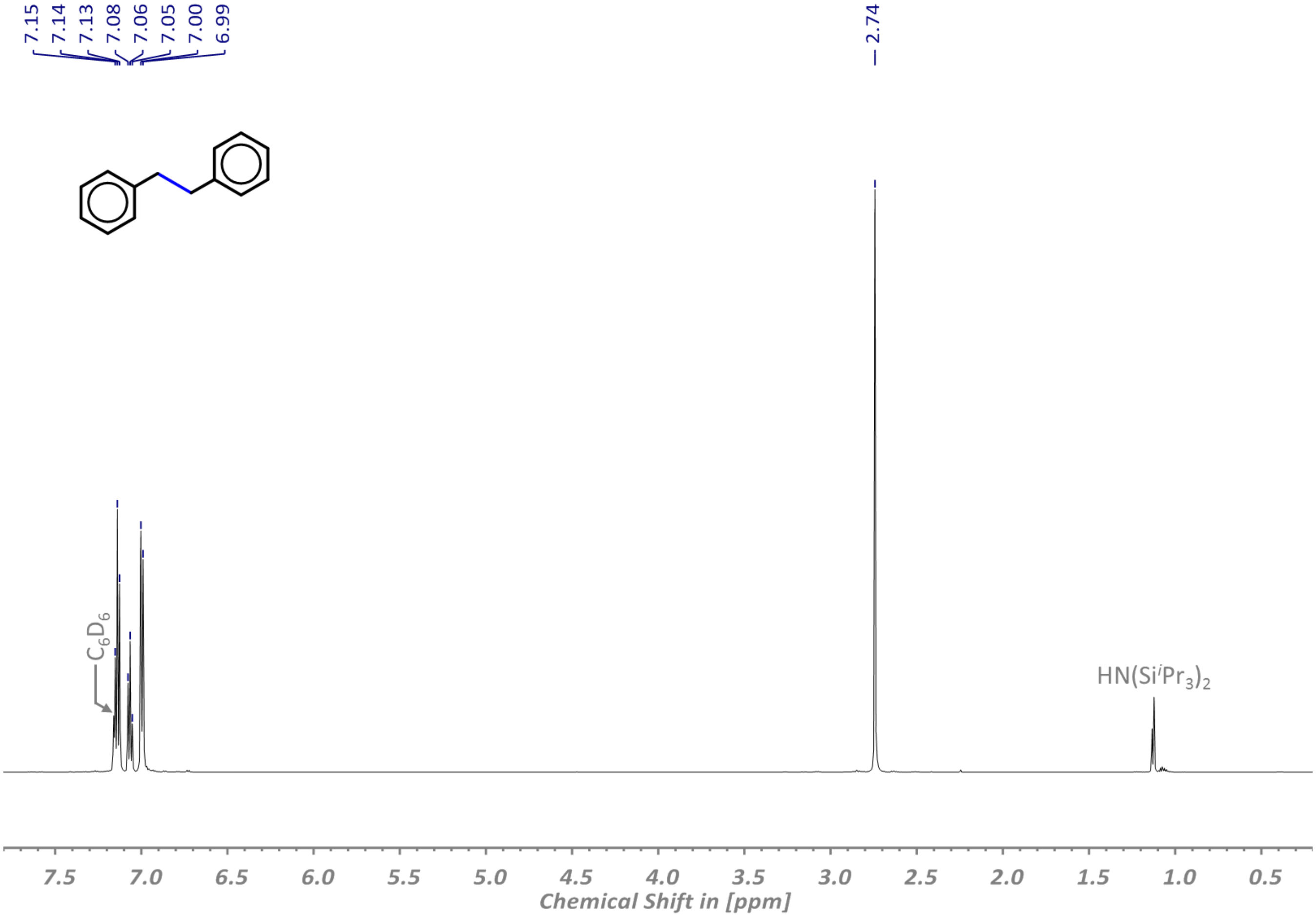

Figure S98. ^{1}H NMR spectrum (600 MHz, C_6D_6, 25 °C) of 1,2-diphenylethane after catalytic hydrogenation (1 h) of *cis*-stilbene with $Ba[N(Si^iPr_3)_2]_2$ (**1-Ba**) (1 mol%) and H_2 (6 bar) at 120 °C (Table S3, entry 16).

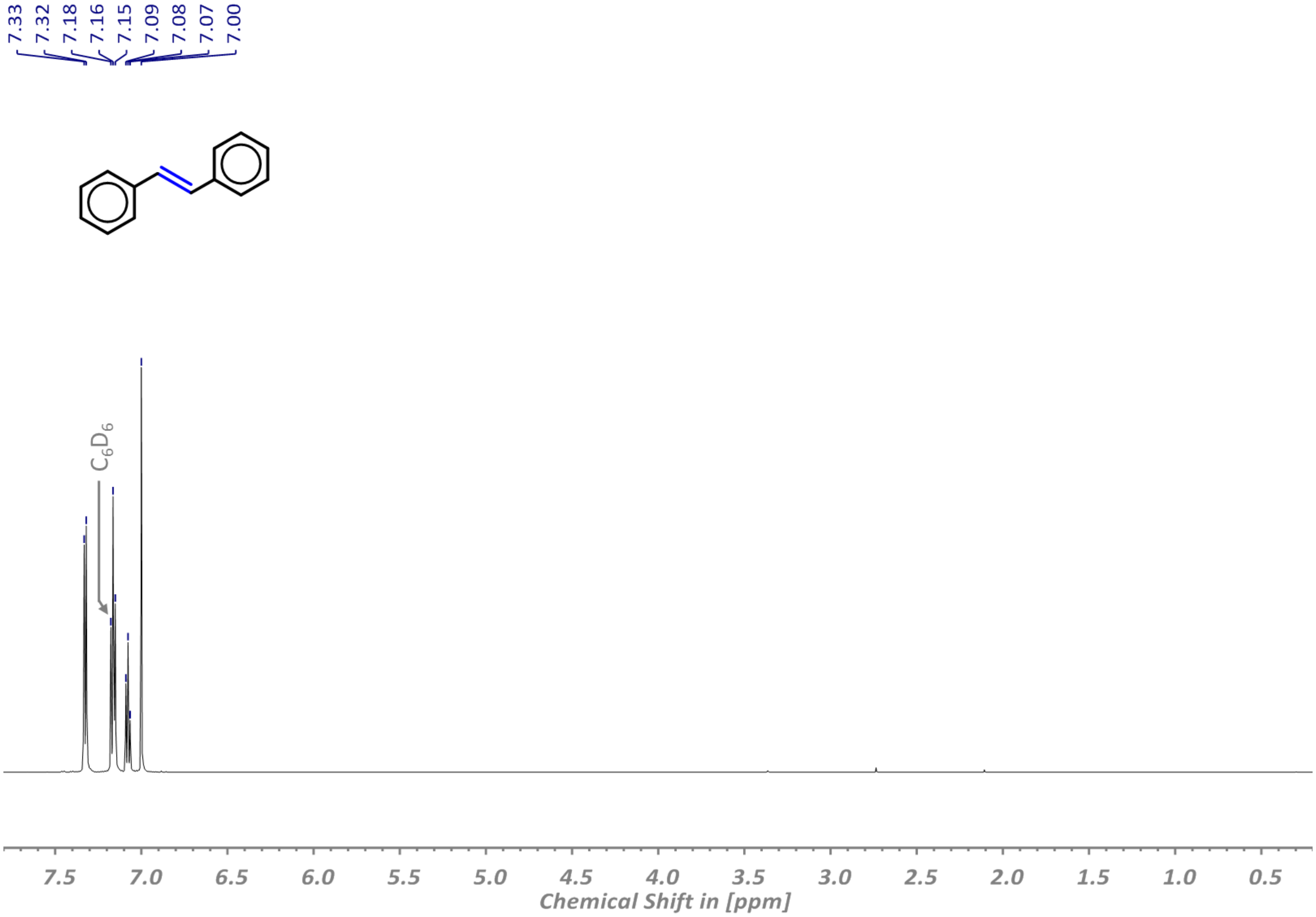

Figure S99. ^{1}H NMR spectrum (600 MHz, C_6D_6, 25 °C) of *trans*-stilbene.

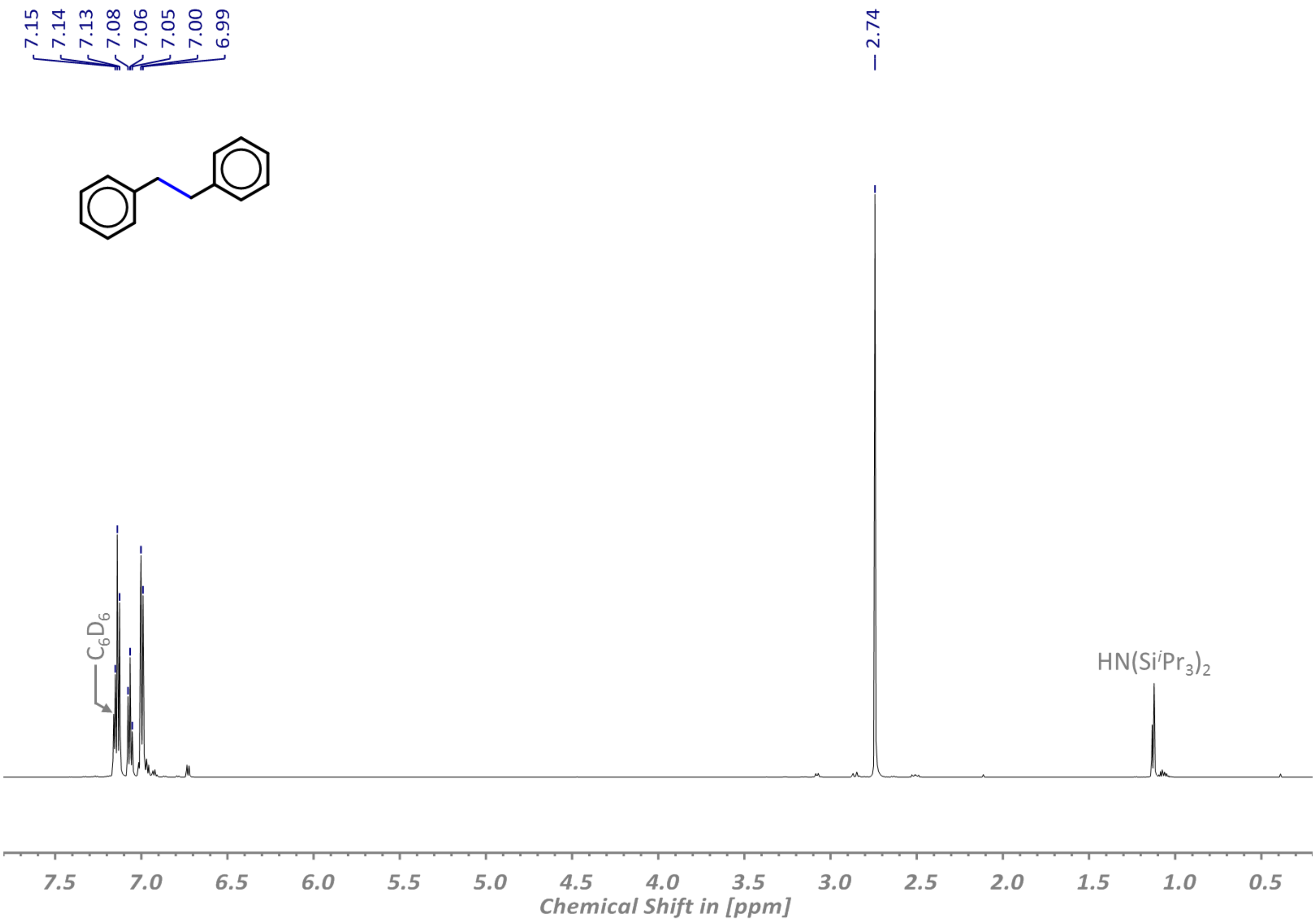

Figure S100. ^{1}H NMR spectrum (600 MHz, C_6D_6, 25 °C) of 1,2-diphenylethane after catalytic hydrogenation (1 h) of *trans*-stilbene with $Ba[N(Si^iPr_3)_2]_2$ (**1-Ba**) (1 mol%) and H_2 (6 bar) at 120 °C (Table S3, entry 17).

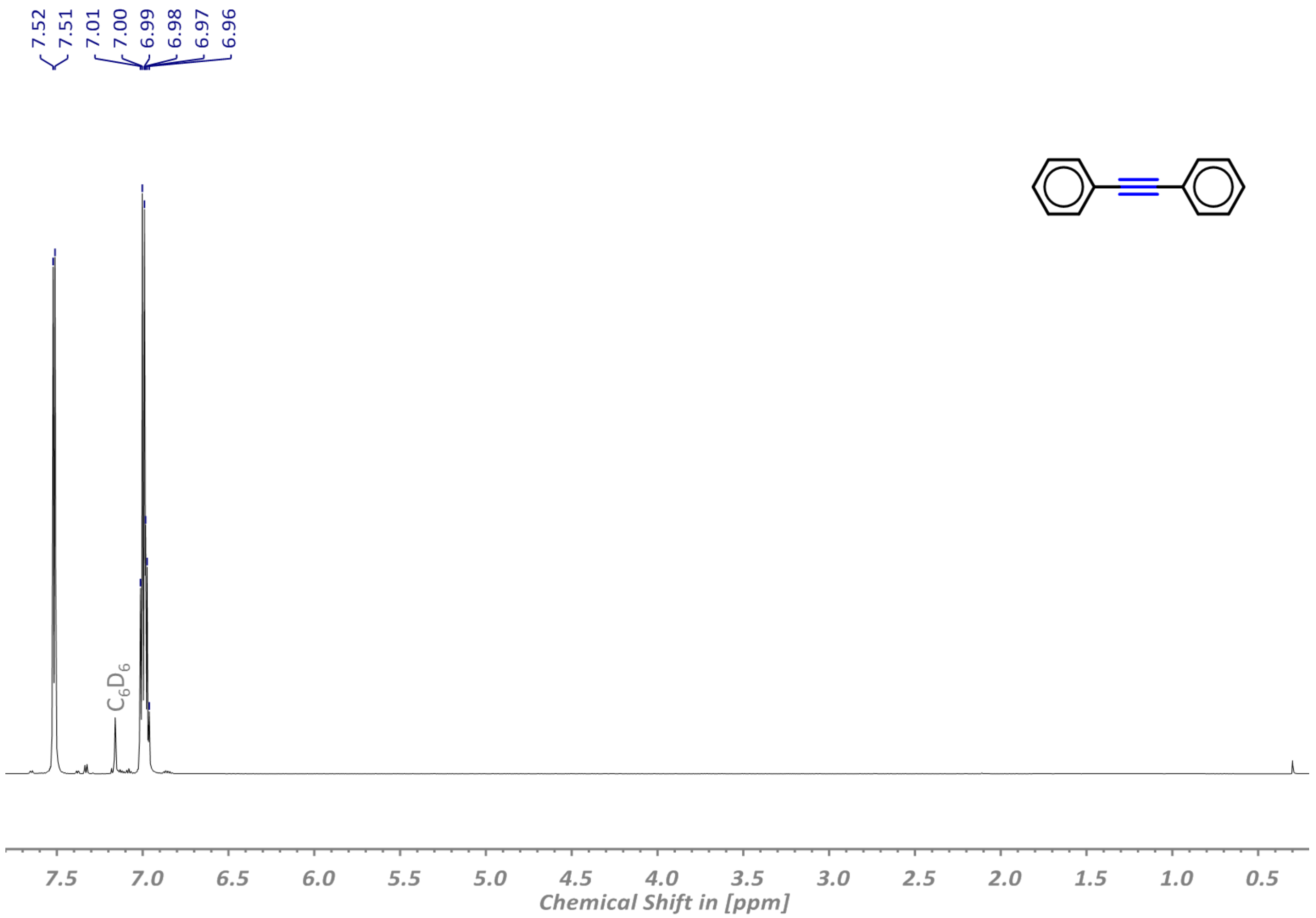

Figure S101. ^{1}H NMR spectrum (600 MHz, C_6D_6, 25 °C) of diphenylacetylene.

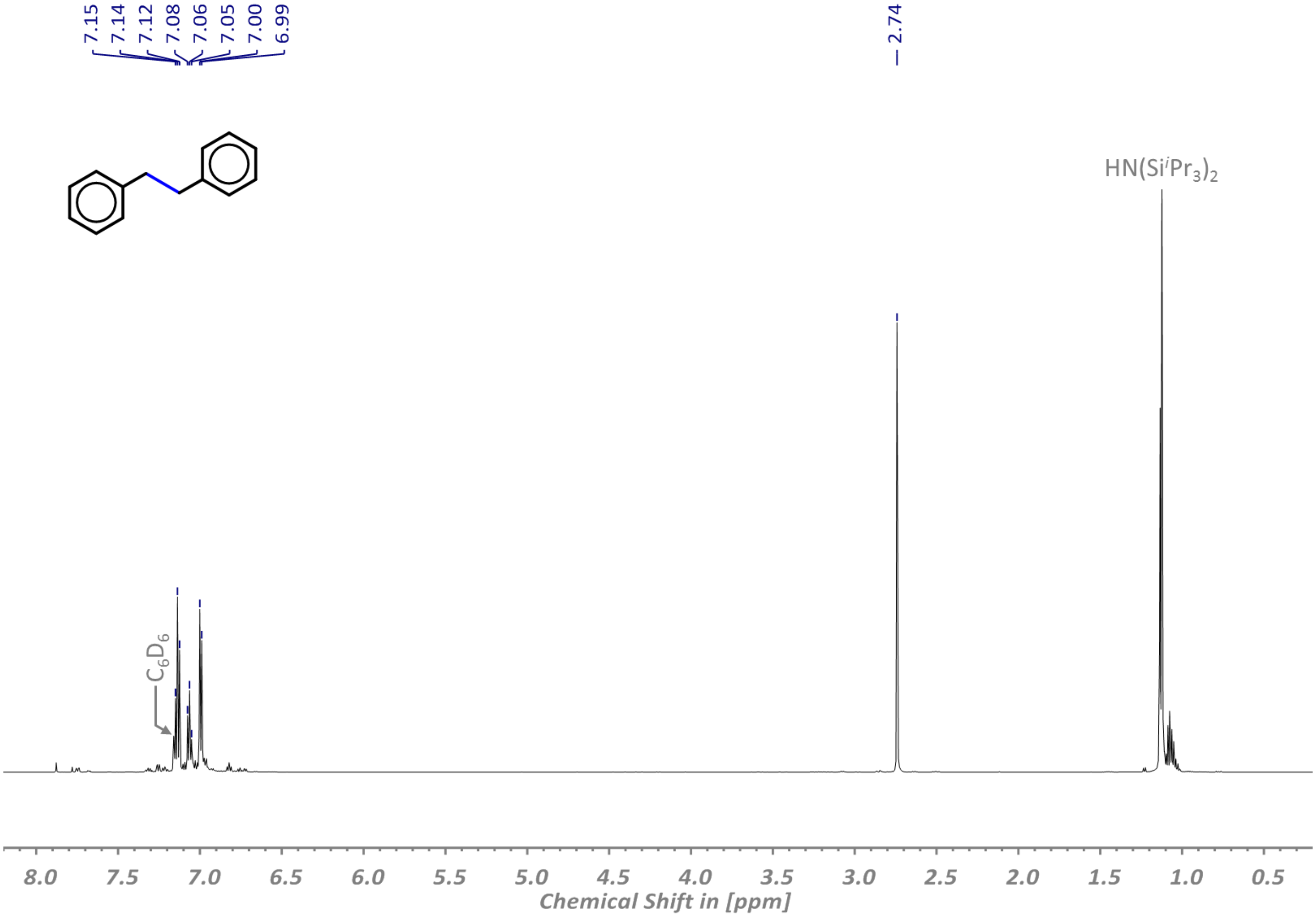

Figure S102. ^{1}H NMR spectrum (600 MHz, C_6D_6, 25 °C) of 1,2-diphenylethane after catalytic hydrogenation (1 h) of diphenylacetylene with Ba[N(Si^{i}Pr$_3$)$_2$]$_2$ (**1-Ba**) (10 mol%) and H_2 (6 bar) at 120 °C (Table S3, entry 18).

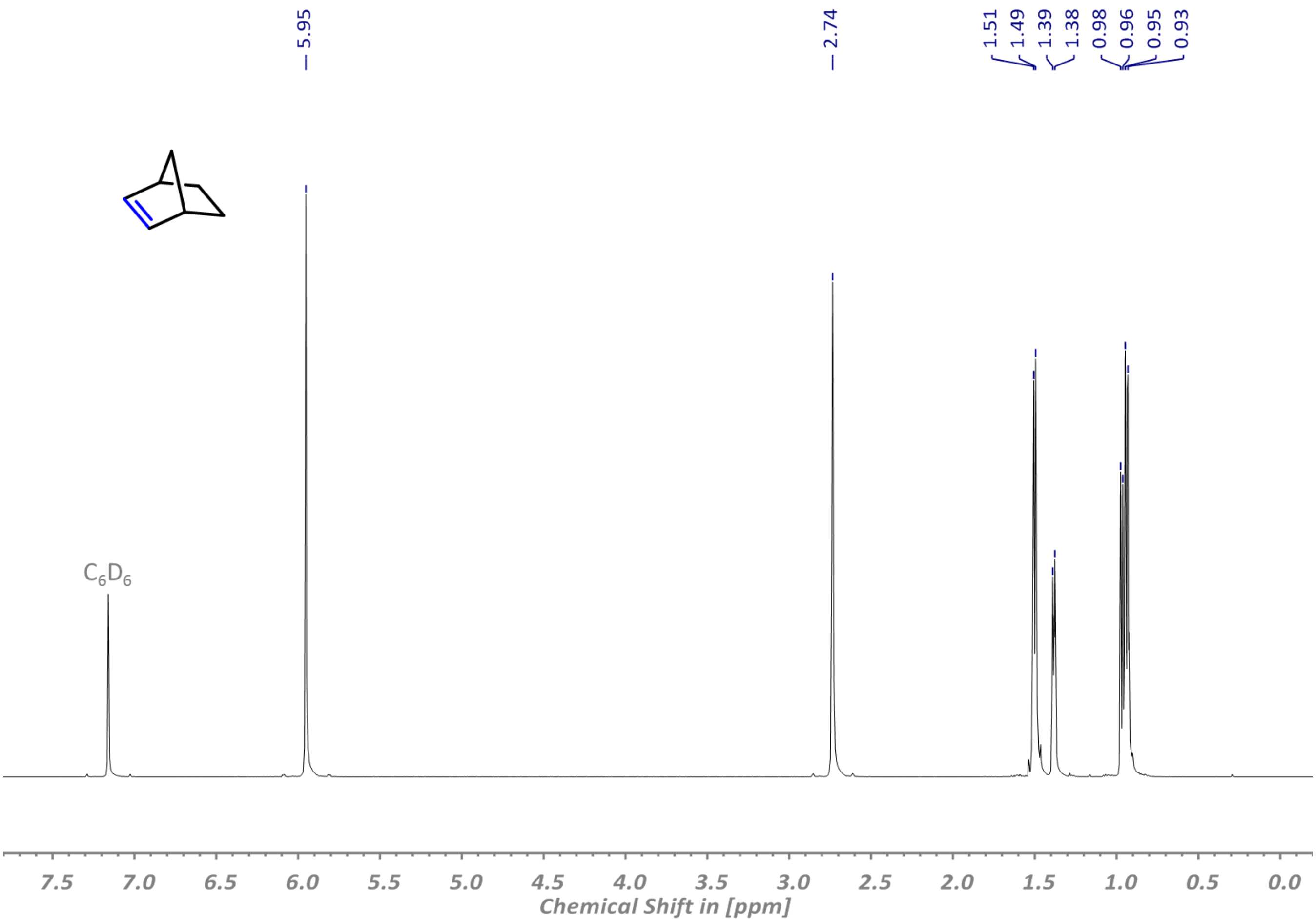

Figure S103. ^{1}H NMR spectrum (600 MHz, C_6D_6, 25 °C) of norbornene.

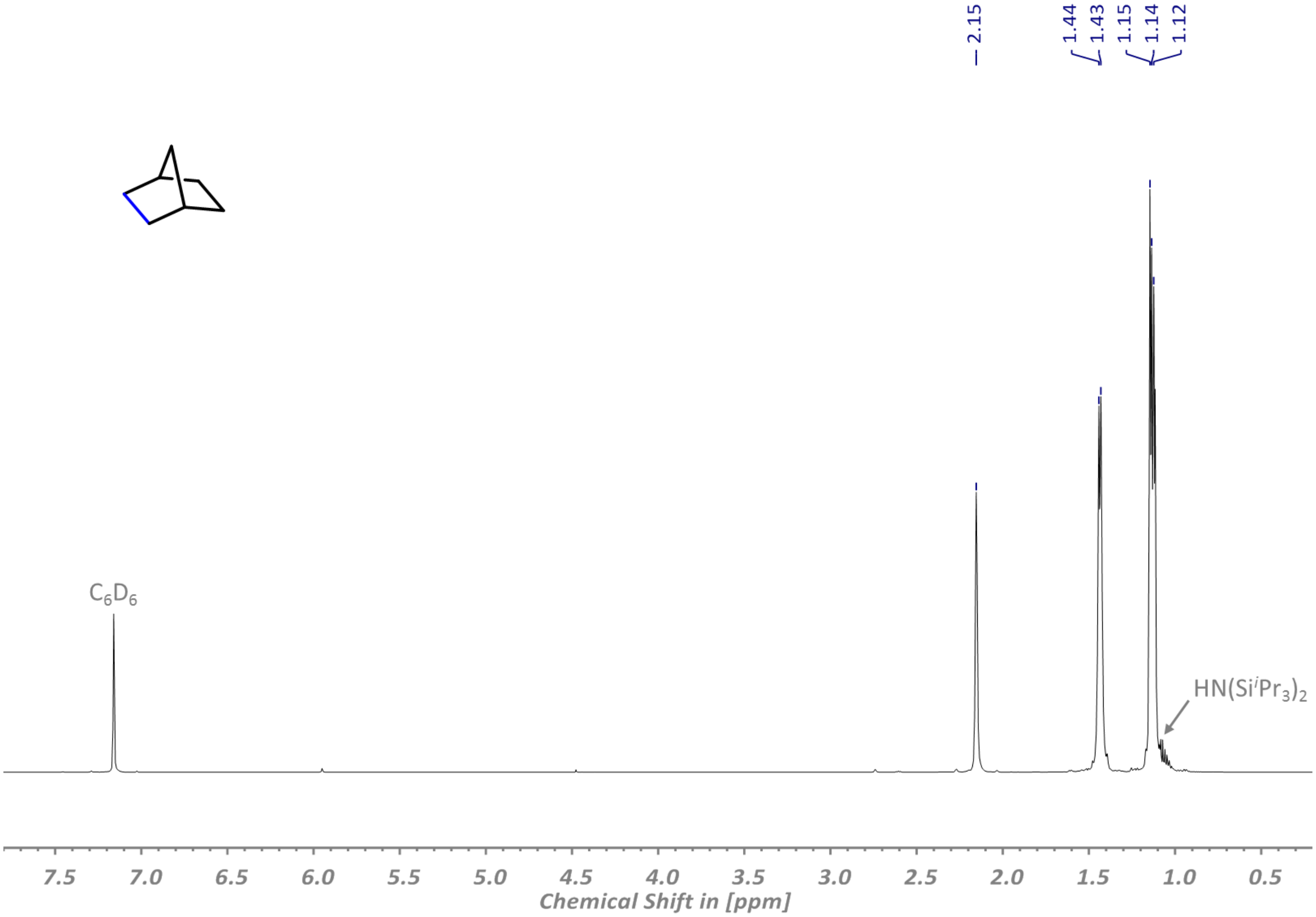

Figure S104. ^{1}H NMR spectrum (600 MHz, C_6D_6, 25 °C) of norbornane after catalytic hydrogenation (0.5 h) of norbornene (1 M) with Ba[N(Si^{i}Pr$_3$)$_2$]$_2$ (**1-Ba**) (1 mol%) and H_2 (6 bar) at 120 °C (Table S3, entry 19).

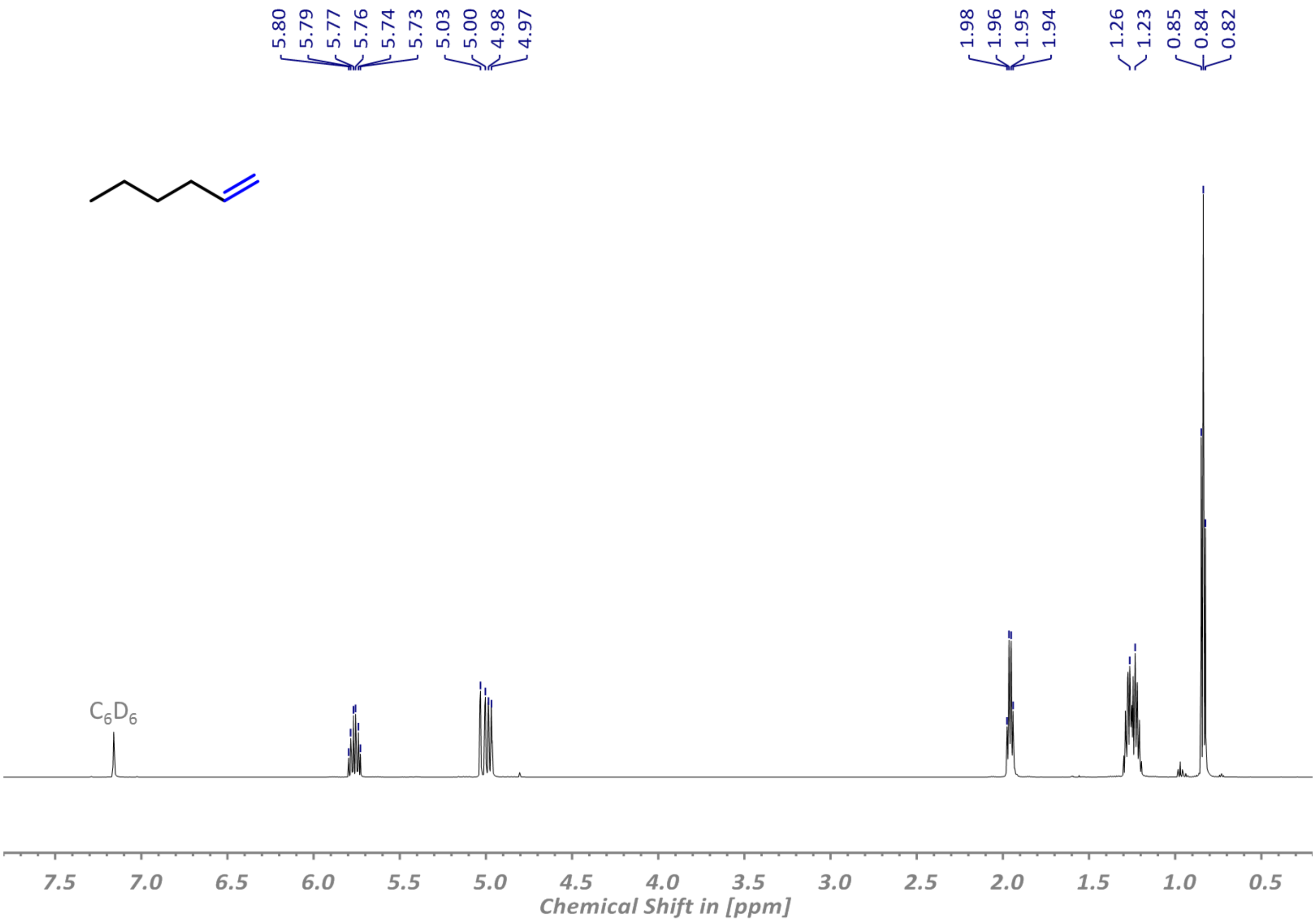

Figure S105. ^{1}H NMR spectrum (600 MHz, C_6D_6, 25 °C) of 1-hexene.

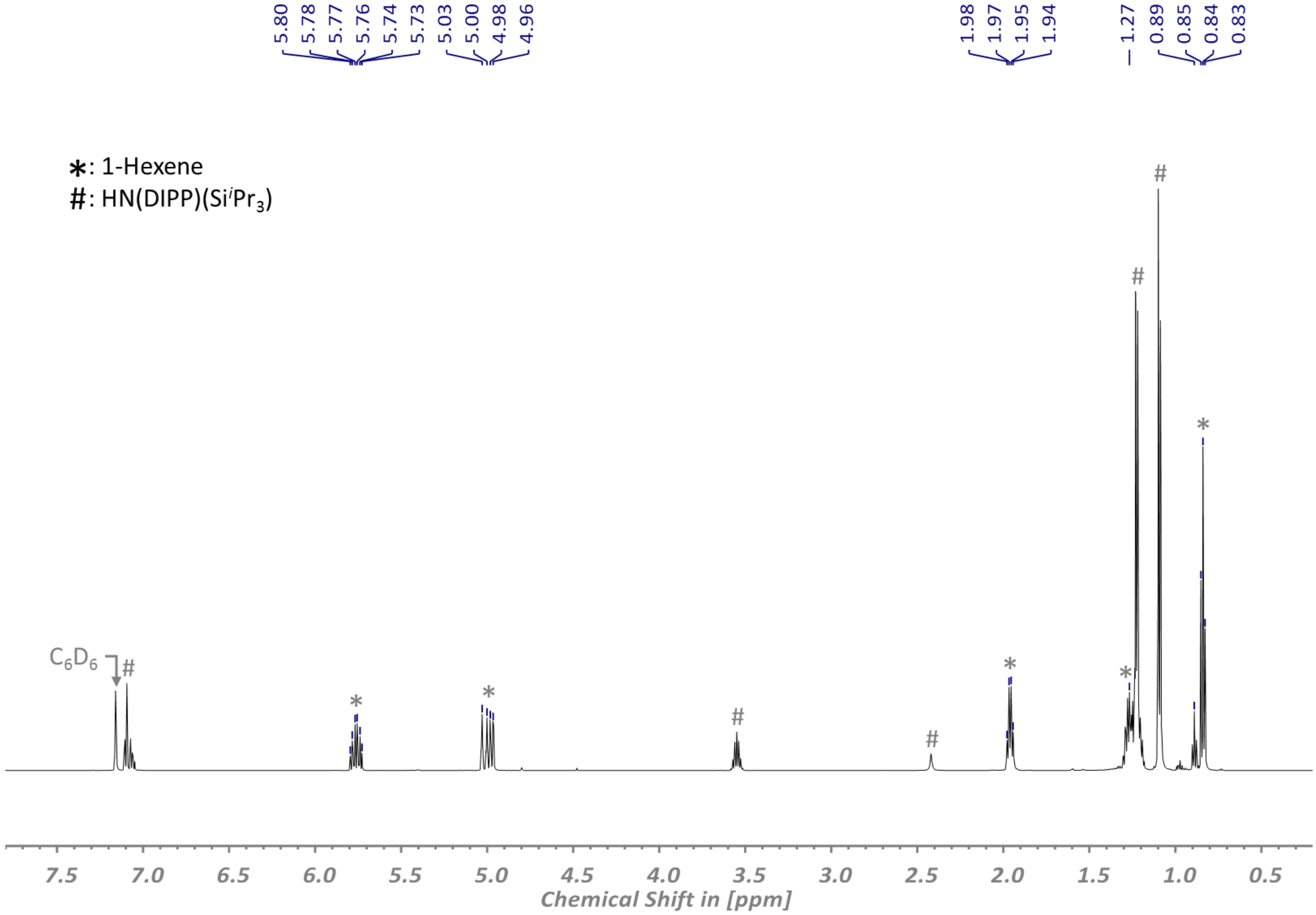

Figure S106. ^{1}H NMR spectrum (600 MHz, C_6D_6, 25 °C) after catalytic hydrogenation (24 h) of 1-hexene (∗) with Mg[N(DIPP)(Si^{i}Pr$_3$)]$_2$ (**2-Mg**) (10 mol%) and H_2 (6 bar) at 120 °C showing the formation of a stoichiometric amount of hexane. *Note:* # denotes HN(DIPP)(Si^{i}Pr$_3$) (Table S3, entry 20).

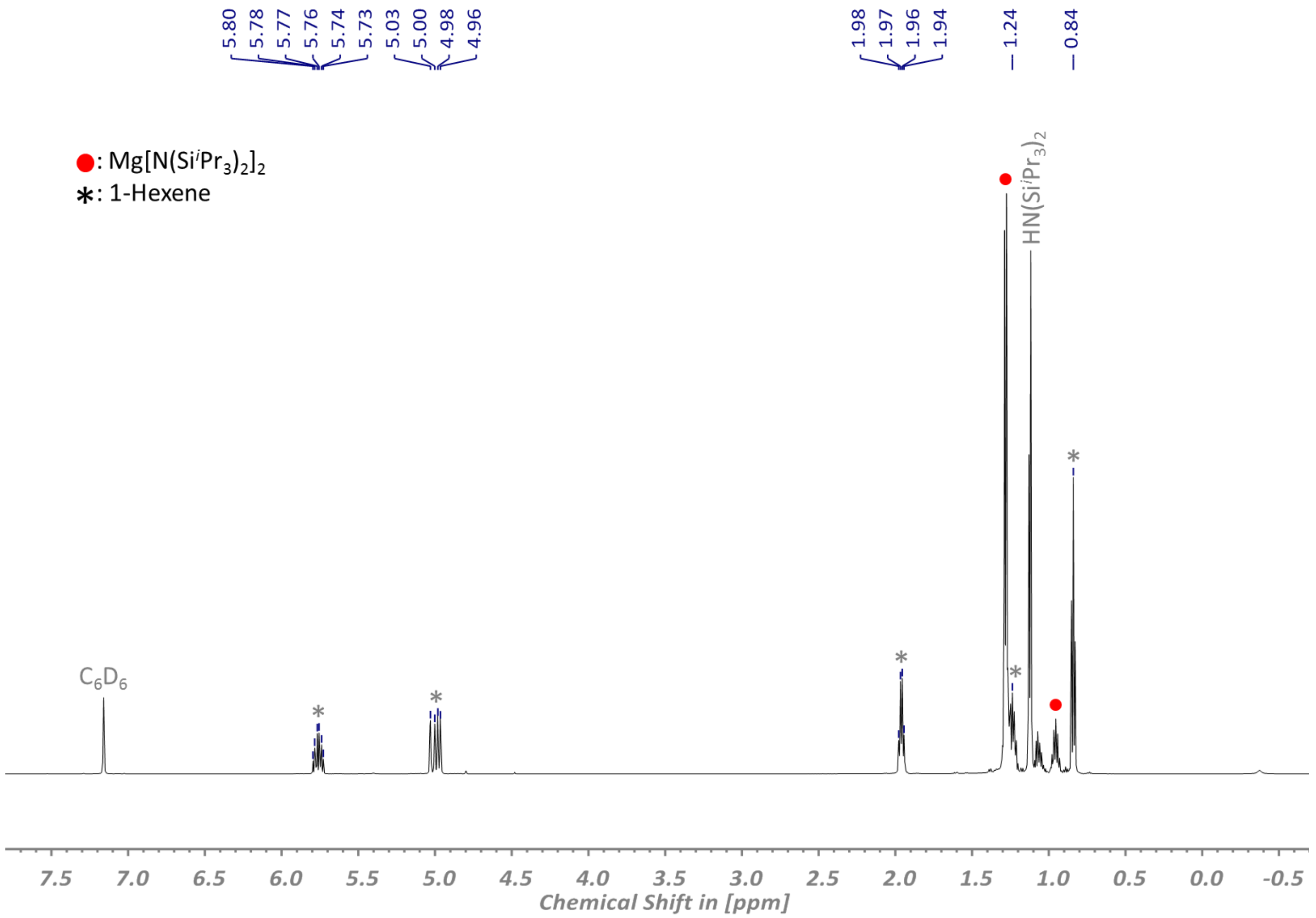

Figure S107. ^{1}H NMR spectrum (600 MHz, C_6D_6, 25 °C) after the reaction (24 h) of 1-hexene (*) with Mg[N(Si^{i}Pr$_3$)$_2$]$_2$ (**1-Mg**) (●) (10 mol%) and H_2 (6 bar) at 120 °C to give only a trace amount of hexane (Table S3, entry 21).

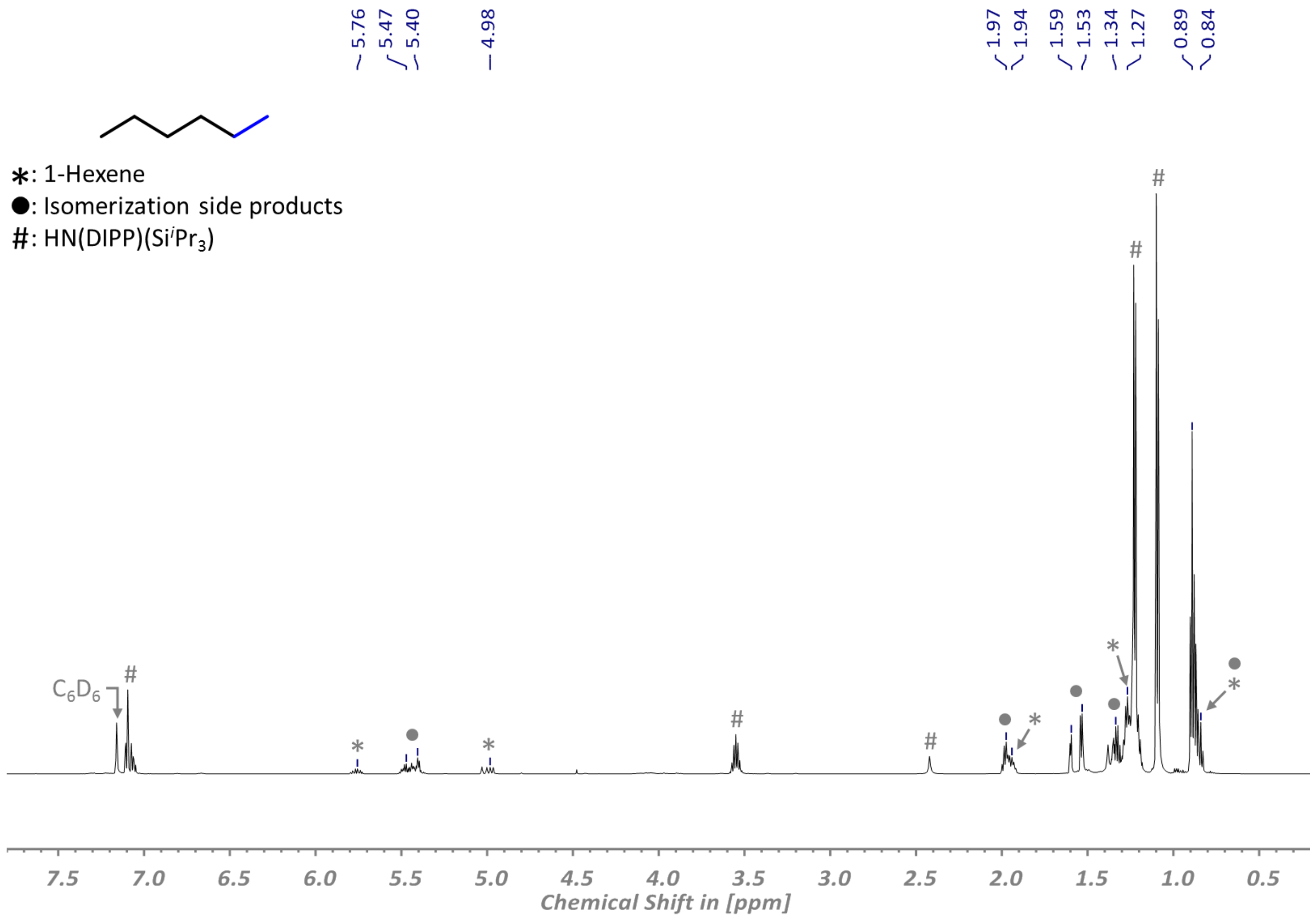

Figure S108. ^{1}H NMR spectrum (600 MHz, C_6D_6, 25 °C) after catalytic hydrogenation (24 h) of 1-hexene (∗) with Ca[N(DIPP)(Si^{i}Pr$_3$)]$_2$ (**2-Ca**) (10 mol%) and H_2 (6 bar) at 120 °C showing the formation of hexane and isomerization side products (●), which consist of 2-hexene (28%) and 3-hexene (14%) as determined by GC/MS analysis. *Note:* # denotes HN(DIPP)(Si^{i}Pr$_3$) (Table S3, entry 22).

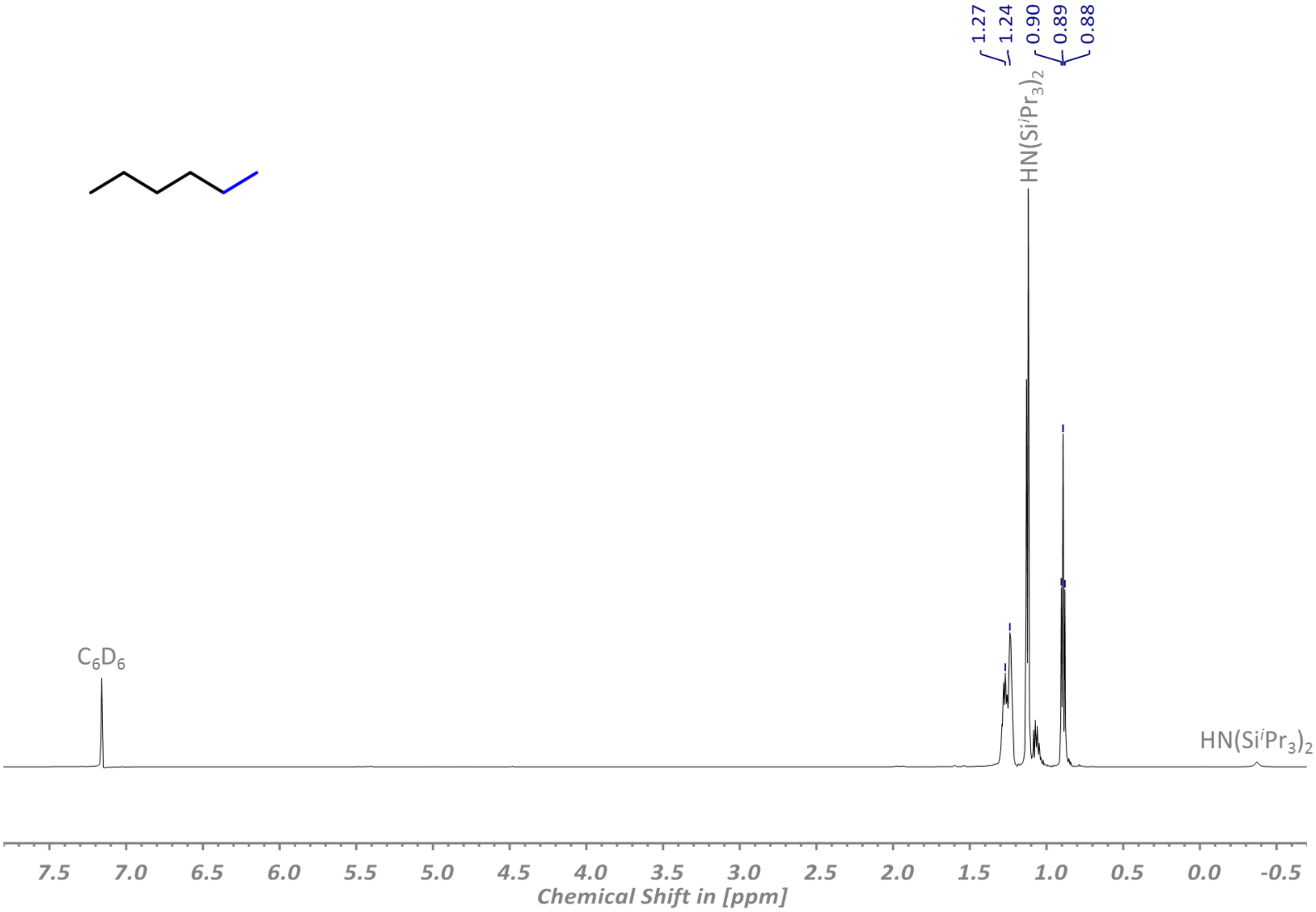

Figure S109. ^1H NMR spectrum (600 MHz, C_6D_6, 25 °C) of hexane after catalytic hydrogenation (3 h) of 1-hexene with Ca[N(Si^{i}Pr$_3$)$_2$]$_2$ (**1-Ca**) (10 mol%) and H_2 (6 bar) at 120 °C (Table S3, entry 23).

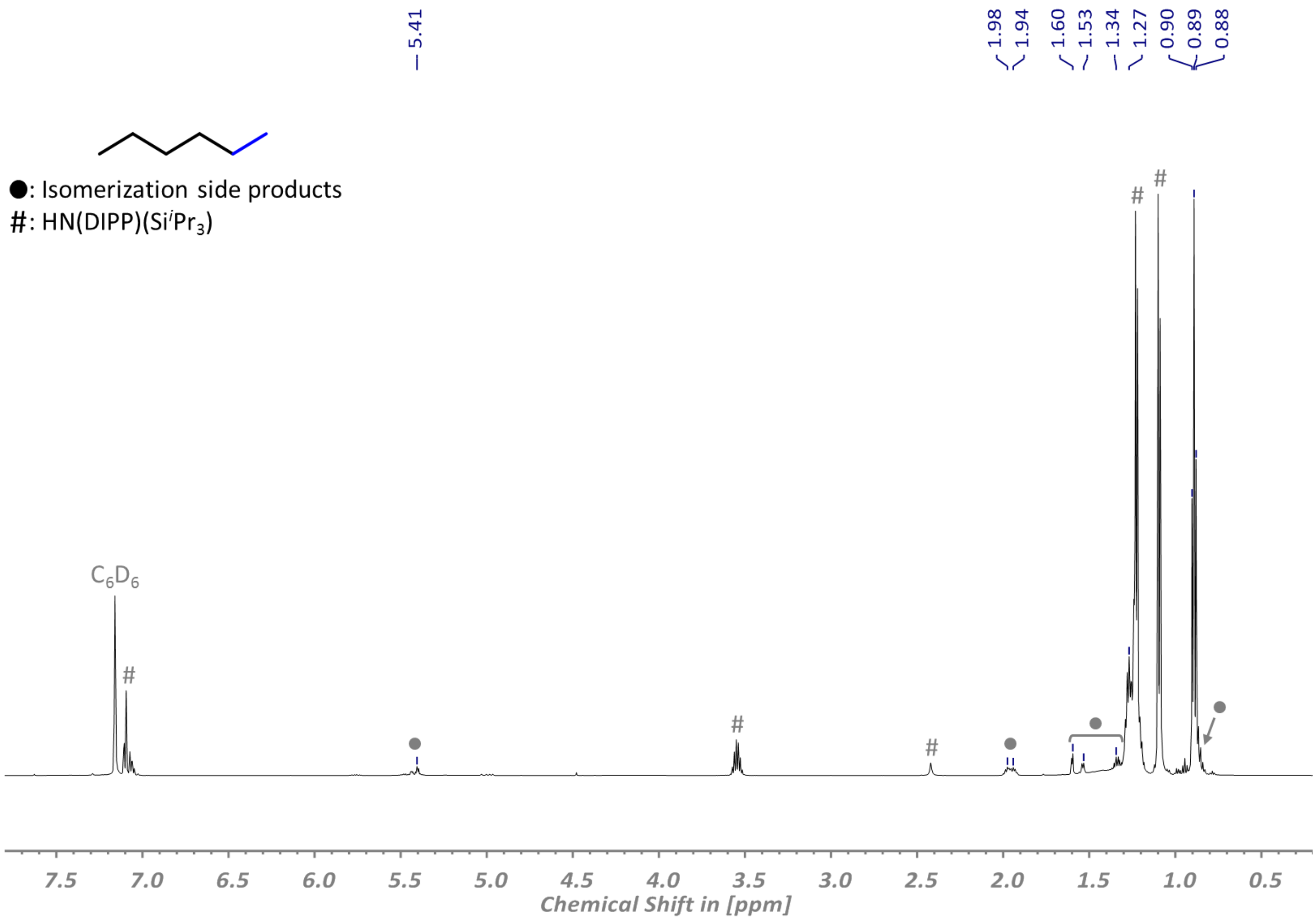

Figure S110. ^{1}H NMR spectrum (600 MHz, C_6D_6, 25 °C) after catalytic hydrogenation (24 h) of 1-hexene (∗) with Sr[N(DIPP)(Si^{i}Pr$_3$)]$_2$ (**2-Sr**) (10 mol%) and H_2 (6 bar) at 120 °C showing the formation of hexane and internal hexene isomers as side products (●). *Note:* # denotes HN(DIPP)(Si^{i}Pr$_3$) (Table S3, entry 24).

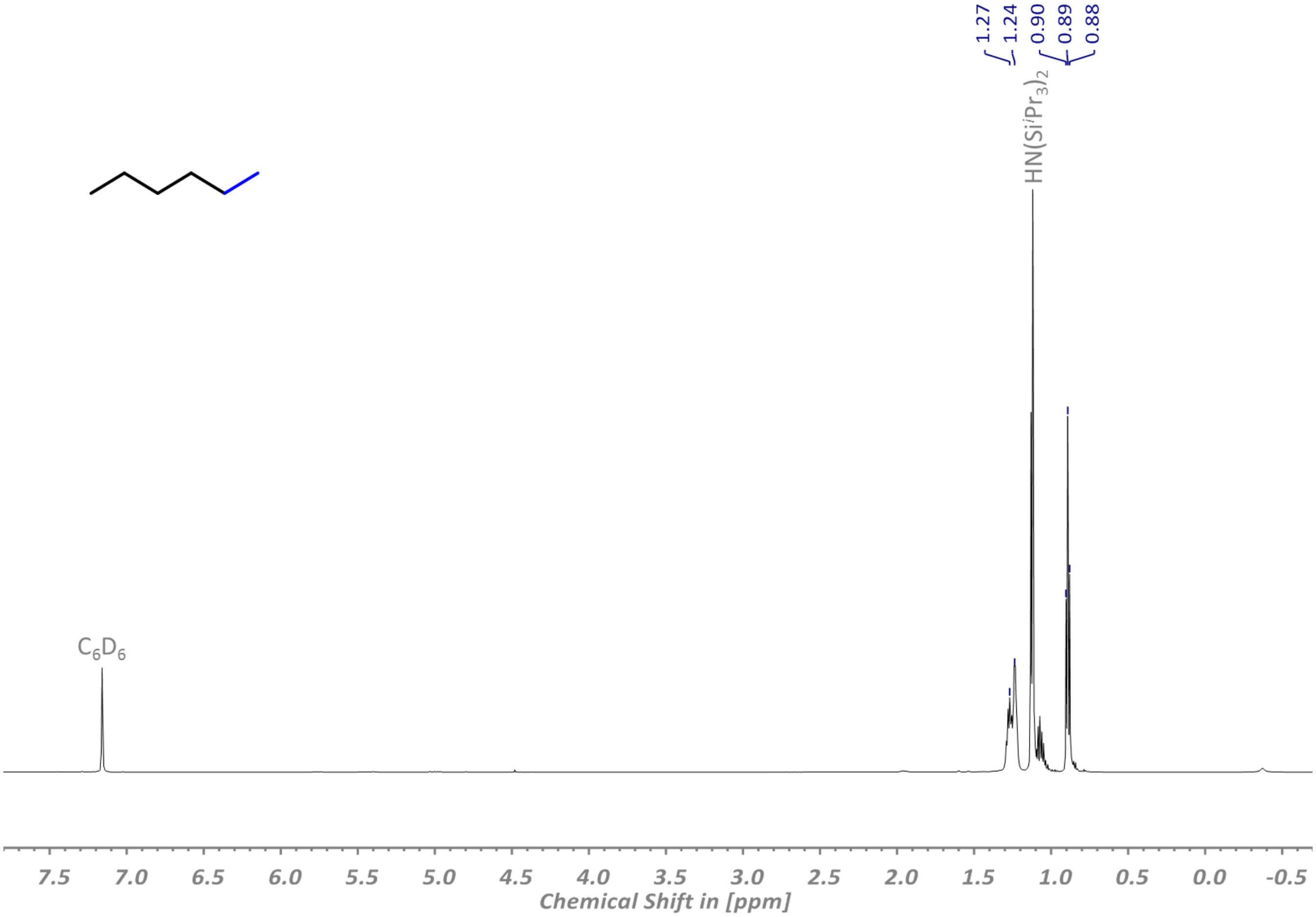

Figure S111. ^{1}H NMR spectrum (600 MHz, C_6D_6, 25 °C) of hexane after catalytic hydrogenation (0.5 h) of 1-hexene with Sr[N(Si^{i}Pr$_3$)$_2$]$_2$ (**1-Sr**) (10 mol%) and H_2 (6 bar) at 120 °C (Table S3, entry 25).

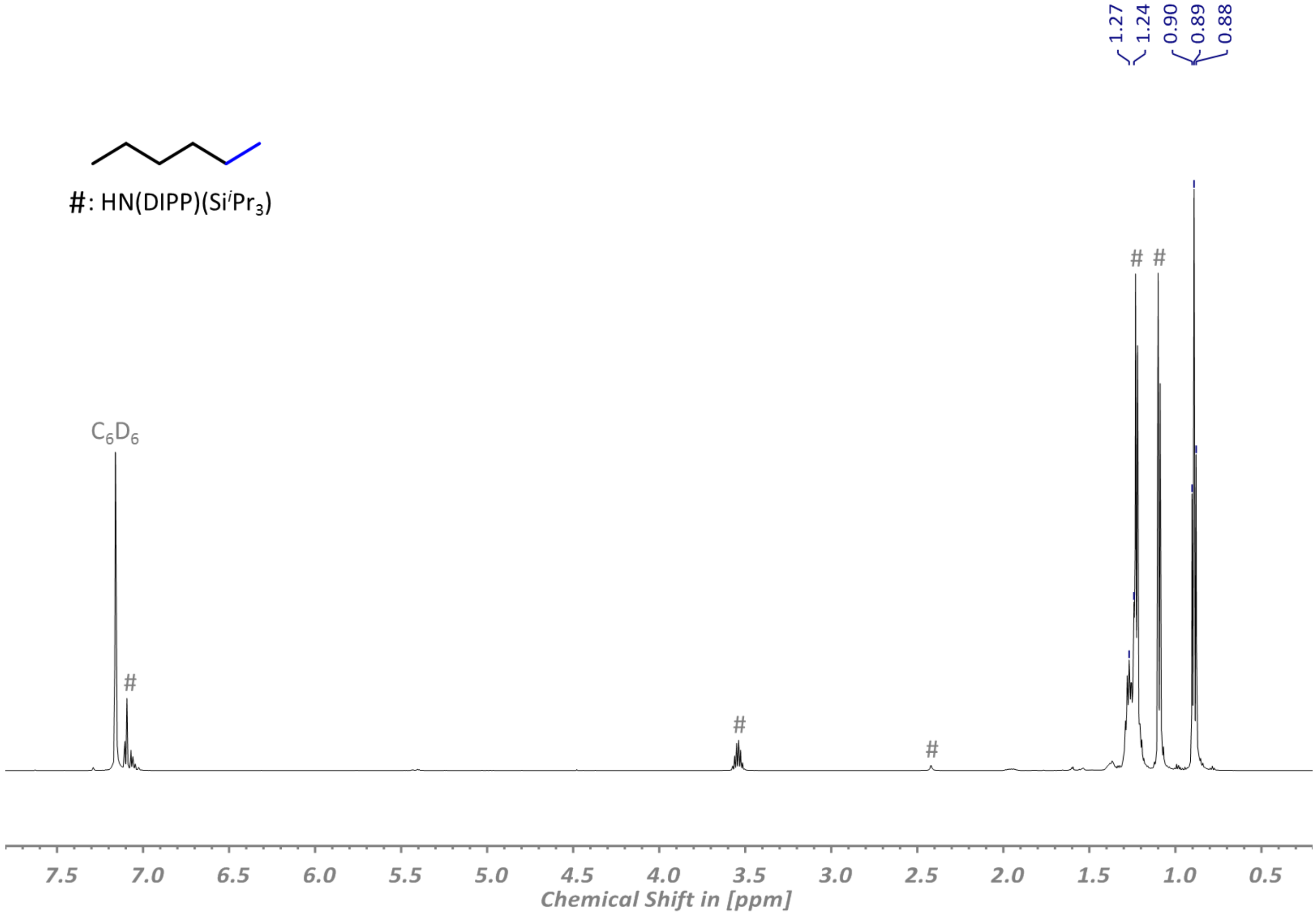

Figure S112. ^{1}H NMR spectrum (600 MHz, C_6D_6, 25 °C) of hexane after catalytic hydrogenation (24 h) of 1-hexene with Ba[N(DIPP)(Si^{i}Pr$_3$)]$_2$ (**2-Ba**) (10 mol%) and H_2 (6 bar) at 120 °C. *Note:* # denotes HN(DIPP)(Si^{i}Pr$_3$) (Table S3, entry 26).

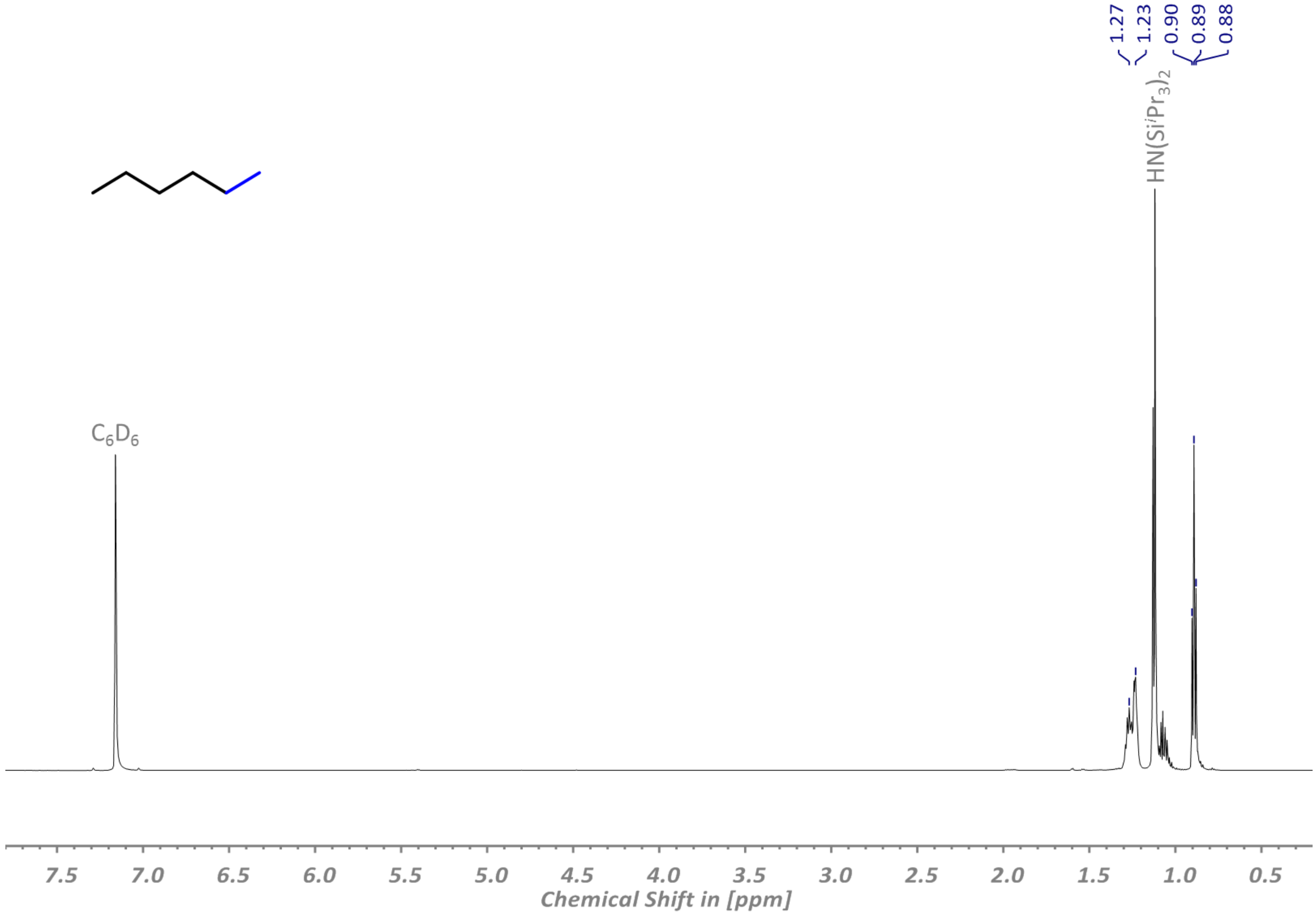

Figure S113. ^{1}H NMR spectrum (600 MHz, C_6D_6, 25 °C) of hexane after catalytic hydrogenation (0.5 h) of 1-hexene with Ba[N(Si^{i}Pr$_3$)$_2$]$_2$ (**1-Ba**) (10 mol%) and H_2 (6 bar) at 120 °C (Table S3, entry 27).

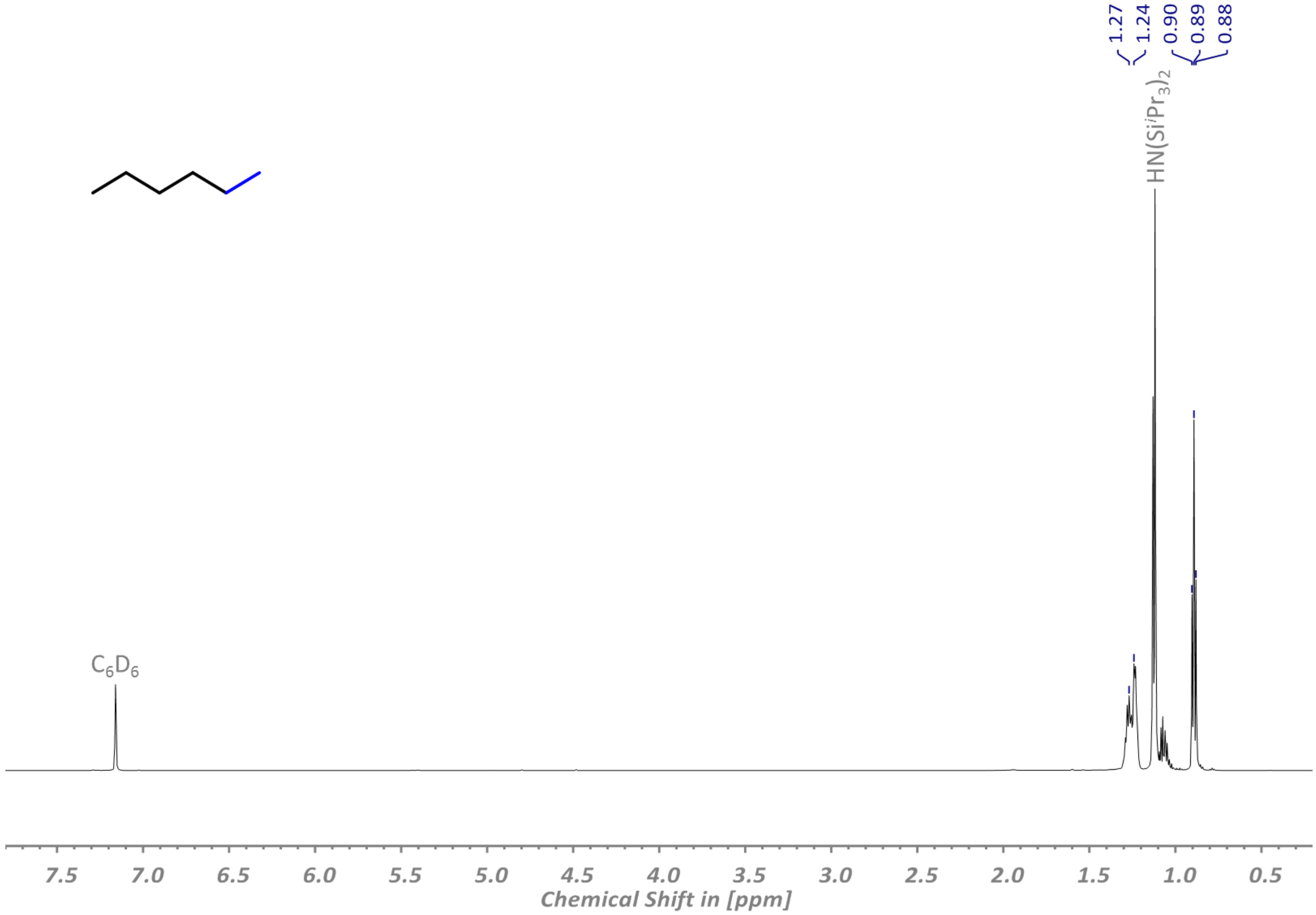

Figure S114. ^{1}H NMR spectrum (600 MHz, C_6D_6, 25 °C) of hexane after catalytic hydrogenation (6 h) of 1-hexene with Ba[N(Si^{i}Pr$_3$)$_2$]$_2$ (**1-Ba**) (10 mol%) and H_2 (6 bar) at 60 °C (Table S3, entry 28).

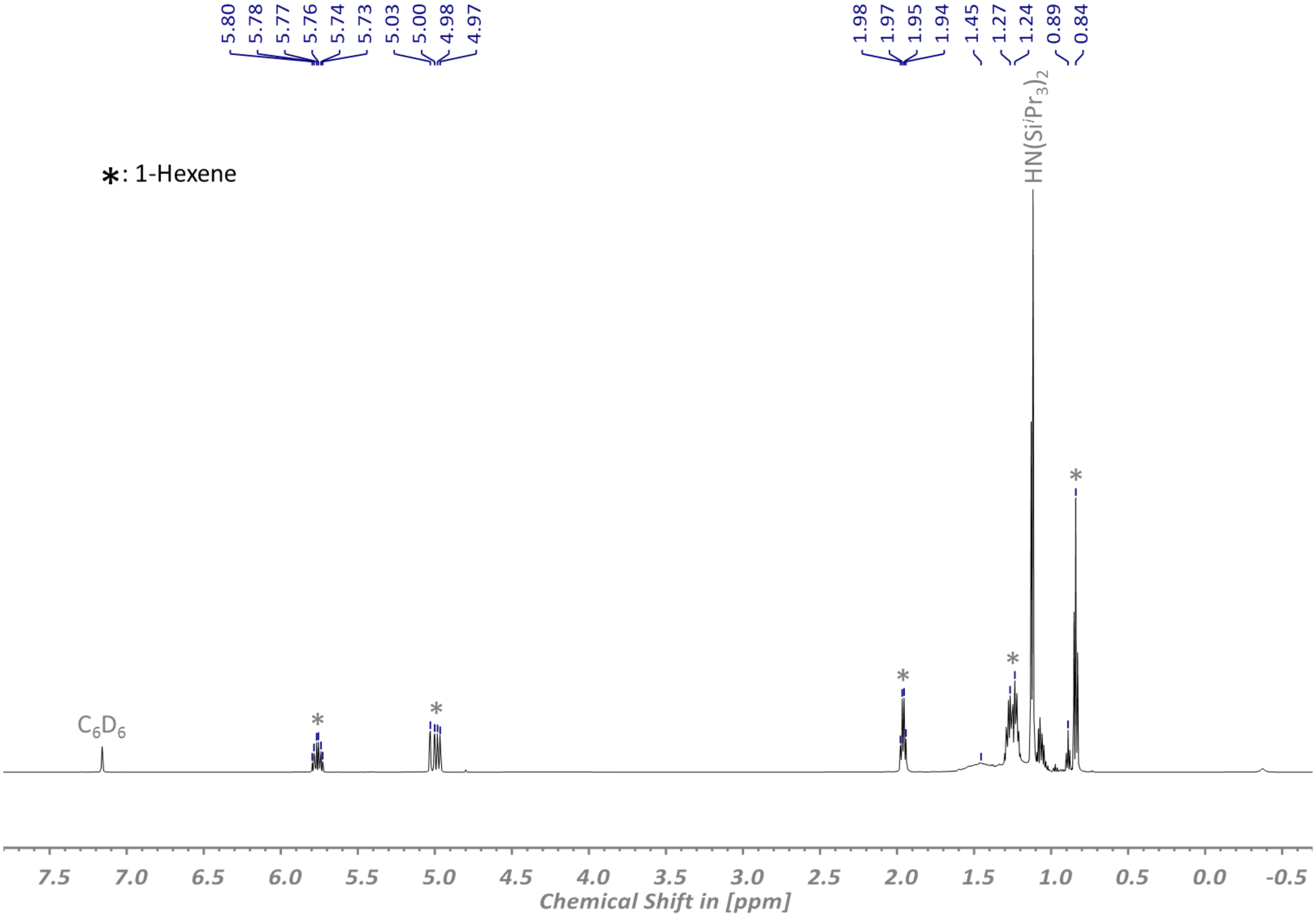

Figure S115. ^{1}H NMR spectrum (600 MHz, C_6D_6, 25 °C) after catalytic hydrogenation (24 h) of 1-hexene (∗) with Ba[N(Si^{i}Pr$_3$)$_2$]$_2$ (**1-Ba**) (10 mol%) and H_2 (6 bar) at 25 °C showing the stoichiometric formation of hexane (Table S3, entry 29).

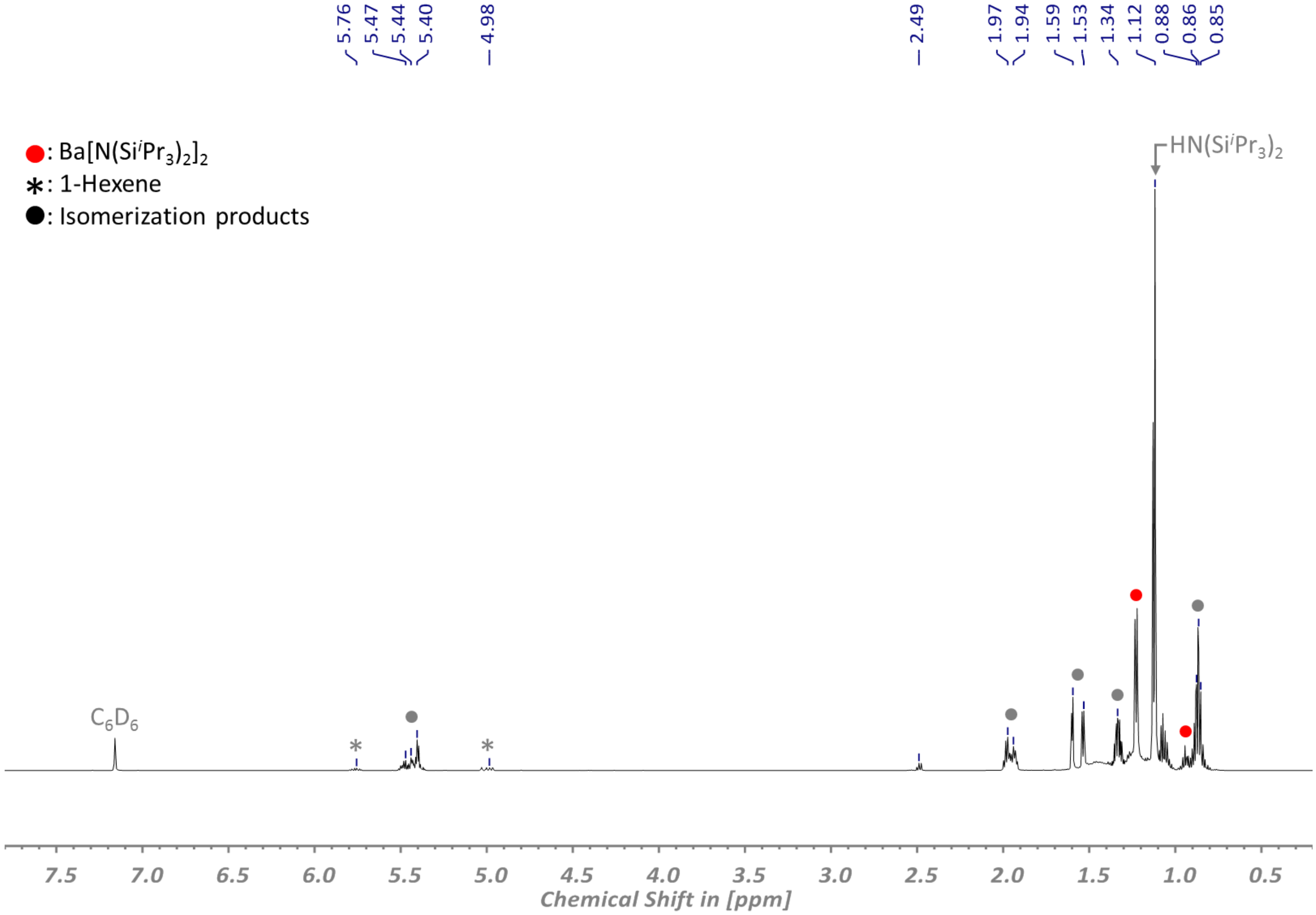

Figure S116. ^{1}H NMR spectrum (600 MHz, C_6D_6, 25 °C) after catalytic hydrogenation (24 h) of 1-hexene (∗) with Ba[N(Si^{i}Pr$_3$)$_2$]$_2$ (**1-Ba**) (●) (10 mol%) and H_2 (1 bar) at 120 °C showing the formation of a stoichiometric amount of hexane and substantial quantities of isomerization side products (●) (Table S3, entry 30).

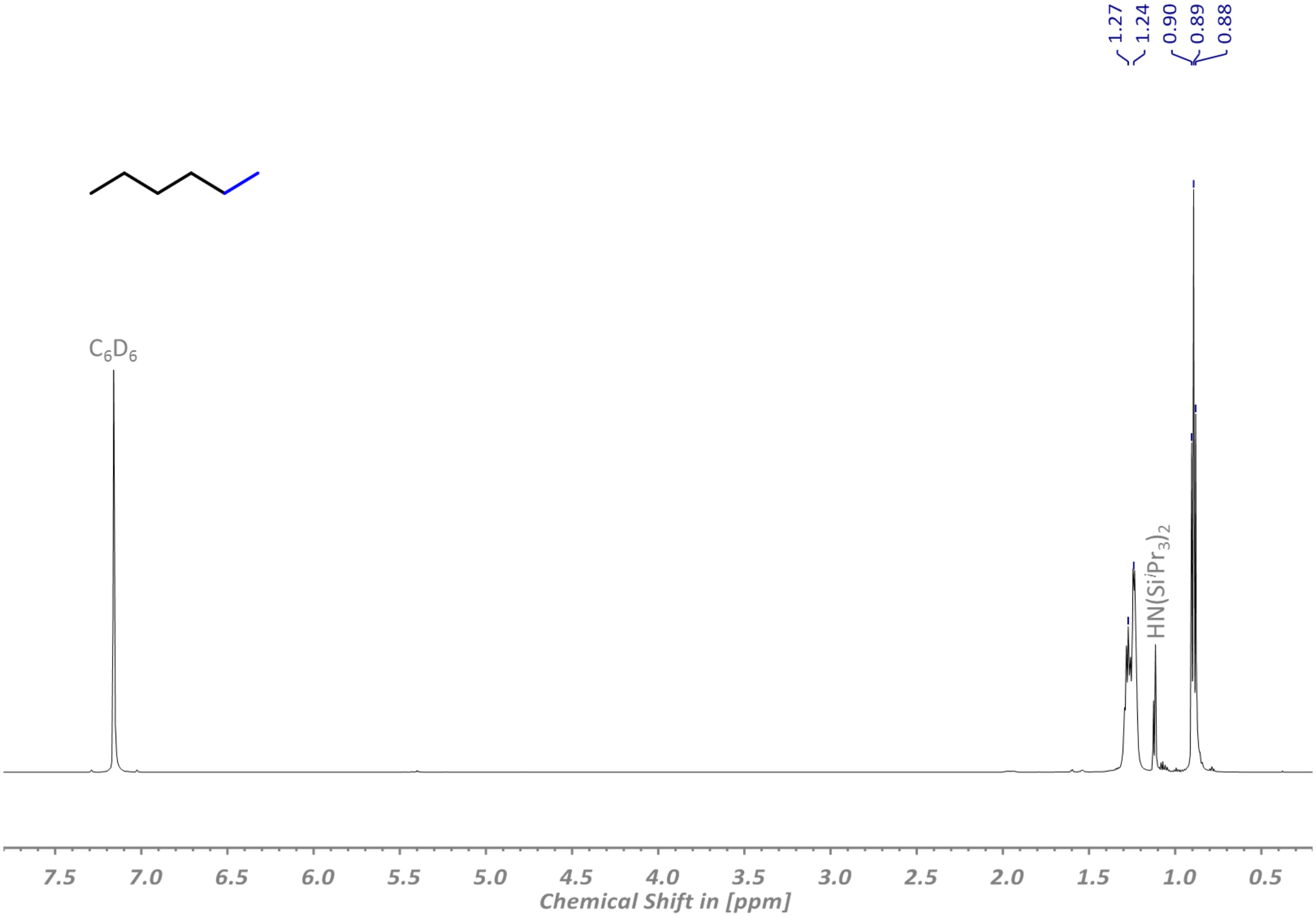

Figure S117. ^{1}H NMR spectrum (600 MHz, C_6D_6, 25 °C) of hexane after catalytic hydrogenation (4 h) of 1-hexene (1 M) with $Ba[N(Si^iPr_3)_2]_2$ (**1-Ba**) (1 mol%) and H_2 (6 bar) at 120 °C (Table S3, entry 31).

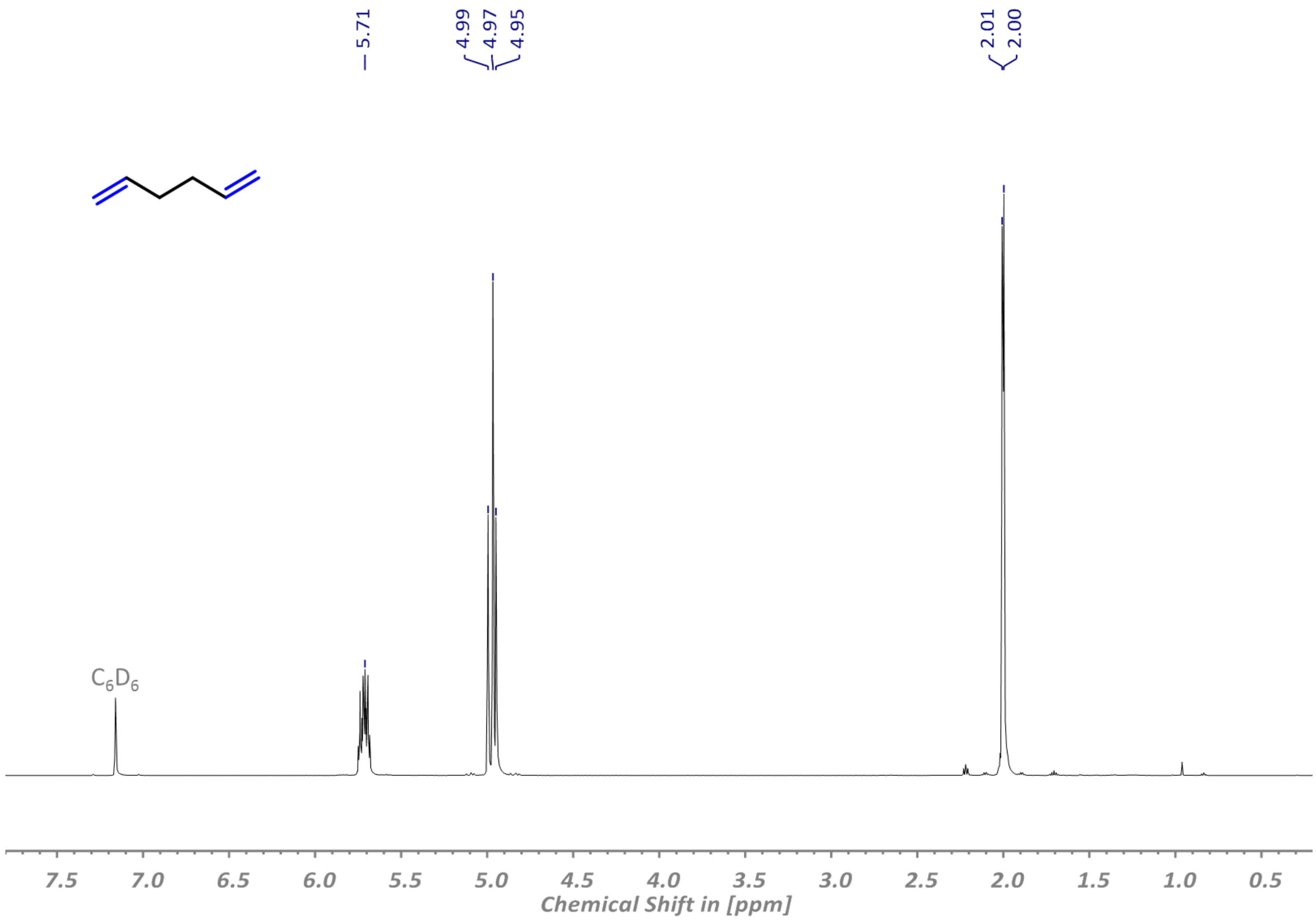

Figure S118. ^{1}H NMR spectrum (600 MHz, C_6D_6, 25 °C) of 1,5-hexadiene.

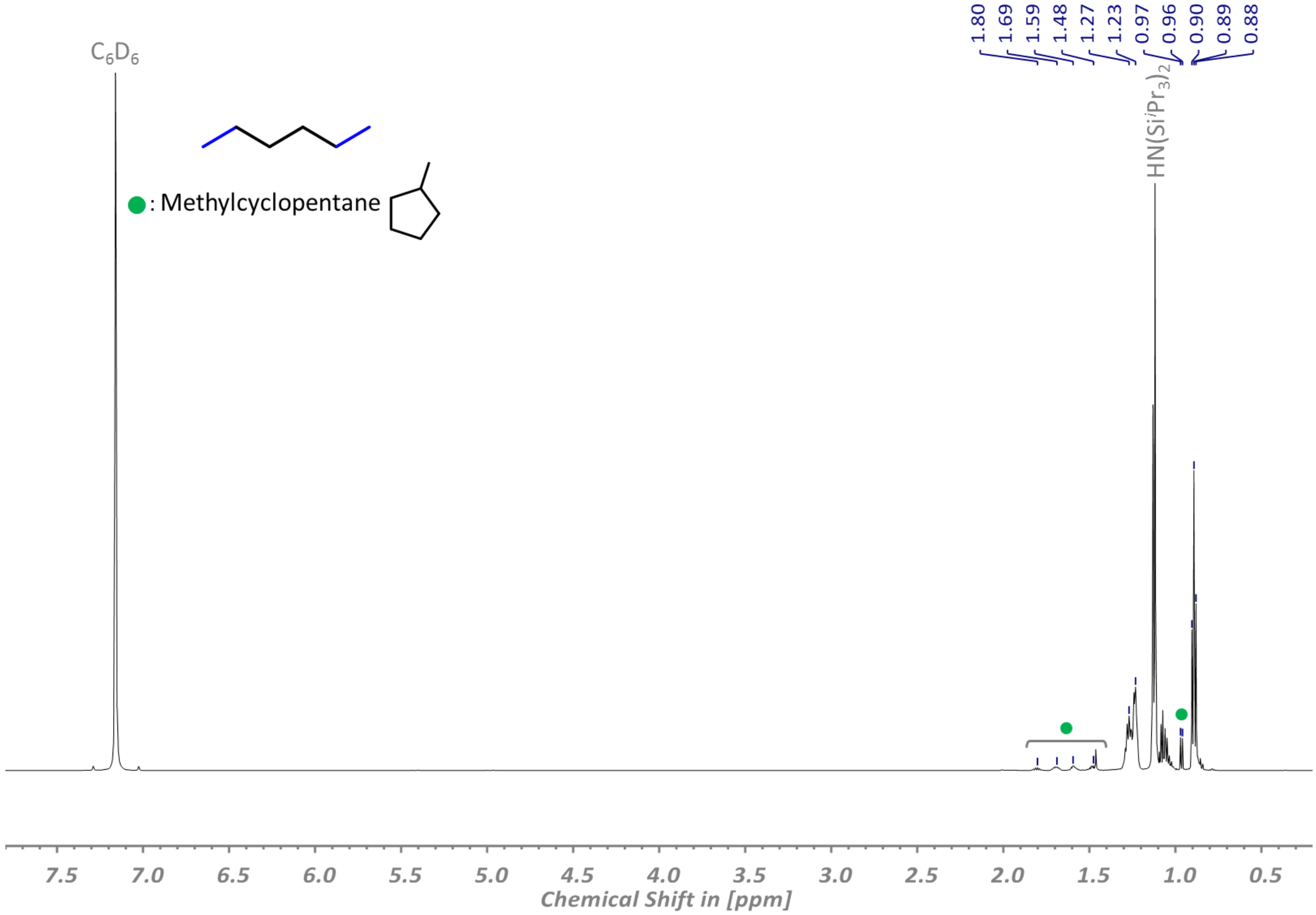

Figure S119. ^{1}H NMR spectrum (600 MHz, C_6D_6, 25 °C) after catalytic hydrogenation (2 h) of 1,5-hexadiene with Ba[N(Si^{i}Pr$_3$)$_2$]$_2$ (**1-Ba**) (10 mol%) and H_2 (6 bar) at 120 °C showing the formation of hexane and additionally methylcyclopentane (●) as product of an intramolecular cyclization side reaction followed by hydrogenation (Table S3, entry 32).

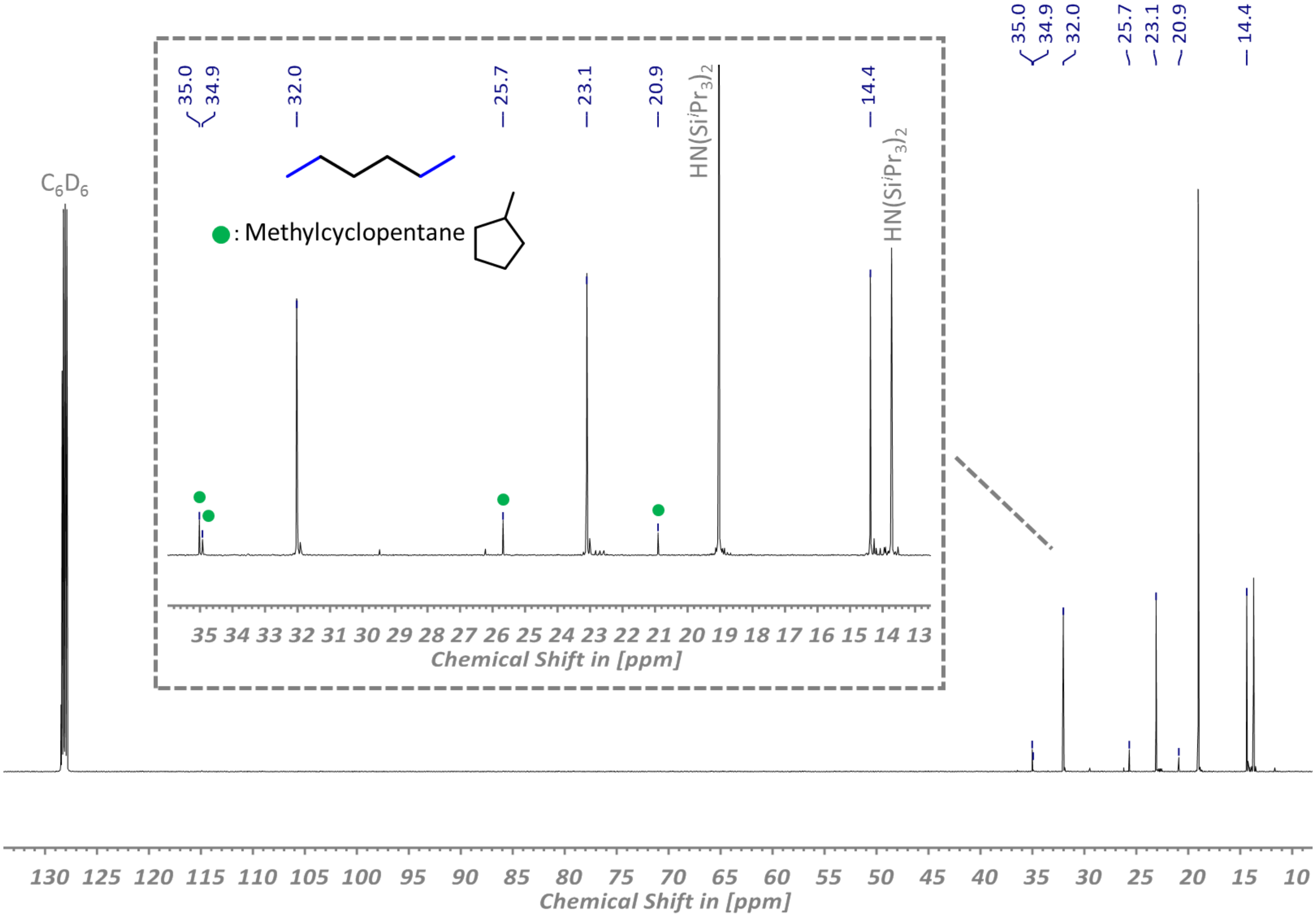

Figure S120. $^{13}C\{^1H\}$ NMR spectrum (151 MHz, C_6D_6, 25 °C) after catalytic hydrogenation (2 h) of 1,5-hexadiene with $Ba[N(Si^iPr_3)_2]_2$ (**1-Ba**) (10 mol%) and H_2 (6 bar) at 120 °C showing the formation of hexane and additionally methylcyclopentane (●) as product of an intramolecular cyclization side reaction followed by hydrogenolysis.[S17] *Note:* Deuterium incorporation in the hexane product (due to H/D isotope exchange in C_6D_6) is visible in both the $^{13}C\{^1H\}$ NMR and GC/MS spectrum (Table S3, entry 32).

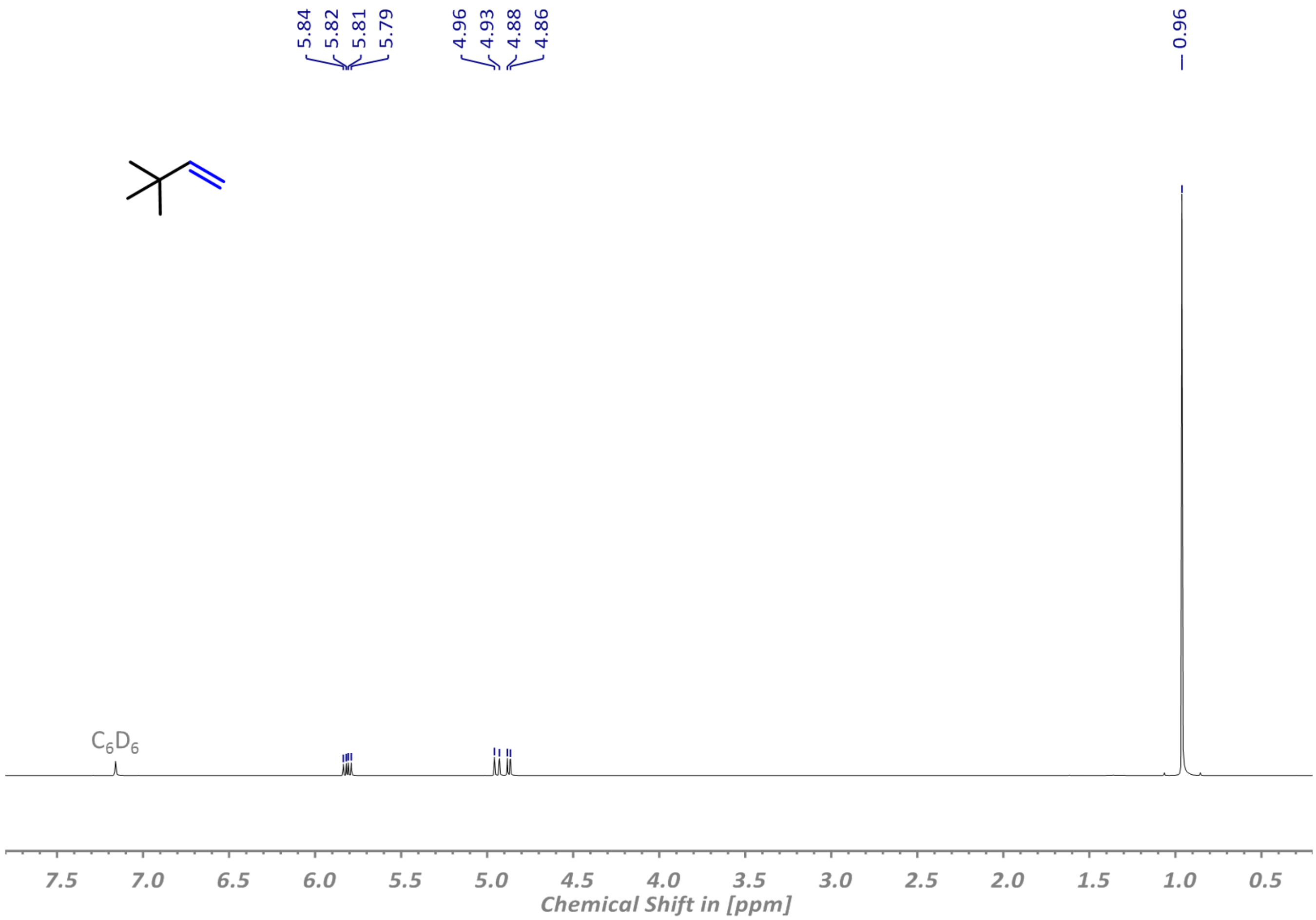

Figure S121. ^{1}H NMR spectrum (600 MHz, C_6D_6, 25 °C) of 3,3-dimethyl-1-butene.

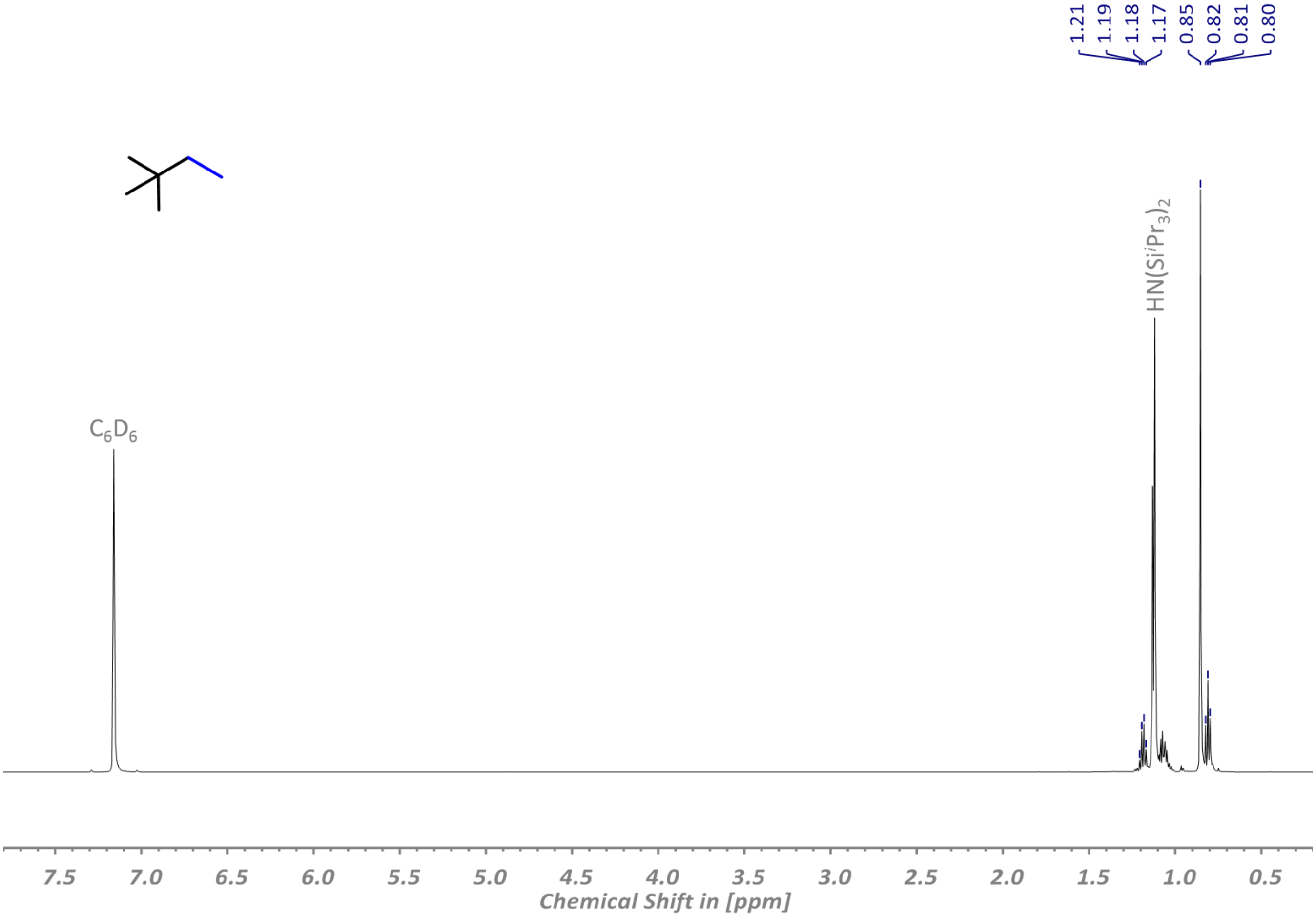

Figure S122. ^{1}H NMR spectrum (600 MHz, C_6D_6, 25 °C) of 2,2-dimethylbutane after catalytic hydrogenation (2 h) of 3,3-dimethyl-1-butene with Ba[N(Si^{i}Pr$_3$)$_2$]$_2$ (**1-Ba**) (10 mol%) and H_2 (6 bar) at 120 °C (Table S3, entry 33).

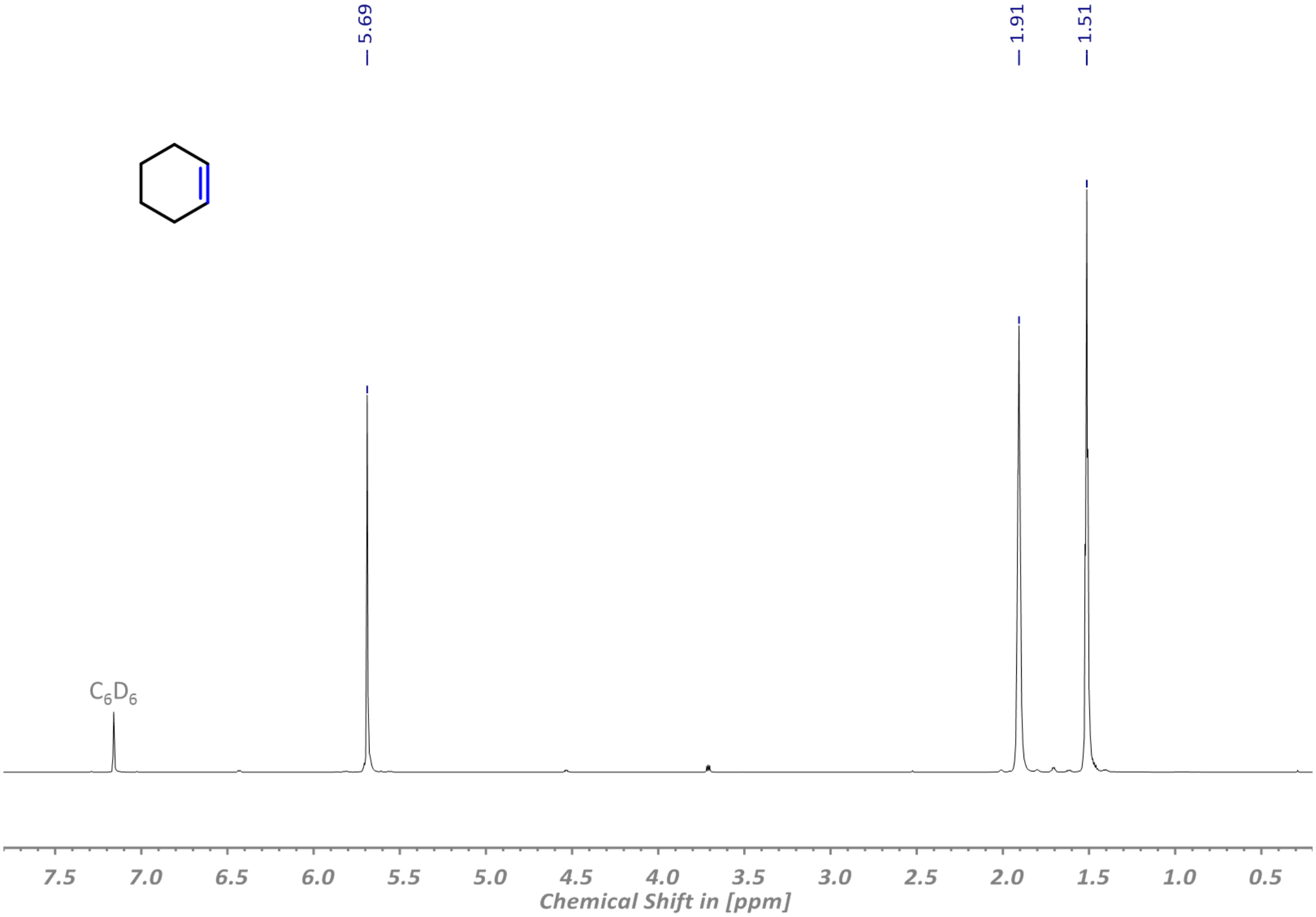

Figure S123. ^{1}H NMR spectrum (600 MHz, C_6D_6, 25 °C) of cyclohexene.

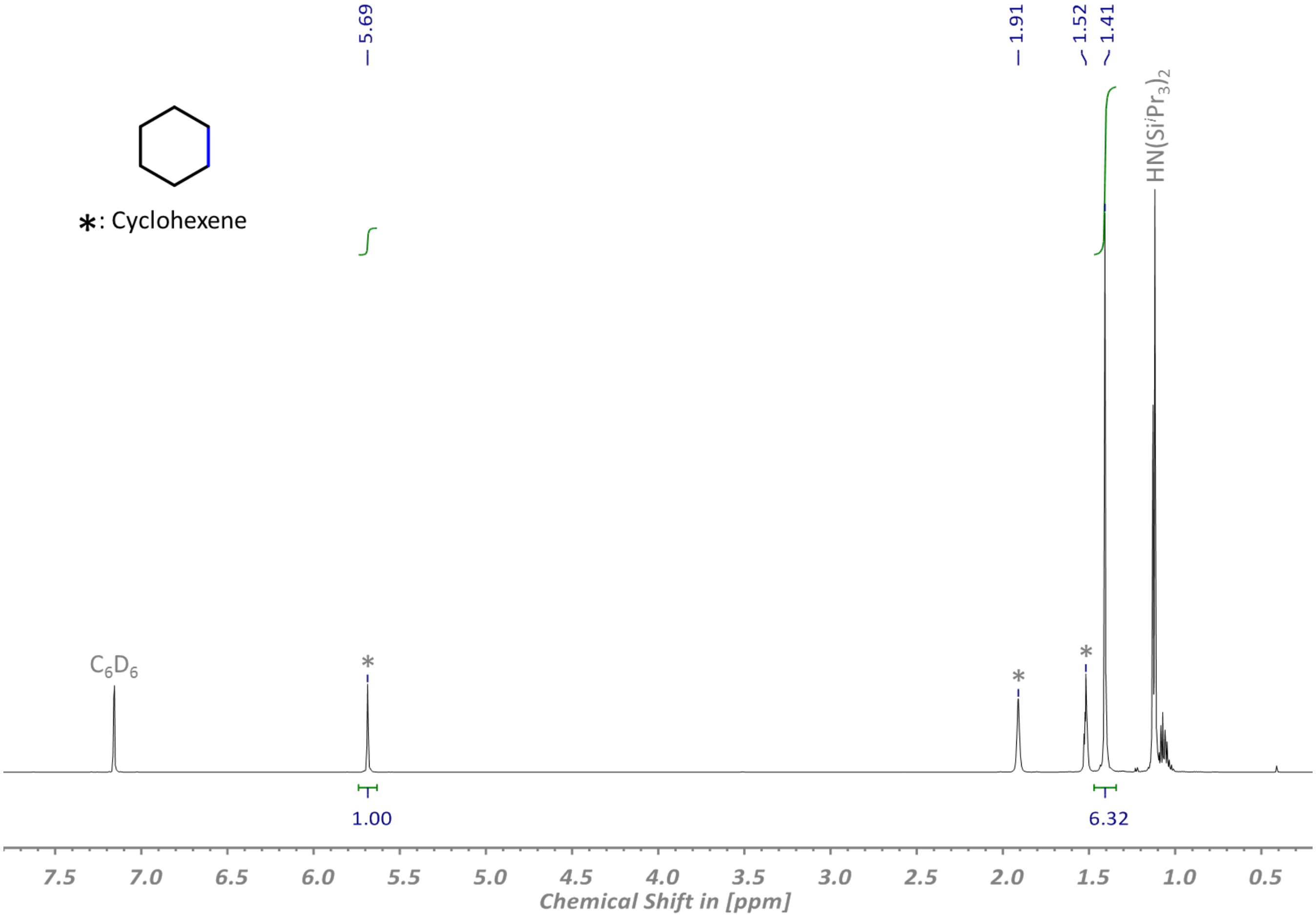

Figure S124. ^{1}H NMR spectrum (600 MHz, C_6D_6, 25 °C) after catalytic hydrogenation (24 h) of cyclohexene (∗) with Ca[N(Si^{i}Pr$_3$)$_2$]$_2$ (**1-Ca**) (10 mol%) and H_2 (6 bar) at 120 °C showing the formation of cyclohexane (Table S3, entry 34).

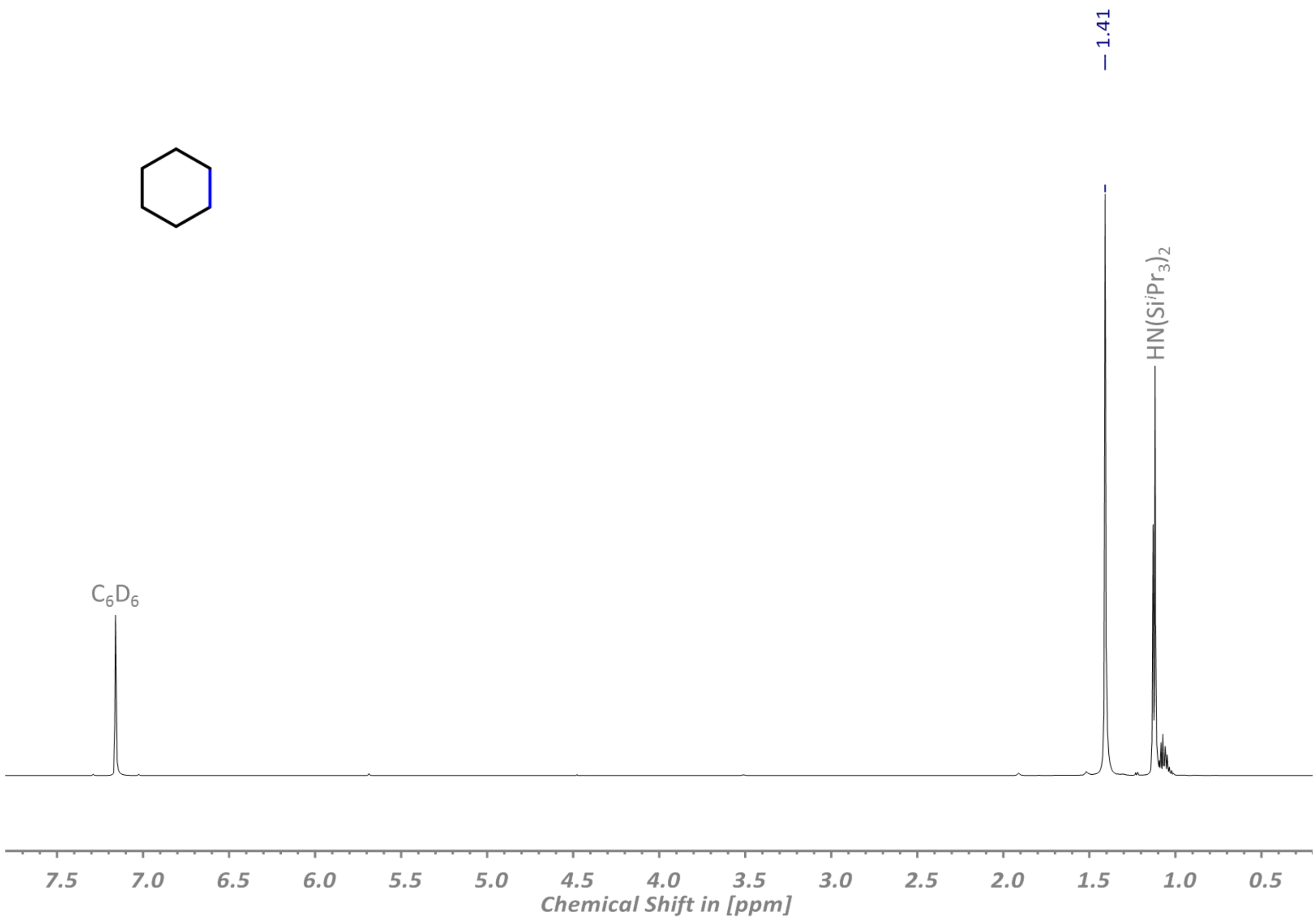

Figure S125. ^{1}H NMR spectrum (600 MHz, C_6D_6, 25 °C) of cyclohexane after catalytic hydrogenation (10 h) of cyclohexene with Sr[N(Si^{i}Pr$_3$)$_2$]$_2$ (**2-Sr**) (10 mol%) and H_2 (6 bar) at 120 °C (Table S3, entry 35).

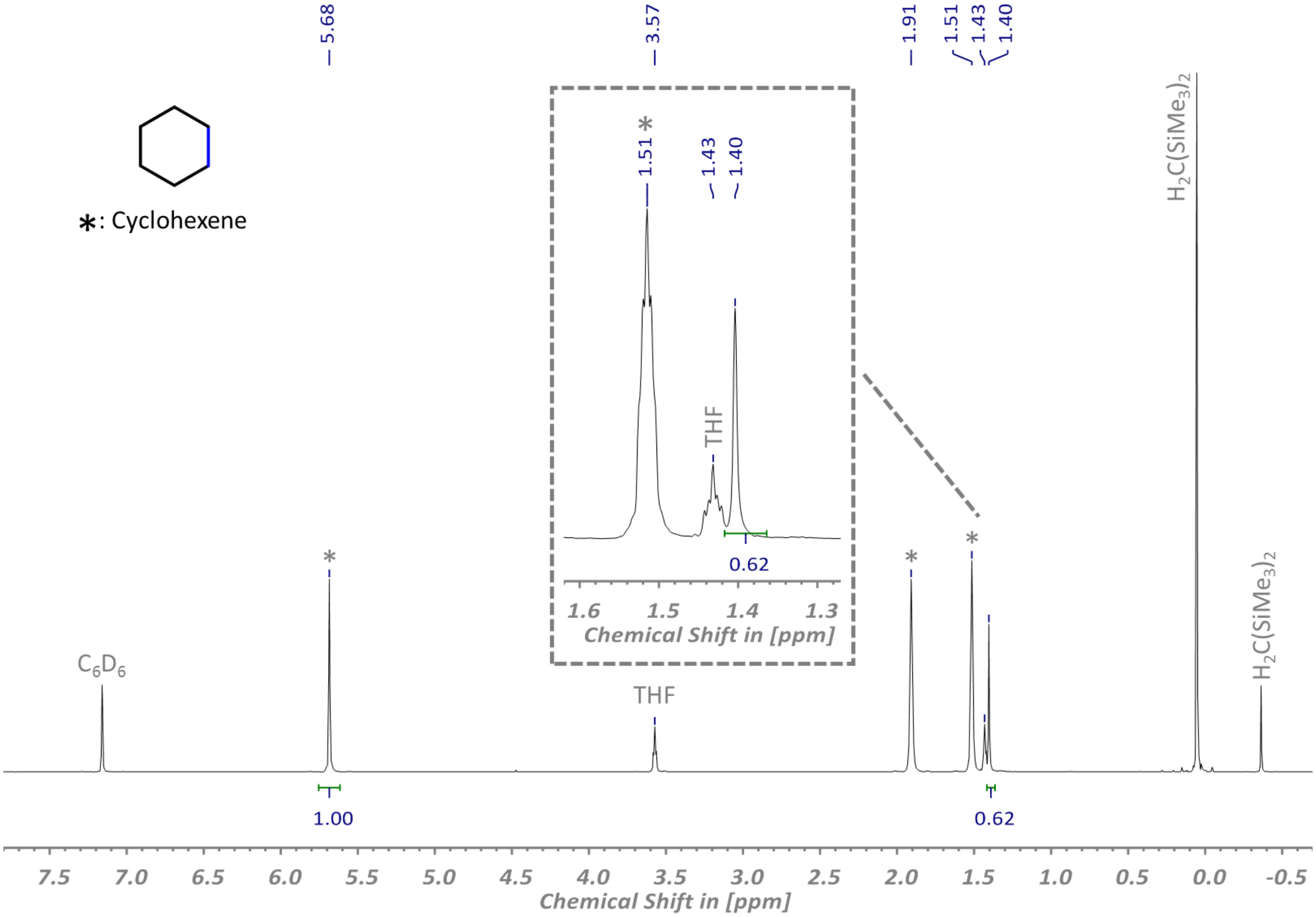

Figure S126. ^{1}H NMR spectrum (600 MHz, C_6D_6, 25 °C) after catalytic hydrogenation (24 h) of cyclohexene (∗) with $Sr[CH(SiMe_3)_2]_2(THF)_2$ (10 mol%) and H_2 (6 bar) at 120 °C showing the formation of cyclohexane. *Note:* The cyclohexane and THF resonances are superimposed preventing an accurate determination of the conversion by integration.

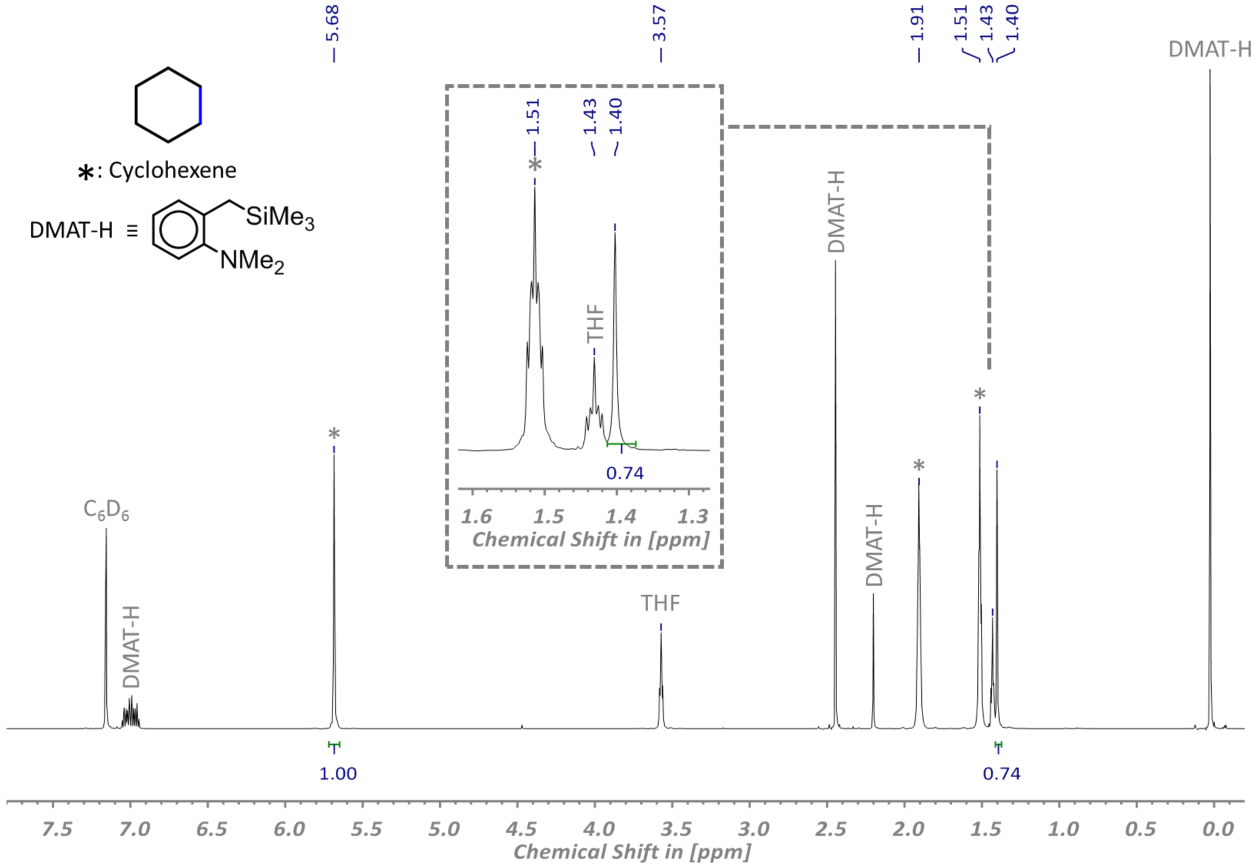

Figure S127. ^{1}H NMR spectrum (600 MHz, C_6D_6, 25 °C) after catalytic hydrogenation (24 h) of cyclohexene (∗) with $(DMAT)_2Sr(THF)_3$ (10 mol%) and H_2 (6 bar) at 120 °C showing the formation of cyclohexane. *Note:* The cyclohexane and THF resonances are superimposed preventing an accurate determination of the conversion by integration.

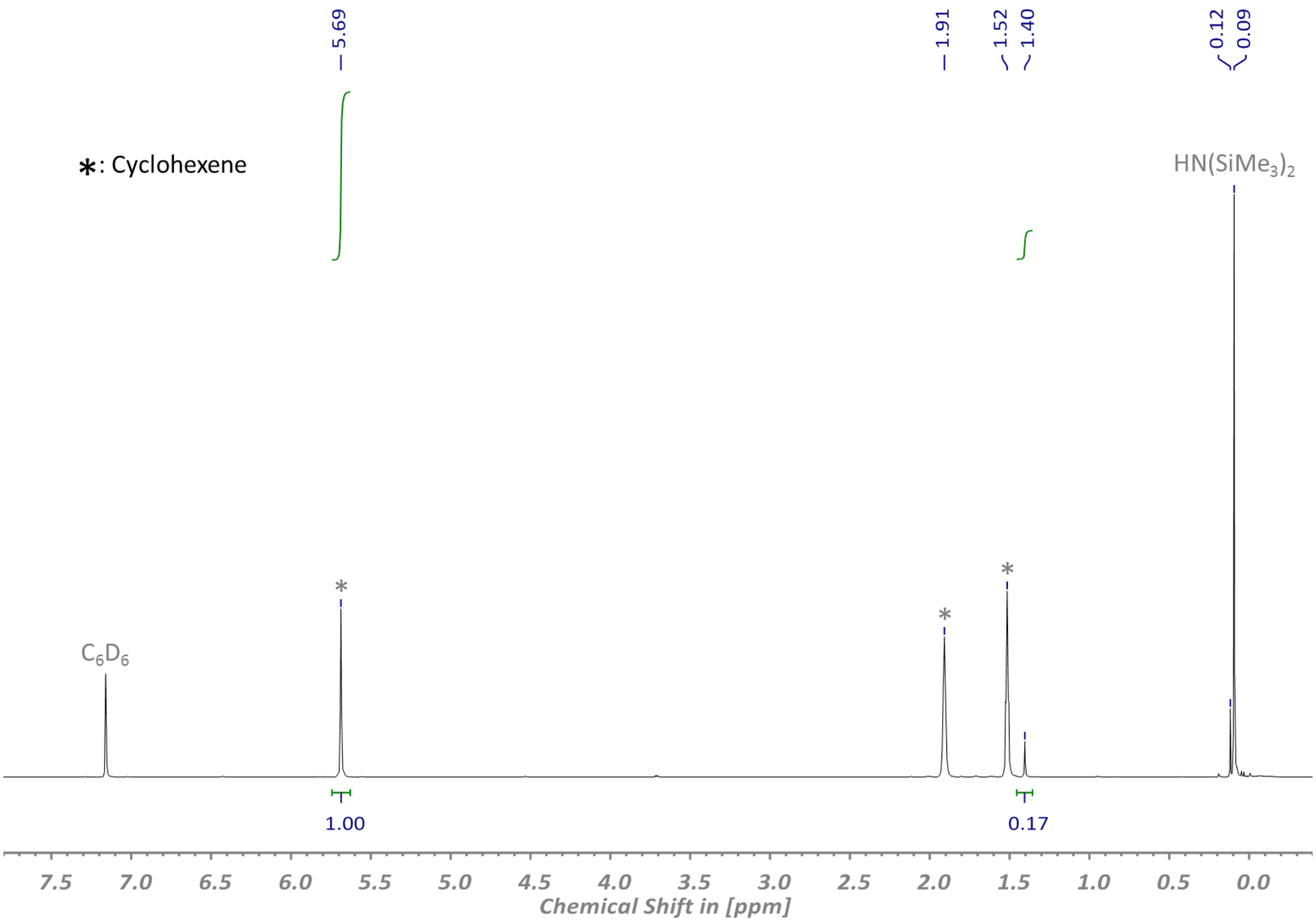

Figure S128. ^{1}H NMR spectrum (600 MHz, C_6D_6, 25 °C) after catalytic hydrogenation (24 h) of cyclohexene (∗) with $Ba[N(SiMe_3)_2]_2$ (10 mol%) and H_2 (6 bar) at 120 °C showing the formation of cyclohexane in a substoichiometric amount (Table S3, entry 36).

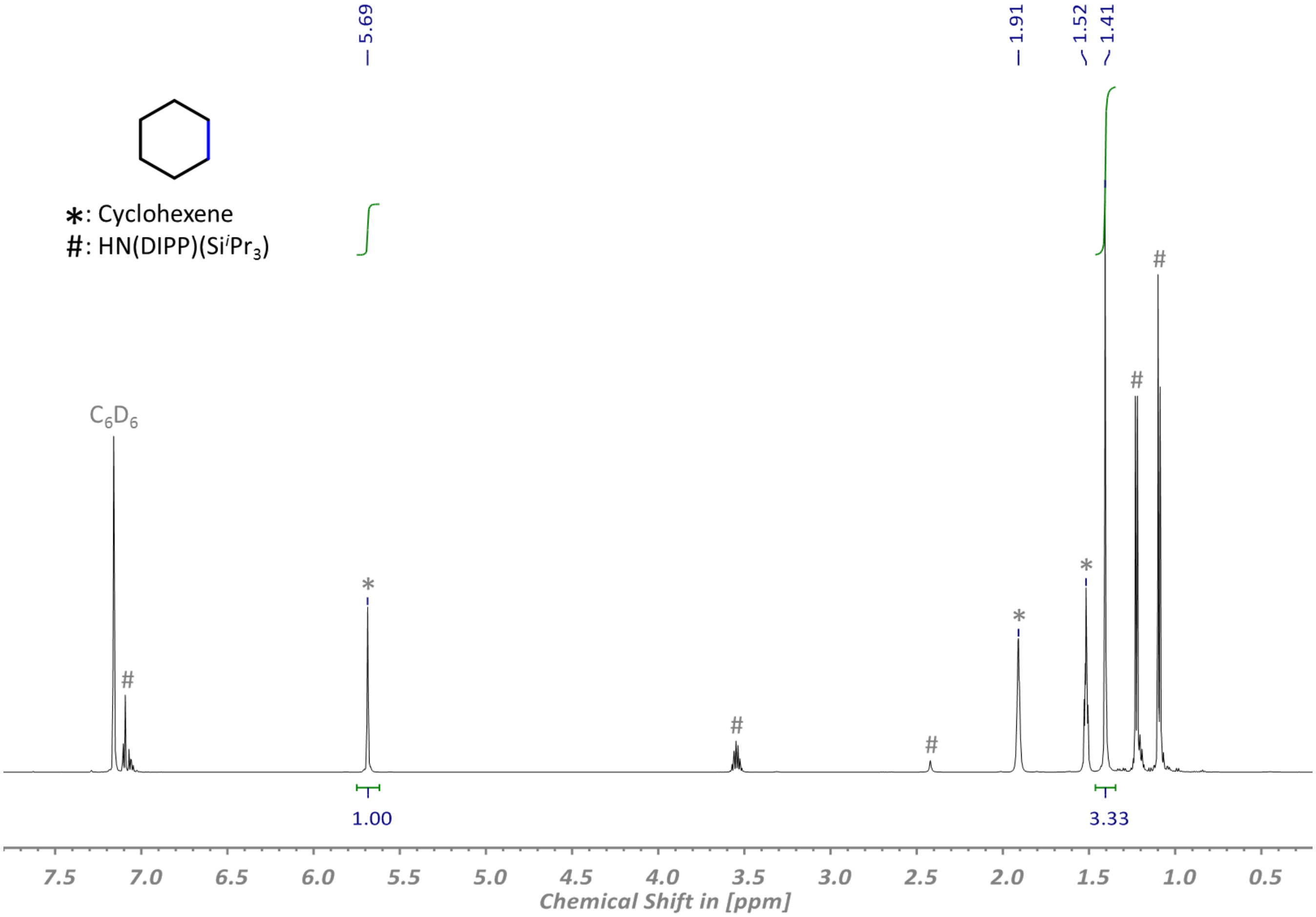

Figure S129. ^{1}H NMR spectrum (600 MHz, C_6D_6, 25 °C) after catalytic hydrogenation (24 h) of cyclohexene (∗) with Ba[N(DIPP)(Si^{i}Pr$_3$)]$_2$ (**2-Ba**) (10 mol%) and H_2 (6 bar) at 120 °C showing the formation of cyclohexane. *Note:* # denotes HN(DIPP)(Si^{i}Pr$_3$) (Table S3, entry 37).

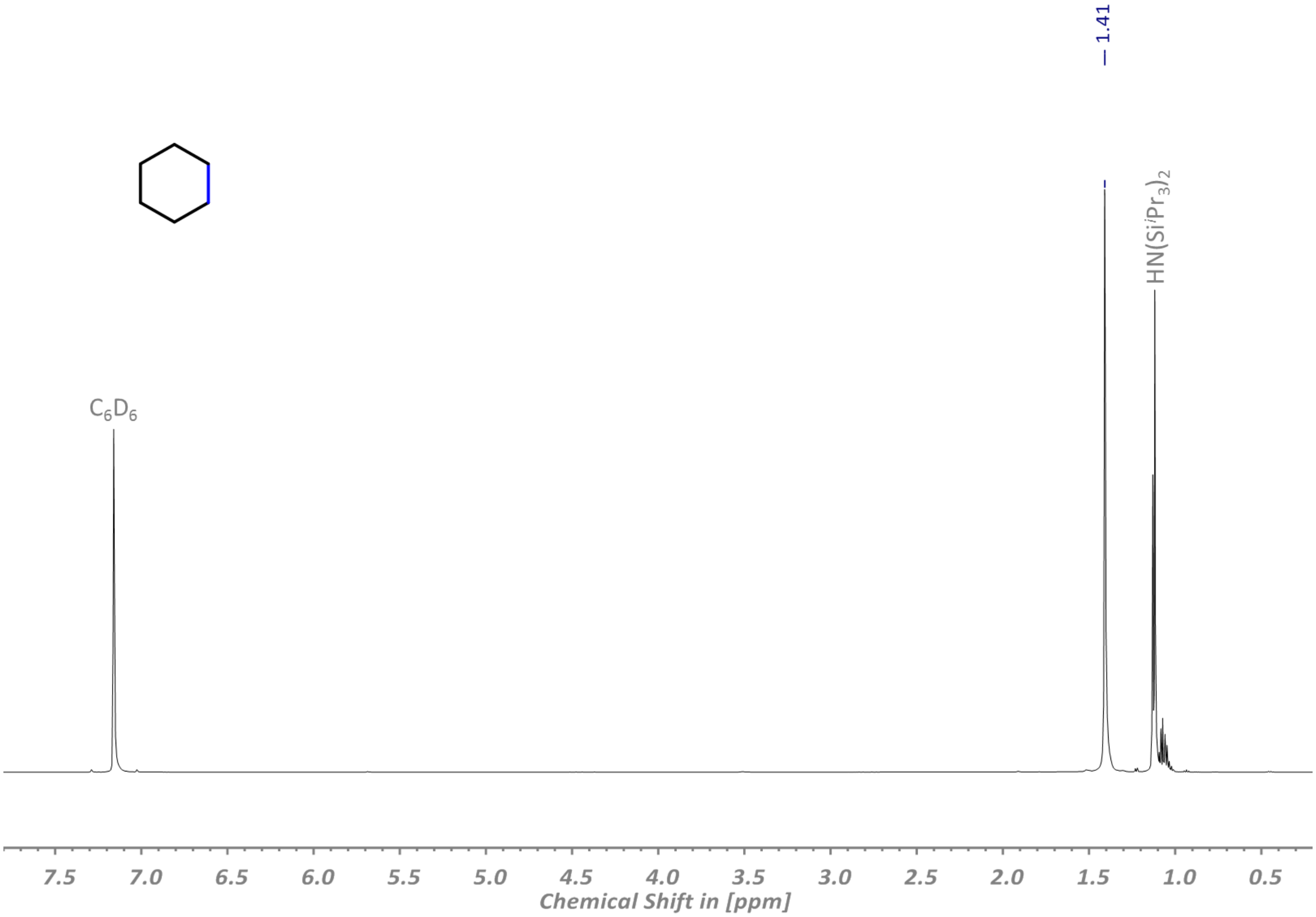

Figure S130. ^{1}H NMR spectrum (600 MHz, C_6D_6, 25 °C) of cyclohexane after catalytic hydrogenation (3 h) of cyclohexene with Ba[N(Si^{i}Pr$_3$)$_2$]$_2$ (**1-Ba**) (10 mol%) and H_2 (6 bar) at 120 °C (Table S3, entry 38).

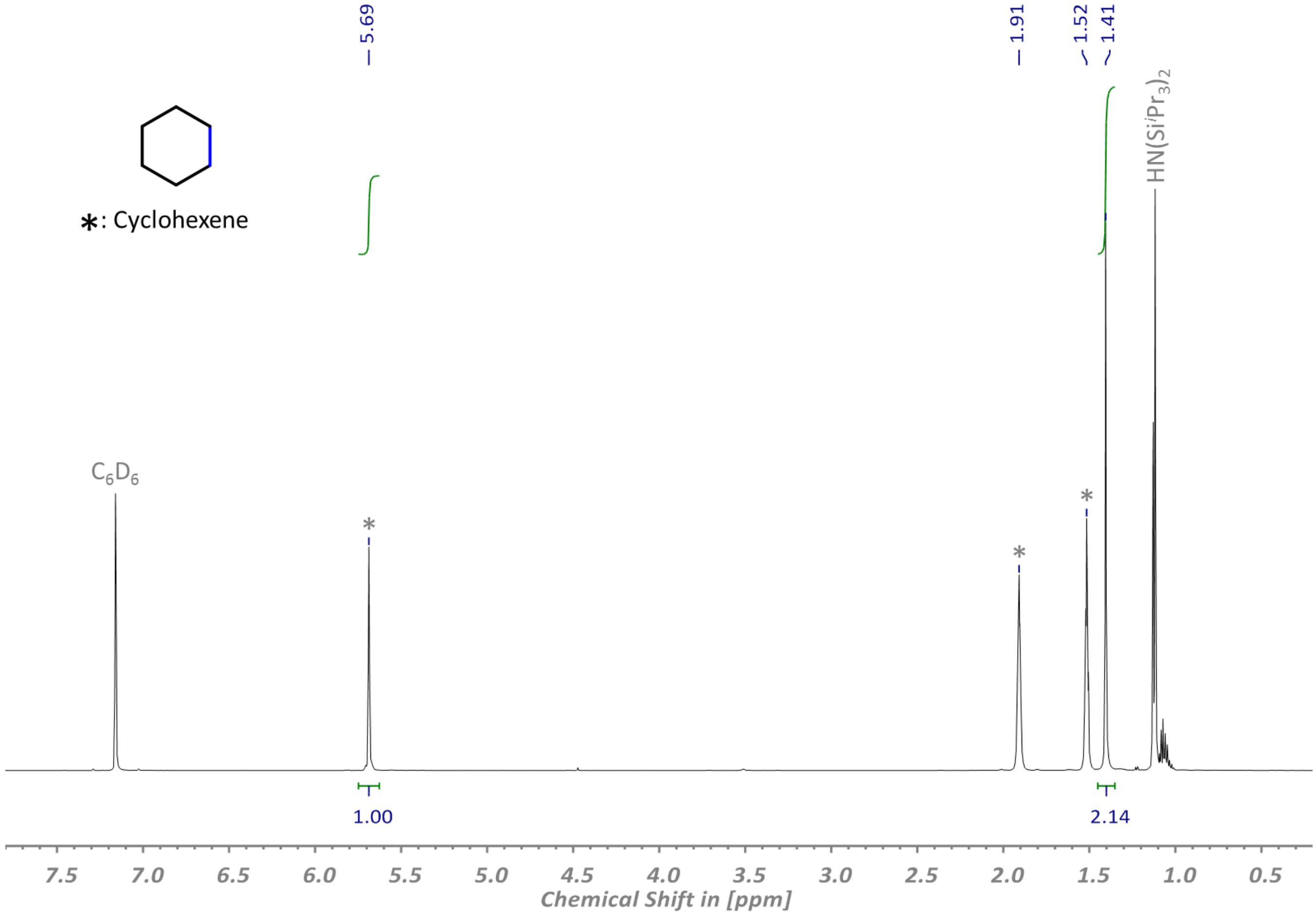

Figure S131. ^{1}H NMR spectrum (600 MHz, C_6D_6, 25 °C) after catalytic hydrogenation (24 h) of cyclohexene (∗) with Ba[N(Si^{i}Pr$_3$)$_2$]$_2$ (**1-Ba**) (5 mol%) and H_2 (6 bar) at 120 °C showing the formation of cyclohexane (Table S3, entry 39).

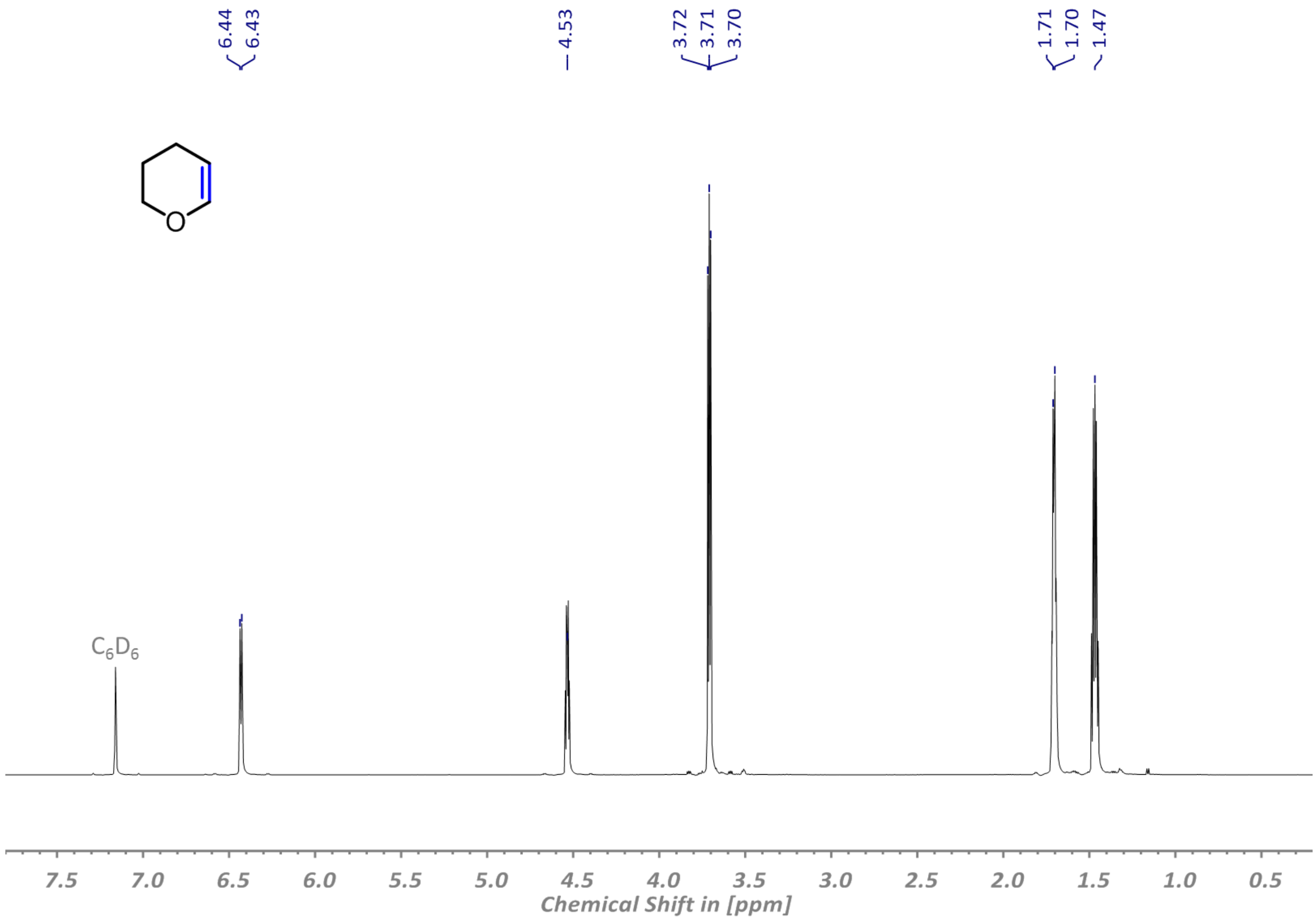

Figure S132. ^{1}H NMR spectrum (600 MHz, C_6D_6, 25 °C) of 3,4-dihydro-2*H*-pyran.

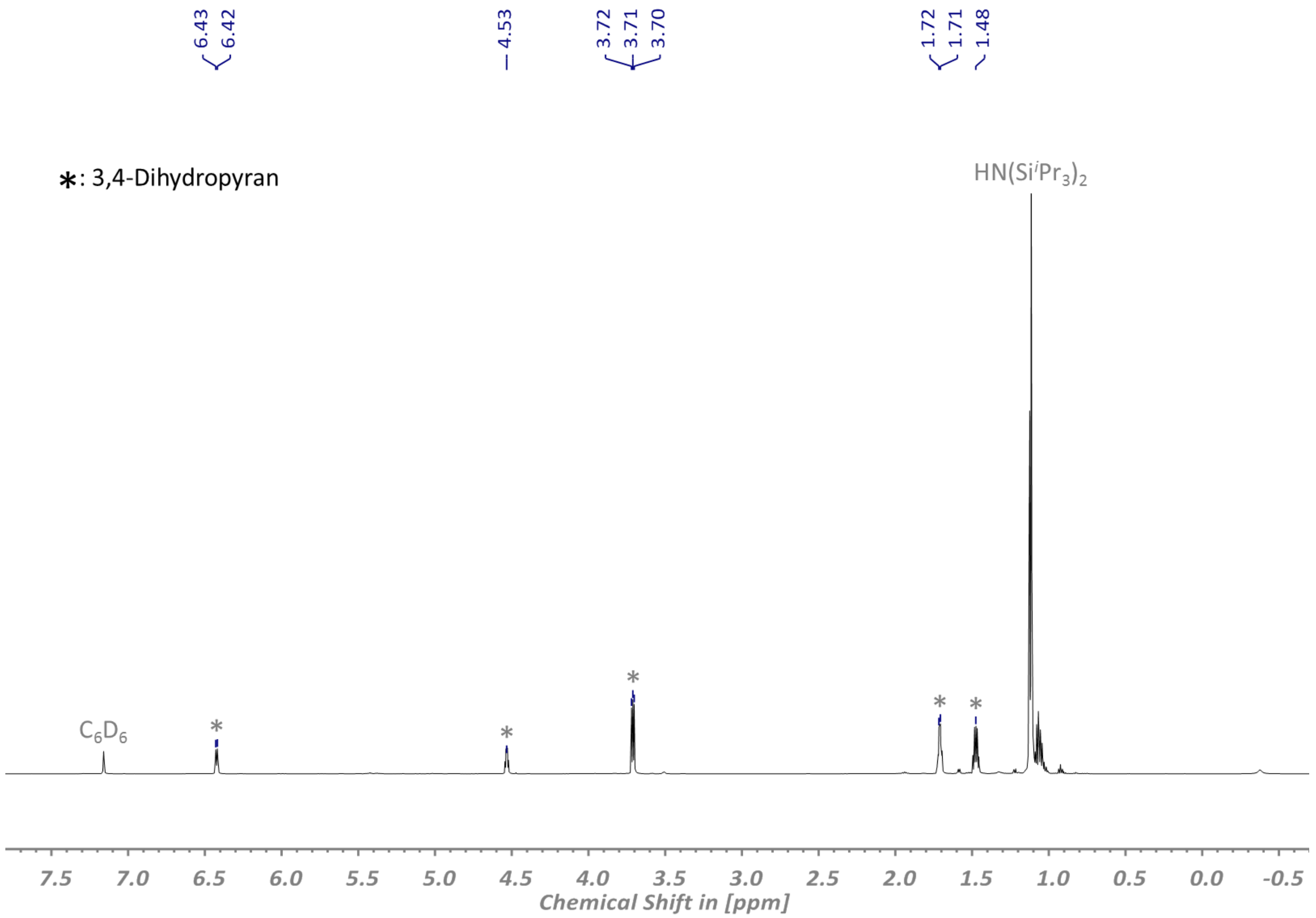

Figure S133. ^{1}H NMR spectrum (600 MHz, C_6D_6, 25 °C) after the reaction (24 h) of 3,4-dihydro-2*H*-pyran (∗) with Ba[N(Si^{i}Pr$_3$)$_2$]$_2$ (**1-Ba**) (10 mol%) and H_2 (6 bar) at 120 °C. *Note:* No formation of tetrahydropyran is observed (Table S3, entry 40).

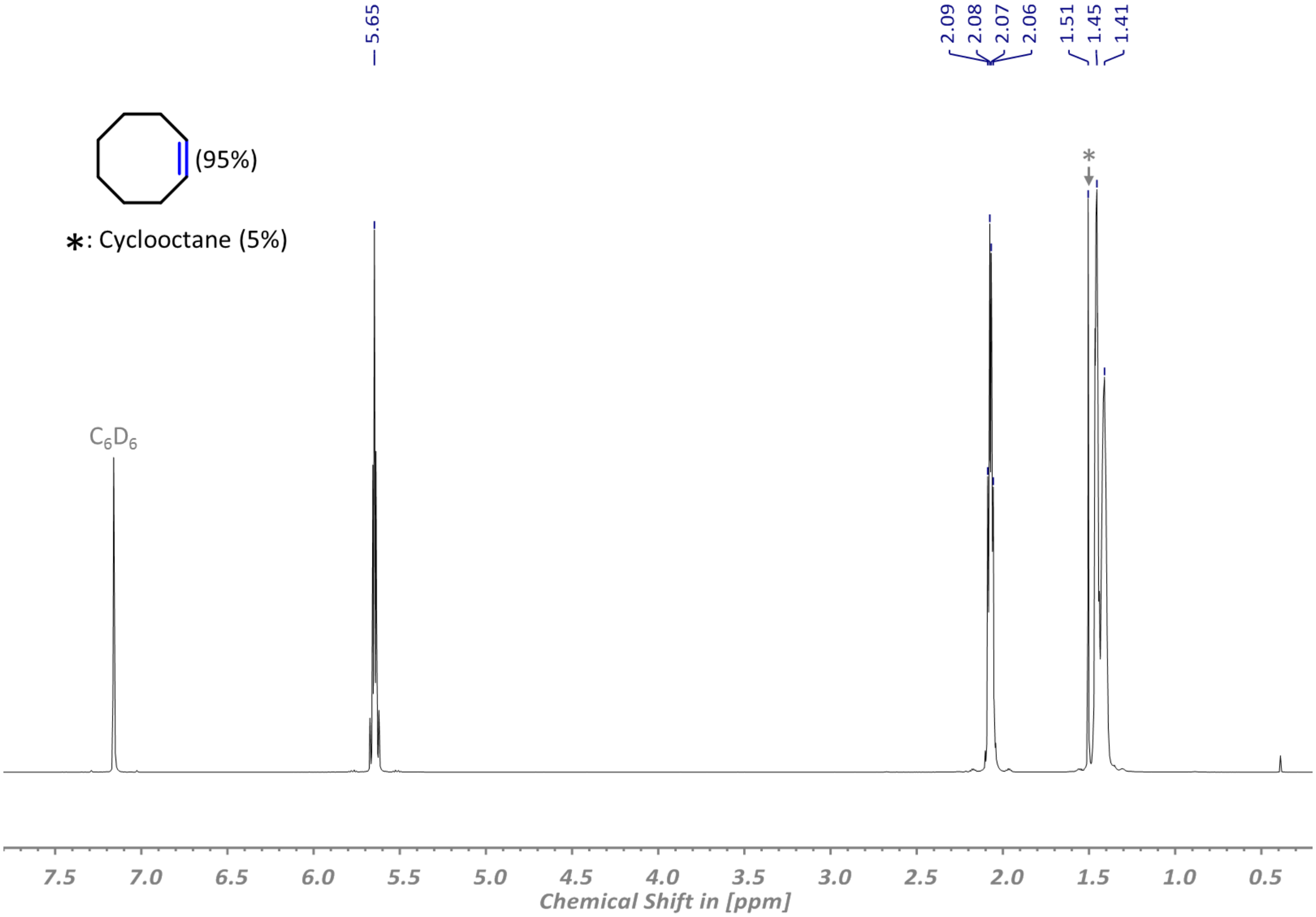

Figure S134. ^{1}H NMR spectrum (600 MHz, C_6D_6, 25 °C) of *cis*-cyclooctene (>95%). *Note:* ∗ denotes traces of a cyclooctane impurity (<5%) as evidenced by GC/MS analysis.

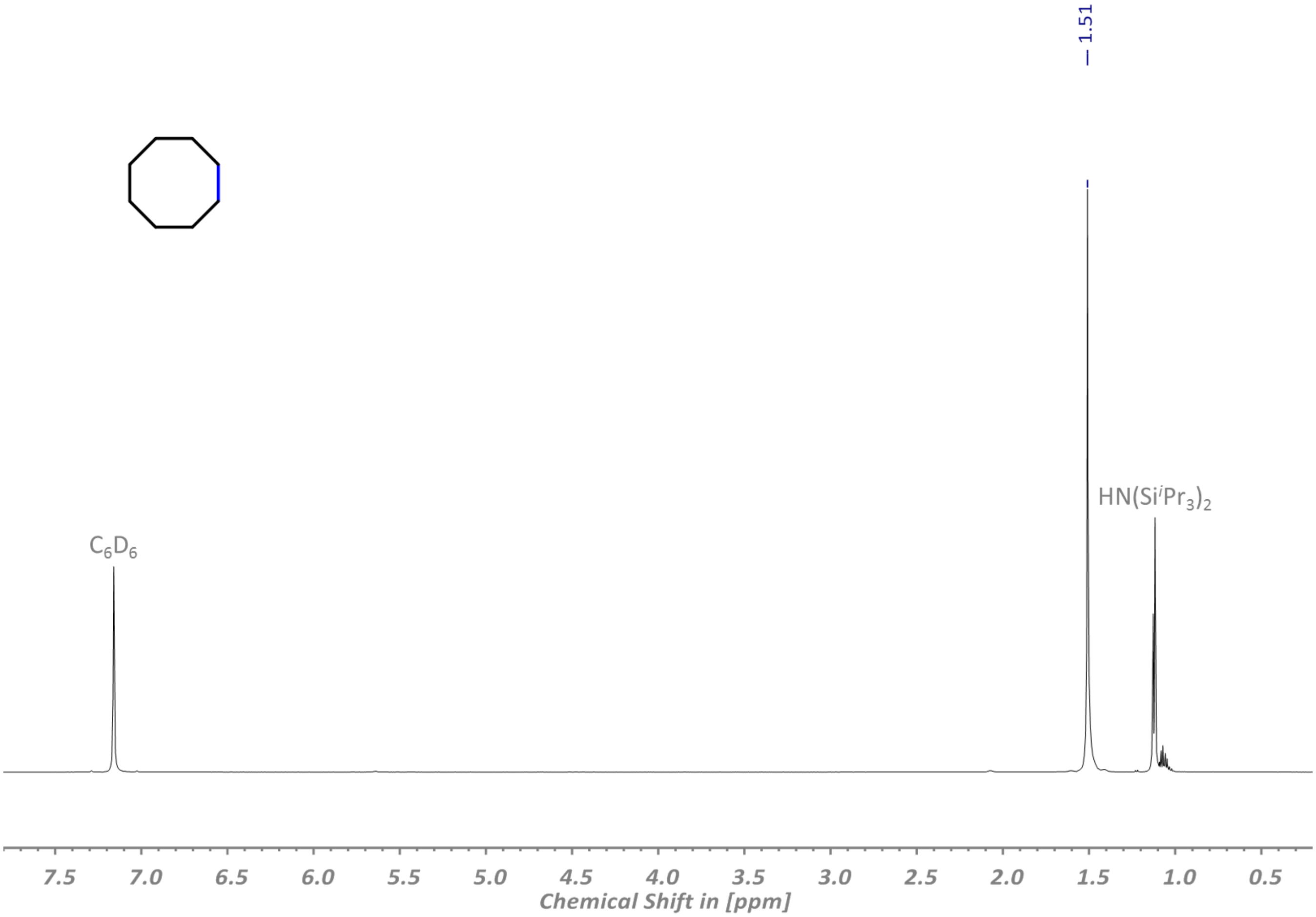

Figure S135. ^{1}H NMR spectrum (600 MHz, C_6D_6, 25 °C) of cyclooctane after catalytic hydrogenation (1.5 h) of *cis*-cyclooctene with Ba[N(Si^{i}Pr$_3$)$_2$]$_2$ (**1-Ba**) (10 mol%) and H_2 (6 bar) at 120 °C (Table S3, entry 41).

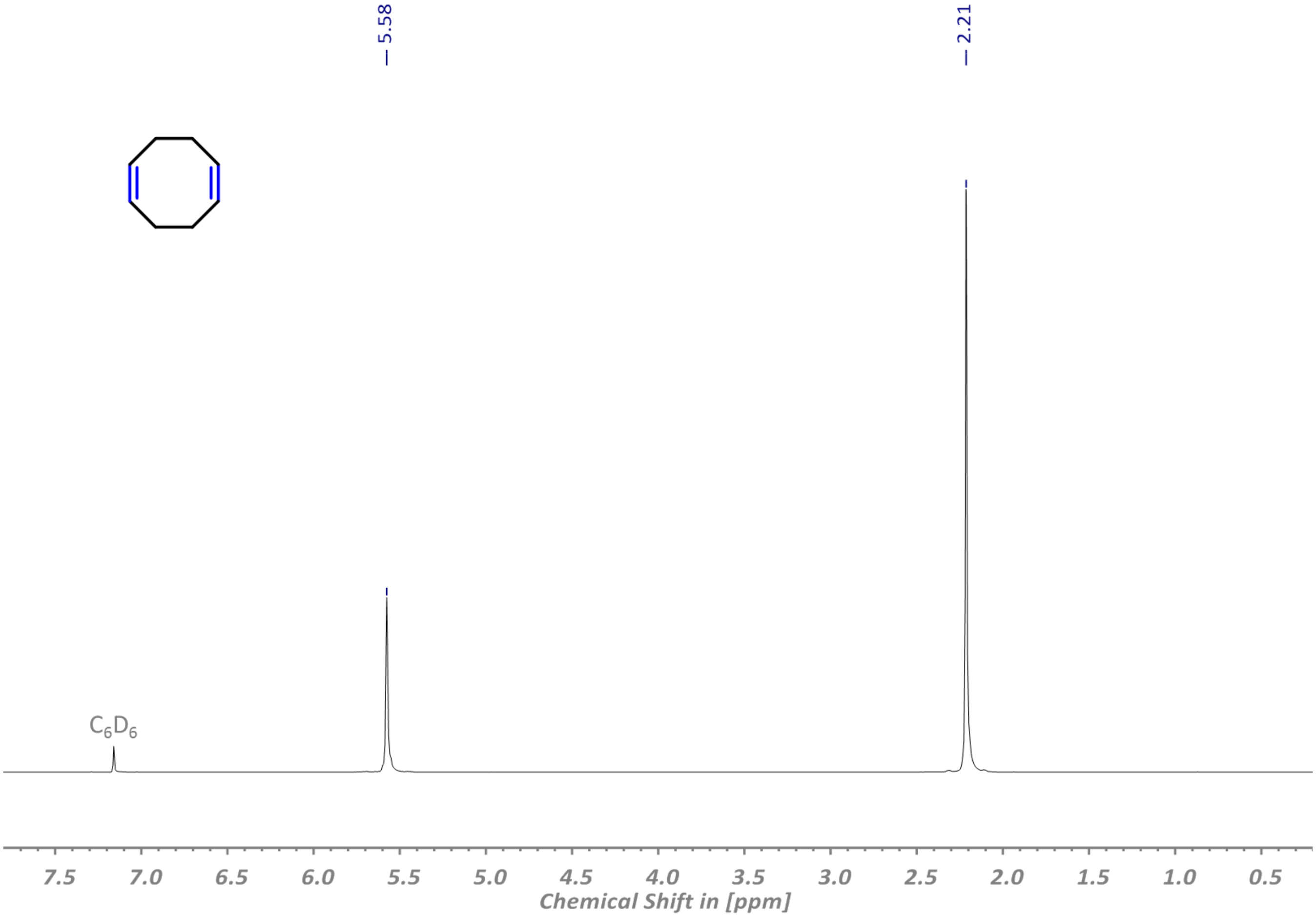

Figure S136. ^{1}H NMR spectrum (600 MHz, C_6D_6, 25 °C) of 1,5-cyclooctadiene.

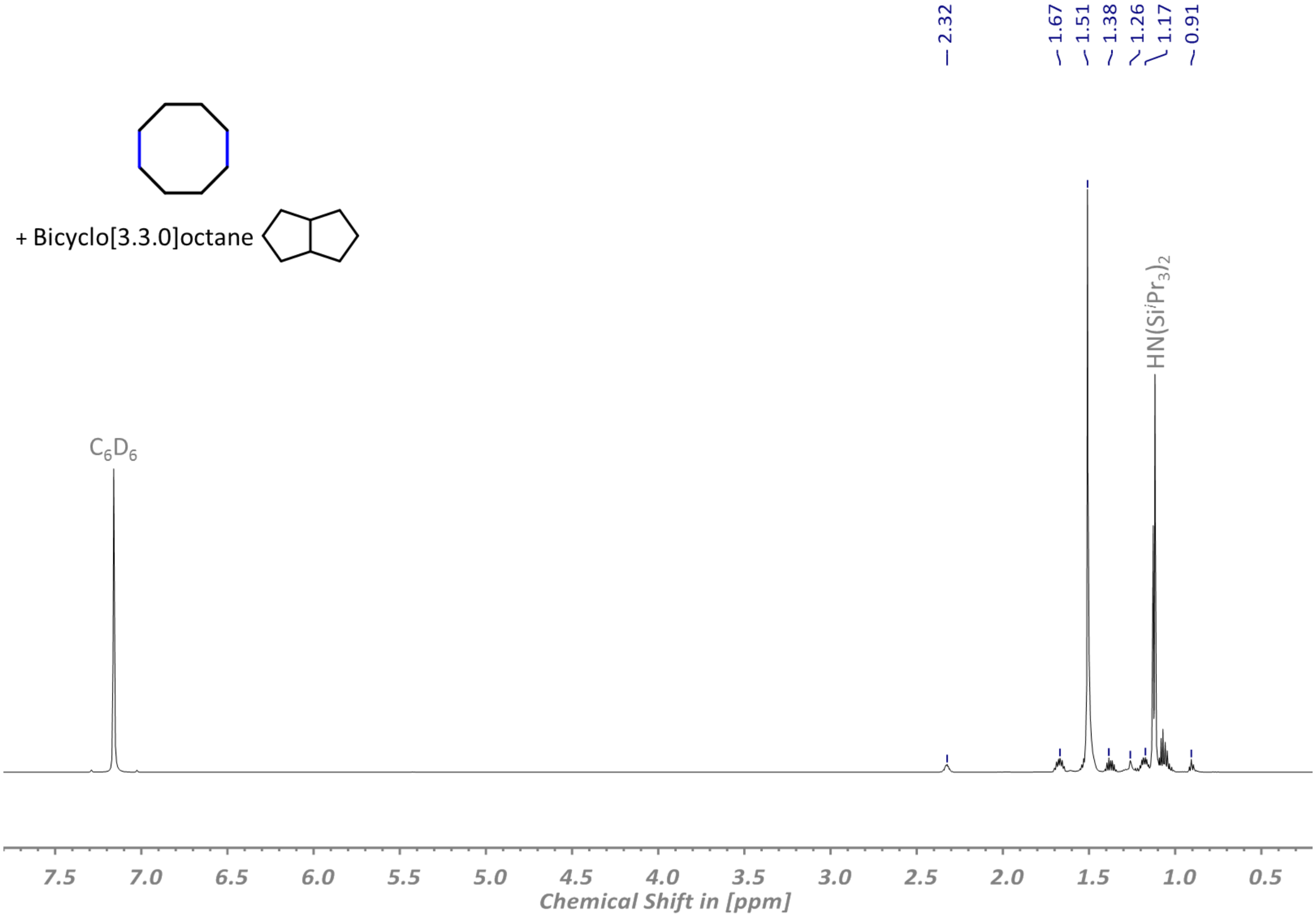

Figure S137. ^{1}H NMR spectrum (600 MHz, C_6D_6, 25 °C) after catalytic hydrogenation (24 h) of 1,5-cyclooctadiene with Ba[N(Si^{i}Pr$_3$)$_2$]$_2$ (**1-Ba**) (10 mol%) and H_2 (6 bar) at 120 °C showing the formation of cyclooctane and bicyclo[3.3.0]octane as side product likely formed *via* an intramolecular cyclization reaction followed by hydrogenolysis. *Note:* Small amounts of unknown species (<6%) are confirmed by GC/MS analysis (Table S3, entry 42).

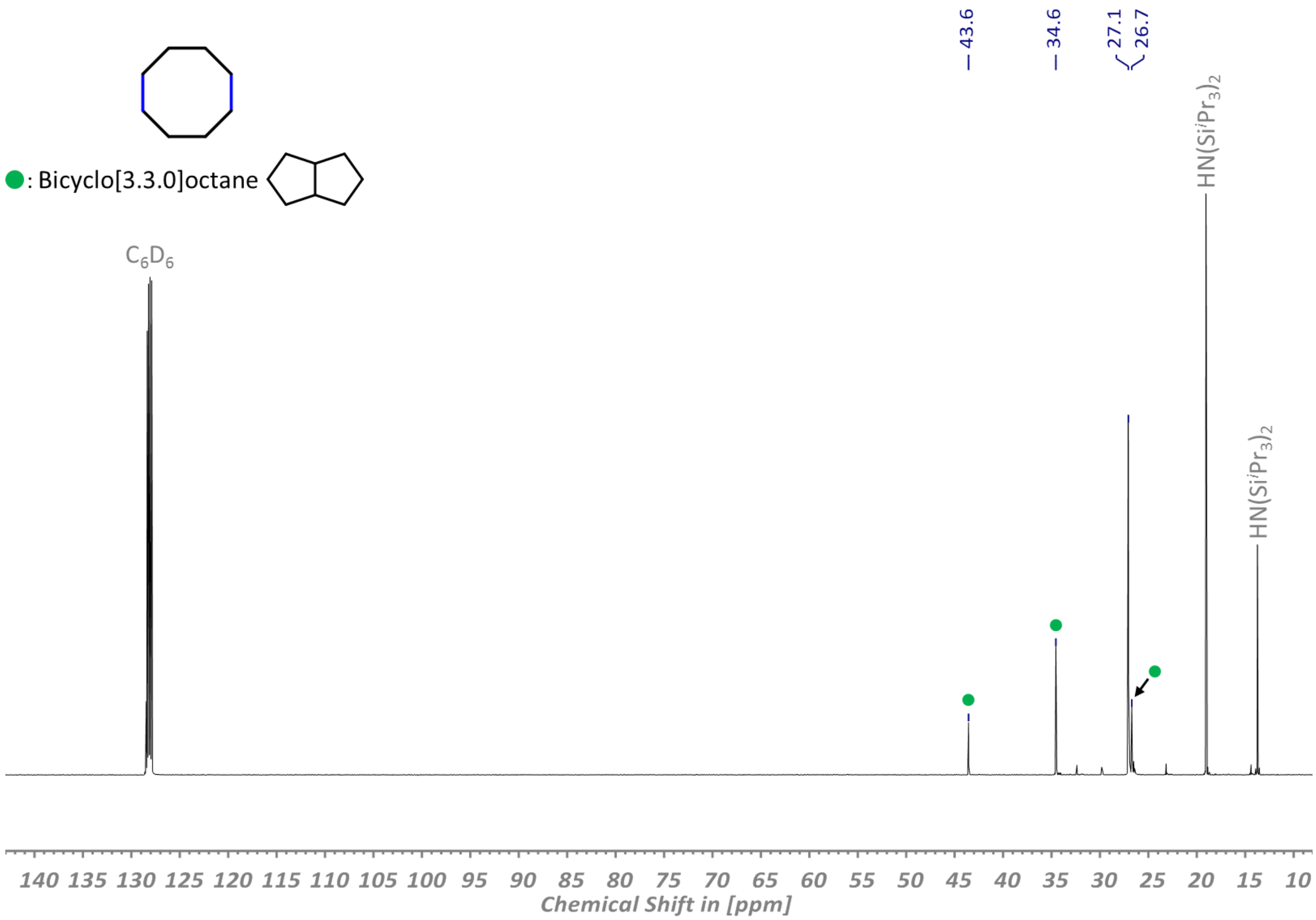

Figure S138. $^{13}\{^1H\}$ NMR spectrum (600 MHz, C_6D_6, 25 °C) after catalytic hydrogenation (24 h) of 1,5-cyclooctadiene with Ba[N(Si^{i}Pr$_3$)$_2$]$_2$ (**1-Ba**) (10 mol%) and H_2 (6 bar) at 120 °C showing the formation of cyclooctane and bicyclo[3.3.0]octane (●) as side product of an intramolecular cyclization reaction followed by hydrogenolysis.[S18] *Note:* Deuterium incorporation in both products (due to H/D isotope exchange in C_6D_6) is observed. The exact conversion was determined from a further independent experiment by GC/MS analysis. (Table S3, entry 46).

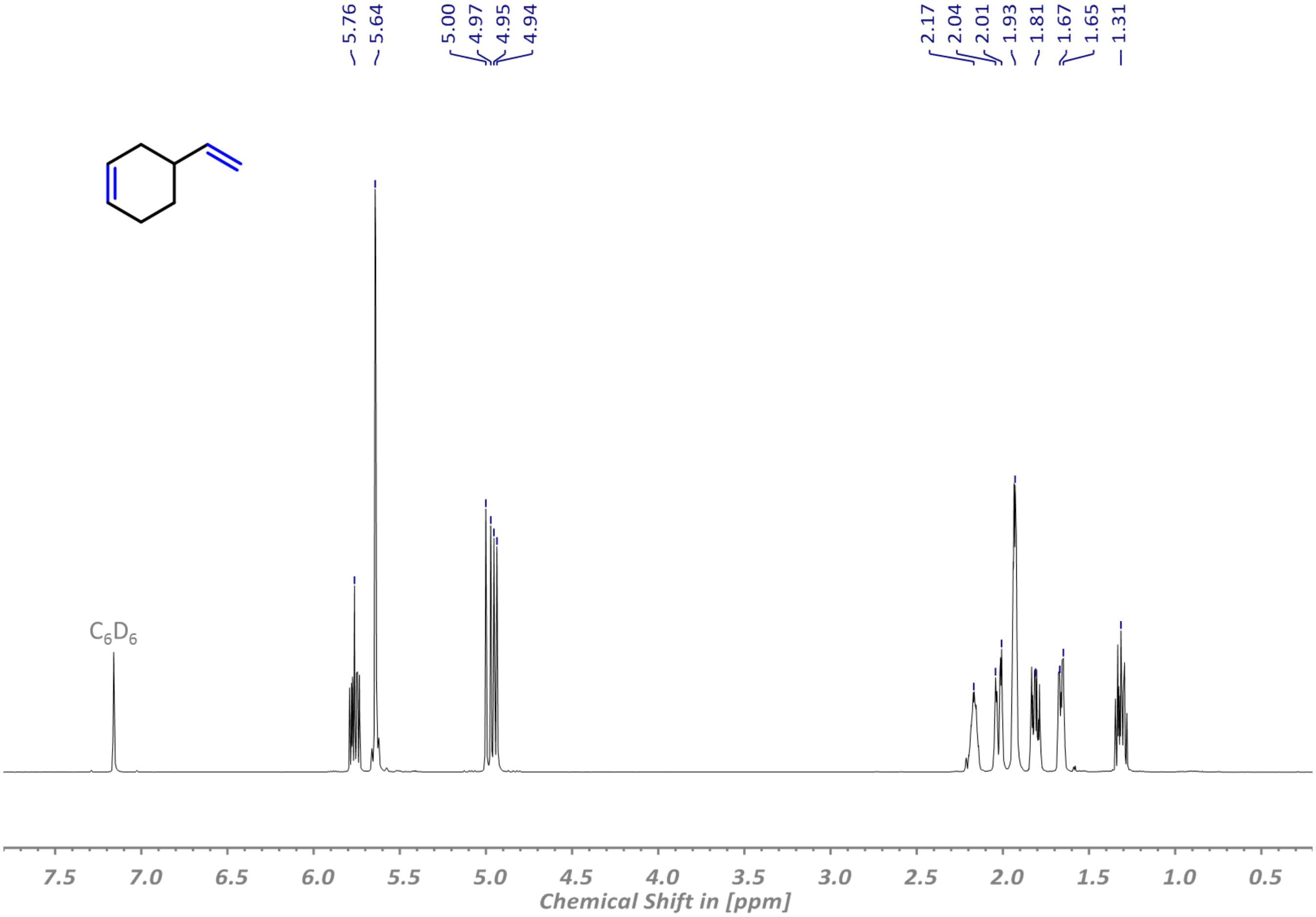

Figure S139. ^{1}H NMR spectrum (600 MHz, C_6D_6, 25 °C) of 4-vinyl-1-cyclohexene.

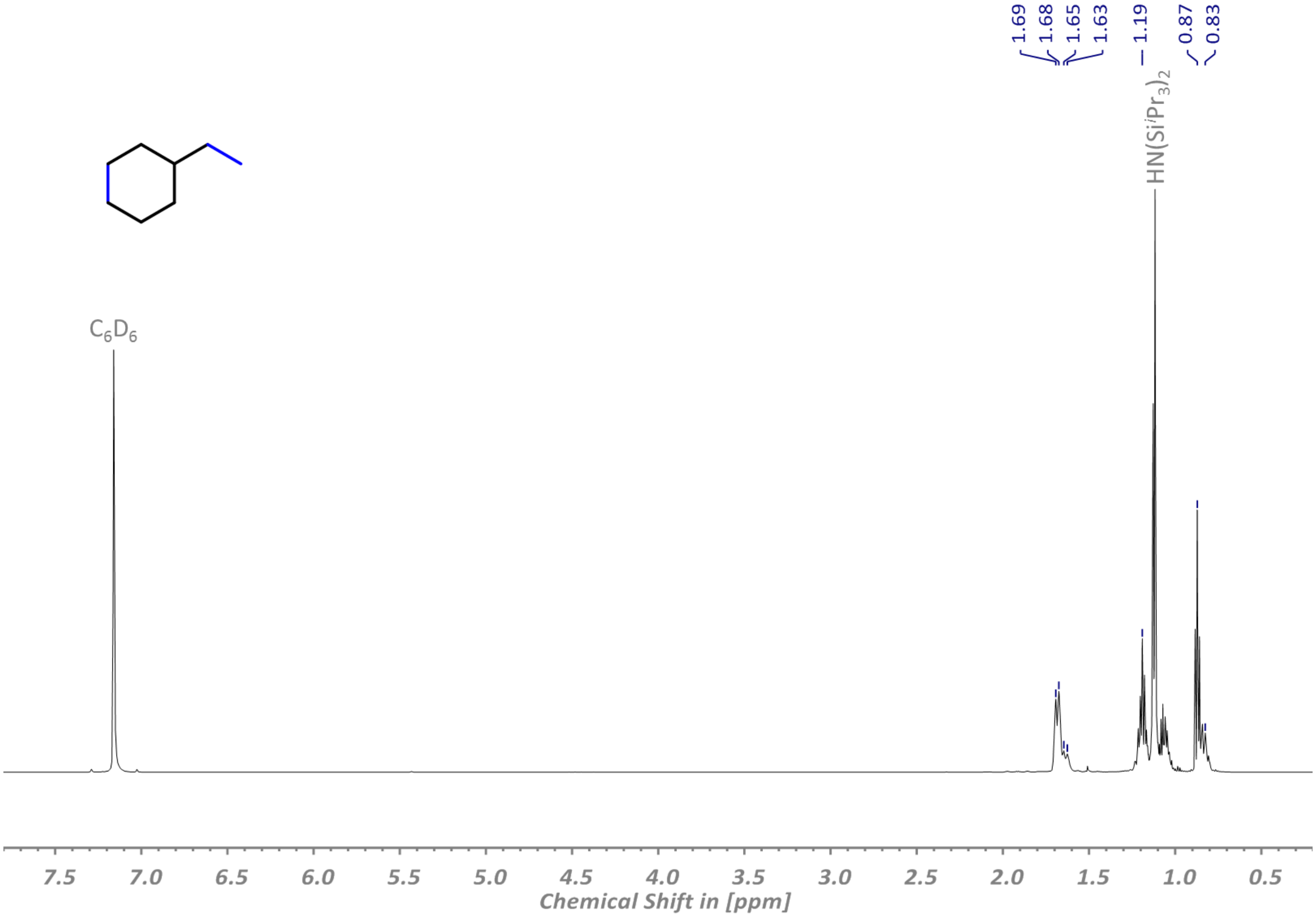

Figure S140. ^{1}H NMR spectrum (600 MHz, C_6D_6, 25 °C) of ethylcyclohexane after catalytic hydrogenation (16 h) of 4-vinyl-1-cyclohexene with Sr[N(Si^{i}Pr$_3$)$_2$]$_2$ (**1-Sr**) (10 mol%) and H_2 (6 bar) at 120 °C (Table S3, entry 43).

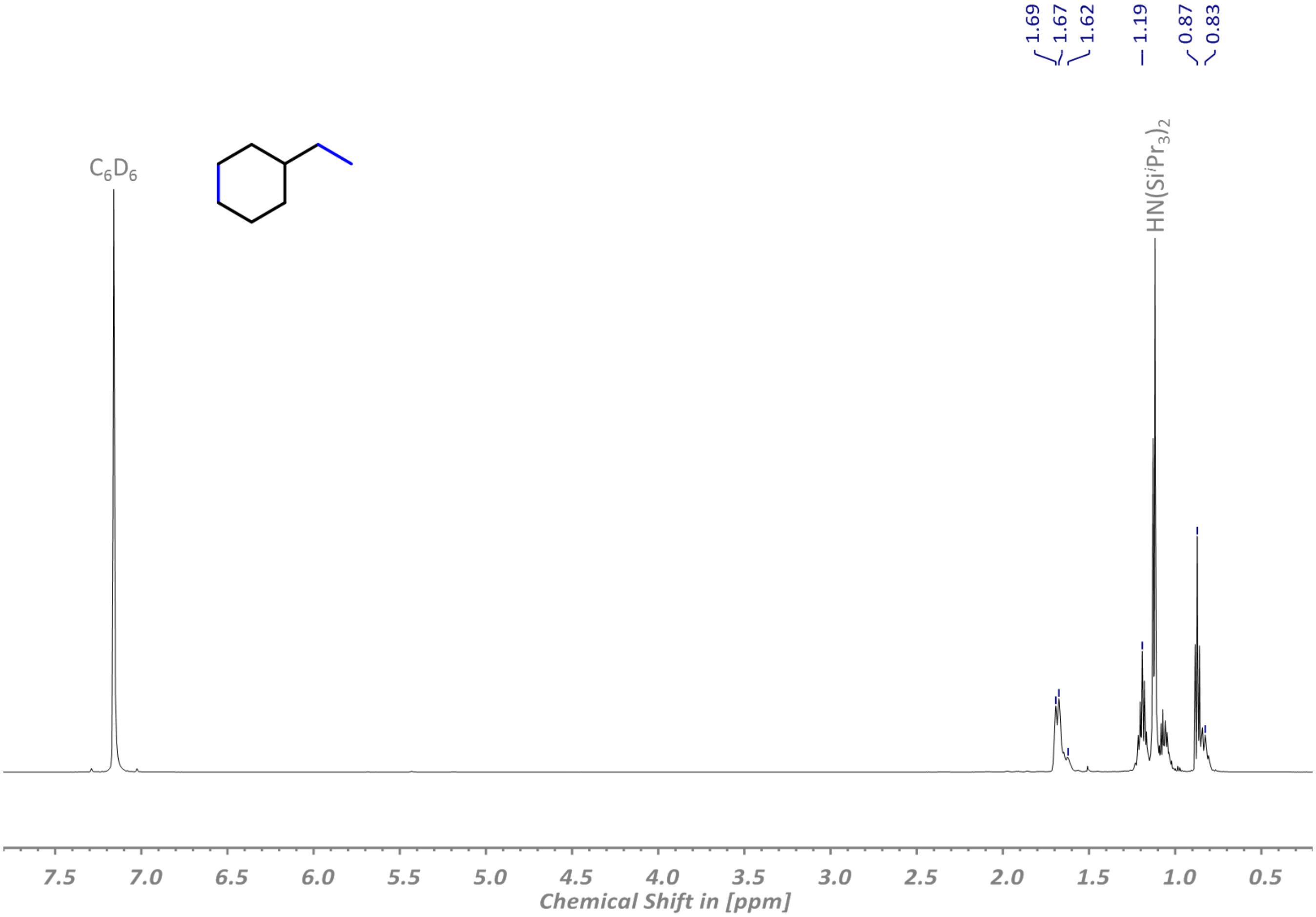

Figure S141. ^{1}H NMR spectrum (600 MHz, C_6D_6, 25 °C) of ethylcyclohexane after catalytic hydrogenation (3.5 h) of 4-vinyl-1-cyclohexene with Ba[N(Si^{i}Pr$_3$)$_2$]$_2$ (**1-Ba**) (10 mol%) and H_2 (6 bar) at 120 °C (Table S3, entry 44).

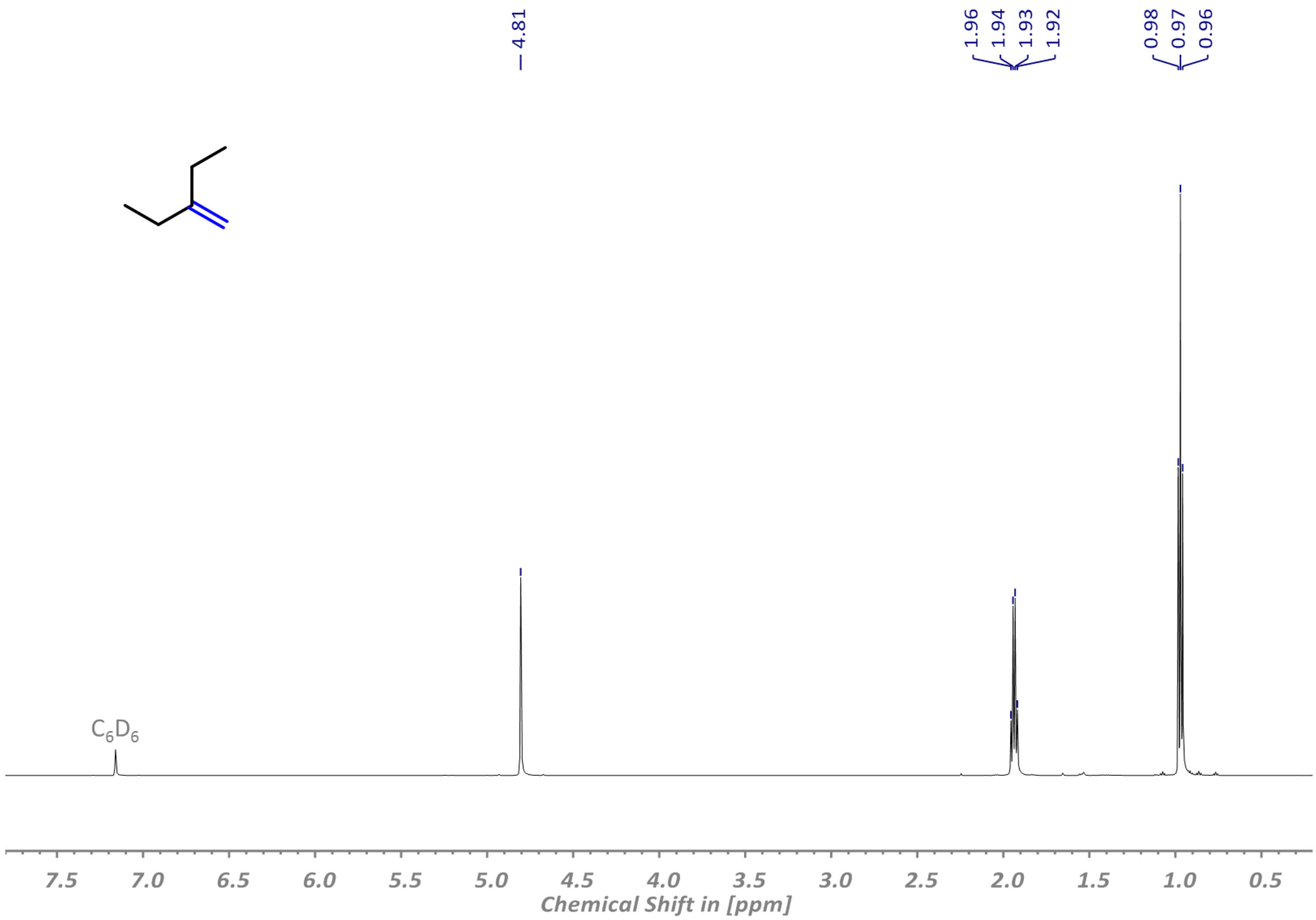

Figure S142. ^{1}H NMR spectrum (600 MHz, C_6D_6, 25 °C) of 2-ethyl-1-butene.

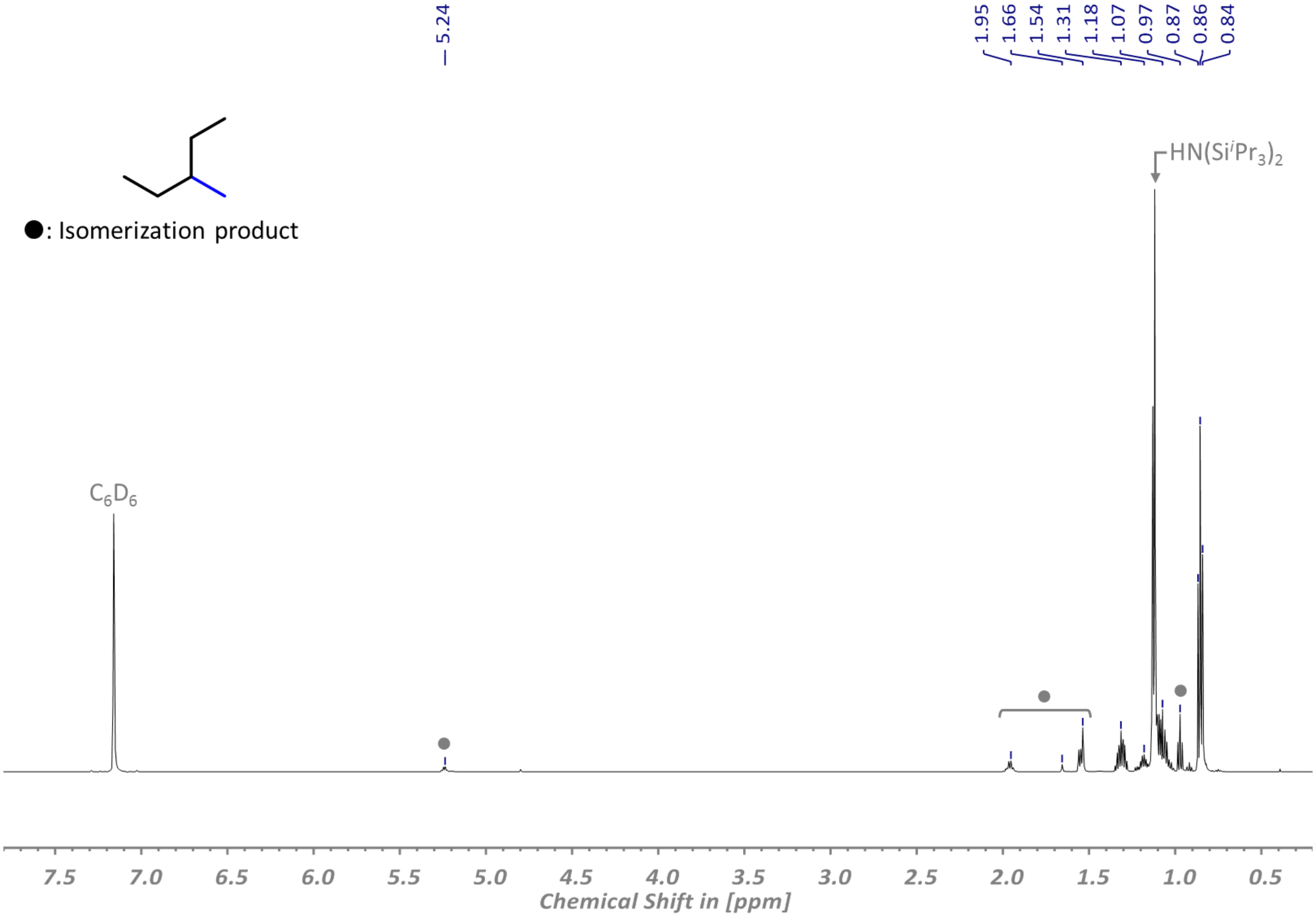

Figure S143. ^{1}H NMR spectrum (600 MHz, C_6D_6, 25 °C) after catalytic hydrogenation (24 h) of 2-ethyl-1-butene with Sr[N(Si^{i}Pr$_3$)$_2$]$_2$ (**1-Sr**) (10 mol%) and H_2 (6 bar) at 120 °C showing the formation of 2-methylpentane[S19] and additionally 3-methyl-2-pentene (●)[S20] as result of isomerization of the starting material by the catalyst (Table S3, entry 45).

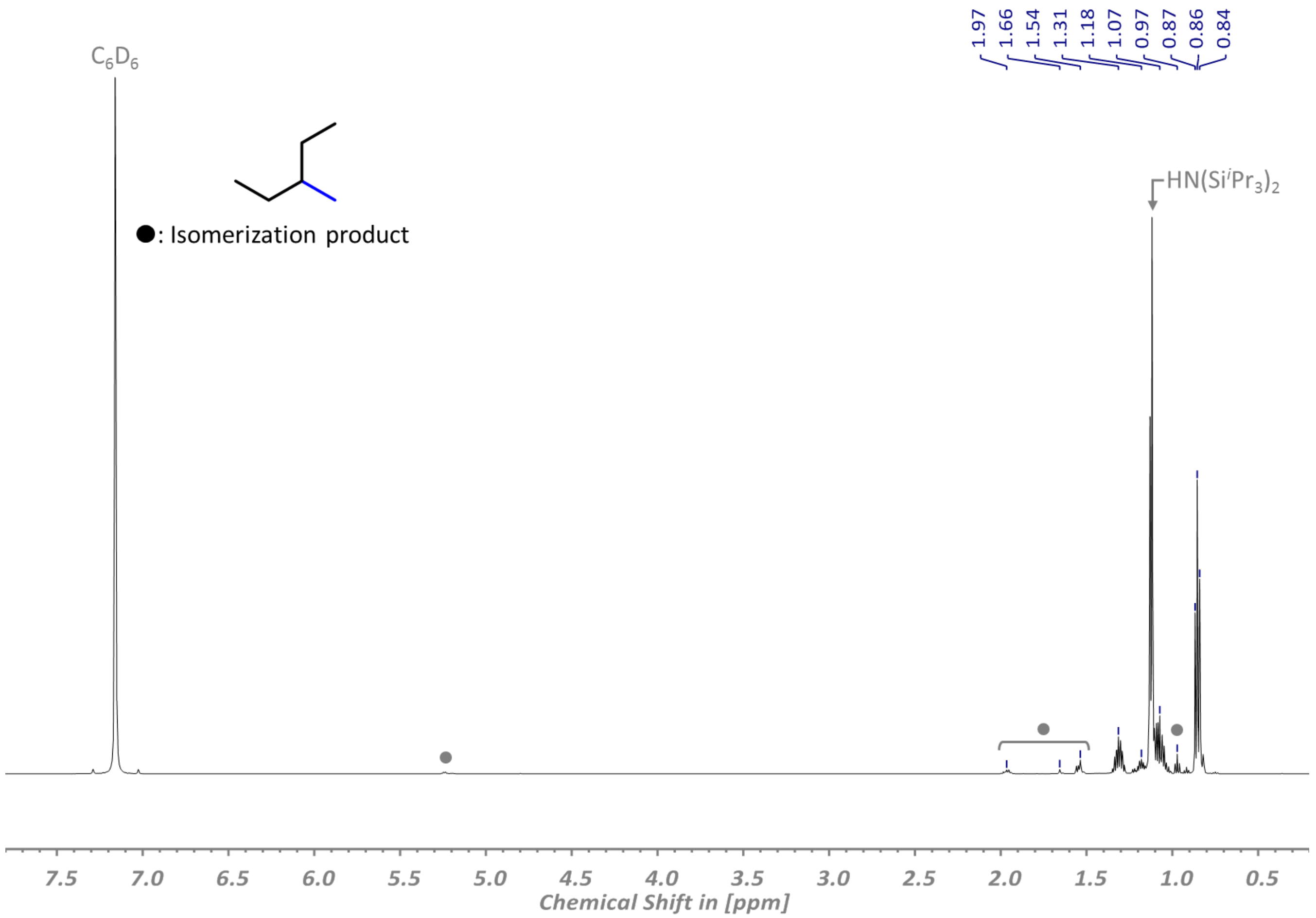

Figure S144. ^{1}H NMR spectrum (600 MHz, C_6D_6, 25 °C) after catalytic hydrogenation (24 h) of 2-ethyl-1-butene with $Ba[N(Si^iPr_3)_2]_2$ (**1-Ba**) (10 mol%) and H_2 (6 bar) at 120 °C showing the formation of 2-methylpentane and the trisubstituted alkene 3-methyl-2-pentene (●) as isomerization side product (Table S3, entry 46).

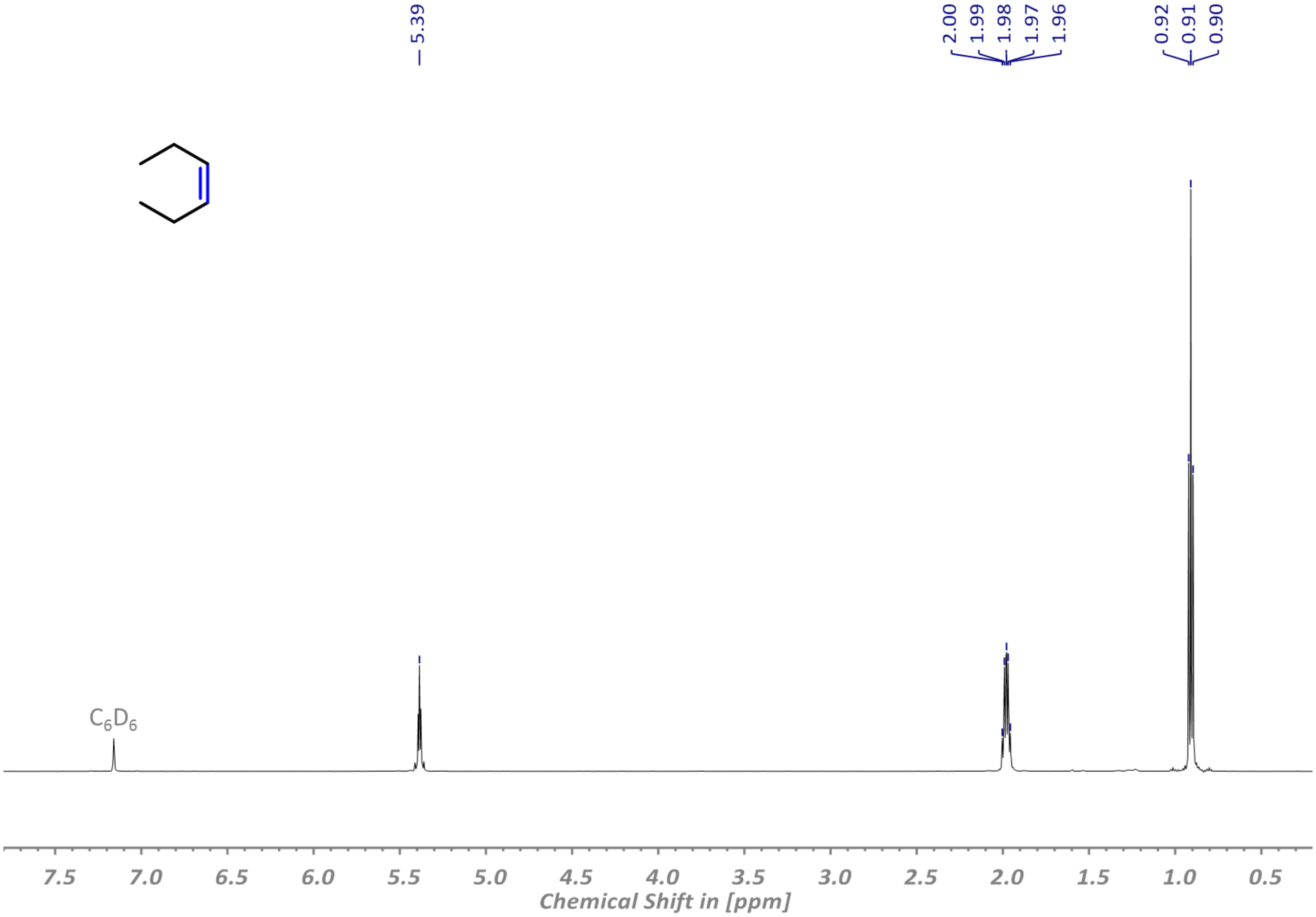

Figure S145. ^{1}H NMR spectrum (600 MHz, C_6D_6, 25 °C) of *cis*-3-hexene.

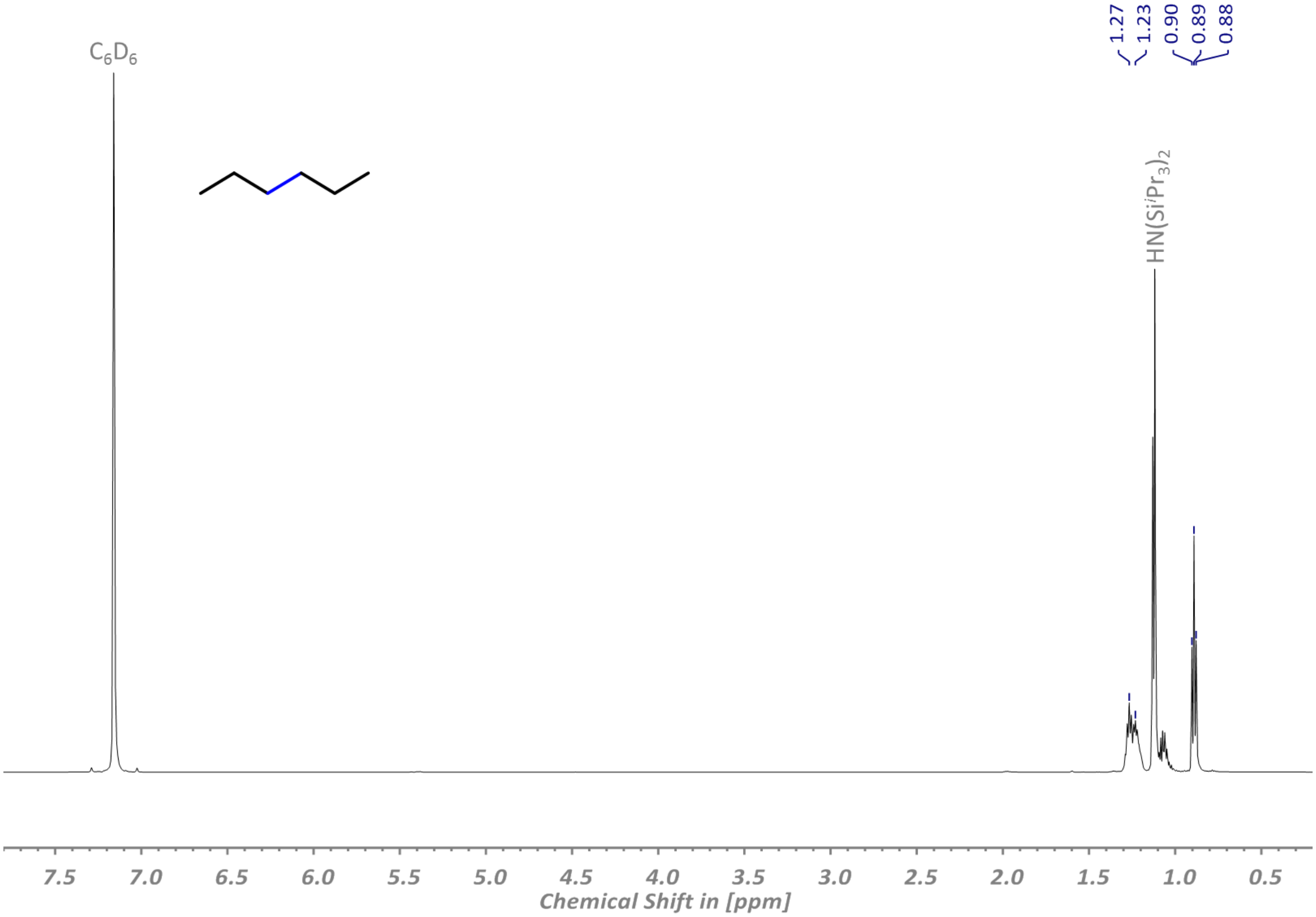

Figure S146. ^{1}H NMR spectrum (600 MHz, C_6D_6, 25 °C) of hexane after catalytic hydrogenation (7 h) of *cis*-3-hexene with $Ba[N(Si^{i}Pr_3)_2]_2$ (**1-Ba**) (10 mol%) and H_2 (6 bar) at 120 °C (Table S3, entry 47).

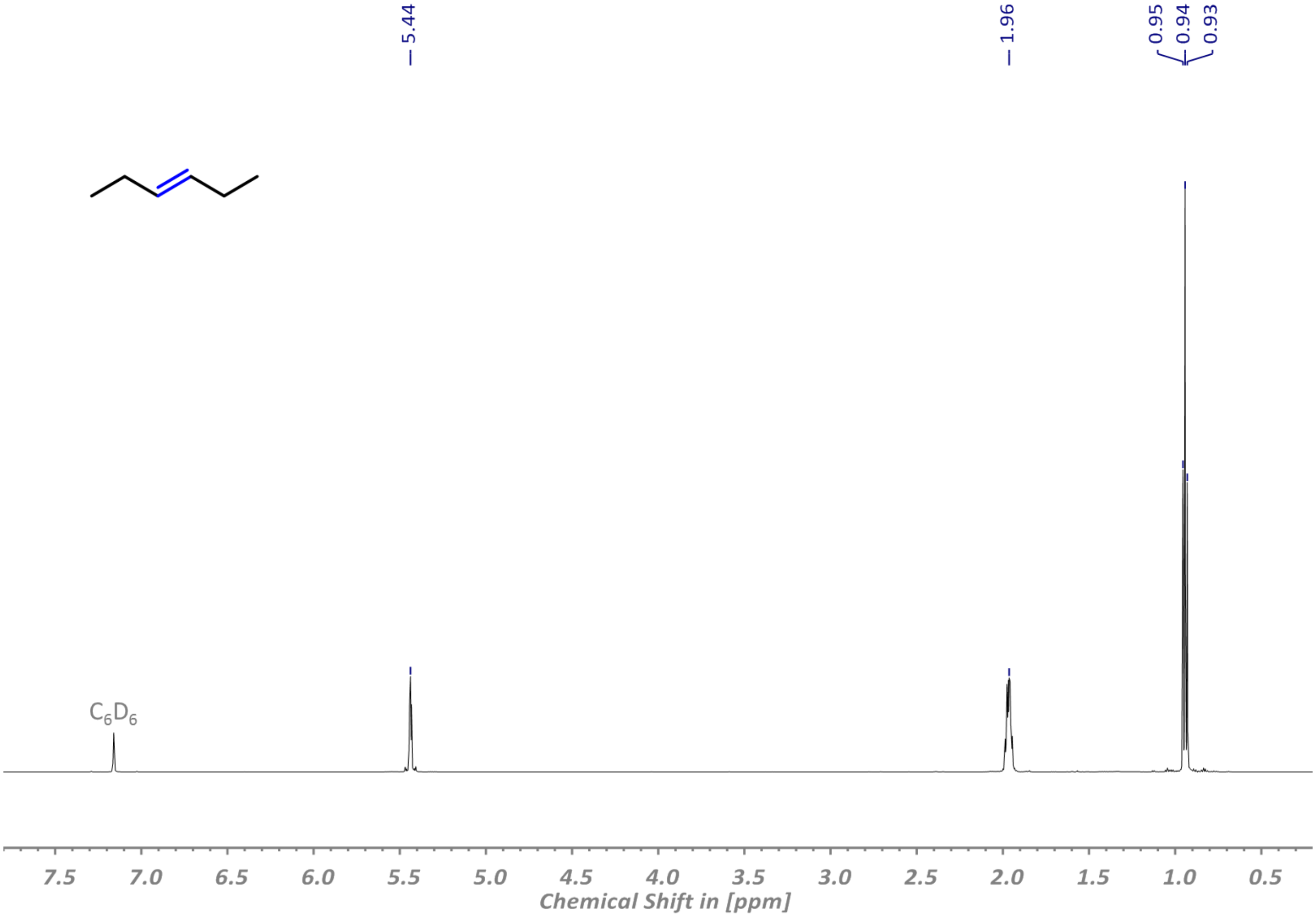

Figure S147. ^{1}H NMR spectrum (600 MHz, C_6D_6, 25 °C) of *trans*-3-hexene.

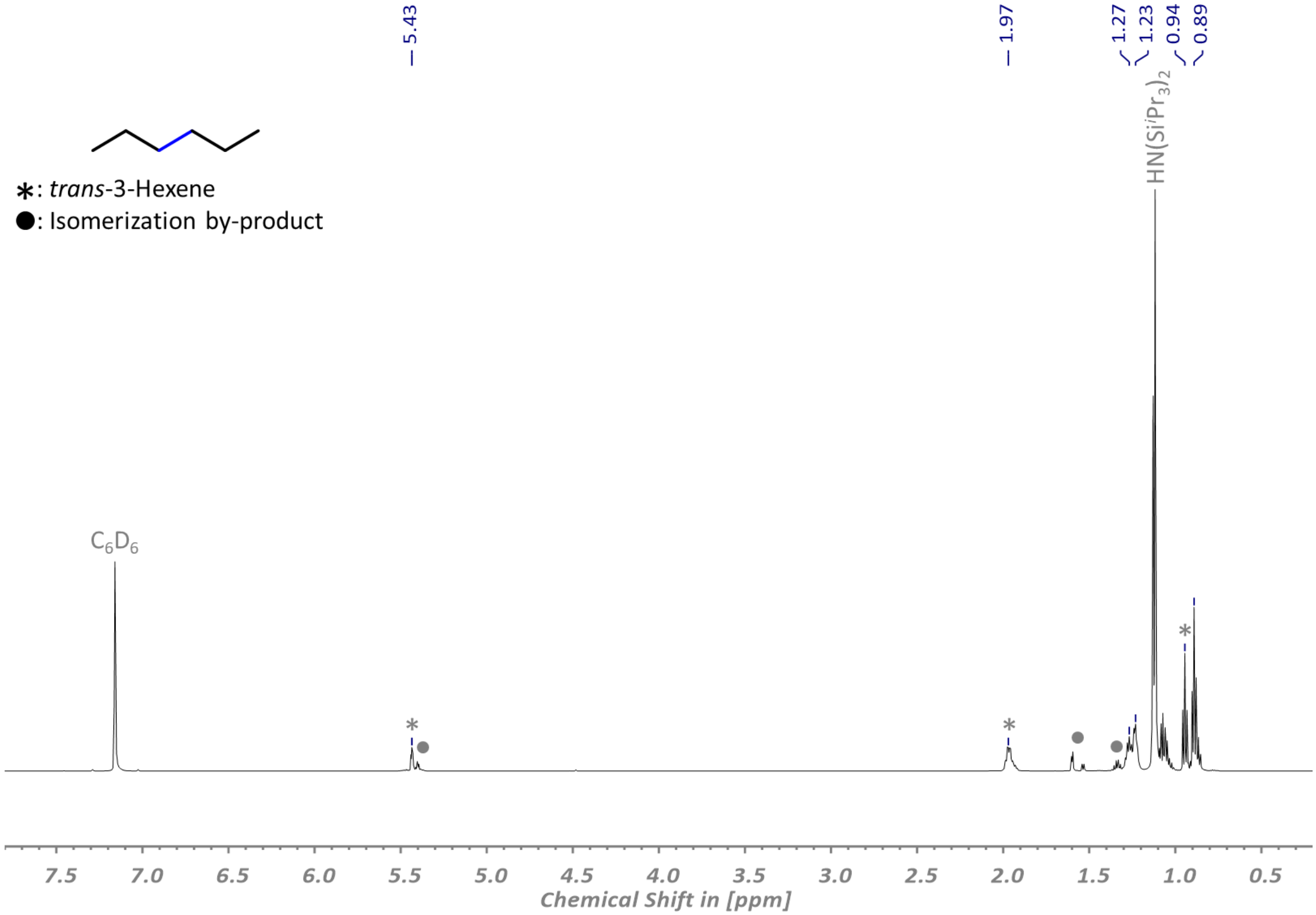

Figure S148. ^{1}H NMR spectrum (600 MHz, C_6D_6, 25 °C) after catalytic hydrogenation (24 h) of *trans*-3-hexene (∗)with Sr[N(Si^{i}Pr$_3$)$_2$]$_2$ (**1-Sr**) (10 mol%) and H_2 (6 bar) at 120 °C showing the formation of hexane and 2-hexene (●). *Note:* The latter side product is formed due to isomerization of the starting material by the catalyst (Table S3, entry 48).

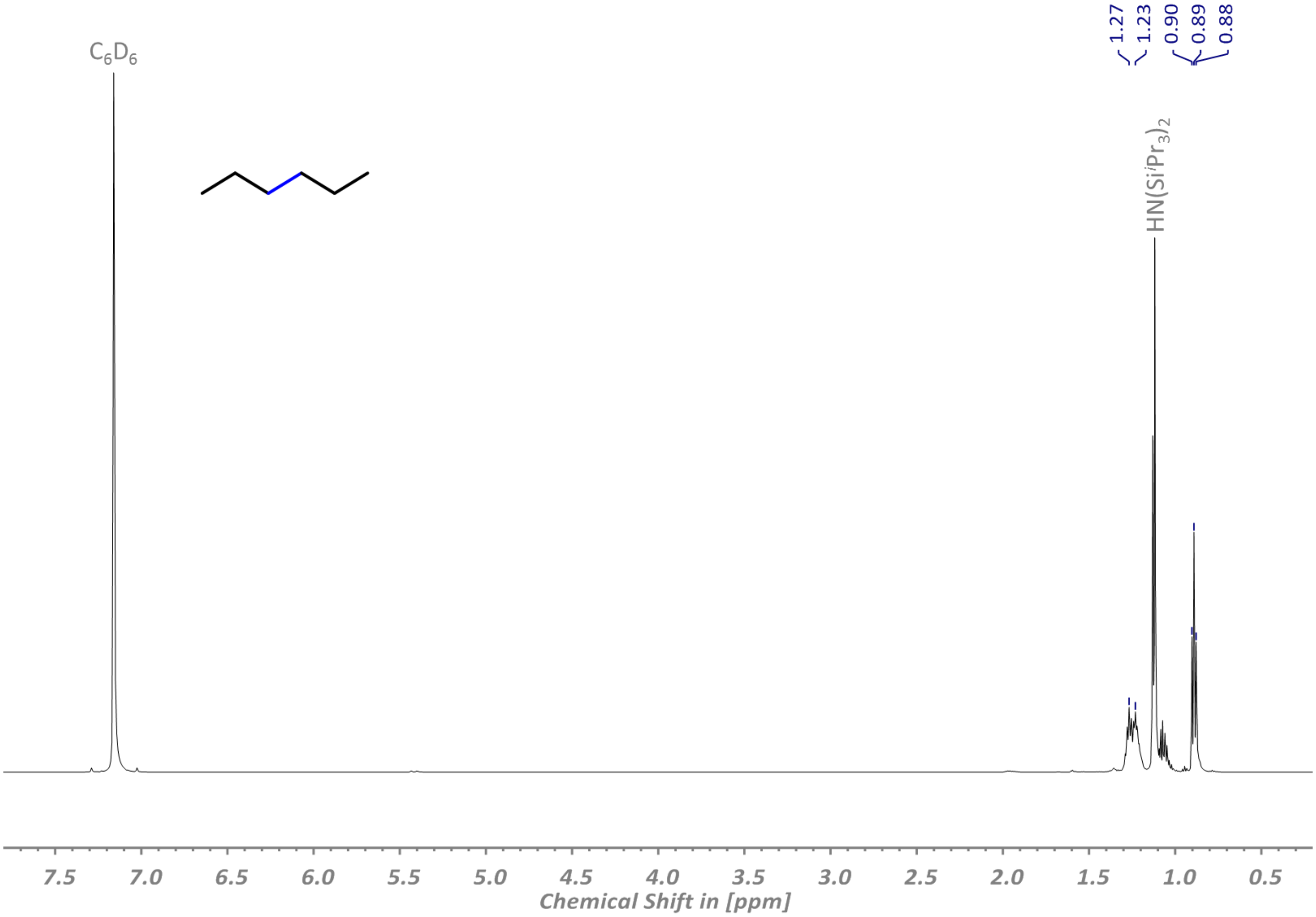

Figure S149. ^{1}H NMR spectrum (600 MHz, C_6D_6, 25 °C) of hexane after catalytic hydrogenation (22 h) of *trans*-3-hexene with Ba[N(Si^{i}Pr$_3$)$_2$]$_2$ (**1-Ba**) (10 mol%) and H_2 (6 bar) at 120 °C (Table S3, entry 49).

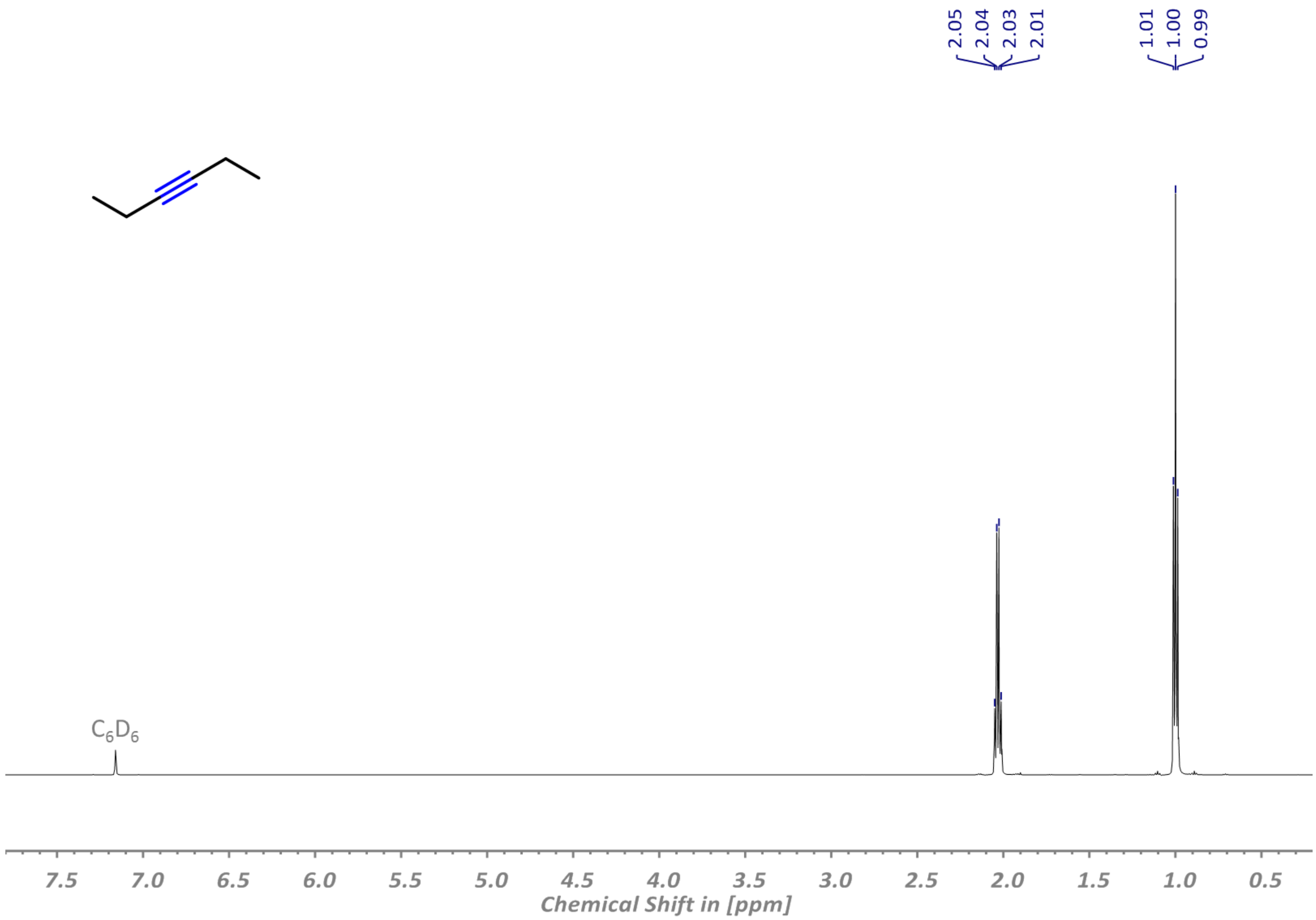

Figure S150. ^{1}H NMR spectrum (600 MHz, C_6D_6, 25 °C) of 3-hexyne.

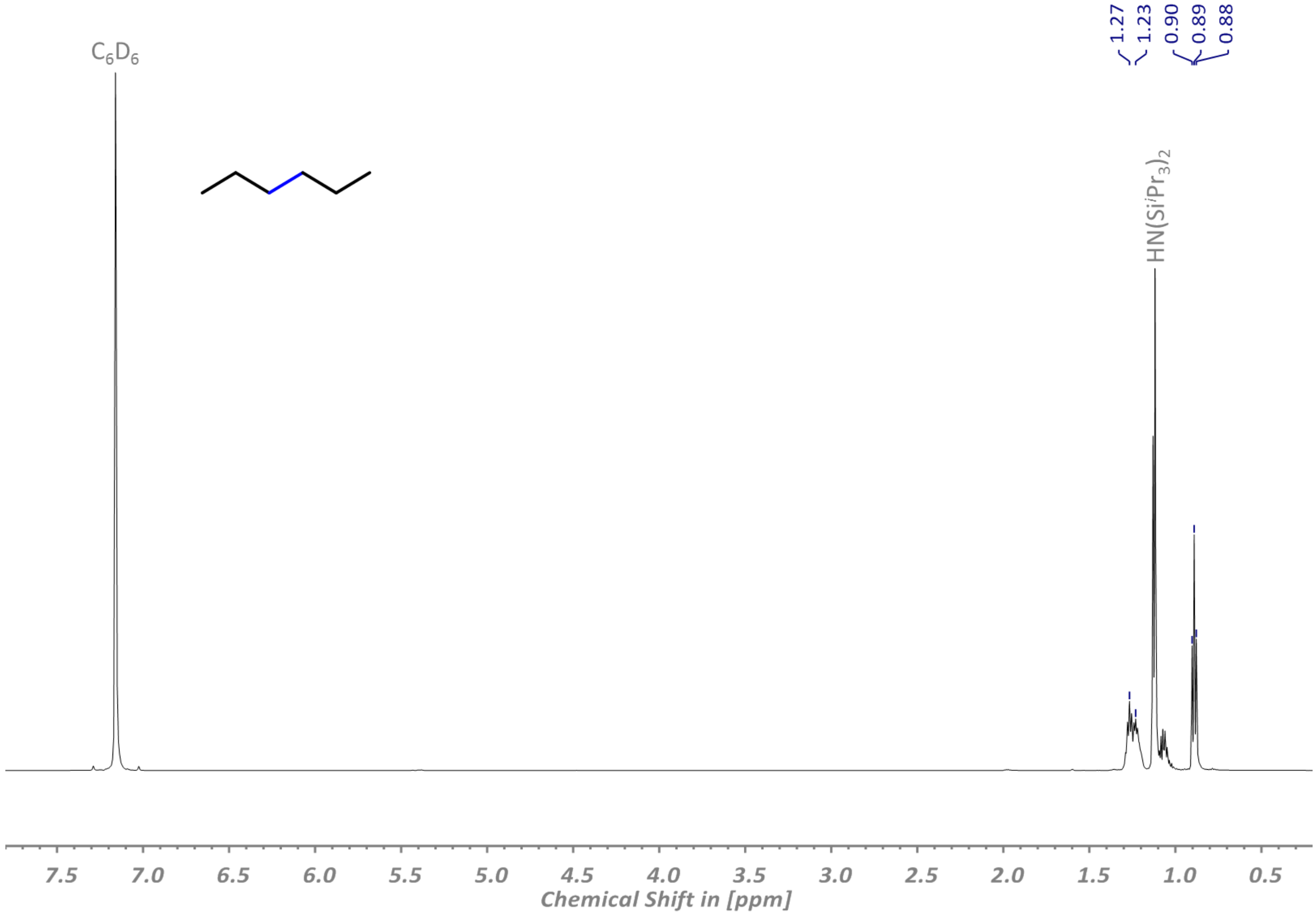

Figure S151. ^{1}H NMR spectrum (600 MHz, C_6D_6, 25 °C) of hexane after catalytic hydrogenation (24 h) of 3-hexyne with $Ba[N(Si^{i}Pr_3)_2]_2$ (**1-Ba**) (10 mol%) and H_2 (6 bar) at 120 °C (Table S3, entry 50).

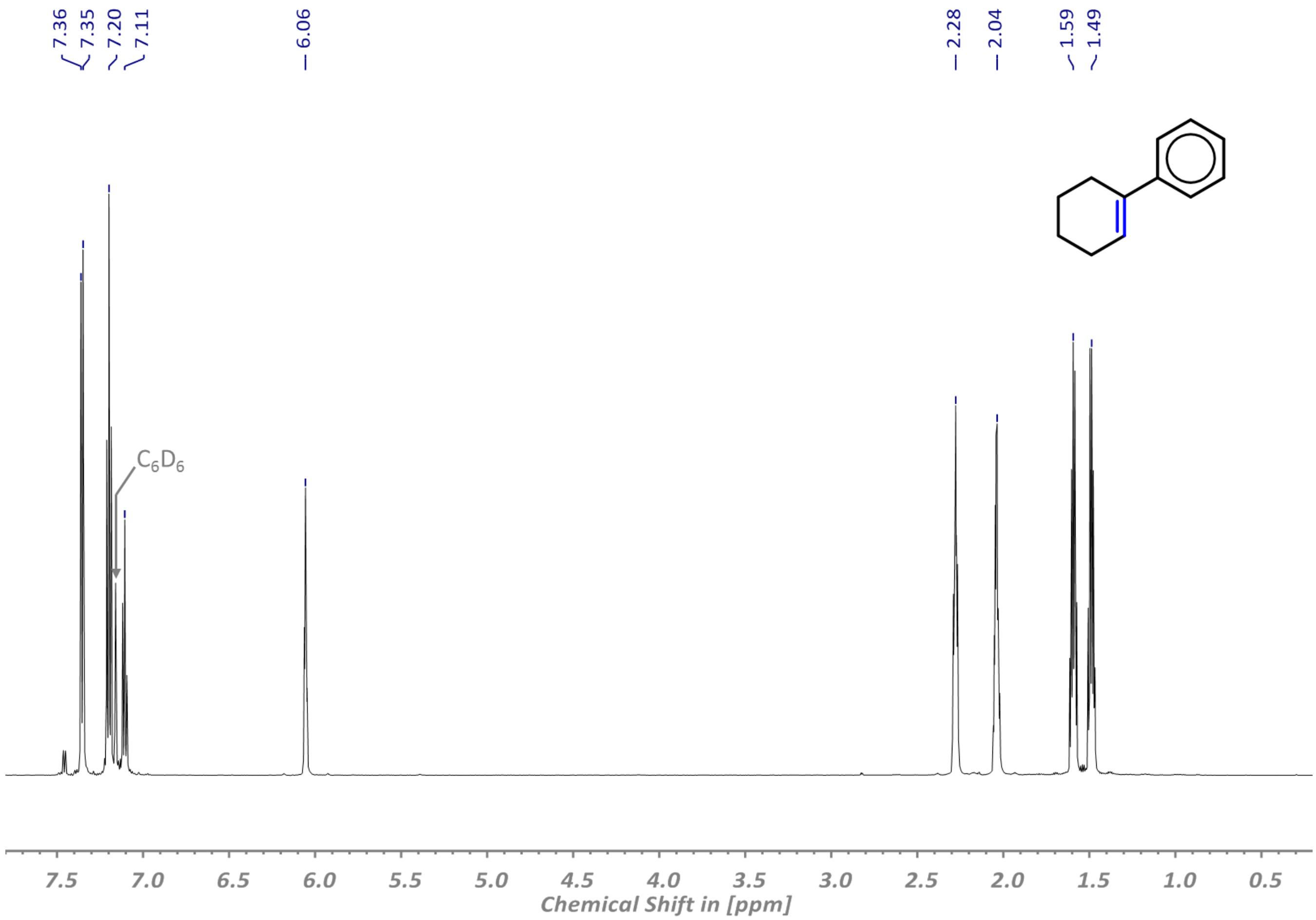

Figure S152. ^{1}H NMR spectrum (600 MHz, C_6D_6, 25 °C) of 1-phenyl-1-cyclohexene.

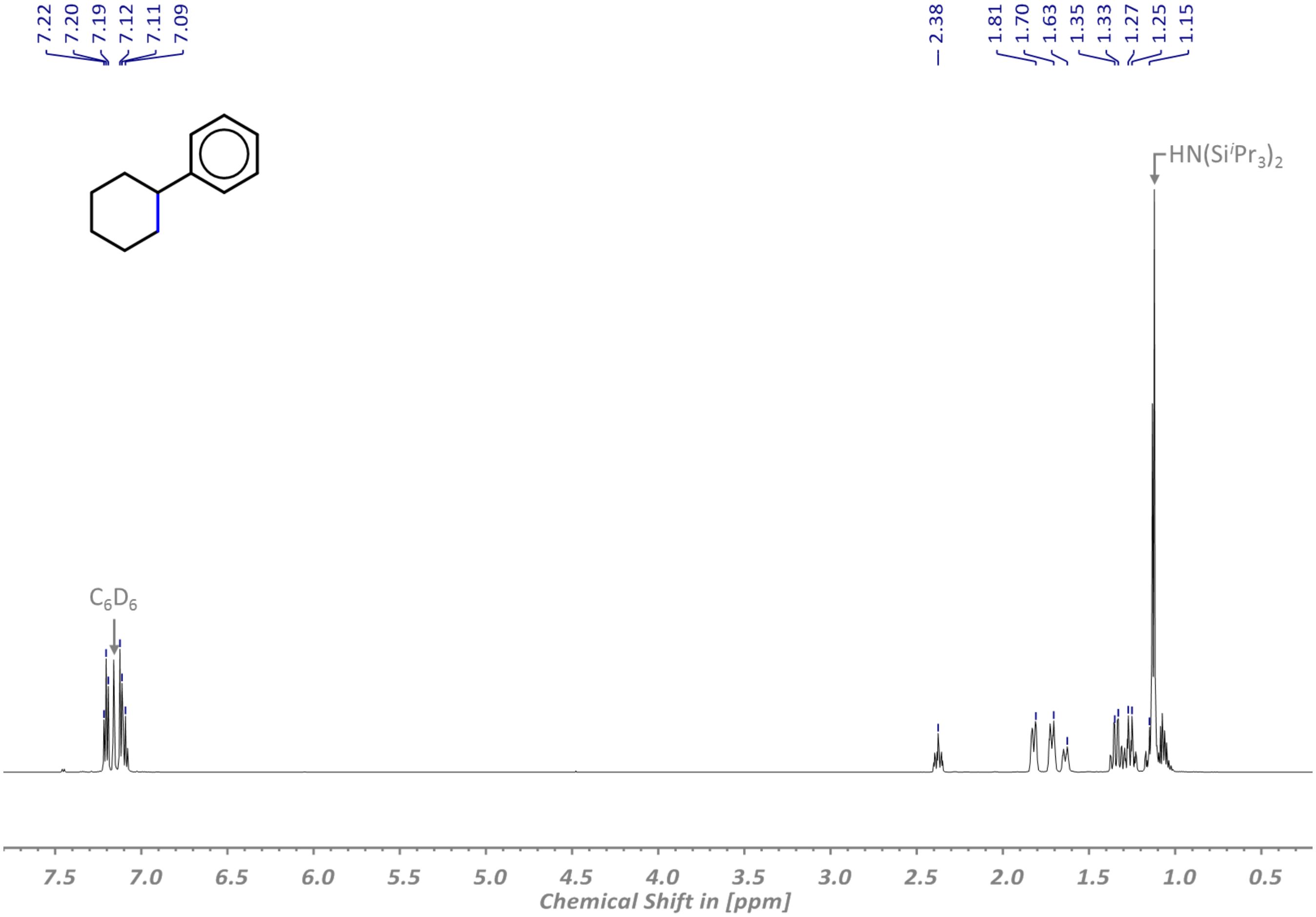

Figure S153. ^{1}H NMR spectrum (600 MHz, C_6D_6, 25 °C) of phenylcyclohexane after catalytic hydrogenation (1 h) of 1-phenyl-1-cyclohexene with Ba[N(Si^{i}Pr$_3$)$_2$]$_2$ (**1-Ba**) (10 mol%) and H_2 (6 bar) at 120 °C (Table S3, entry 51).

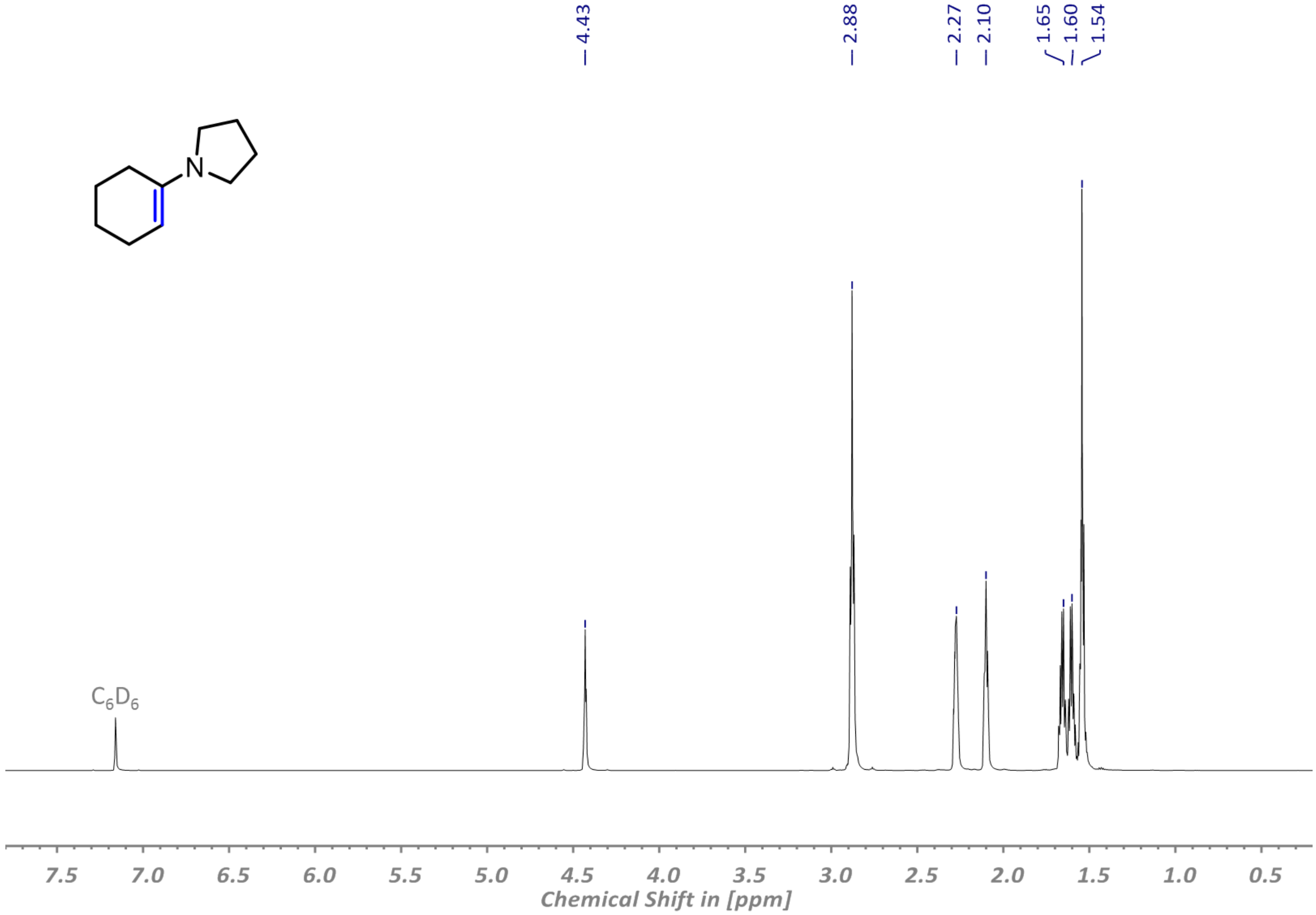

Figure S154. ^1H NMR spectrum (600 MHz, C_6D_6, 25 °C) of 1-pyrrolidino-1-cyclohexene.[S7]

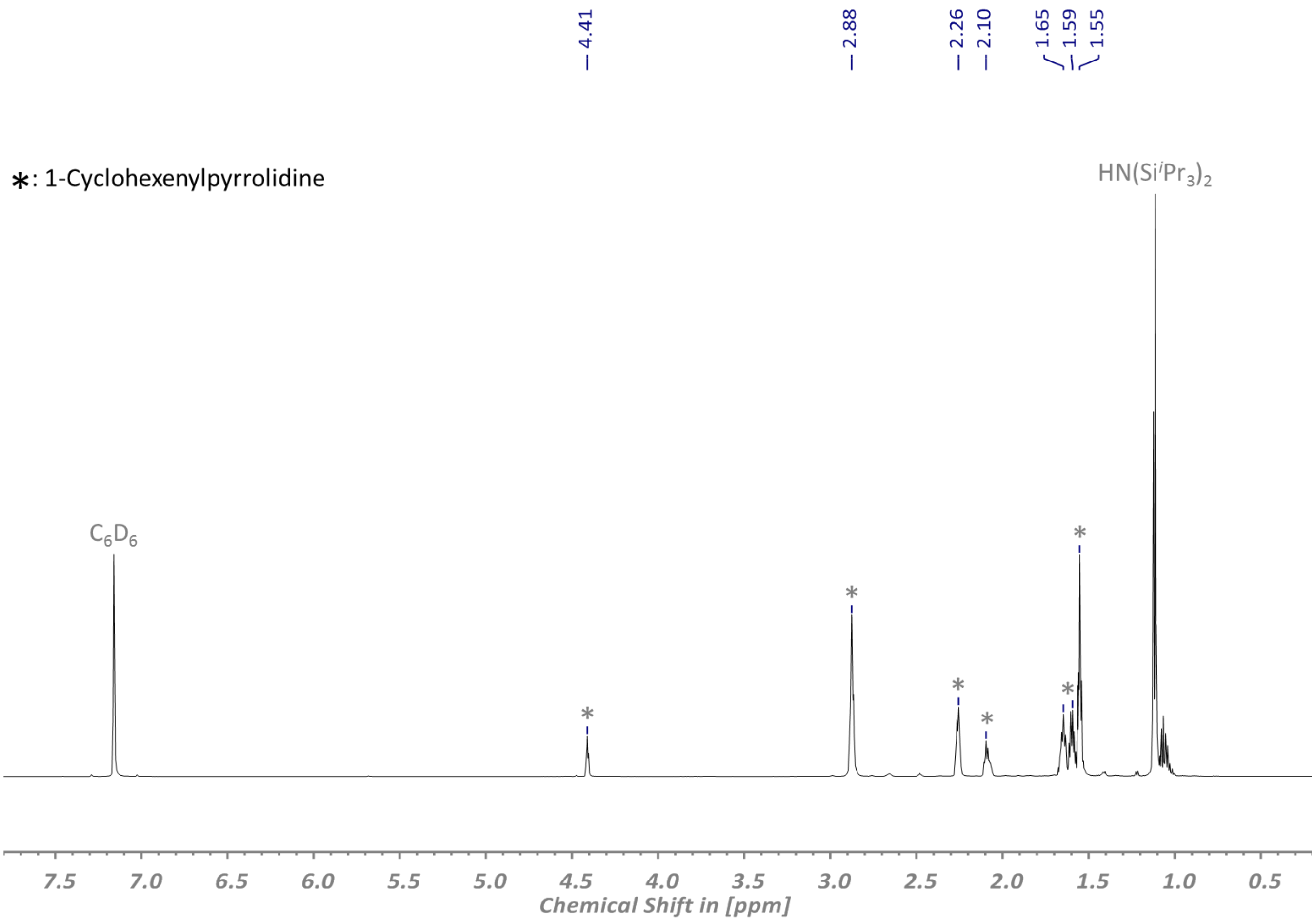

Figure S155. ^{1}H NMR spectrum (600 MHz, C_6D_6, 25 °C) after the reaction (24 h) of the enamine 1-pyrrolidino-1-cyclohexene (∗) with Ba[N(Si^{i}Pr$_3$)$_2$]$_2$ (**1-Ba**) (10 mol%) and H_2 (6 bar) at 120 °C. *Note:* No formation of *N*-cyclohexylpyrrolidine is observed (Table S3, entry 52).

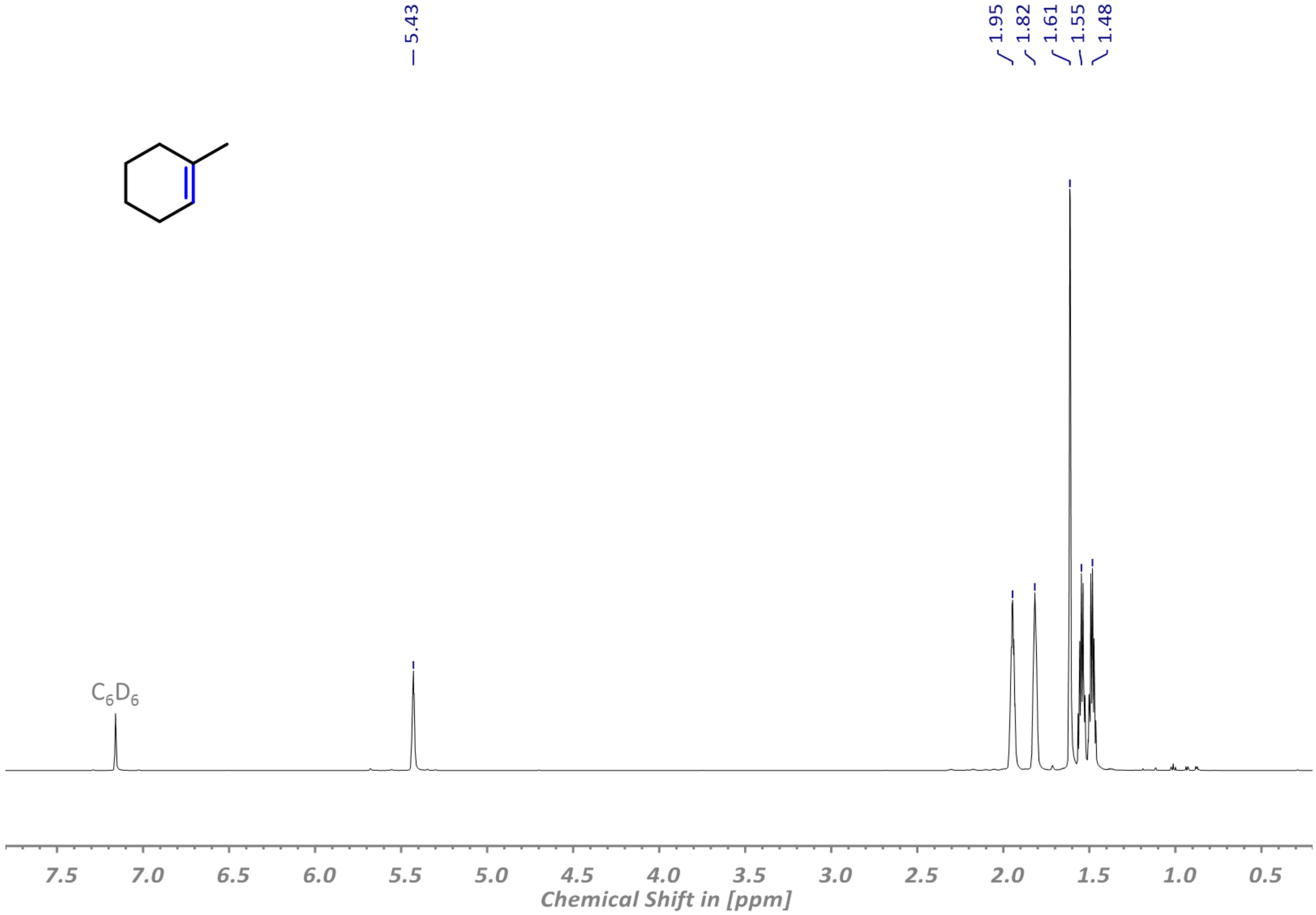

Figure S156. ^{1}H NMR spectrum (600 MHz, C_6D_6, 25 °C) of 1-methyl-1-cyclohexene.

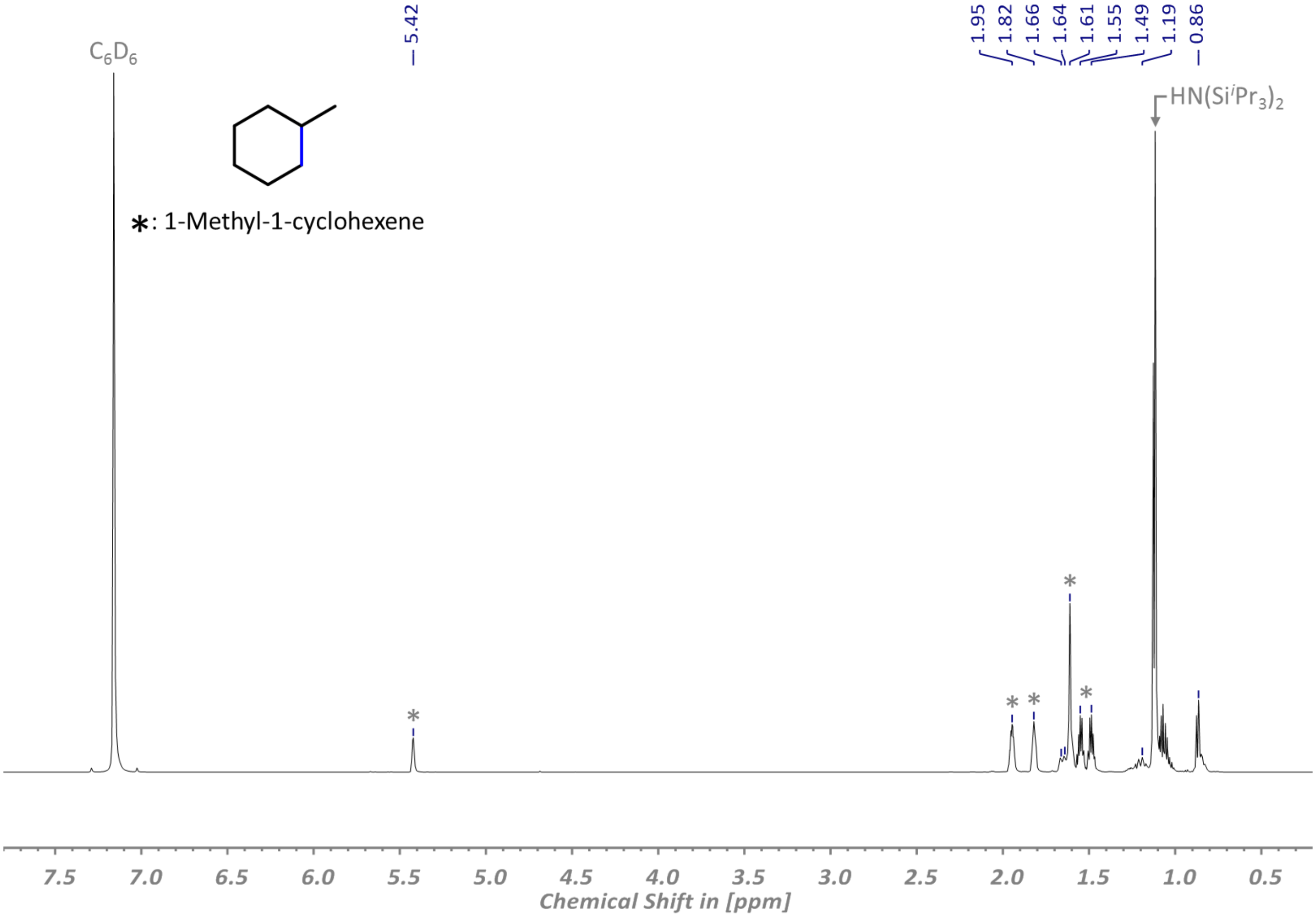

Figure S157. ^{1}H NMR spectrum (600 MHz, C_6D_6, 25 °C) after catalytic hydrogenation (24 h) of 1-methyl-1-cyclohexene (∗) with $Sr[N(Si^{i}Pr_3)_2]_2$ (**1-Sr**) (10 mol%) and H_2 (6 bar) at 120 °C showing the formation of methylcyclohexane (Table S3, entry 53).

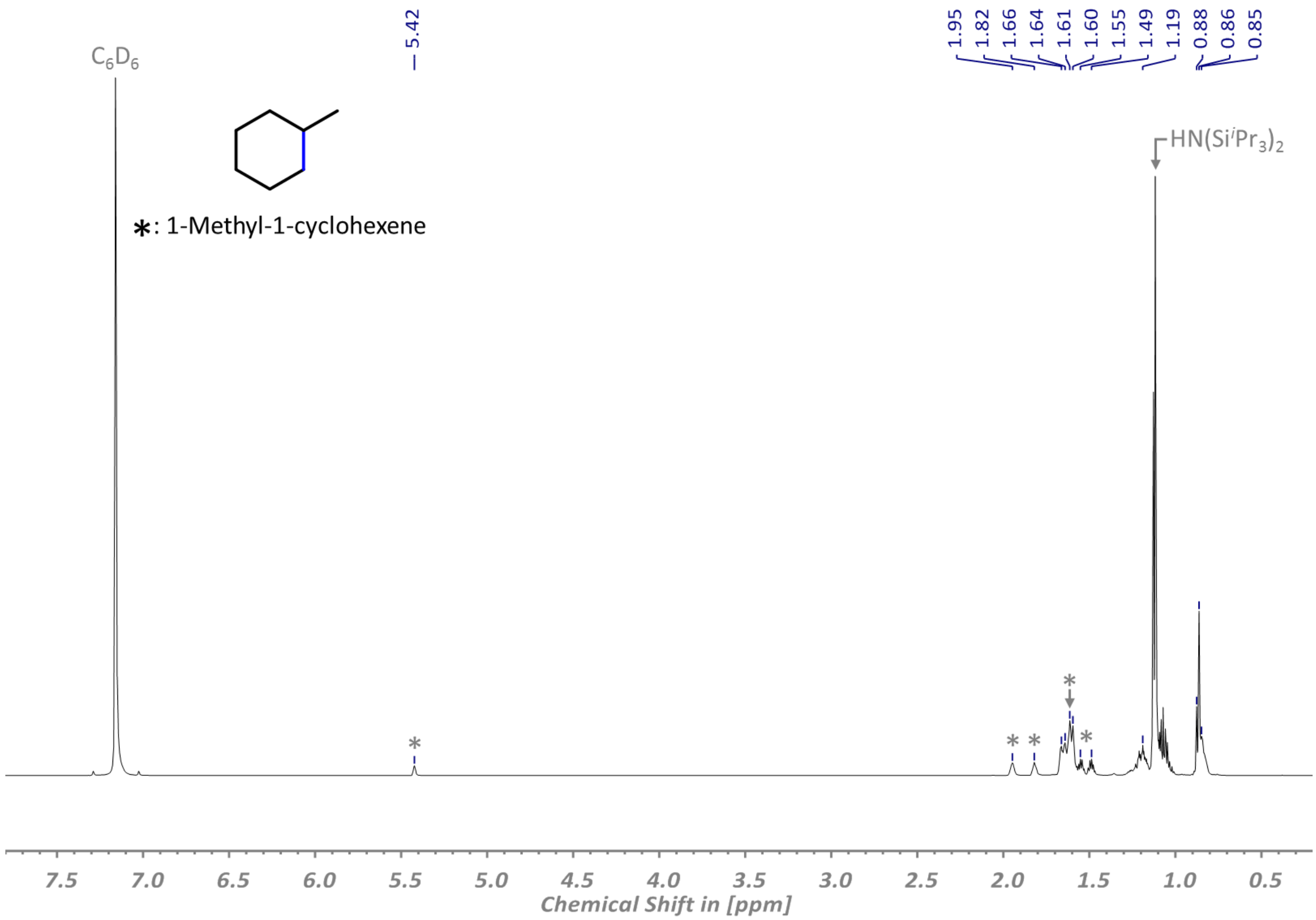

Figure S158. ^{1}H NMR spectrum (600 MHz, C_6D_6, 25 °C) after catalytic hydrogenation (24 h) of 1-methyl-1-cyclohexene (∗) with $Ba[N(Si^iPr_3)_2]_2$ (**1-Ba**) (10 mol%) and H_2 (6 bar) at 120 °C showing the formation of methylcyclohexane (Table S3, entry 54).

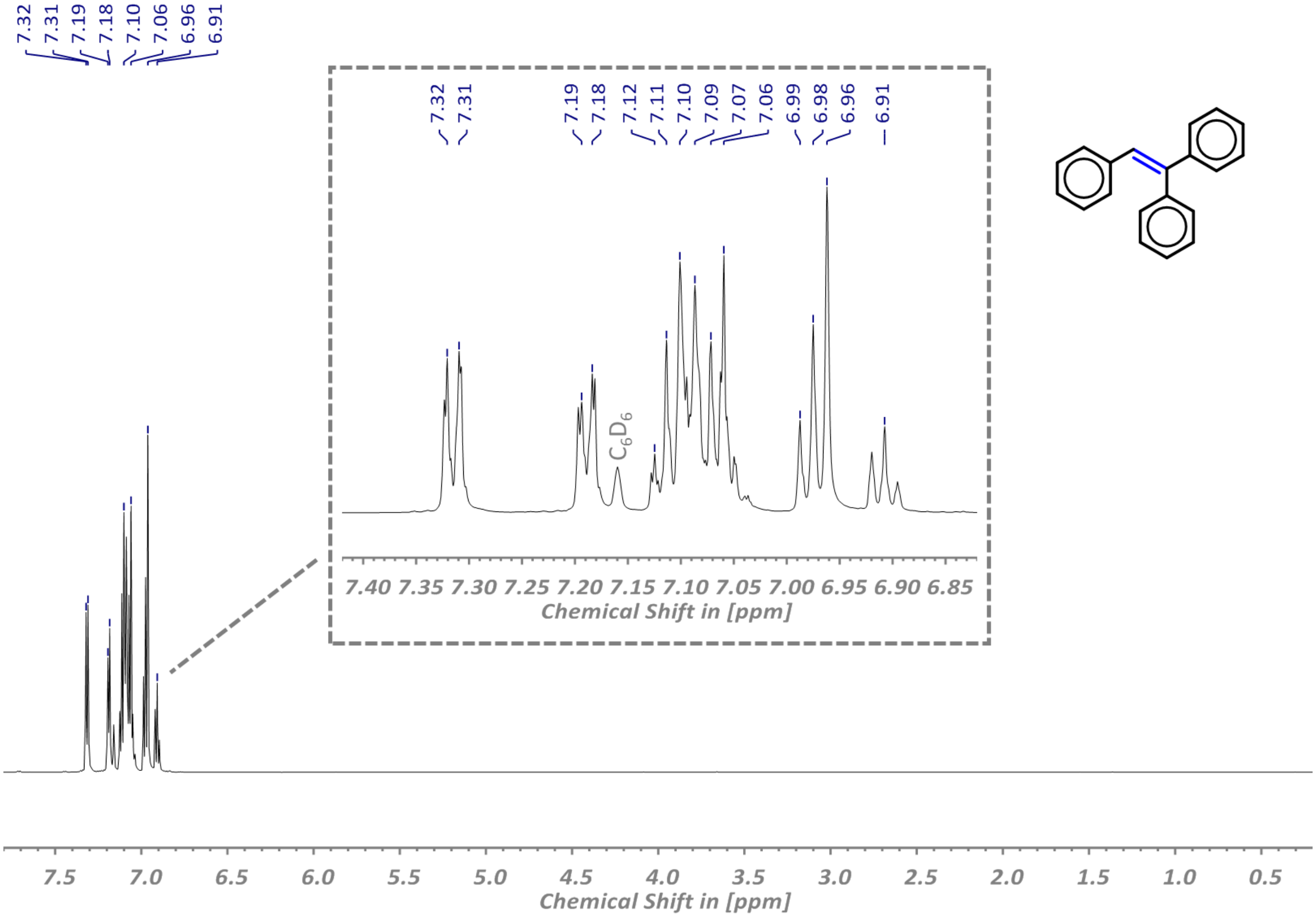

Figure S159. ^1H NMR spectrum (600 MHz, C_6D_6, 25 °C) of triphenylethylene.

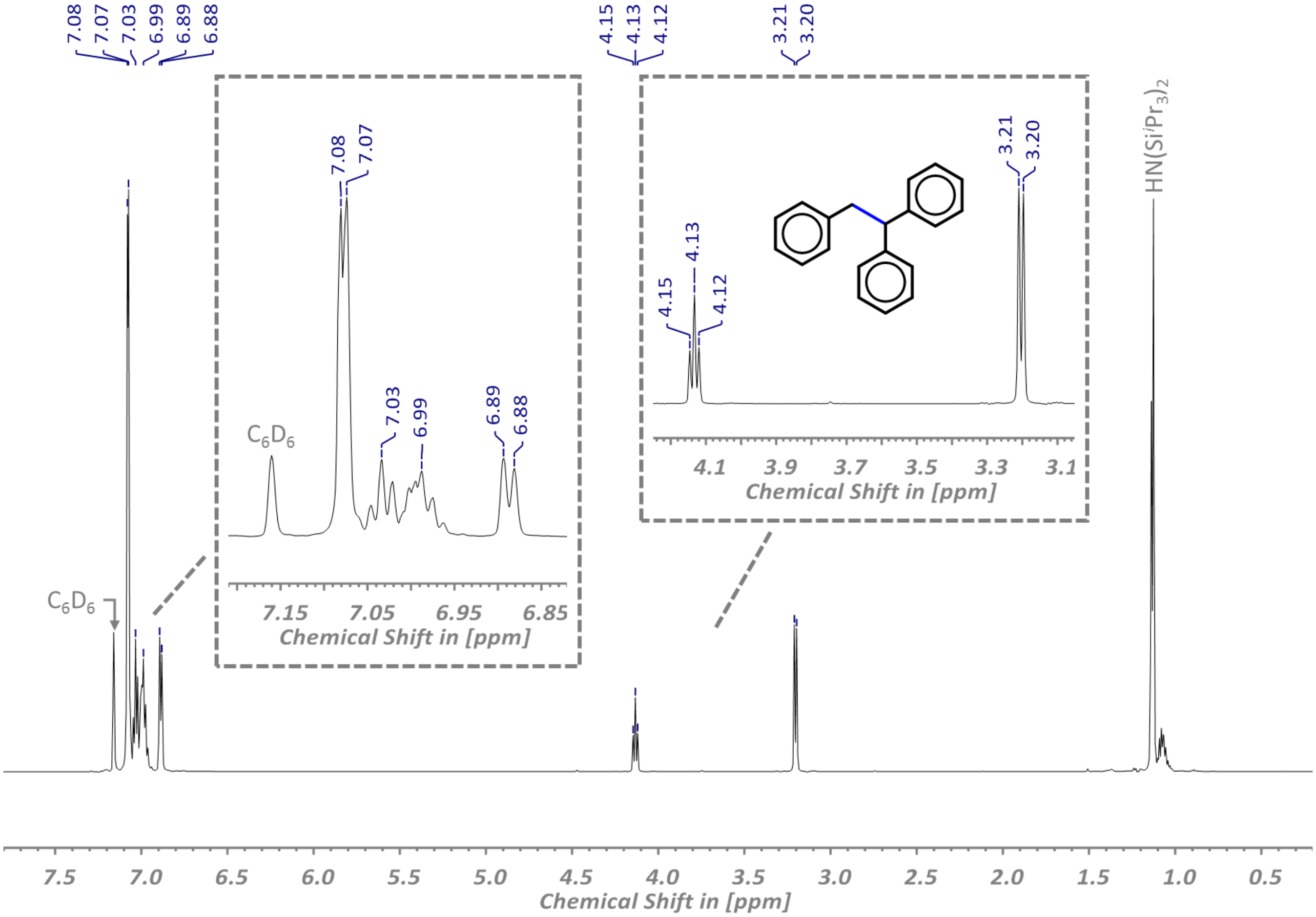

Figure S160. ^{1}H NMR spectrum (600 MHz, C_6D_6, 25 °C) of 1,1,2-triphenylethane after catalytic hydrogenation (18 h) of triphenylethylene with Ca[N(Si^{i}Pr$_3$)$_2$]$_2$ (**1-Ca**) (10 mol%) and H_2 (6 bar) at 120 °C (Table S3, entry 55).

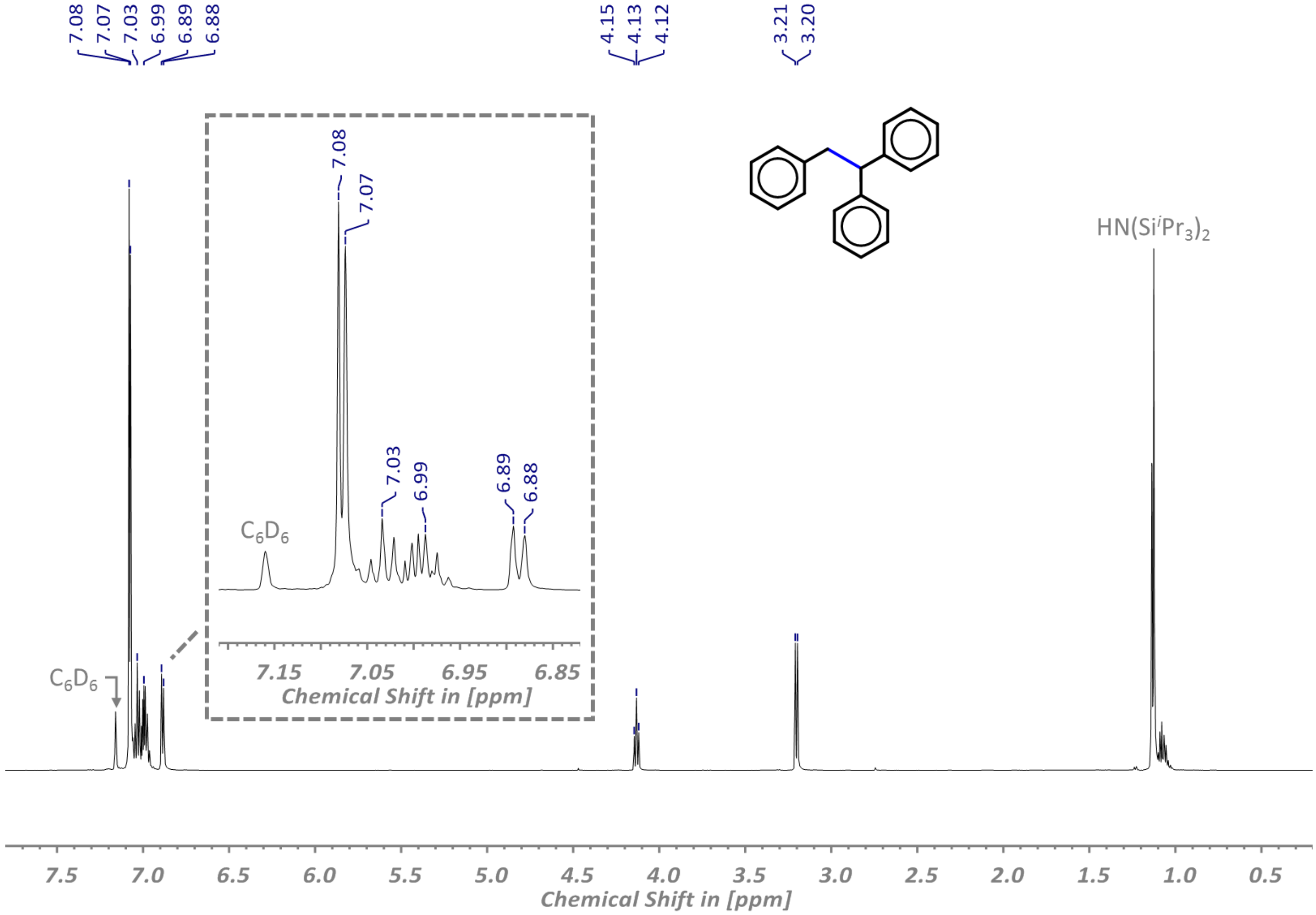

Figure S161. ^{1}H NMR spectrum (600 MHz, C_6D_6, 25 °C) of 1,1,2-triphenylethane after catalytic hydrogenation (1 h) of triphenylethylene with Ba[N(Si^{i}Pr$_3$)$_2$]$_2$ (**1-Ba**) (10 mol%) and H_2 (6 bar) at 120 °C (Table S3, entry 56).

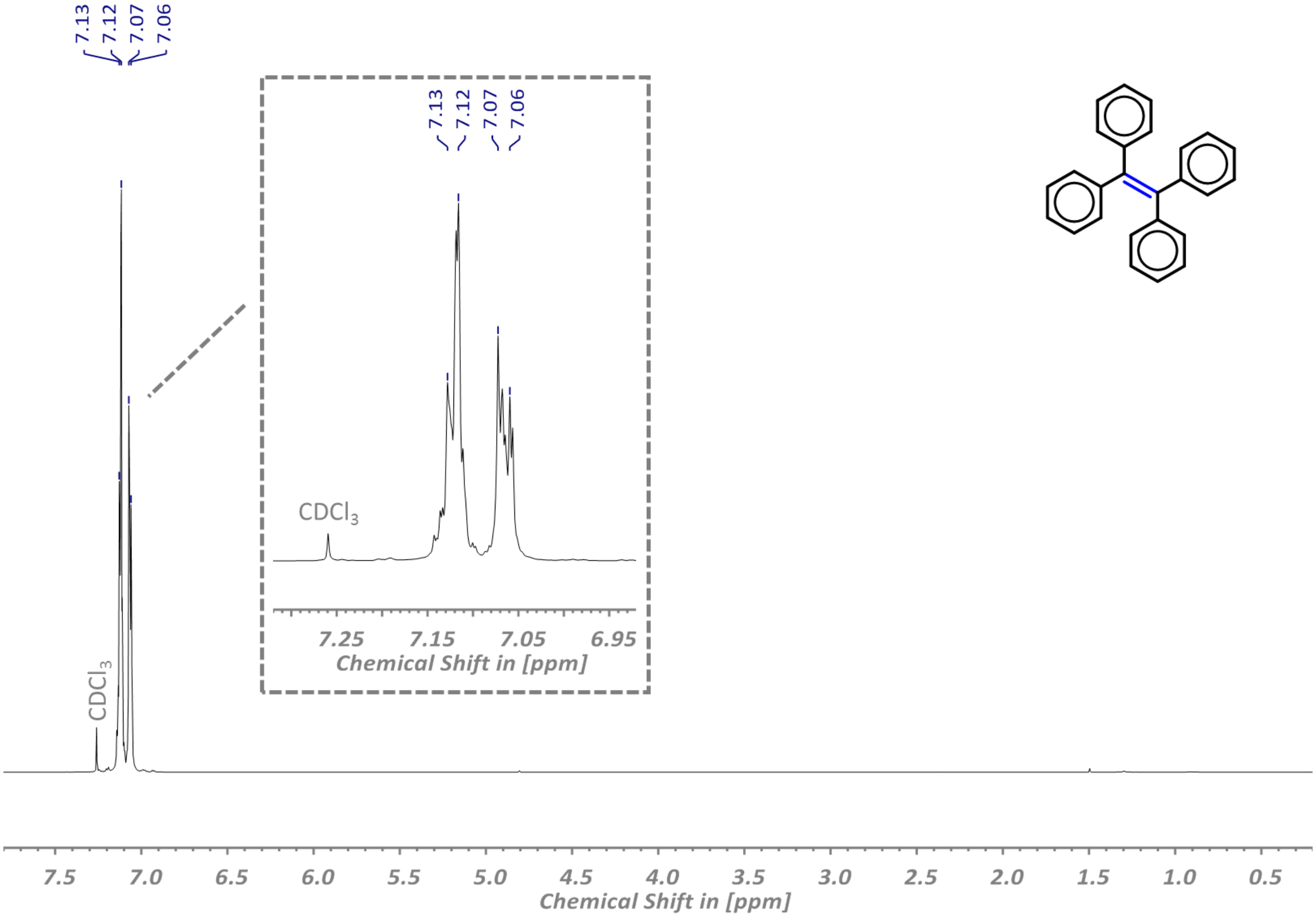

Figure S162. ^{1}H NMR spectrum (600 MHz, $CDCl_3$, 25 °C) of tetraphenylethylene.

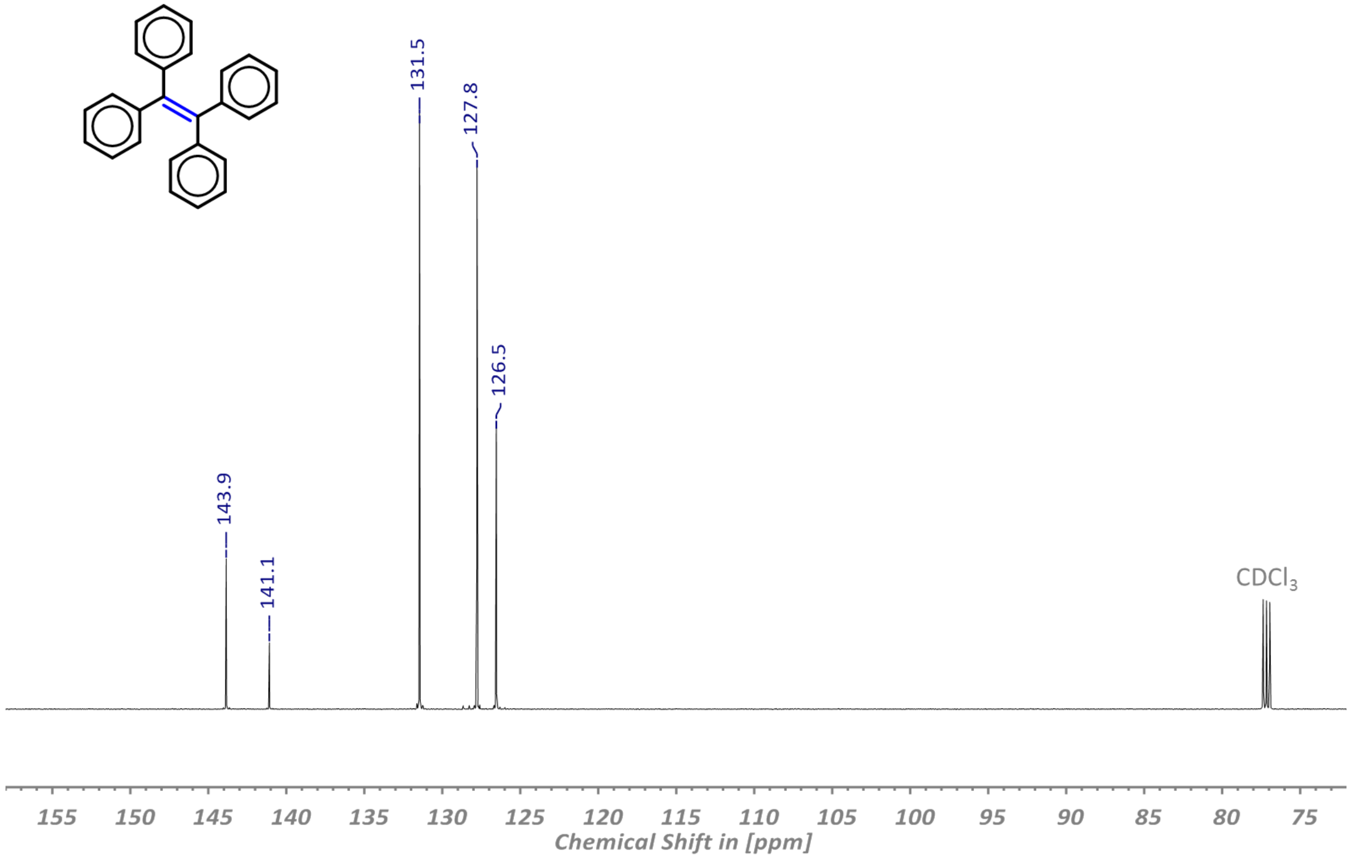

Figure S163. $^{13}C\{^1H\}$ NMR spectrum (151 MHz, $CDCl_3$, 25 °C) of tetraphenylethylene.

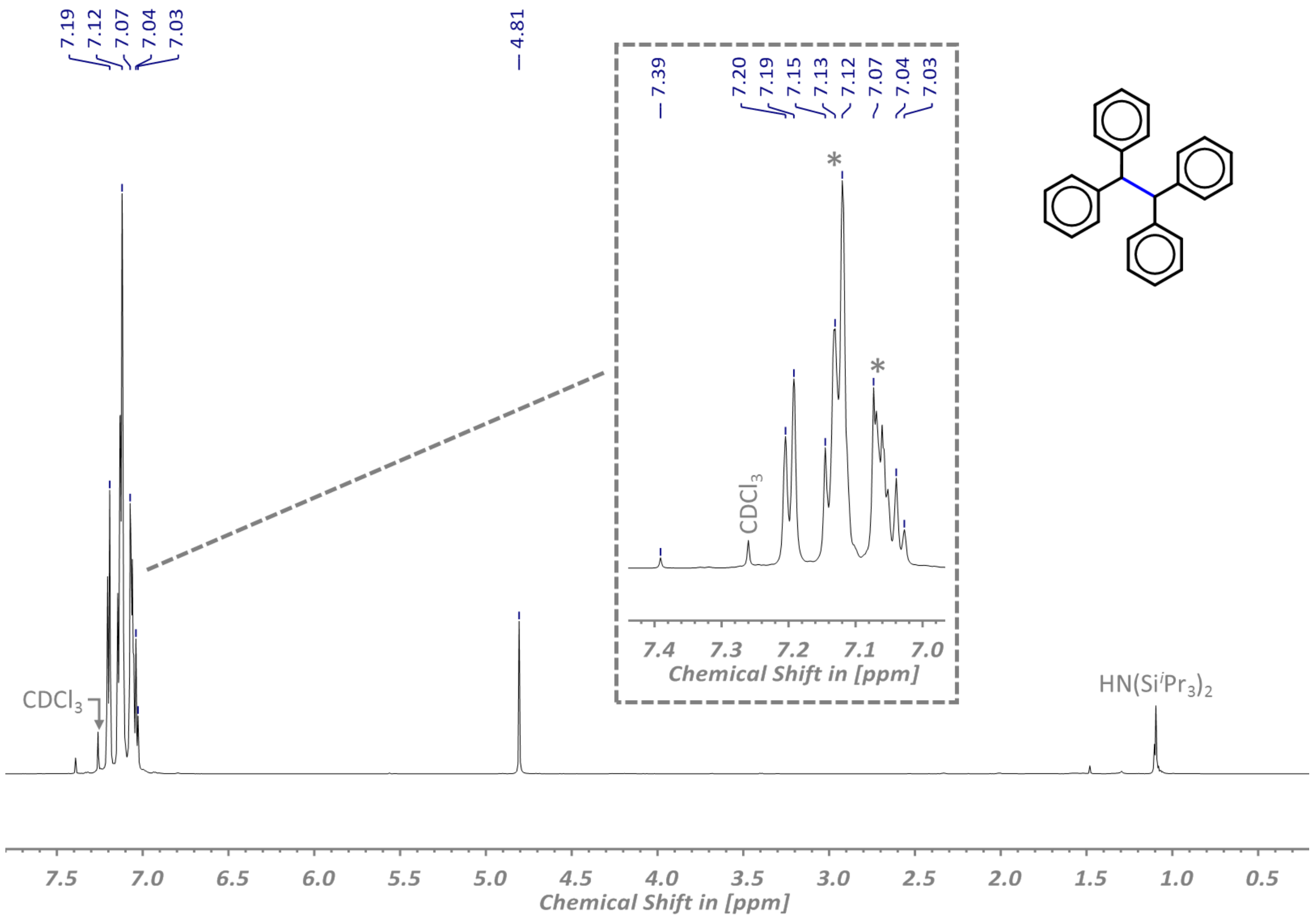

Figure S164. ^{1}H NMR spectrum (600 MHz, $CDCl_3$, 25 °C) after catalytic hydrogenation (24 h) of tetraphenylethylene (∗) with $Ba[N(Si^{i}Pr_3)_2]_2$ (**1-Ba**) (10 mol%) and H_2 (6 bar) at 120 °C showing the formation of 1,1,2,2-tetraphenylethane. *Note:* The exact conversion was determined from a further independent experiment by GC/MS analysis (Table S3, entry 57).

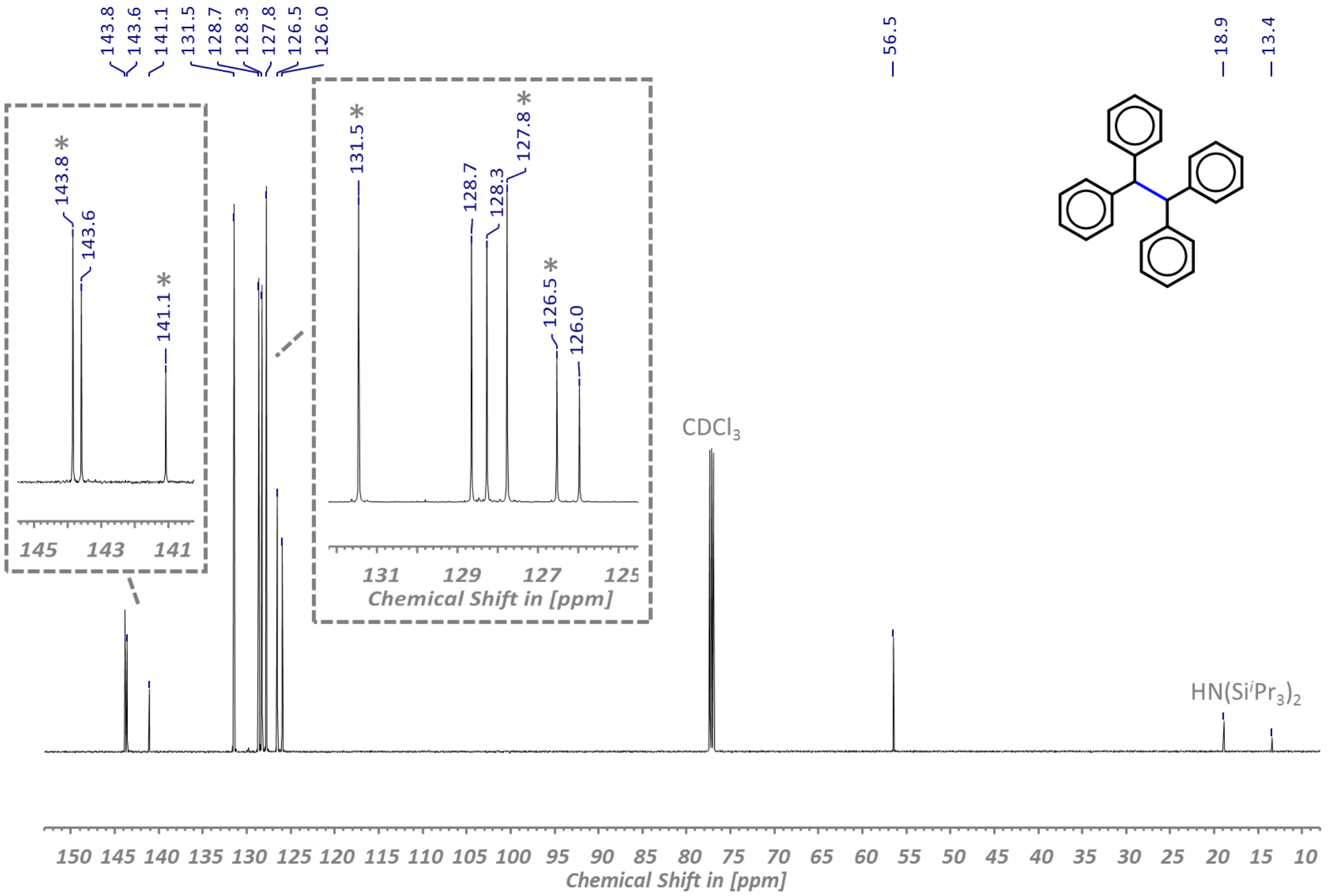

Figure S165. $^{13}C\{^{1}H\}$ NMR spectrum (151 MHz, $CDCl_3$, 25 °C) after catalytic hydrogenation (24 h) of tetraphenylethylene (∗) with $Ba[N(Si^{i}Pr_3)_2]_2$ (**1-Ba**) (10 mol%) and H_2 (6 bar) at 120 °C showing the formation of 1,1,2,2-tetraphenylethane.[S21] *Note:* The exact conversion was determined from a further independent experiment by GC/MS analysis (Table S3, entry 57).

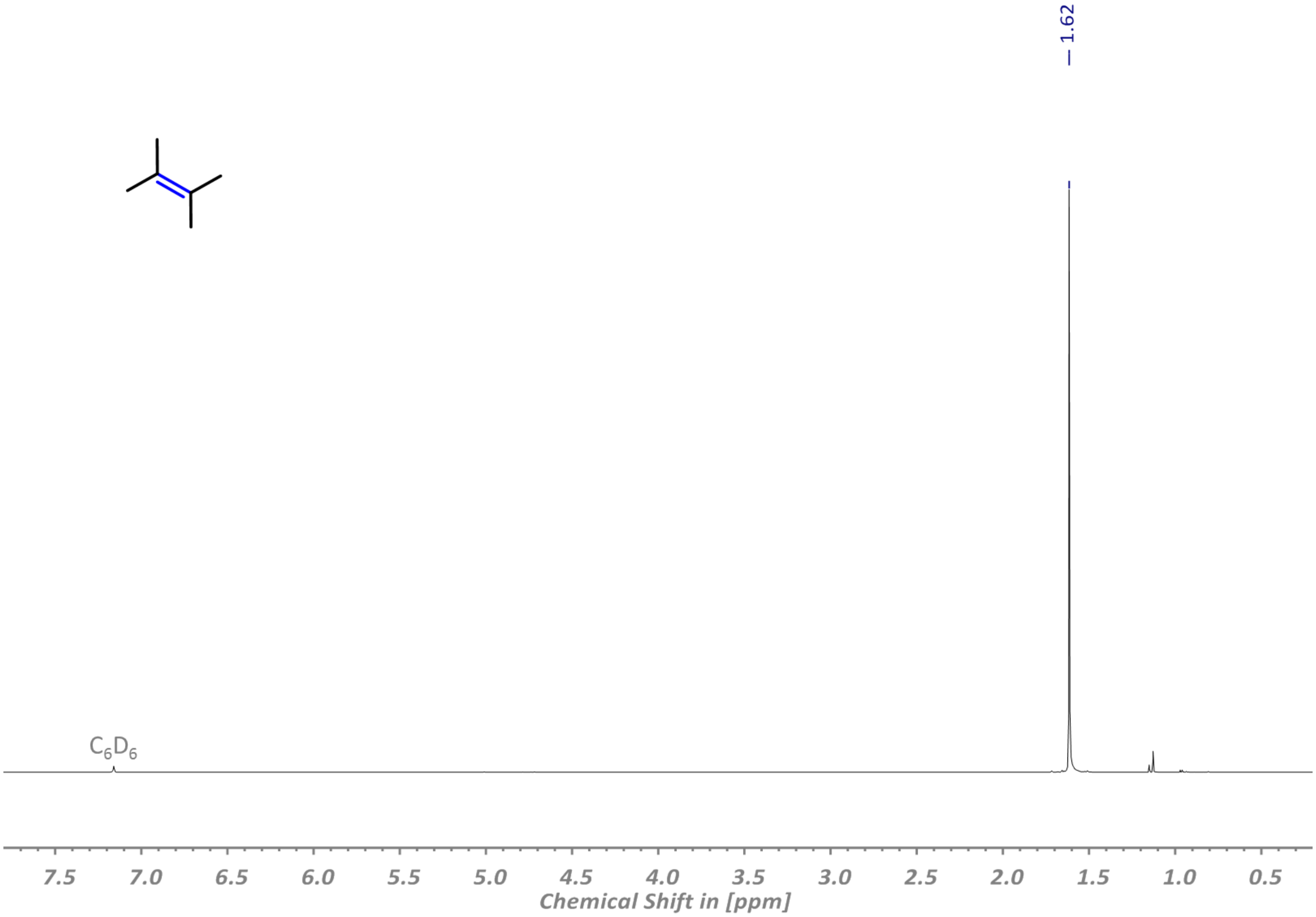

Figure S166. ^{1}H NMR spectrum (600 MHz, C_6D_6, 25 °C) of 2,3-dimethyl-2-butene.

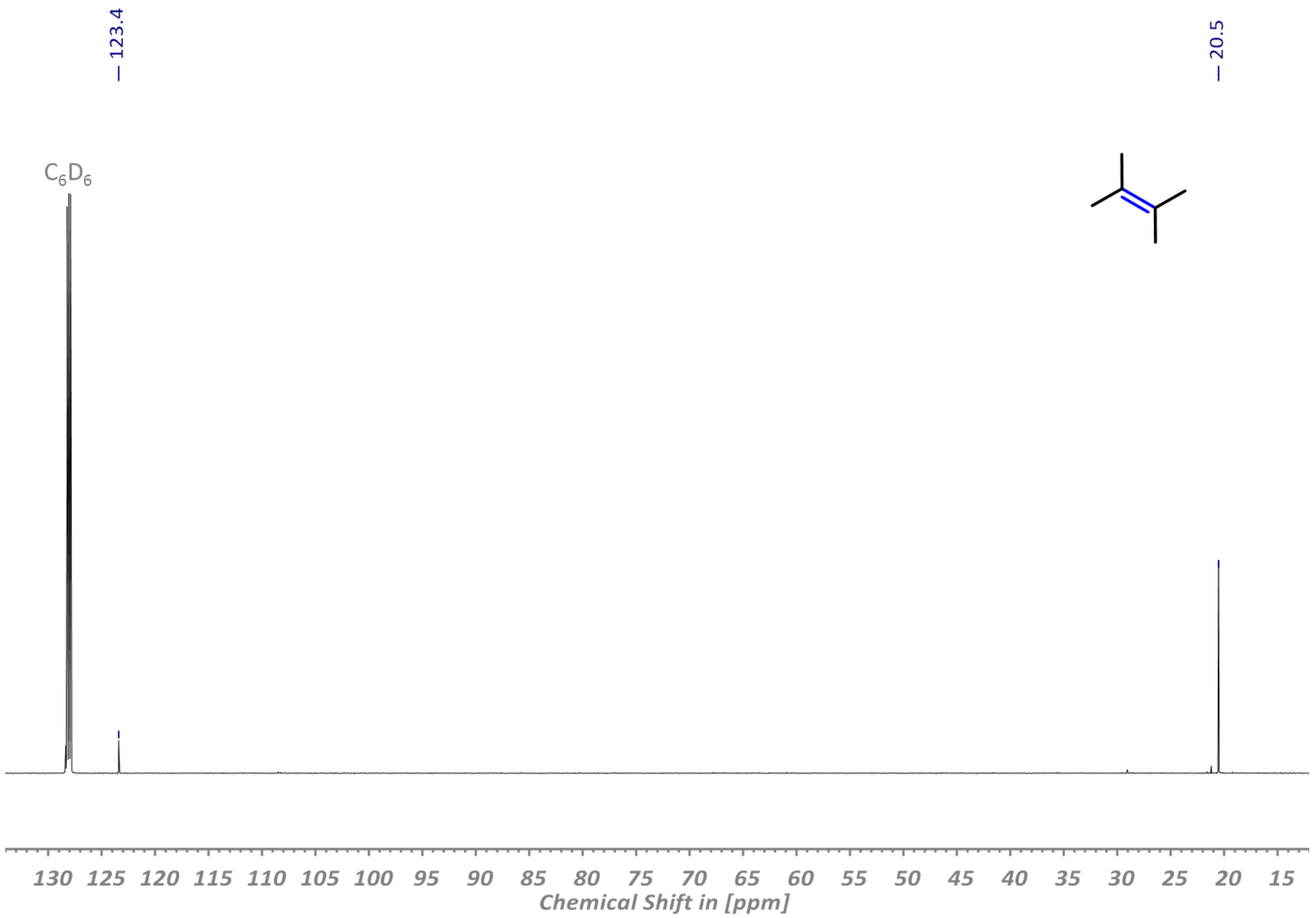

Figure S167. $^{13}C\{^{1}H\}$ NMR spectrum (151 MHz, C_6D_6, 25 °C) of 2,3-dimethyl-2-butene.

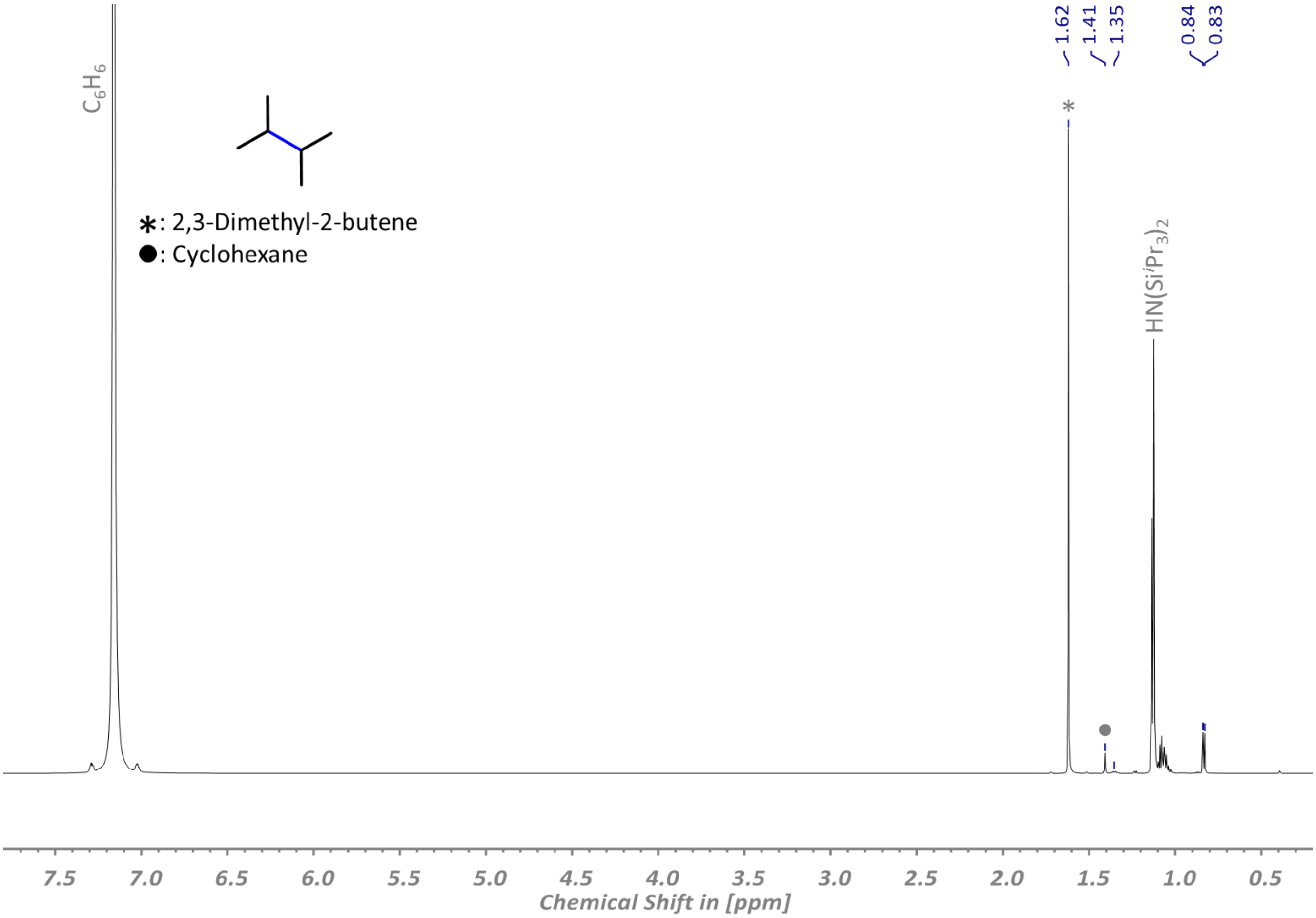

Figure S168. ^1H NMR spectrum (600 MHz, C_6D_6, 25 °C) after catalytic hydrogenation (24 h) of 2,3-dimethyl-2-butene (∗) with $Ba[N(Si^iPr_3)_2]_2$ (**1-Ba**) (20 mol%) and H_2 (20 bar) at 120 °C showing the formation of a substoichiometric amount of 2,3-dimethylbutane.[S21] *Note:* The product mixture contained small amounts of cyclohexane (●) likely from the competitive hydrogenation of the solvent C_6H_6 (Table S3, entry 58).

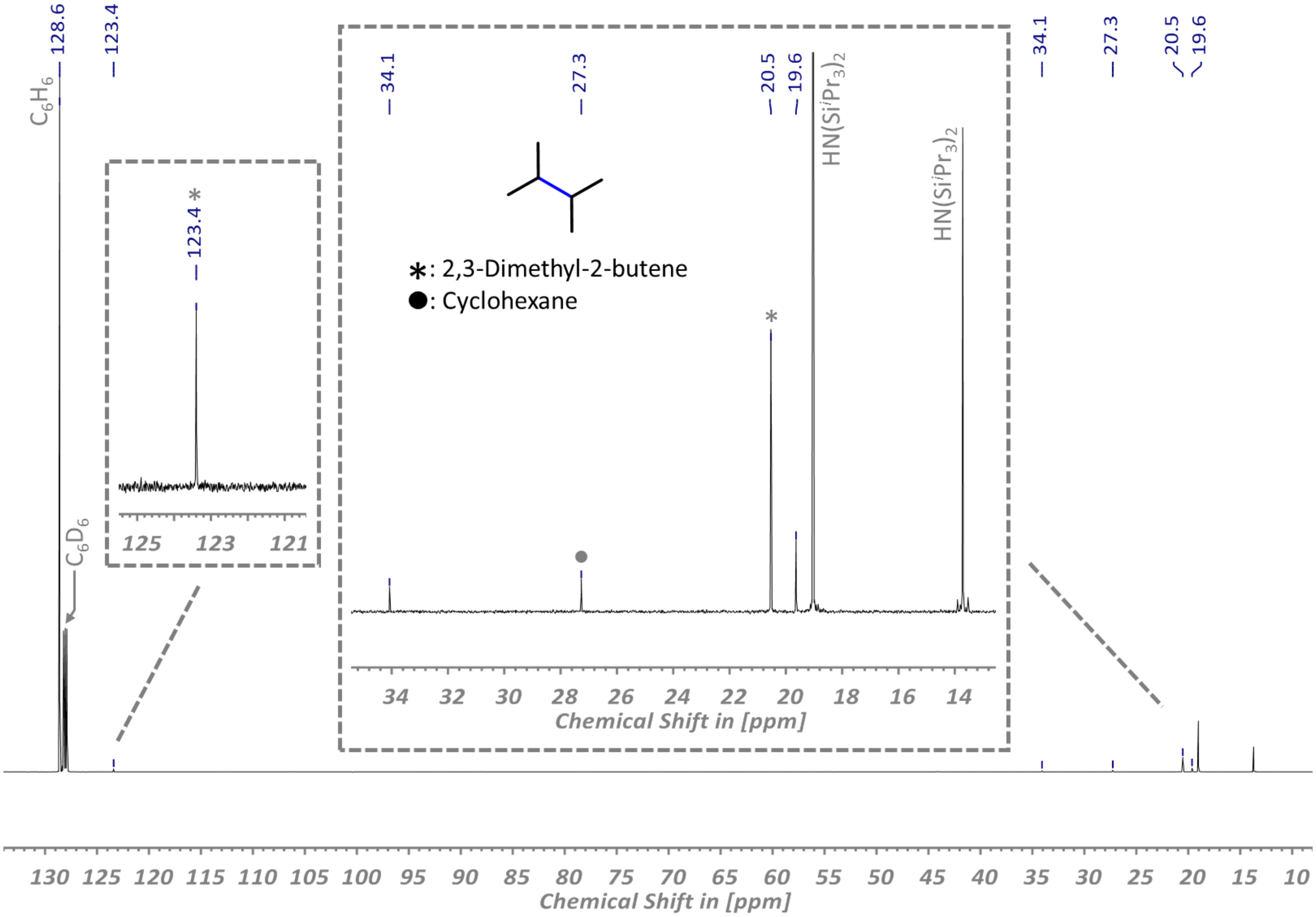

Figure S169. $^{13}C\{^1H\}$ NMR spectrum (151 MHz, C_6D_6, 25 °C) after catalytic hydrogenation (24 h) of 2,3-dimethyl-2-butene (∗) with $Ba[N(Si^iPr_3)_2]_2$ (**1-Ba**) (20 mol%) and H_2 (20 bar) at 120 °C showing the formation of a substoichiometric amount of 2,3-dimethylbutane.[S21] *Note:* The product mixture contained small amounts of cyclohexane (●) likely from the competitive hydrogenation of the solvent C_6H_6 (Table S3, entry 58).

6 Catalytic Hydrogenation of Arenes

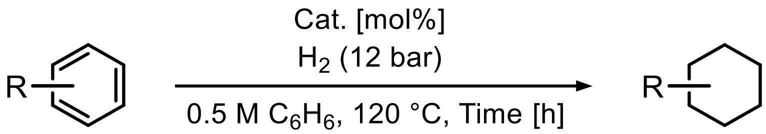

General Catalytic Procedure for Hydrogenation of Arenes:

In a typical hydrogenation experiment, the specified crystalline alkaline-earth metal pre-catalyst (0.05 mmol, 10 mol% or 12.5 µmol, 2.5 mol%), the corresponding arene substrate (0.5 mmol) and benzene (1 mL) were added to an oven-dried miniature stainless steel autoclave (15 mL) equipped with a magnetic stir bar under an atmosphere of N_2. The reactor was sealed tightly, brought out of the glovebox and attached to a H_2 gas manifold. After the supply line was evacuated and refilled several times with N_2, followed by three times with H_2, the autoclave was pressurized with H_2 (12 bar) and re-sealed. The reaction mixture was then stirred for the indicated time at 120 °C in a preheated aluminum heating block. At the end of the reaction, the pressure vessel was allowed to cool to room temperature and carefully vented in a fume hood. The reaction mixture was filtered through a glass microfiber filter in a Pasteur pipette and an aliquot of the crude filtrate was analyzed by GC/MS without further purification. The product composition was determined by the product/substrate peak ratios. The filtered product mixture was further analyzed using ^{1}H and $^{13}C\{^{1}H\}$ NMR spectroscopy following solvent evaporation under a dynamic vacuum and dissolving the residue in C_6D_6 (or $CDCl_3$). The identities of the hydrogenated products were confirmed by comparison of the obtained ^{1}H and $^{13}C\{^{1}H\}$ NMR data with corresponding data reported in the literature.

Table S4. Catalytic hydrogenation of arenes with alkaline-earth metal amide pre-catalysts.[a]

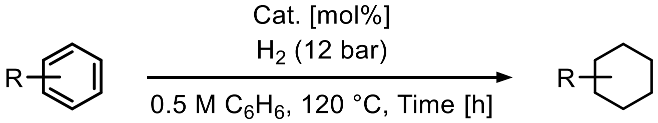

Entry	Catalyst	mol%	Substrate	t [h]	Product(s)	Conv.[b] [%]
1[c]	$Ca[N(SiMe_3)_2]_2$	10		24		44 / 1
2[c]	$Ca[N(Si^iPr_3)_2]_2$	10		24		83 / 2
3[c]	$Sr[N(SiMe_3)_2]_2$	10		24		97 / 2
4[c]	$Sr[N(DIPP)(Si^iPr_3)]_2$	10		24		96 / 2
5[c]	$Sr[N(Si^iPr_3)_2]_2$	10	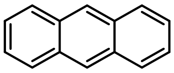	24	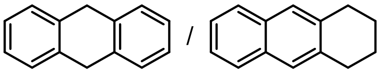	94 / 3
6[c]	$Sr[CH(SiMe_3)_2]_2(THF)_2$	10		24		66 / 1
7[c]	$(DMAT)_2Sr(THF)_3$	10		24		92 / 2
8[c]	$Ba[N(SiMe_3)_2]_2$	10		24		95 / 3
9[c]	$Ba[N(DIPP)(Si^iPr_3)]_2$	10		24		94 / 4
10[c]	$Ba[N(Si^iPr_3)_2]_2$	10		2.5		94 / 2
11	$Ca[N(Si^iPr_3)_2]_2$	10		24		7
12	$Sr[N(Si^iPr_3)_2]_2$	10		24		99
13	$Ba[N(SiMe_3)_2]_2$	10	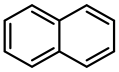	24	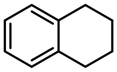	53
14	$Ba[N(DIPP)(Si^iPr_3)]_2$	10		24		98
15	$Ba[N(Si^iPr_3)_2]_2$	10		2		99
16	$Ba[N(Si^iPr_3)_2]_2$	2.5		24		35
17[c]	$Ba[N(Si^iPr_3)_2]_2$	10	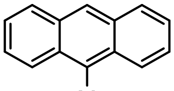 Me	24	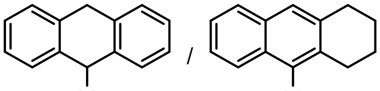 Me / Me	84 / 15
18	$Ba[N(Si^iPr_3)_2]_2$	10	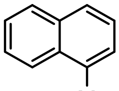 Me	24	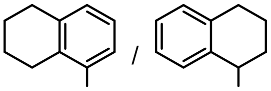 Me / Me	80 / 20
19	$Ba[N(Si^iPr_3)_2]_2$	10	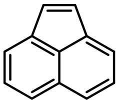	24	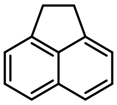	0
20	$Ba[N(Si^iPr_3)_2]_2$	10	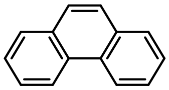	24	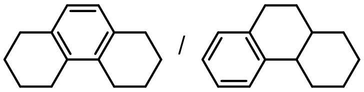	42 / 32<br>15 / 11
21[d]	$Ba[N(Si^iPr_3)_2]_2$	10		48	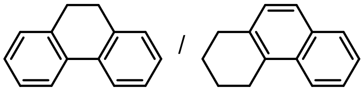	51 / 49<br>0 / 0

Table S4 (contd.). Catalytic hydrogenation of arenes with alkaline-earth metal amide pre-catalysts.[a]

Entry	Catalyst	mol%	Substrate	t [h]	Product(s)	Conv.[b] [%]
22[c]	Ba[N(Si^{i}Pr$_3$)$_2$]$_2$	10	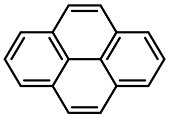	24	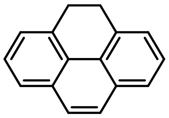	6
23	Ca[N(Si^{i}Pr$_3$)$_2$]$_2$	10		24		18
24	Sr[N(Si^{i}Pr$_3$)$_2$]$_2$	10	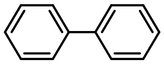	24	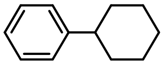	68
25	Ba[N(Si^{i}Pr$_3$)$_2$]$_2$	10		10		99
26[c]	Ba[N(Si^{i}Pr$_3$)$_2$]$_2$	10	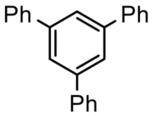	24	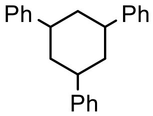	99

[a] General catalytic reaction conditions: arene (0.5 mmol), Ae metal catalyst (10 mol%), C_6H_6 (1 mL), H_2 (12 bar), 120 °C. [b] Conversion and product distribution were determined by GC/MS analysis; NMR spectroscopy was also used to corroborate the product identification. [c] Reaction conducted with $[arene]_0$ = 0.25 M in C_6H_6 (1 mL); [d] Reaction at 20 bar H_2.

NMR spectra

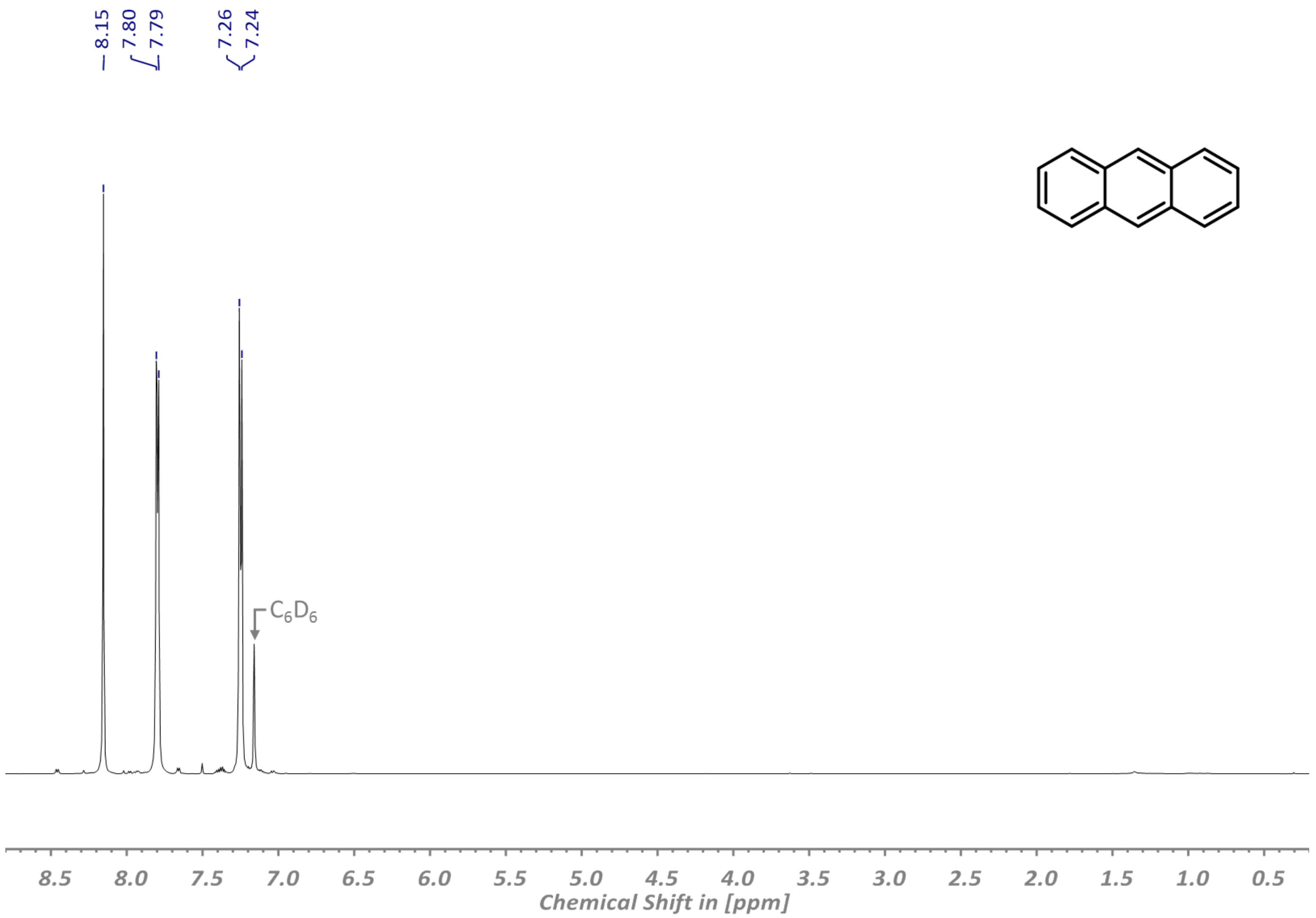

Figure S170. ^{1}H NMR spectrum (600 MHz, C_6D_6, 25 °C) of anthracene (98.5% purity as determined by GC/MS analysis).

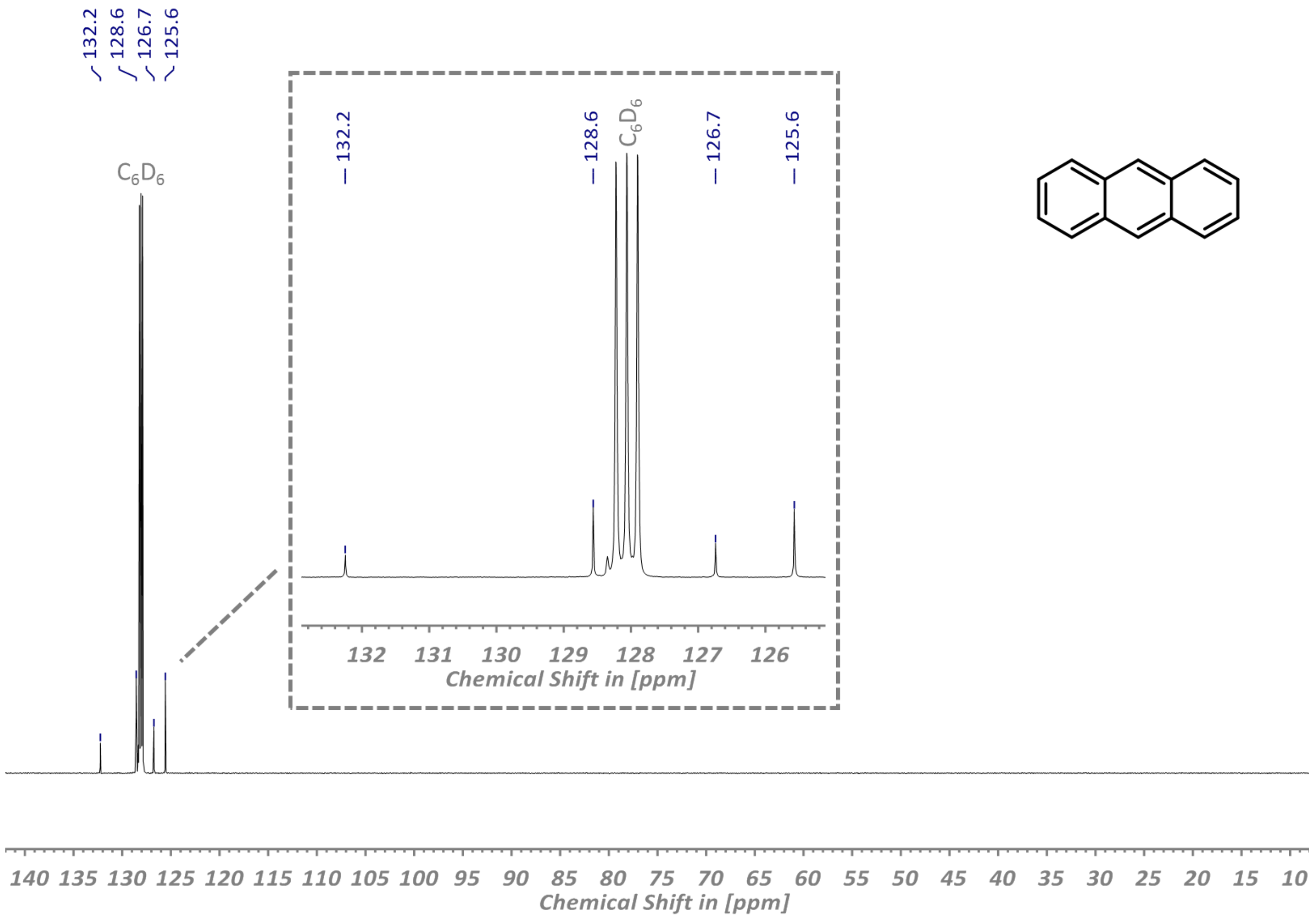

Figure S171. $^{13}C\{^{1}H\}$ NMR spectrum (151 MHz, C_6D_6, 25 °C) of anthracene.

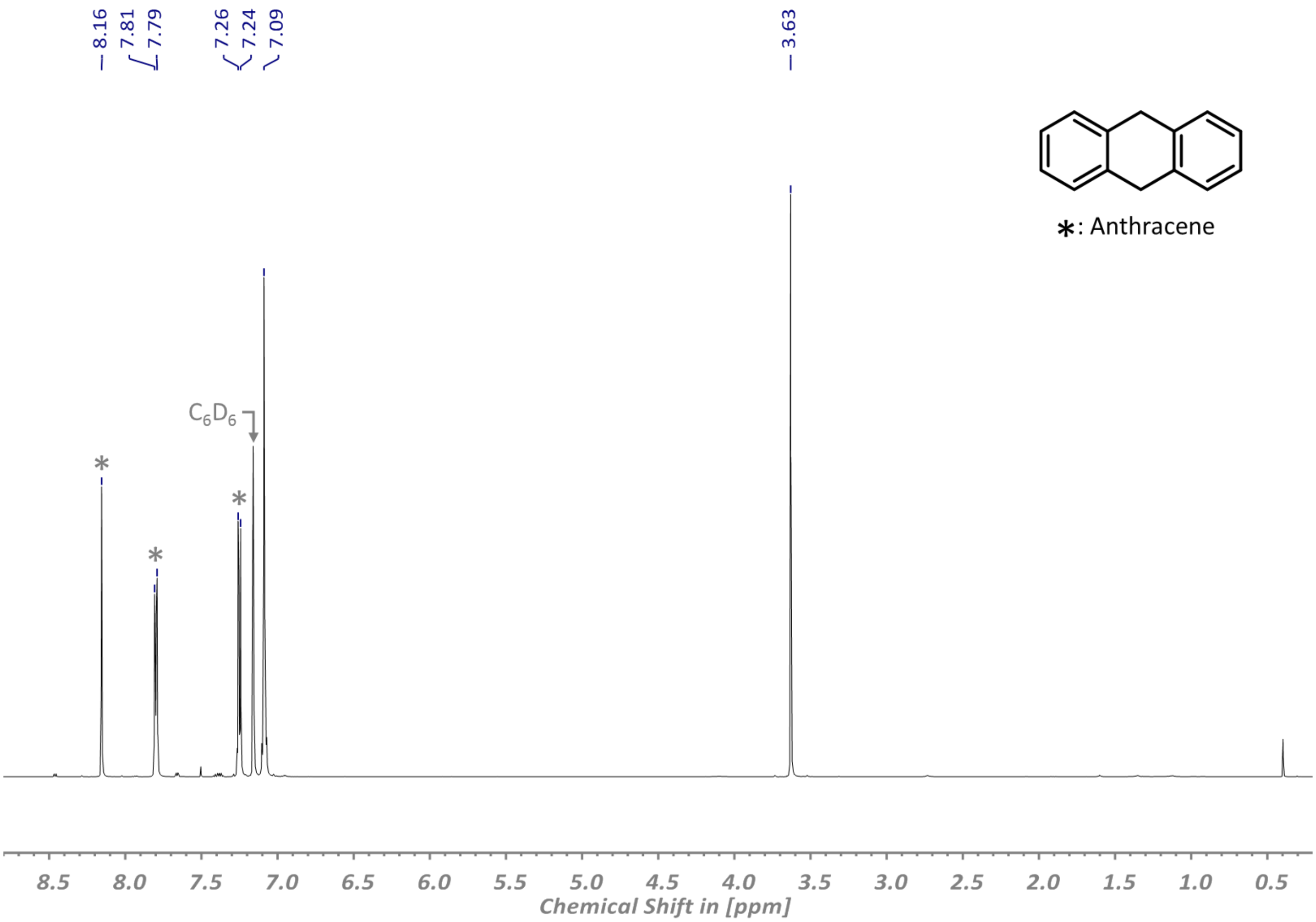

Figure S172. ^{1}H NMR spectrum (600 MHz, C_6D_6, 25 °C) after catalytic hydrogenation (24 h) of anthracene (∗) with $Ca[N(SiMe_3)_2]_2$ (10 mol%) and H_2 (12 bar) at 120 °C showing the formation of 9,10-dihydroanthracene (Table S4, entry 1).

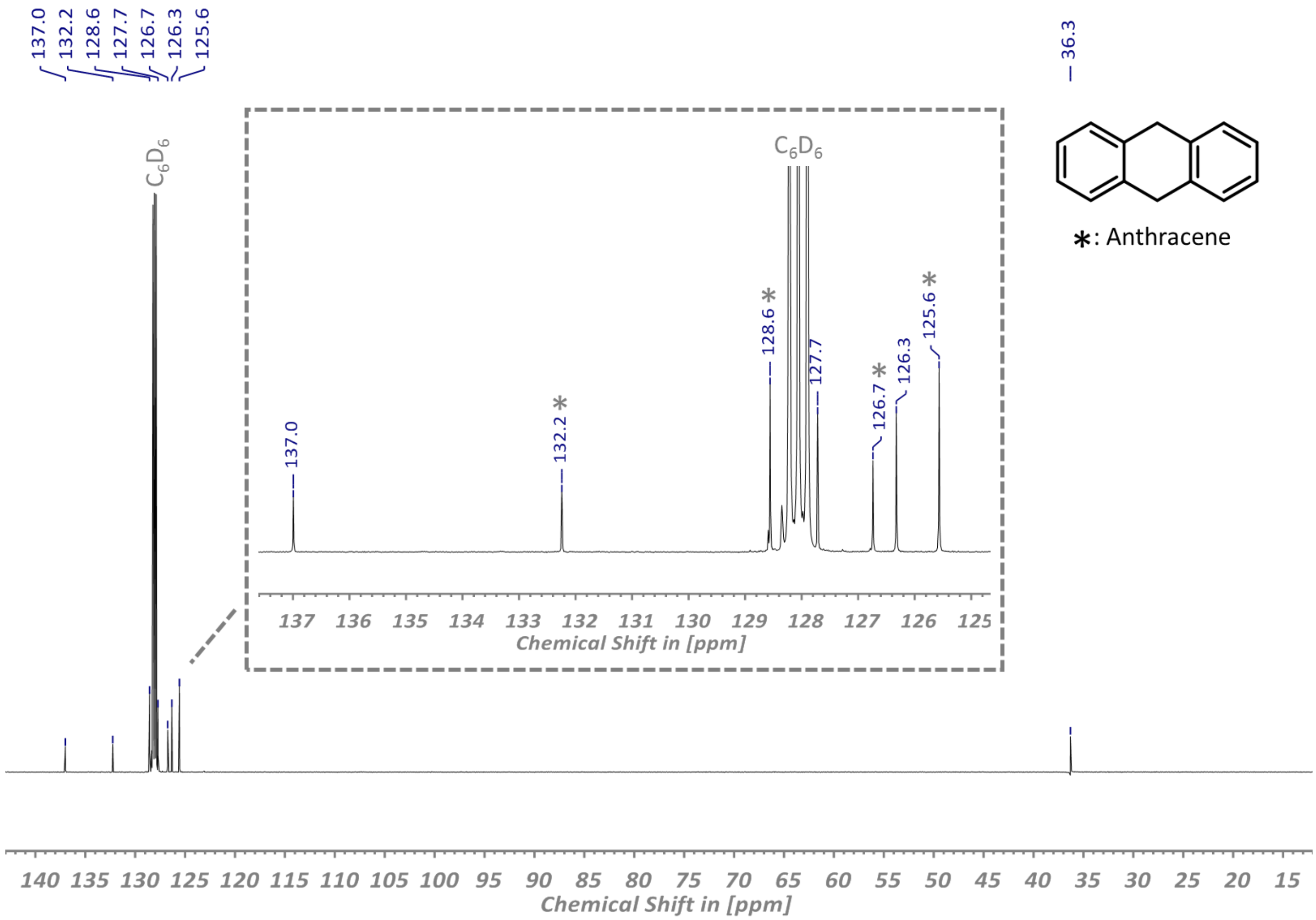

Figure S173. $^{13}C\{^{1}H\}$ NMR spectrum (151 MHz, C_6D_6, 25 °C) after catalytic hydrogenation (24 h) of anthracene (∗) with $Ca[N(SiMe_3)_2]_2$ (10 mol%) and H_2 (12 bar) at 120 °C showing the formation of 9,10-dihydroanthracene (Table S4, entry 1).

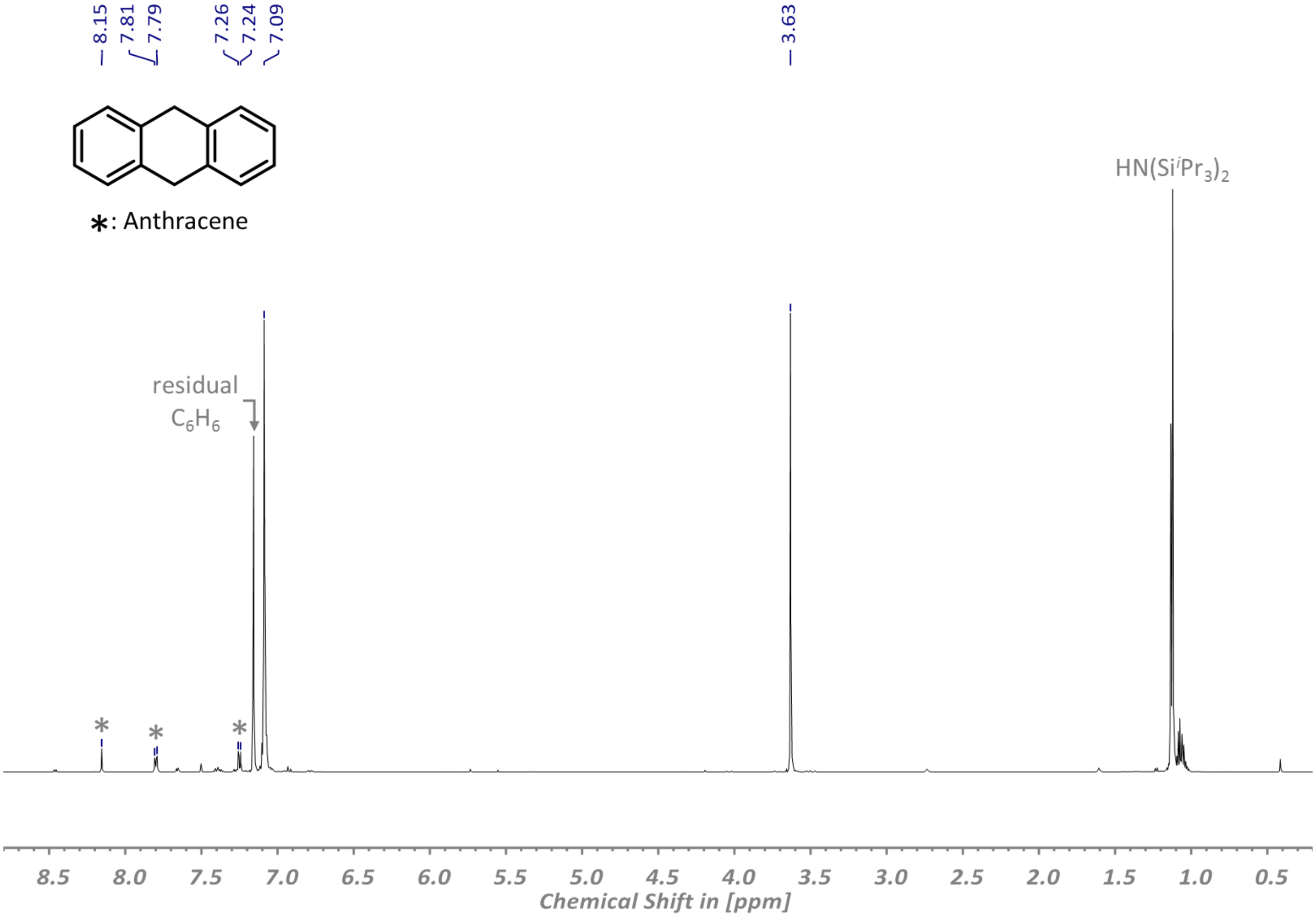

Figure S174. ^{1}H NMR spectrum (600 MHz, C_6D_6, 25 °C) after catalytic hydrogenation (24 h) of anthracene (∗) with Ca[N(Si^{i}Pr$_3$)$_2$]$_2$ (**1-Ca**) (10 mol%) and H_2 (12 bar) at 120 °C showing the formation of 9,10-dihydroanthracene (Table S4, entry 2).

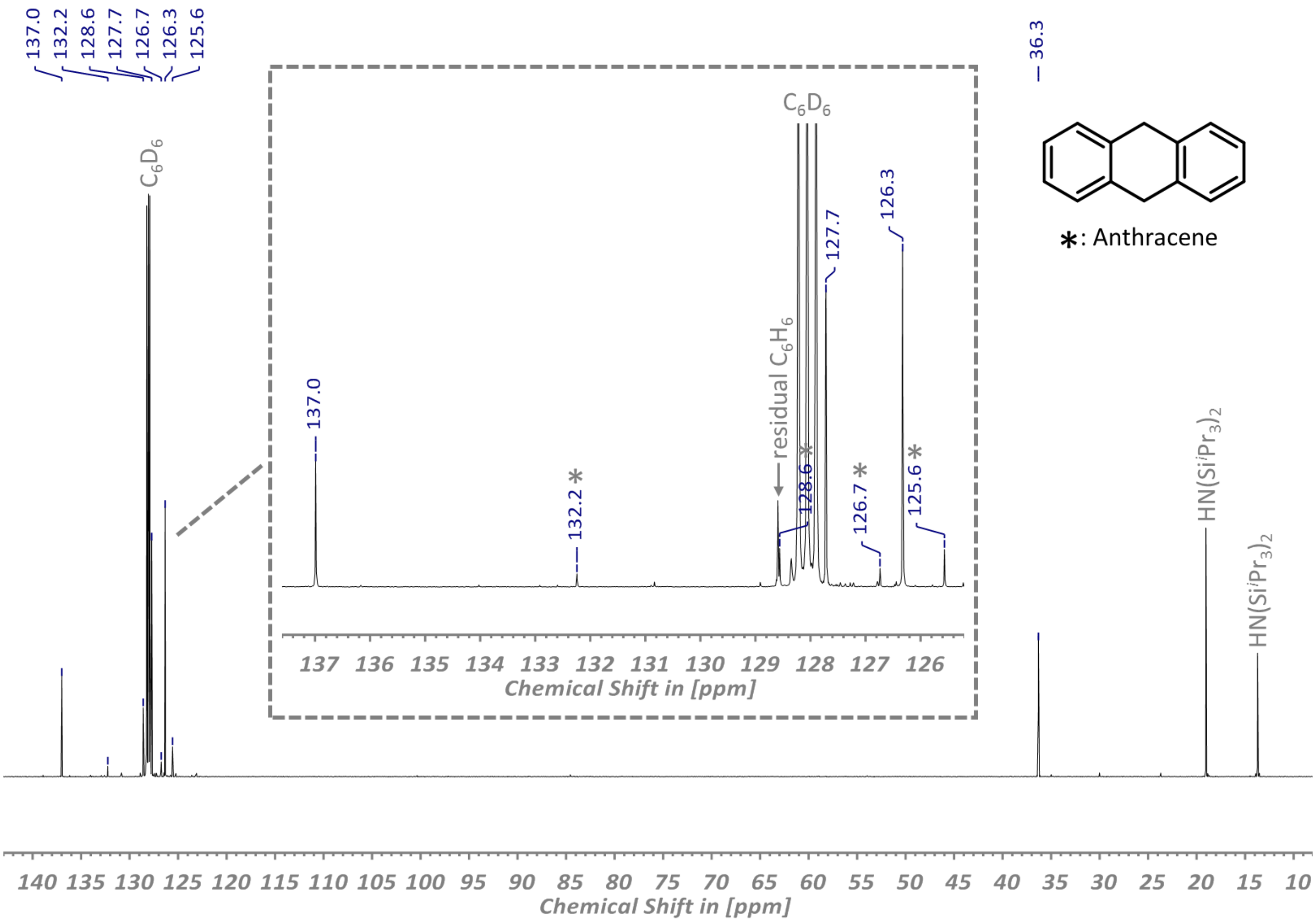

Figure S175. $^{13}C\{^1H\}$ NMR spectrum (151 MHz, C_6D_6, 25 °C) after catalytic hydrogenation (24 h) of anthracene (∗) with $Ca[N(Si^iPr_3)_2]_2$ (**1-Ca**) (10 mol%) and H_2 (12 bar) at 120 °C showing the formation of 9,10-dihydroanthracene (Table S4, entry 2).

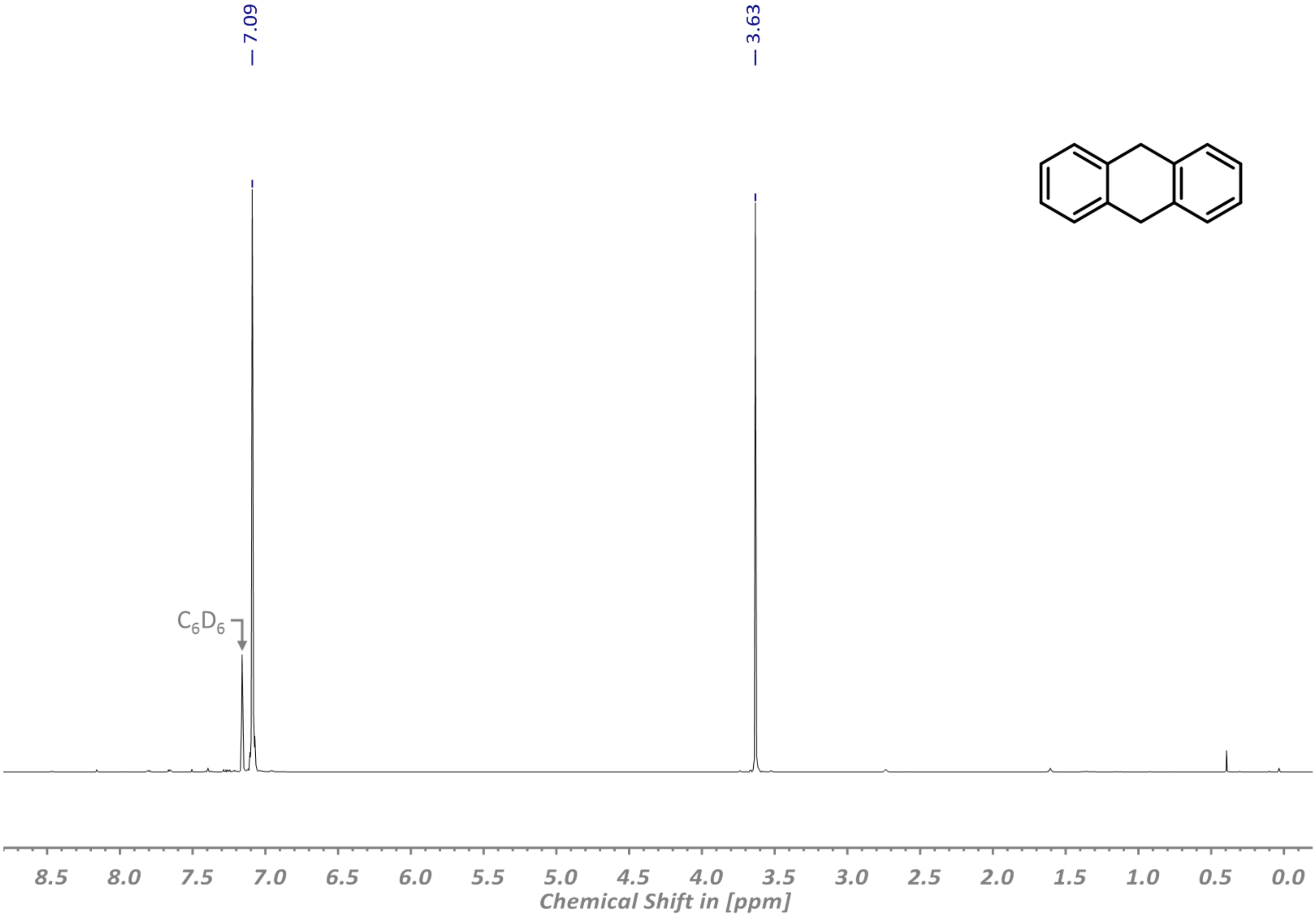

Figure S176. ^{1}H NMR spectrum (600 MHz, C_6D_6, 25 °C) of 9,10-dihydroanthracene after catalytic hydrogenation (24 h) of anthracene with $Sr[N(SiMe_3)_2]_2$ (10 mol%) and H_2 (12 bar) at 120 °C (Table S4, entry 3).

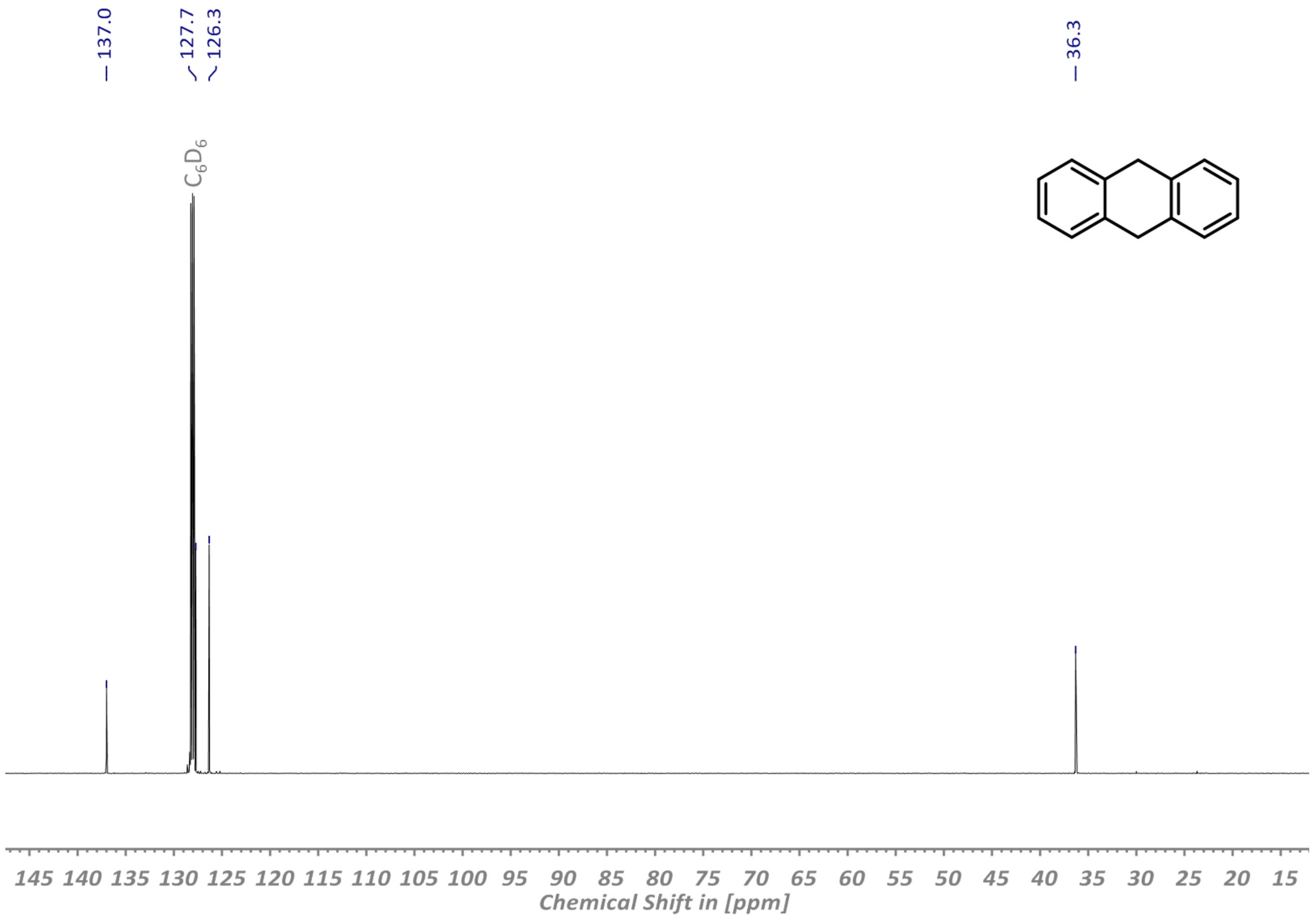

Figure S177. $^{13}C\{^{1}H\}$ NMR spectrum (600 MHz, C_6D_6, 25 °C) of 9,10-dihydroanthracene after catalytic hydrogenation (24 h) of anthracene with $Sr[N(SiMe_3)_2]_2$ (10 mol%) and H_2 (12 bar) at 120 °C (Table S4, entry 3).

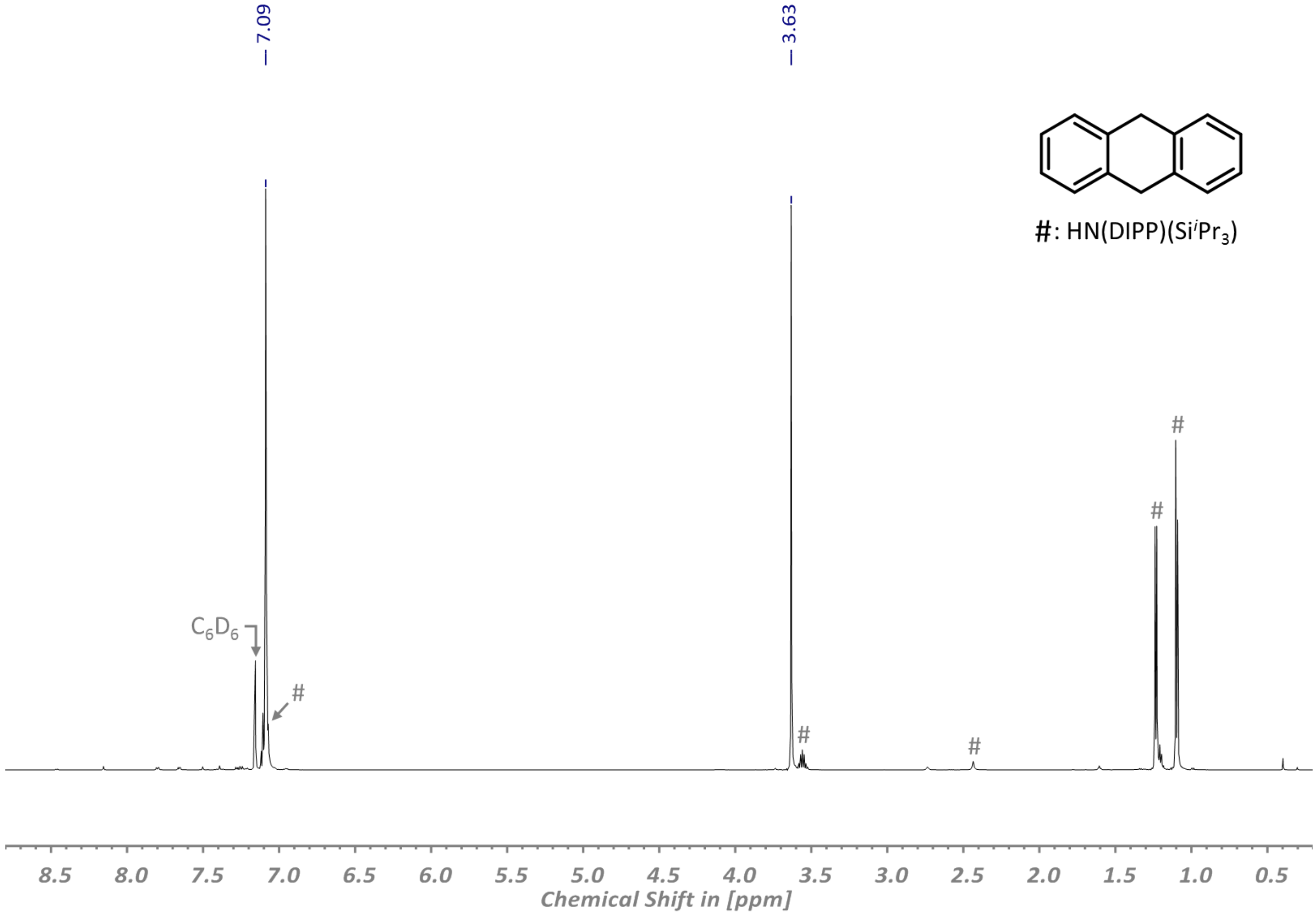

Figure S178. ^{1}H NMR spectrum (600 MHz, C_6D_6, 25 °C) of 9,10-dihydroanthracene after catalytic hydrogenation (24 h) of anthracene with $Sr[N(DIPP)(Si^{i}Pr_3)]_2$ (**2-Sr**) (10 mol%) and H_2 (12 bar) at 120 °C. *Note:* # denotes $HN(DIPP)(Si^{i}Pr_3)$ (Table S4, entry 4).

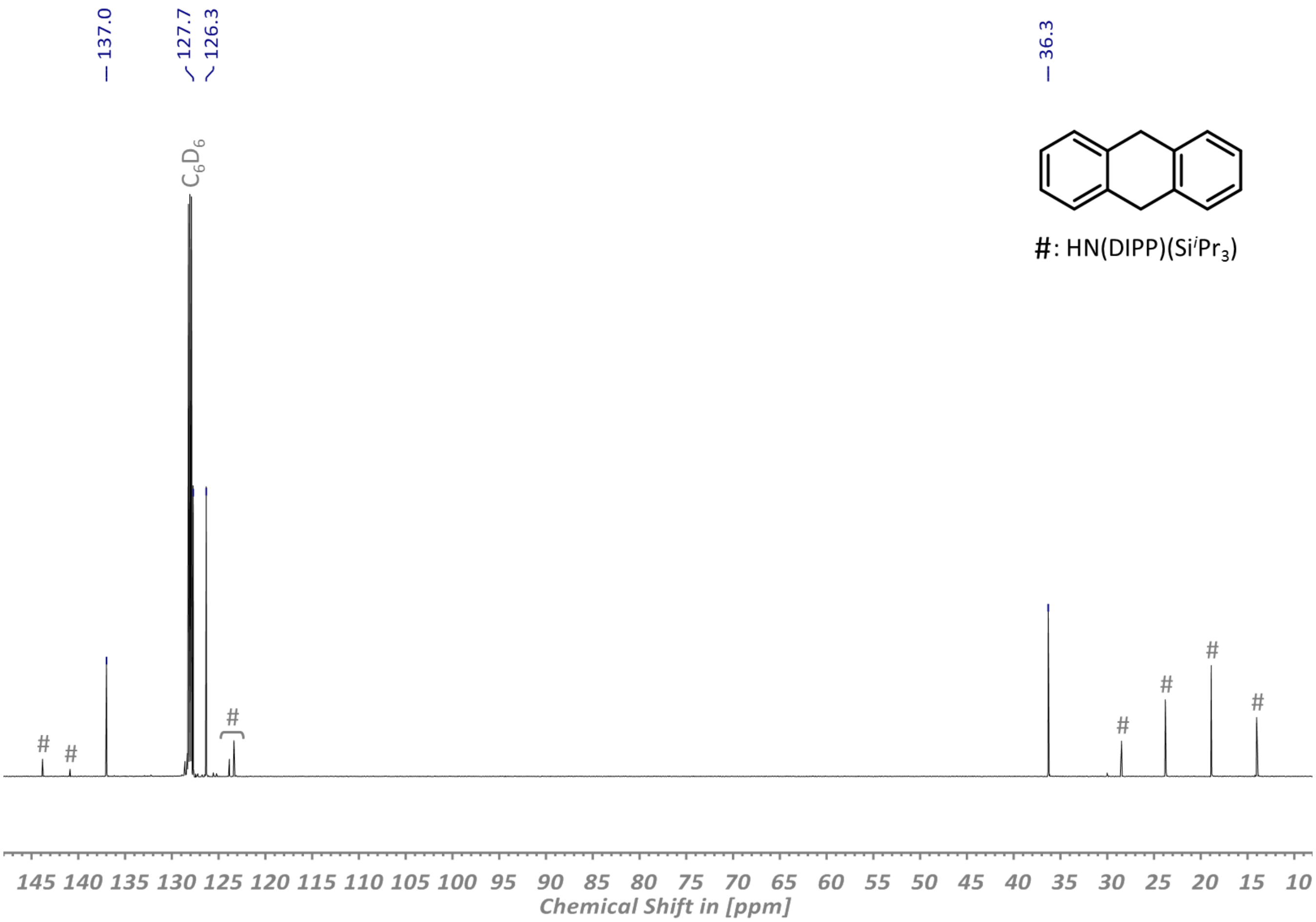

Figure S179. $^{13}C\{^1H\}$ NMR spectrum (151 MHz, C_6D_6, 25 °C) of 9,10-dihydroanthracene after catalytic hydrogenation (24 h) of anthracene with $Sr[N(DIPP)(Si^iPr_3)]_2$ (**2-Sr**) (10 mol%) and H_2 (12 bar) at 120 °C. *Note:* # denotes $HN(DIPP)(Si^iPr_3)$ (Table S4, entry 4).

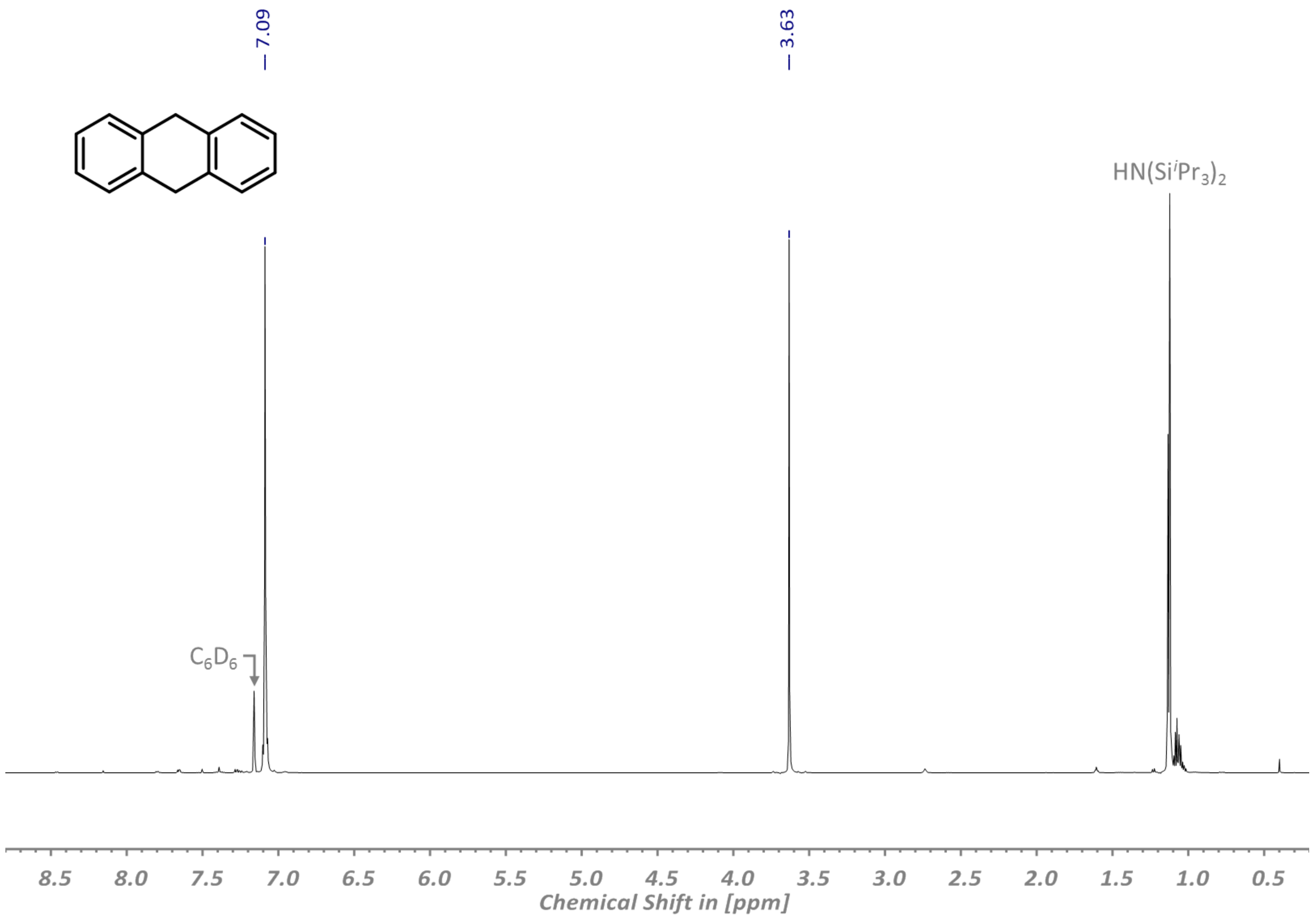

Figure S180. ^{1}H NMR spectrum (600 MHz, C_6D_6, 25 °C) of 9,10-dihydroanthracene after catalytic hydrogenation (24 h) of anthracene with $Sr[N(Si^iPr_3)_2]_2$ (**1-Sr**) (10 mol%) and H_2 (12 bar) at 120 °C (Table S4, entry 5).

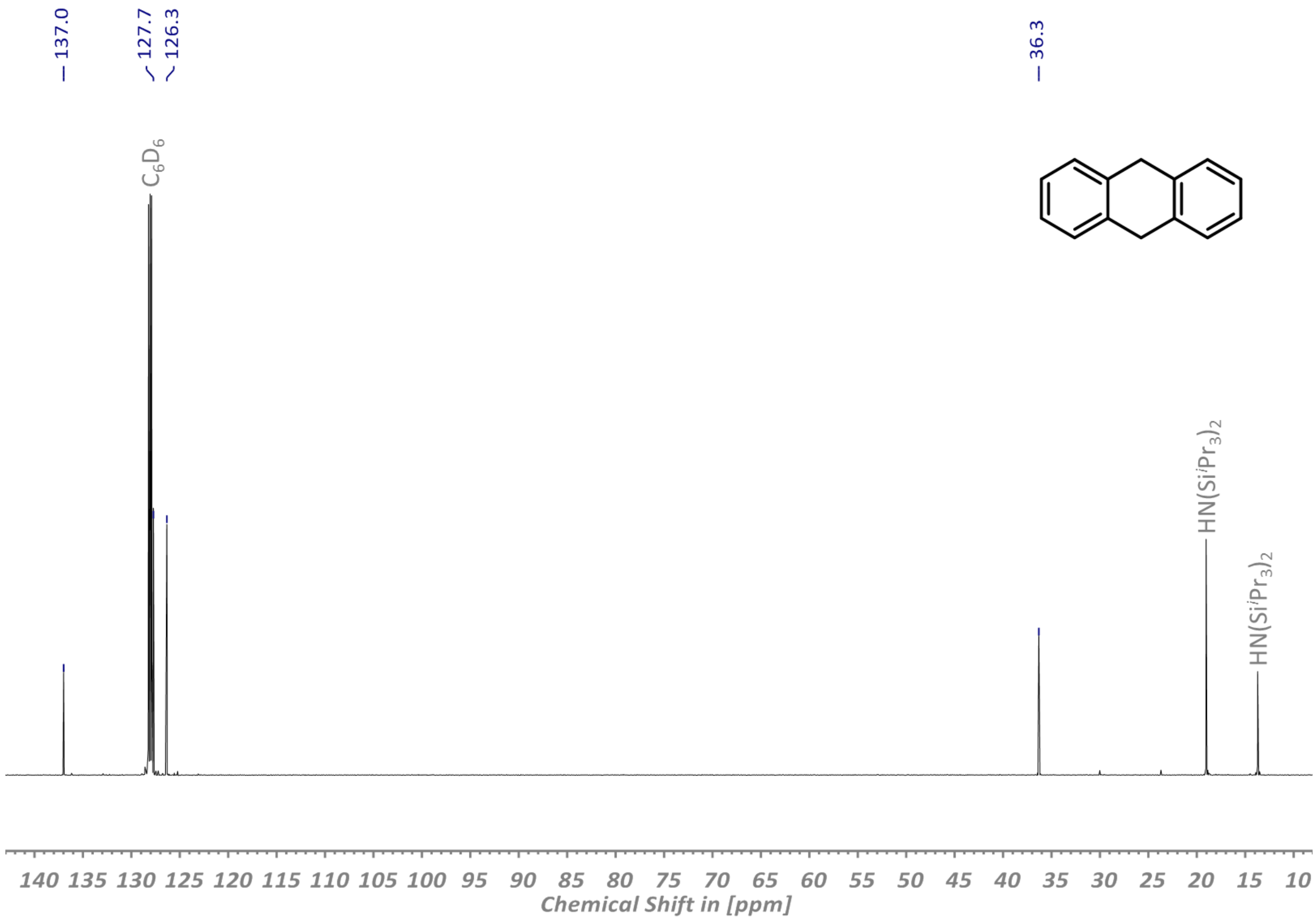

Figure S181. $^{13}C\{^{1}H\}$ NMR spectrum (151 MHz, C_6D_6, 25 °C) of 9,10-dihydroanthracene after catalytic hydrogenation (24 h) of anthracene with $Sr[N(Si^{i}Pr_3)_2]_2$ (**1-Sr**) (10 mol%) and H_2 (12 bar) at 120 °C (Table S4, entry 5).

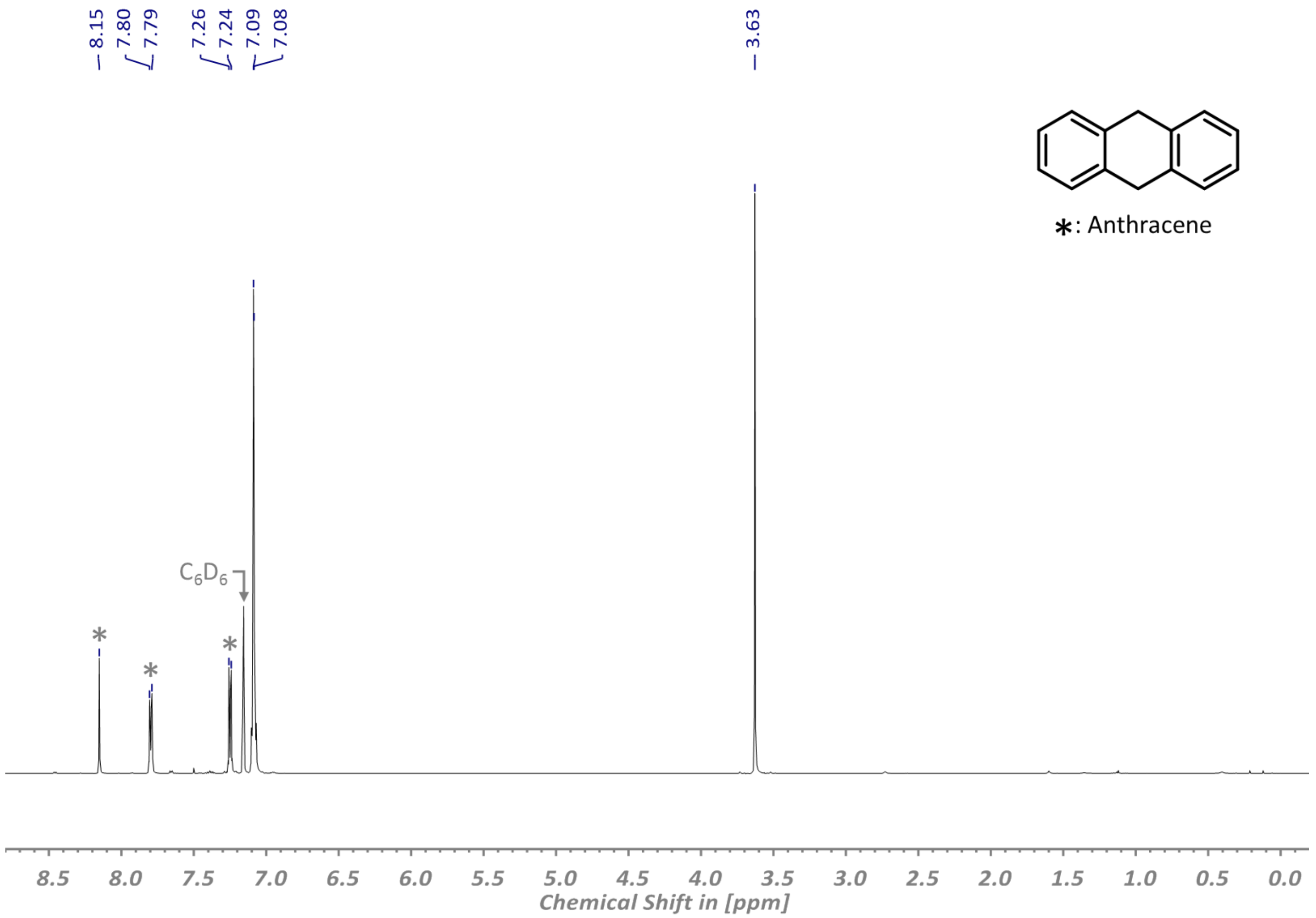

Figure S182. ^1H NMR spectrum (600 MHz, C_6D_6, 25 °C) after catalytic hydrogenation (24 h) of anthracene (∗) with $Sr[CH(SiMe_3)_2]_2(THF)_2$ (10 mol%) and H_2 (12 bar) at 120 °C showing the formation of 9,10-dihydroanthracene (Table S4, entry 6).

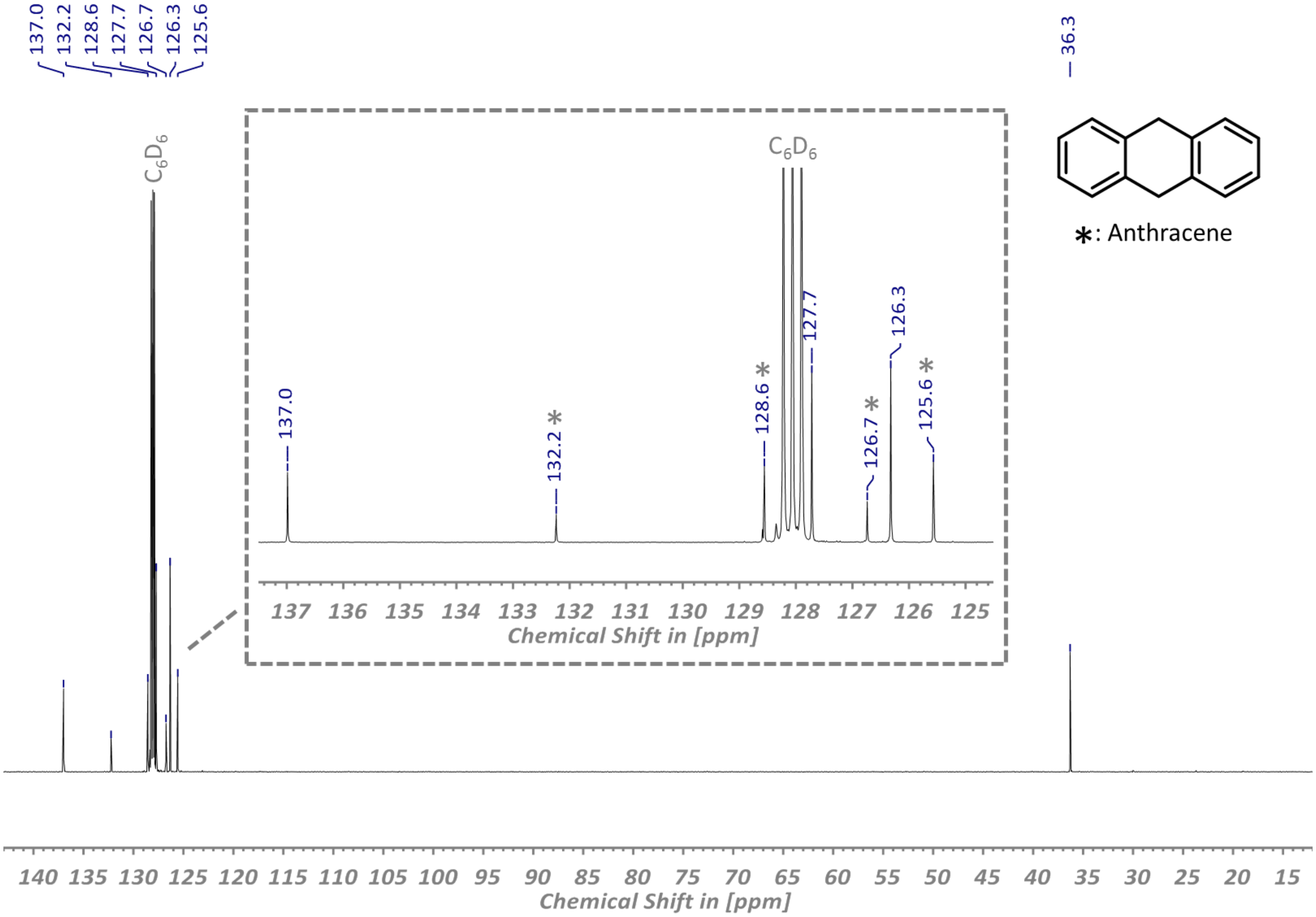

Figure S183. $^{13}C\{^1H\}$ NMR spectrum (151 MHz, C_6D_6, 25 °C) after catalytic hydrogenation (24 h) of anthracene (∗) with $Sr[CH(SiMe_3)_2]_2(THF)_2$ (10 mol%) and H_2 (12 bar) at 120 °C showing the formation of 9,10-dihydroanthracene (Table S4, entry 6).

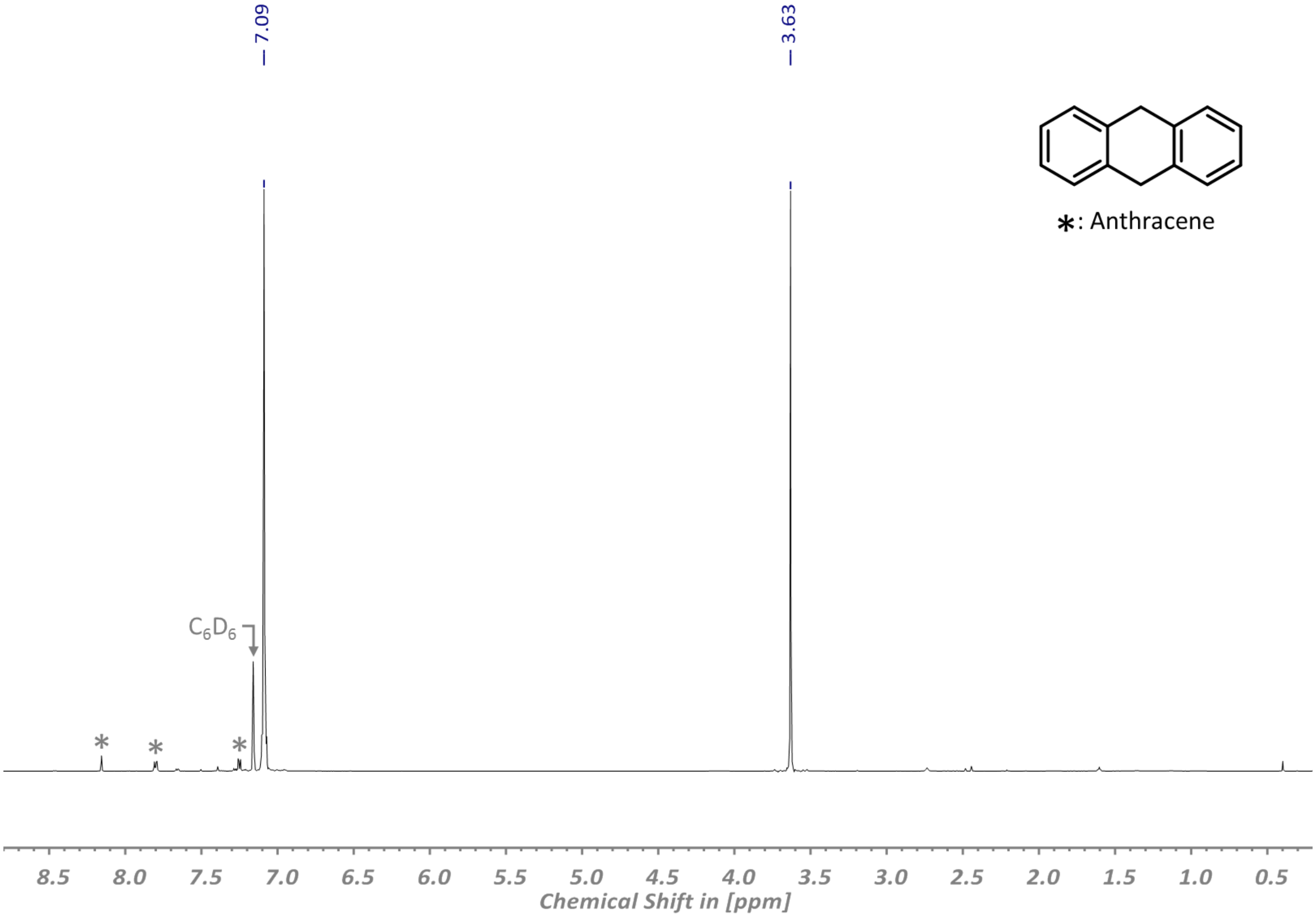

Figure S184. ^{1}H NMR spectrum (600 MHz, C_6D_6, 25 °C) after catalytic hydrogenation (24 h) of anthracene (∗) with $(DMAT)_2Sr(THF)_3$ (10 mol%) and H_2 (12 bar) at 120 °C showing the formation of 9,10-dihydroanthracene (Table S4, entry 7).

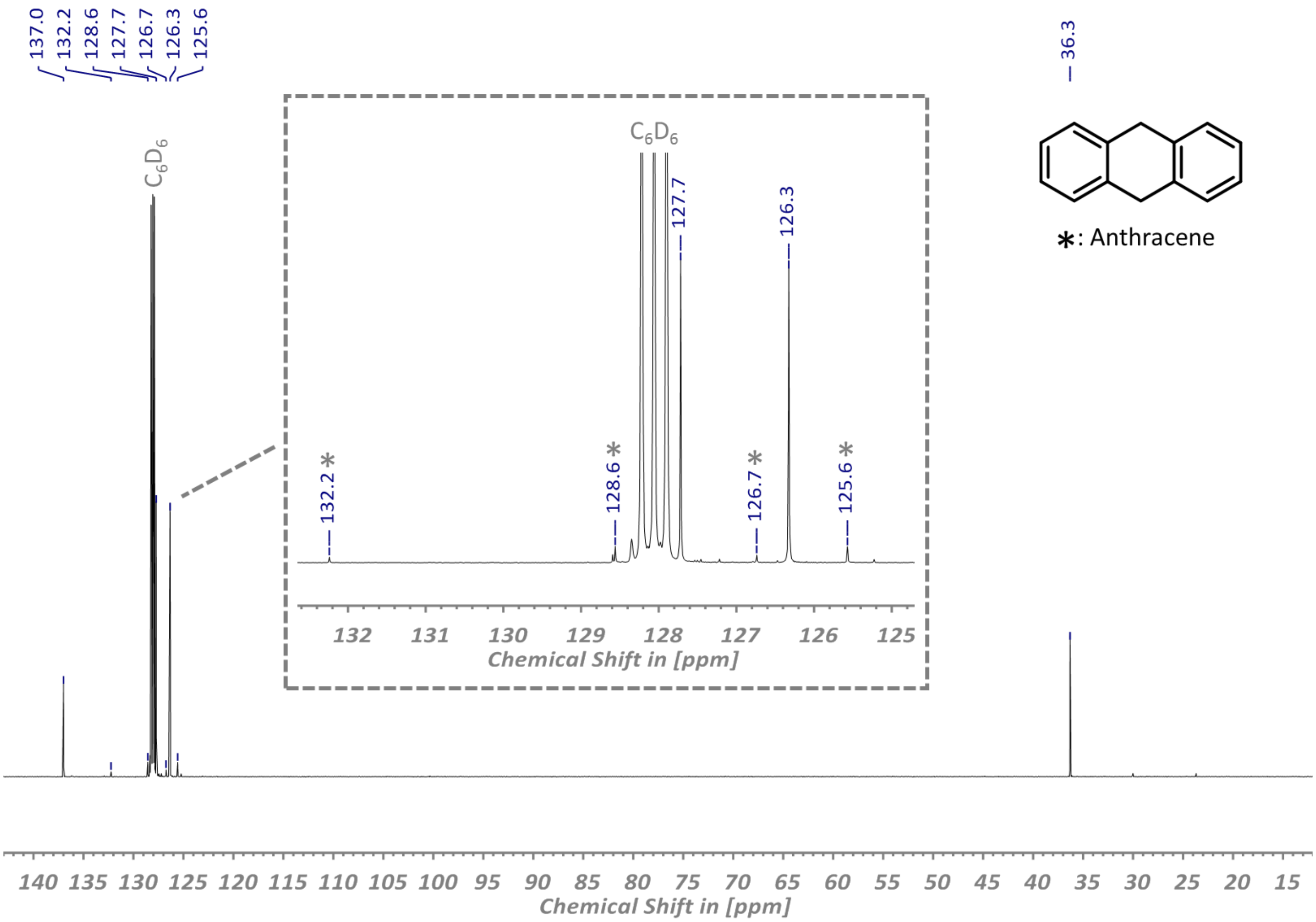

Figure S185. $^{13}C\{^{1}H\}$ NMR spectrum (151 MHz, C_6D_6, 25 °C) after catalytic hydrogenation (24 h) of anthracene (∗) with $(DMAT)_2Sr(THF)_3$ (10 mol%) and H_2 (12 bar) at 120 °C showing the formation of 9,10-dihydroanthracene (Table S4, entry 7).

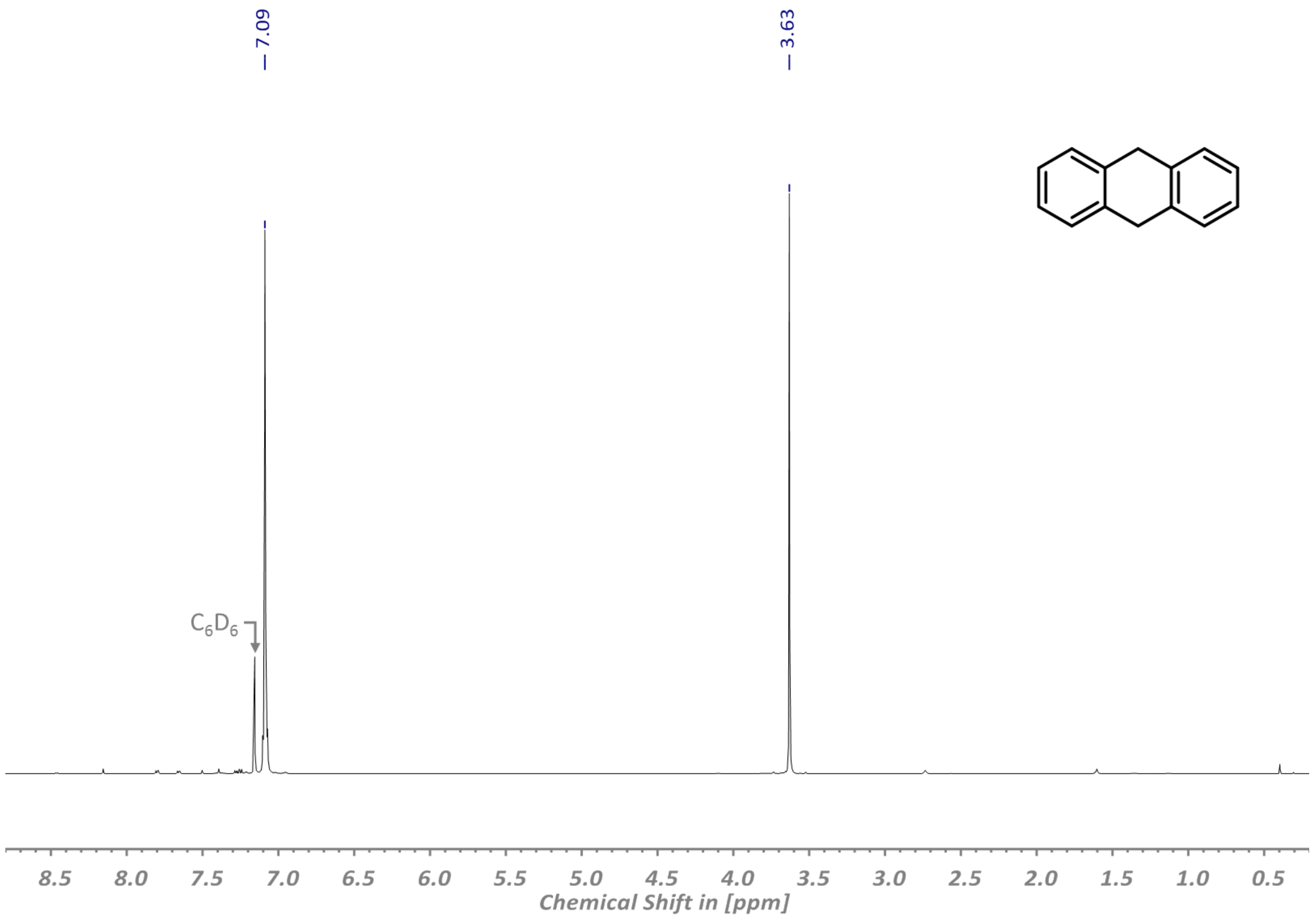

Figure S186. ^{1}H NMR spectrum (600 MHz, C_6D_6, 25 °C) of 9,10-dihydroanthracene after catalytic hydrogenation (24 h) of anthracene with $Ba[N(SiMe_3)_2]_2$ (10 mol%) and H_2 (12 bar) at 120 °C (Table S4, entry 8).

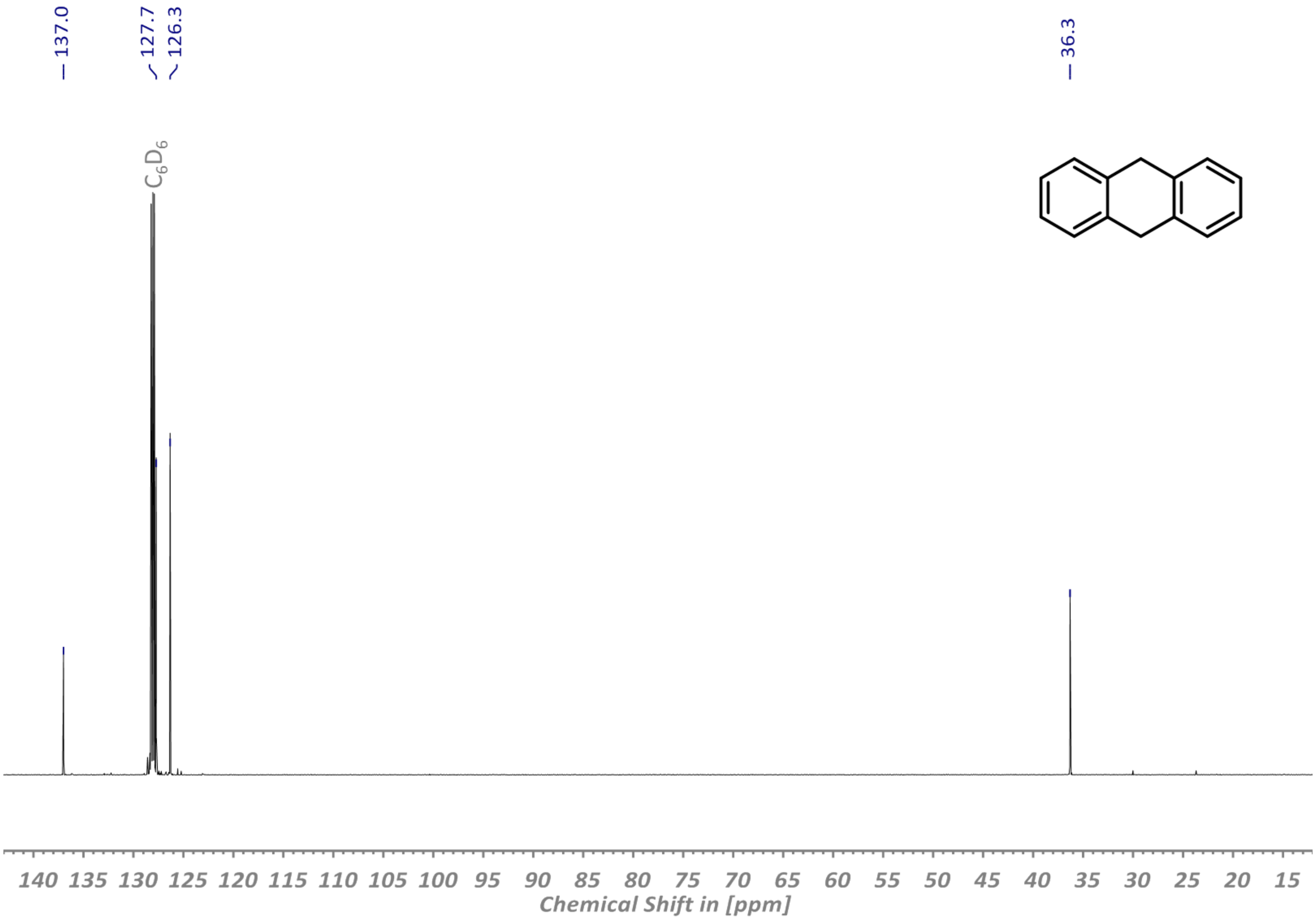

Figure S187. $^{13}C\{^{1}H\}$ NMR spectrum (151 MHz, C_6D_6, 25 °C) of 9,10-dihydroanthracene after catalytic hydrogenation (24 h) of anthracene with $Ba[N(SiMe_3)_2]_2$ (10 mol%) and H_2 (12 bar) at 120 °C (Table S4, entry 8).

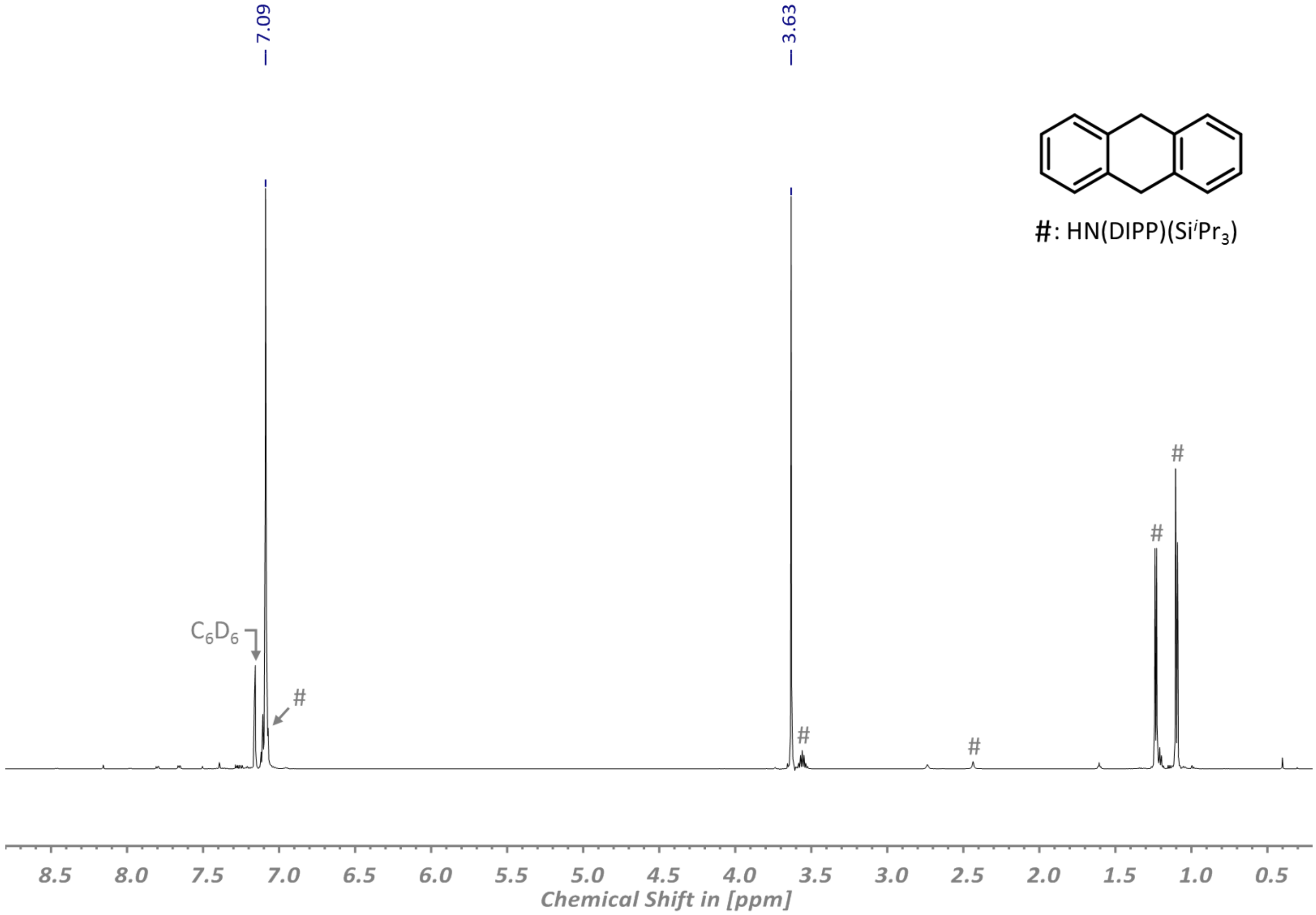

Figure S188. ^{1}H NMR spectrum (600 MHz, C_6D_6, 25 °C) of 9,10-dihydroanthracene after catalytic hydrogenation (24 h) of anthracene with Ba[N(DIPP)(Si^{i}Pr$_3$)]$_2$ (**2-Ba**) (10 mol%) and H_2 (12 bar) at 120 °C. *Note:* # denotes HN(DIPP)(Si^{i}Pr$_3$) (Table S4, entry 9).

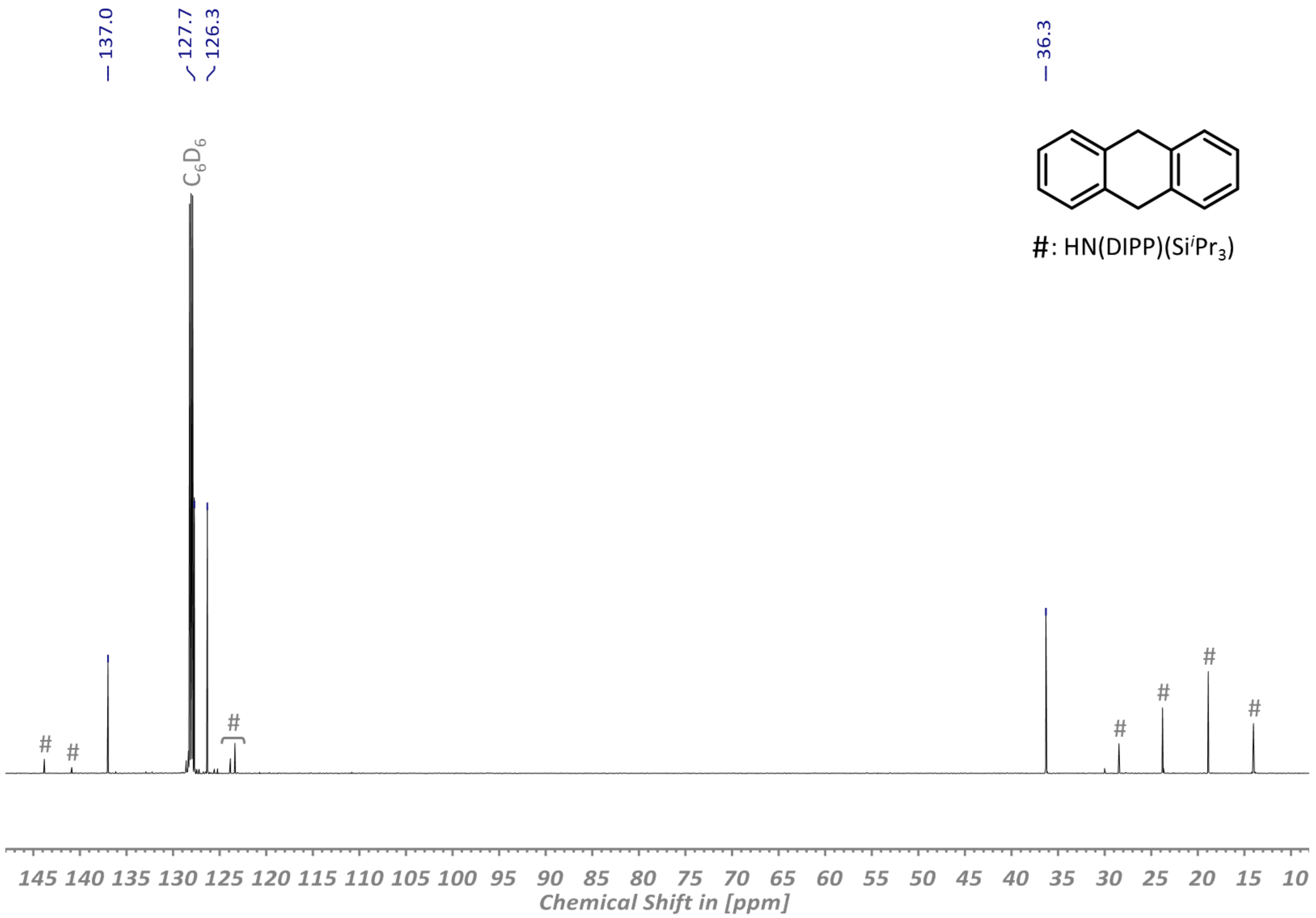

Figure S189. $^{13}C\{^{1}H\}$ NMR spectrum (151 MHz, C_6D_6, 25 °C) of 9,10-dihydroanthracene after catalytic hydrogenation (24 h) of anthracene with Ba[N(DIPP)(Si^{i}Pr$_3$)]$_2$ (**2-Ba**) (10 mol%) and H_2 (12 bar) at 120 °C. *Note:* # denotes HN(DIPP)(Si^{i}Pr$_3$) (Table S4, entry 9).

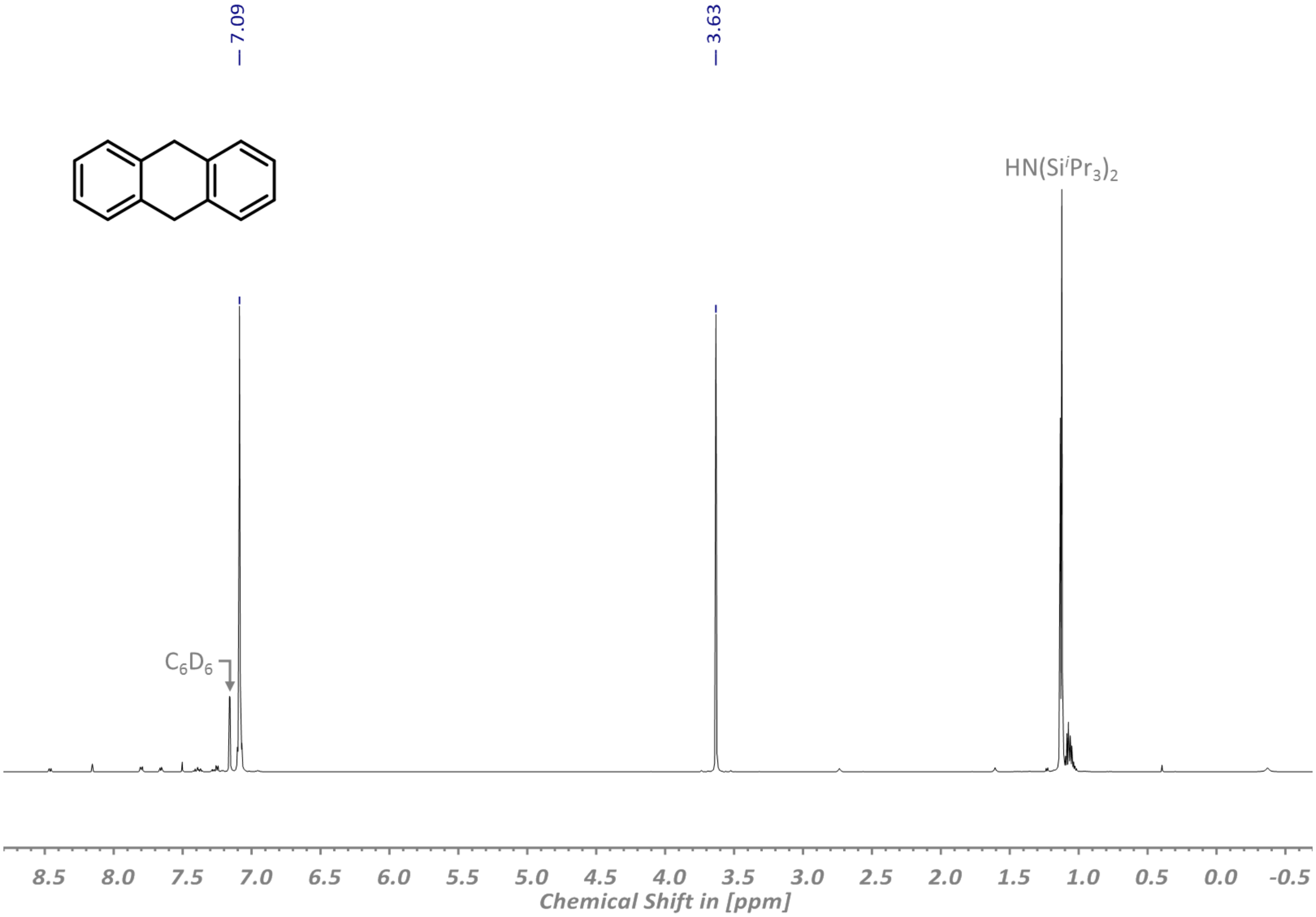

Figure S190. ^{1}H NMR spectrum (600 MHz, C_6D_6, 25 °C) of 9,10-dihydroanthracene after catalytic hydrogenation (2.5 h) of anthracene with Ba[N(Si^{i}Pr$_3$)$_2$]$_2$ (**1-Ba**) (10 mol%) and H_2 (12 bar) at 120 °C (Table S4, entry 10).

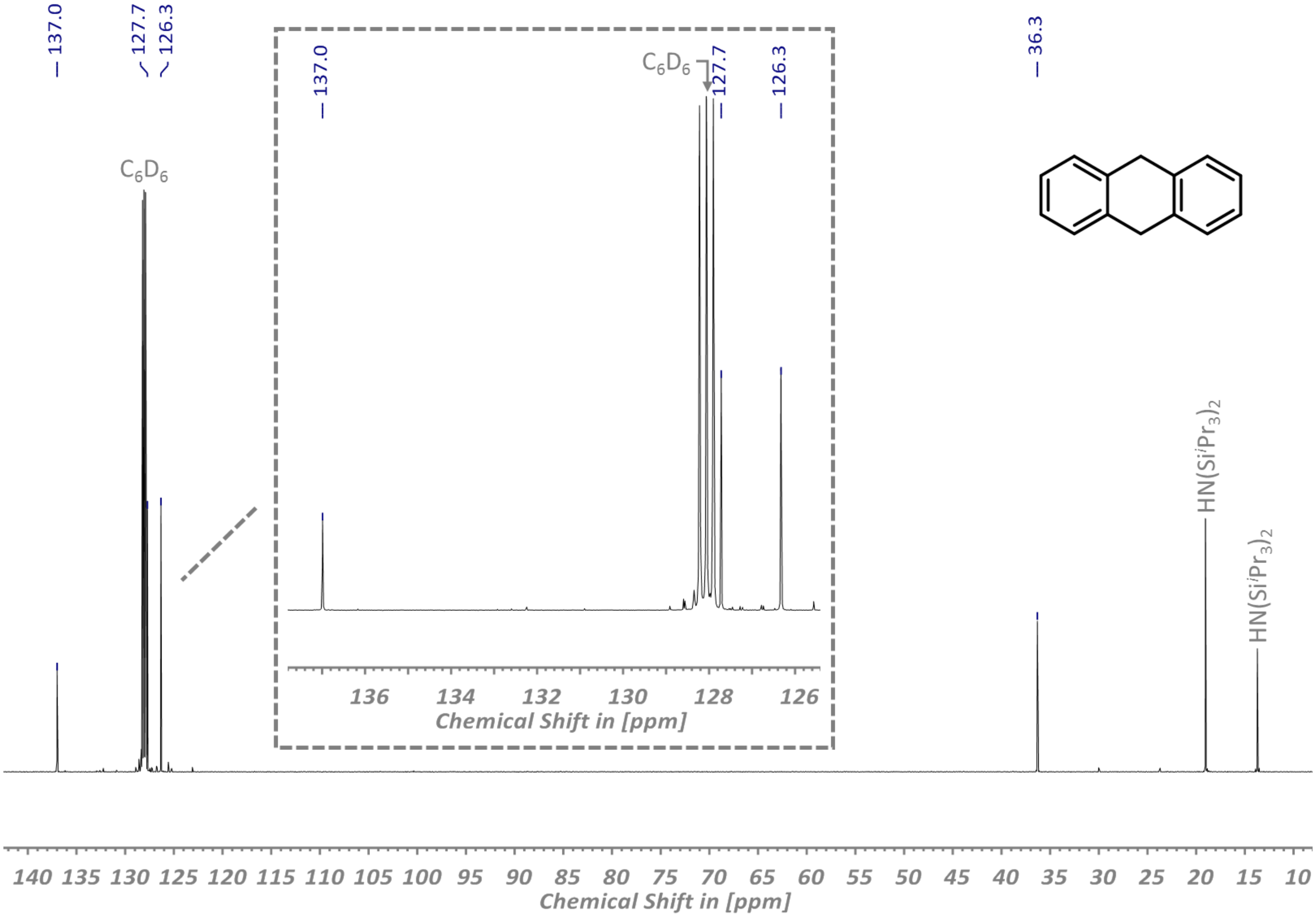

Figure S191. $^{13}C\{^1H\}$ NMR spectrum (151 MHz, C_6D_6, 25 °C) of 9,10-dihydroanthracene after catalytic hydrogenation (2.5 h) of anthracene with $Ba[N(Si^iPr_3)_2]_2$ (**1-Ba**) (10 mol%) and H_2 (12 bar) at 120 °C (Table S4, entry 10).

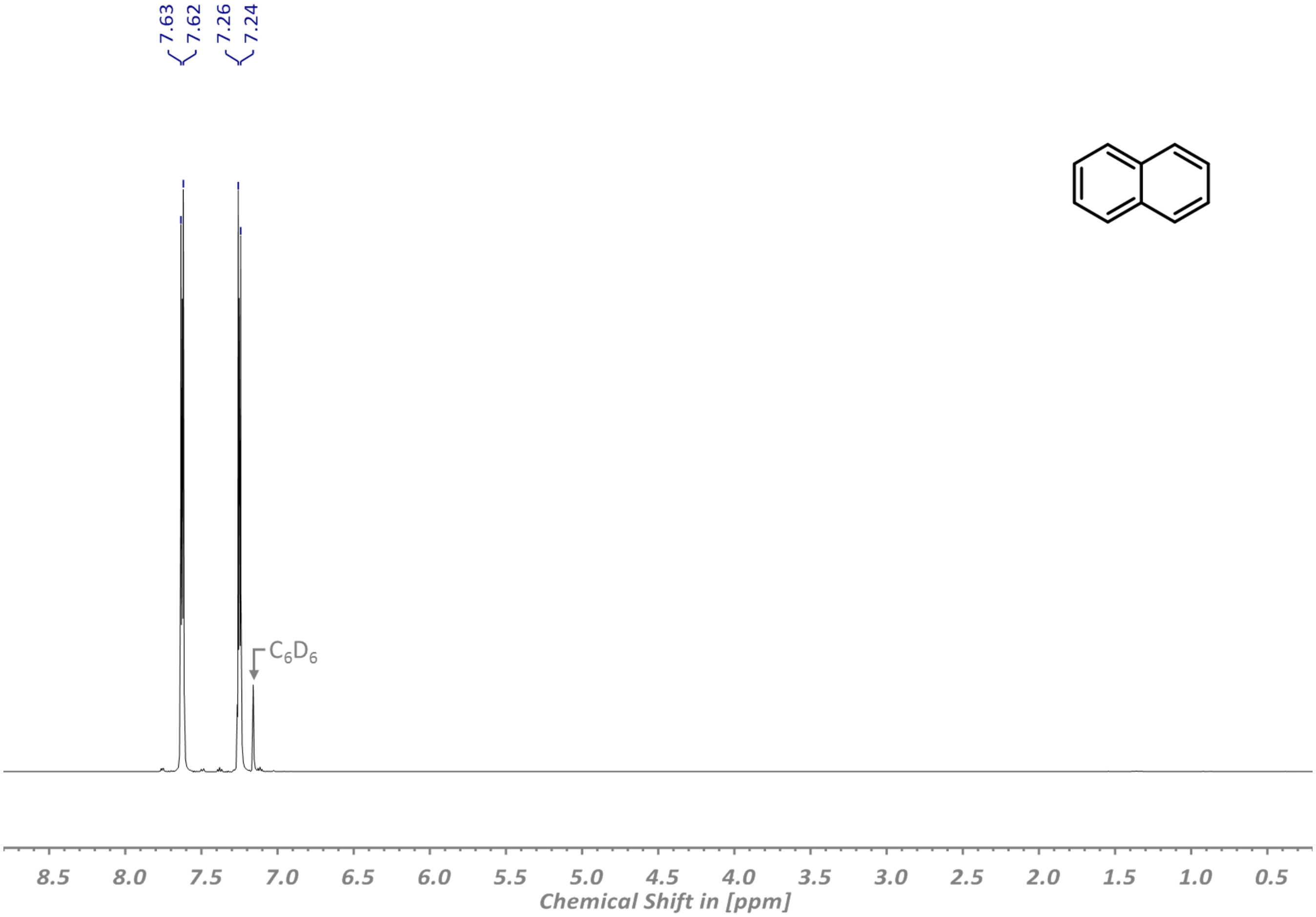

Figure S192. ^{1}H NMR spectrum (600 MHz, C_6D_6, 25 °C) of naphthalene.

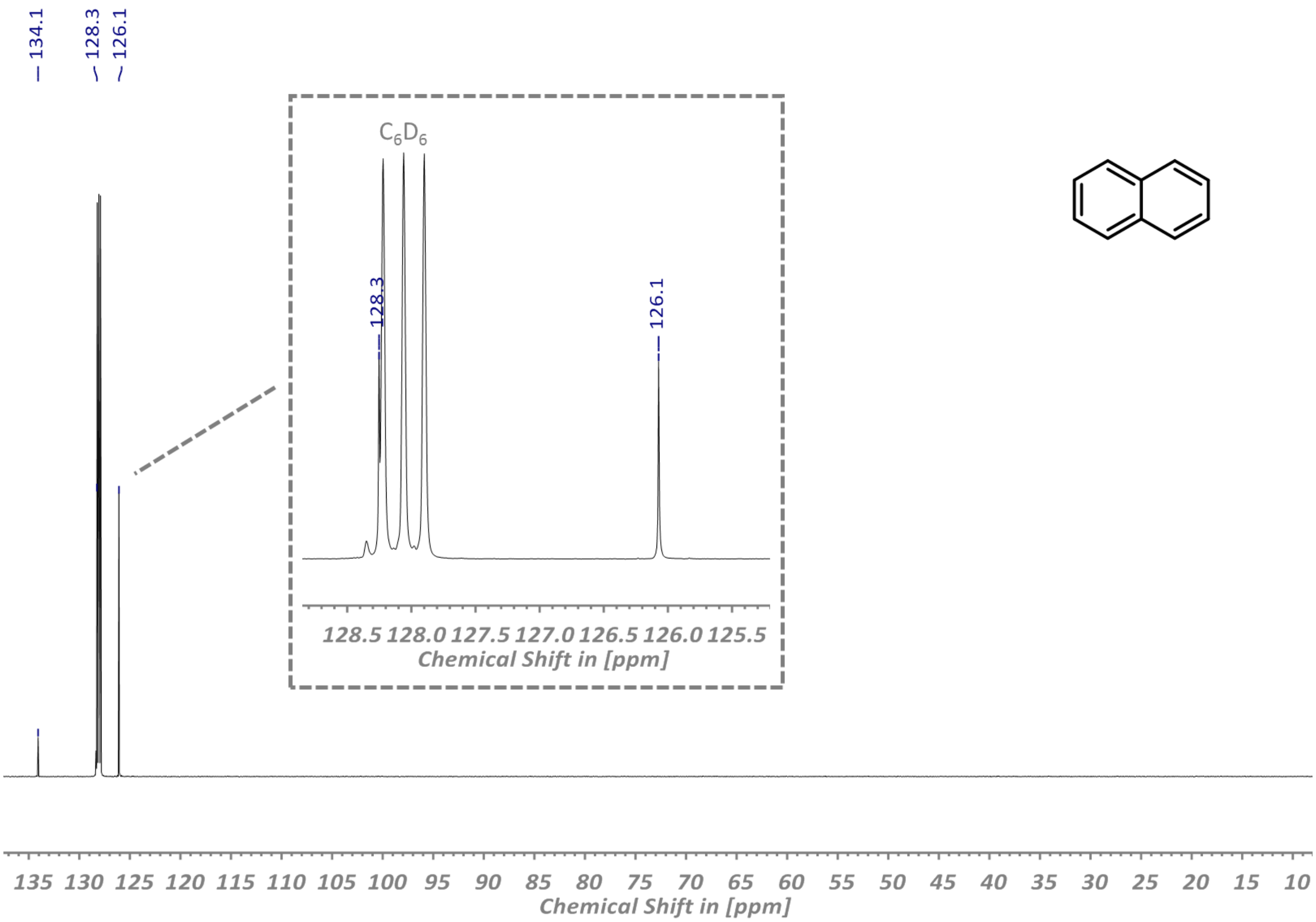

Figure S193. $^{13}C\{^1H\}$ NMR spectrum (151 MHz, C_6D_6, 25 °C) of naphthalene.

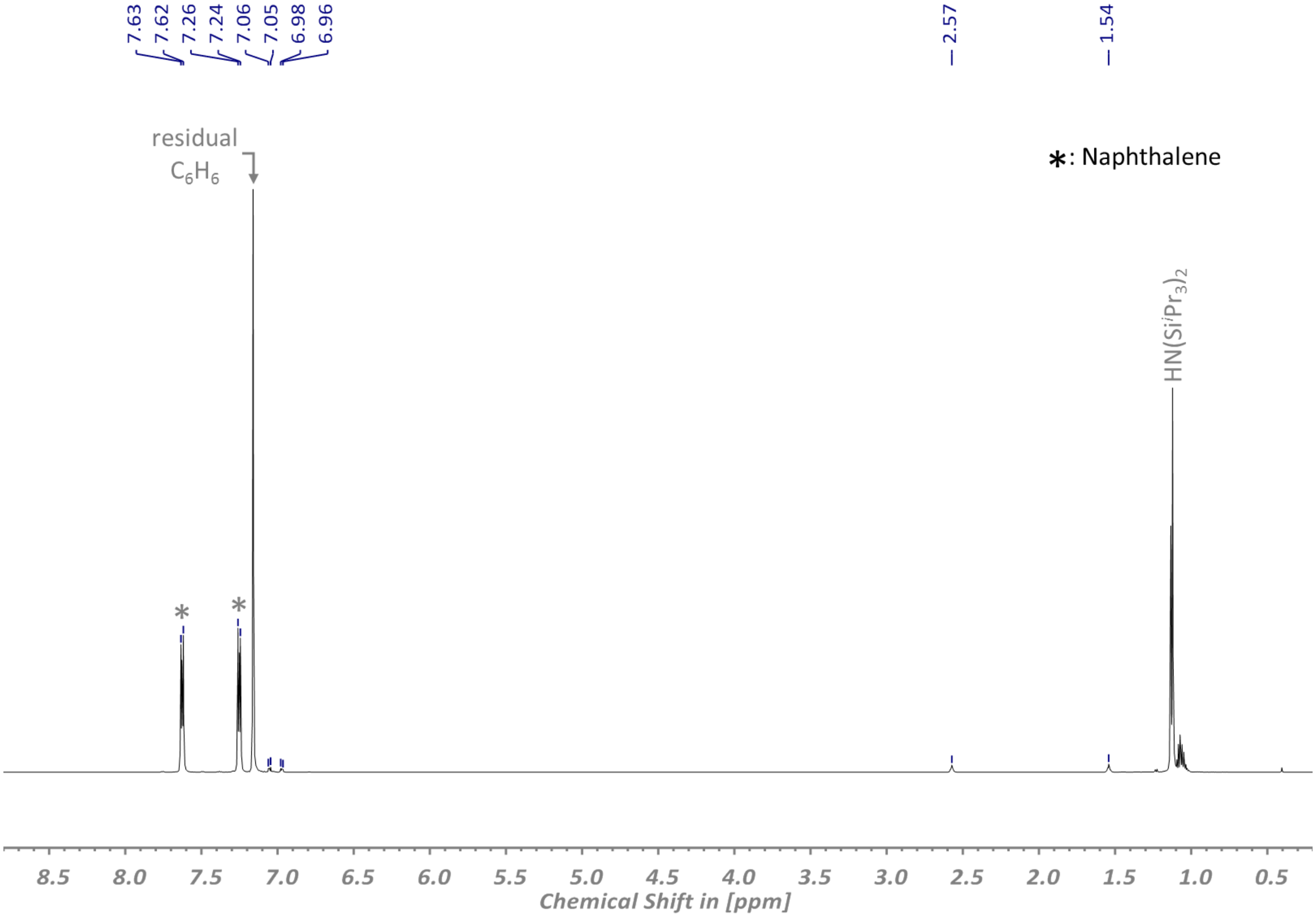

Figure S194. ^1H NMR spectrum (600 MHz, C_6D_6, 25 °C) after catalytic hydrogenation (24 h) of naphthalene (∗) with $Ca[N(Si^iPr_3)_2]_2$ (**1-Ca**) (10 mol%) and H_2 (12 bar) at 120 °C showing the formation of tetralin in a substoichiometric amount (Table S4, entry 11).

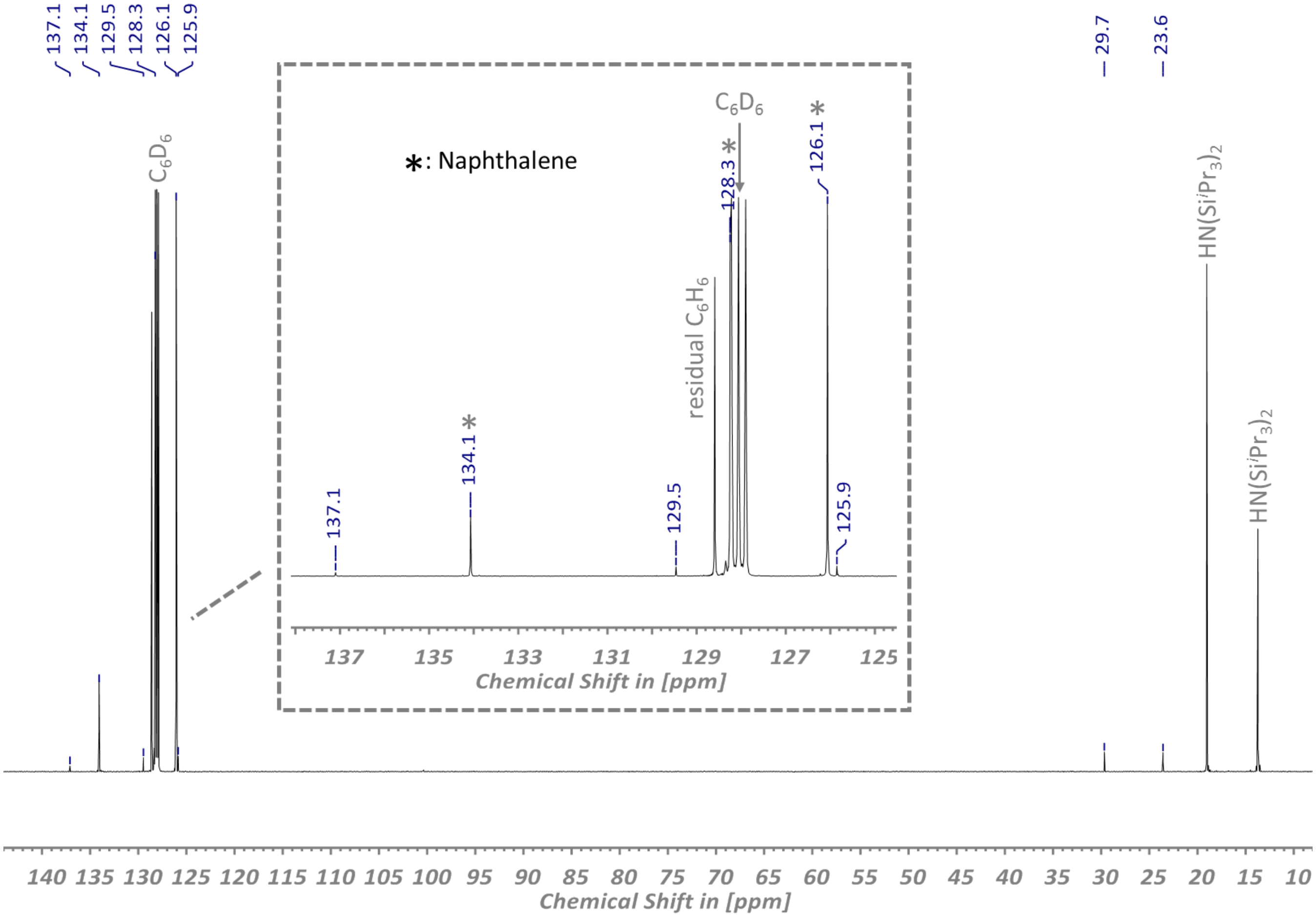

Figure S195. $^{13}C\{^{1}H\}$ NMR spectrum (151 MHz, C_6D_6, 25 °C) after catalytic hydrogenation (24 h) of naphthalene (∗) with $Ca[N(Si^{i}Pr_3)_2]_2$ (**1-Ca**) (10 mol%) and H_2 (12 bar) at 120 °C showing the formation of tetralin in a substoichiometric amount (Table S4, entry 11).

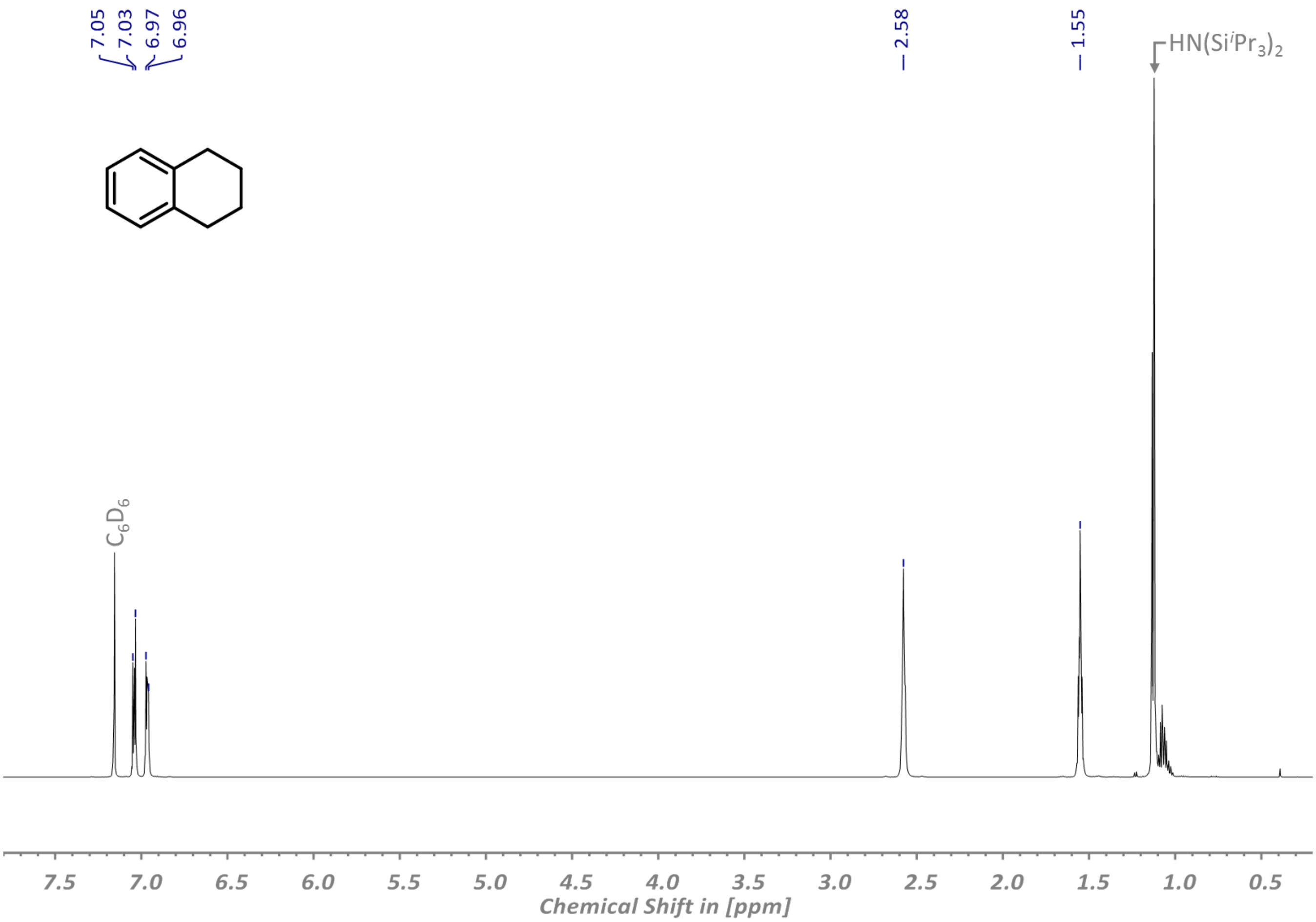

Figure S196. ^{1}H NMR spectrum (600 MHz, C_6D_6, 25 °C) of tetralin after catalytic hydrogenation (24 h) of naphthalene with Sr[N(Si^{i}Pr$_3$)$_2$]$_2$ (**1-Sr**) (10 mol%) and H_2 (12 bar) at 120 °C (Table S4, entry 12).

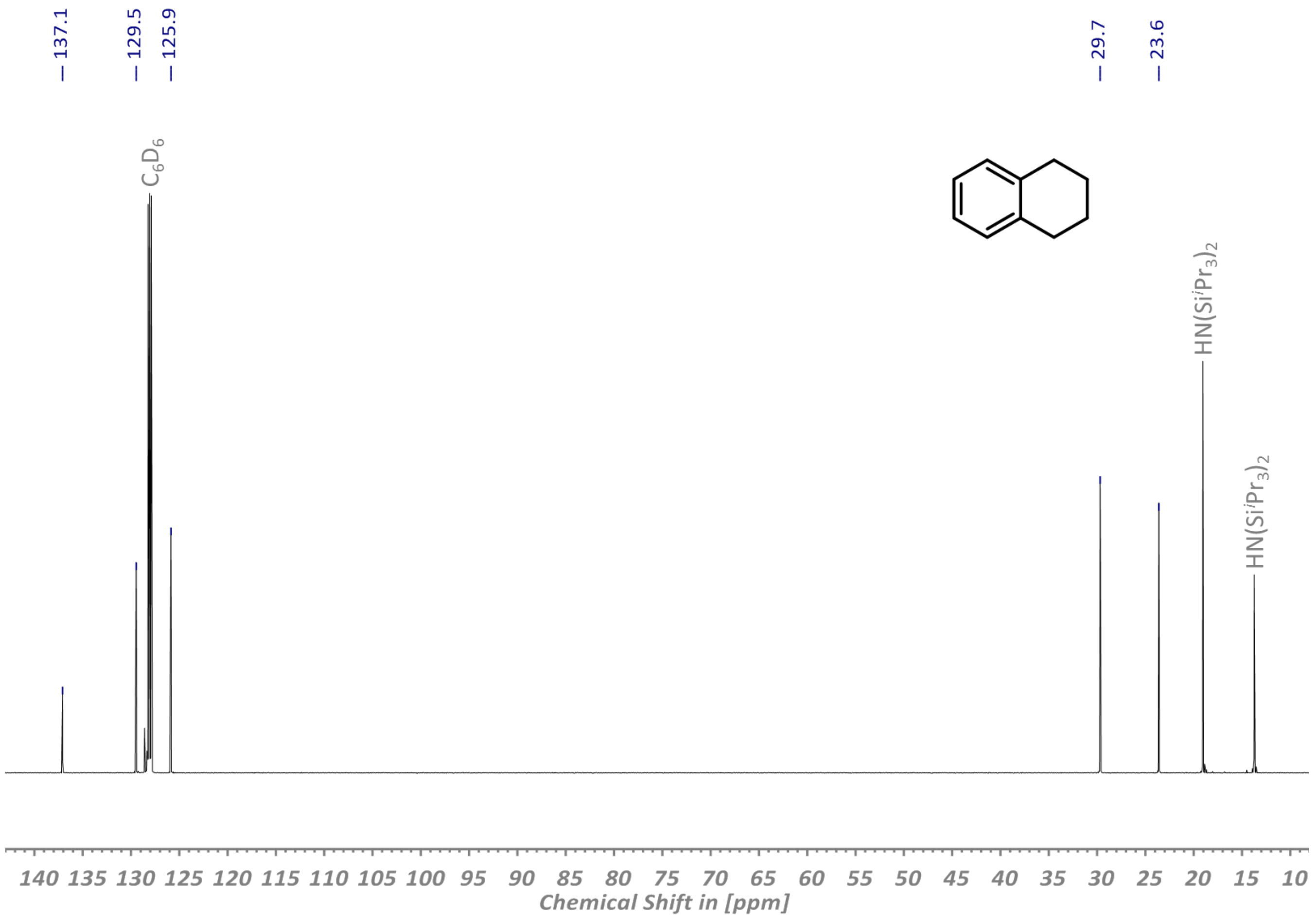

Figure S197. $^{13}C\{^1H\}$ NMR spectrum (151 MHz, C_6D_6, 25 °C) of tetralin after catalytic hydrogenation (24 h) of naphthalene with $Sr[N(Si^iPr_3)_2]_2$ (**2-Sr**) (10 mol%) and H_2 (12 bar) at 120 °C (Table S4, entry 12).

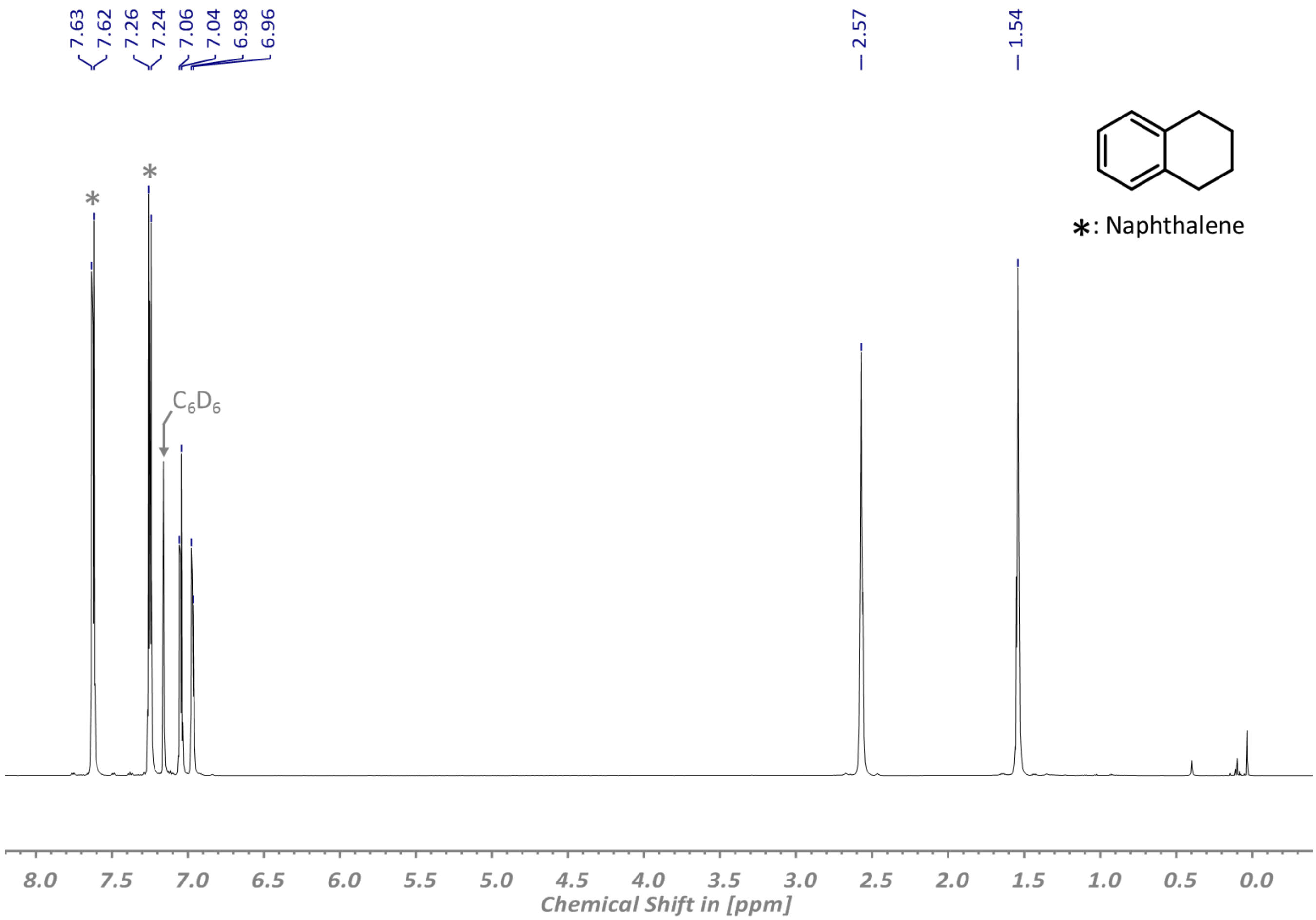

Figure S198. ^{1}H NMR spectrum (600 MHz, C_6D_6, 25 °C) after catalytic hydrogenation (24 h) of naphthalene (∗) with $Ba[N(SiMe_3)_2]_2$ (10 mol%) and H_2 (12 bar) at 120 °C showing the formation of tetralin (Table S4, entry 13).

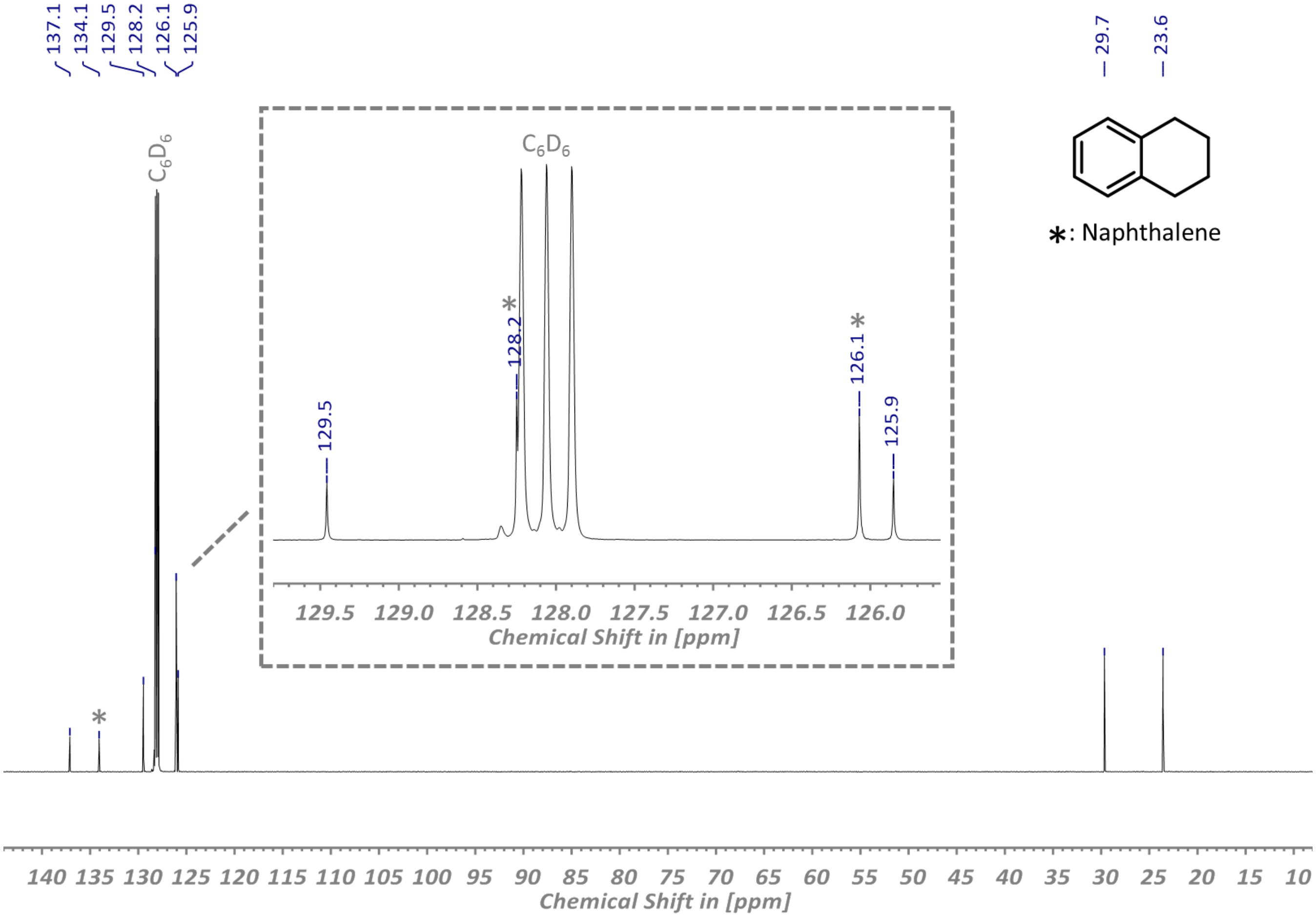

Figure S199. $^{13}C\{^{1}H\}$ NMR spectrum (151 MHz, C_6D_6, 25 °C) after catalytic hydrogenation (24 h) of naphthalene (∗) with $Ba[N(SiMe_3)_2]_2$ (10 mol%) and H_2 (12 bar) at 120 °C showing the formation of tetralin (Table S4, entry 13).

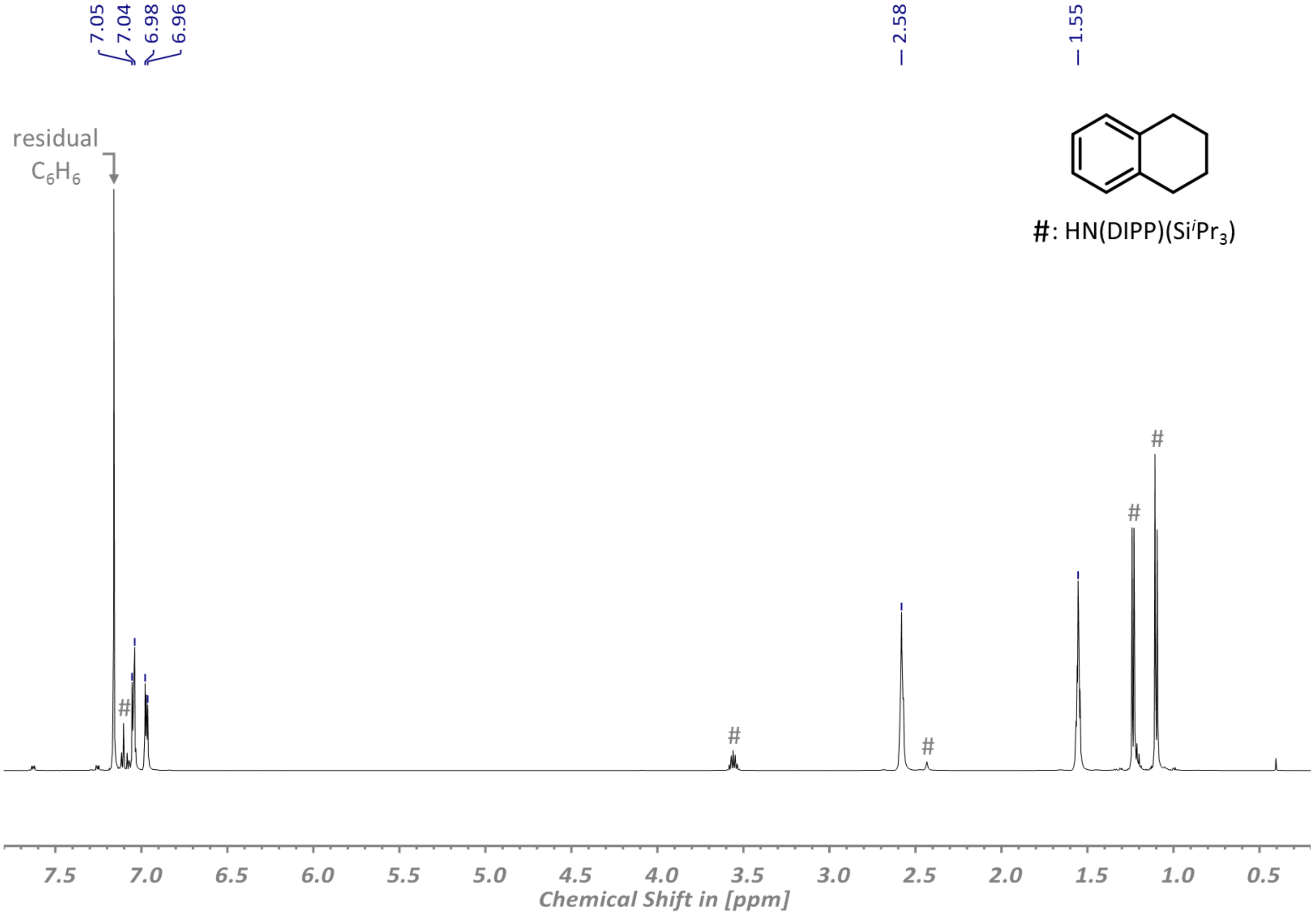

Figure S200. ^{1}H NMR spectrum (600 MHz, C_6D_6, 25 °C) of tetralin after catalytic hydrogenation (24 h) of naphthalene with Ba[N(DIPP)(Si^{i}Pr$_3$)]$_2$ (**2-Ba**) (10 mol%) and H_2 (12 bar) at 120 °C. *Note:* # denotes HN(DIPP)(Si^{i}Pr$_3$) (Table S4, entry 14).

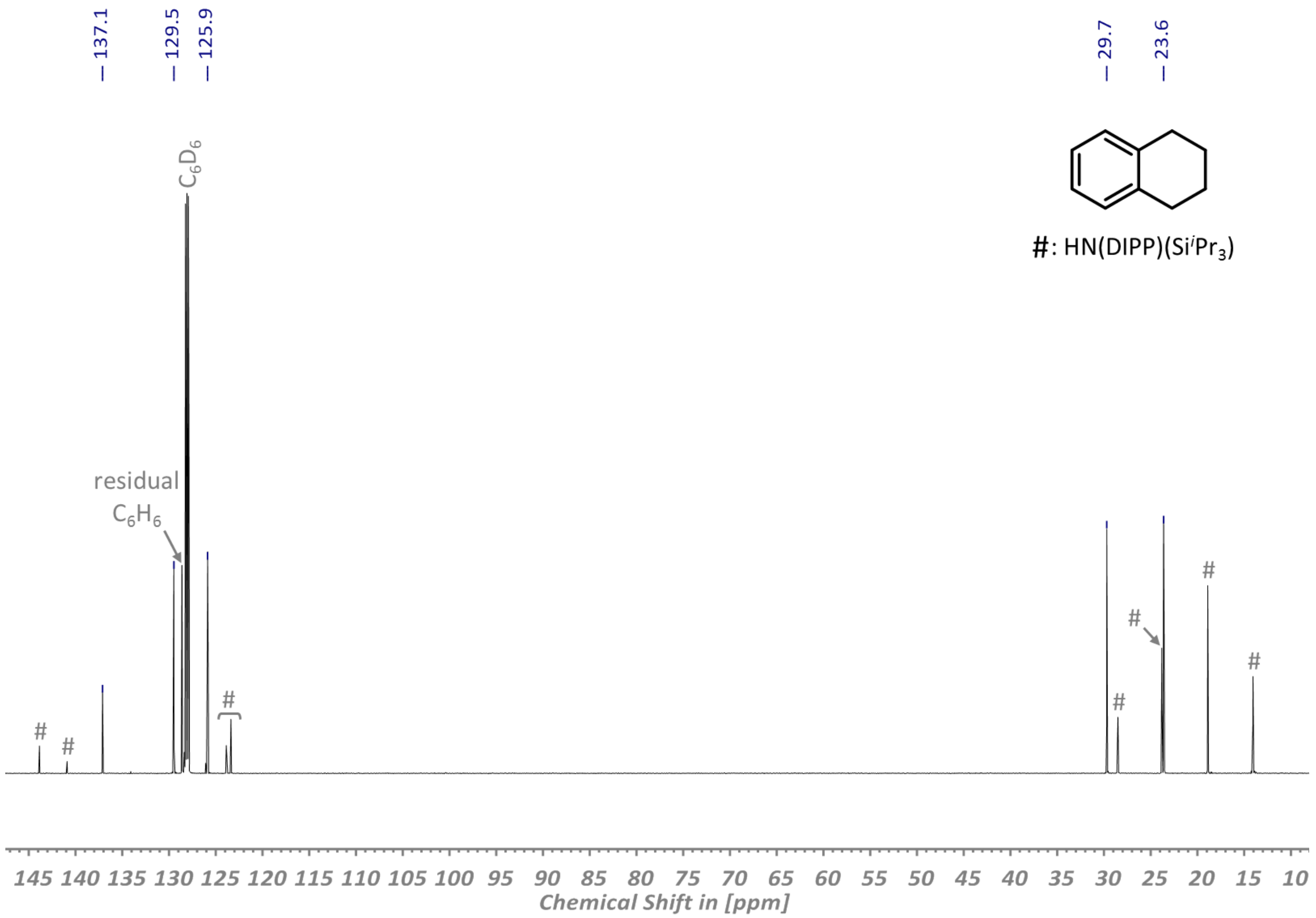

Figure S201. $^{13}C\{^1H\}$ NMR spectrum (151 MHz, C_6D_6, 25 °C) of tetralin after catalytic hydrogenation (24 h) of naphthalene with Ba[N(DIPP)(Si^{i}Pr$_3$)]$_2$ (**2-Ba**) (10 mol%) and H_2 (12 bar) at 120 °C. *Note:* # denotes HN(DIPP)(Si^{i}Pr$_3$) (Table S4, entry 14).

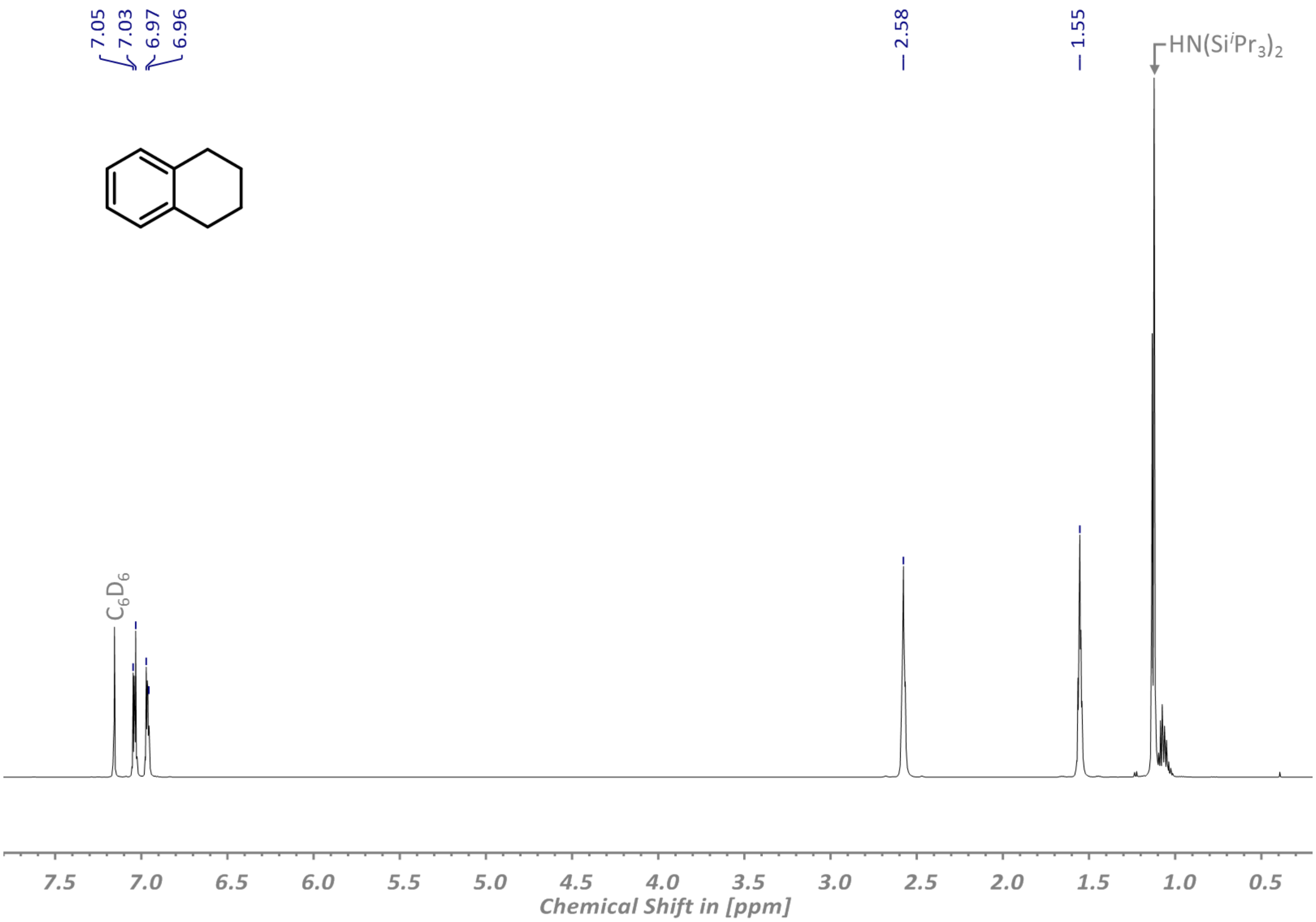

Figure S202. ^{1}H NMR spectrum (600 MHz, C_6D_6, 25 °C) of tetralin after catalytic hydrogenation (2 h) of naphthalene with Ba[N(Si^{i}Pr$_3$)$_2$]$_2$ (**1-Ba**) (10 mol%) and H_2 (12 bar) at 120 °C (Table S4, entry 15).

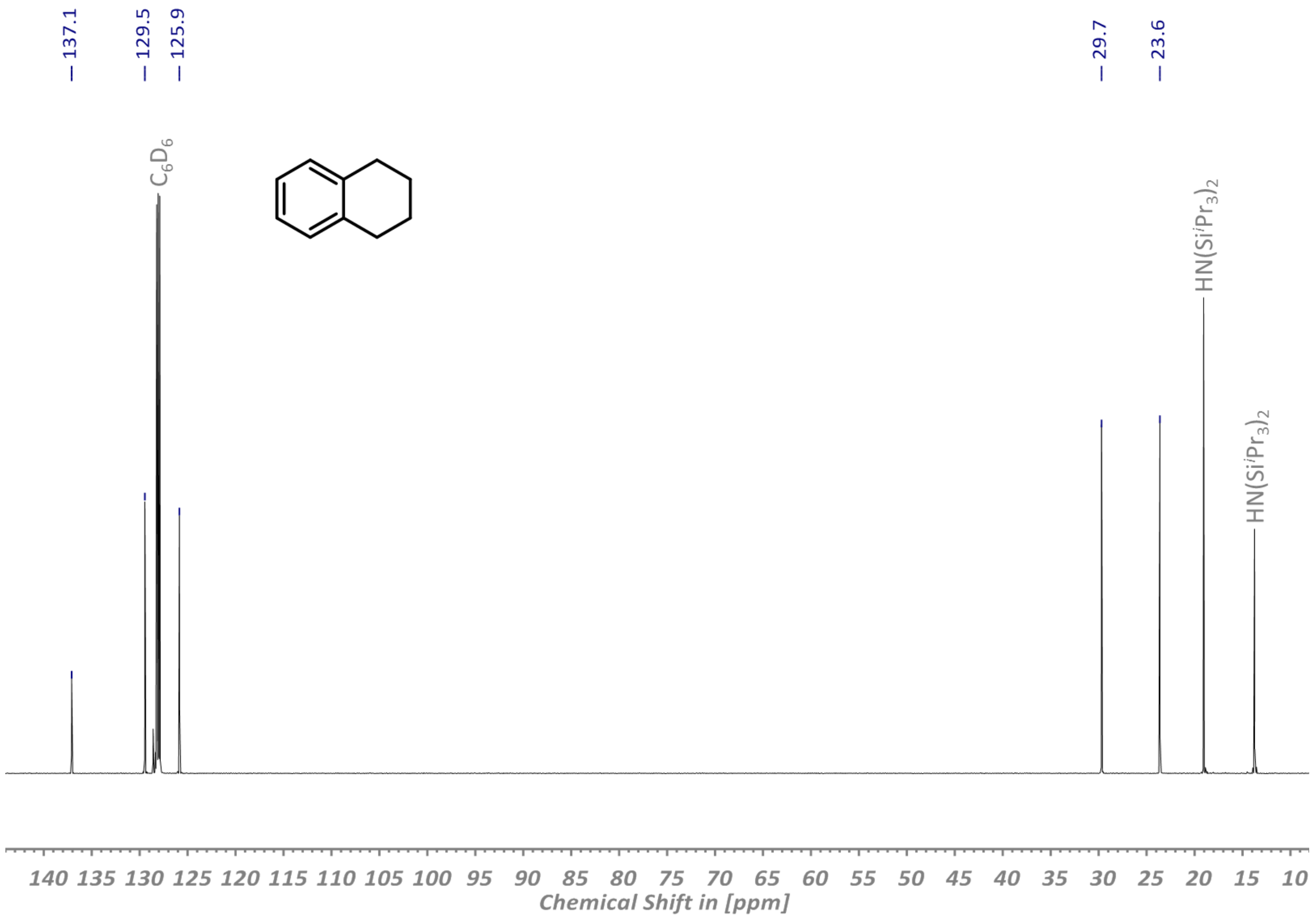

Figure S203. $^{13}C\{^1H\}$ NMR spectrum (151 MHz, C_6D_6, 25 °C) of tetralin after catalytic hydrogenation (2 h) of naphthalene with $Ba[N(Si^iPr_3)_2]_2$ (**1-Ba**) (10 mol%) and H_2 (12 bar) at 120 °C (Table S4, entry 15).

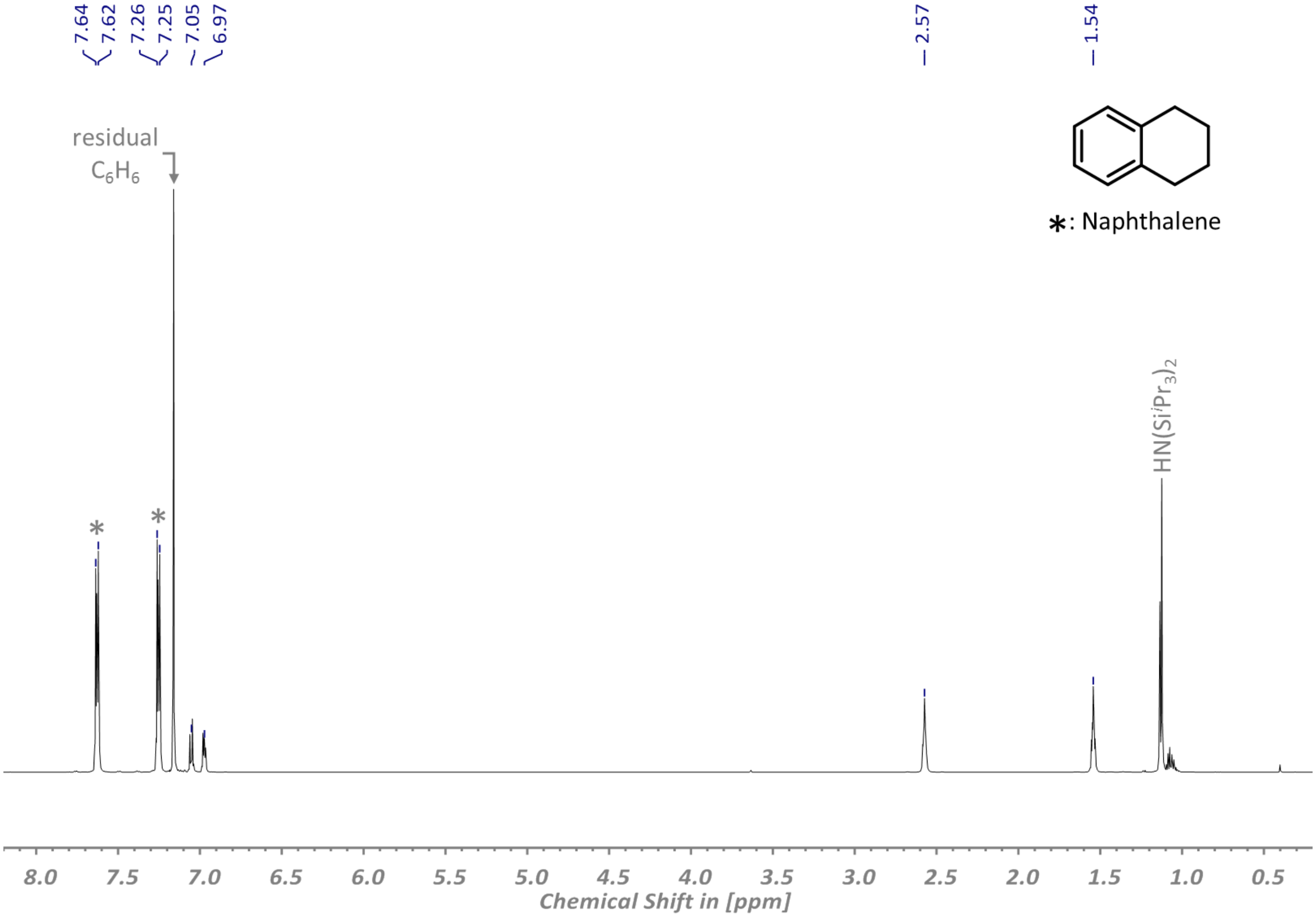

Figure S204. ^{1}H NMR spectrum (600 MHz, C_6D_6, 25 °C) after catalytic hydrogenation (24 h) of naphthalene (∗) with $Ba[N(Si^{i}Pr_3)_2]_2$ (**1-Ba**) (2.5 mol%) and H_2 (12 bar) at 120 °C showing the formation of tetralin (Table S4, entry 16).

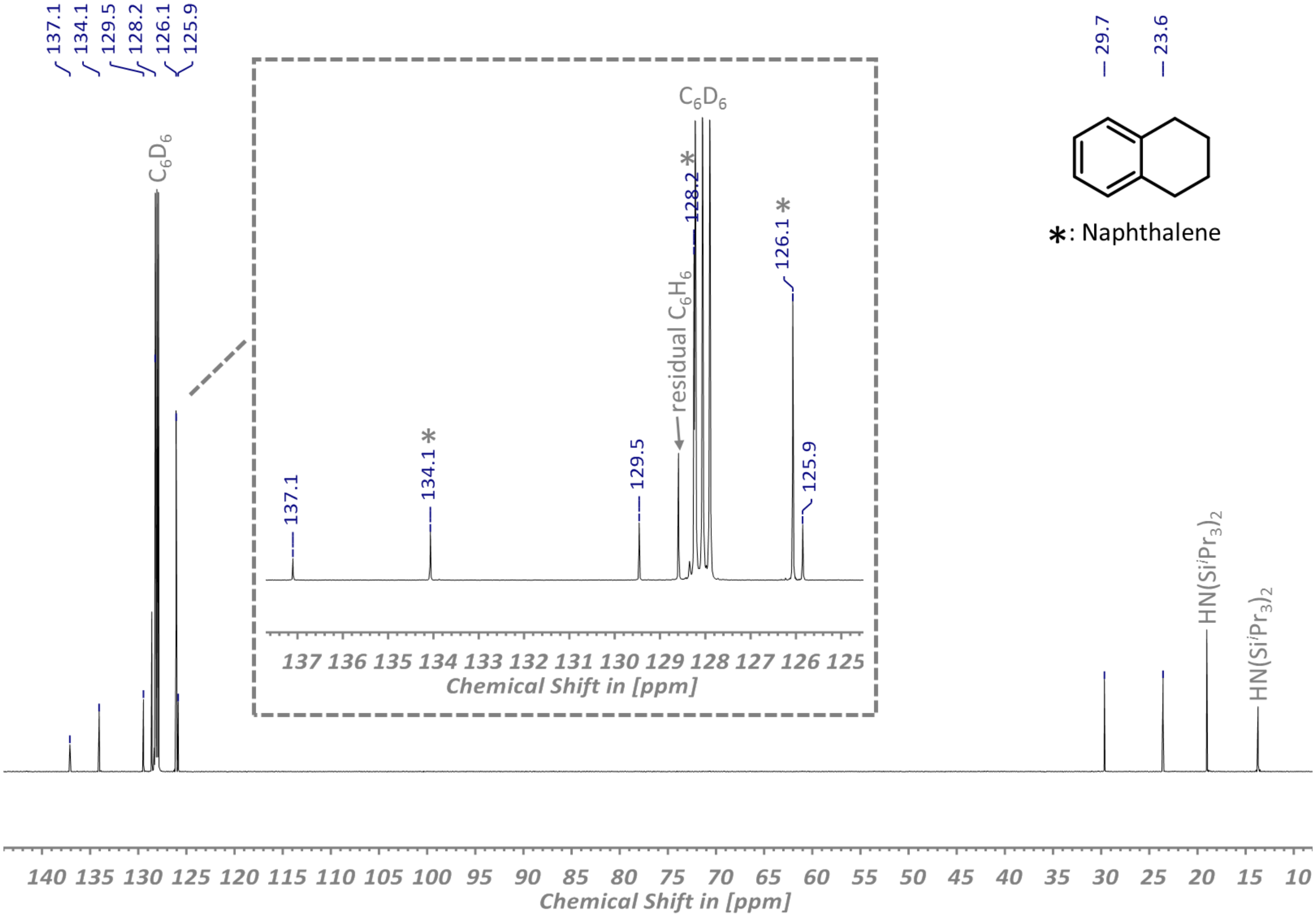

Figure S205. $^{13}C\{^{1}H\}$ NMR spectrum (151 MHz, C_6D_6, 25 °C) after catalytic hydrogenation (24 h) of naphthalene (∗) with $Ba[N(Si^{i}Pr_3)_2]_2$ (**1-Ba**) (2.5 mol%) and H_2 (12 bar) at 120 °C showing the formation of tetralin (Table S4, entry 16).

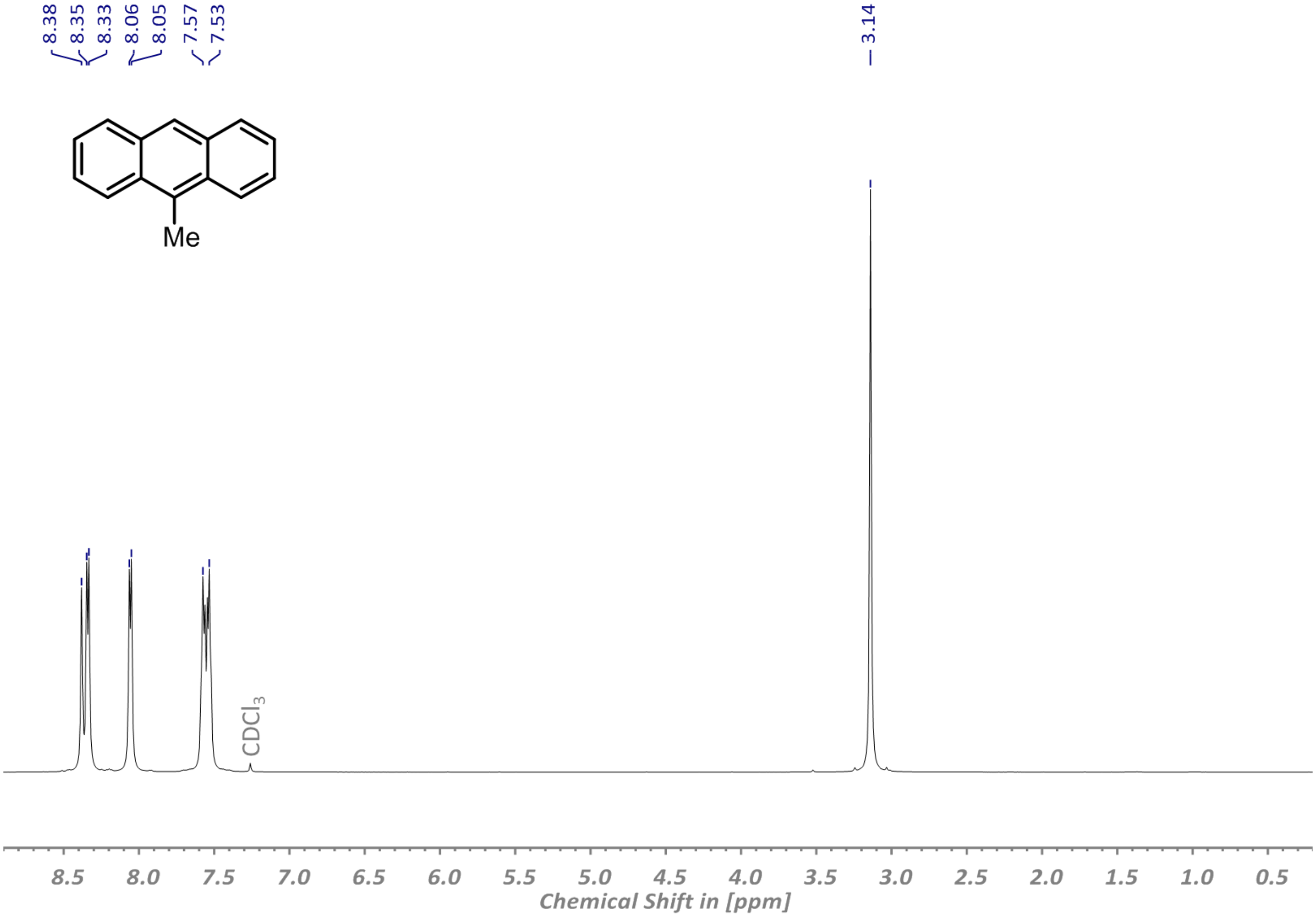

Figure S206. ^{1}H NMR spectrum (600 MHz, $CDCl_3$, 25 °C) of 9-methylanthracene.

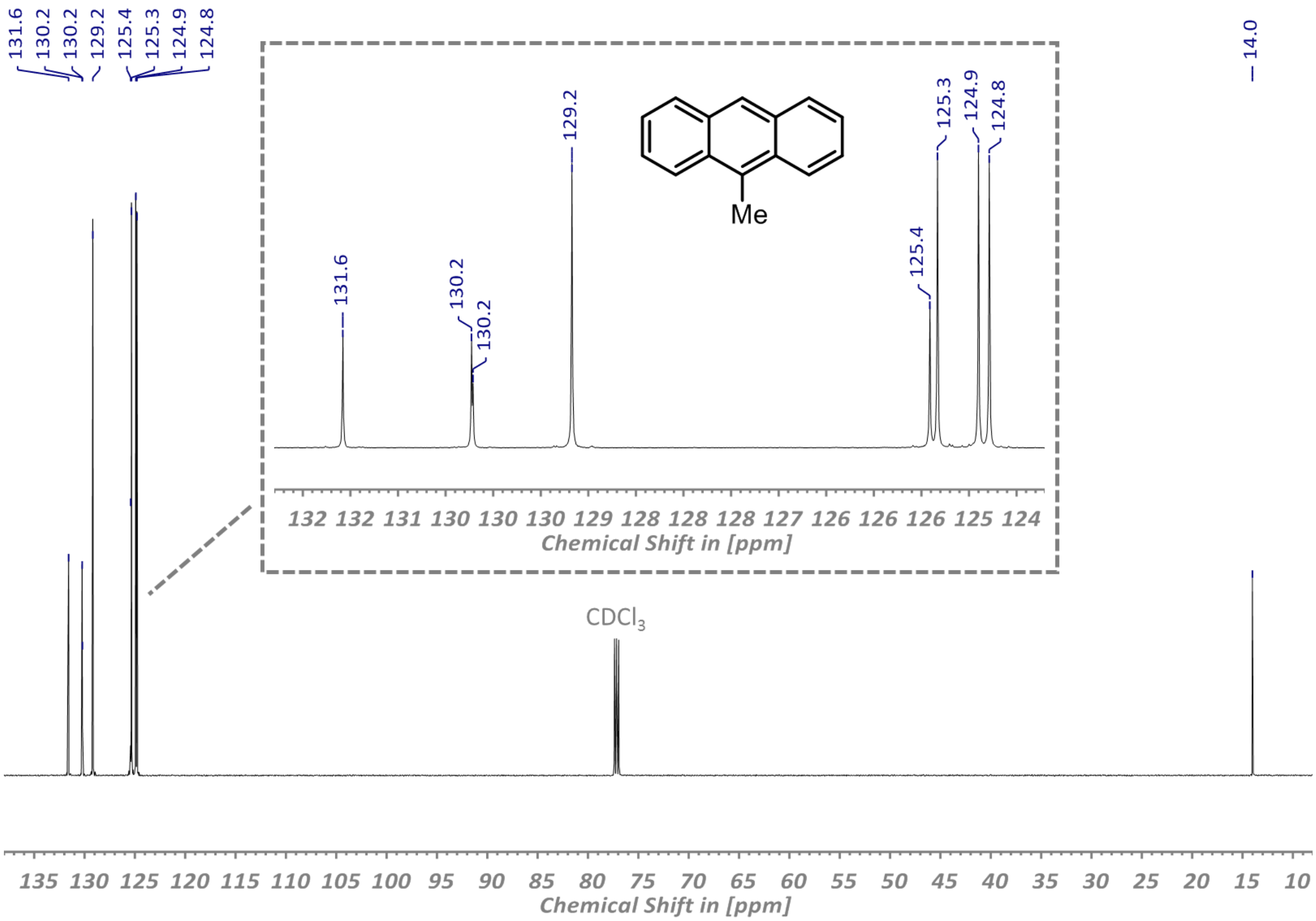

Figure S207. $^{13}C\{^{1}H\}$ NMR spectrum (151 MHz, $CDCl_3$, 25 °C) of 9-methylanthracene.

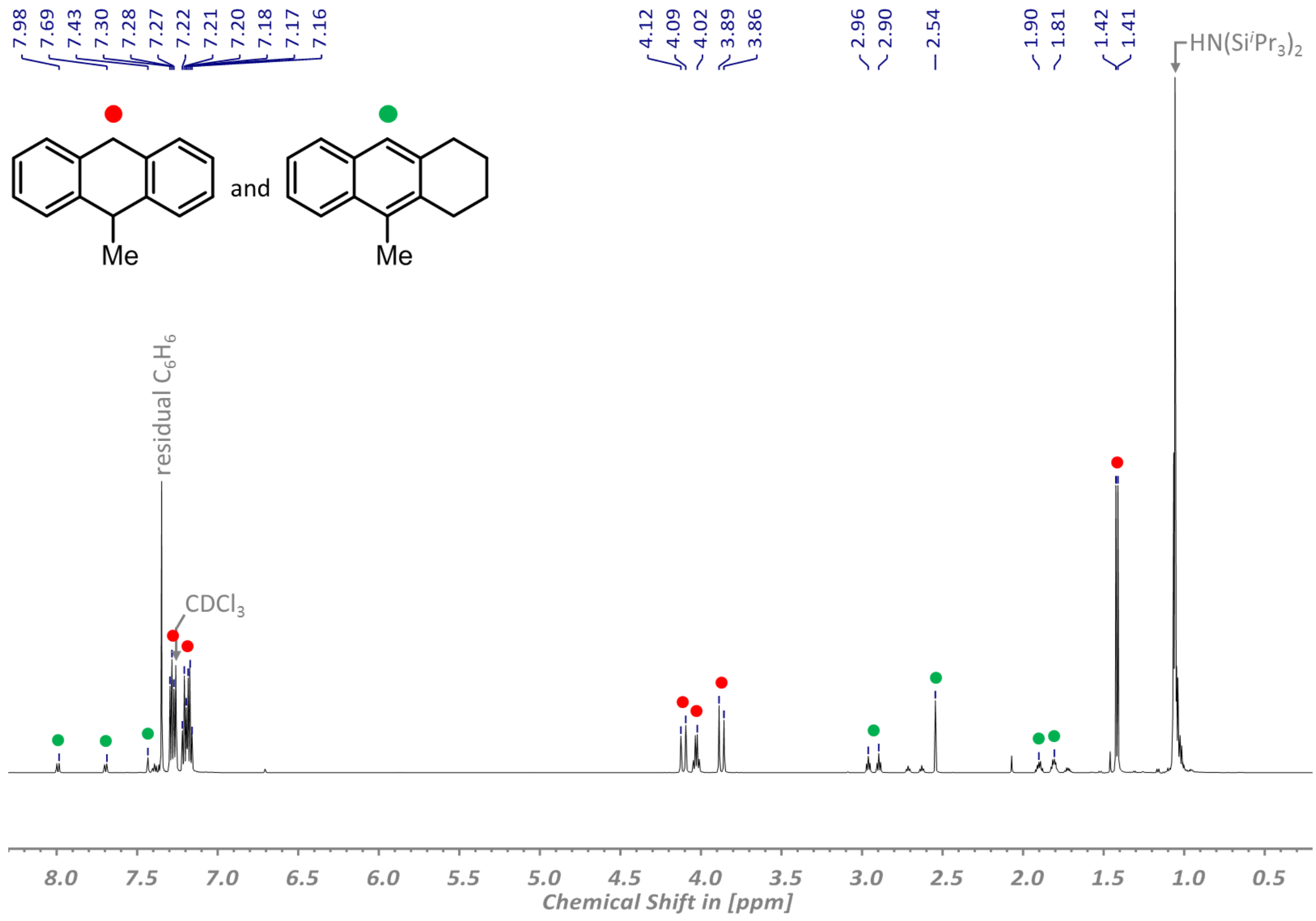

Figure S208. ^{1}H NMR spectrum (600 MHz, $CDCl_3$, 25 °C) after catalytic hydrogenation (24 h) of 9-methylanthracene with Ba[N(Si^{i}Pr$_3$)$_2$]$_2$ (**1-Ba**) (10 mol%) and H_2 (12 bar) at 120 °C showing the formation of 9-methyl-9,10-dihydroanthracene (●) and 9-methyl-1,2,3,4-tetrahydroanthracene (●). *Note:* Trace amounts of unidentified species are visible in the ^{1}H NMR spectrum. The conversion was determined from a further independent experiment by GC/MS analysis (Table S4, entry 17).

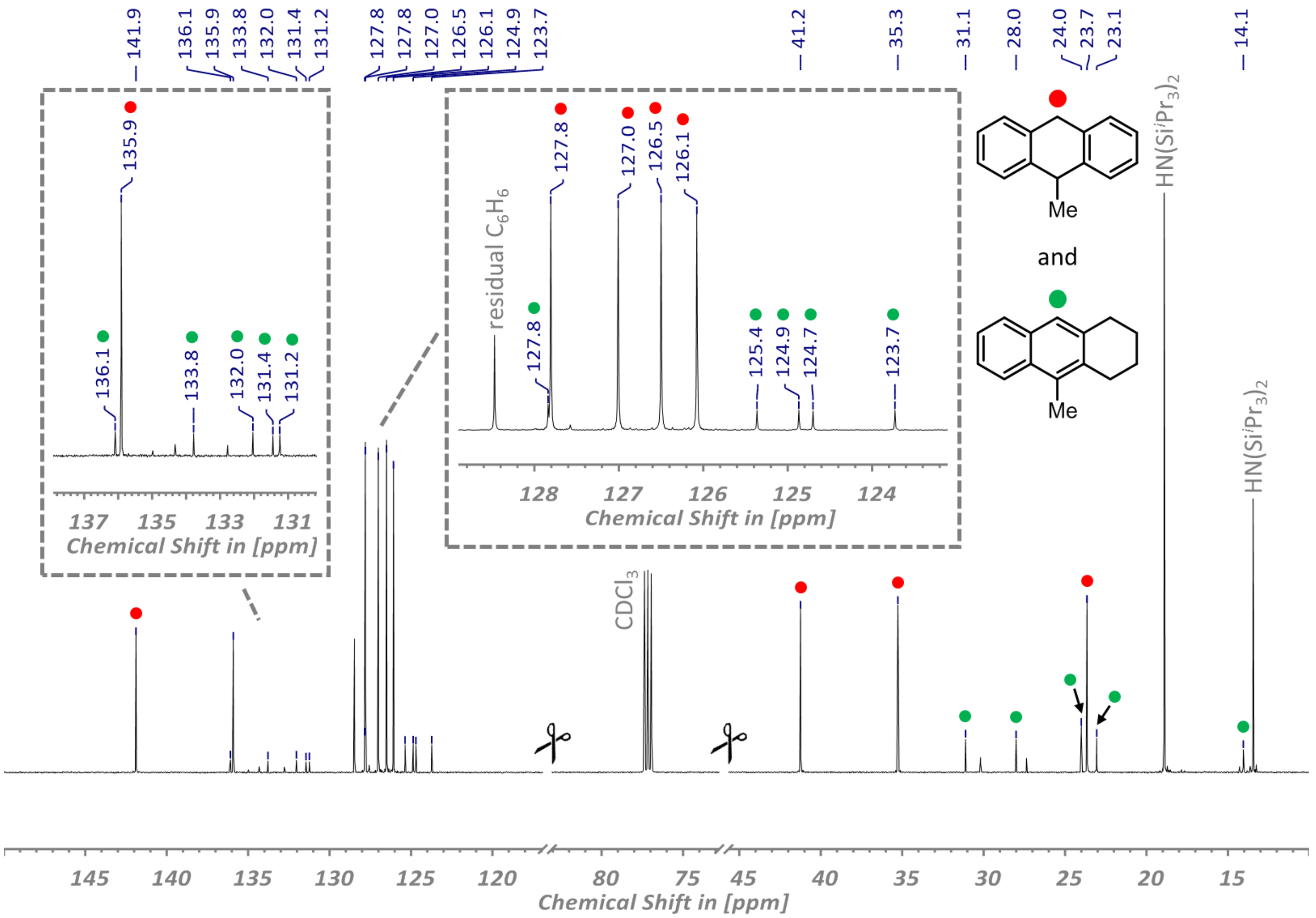

Figure S209. $^{13}C\{^1H\}$ NMR spectrum (151 MHz, $CDCl_3$, 25 °C) after catalytic hydrogenation (24 h) of 9-methylanthracene with Ba[N(Si^{i}Pr$_3$)$_2$]$_2$ (**1-Ba**) (10 mol%) and H_2 (12 bar) at 120 °C showing the formation of 9-methyl-9,10-dihydroanthracene (●) and 9-methyl-1,2,3,4-tetrahydroanthracene (●). *Note:* Trace amounts of unidentified species are observed in the $^{13}C\{^1H\}$ NMR spectrum. The exact conversion was determined from a further independent experiment by GC/MS analysis. The NMR data are in good agreement with those previously reported for both compounds[S22][S23] (Table S4, entry 17).

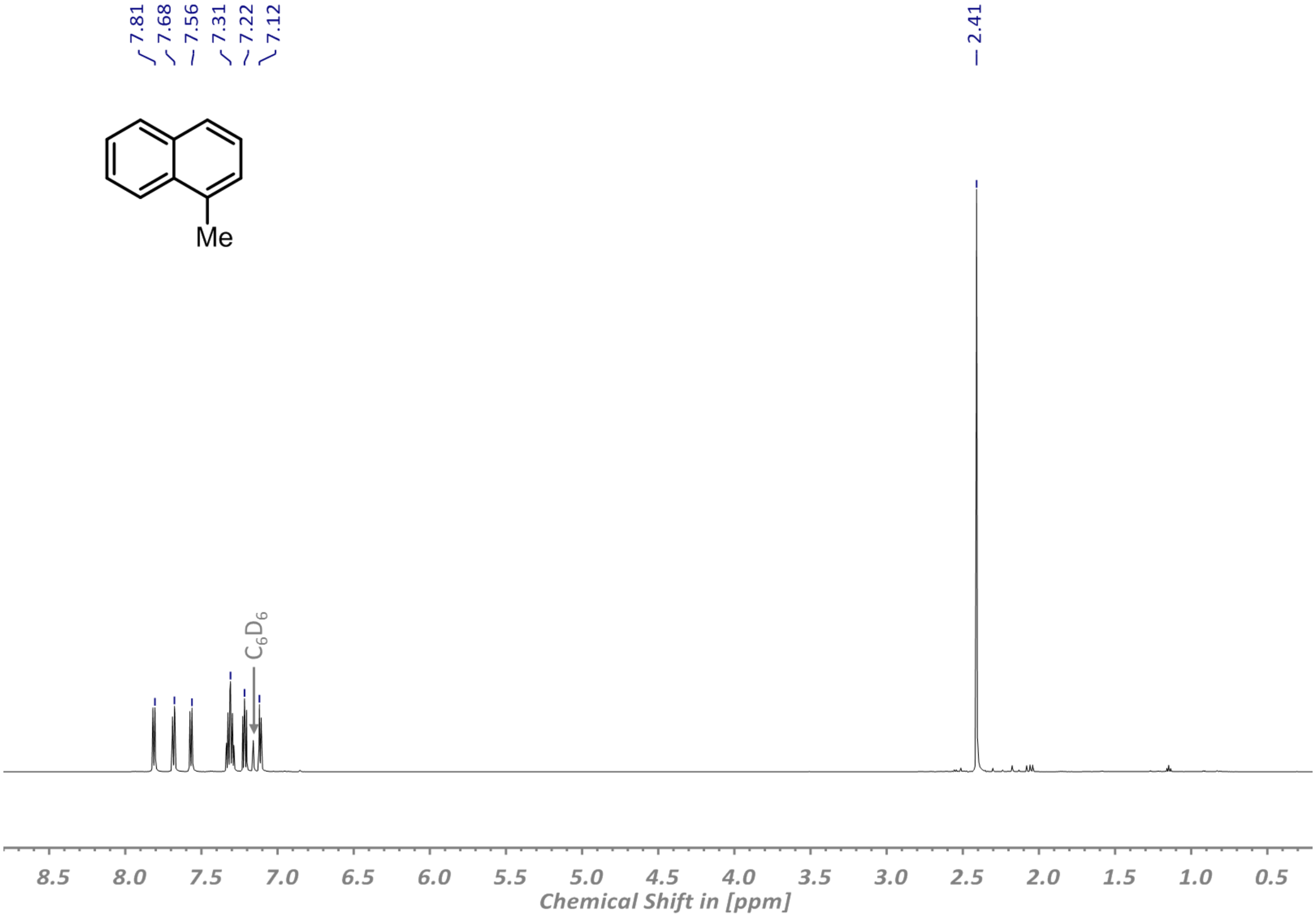

Figure S210. ^{1}H NMR spectrum (600 MHz, C_6D_6, 25 °C) of 1-methylnaphthalene.

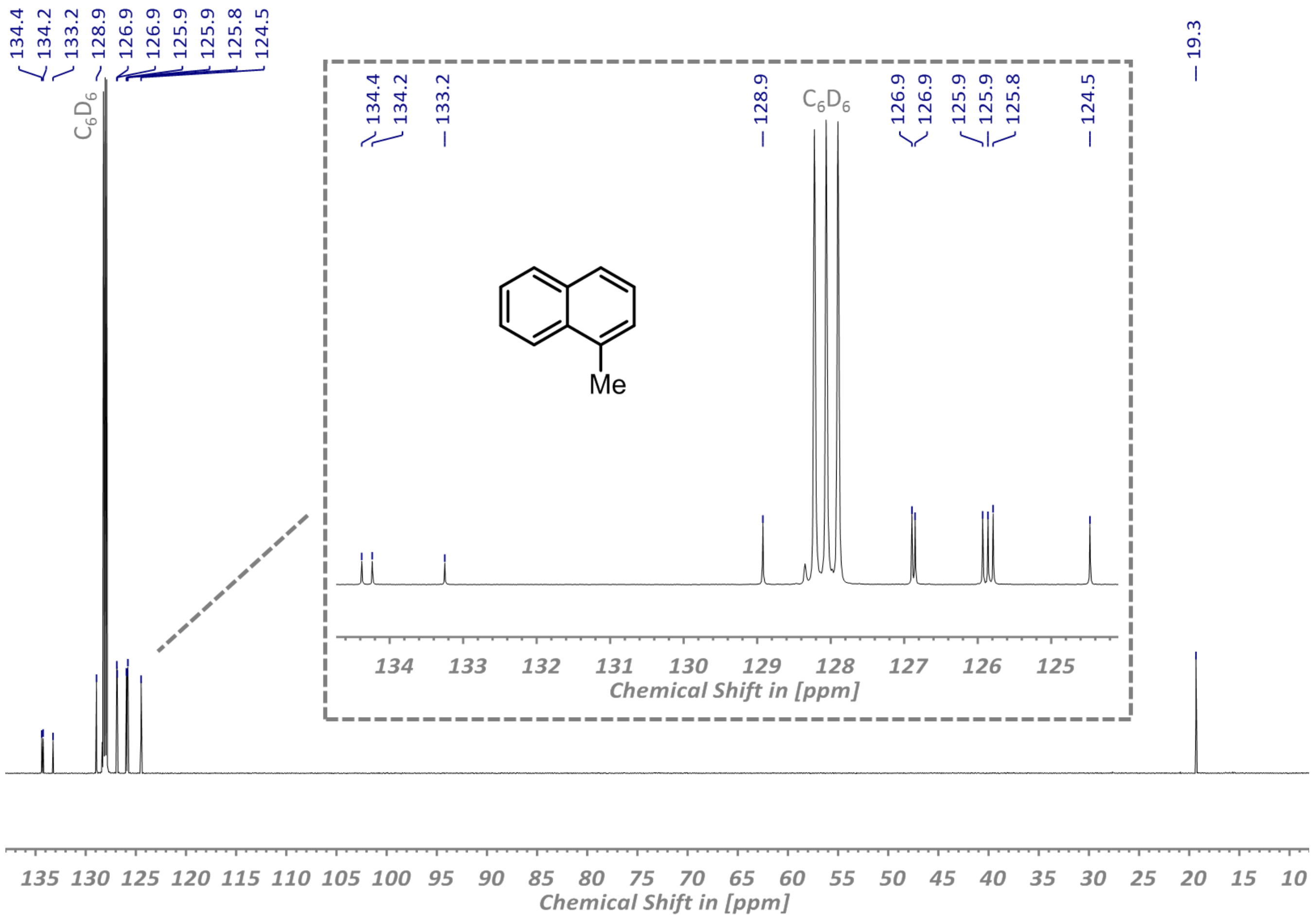

Figure S211. $^{13}C\{^1H\}$ NMR spectrum (151 MHz, C_6D_6, 25 °C) of 1-methylnaphthalene.

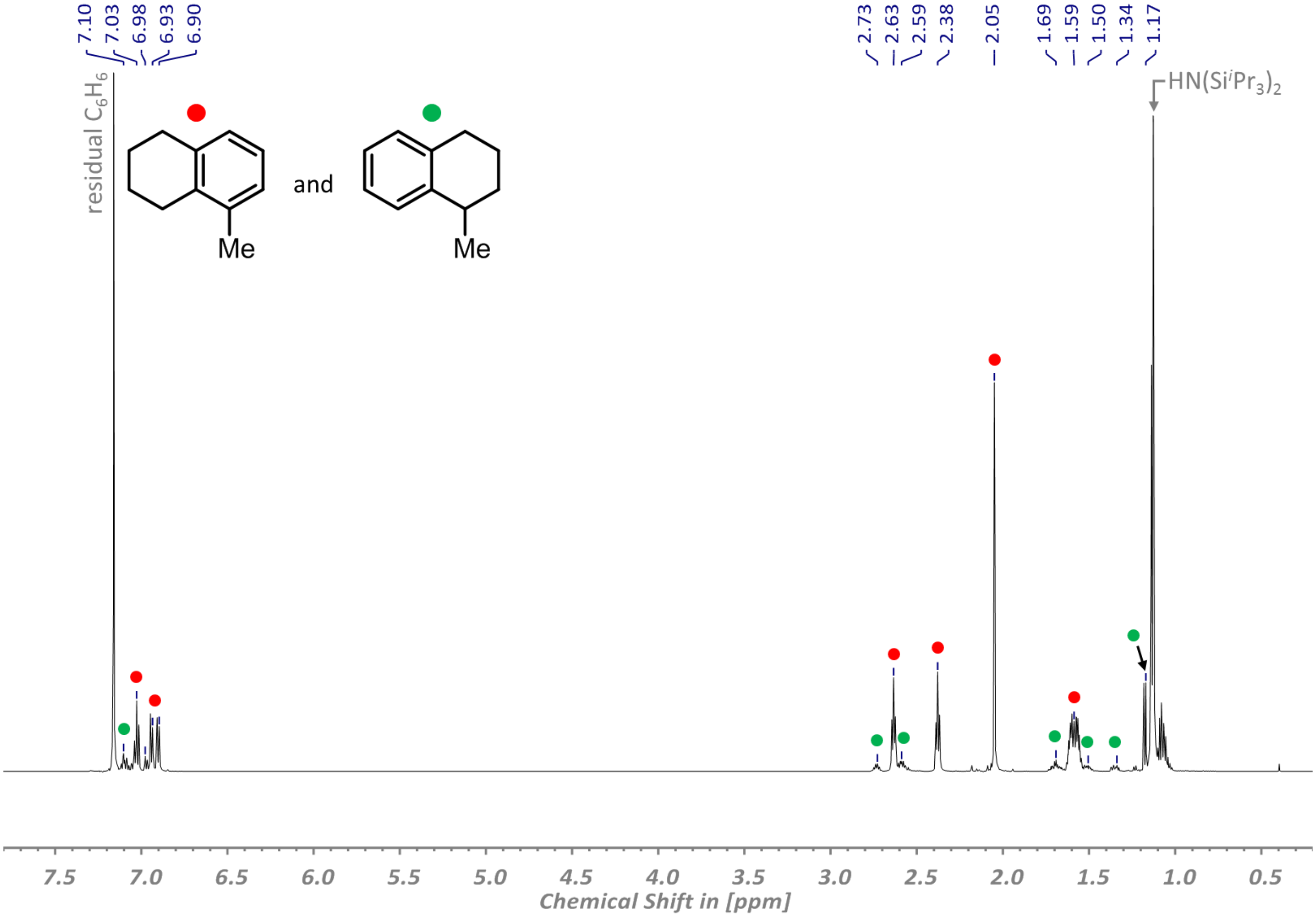

Figure S212. ^{1}H NMR spectrum (600 MHz, C_6D_6, 25 °C) after catalytic hydrogenation (24 h) of 1-methylnaphthalene with Ba[N(Si^{i}Pr$_3$)$_2$]$_2$ (**1-Ba**) (10 mol%) and H_2 (12 bar) at 120 °C showing the formation of 5-methyltetralin (●) and 1-methyltetralin (●) (Table S4, entry 18).

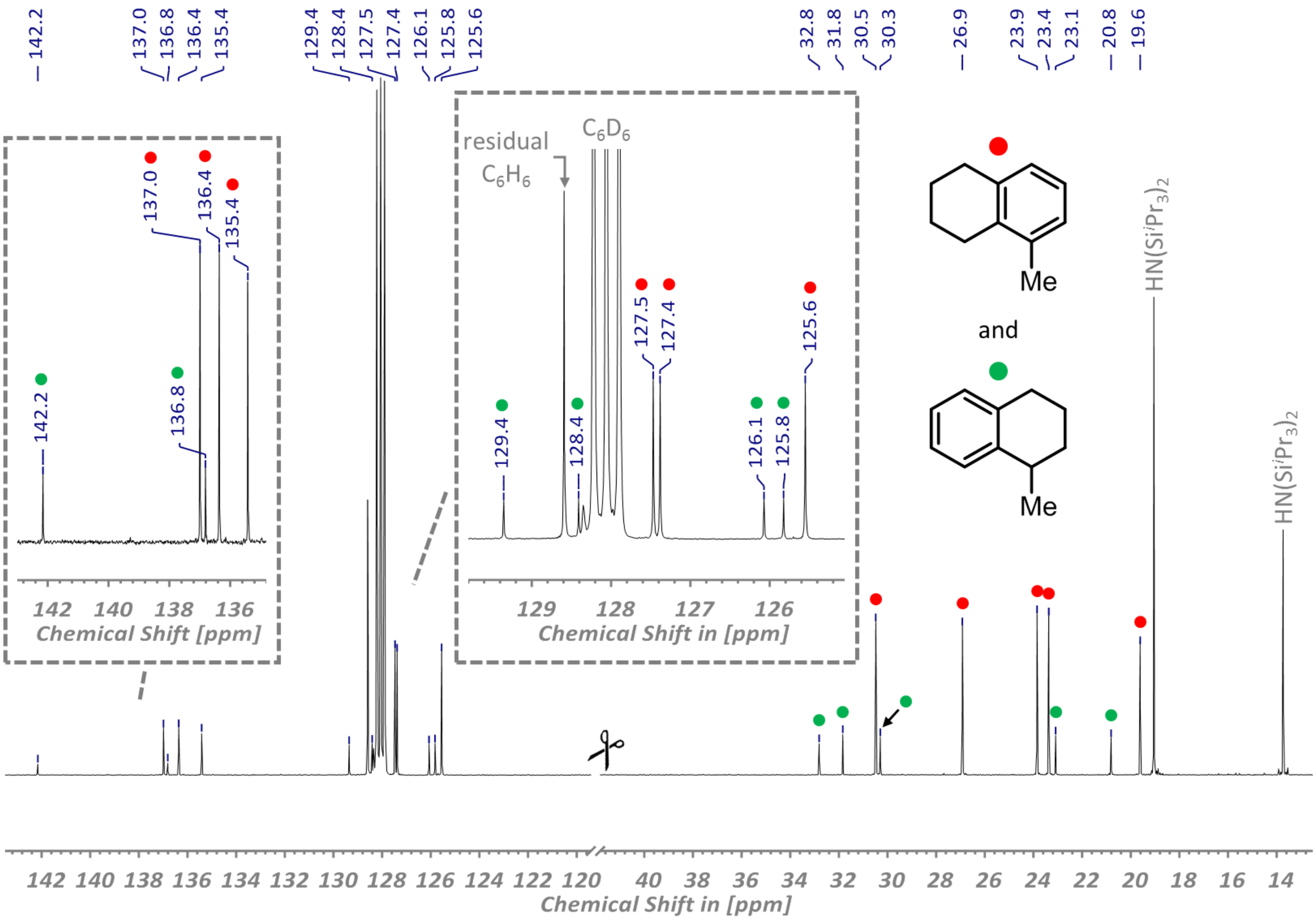

Figure S213. $^{13}C\{^1H\}$ NMR spectrum (151 MHz, C_6D_6, 25 °C) after catalytic hydrogenation (24 h) of 1-methylnaphthalene with Ba[N(Si^{i}Pr$_3$)$_2$]$_2$ (**1-Ba**) (10 mol%) and H_2 (12 bar) at 120 °C showing the formation of 5-methyltetralin (●) and 1-methyltetralin (●). *Note:* The obtained spectroscopic data for both compounds are in good agreement with the literature[S21][S24-26] (Table S4, entry 18).

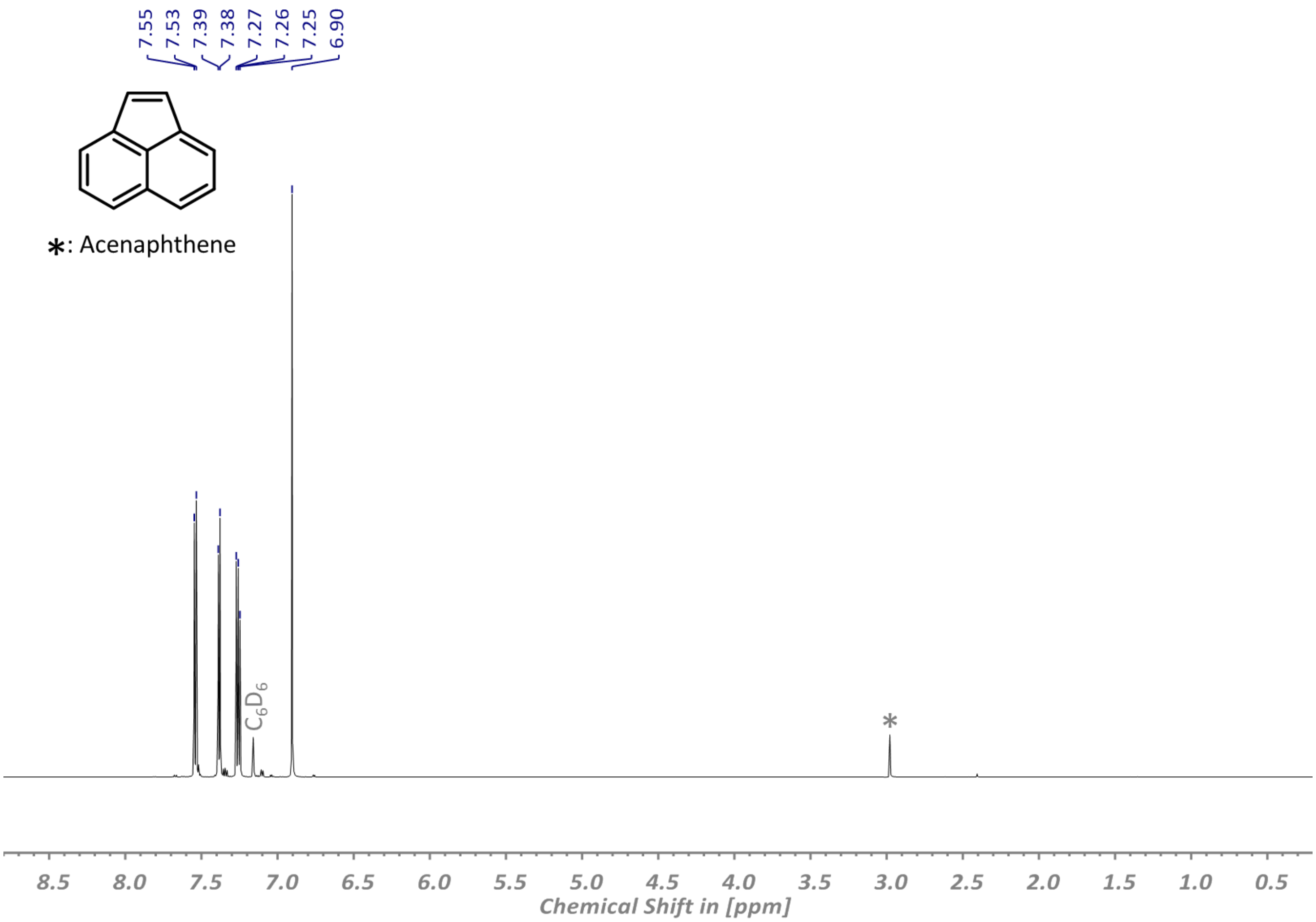

Figure S214. ^{1}H NMR spectrum (600 MHz, C_6D_6, 25 °C) of acenaphthylene (96%). *Note:* Trace impurities (4%) of acenaphthene (∗) are evidenced by GC/MS analysis.

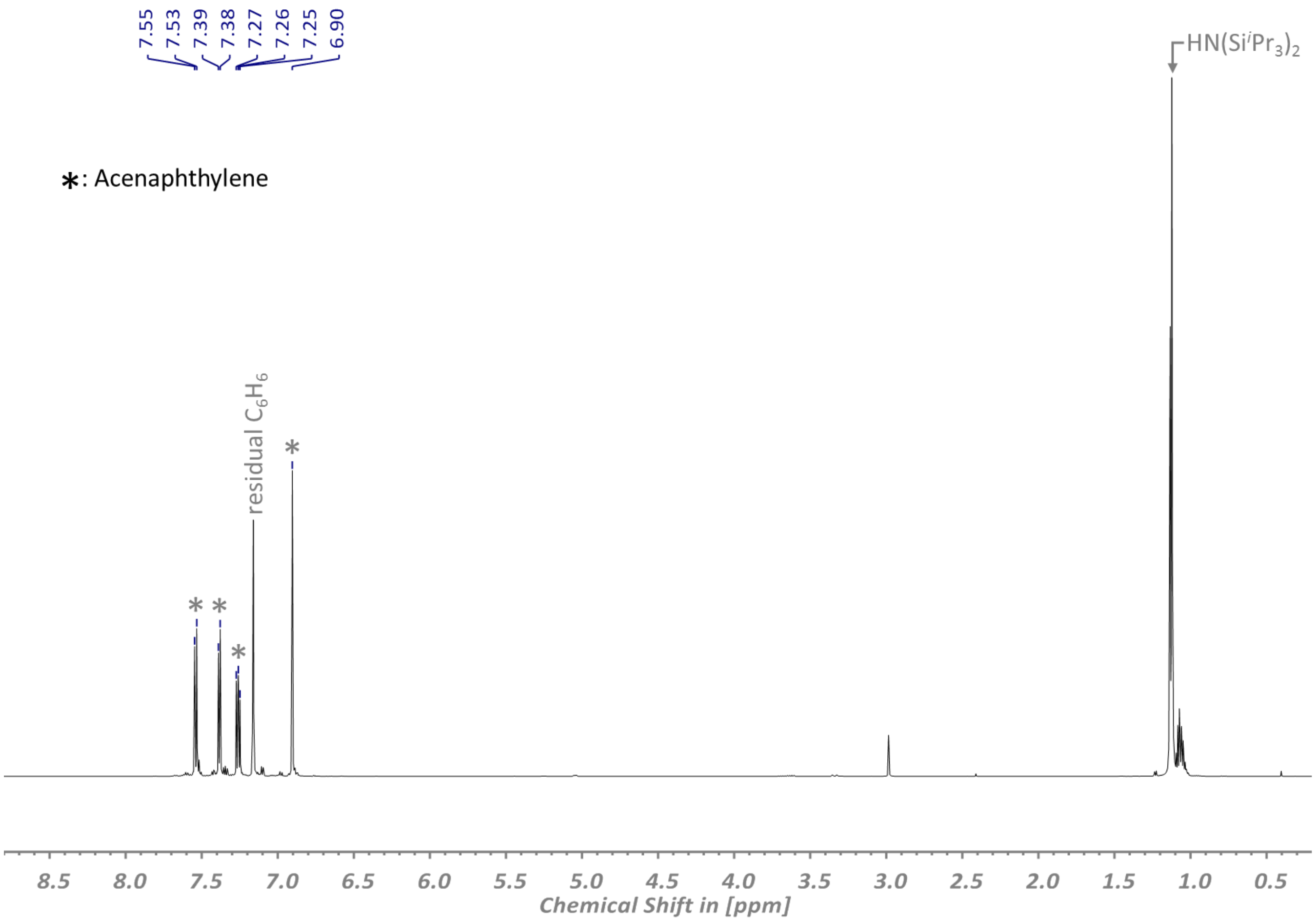

Figure S215. ^{1}H NMR spectrum (600 MHz, C_6D_6, 25 °C) after the reaction (24 h) of acenaphthylene (∗) with Ba[N(Si^{i}Pr$_3$)$_2$]$_2$ (**1-Ba**) (10 mol%) and H_2 (12 bar) showing no formation of acenaphthene (Table S4, entry 19).

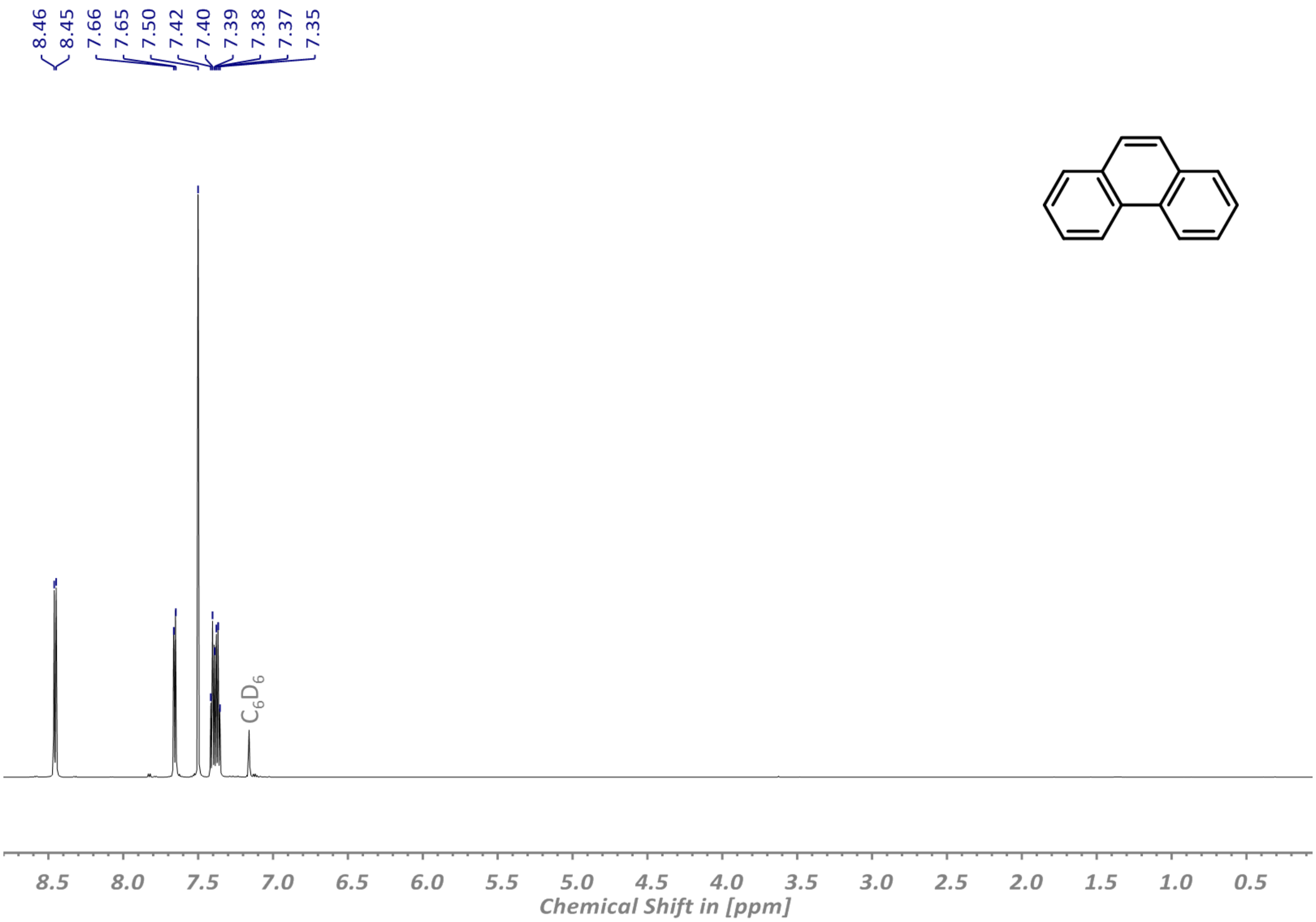

Figure S216. ^{1}H NMR spectrum (600 MHz, C_6D_6, 25 °C) of phenanthrene.

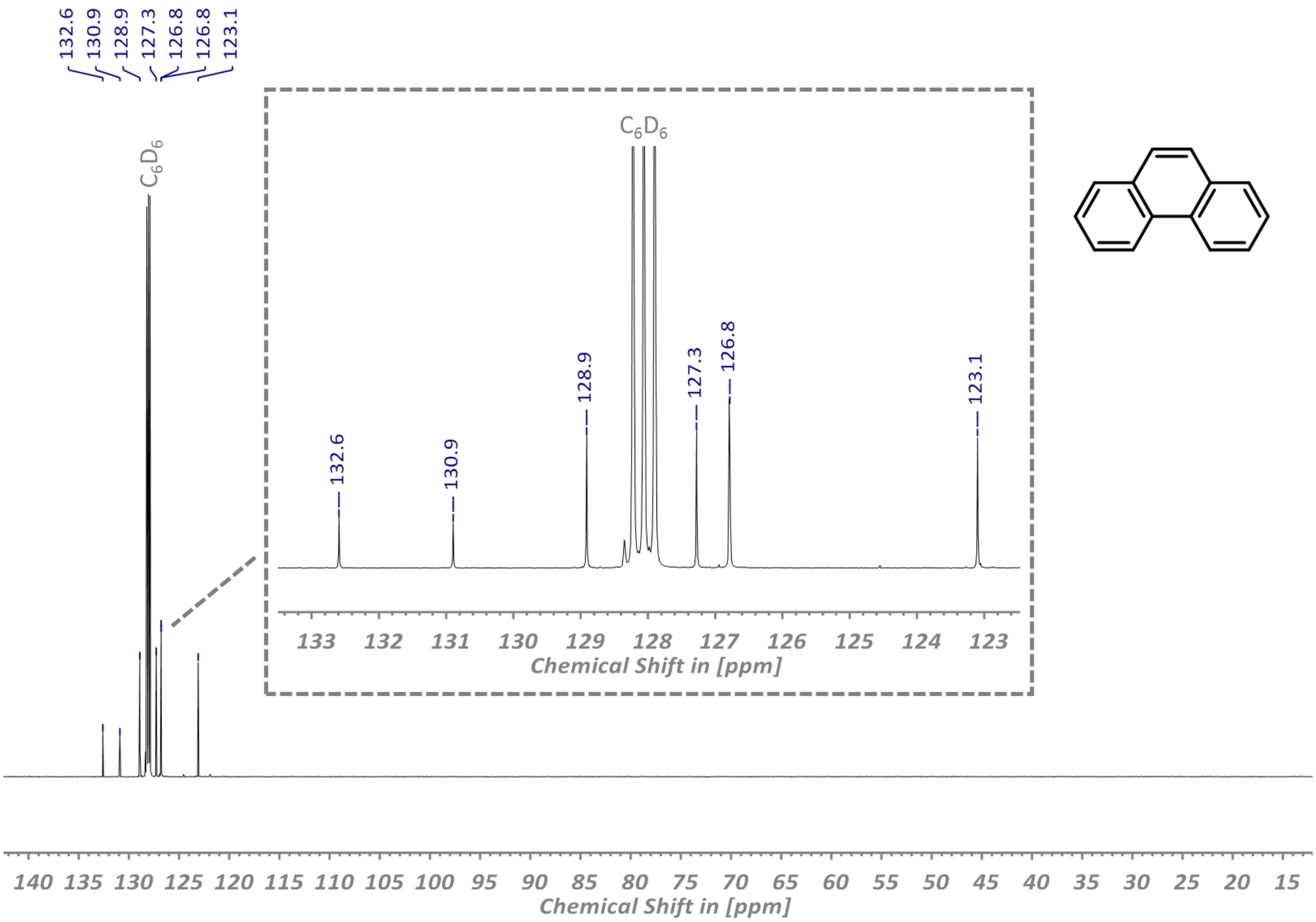

Figure S217. $^{13}C\{^{1}H\}$ NMR spectrum (151 MHz, C_6D_6, 25 °C) of phenanthrene.

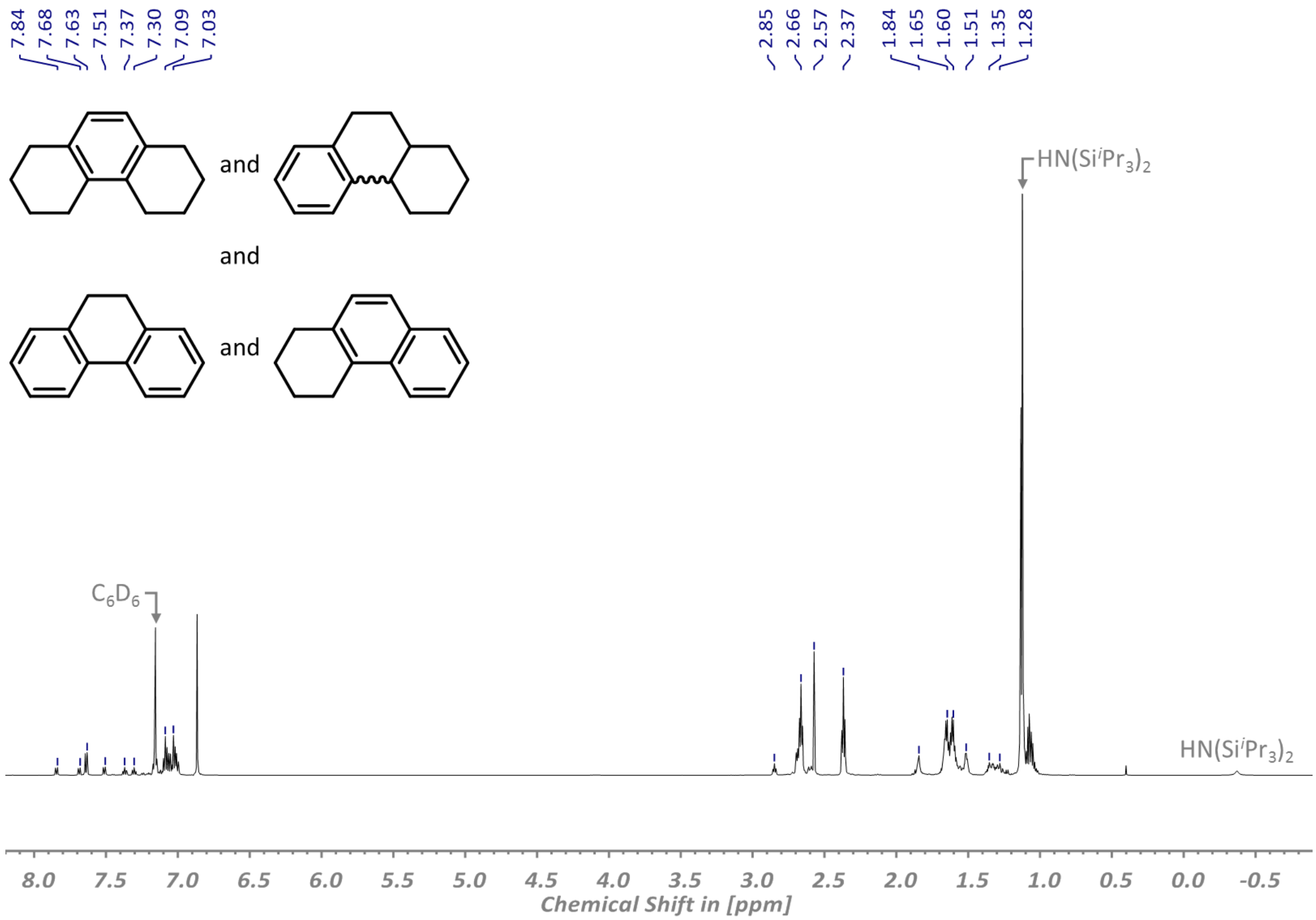

Figure S218. ^{1}H NMR spectrum (600 MHz, C_6D_6, 25 °C) after the catalytic hydrogenation (24 h) of phenanthrene with Ba[N(Si^{i}Pr$_3$)$_2$]$_2$ (**1-Ba**) (10 mol%) and H_2 (12 bar) showing the formation of various hydrogenated products (Table S4, entry 20).

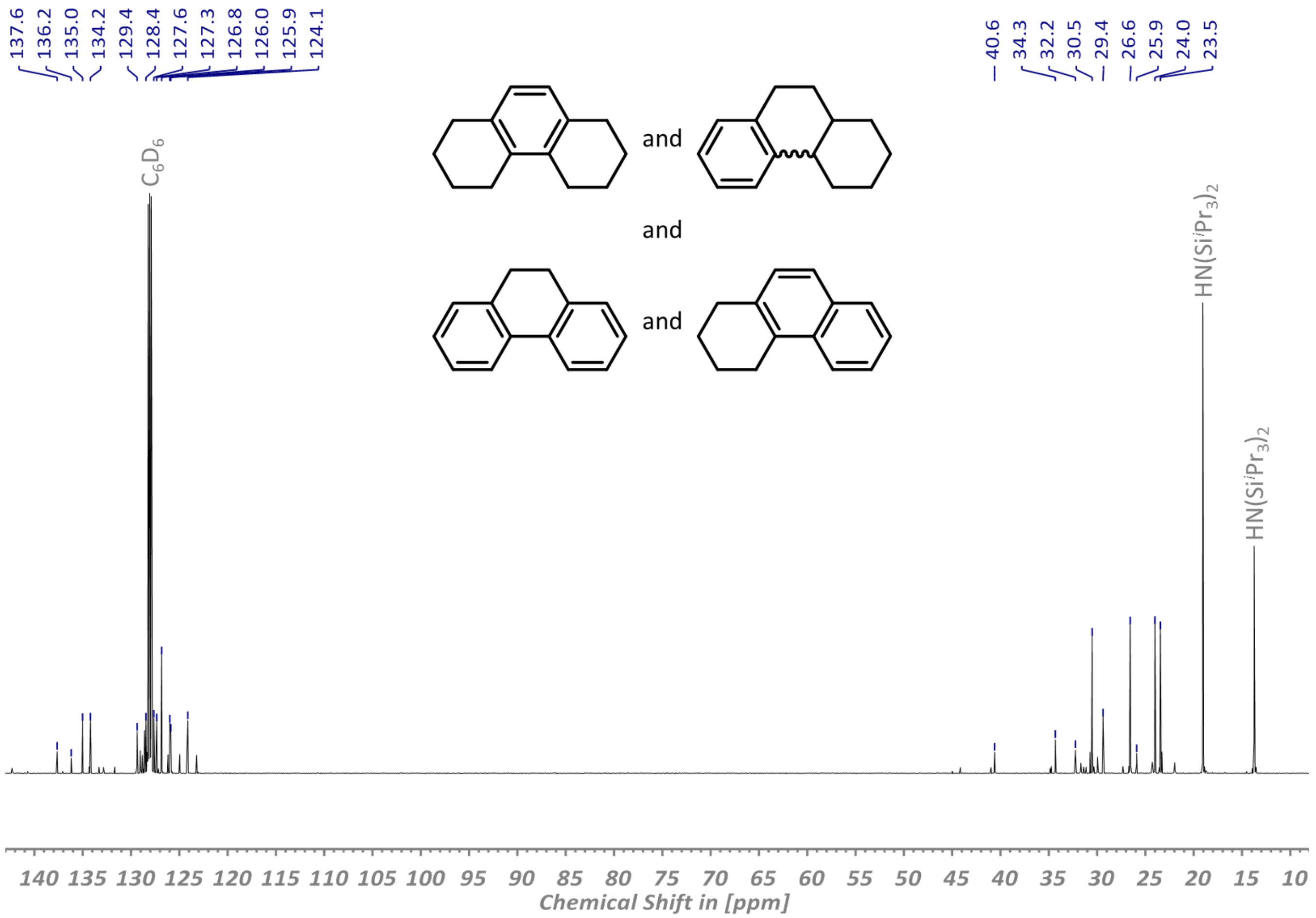

Figure S219. ^{1}H NMR spectrum (151 MHz, C_6D_6, 25 °C) after the catalytic hydrogenation (24 h) of phenanthrene with $Ba[N(Si^iPr_3)_2]_2$ (**1-Ba**) (10 mol%) and H_2 (12 bar) showing the formation of various hydrogenated products (Table S4, entry 20).

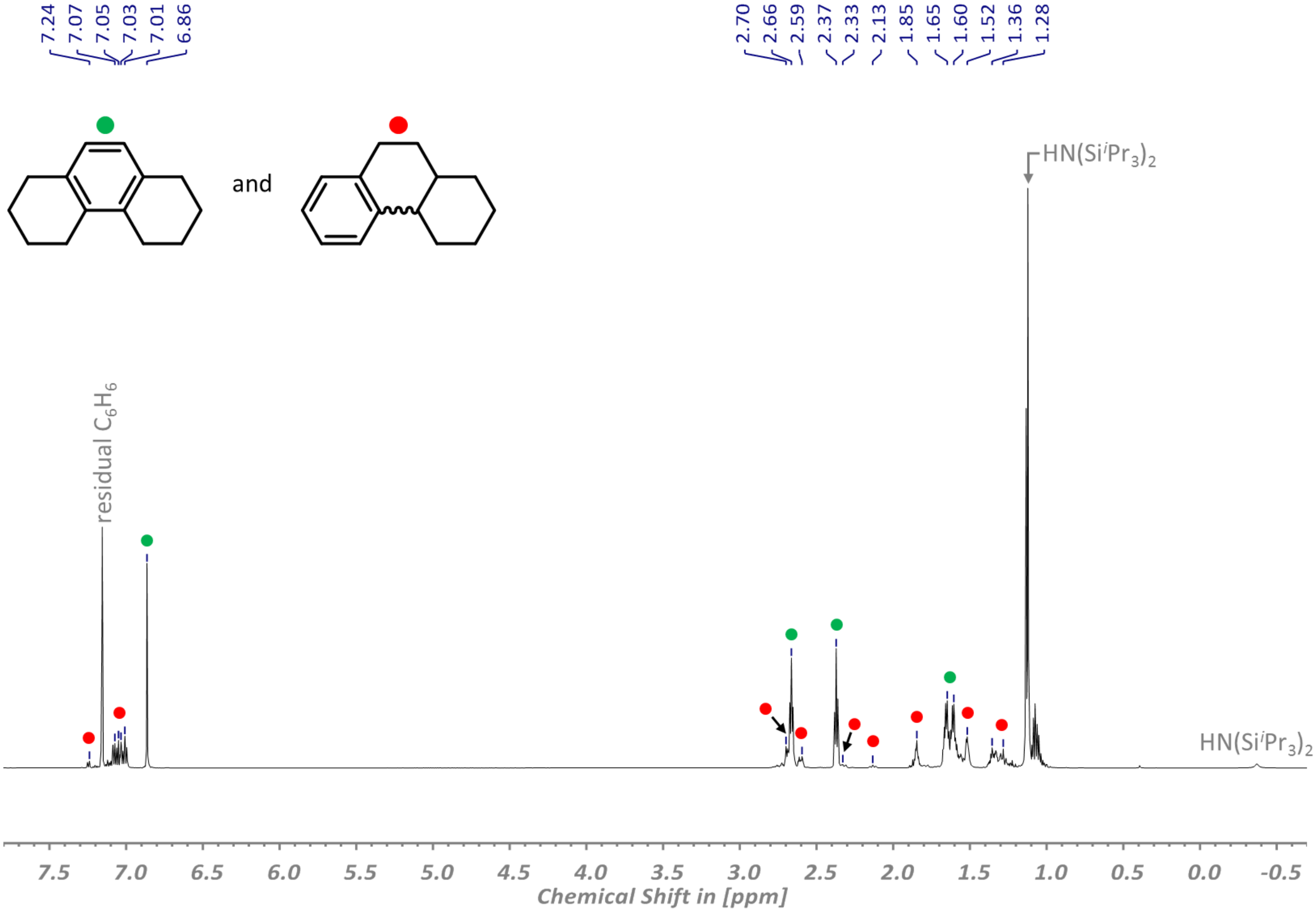

Figure S220. ^{1}H NMR spectrum (151 MHz, C_6D_6, 25 °C) after the catalytic hydrogenation (48 h) of phenanthrene with Ba[N(Si^{i}Pr$_3$)$_2$]$_2$ (**1-Ba**) (10 mol%) and H_2 (20 bar) showing the formation of 1,2,3,4,5,6,7,8-octahydrophenanthrene (●) and 1,2,3,4,4a,9,10,10a-octahydrophenanthrene (●). *Note:* The latter is obtained as a mixture of two diastereomers (*cis*/*trans*) following hydrogenation (Table S4, entry 21).

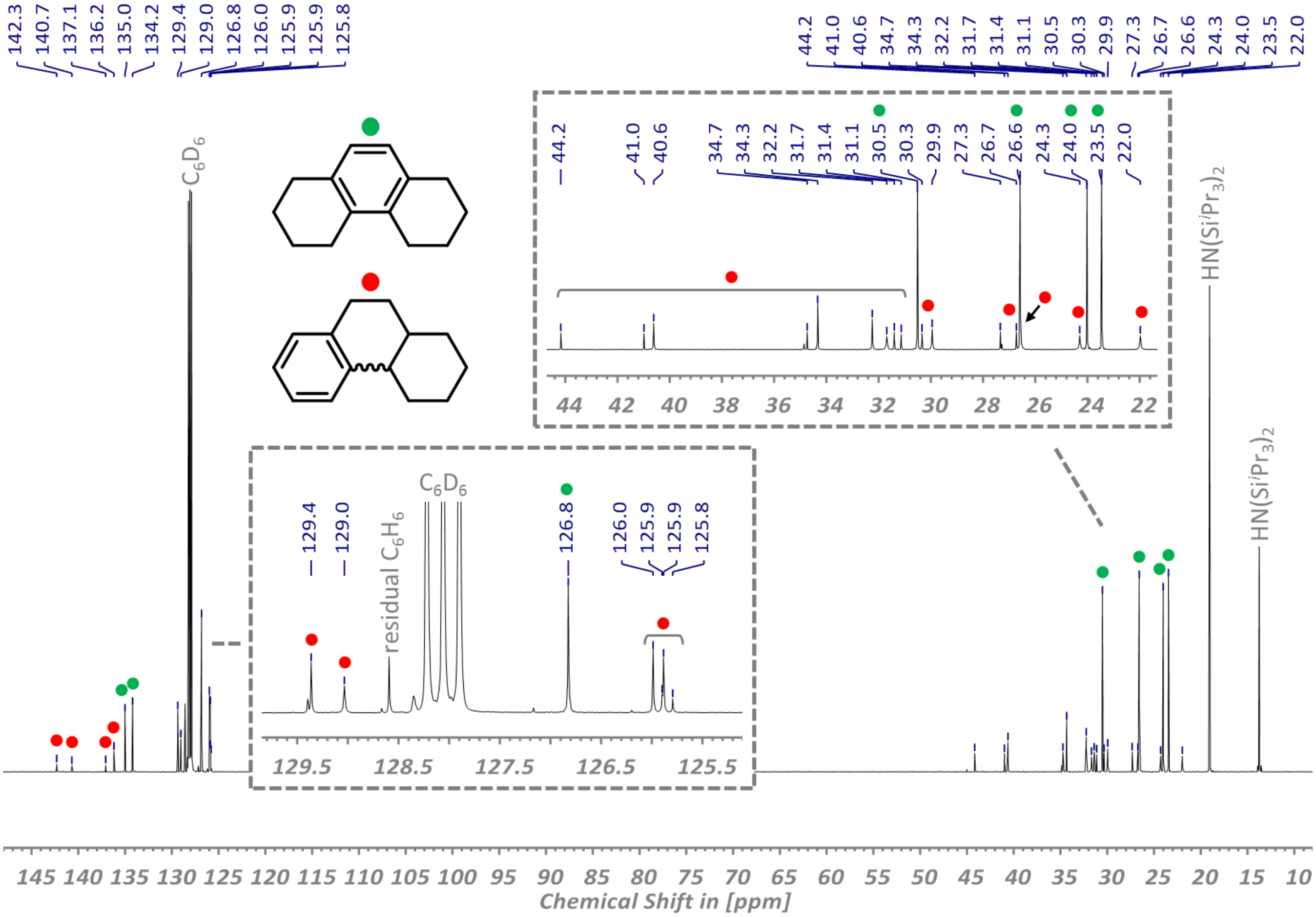

Figure S221. $^{13}C\{^1H\}$ NMR spectrum (151 MHz, C_6D_6, 25 °C) after the catalytic hydrogenation (48 h) of phenanthrene with $Ba[N(Si^iPr_3)_2]_2$ (**1-Ba**) (10 mol%) and H_2 (20 bar) showing the formation of 1,2,3,4,5,6,7,8-octahydrophenanthrene (●) and 1,2,3,4,4a,9,10,10a-octahydrophenanthrene (●). *Note:* The latter is obtained as a mixture of two diastereomers (*cis*/*trans*) following hydrogenation. The spectroscopic data are in good agreement with those reported in the literature[S23][S27,28] (Table S4, entry 21).

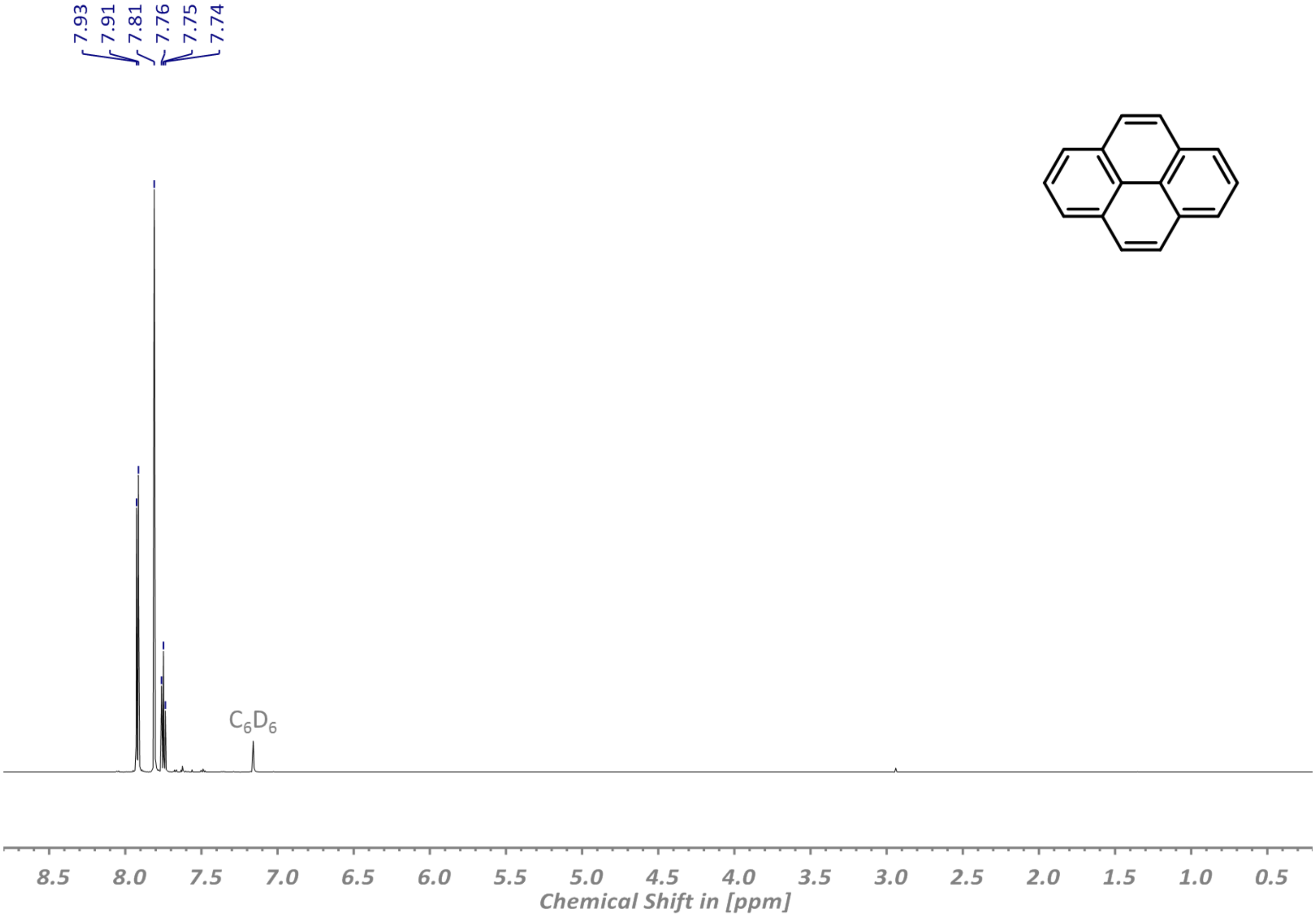

Figure S222. ^{1}H NMR spectrum (600 MHz, C_6D_6, 25 °C) of pyrene.

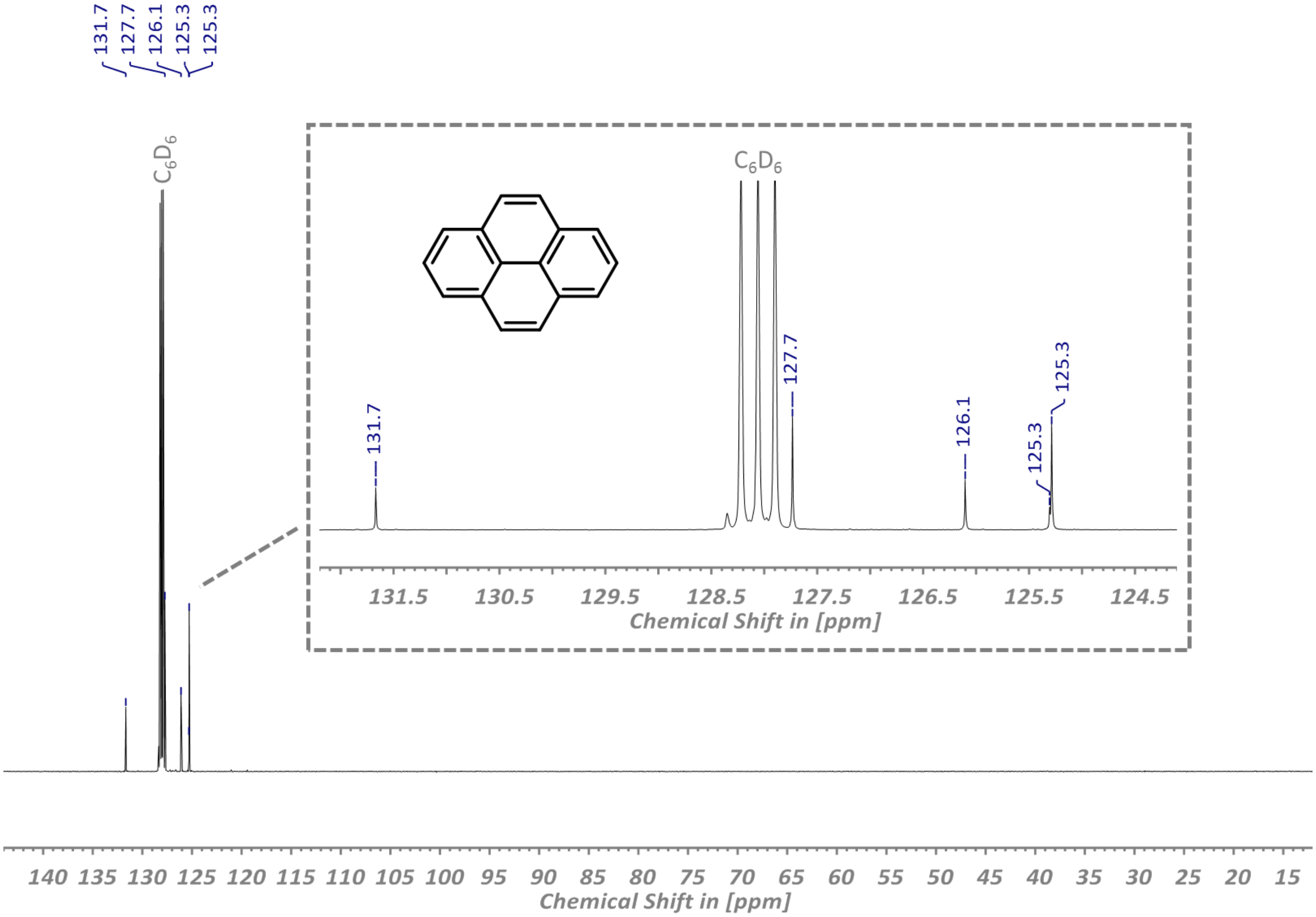

Figure S223. $^{13}C\{^1H\}$ NMR spectrum (151 MHz, C_6D_6, 25 °C) of pyrene.

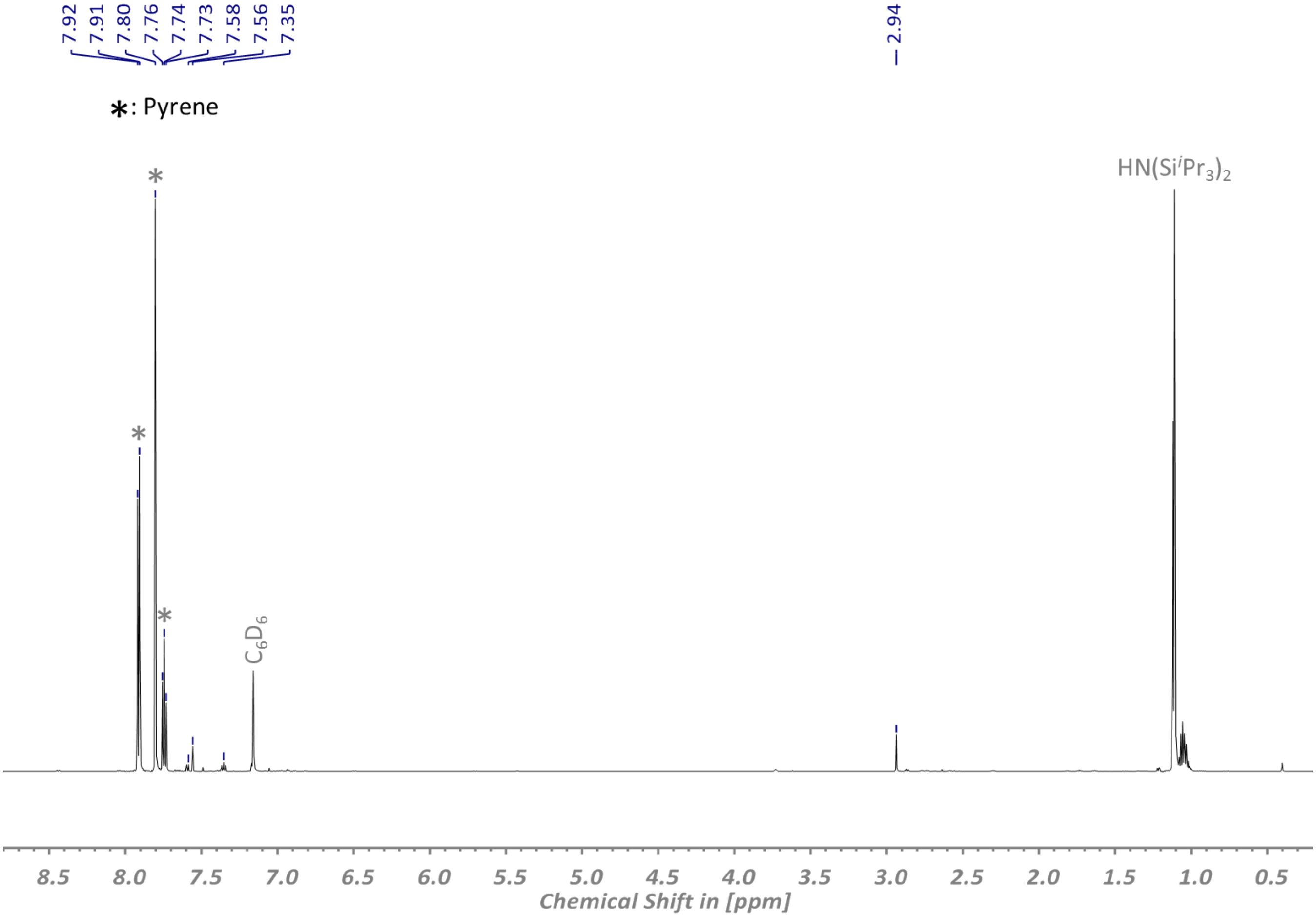

Figure S224. ^{1}H NMR spectrum (600 MHz, C_6D_6, 25 °C) after the catalytic hydrogenation (24 h) of pyrene (∗) with $Ba[N(Si^iPr_3)_2]_2$ (**1-Ba**) (10 mol%) and H_2 (12 bar) showing the formation of 4,5-dihydropyrene in a substoichiometric amount (Table S4, entry 22).

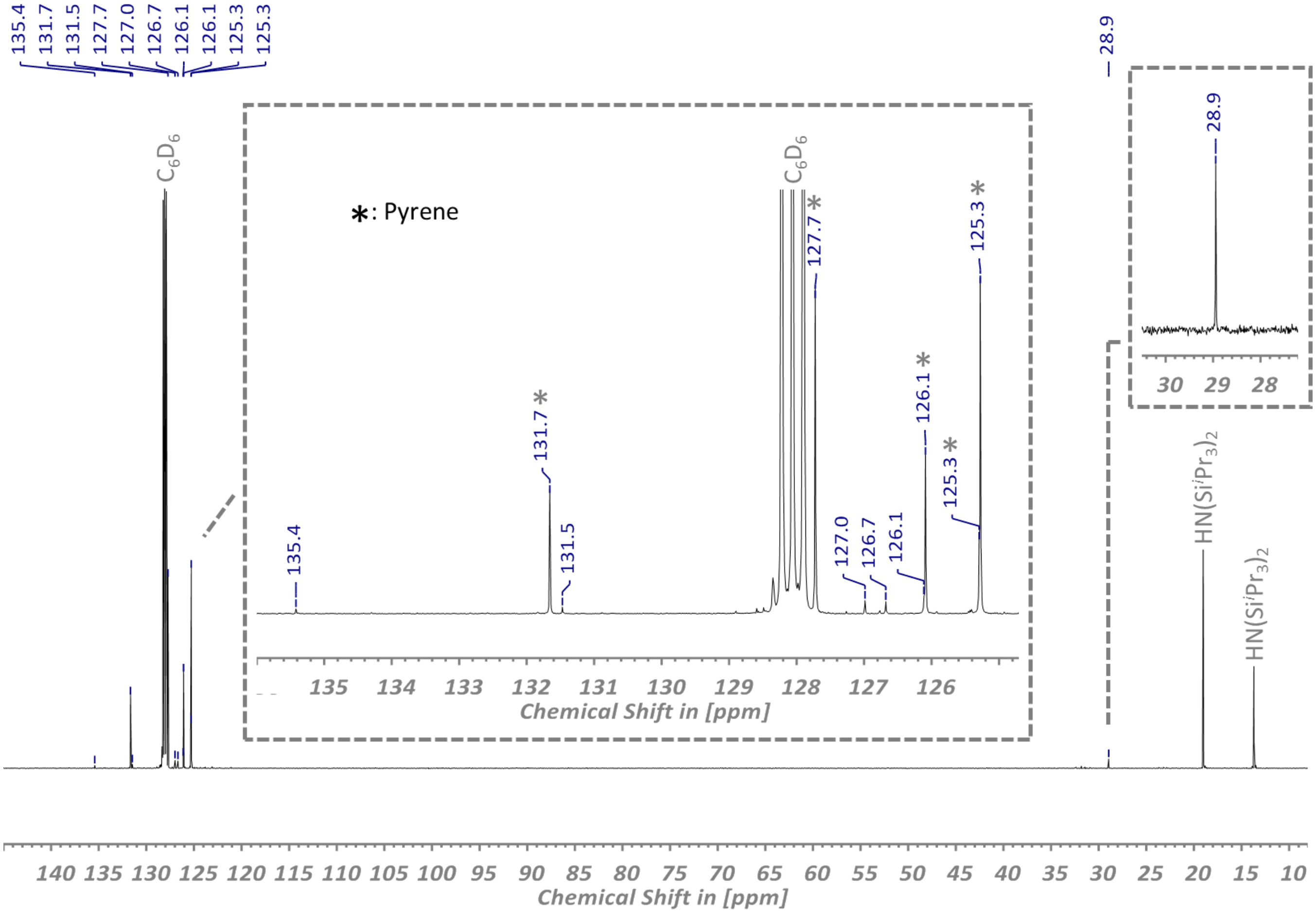

Figure S225. $^{13}C\{^{1}H\}$ NMR spectrum (151 MHz, C_6D_6, 25 °C) after the catalytic hydrogenation (24 h) of pyrene (∗) with $Ba[N(Si^{i}Pr_3)_2]_2$ (**1-Ba**) (10 mol%) and H_2 (12 bar) showing the formation of 4,5-dihydropyrene in a substoichiometric amount. *Note:* The minimal yield (6%) of 4,5-dihydropyrene in combination with superimposition of resonance signals precluded a meaningful assignment of the $^{13}C\{^{1}H\}$ NMR spectrum (Table S4, entry 22).

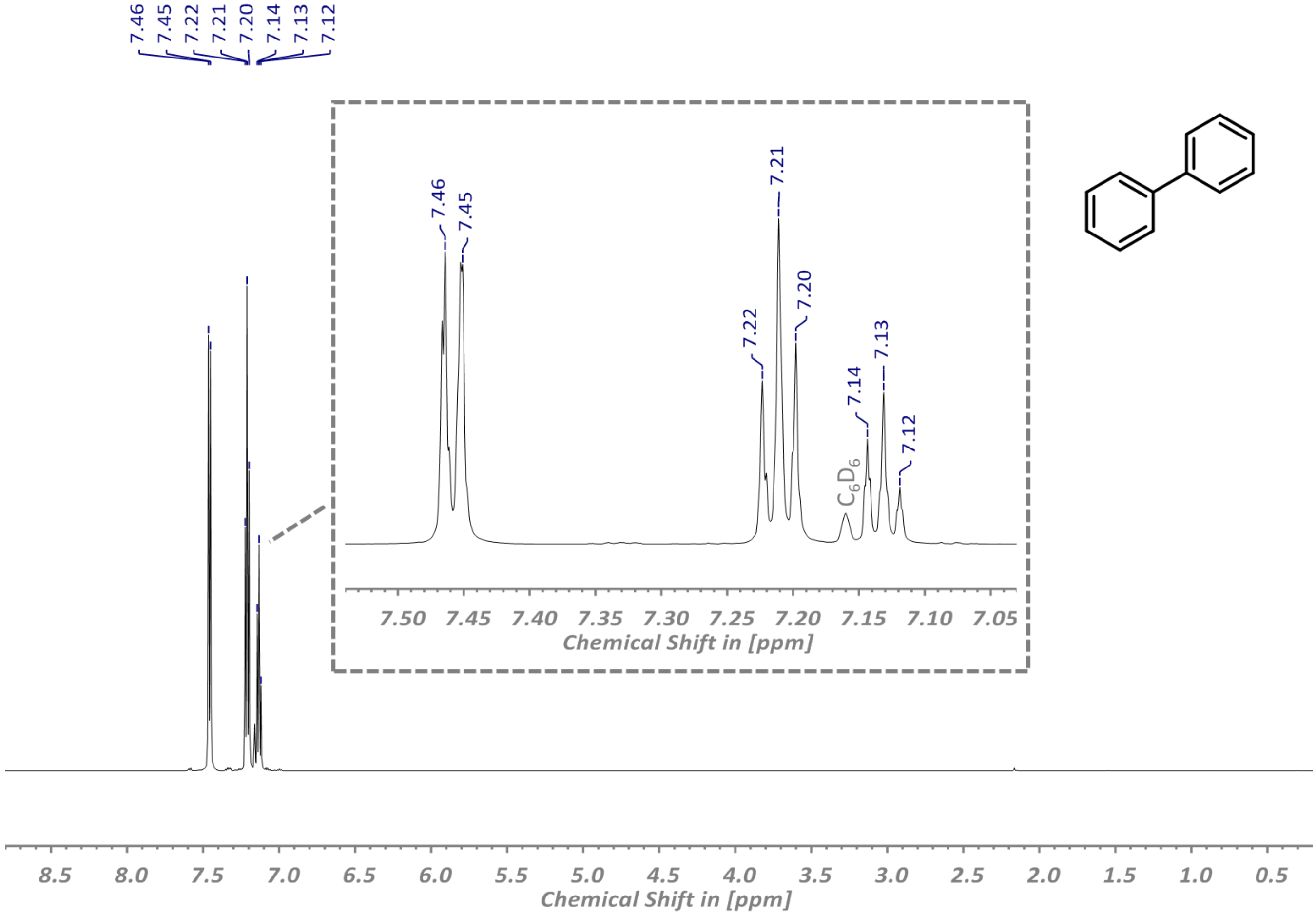

Figure S226. ^{1}H NMR spectrum (600 MHz, C_6D_6, 25 °C) of biphenyl.

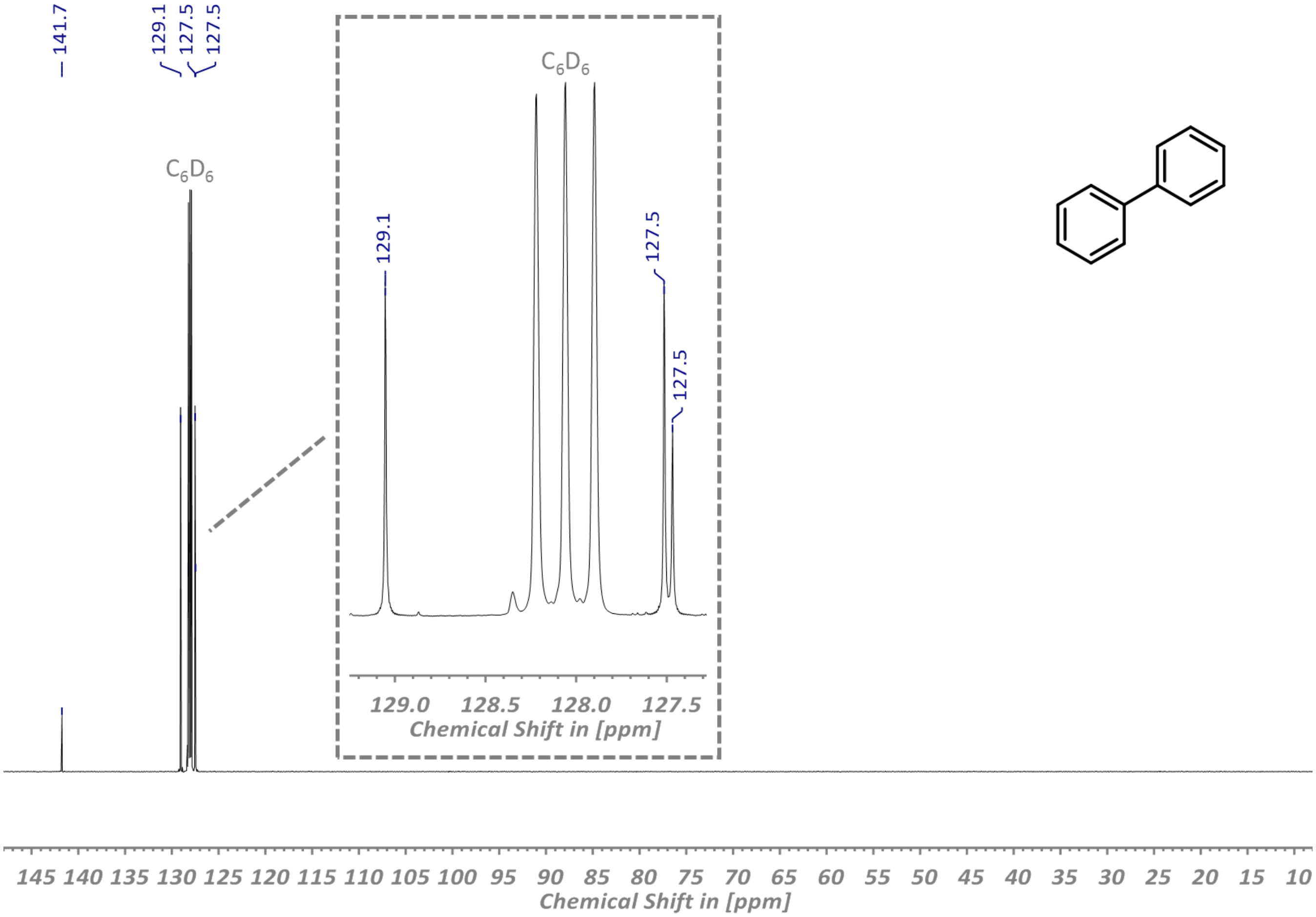

Figure S227. $^{13}C\{^{1}H\}$ NMR spectrum (151 MHz, C_6D_6, 25 °C) of biphenyl.

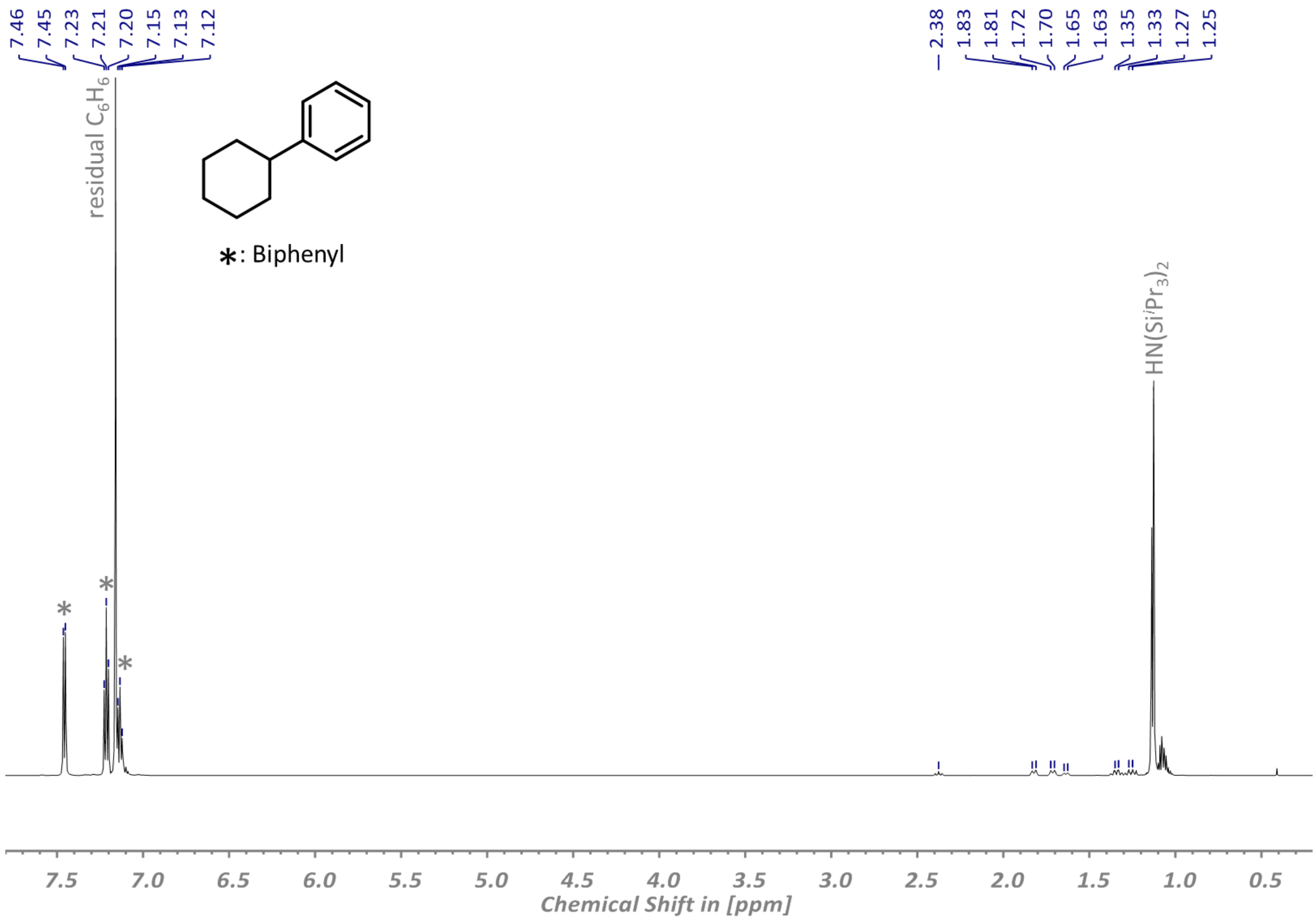

Figure S228. ^{1}H NMR spectrum (600 MHz, C_6D_6, 25 °C) after catalytic hydrogenation (24 h) of biphenyl (∗) with Ca[N(Si^{i}Pr$_3$)$_2$]$_2$ (**1-Ca**) (10 mol%) and H_2 (12 bar) at 120 °C showing the formation of phenylcyclohexane (Table S4, entry 23).

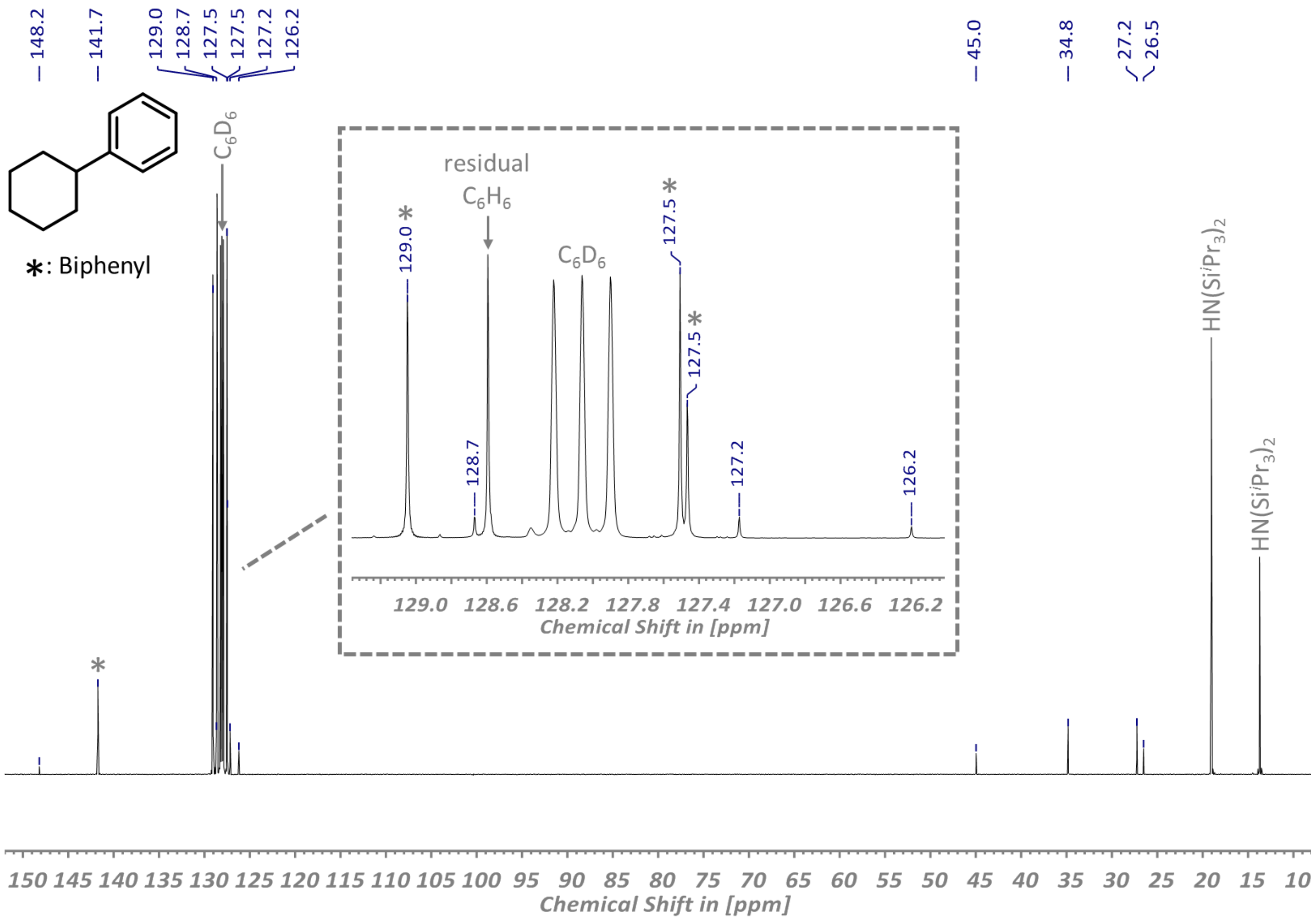

Figure S229. $^{13}C\{^{1}H\}$ NMR spectrum (151 MHz, C_6D_6, 25 °C) after catalytic hydrogenation (24 h) of biphenyl (∗) with Ca[N(Si^{i}Pr$_3$)$_2$]$_2$ (**1-Ca**) (10 mol%) and H_2 (12 bar) at 120 °C showing the formation of phenylcyclohexane (Table S4, entry 23).

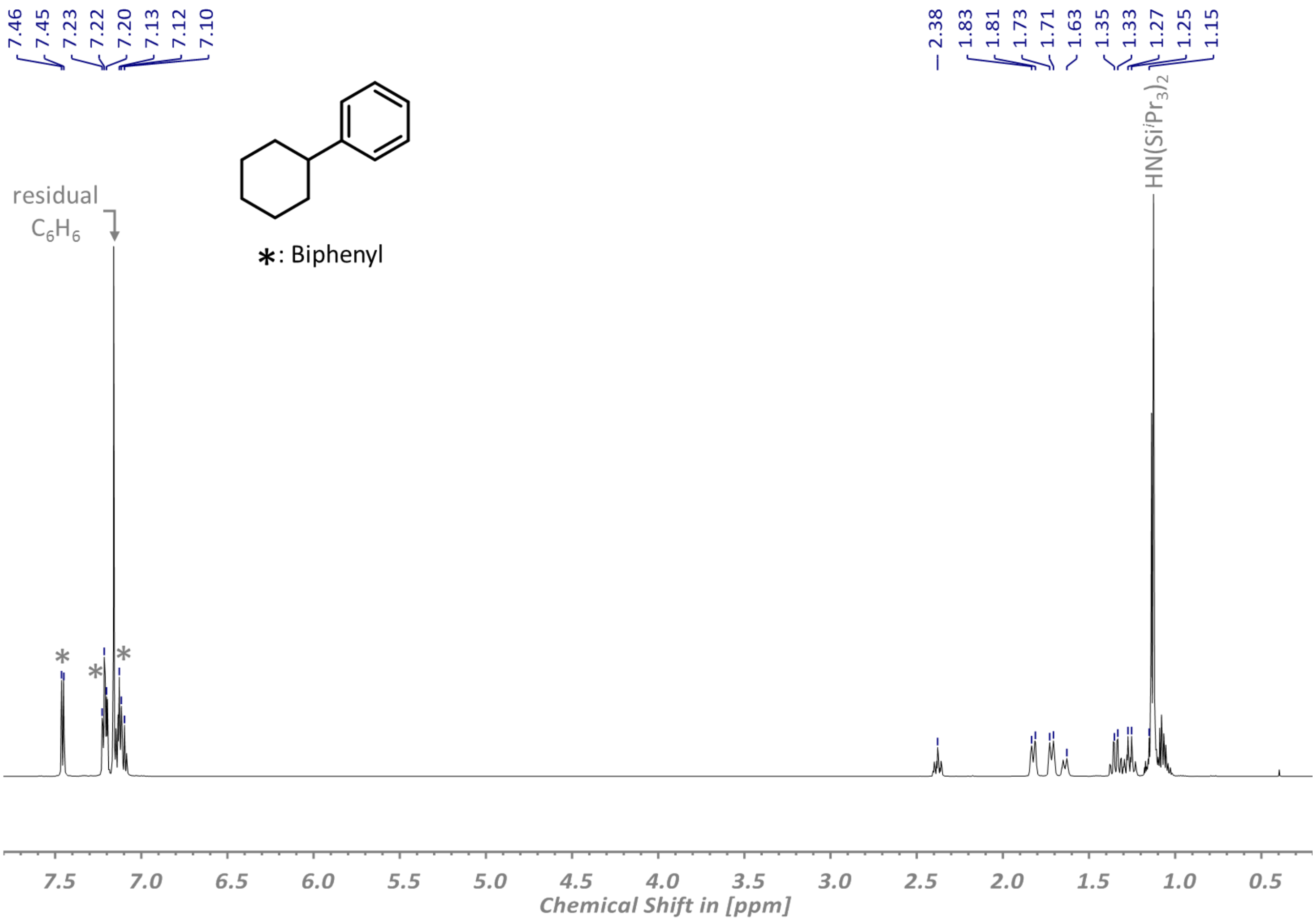

Figure S230. ^{1}H NMR spectrum (600 MHz, C_6D_6, 25 °C) after catalytic hydrogenation (24 h) of biphenyl (∗) with $Sr[N(Si^{i}Pr_3)_2]_2$ (**1-Sr**) (10 mol%) and H_2 (12 bar) at 120 °C showing the formation of phenylcyclohexane (Table S4, entry 24).

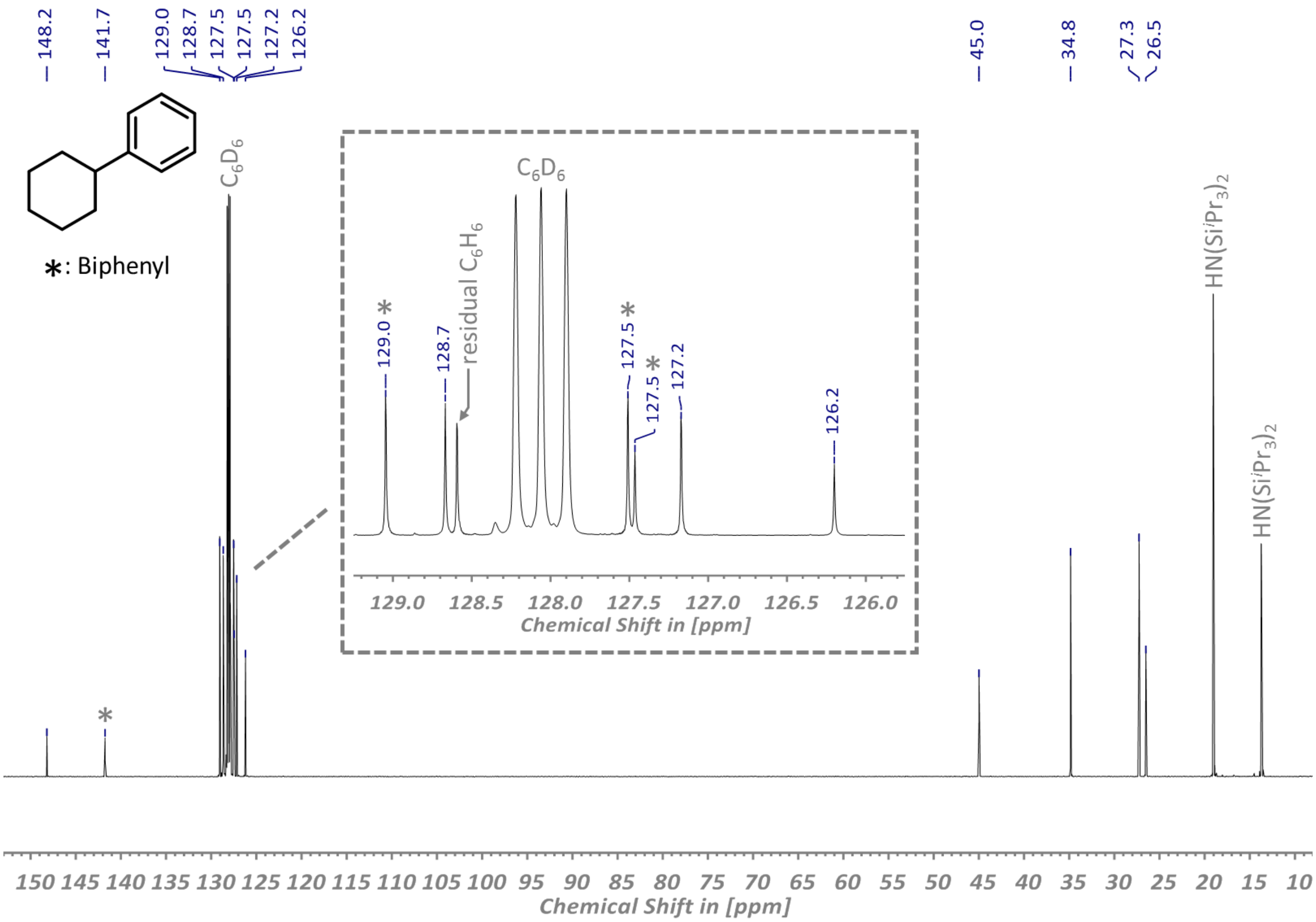

Figure S231. $^{13}C\{^1H\}$ NMR spectrum (151 MHz, C_6D_6, 25 °C) after catalytic hydrogenation (24 h) of biphenyl (∗) with $Sr[N(Si^iPr_3)_2]_2$ (**1-Sr**) (10 mol%) and H_2 (12 bar) at 120 °C showing the formation of phenylcyclohexane (Table S4, entry 24).

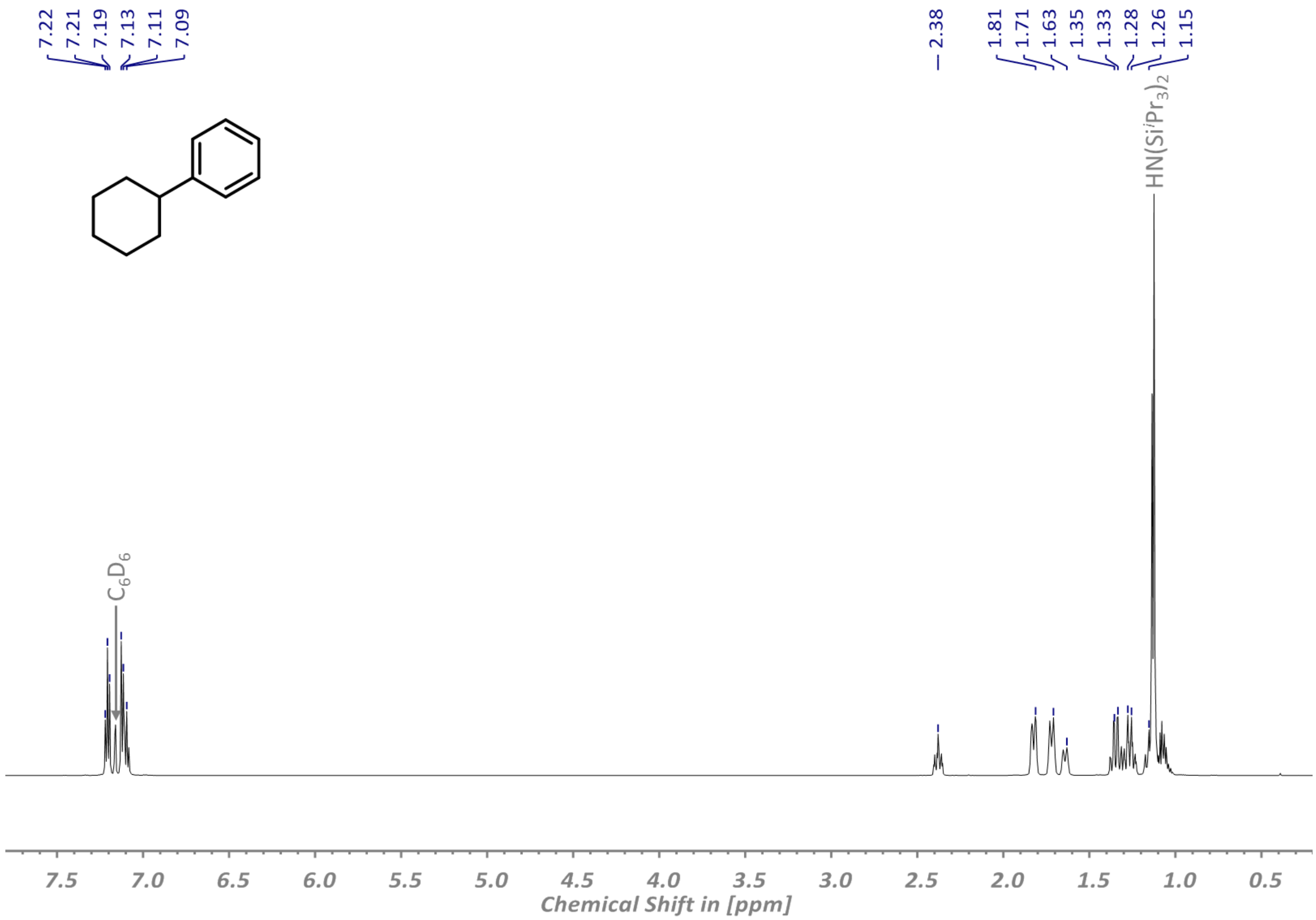

Figure S232. ^{1}H NMR spectrum (600 MHz, C_6D_6, 25 °C) of phenylcyclohexane after catalytic hydrogenation (10 h) of biphenyl with Ba[N(Si^{i}Pr$_3$)$_2$]$_2$ (**1-Ba**) (10 mol%) and H_2 (12 bar) at 120 °C (Table S3, entry 25).

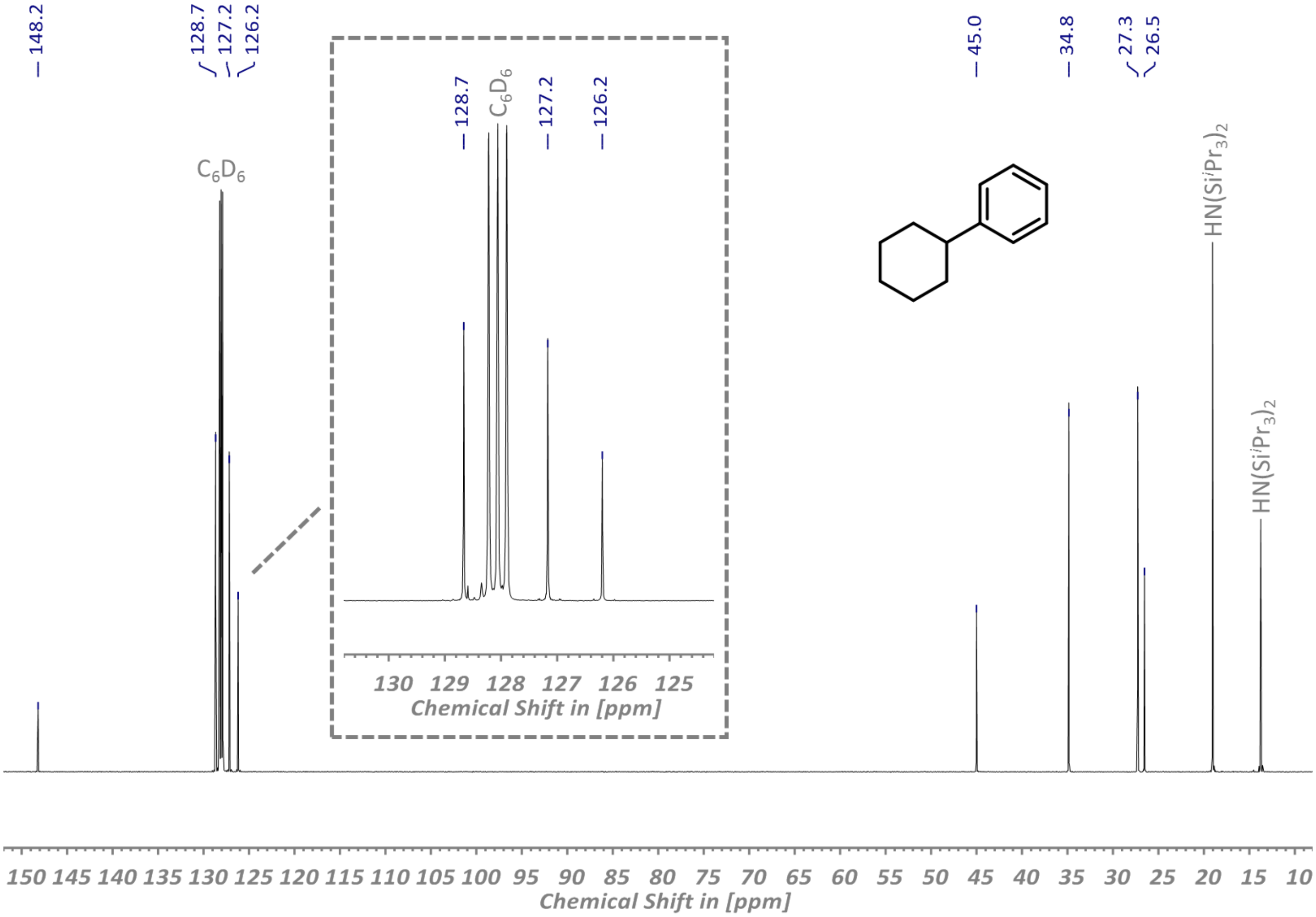

Figure S233. $^{13}C\{^1H\}$ NMR spectrum (151 MHz, C_6D_6, 27 °C) of phenylcyclohexane after catalytic hydrogenation (10 h) of biphenyl with $Ba[N(Si^iPr_3)_2]_2$ (**1-Ba**) (10 mol%) and H_2 (12 bar) at 120 °C. *Note:* The spectroscopic data matched those previously reported for this compound[S21] (Table S3, entry 25).

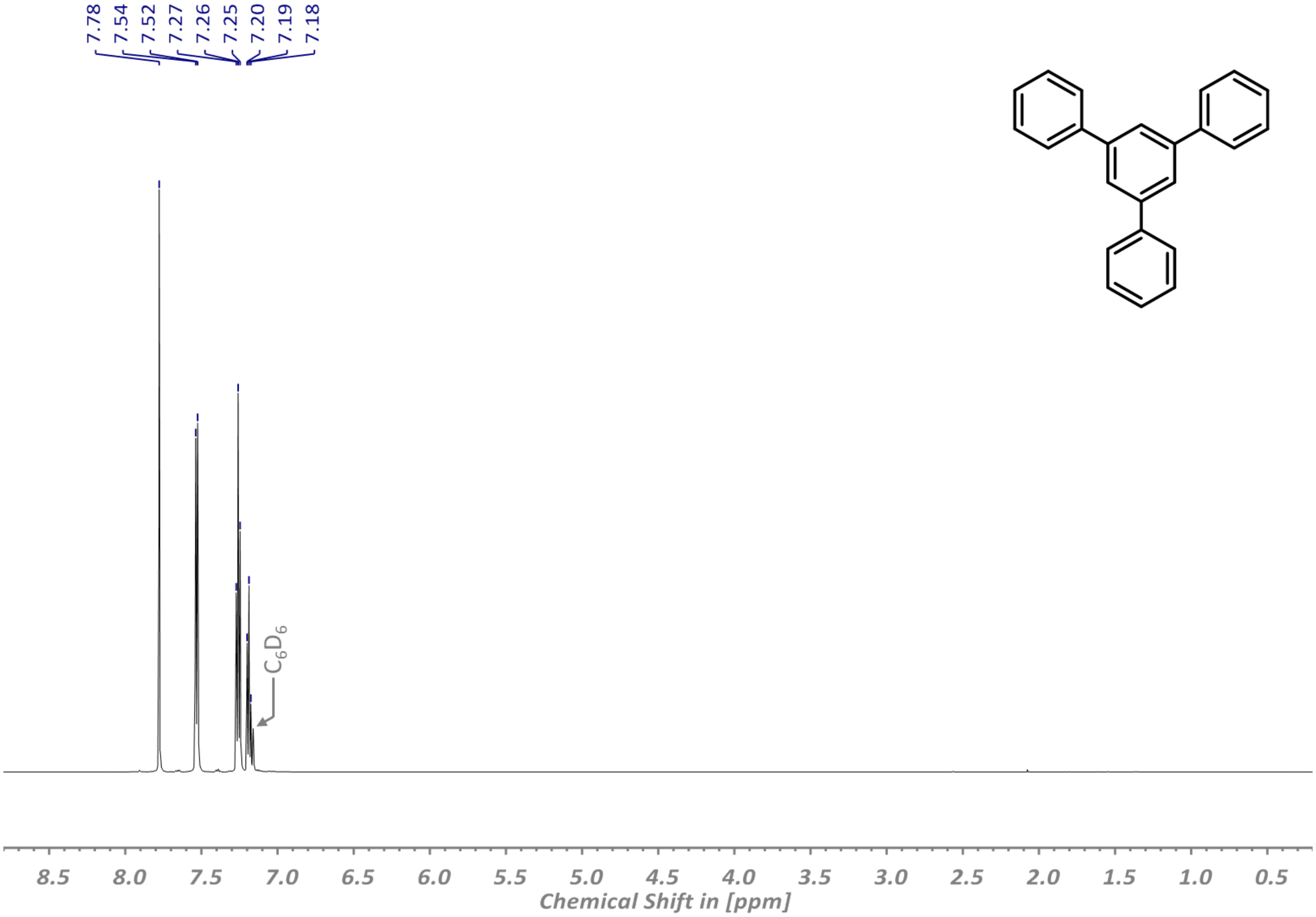

Figure S234. ^{1}H NMR spectrum (600 MHz, C_6D_6, 25 °C) of 1,3,5-triphenylbenzene.

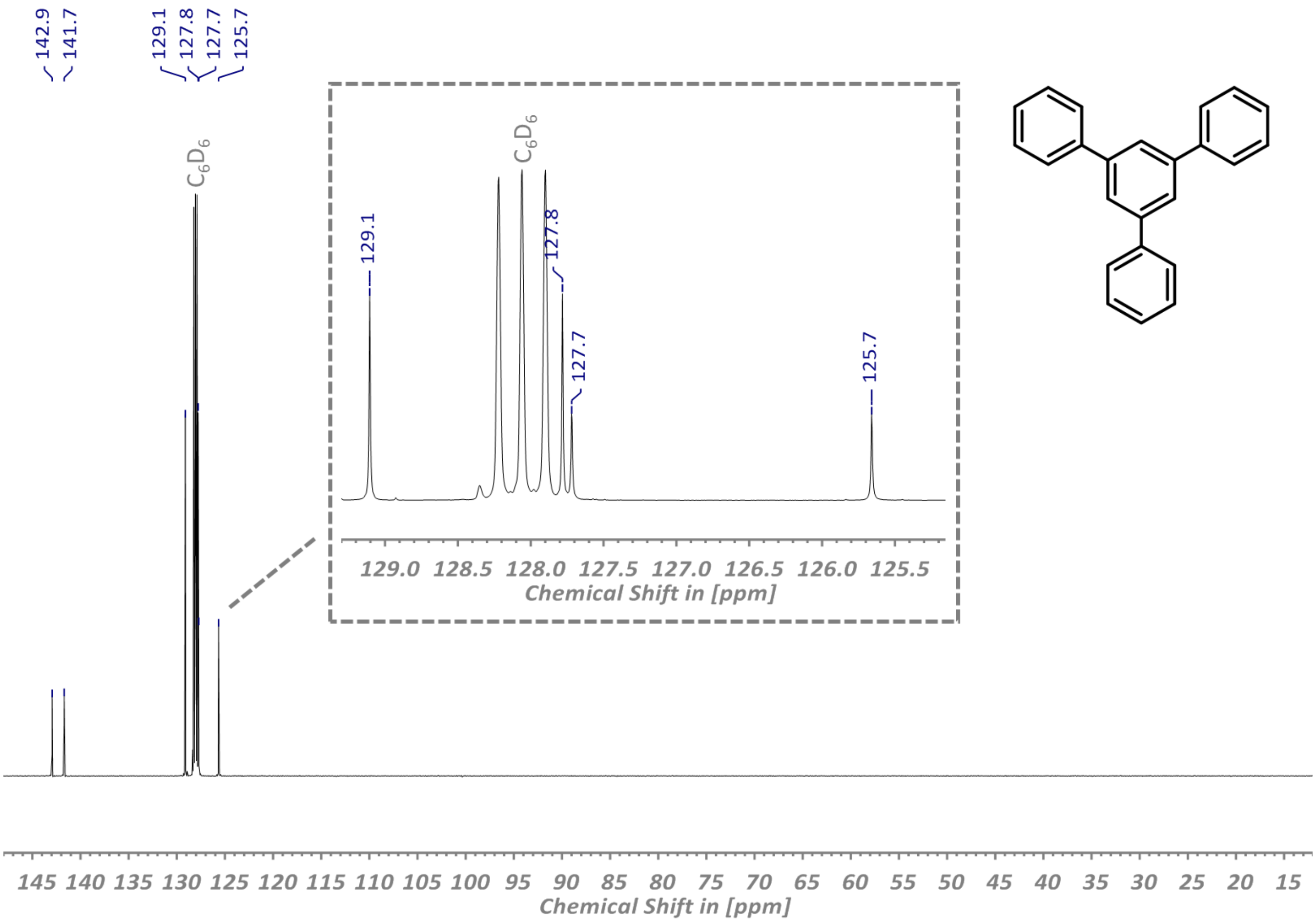

Figure S235. $^{13}C\{^{1}H\}$ NMR spectrum (151 MHz, C_6D_6, 25 °C) of 1,3,5-triphenylbenzene.

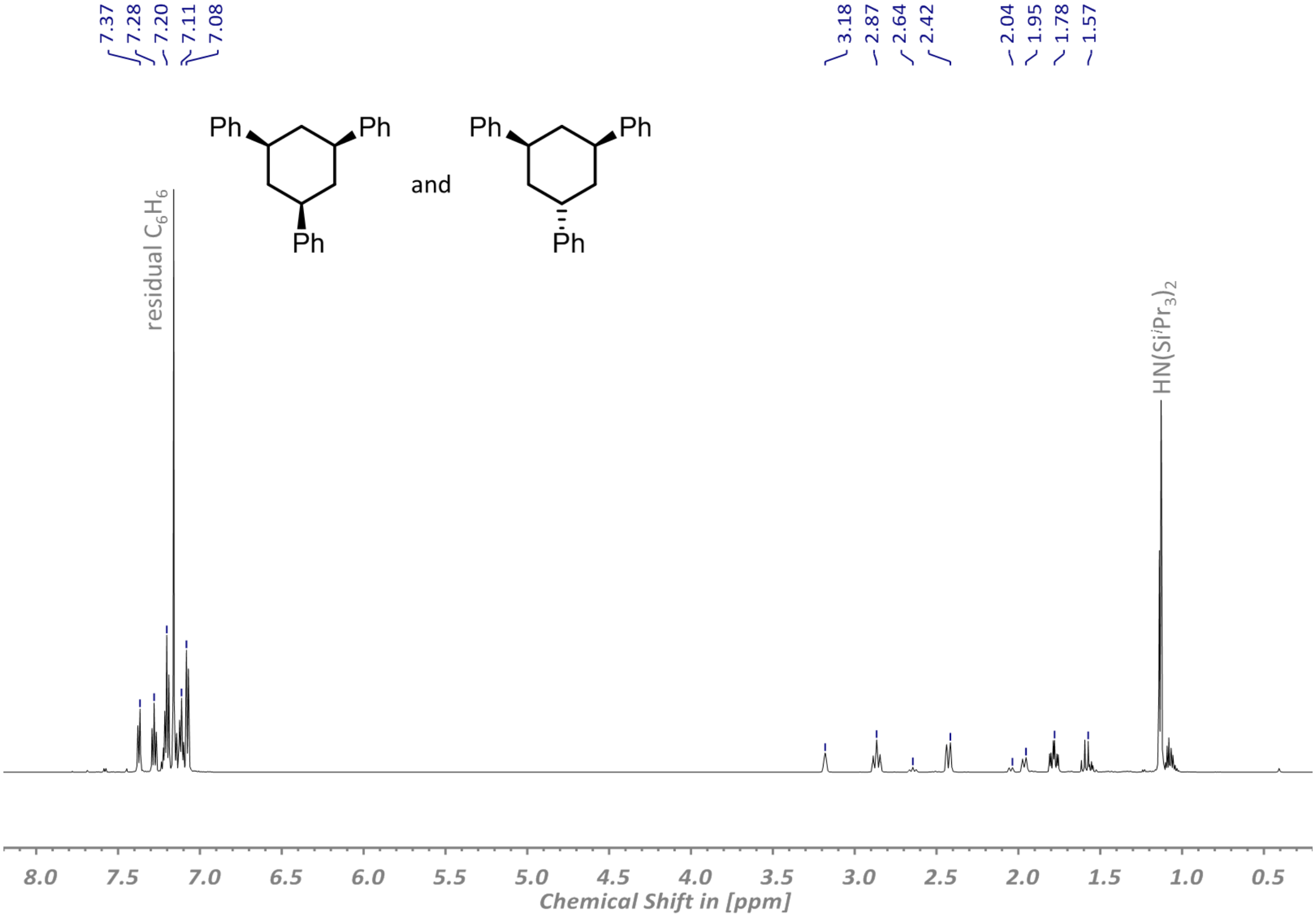

Figure S236. ^{1}H NMR spectrum (600 MHz, C_6D_6, 25 °C) of 1,3,5-triphenylcyclohexane after catalytic hydrogenation (24 h) of 1,3,5-triphenylbenzene with $Ba[N(Si^{i}Pr_3)_2]_2$ (**1-Ba**) (10 mol%) and H_2 (12 bar) at 120 °C. *Note:* 1,3,5-Triphenylcyclohexane is obtained as a mixture of two stereoisomers (1,3,5-*e*,*e*,*e*-$C_6H_9Ph_3$ and 1,3,5-*a*,*e*,*e*-$C_6H_9Ph_3$) following hydrogenation (Table S4, entry 26).

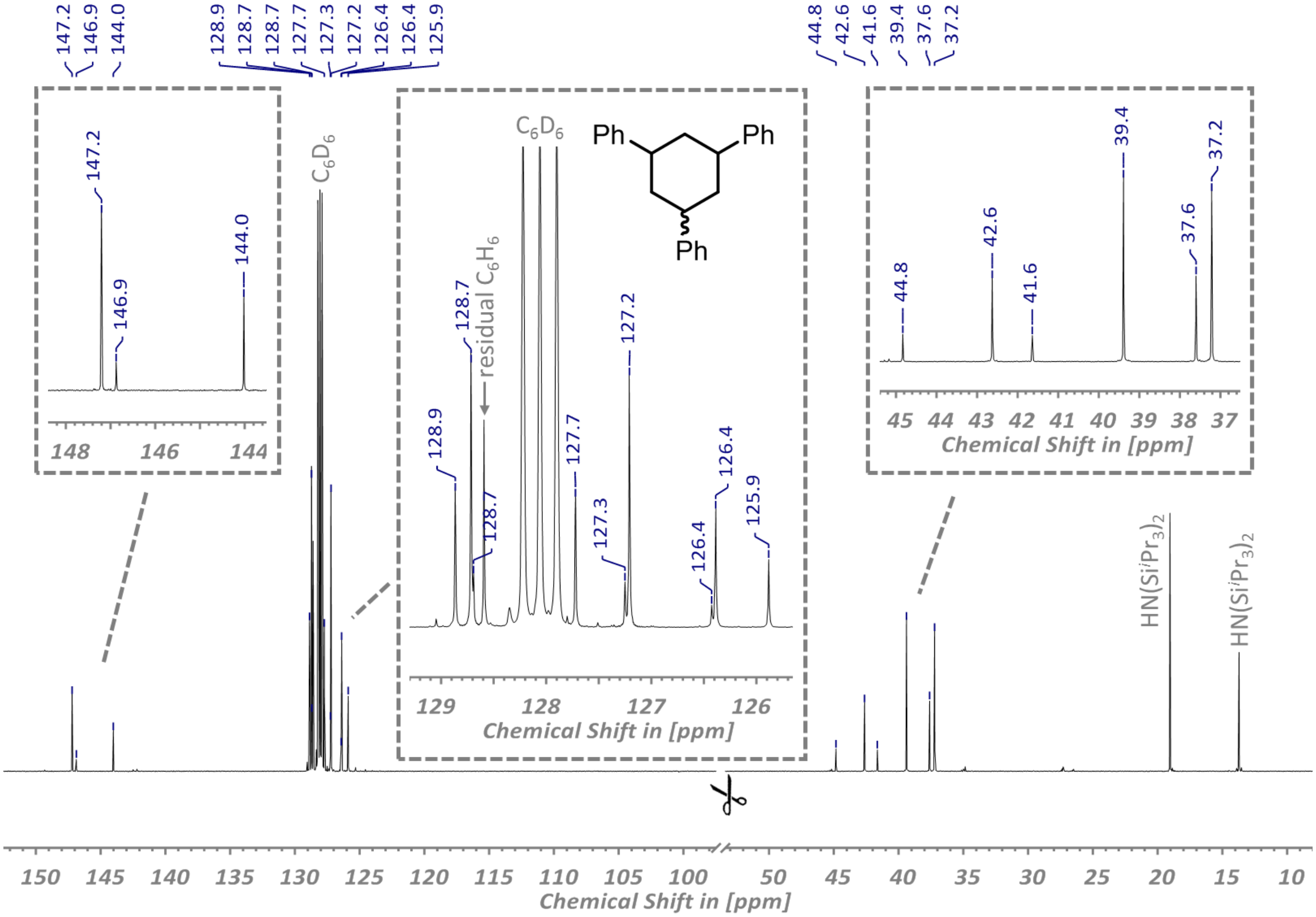

Figure S237. $^{13}C\{^{1}H\}$ NMR spectrum (151 MHz, C_6D_6, 25 °C) of 1,3,5-triphenylcyclohexane after catalytic hydrogenation (24 h) of 1,3,5-triphenylbenzene with $Ba[N(Si^iPr_3)_2]_2$ (**1-Ba**) (10 mol%) and H_2 (12 bar) at 120 °C. *Note:* 1,3,5-Triphenylcyclohexane is obtained as a mixture of two stereoisomers (1,3,5-*e*,*e*,*e*-$C_6H_9Ph_3$ and 1,3,5-*a*,*e*,*e*-$C_6H_9Ph_3$) following hydrogenation[S29] (Table S4, entry 26).

7 Catalytic Hydrogenation of Benzene

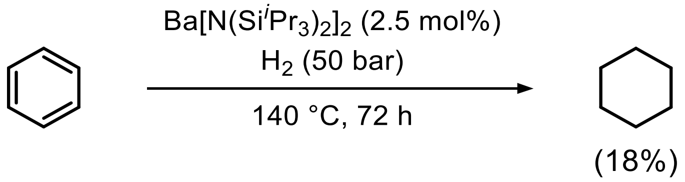

In an N_2-filled glovebox, an oven-dried miniature stainless steel autoclave (15 mL) containing a magnetic stir bar was charged with Ba[N(Si^{i}Pr$_3$)$_2$]$_2$ (180 mg, 0.23 mmol, 2.5 mol%) and benzene (800°µL, 699 mg, 8.95 mmol). The pressure vessel was sealed tightly, taken out of the glovebox and connected to a H_2 gas manifold. Following evacuation and refilling of the supply line with N_2 and then H_2 three times each, the autoclave was pressurized to 50 bars with H_2 gas, re-sealed and placed in a preheated aluminum heating block set to 140 °C. The reaction mixture was mechanically stirred under the H_2 pressure for 72 hours, at which point the reactor was left to cool to room temperature and carefully vented in a fume hood. The barium decomposition products were removed by filtration through a glass microfiber filter in a Pasteur pipette. An aliquot of the filtered reaction mixture (100 µL) was diluted with $CDCl_3$ (500 µL) and analyzed by NMR spectroscopy without additional purification. Quantitative $^{13}C\{^1H\}$ NMR analysis established 18% conversion of benzene to cyclohexane.

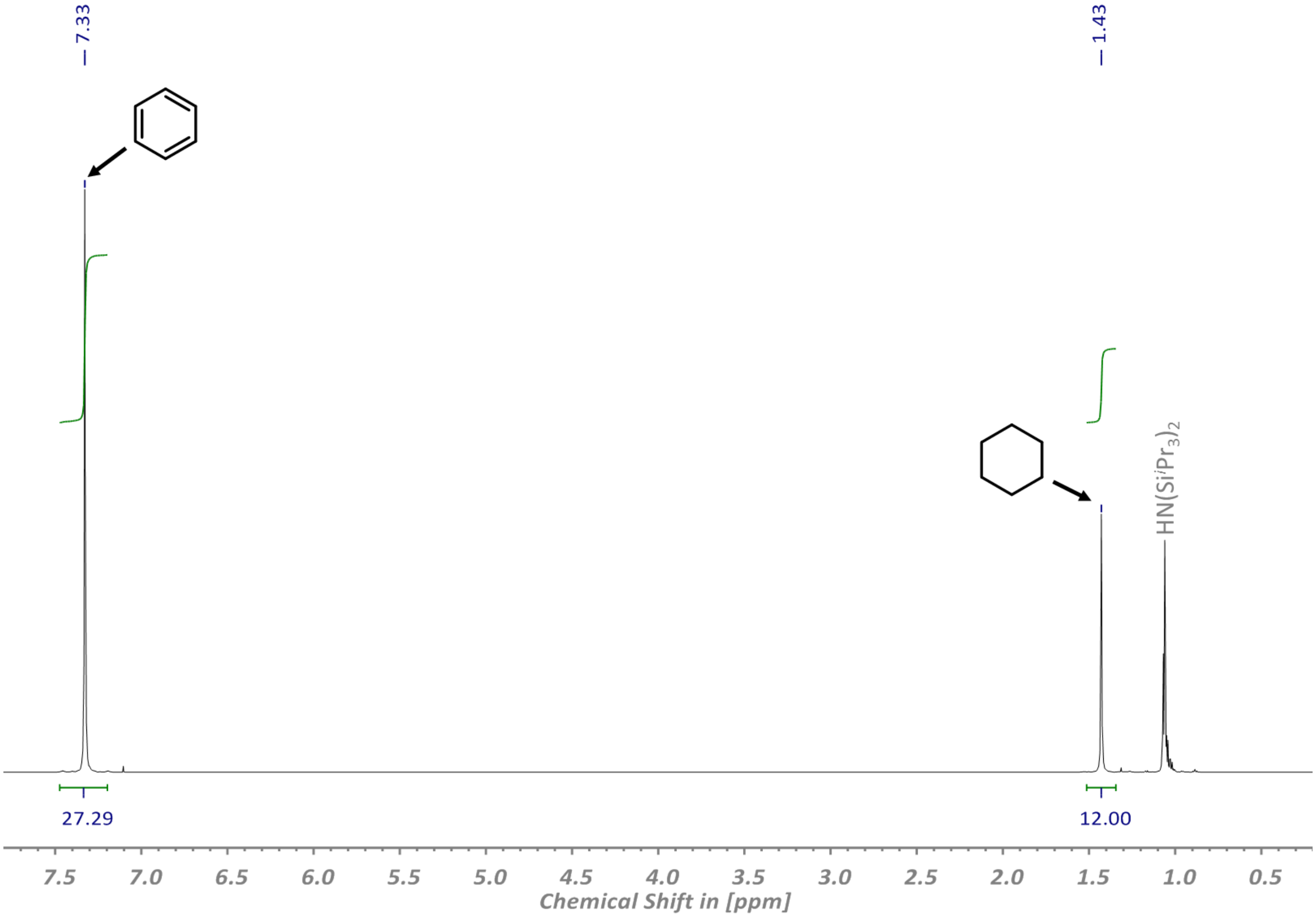

Figure S238. ^{1}H NMR spectrum (600 MHz, $CDCl_3$, 25 °C) after catalytic hydrogenation (72 h) of benzene with Ba[N(Si^{i}Pr$_3$)$_2$]$_2$ (**1-Ba**) (2.5 mol%) and H_2 (50 bar) at 140 °C showing the formation of cyclohexane.

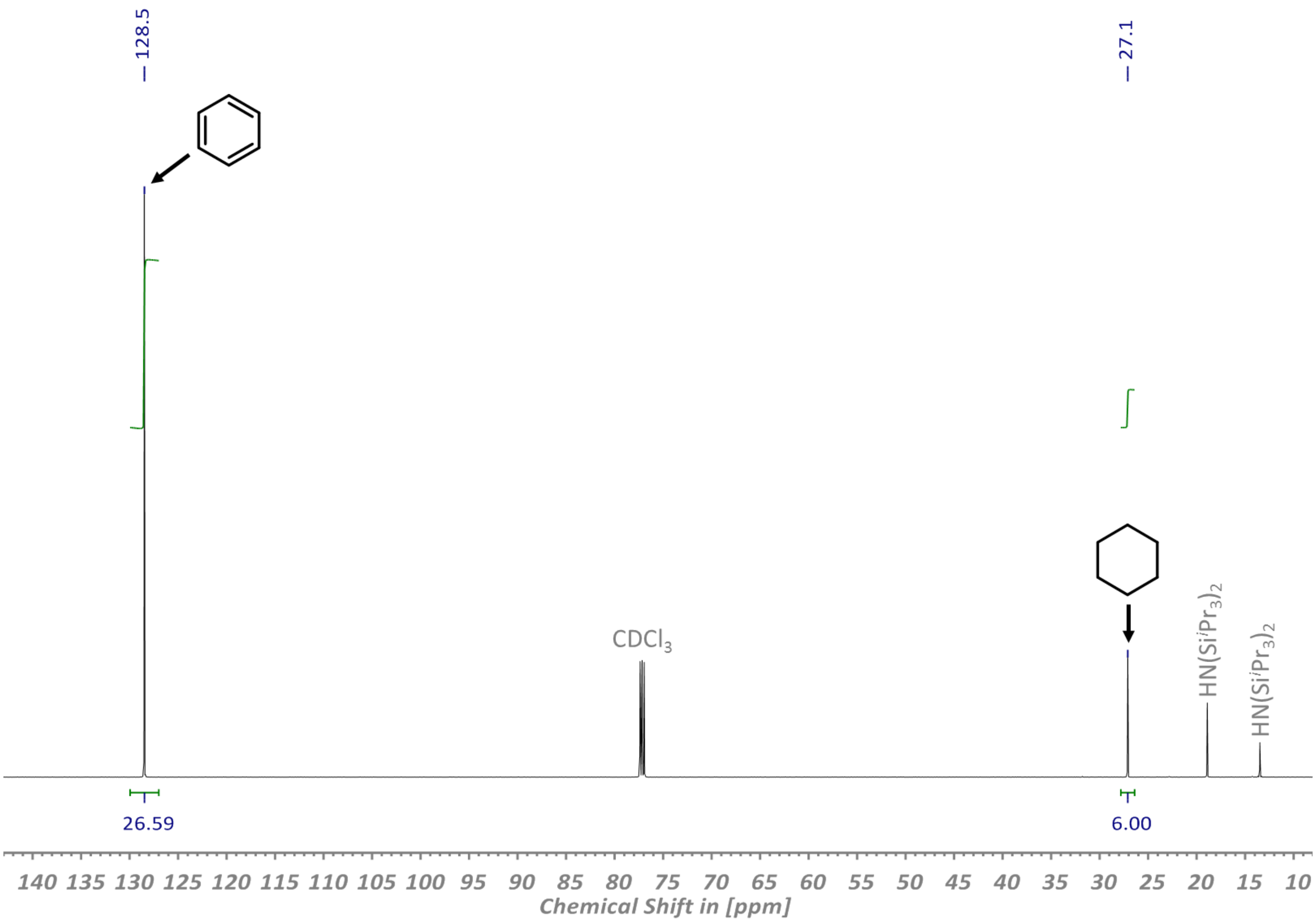

Figure S239. Quantitative $^{13}C\{^1H\}$ NMR spectrum (151 MHz, $CDCl_3$, 25 °C) after catalytic hydrogenation (72 h) of benzene with $Ba[N(Si^iPr_3)_2]_2$ (**1-Ba**) (2.5 mol%) and H_2 (50 bar) at 140 °C showing the formation of cyclohexane (18%).

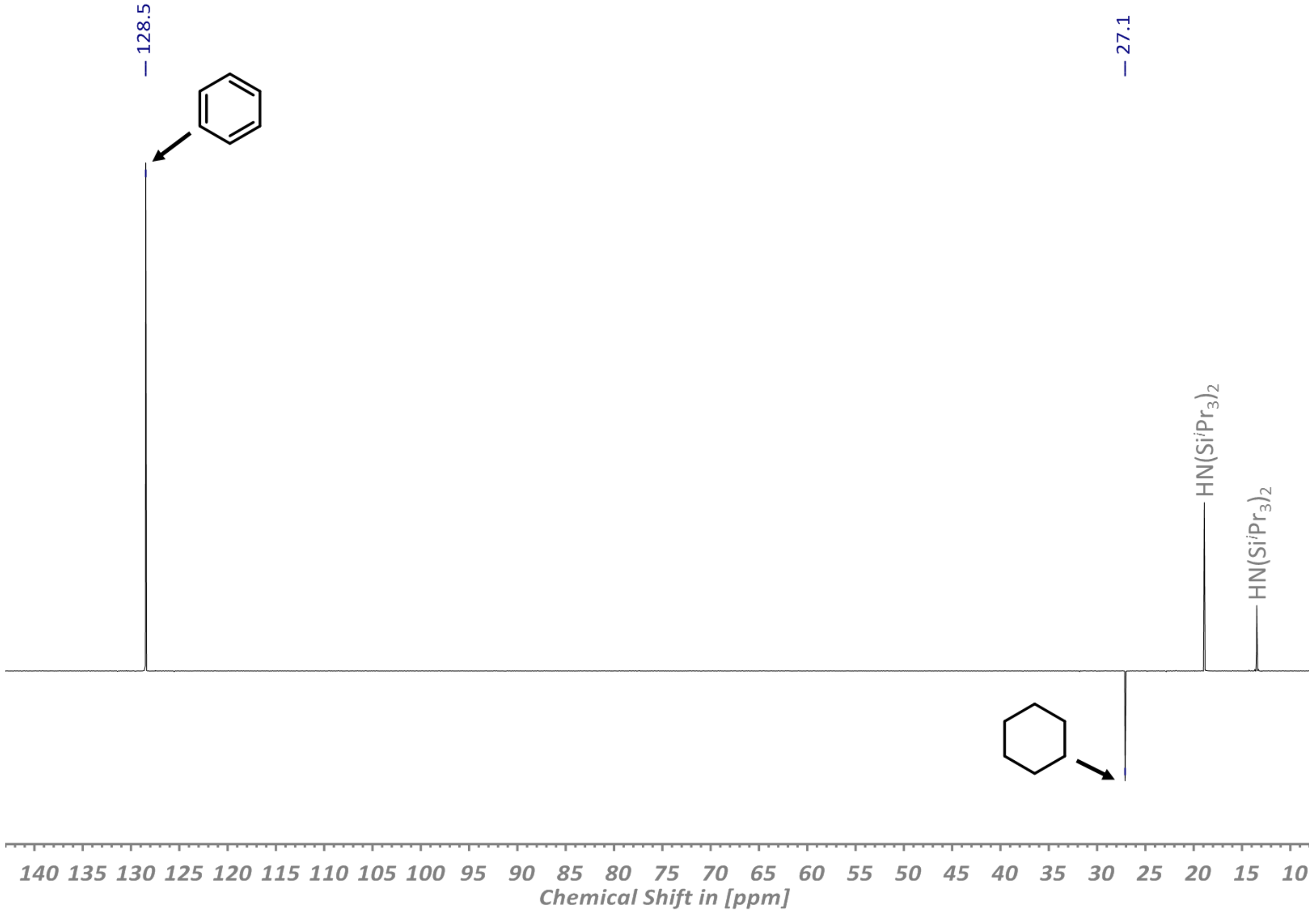

Figure S240. ^{13}C DEPT-135 NMR spectrum (151 MHz, $CDCl_3$, 25 °C) after catalytic hydrogenation (72 h) of benzene with Ba[N(Si^{i}Pr$_3$)$_2$]$_2$ (**1-Ba**) (2.5 mol%) and H_2 (50 bar) at 140 °C showing the formation of cyclohexane.

8 Reactions of $Ae[N(Si^{i}Pr_3)_2]_2$ with H_2

Reaction of $Ba[N(Si^{i}Pr_3)_2]_2$ with H_2 in C_6D_6

In an N_2-filled glovebox, $Ba[N(Si^{i}Pr_3)_2]_2$ (20 mg, 0.025 mmol) was dissolved in C_6D_6 (600 µL) in a J. Young NMR tube, which was sealed and then transferred out of the glovebox. The reaction solution was degassed *via* three consecutive freeze-pump-thaw cycles and H_2 (1 bar) was admitted at room temperature. The J. Young NMR tube was then heated in a heating block set to 90 °C without stirring. The reaction progress was periodically (1 h-intervals) monitored using ^{1}H NMR spectroscopy.

Reaction of $Ca[N(Si^{i}Pr_3)_2]_2$ with H_2 in C_6D_6

In an N_2-filled glovebox, a J. Young NMR tube was charged with crystalline $Ca[N(Si^{i}Pr_3)_2]_2$ (20 mg, 0.029 mmol) and C_6D_6 (600 µL). The sealed NMR tube was taken out of the glovebox and the contents were frozen by submersion in liquid nitrogen. Following evacuation of the headspace and thawing, H_2 (1 bar) was introduced at room temperature and the tube re-sealed. The colorless solution was then mechanically agitated in a heating block set to 60 °C and periodically removed and analyzed v*ia* ^{1}H NMR spectroscopy.

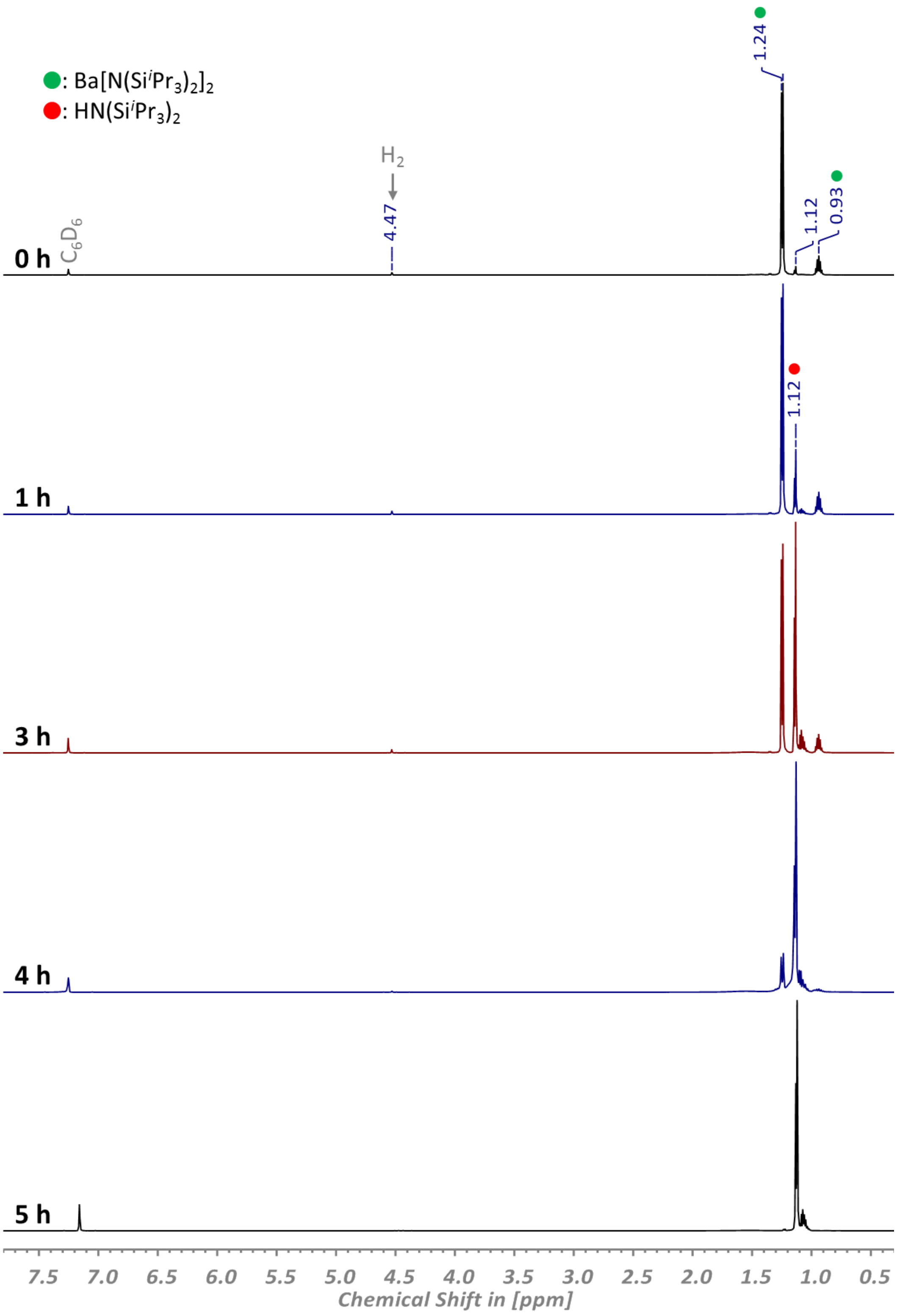

Figure S241. Stacked ^{1}H NMR spectra (600 MHz, C_6D_6, 25 °C) of the reaction of Ba[N(Si^{i}Pr$_3$)$_2$]$_2$ **(1-Ba)** with H_2 (1 bar) at 90 °C over 5 hours.

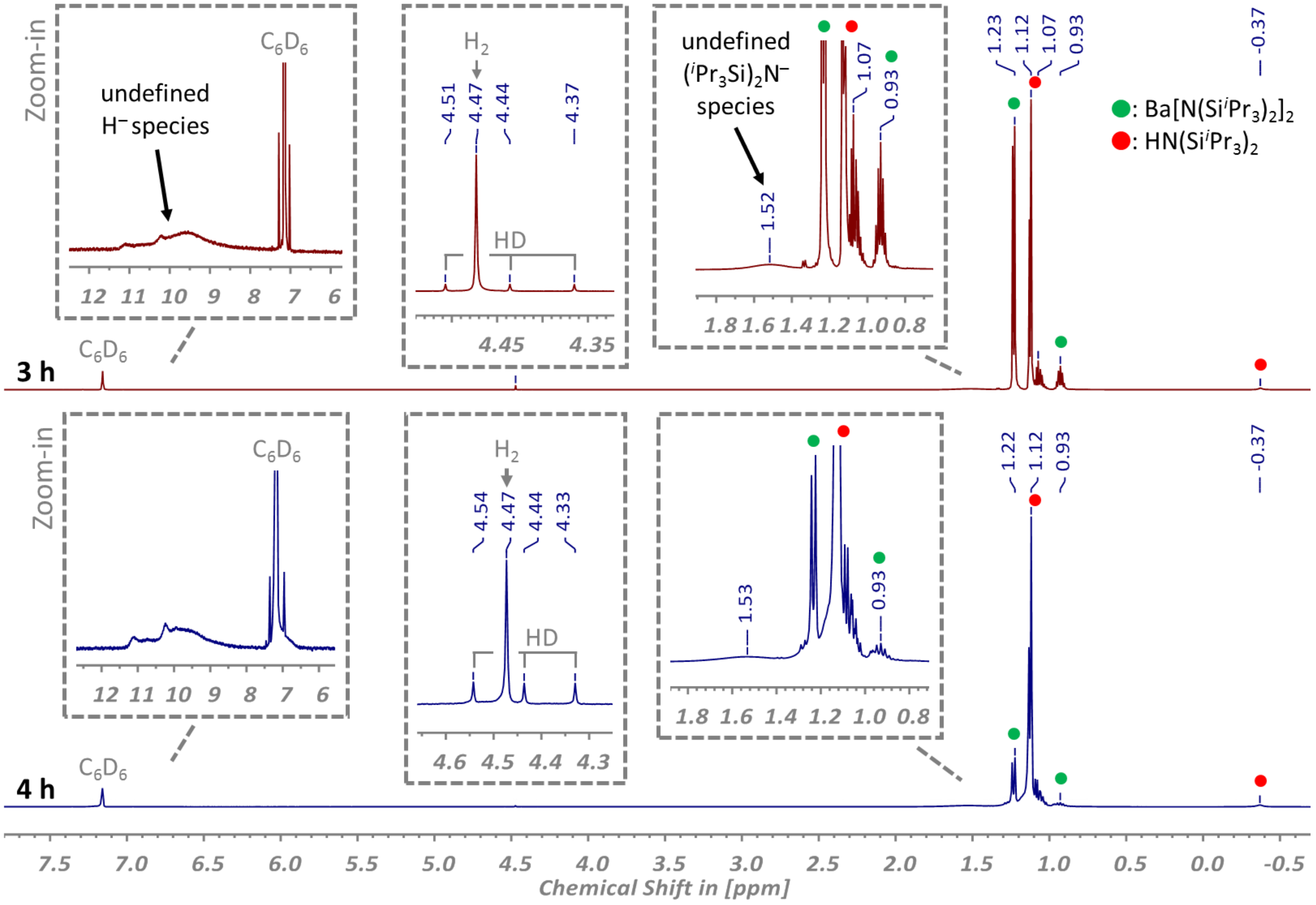

Figure S242. Stacked ^{1}H NMR spectra (600 MHz, C_6D_6, 25 °C) of the reaction of $Ba[N(Si^iPr_3)_2]_2$ **(1-Ba)** with H_2 (1 bar) at 90 °C after (*top*) 3 hours and (*bottom*) 4 hours.

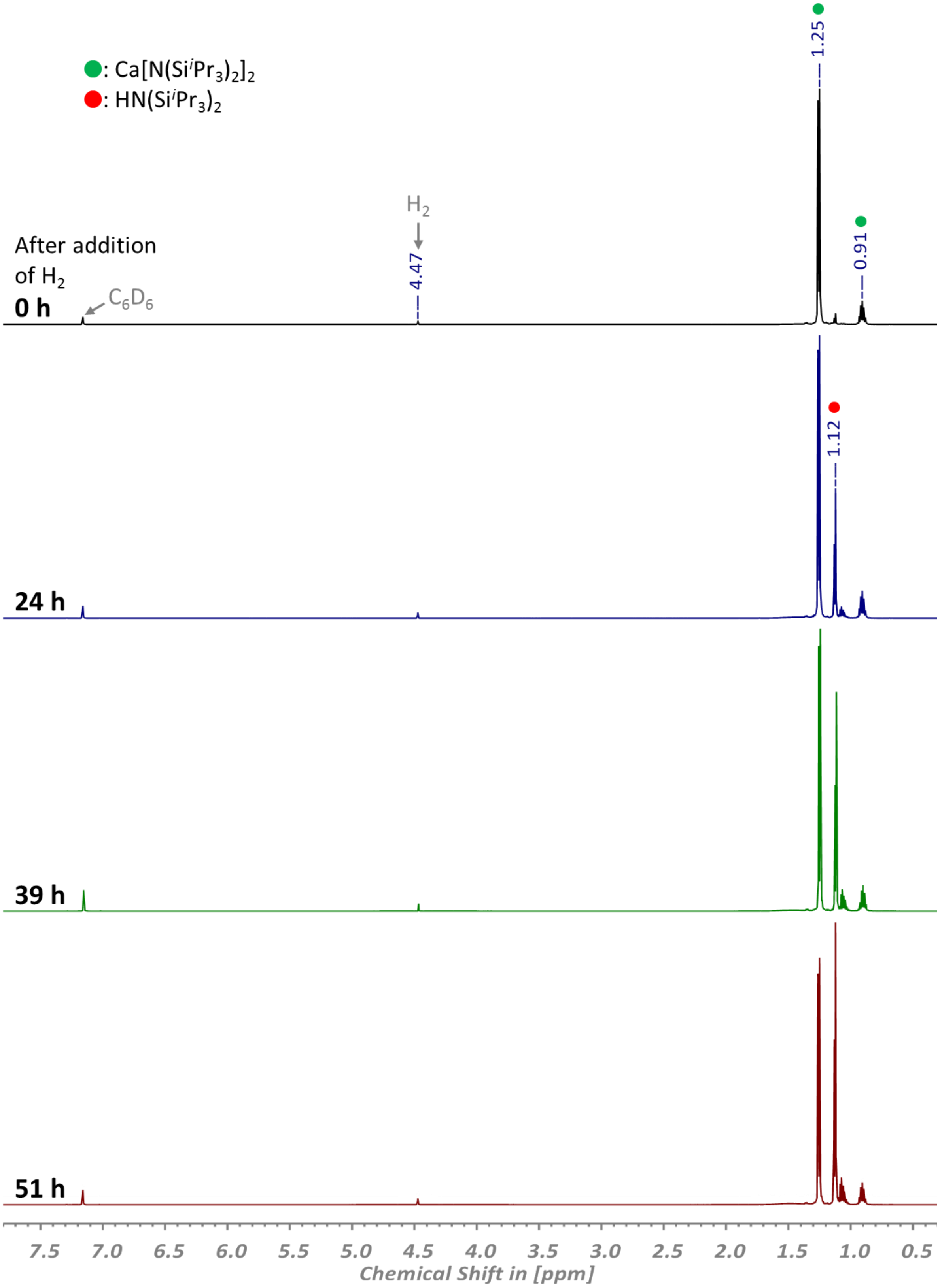

Figure S243. Stacked ^{1}H NMR spectra (600 MHz, C_6D_6, 25 °C) of the reaction of Ca[N(Si^{i}Pr$_3$)$_2$]$_2$ **(1-Ca)** with H_2 (1 bar) at 60 °C over 51 hours.

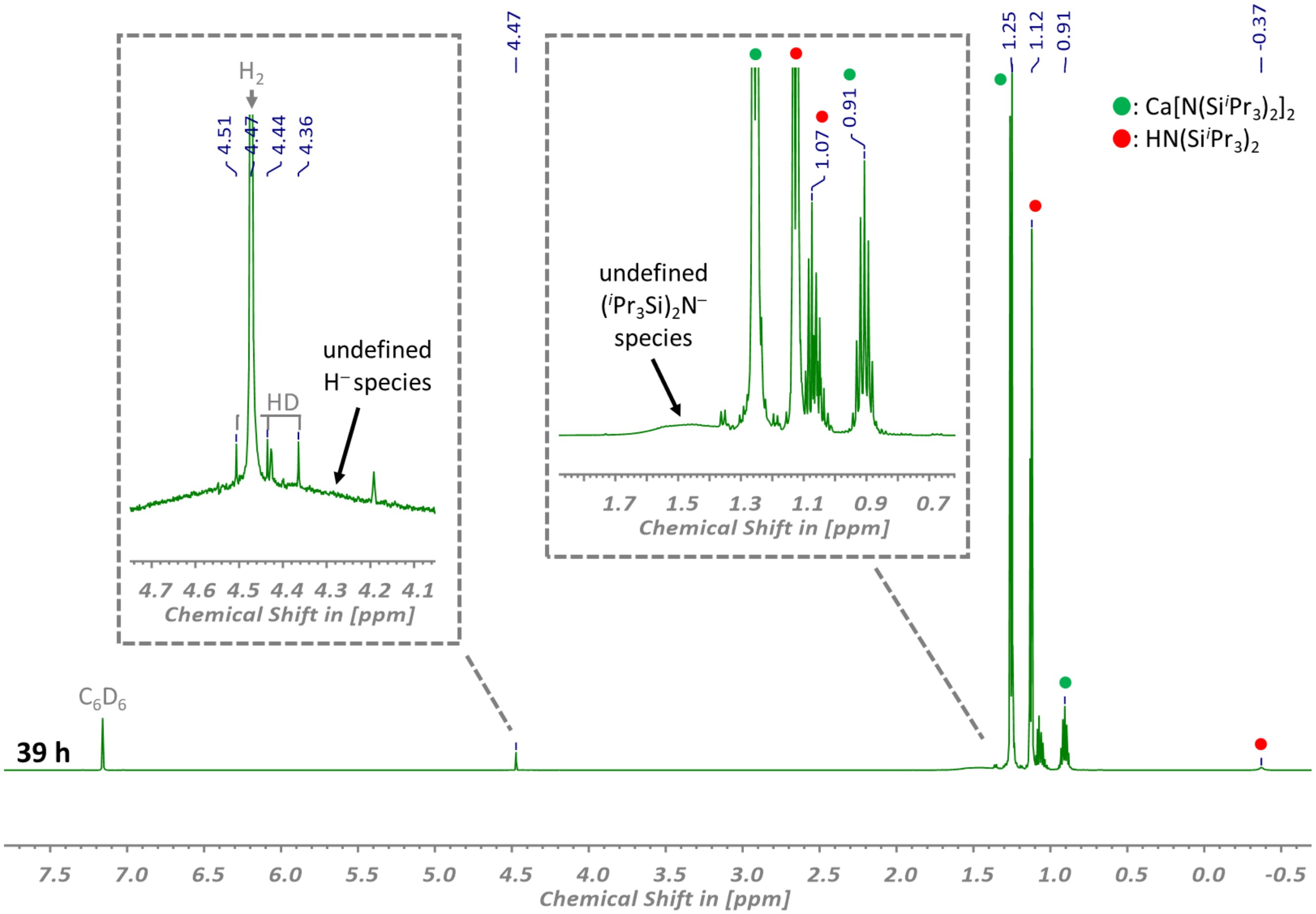

Figure S244. ^{1}H NMR spectrum (600 MHz, C_6D_6, 25 °C) of the reaction of Ca[N(Si^{i}Pr$_3$)$_2$]$_2$ **(1-Ca)** with H_2 (1 bar) at 60 °C after 39 hours.

9 Diffusion Ordered Spectroscopy

General methods

Diffusion measurements were conducted at 25 °C on a Bruker AVANCE NMR spectrometer operating at 600.13 MHz for proton resonance equipped with a 5 mm PABDO BB/19F-1H/D probe with Z-GRD and actively shielded gradient coil with a maximum gradient strength of 5.3500094 G/mm (at 10 A).

Parameter optimization was carried out empirically employing the pulse program ledbpgp2s1D using stimulated echo and LED (D21 = 5 ms, longitudinal eddy current delay as a Z-filter) with bipolar gradient pulses (P30) and two spoiling gradients (P19 = 600 µs) leading to values for gradient pulse length (in case of bipolar gradients ″little DELTA″*0.5) and diffusion time (D20 = 60 ms; ″big DELTA″). Delay for gradient recovery was set to 200 µs.

The diffusion experiment was executed with variable gradients from 2% to 98% gradient strength with 32 increment values (difframp calculated with the AU-program *DOSY*). In this case the pulse program ledbpgp2s was applied for data acquiring of this pseudo-2D Experiment. Data processing was performed with the T1/T2 software package (SimFit) of TopSpin (v3.2, Bruker Biospin) by fitting area data (integration of all peaks of interest of the same molecule) of diffusion decays. From these Stejskal-Tanner fitting curves calculated diffusion constants were obtained (with *Gamma* values for proton γ = 4258 Hz/G) and assimilated statistically.

For molecular weight estimation Stalke's method was applied was applied: external calibration curves ECC's under assumption of DSE-shaped molecules (dissipated spheres and ellipsoids) with the residual *protio*-solvent signal of deuterated benzene as internal reference with normalized diffusion coefficients.[S30]

Diffusion ordered spectroscopy on Ca[N(Si^{i}Pr$_3$)$_2$]$_2$

Table S5. Results for diffusion measurement of Ca[N(Si^{i}Pr$_3$)$_2$]$_2$ **(1-Ca)** applying ^{1}H DOSY in C_6D_6.

	**Ca[N(Si^{i}Pr$_3$)$_2$]$_2$** ($C_{36}H_{84}N_2Si_4Ca$)
**Theoretical molecular weight MW$^{Calc.}$** in [g mol^{-1}]	697
**van-der-Waals density** in [g (mol m^3)$^{-1}$]	$4.02 \cdot 10^{29}$
**Diffusion coefficient D** in [m s^{-1}]	$6.765 \cdot 10^{-10}$
**Determined molecular weight MW^{Exp}** in [g mol^{-1}] (applied method)	515 (ECC merge)<br>469 (ECC DSE)

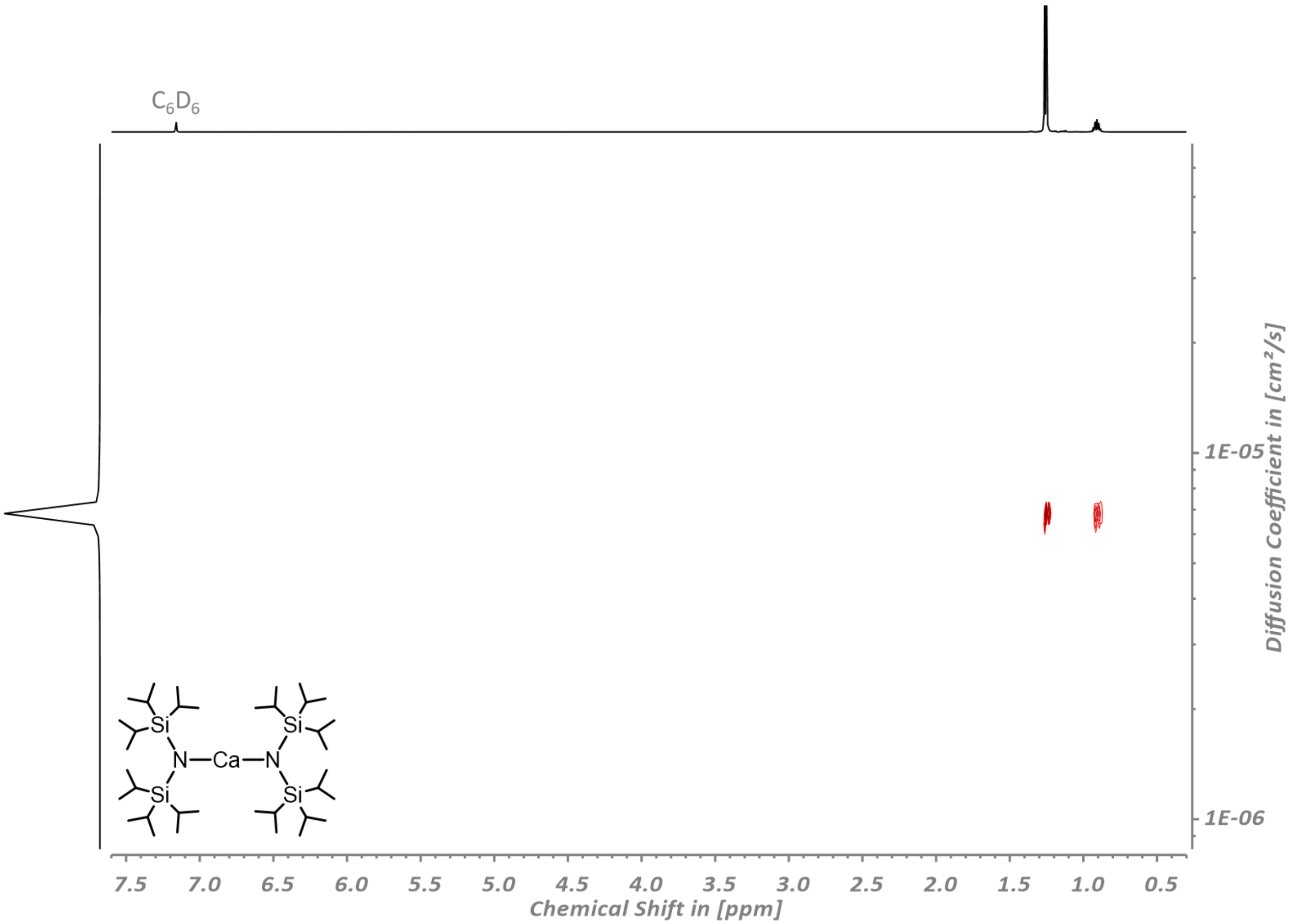

Figure S245. ^{1}H DOSY NMR spectrum of Ca[N(Si^{i}Pr$_3$)$_2$]$_2$ **(1-Ca)** in C_6D_6.

Diffusion ordered spectroscopy on the reaction of Ca[N(Si^{i}Pr$_3$)$_2$]$_2$ with H_2 (60 °C, 39 h, 1 bar H_2)

Table S6. Results for diffusion measurement of the reaction of Ca[N(Si$_i$Pr$_3$)$_2$]$_2$ **(1-Ca)** with H_2 (1 bar) applying ^{1}H DOSY in C_6D_6.

	**Undefined Ca$_x$H$_y$[N(Si^{i}Pr$_3$)$_2$]$_z$ species**	HN(Si^{i}Pr$_3$)$_2$	Ca[N(Si^{i}Pr$_3$)$_2$]$_2$
**Theoretical molecular weight MW$^{Calc.}$** in [g mol^{-1}]	-	330	697
**Diffusion coefficient D** in [m s^{-1}]	$2.966 \cdot 10^{-10}$	$8.592 \cdot 10^{-10}$	$2.966 \cdot 10^{-10}$
**Determined molecular weight MW^{Exp}** in [g mol^{-1}] (applied method: ECC DSE)	2190	307	509

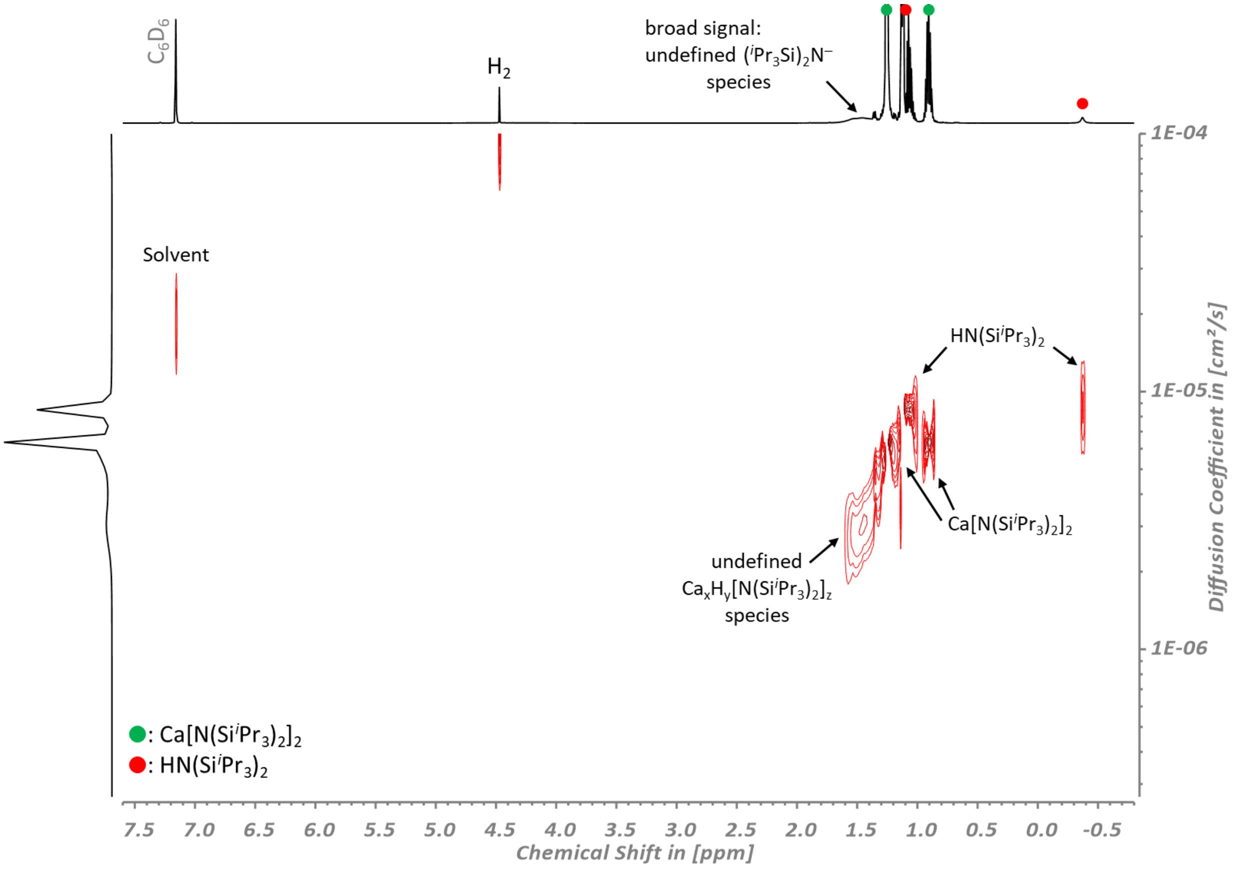

Figure S246. ^{1}H DOSY NMR spectrum on the reaction of Ca[N(Si^{i}Pr$_3$)$_2$]$_2$ **(1-Ca)** with H_2 (1 bar) in C_6D_6.

Diffusion ordered spectroscopy on $Ba[N(Si^iPr_3)_2]_2$ (1-Ba)

Table S7. Results for diffusion measurement of $Ba[N(Si^iPr_3)_2]_2$ **(1-Ba)** applying ^1H DOSY in C_6D_6.

	**$Ba[N(Si^iPr_3)_2]_2$** ($C_{36}H_{84}N_2Si_4Ba$)
**Theoretical molecular weight $MW^{Calc.}$** in [g mol^{-1}]	795
**van-der-Waals density** in [g (mol m^3)$^{-1}$]	$4.48 \cdot 10^{29}$
**Diffusion coefficient D** in [m s^{-1}]	$5.767 \cdot 10^{-10}$
**Determined molecular weight MW^{Exp}** in [g mol^{-1}] (applied method: ECC DSE)	548

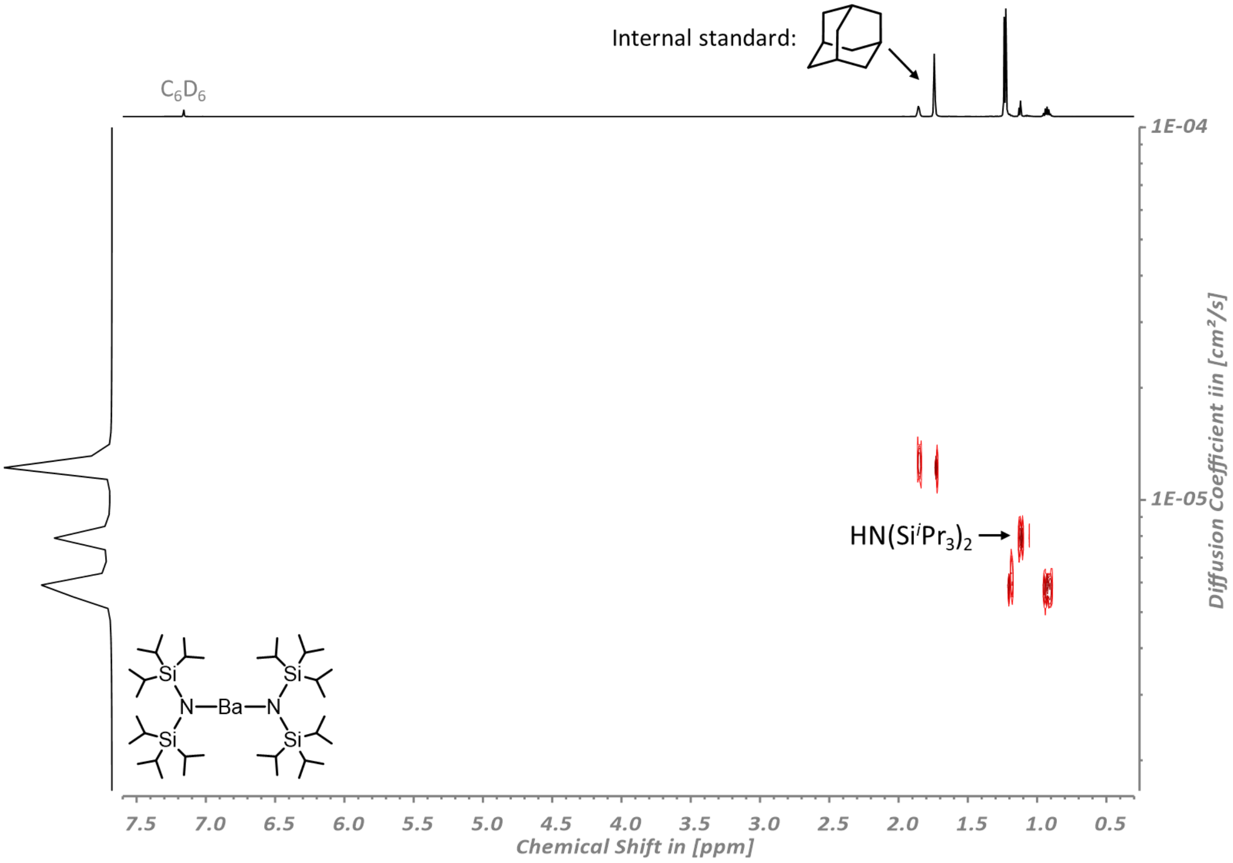

Figure S247. ^1H DOSY NMR spectrum of $Ba[N(Si^iPr_3)_2]_2$ **(1-Ba)** in C_6D_6 with adamantane as internal standard.

Diffusion ordered spectroscopy on the reaction of Ba[N(Si^{i}Pr$_3$)$_2$]$_2$ (1-Ba) with H_2 (3 h, 90 °C, 1 bar H_2)

Table S8. Results for diffusion measurement of the reaction of Ba[N(Si^{i}Pr$_3$)$_2$]$_2$ **(1-Ba)** with H_2 (1 bar) applying ^{1}H DOSY in C_6D_6.

	**Undefined Ba$_x$H$_y$[N(Si^{i}Pr$_3$)$_2$]$_z$ species**	HN(Si^{i}Pr$_3$)$_2$	Ba[N(Si^{i}Pr$_3$)$_2$]$_2$
**Theoretical molecular weight MW$^{Calc.}$** in [g mol^{-1}]	-	330	795
**Diffusion coefficient D** in [m s^{-1}]	$1.83\cdot10^{-10}$	$8.704\cdot10^{-10}$	$5.921\cdot10^{-10}$
**Determined molecular weight MW^{Exp}** in [g mol^{-1}] (applied method: ECC DSE)	3423	279	518

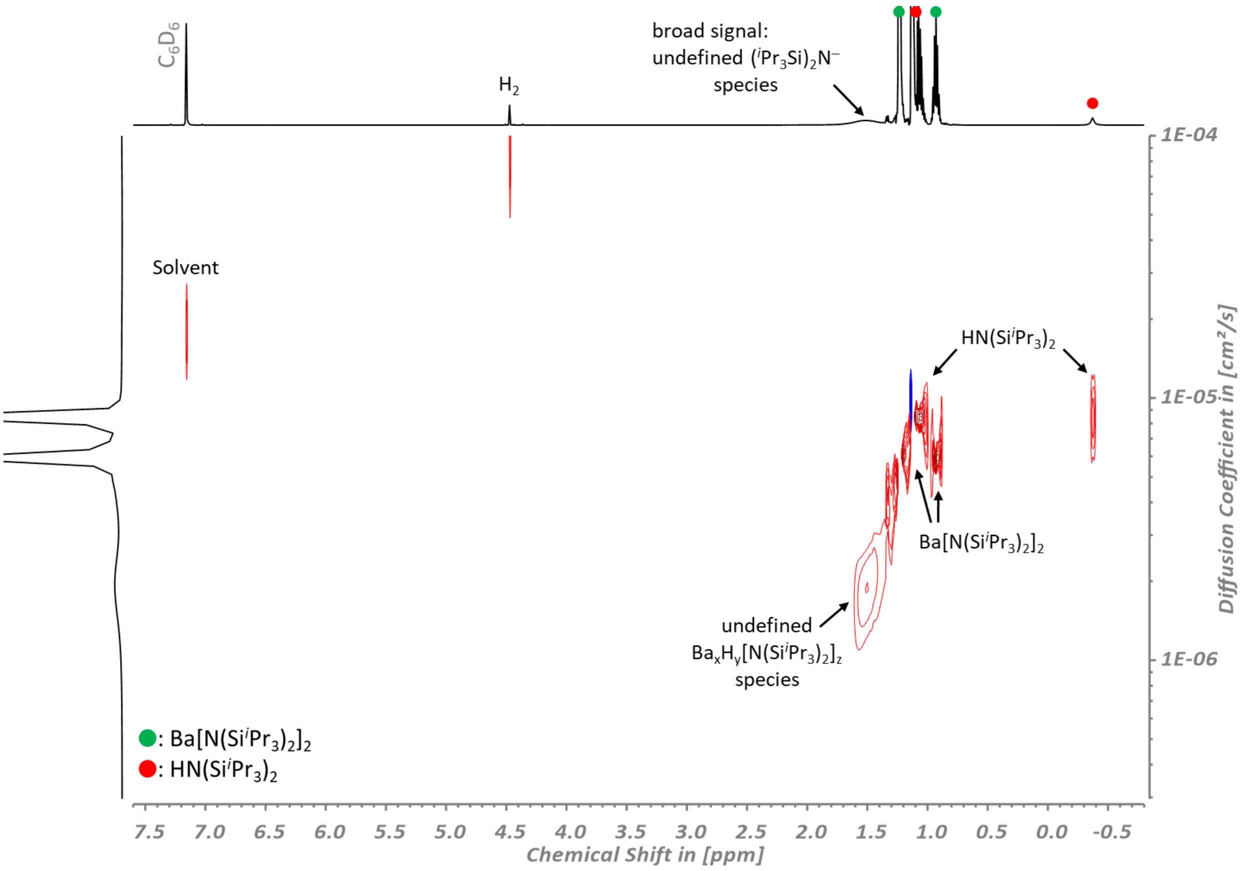

Figure S248. ^{1}H DOSY NMR spectrum on the reaction of Ba[N(Si^{i}Pr$_3$)$_2$]$_2$ **(1-Ba)** with H_2 (1 bar) in C_6D_6.

10 Theoretical Calculations

Calculation of energy profiles for ethylene hydrogenation (monomeric catalysts)

All geometry optimizations were carried out using Gaussian 16 Rev. A.0.3.[S31] All structures were fully optimized at a B3PW91/def2TZVPP level of theory.[S32] In all cases Grimme's third dispersion correction with Becke-Johnson damping (GD3BJ) was added.[S33] In addition, implicit solvent effects were computed using the polarizable continuum model (PCM, benzene).[S34] In order to determine zero-point energies and to characterize the structures as minima, frequency analysis has been applied. Molecules were drawn and evaluated using Molecules v2.311.[S35]

Note: The following abbreviations are consistently used:

N* = general amide, $N(TRIP)_2$ = $(i Pr_3Si)_2N$ and N" = $(Me_3Si)_2N$.

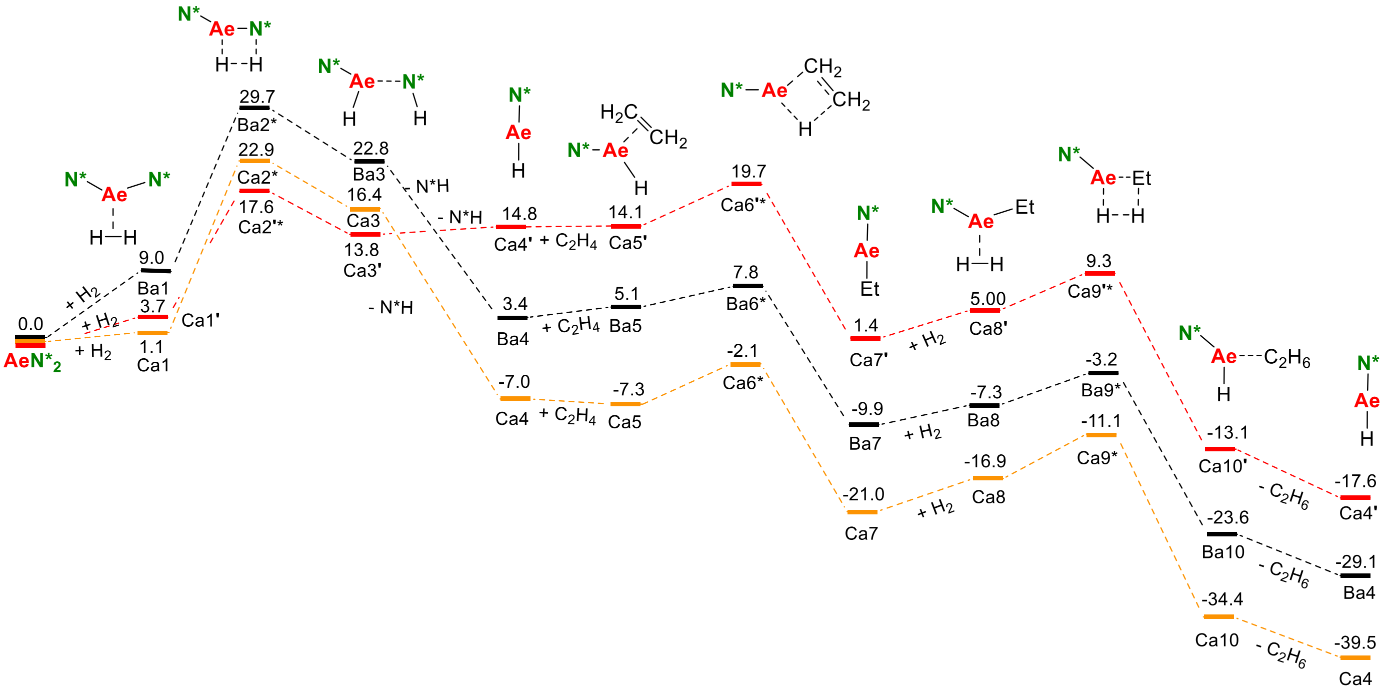

Scheme S1. Energy profiles (ΔH in kcal/mol) for the hydrogenation of ethylene by catalysts **1**-Ca (orange), **1**-Ba (black) and CaN''$_2$ (red). B3PW91/def2tzvpp including correction for dispersion (GD3BJ) and solvent (PCM=benzene).

Calculation of energy profiles for ethylene hydrogenation (dimeric catalysts)

Calculations on dimeric catalysts $(N^{*}AeH)_2$ have been performed at the same level of theory. Results are shown in Scheme S2.

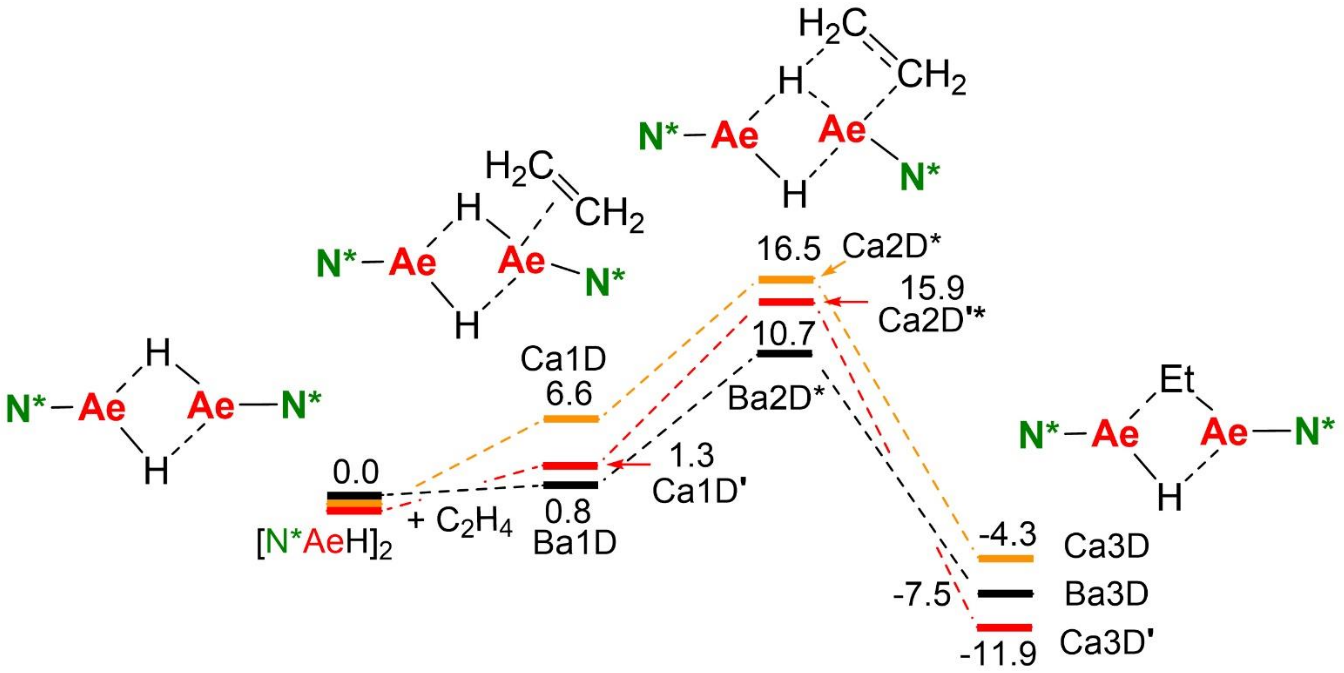

B3PW91/TZVPP PCM ΔH	$[HCaN(SiMe_3)_2]_2$	$[HCaN(Si\textit{i}Pr_3)_2]_2$	$[HBaN(Si\textit{i}Pr_3)_2]_2$
π-Ethylene Complex	1.3	6.6	0.8
TS	15.9	16.5	10.7
Alkyl product	-11.9	-4.3	-7.5

Scheme S2. Energy profiles (ΔH in kcal/mol) for the hydrogenation of ethylene by dimeric catalysts $[HCaN'']_2$ (red), $[HCaN(TRIP)_2]_2$ (orange) and $[HBaN(TRIP)_2]_2$. B3PW91/def2tzvpp including correction for dispersion (GD3BJ) and solvent (PCM=benzene).

Calculation of energy profiles for benzene hydrogenation

Calculations on benzene hydrogenation have been performed at the same level of theory. Results are shown in Scheme S3.

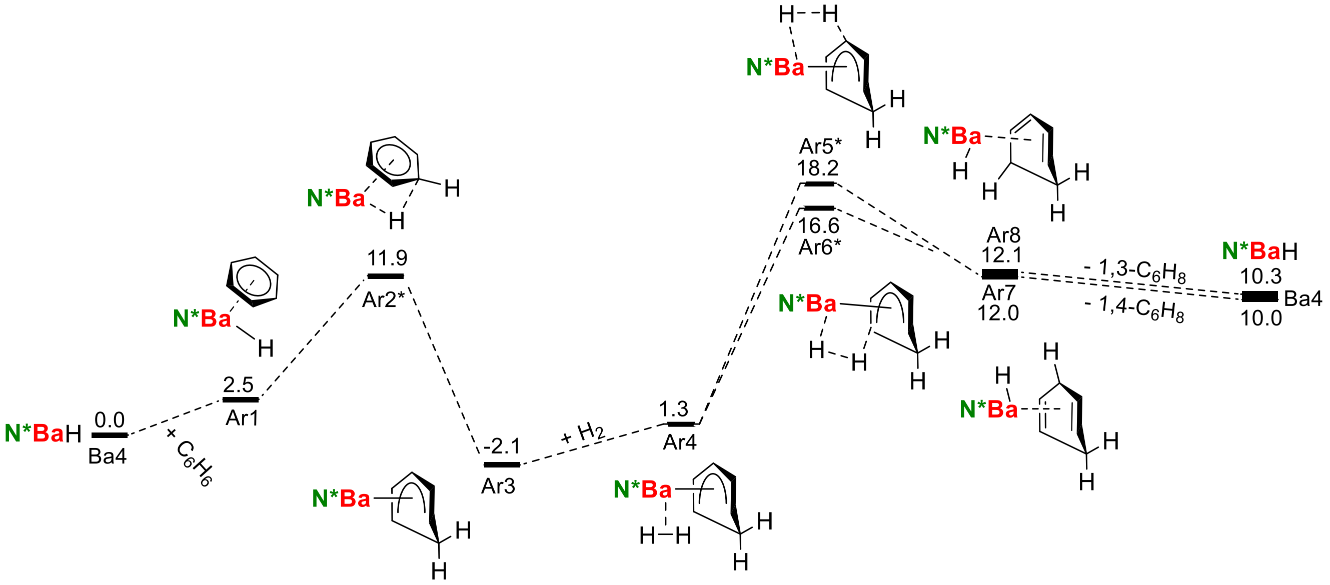

Scheme S3. Energy profiles (ΔH in kcal/mol) for benzene hydrogenation by catalyst **1**-Ba; B3PW91/def2tzvpp including correction for dispersion (GD3BJ) and solvent (PCM=benzene).

Calculations on catalyst aggregation

All structures were fully optimized on B3PW91/def2SVP level of theory. In all cases Grimme's third dispersion correction with Becke-Johnson damping (GD3BJ) was added. Single point calculations were carried out using B3PW91/def2TZVPP and solvent effects using the polarizable continuum model (PCM, benzene). Frequency calculations were run at the B3PW91/def2SVP level.

The thermodynamics of aggregation of three different $(R_2N)CaH$ species have been evaluated with amide ligands of increasing size: Me_2N < $(Me_3Si)_2N$ < $(iPr_3Si)_2N$. Structures of monomers, dimers and tetramers have been optimized (Scheme S4). Aggregation energies are shown in Table S9.

Table S9. Aggregation energies (ΔH and ΔG) for dimerization and tetramerization of Ca hydride/amide species with increasing amide size.

**ΔH (kcal/mol)**	$HCaNMe_2$	$HCaN(SiMe_3)_2$	$HCaN(Si\textit{i}Pr_3)_2$
2 monomers → dimer	-32.55	-29.41	-30.66
2 dimers → tetramer	-22.56	-13.58	+21.67
4 monomers → tetramer	-87.66	-72.40	-39.57

**ΔG (kcal/mol)**	$HCaNMe_2$	$HCaN(SiMe_3)_2$	$HCaN(Si\textit{i}Pr_3)_2$
2 monomers → dimer	-24.59	-18.70	-19.95
2 dimers → tetramer	-6.61	+15.84	+48.05
4 monomers → tetramer	-55.78	-21.55	+8.15

Scheme S4. Optimized structures for $(R_2NCaH)_x$ cluster with R = Me, $SiMe_3$ or Si*i*Pr_3 and x = 1,2 or 4. Note that $[(Me_3Si)_2NCaH]_4$ features a minimum with unusual bridging of $N(SiMe_3)_2$ anions.

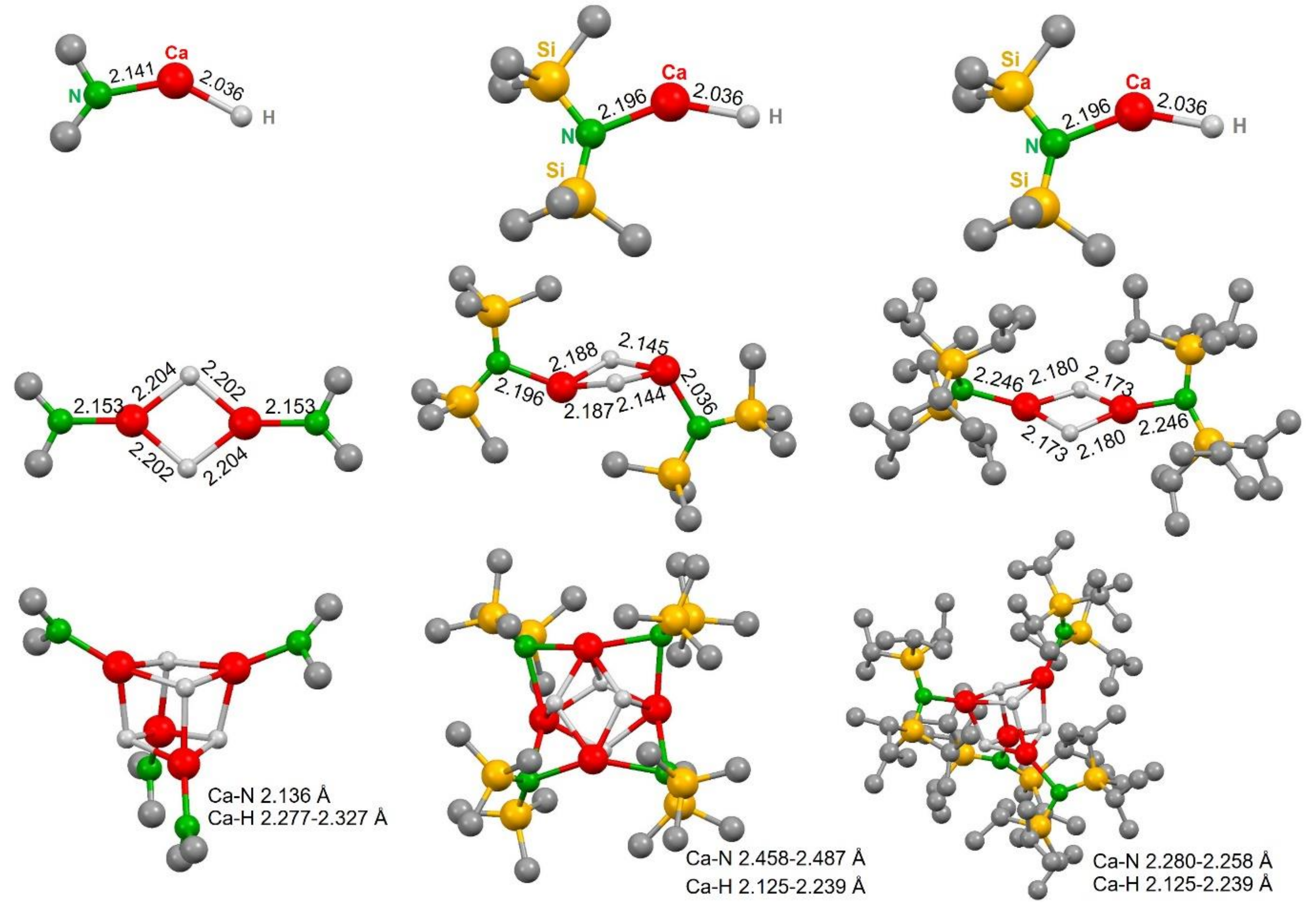

XYZ-Files for aggregation of R_2NCaH

22

$(Me_2NCaH)_2$ Dimer

Ca	1.697909	-0.048997	0.000318
N	3.850642	-0.040166	0.000942
C	4.502686	1.240764	0.000789
C	4.805338	-1.111567	0.000828
H	0.041084	1.402086	-0.000785
H	5.154032	1.409464	0.889889
H	5.153782	1.409399	-0.888510
H	3.764442	2.071126	0.000885
H	5.479252	-1.100366	0.888873
H	4.302097	-2.096844	0.001096

H	5.478809	-1.100597	-0.887559
Ca	-1.697705	0.047090	-0.001874
N	-3.850492	0.040274	-0.000050
C	-4.802802	1.113804	0.000730
C	-4.505710	-1.239046	0.000474
H	-0.040818	-1.403999	-0.000761
H	-5.156708	-1.405978	0.890174
H	-5.158135	-1.405983	-0.888179
H	-3.769619	-2.071250	-0.000098
H	-5.475799	1.104229	0.889487
H	-4.297472	2.098050	0.000394
H	-5.477169	1.104315	-0.886990

11

Me_2NCaH Monomer

Ca	1.465204	0.003993	-0.000369
N	-0.673642	-0.086729	-0.000024
C	-1.702360	-1.086048	-0.000073
C	-1.224158	1.239325	0.000367
H	3.108568	-1.201087	0.004531
H	-2.372127	-1.024340	0.888268
H	-2.372029	-1.024360	-0.888492
H	-1.266122	-2.100186	-0.000012
H	-1.854196	1.471413	0.890885
H	-0.418458	2.014482	0.000029
H	-1.855129	1.471663	-0.889425

44

$(Me_2NCaH)_4$ Tetramer

Ca	-1.495333	-1.554014	-0.967859
Ca	-1.000383	0.968332	1.479258
N	-3.320955	-2.522788	-1.506831
N	-1.724340	2.558995	2.707171
C	-4.310573	-1.837481	-0.724013
C	-2.454063	2.850780	3.904827
C	-3.900586	-3.548808	-2.321320
C	-1.296206	3.755074	2.036404
H	-1.171451	0.692181	-0.781593
H	-0.792767	-1.335408	1.226598
H	-1.859942	3.428481	4.649800
H	-3.374263	3.451797	3.721253
H	-2.776290	1.921521	4.406794
H	-0.608748	4.394223	2.637158

H	-0.728406	3.516902	1.102257
H	-2.130164	4.416949	1.706913
H	-4.654711	-3.160616	-3.043868
H	-3.126061	-4.064095	-2.915861
H	-4.425559	-4.333310	-1.729267
H	-5.088522	-1.313235	-1.325738
H	-4.857803	-2.489863	-0.004794
H	-3.846880	-1.036063	-0.093115
Ca	1.496532	-1.553407	0.966363
Ca	1.000032	0.969044	-1.480447
N	3.321705	-2.523056	1.505412
N	1.723362	2.562214	-2.705444
C	3.900042	-3.549725	2.320013
C	1.294759	3.756328	-2.031490
C	4.312373	-1.838005	0.723703
C	2.452555	2.857465	-3.902565
H	0.793941	-1.334989	-1.228358
H	1.171263	0.692659	0.780266
H	0.606773	4.396659	-2.630377
H	0.727315	3.515455	-1.097784
H	2.128418	4.417799	-1.700440
H	1.857903	3.436798	-4.645843
H	3.372514	3.458464	-3.717729
H	2.775074	1.929664	-4.407026
H	5.090165	-1.314541	1.326314
H	4.859791	-2.490431	0.004667
H	3.849667	-1.036057	0.092790
H	4.653735	-3.162241	3.043392
H	3.124708	-4.064837	2.913661
H	4.425109	-4.334281	1.728116

58

$(N''CaH)_2$ Dimer

Ca	1.205559	0.061779	-0.008958
H	-0.172414	-0.940746	1.361908
Ca	-1.729580	-1.465131	-0.015942
H	-0.169868	-0.933674	-1.389091
Si	-4.824452	-0.932803	0.019664
Si	-3.126507	1.681657	-0.020508
N	-3.387358	-0.012356	-0.008179
C	-4.252611	-2.779161	0.019895
C	-5.926555	-0.729683	-1.495374
C	-5.876062	-0.715667	1.568282

C	-1.235192	1.958297	-0.015717
C	-3.816562	2.558568	1.500235
C	-3.803215	2.531980	-1.562160
Si	4.105344	-1.649039	-0.010462
Si	4.076341	1.486655	0.018774
N	3.406580	-0.082499	-0.001013
C	2.660826	-2.887818	-0.030134
C	5.150953	-2.007352	1.521175
C	5.170382	-1.979979	-1.534869
C	2.571447	2.682768	0.025529
C	5.103443	1.921697	-1.502688
C	5.091069	1.889018	1.557429
H	5.419215	2.977775	-1.500700
H	6.011984	1.299610	-1.541202
H	4.534318	1.730730	-2.426320
H	5.405073	2.945356	1.581280
H	4.515144	1.676738	2.472145
H	6.000381	1.267679	1.589153
H	5.533564	-3.020467	-1.563016
H	4.598965	-1.789341	-2.457334
H	6.050771	-1.317324	-1.546497
H	5.513238	-3.048441	1.535342
H	6.031422	-1.345641	1.555872
H	4.567894	-1.832859	2.439539
H	3.025605	-3.927464	-0.036391
H	2.018911	-2.777623	0.862737
H	2.029757	-2.762817	-0.928668
H	1.936412	2.601763	-0.878158
H	1.929935	2.582896	0.922710
H	2.918093	3.728103	0.037616
H	-0.961555	3.023991	-0.019575
H	-0.820372	1.500147	0.901378
H	-0.815551	1.492566	-0.926835
H	-3.544950	3.626711	1.515615
H	-4.916260	2.493221	1.521942
H	-3.437811	2.091785	2.423395
H	-3.538123	3.601498	-1.590791
H	-3.410180	2.053553	-2.473344
H	-4.902067	2.458631	-1.596420
H	-6.720436	-1.423355	1.597960
H	-5.269930	-0.857218	2.477001
H	-6.292161	0.303708	1.606121
H	-6.771738	-1.436999	-1.490725

H	-6.343474	0.289617	-1.528055
H	-5.351015	-0.879445	-2.422466
H	-5.140073	-3.430882	0.034849
H	-3.699461	-3.082639	-0.892058
H	-3.675076	-3.074716	0.919252

29

N“CaH Monomer

H	-0.163246	4.243898	0.332641
Ca	-0.711448	2.316511	-0.026272
Si	1.699215	-0.219570	-0.009117
Si	-1.357772	-0.734270	0.001103
N	0.037941	0.252186	-0.039473
C	2.720940	1.264665	-0.587716
C	2.067854	-1.683912	-1.143344
C	2.260856	-0.677907	1.734784
C	-2.856206	0.487370	0.109213
C	-1.490095	-1.864190	1.502163
C	-1.657982	-1.752191	-1.556219
H	-3.783341	-0.105330	0.156195
H	-2.882635	1.112650	1.024644
H	-2.994865	1.134369	-0.781472
H	-2.456485	-2.392221	1.542679
H	-0.694375	-2.625945	1.468664
H	-1.362607	-1.295542	2.436738
H	-2.626033	-2.278168	-1.524867
H	-1.639713	-1.112730	-2.453187
H	-0.866943	-2.508573	-1.676605
H	3.330391	-0.943183	1.764298
H	2.100887	0.162931	2.429110
H	1.688610	-1.538075	2.117480
H	3.147420	-1.904128	-1.173706
H	1.554130	-2.594140	-0.792940
H	1.731100	-1.482511	-2.172733
H	3.801816	1.057788	-0.531532
H	2.490575	1.522711	-1.635263
H	2.536281	2.161429	0.030522

116

$(N“CaH)_4$ Tetramer

Ca	-1.642143	0.916692	0.835238
Ca	0.917021	1.640223	-0.836441
H	-0.986959	0.573067	-1.156710

H	0.572699	0.985934	1.155519
Ca	1.640570	-0.916616	0.837750
Ca	-0.916228	-1.640769	-0.837639
H	-0.574892	-0.985831	1.154384
H	0.986630	-0.573135	-1.155264
N	3.126310	0.833436	-0.119337
Si	4.137363	1.165302	1.243926
Si	3.817898	0.571383	-1.682628
C	4.157733	-1.251659	-2.003265
H	4.926827	-1.625572	-1.308925
H	4.500076	-1.454310	-3.030352
H	3.234623	-1.825586	-1.835313
C	5.390538	1.560716	-2.034080
H	5.201377	2.642478	-1.946399
H	5.743597	1.362411	-3.059364
H	6.211760	1.307815	-1.347083
C	5.619686	-0.013676	1.354735
H	5.287087	-1.065758	1.348480
H	6.181314	0.150069	2.289288
H	6.323950	0.110465	0.518212
C	4.801595	2.927018	1.327043
H	5.474230	3.121099	0.477751
H	5.373693	3.091883	2.254578
H	3.989444	3.667628	1.292473
C	2.613582	1.167373	-3.047007
H	1.655934	0.617581	-3.070431
H	3.087212	0.983542	-4.023889
H	2.426928	2.257732	-3.019531
C	3.144215	0.809327	2.831673
H	2.105127	1.171905	2.763604
H	3.618989	1.306856	3.691582
H	3.126274	-0.264538	3.095358
N	-3.126369	-0.833556	-0.120201
Si	-4.137840	-1.165749	1.242554
Si	-3.816704	-0.570321	-1.683831
C	-4.156004	1.252682	-2.005224
H	-4.923378	1.627894	-1.309677
H	-4.500469	1.454340	-3.031796
H	-3.232160	1.826272	-1.840055
C	-5.388842	-1.559936	-2.036552
H	-5.200249	-2.641451	-1.944982
H	-5.739217	-1.364683	-3.063343
H	-6.211736	-1.304447	-1.352470

C	-5.619175	0.014687	1.352901
H	-5.285295	1.066394	1.350210
H	-6.183647	-0.150665	2.285442
H	-6.321274	-0.106334	0.514088
C	-4.803891	-2.926933	1.323810
H	-5.478910	-3.118035	0.475718
H	-5.374171	-3.092919	2.252256
H	-3.993051	-3.668819	1.285613
C	-2.611591	-1.165293	-3.048089
H	-1.654082	-0.615277	-3.070755
H	-3.085171	-0.979899	-4.024725
H	-2.425053	-2.255721	-3.022450
C	-3.149071	-0.812545	2.833711
H	-2.116324	-1.193693	2.779388
H	-3.642751	-1.292552	3.693029
H	-3.113614	0.263057	3.088461
N	0.834157	-3.124825	0.120446
Si	1.174896	-4.134470	-1.241788
Si	0.561772	-3.816765	1.681903
C	1.546244	-5.391254	2.038646
H	1.296578	-6.211995	1.349936
H	1.341153	-5.744149	3.062648
H	2.628755	-5.203395	1.957736
C	-1.264267	-4.152284	1.990073
H	-1.833927	-3.226301	1.822517
H	-1.474611	-4.497991	3.014488
H	-1.636779	-4.917104	1.290263
C	2.939140	-4.793616	-1.313242
H	3.676671	-3.978837	-1.273664
H	3.112885	-5.365443	-2.239308
H	3.129597	-5.465083	-0.462188
C	0.000022	-5.619501	-1.359843
H	0.120134	-6.323949	-0.522925
H	0.170818	-6.180220	-2.293675
H	-1.052732	-5.288918	-1.360330
C	1.152045	-2.614425	3.050160
H	2.243035	-2.430939	3.029299
H	0.960539	-3.086955	4.026124
H	0.604765	-1.655294	3.068955
C	0.826837	-3.143184	-2.832112
H	-0.246063	-3.122073	-3.099411
H	1.325919	-3.621541	-3.689189
H	1.191837	-2.105186	-2.764684

N	-0.831368	3.125579	0.119683
Si	-0.562835	3.815733	1.682463
Si	-1.172694	4.135578	-1.242155
C	0.000449	5.621871	-1.359436
H	-0.121645	6.326531	-0.522989
H	-0.169689	6.181990	-2.293757
H	1.053533	5.292398	-1.358299
C	-2.937697	4.792689	-1.313958
H	-3.674356	3.977312	-1.271171
H	-3.112770	5.361384	-2.241707
H	-3.128090	5.466767	-0.464934
C	1.262779	4.148292	1.996309
H	1.831557	3.221881	1.828558
H	1.470954	4.492318	3.021703
H	1.637723	4.913703	1.298422
C	-1.544975	5.391984	2.037925
H	-1.291919	6.212765	1.350515
H	-1.340996	5.743494	3.062651
H	-2.627800	5.206930	1.954871
C	-0.821693	3.143207	-2.831308
H	0.250024	3.134737	-3.103683
H	-1.332302	3.609952	-3.687956
H	-1.173955	2.100969	-2.757063
C	-1.159289	2.613836	3.048562
H	-2.250672	2.432972	3.025202
H	-0.968940	3.085722	4.025073
H	-0.614317	1.653392	3.068938

130

$[(SiiPr_3)_2NCaH]_2$ Dimer

Ca	1.668184	-0.624993	-0.134431
Ca	-1.668300	-0.626725	0.132025
H	0.114732	-0.759844	1.379245
H	-0.114770	-0.758776	-1.381856
Si	-3.930807	1.439790	0.949316
Si	-4.770211	-1.275528	-0.606435
N	-3.844733	-0.075322	0.176072
C	-2.213392	2.276324	0.592486
H	-1.460644	1.649849	1.127928
C	-1.820374	2.317046	-0.887977
H	-1.971300	1.362502	-1.423275
H	-0.766132	2.606507	-1.027811
H	-2.434894	3.045620	-1.435047

C	-2.073558	3.663202	1.221938
H	-2.757967	4.379561	0.741916
H	-1.052656	4.065701	1.104789
H	-2.305060	3.654753	2.297389
C	-3.972585	1.221466	2.851412
H	-3.872914	2.224232	3.306081
C	-2.795046	0.364944	3.319101
H	-2.838123	-0.639132	2.857388
H	-2.811764	0.200672	4.410095
H	-1.813359	0.804633	3.080682
C	-5.289975	0.605867	3.322413
H	-6.165314	1.205053	3.028497
H	-5.318472	0.498950	4.420794
H	-5.423652	-0.399056	2.892642
C	-5.382926	2.552680	0.406652
H	-6.235798	1.847644	0.418426
C	-5.715500	3.694436	1.369453
H	-5.899031	3.340037	2.394457
H	-6.619559	4.237349	1.042971
H	-4.900403	4.433056	1.421893
C	-5.240253	3.066408	-1.027128
H	-4.477724	3.859526	-1.091243
H	-6.185092	3.503561	-1.393315
H	-4.948972	2.270617	-1.727734
C	-3.450119	-2.635503	-1.027177
H	-2.624653	-2.076775	-1.529144
C	-2.904647	-3.306173	0.243807
H	-2.760419	-2.620162	1.102372
H	-1.958395	-3.848799	0.069839
H	-3.628592	-4.042511	0.623464
C	-3.844174	-3.694938	-2.057365
H	-3.020559	-4.407769	-2.236456
H	-4.099483	-3.240092	-3.024297
H	-4.716519	-4.277066	-1.726797
C	-5.529064	-0.601015	-2.222248
H	-6.056708	0.304359	-1.866313
C	-4.426608	-0.139033	-3.176866
H	-3.694629	0.505386	-2.666186
H	-4.835780	0.432266	-4.027855
H	-3.871975	-0.992447	-3.601309
C	-6.559604	-1.478621	-2.932844
H	-6.127588	-2.429525	-3.280600
H	-6.968576	-0.968286	-3.822463

H	-7.410854	-1.727032	-2.282272
C	-6.118647	-2.021794	0.524001
H	-5.602451	-2.097363	1.500179
C	-7.276351	-1.035159	0.693091
H	-6.922997	-0.030016	0.964281
H	-7.973908	-1.360943	1.483832
H	-7.861138	-0.937018	-0.235563
C	-6.633317	-3.415619	0.162459
H	-7.087652	-3.442348	-0.840059
H	-7.407890	-3.748158	0.875636
H	-5.835715	-4.173242	0.178984
Si	3.932719	1.439805	-0.949032
Si	4.768915	-1.276753	0.606549
N	3.845242	-0.075444	-0.176360
C	2.215657	2.277317	-0.592597
H	1.462697	1.651841	-1.128917
C	1.821965	2.317095	0.887728
H	1.972461	1.362088	1.422330
H	0.767780	2.606815	1.027340
H	2.436531	3.045016	1.435617
C	2.077009	3.664751	-1.221081
H	2.761635	4.380319	-0.740193
H	1.056288	4.067772	-1.104152
H	2.309059	3.656922	-2.296420
C	3.975128	1.222034	-2.851171
H	3.876031	2.224957	-3.305620
C	2.797432	0.366093	-3.319536
H	2.840052	-0.638166	-2.858174
H	2.814404	0.202188	-4.410579
H	1.815811	0.806056	-3.081311
C	5.292459	0.606001	-3.321784
H	6.167913	1.204762	-3.027350
H	5.321375	0.499329	-4.420178
H	5.425543	-0.399078	-2.892186
C	5.385312	2.551546	-0.405381
H	6.237713	1.845930	-0.417275
C	5.718962	3.693654	-1.367369
H	5.902272	3.339805	-2.392605
H	6.623460	4.235566	-1.040441
H	4.904509	4.433014	-1.419372
C	5.242442	3.064455	1.028677
H	4.480300	3.857929	1.093035
H	6.187383	3.500880	1.395469

H	4.950505	2.268358	1.728656
C	3.446910	-2.635292	1.025736
H	2.621738	-2.075632	1.527157
C	2.901650	-3.304607	-0.246099
H	2.760057	-2.618107	-1.104713
H	1.953971	-3.845185	-0.073448
H	3.624450	-4.042496	-0.624907
C	3.838529	-3.695768	2.055756
H	3.013775	-4.407558	2.233737
H	4.093541	-3.241748	3.023162
H	4.710401	-4.278928	1.725732
C	5.527407	-0.603736	2.223118
H	6.056451	0.301147	1.868007
C	4.424840	-0.140897	3.177187
H	3.694001	0.504674	2.666338
H	4.834111	0.429461	4.028760
H	3.868869	-0.993862	3.600776
C	6.556330	-1.483021	2.934010
H	6.122874	-2.433618	3.280821
H	6.965182	-0.973723	3.824277
H	7.407817	-1.732042	2.283977
C	6.117205	-2.024015	-0.523395
H	5.601437	-2.098628	-1.499869
C	7.276044	-1.038557	-0.691438
H	6.923886	-0.032991	-0.962643
H	7.973837	-1.364863	-1.481758
H	7.860278	-0.941215	0.237651
C	6.630049	-3.418563	-0.162052
H	7.083805	-3.446169	0.840703
H	7.404609	-3.751811	-0.874917
H	5.831531	-4.175208	-0.179246

65

$(SiiPr_3)_2NCaH$ Monomer

Ca	-0.252488	2.756924	-0.164502
Si	-1.642983	-0.198444	0.163550
Si	1.549099	-0.170716	-0.036110
N	-0.057765	0.404068	0.020381
C	-2.752025	1.016141	-0.878336
H	-2.730664	1.983118	-0.321793
C	-2.226315	1.282667	-2.292871
H	-1.128862	1.413750	-2.327578
H	-2.699417	2.162200	-2.761623

H	-2.417702	0.420654	-2.947785
C	-4.232366	0.634651	-0.911129
H	-4.382626	-0.299459	-1.473906
H	-4.844205	1.410949	-1.401443
H	-4.640405	0.479510	0.098888
C	-2.278707	0.031245	1.962557
H	-3.359046	-0.202200	1.970879
C	-2.105322	1.478906	2.424792
H	-1.032258	1.752596	2.427448
H	-2.452994	1.631213	3.460619
H	-2.662875	2.202867	1.805026
C	-1.569659	-0.905807	2.939882
H	-1.693650	-1.965394	2.671087
H	-1.946232	-0.781963	3.970154
H	-0.488066	-0.699761	2.958145
C	-1.926308	-2.004671	-0.387890
H	-1.056409	-2.534862	0.041550
C	-3.189891	-2.658693	0.176078
H	-3.226542	-2.626861	1.274779
H	-3.252321	-3.719455	-0.122884
H	-4.103350	-2.167936	-0.193935
C	-1.854889	-2.176496	-1.906953
H	-2.750286	-1.762213	-2.397853
H	-1.803473	-3.242680	-2.186894
H	-0.977920	-1.677452	-2.343354
C	2.601382	1.454650	-0.180907
H	2.082915	2.030439	-0.984703
C	2.559751	2.264446	1.124377
H	1.584375	2.225668	1.648261
H	2.841035	3.323178	0.986687
H	3.265142	1.840650	1.854687
C	4.040382	1.326057	-0.680491
H	4.536872	2.310233	-0.737802
H	4.080703	0.878106	-1.682930
H	4.644277	0.693267	-0.013967
C	1.833051	-1.261638	-1.578089
H	1.035362	-2.021289	-1.473399
C	1.540328	-0.471615	-2.854659
H	0.568758	0.042203	-2.794206
H	1.515985	-1.124678	-3.744119
H	2.309926	0.295849	-3.043180
C	3.161927	-2.011515	-1.673414
H	4.023555	-1.328554	-1.725510

H	3.196761	-2.641571	-2.579567
H	3.324874	-2.675429	-0.812013
C	2.084039	-1.099435	1.551038
H	1.624167	-0.493221	2.355201
C	1.454450	-2.492584	1.611869
H	0.367403	-2.460789	1.455750
H	1.629288	-2.972552	2.590317
H	1.876206	-3.160358	0.843512
C	3.586201	-1.173530	1.830868
H	4.130314	-1.702632	1.033543
H	3.785622	-1.717156	2.770990
H	4.044243	-0.178902	1.932136
H	-0.406160	4.865528	-0.376327

260

$[(SiiPr_3)_2NCaH]_4$ Tetramer

Ca	-1.249361	1.758022	0.759608
Ca	0.742058	-1.053414	1.771637
H	0.873661	0.909940	0.711481
H	-1.419878	-0.455977	1.317200
Ca	-1.764836	-1.443893	-0.668869
Ca	1.274424	0.359600	-1.428634
H	-1.034635	0.649809	-1.136215
H	0.425815	-1.486147	-0.441862
Si	2.309549	-0.314072	-4.407739
Si	4.352527	1.382501	-2.683478
N	2.901801	0.499836	-3.019139
C	0.439522	-0.684868	-4.021776
H	0.399347	-1.162571	-3.010139
C	-0.372655	0.612447	-4.009179
H	0.133174	1.450589	-3.496450
H	-1.361574	0.502698	-3.547260
H	-0.526073	0.960883	-5.039122
C	-0.220687	-1.713215	-4.941414
H	-0.211671	-1.367663	-5.985357
H	-1.275432	-1.888069	-4.667559
H	0.290216	-2.684758	-4.911362
C	3.088067	-2.018265	-4.776542
H	2.477255	-2.435946	-5.598537
C	2.959028	-2.956143	-3.577814
H	3.571785	-2.604550	-2.735565
H	3.293098	-3.980508	-3.817221
H	1.924620	-3.029109	-3.211625

C	4.532705	-1.940127	-5.269645
H	4.630066	-1.322410	-6.175884
H	4.930624	-2.941260	-5.510929
H	5.195112	-1.507153	-4.504816
C	2.446703	0.718329	-6.013810
H	3.542768	0.848810	-6.078705
C	2.005260	0.007606	-7.294041
H	2.466288	-0.985268	-7.406247
H	2.272503	0.595932	-8.189059
H	0.912729	-0.132785	-7.323025
C	1.836549	2.119666	-5.927399
H	0.750959	2.098669	-6.103107
H	2.263861	2.793437	-6.689198
H	1.999195	2.581364	-4.941300
C	5.243479	0.472274	-1.242389
H	4.411224	0.241392	-0.549321
C	5.800167	-0.870448	-1.715684
H	5.035335	-1.458208	-2.239267
H	6.176530	-1.476615	-0.872949
H	6.645289	-0.743000	-2.408957
C	6.281085	1.253463	-0.435089
H	6.787003	0.597728	0.292873
H	5.821125	2.069067	0.139228
H	7.063086	1.695134	-1.069318
C	3.800111	3.111703	-2.073081
H	3.273205	3.537320	-2.945985
C	2.769144	2.951936	-0.956342
H	1.768841	2.730620	-1.365598
H	2.626553	3.879428	-0.381818
H	3.041519	2.183719	-0.212403
C	4.893107	4.103493	-1.673182
H	5.378362	3.821889	-0.728696
H	4.476677	5.116316	-1.532555
H	5.685172	4.178144	-2.432165
C	5.541543	1.631877	-4.174749
H	5.408456	0.716851	-4.779419
C	5.149881	2.829759	-5.045316
H	4.082624	2.853508	-5.290622
H	5.708646	2.827559	-5.997233
H	5.384779	3.779751	-4.540192
C	7.029591	1.734998	-3.828357
H	7.241980	2.620270	-3.207822
H	7.634986	1.837852	-4.745860

H	7.407447	0.857800	-3.286433
Si	3.338469	-2.561342	3.039063
Si	2.335151	0.164579	4.385715
N	2.435501	-1.128739	3.269235
C	2.787230	-3.130294	1.267459
H	1.675248	-3.211531	1.301917
C	3.172735	-2.077898	0.227685
H	2.970722	-1.040626	0.562987
H	2.681135	-2.246160	-0.742819
H	4.255055	-2.100697	0.054155
C	3.241506	-4.511715	0.797422
H	4.330308	-4.540043	0.653289
H	2.780110	-4.776123	-0.169179
H	2.983005	-5.301456	1.516592
C	2.848137	-3.972318	4.249853
H	2.932391	-4.908625	3.667293
C	1.392557	-3.823897	4.690803
H	1.269714	-2.952531	5.350732
H	1.036101	-4.712984	5.238653
H	0.706795	-3.673988	3.843801
C	3.754573	-4.111427	5.474791
H	4.798808	-4.324755	5.204155
H	3.412964	-4.931469	6.130123
H	3.755025	-3.193969	6.083275
C	5.237466	-2.371040	3.121431
H	5.412433	-2.243291	4.206253
C	6.010439	-3.616407	2.681127
H	5.592397	-4.549945	3.088837
H	7.067011	-3.562707	2.994812
H	6.012105	-3.711868	1.583954
C	5.776306	-1.123104	2.428120
H	5.667002	-1.188734	1.336161
H	6.851000	-0.979246	2.634132
H	5.249854	-0.215726	2.748168
C	0.497427	0.755368	4.195354
H	0.389846	0.954321	3.098820
C	-0.453737	-0.377580	4.595336
H	-0.169614	-1.356913	4.172112
H	-1.498824	-0.175914	4.318038
H	-0.425503	-0.531323	5.684722
C	0.096362	2.065053	4.871838
H	-0.971924	2.289963	4.717590
H	0.668362	2.917480	4.485974

H	0.260384	2.025449	5.957191
C	3.554476	1.558957	3.922750
H	4.532982	1.073558	4.095690
C	3.469136	1.894484	2.436835
H	3.625376	0.999350	1.816782
H	4.222066	2.645416	2.141352
H	2.482116	2.296781	2.168951
C	3.526752	2.818539	4.788835
H	2.615296	3.411858	4.622405
H	4.381170	3.477655	4.556651
H	3.573266	2.590614	5.864316
C	2.679658	-0.373679	6.193046
H	2.284112	-1.404935	6.221798
C	4.187839	-0.455203	6.445198
H	4.716414	-1.002020	5.650244
H	4.409489	-0.965786	7.398122
H	4.639541	0.547904	6.503266
C	1.991685	0.410741	7.310487
H	2.278369	1.474424	7.308062
H	2.267658	0.007539	8.300566
H	0.894888	0.366209	7.240546
Si	-3.948008	3.638168	1.375056
Si	-1.461819	4.913222	-0.142134
N	-2.410437	3.708239	0.629161
C	-4.171553	1.752537	1.768637
H	-3.793153	1.234240	0.855365
C	-3.330469	1.300605	2.964676
H	-2.301093	1.706829	2.987806
H	-3.249183	0.203856	3.023595
H	-3.776431	1.657670	3.904614
C	-5.608974	1.265235	1.950066
H	-6.095066	1.771310	2.797659
H	-5.645723	0.182161	2.152650
H	-6.221763	1.453837	1.059219
C	-5.456941	4.140587	0.299360
H	-6.278936	3.512548	0.686928
C	-5.236072	3.762868	-1.163987
H	-4.508608	4.436908	-1.638728
H	-6.170235	3.818792	-1.749002
H	-4.831664	2.745710	-1.273549
C	-5.921180	5.592658	0.427568
H	-6.209036	5.849955	1.457581
H	-6.800465	5.782209	-0.212608

H	-5.142182	6.305902	0.119804
C	-4.081183	4.662185	2.988915
H	-4.217329	5.689534	2.603054
C	-5.315915	4.333211	3.829561
H	-6.241995	4.328893	3.233586
H	-5.455067	5.062496	4.646453
H	-5.229697	3.341531	4.302233
C	-2.810522	4.673858	3.834815
H	-2.634074	3.702479	4.321381
H	-2.865409	5.426587	4.640251
H	-1.924645	4.898852	3.224451
C	-0.740851	4.035886	-1.704410
H	-0.269036	3.108742	-1.297088
C	-1.888635	3.578397	-2.605156
H	-2.593035	2.934365	-2.062114
H	-1.521714	3.025304	-3.483614
H	-2.459579	4.441572	-2.983001
C	0.337978	4.742070	-2.524875
H	0.741188	4.074126	-3.305818
H	1.184324	5.086036	-1.918822
H	-0.069389	5.622605	-3.040539
C	-0.063868	5.381388	1.083667
H	-0.618088	5.922571	1.872495
C	0.513717	4.129073	1.740825
H	-0.250908	3.617715	2.350281
H	1.344504	4.359340	2.427792
H	0.929858	3.414651	1.008103
C	1.033304	6.319654	0.584188
H	1.696717	5.828755	-0.142858
H	1.673380	6.668149	1.413218
H	0.620075	7.212272	0.091175
C	-2.415046	6.498821	-0.619159
H	-3.406446	6.113189	-0.914424
C	-2.613958	7.402690	0.599866
H	-2.988366	6.850300	1.474454
H	-3.335354	8.210424	0.387755
H	-1.669278	7.884706	0.898997
C	-1.868798	7.298011	-1.802234
H	-0.835053	7.639191	-1.632523
H	-2.479650	8.200000	-1.981054
H	-1.873608	6.714536	-2.734100
Si	-4.685917	-2.751102	-1.312781
Si	-2.349103	-4.689846	-0.332201

N	-3.128080	-3.238320	-0.805707
C	-4.554640	-0.818232	-1.457003
H	-4.042741	-0.482302	-0.522345
C	-3.723821	-0.372184	-2.664913
H	-2.823393	-0.986793	-2.857540
H	-3.402238	0.679458	-2.594899
H	-4.313147	-0.476418	-3.587541
C	-5.892224	-0.077242	-1.454426
H	-6.528085	-0.406003	-2.289143
H	-5.751784	1.009189	-1.559647
H	-6.452096	-0.247271	-0.526033
C	-6.110804	-3.038546	-0.066184
H	-6.850559	-2.261572	-0.328242
C	-5.623881	-2.754994	1.355291
H	-4.919235	-3.530526	1.691462
H	-6.457508	-2.726010	2.077985
H	-5.090036	-1.793809	1.427152
C	-6.842786	-4.377445	-0.153361
H	-7.244548	-4.567212	-1.160199
H	-7.694524	-4.406881	0.548333
H	-6.189832	-5.223566	0.101733
C	-5.203640	-3.498399	-2.995614
H	-5.435090	-4.544305	-2.718669
C	-6.476666	-2.901758	-3.597032
H	-7.304221	-2.856386	-2.871815
H	-6.829171	-3.490990	-4.461243
H	-6.306001	-1.876279	-3.962902
C	-4.069095	-3.552687	-4.019225
H	-3.862081	-2.560183	-4.448861
H	-4.316827	-4.217946	-4.864168
H	-3.135029	-3.914829	-3.566321
C	-1.141789	-4.167485	1.080736
H	-0.552336	-3.331322	0.629198
C	-1.905721	-3.615898	2.286123
H	-2.622469	-2.832877	1.998180
H	-1.235916	-3.190424	3.055292
H	-2.478404	-4.411694	2.787682
C	-0.118011	-5.228983	1.478244
H	0.525090	-4.894594	2.305396
H	0.540626	-5.486430	0.640653
H	-0.604099	-6.155886	1.810303
C	-1.318604	-5.343108	-1.806122
H	-2.089347	-5.463402	-2.590186

C	-0.347989	-4.268483	-2.290572
H	-0.879385	-3.339092	-2.559330
H	0.195545	-4.582919	-3.196626
H	0.410745	-4.015179	-1.531431
C	-0.618966	-6.693968	-1.653533
H	0.217518	-6.650565	-0.940842
H	-0.199102	-7.033726	-2.616331
H	-1.303060	-7.479728	-1.301344
C	-3.553695	-6.049942	0.259766
H	-4.330451	-5.471224	0.790469
C	-4.218013	-6.726401	-0.942601
H	-4.608602	-5.998788	-1.668579
H	-5.062882	-7.364154	-0.630739
H	-3.507442	-7.372042	-1.481883
C	-3.022453	-7.096188	1.239862
H	-2.182691	-7.672055	0.819756
H	-3.811936	-7.823362	1.498301
H	-2.676756	-6.648489	2.183154

XYZ-Files for hydride-ethylene insertion (monomeric catalysts HCaN(TRIP)$_2$, HBaN(TRIP)$_2$ and HCaN'')

6
Ethene C_2H_4

C	0.000000	0.000000	0.661945
C	0.000000	0.000000	-0.661945
H	0.000000	0.922698	1.230337
H	0.000000	-0.922698	1.230337
H	0.000000	0.922698	-1.230337
H	0.000000	-0.922698	-1.230337

8
Ethane C_2H_6

C	-0.746906	0.016216	0.143048
C	0.746906	-0.016216	-0.143048
H	-1.248874	0.786126	-0.446130
H	-1.219164	-0.938774	-0.095355
H	1.219164	0.938774	0.095355
H	1.248874	-0.786126	0.446130
H	0.947057	-0.226858	-1.195830
H	-0.947057	0.226858	1.195830

2
Hydrogen H_2

H	0.000000	0.000000	0.372376
H	0.000000	0.000000	-0.372376

127
BaN^*_2

Ba	0.000000	0.000003	-0.000053
Si	-3.244762	1.536516	-0.360082
Si	-3.244826	-1.536480	0.360108
N	-2.660374	0.000006	0.000013
C	-2.127795	2.204660	-1.771621
H	-1.147137	2.331817	-1.285369
C	-1.928514	1.216843	-2.917807
H	-1.736653	0.202385	-2.556504
H	-1.106678	1.514846	-3.576542
H	-2.827121	1.143833	-3.532628
C	-2.487919	3.587811	-2.304805
H	-3.424199	3.553256	-2.865067
H	-1.718908	3.968607	-2.984144
H	-2.611959	4.317810	-1.503356
C	-2.951654	2.759804	1.083118
H	-3.230885	3.754314	0.716901
C	-1.490327	2.820661	1.504549
H	-1.169139	1.853435	1.912158
H	-1.315645	3.543018	2.307467
H	-0.828975	3.104958	0.682837

C	-3.811582	2.436491	2.300947
H	-4.875326	2.406361	2.061631
H	-3.673374	3.173983	3.098223
H	-3.540512	1.460827	2.709772
C	-5.093681	1.666631	-0.782811
H	-5.578025	1.097341	0.019965
C	-5.664460	3.084085	-0.741628
H	-5.497381	3.574074	0.218052
H	-6.744758	3.070321	-0.918025
H	-5.223168	3.718221	-1.512395
C	-5.450514	0.991587	-2.104144
H	-5.044886	1.549524	-2.951740
H	-6.534980	0.941208	-2.244021
H	-5.060871	-0.023416	-2.163572
C	-2.127928	-2.204655	1.771681
H	-1.147248	-2.331801	1.285467
C	-1.928688	-1.216855	2.917888
H	-1.736765	-0.202406	2.556598
H	-1.106900	-1.514890	3.576668
H	-2.827329	-1.143824	3.532657
C	-2.488090	-3.587811	2.304824
H	-3.424397	-3.553253	2.865041
H	-1.719117	-3.968626	2.984196
H	-2.612100	-4.317798	1.503360
C	-2.951733	-2.759786	-1.083081
H	-3.231022	-3.754280	-0.716869
C	-1.490396	-2.820719	-1.504469
H	-1.169159	-1.853523	-1.912110
H	-1.315722	-3.543114	-2.307356
H	-0.829078	-3.105007	-0.682728
C	-3.811607	-2.436436	-2.300938
H	-4.875357	-2.406261	-2.061657
H	-3.673404	-3.173932	-3.098211
H	-3.540483	-1.460781	-2.709752
C	-5.093767	-1.666522	0.782769
H	-5.578059	-1.097224	-0.020034
C	-5.450631	-0.991447	2.104078
H	-5.045071	-1.549395	2.951700
H	-6.535101	-0.941015	2.243902
H	-5.060942	0.023539	2.163515
C	-5.664597	-3.083955	0.741584
H	-5.497501	-3.573963	-0.218083
H	-6.744900	-3.070148	0.917939
H	-5.223358	-3.718098	1.512376
Si	3.244761	-1.536517	-0.360082
Si	3.244827	1.536479	0.360107
N	2.660373	-0.000006	0.000013
C	2.127793	-2.204659	-1.771621
H	1.147134	-2.331815	-1.285368

C	1.928511	-1.216843	-2.917807
H	1.736652	-0.202383	-2.556504
H	1.106675	-1.514844	-3.576541
H	2.827118	-1.143834	-3.532629
C	2.487914	-3.587811	-2.304804
H	3.424194	-3.553257	-2.865066
H	1.718903	-3.968607	-2.984143
H	2.611954	-4.317810	-1.503355
C	2.951654	-2.759805	1.083117
H	3.230885	-3.754315	0.716902
C	1.490325	-2.820662	1.504549
H	1.169138	-1.853436	1.912156
H	1.315645	-3.543018	2.307468
H	0.828974	-3.104959	0.682837
C	3.811582	-2.436491	2.300947
H	4.875326	-2.406361	2.061630
H	3.673374	-3.173983	3.098223
H	3.540512	-1.460827	2.709772
C	5.093680	-1.666632	-0.782811
H	5.578024	-1.097342	0.019963
C	5.664458	-3.084086	-0.741631
H	5.497380	-3.574075	0.218049
H	6.744755	-3.070323	-0.918029
H	5.223164	-3.718223	-1.512397
C	5.450511	-0.991588	-2.104146
H	5.044884	-1.549526	-2.951741
H	6.534978	-0.941209	-2.244024
H	5.060869	0.023415	-2.163573
C	2.127930	2.204655	1.771681
H	1.147251	2.331803	1.285468
C	1.928690	1.216856	2.917888
H	1.736765	0.202406	2.556599
H	1.106904	1.514892	3.576669
H	2.827332	1.143823	3.532656
C	2.488096	3.587810	2.304825
H	3.424403	3.553251	2.865041
H	1.719123	3.968627	2.984197
H	2.612106	4.317797	1.503360
C	2.951733	2.759785	-1.083081
H	3.231024	3.754279	-0.716869
C	1.490397	2.820719	-1.504469
H	1.169158	1.853524	-1.912111
H	1.315722	3.543115	-2.307355
H	0.829079	3.105007	-0.682727
C	3.811607	2.436435	-2.300939
H	4.875357	2.406260	-2.061658
H	3.673404	3.173931	-3.098211
H	3.540482	1.460781	-2.709753
C	5.093767	1.666519	0.782768

H	5.578059	1.097222	-0.020036
C	5.450634	0.991444	2.104077
H	5.045075	1.549393	2.951699
H	6.535105	0.941011	2.243899
H	5.060944	-0.023540	2.163514
C	5.664599	3.083952	0.741583
H	5.497502	3.573961	-0.218084
H	6.744903	3.070144	0.917935
H	5.223362	3.718095	1.512375

64

N*H

H	0.000000	0.000000	1.707097
Si	1.644386	0.146301	0.149334
Si	-1.644387	-0.146301	0.149334
N	0.000000	0.000000	0.691681
C	2.670377	-1.056075	1.200013
H	2.588961	-0.636055	2.212027
C	2.111854	-2.475546	1.265897
H	1.063332	-2.482295	1.563786
H	2.668779	-3.081102	1.987473
H	2.179809	-2.980005	0.300624
C	4.153756	-1.069465	0.833580
H	4.312452	-1.537493	-0.140435
H	4.733452	-1.643612	1.562462
H	4.581671	-0.066137	0.790121
C	2.289339	1.889318	0.532363
H	3.344823	1.902571	0.235549
C	2.219557	2.209154	2.025111
H	1.191979	2.147820	2.394721
H	2.569192	3.226254	2.226078
H	2.828573	1.530288	2.624138
C	1.557265	2.962378	-0.270349
H	1.625268	2.796905	-1.346982
H	1.965261	3.956572	-0.064440
H	0.498397	2.986145	-0.007797
C	1.773720	-0.141265	-1.714600
H	0.965805	0.461202	-2.142744
C	3.085478	0.380580	-2.305428
H	3.246984	1.437941	-2.095033
H	3.089848	0.256975	-3.392409
H	3.946271	-0.165739	-1.916364
C	1.551047	-1.592952	-2.134492
H	2.387748	-2.220848	-1.821676
H	1.475249	-1.677163	-3.222761
H	0.645955	-2.019600	-1.704005
C	-2.670377	1.056076	1.200012
H	-2.588961	0.636055	2.212027
C	-2.111854	2.475546	1.265896

H	-1.063332	2.482295	1.563785
H	-2.668778	3.081102	1.987472
H	-2.179809	2.980006	0.300623
C	-4.153756	1.069465	0.833579
H	-4.312451	1.537492	-0.140435
H	-4.733453	1.643612	1.562462
H	-4.581670	0.066136	0.790120
C	-2.289339	-1.889317	0.532363
H	-3.344823	-1.902571	0.235550
C	-2.219557	-2.209153	2.025111
H	-1.191979	-2.147820	2.394720
H	-2.569192	-3.226254	2.226080
H	-2.828573	-1.530288	2.624138
C	-1.557264	-2.962378	-0.270349
H	-1.625268	-2.796906	-1.346982
H	-1.965261	-3.956572	-0.064439
H	-0.498398	-2.986145	-0.007797
C	-1.773720	0.141265	-1.714601
H	-0.965804	-0.461202	-2.142744
C	-1.551046	1.592951	-2.134491
H	-2.387747	2.220848	-1.821677
H	-1.475248	1.677162	-3.222762
H	-0.645954	2.019600	-1.704005
C	-3.085478	-0.380580	-2.305428
H	-3.246984	-1.437942	-2.095032
H	-3.089847	-0.256976	-3.392409
H	-3.946271	0.165738	-1.916363

129

Ba1

Ba	-0.019741	0.115043	-0.397164
Si	3.191925	1.363738	0.760875
Si	-2.911791	-1.306305	1.109884
Si	-3.410263	1.138425	-0.846934
Si	2.969502	-1.442477	-0.801354
N	-2.613446	-0.103360	-0.034462
N	2.597422	-0.044187	0.053562
C	4.045336	0.979565	2.418394
C	3.044021	0.371089	3.399117
C	4.868490	2.084783	3.073738
C	-1.293575	-1.484807	2.144474
C	-1.311002	-2.591260	3.196852
C	-0.803289	-0.175948	2.767811
C	4.793099	-1.981402	-0.948732
C	5.602370	-1.704545	0.315782
C	4.997998	-3.440698	-1.359794
C	1.922554	-2.850661	-0.023366
C	1.632458	-4.089523	-0.864460
C	2.439882	-3.229395	1.362489

C	-4.987407	0.737649	-1.826702
C	-6.088673	0.198720	-0.915550
C	-4.775465	-0.177495	-3.030884
C	-3.881429	2.534666	0.363982
C	-2.639907	3.202988	0.946793
C	-4.883137	3.581127	-0.116740
C	-2.130488	1.853593	-2.103753
C	-1.546371	0.796974	-3.056610
C	-2.630475	3.051978	-2.908098
C	-3.113476	-3.014658	0.288267
C	-4.418607	-3.164719	-0.485705
C	-1.932625	-3.294894	-0.636731
C	2.257570	-1.219344	-2.589840
C	2.477993	0.205209	-3.093061
C	2.752189	-2.204627	-3.646904
C	-4.387740	-0.995947	2.268576
C	-4.931209	-2.241312	2.968657
C	-4.118959	0.102405	3.297441
C	1.647294	2.479857	1.096312
C	1.132618	3.131236	-0.191269
C	1.755054	3.547291	2.184486
C	4.295667	2.387934	-0.415821
C	4.620342	3.816988	0.017027
C	5.577551	1.664378	-0.814309
H	1.410358	3.252170	-3.737523
H	0.824627	3.676024	-3.559730
H	4.738847	0.175504	2.153913
H	-0.533169	-1.822249	1.419592
H	5.206868	-1.356277	-1.751126
H	0.940017	-2.380511	0.155580
H	-5.326785	1.708555	-2.208677
H	-4.362544	1.979310	1.177407
H	-1.315383	2.259487	-1.482764
H	-3.104325	-3.763333	1.088760
H	1.166255	-1.392021	-2.517848
H	-5.167972	-0.628196	1.592823
H	0.884441	1.785138	1.483981
H	3.677106	2.455421	-1.320656
H	-0.521853	1.027472	-3.371745
H	-1.582903	-0.222196	-2.653462
H	-2.135416	0.747201	-3.971246
H	-3.523664	2.792491	-3.480063
H	-2.885704	3.891895	-2.263272
H	-1.876467	3.400602	-3.620351
H	-5.731853	-0.507441	-3.448805
H	-4.232430	0.326521	-3.830794
H	-4.209626	-1.073540	-2.769053
H	-7.031440	0.087628	-1.460309
H	-5.826717	-0.783046	-0.521726

H	-6.276095	0.853722	-0.062206
H	-2.883633	3.853602	1.792162
H	-1.916190	2.467103	1.307923
H	-2.140327	3.826268	0.199972
H	-5.139342	4.276270	0.689428
H	-4.488190	4.180139	-0.939288
H	-5.812675	3.128614	-0.463582
H	-5.046535	0.432145	3.775383
H	-3.465354	-0.263186	4.092517
H	-3.641595	0.979634	2.859319
H	-5.827075	-2.001731	3.550413
H	-5.197674	-3.032416	2.267887
H	-4.200735	-2.658519	3.664399
H	-2.036793	-4.252631	-1.155444
H	-1.865265	-2.520789	-1.409995
H	-0.983003	-3.329645	-0.100334
H	-4.512722	-4.166945	-0.915951
H	-5.297792	-2.991212	0.136401
H	-4.460431	-2.453256	-1.310361
H	0.270907	-0.184777	2.968443
H	-1.043853	0.703942	2.160442
H	-1.302315	0.004951	3.719105
H	-0.328693	-2.712180	3.662769
H	-2.020816	-2.357177	3.991939
H	-1.596275	-3.553874	2.771387
H	3.369648	-3.796483	1.288049
H	2.640354	-2.345157	1.968259
H	1.721311	-3.855267	1.900307
H	0.987395	-4.787591	-0.322052
H	1.125344	-3.841034	-1.797669
H	2.546395	-4.626271	-1.117189
H	6.059187	-3.639314	-1.538997
H	4.678848	-4.117981	-0.565629
H	4.456425	-3.714075	-2.263401
H	6.648419	-1.995916	0.177558
H	5.587456	-0.652976	0.586784
H	5.216412	-2.268806	1.166883
H	5.185922	4.336329	-0.763606
H	3.728742	4.410945	0.216383
H	5.229558	3.837953	0.921089
H	6.130916	2.231668	-1.569832
H	6.245781	1.533569	0.040641
H	5.373510	0.677707	-1.227155
H	0.814773	4.098715	2.285926
H	1.986751	3.112259	3.155059
H	2.535634	4.273592	1.957428
H	0.107803	3.501273	-0.093007
H	1.752138	3.989142	-0.452387
H	1.189554	2.487932	-1.077742

H	5.295161	1.740387	4.021637
H	5.699365	2.397172	2.440309
H	4.273415	2.972552	3.293273
H	3.547150	-0.035365	4.281967
H	2.320675	1.108494	3.754960
H	2.485096	-0.440460	2.930867
H	2.265705	-2.026110	-4.611004
H	3.826790	-2.088722	-3.802346
H	2.568410	-3.242186	-3.372108
H	1.986558	0.378596	-4.055224
H	2.135672	0.960549	-2.381896
H	3.544456	0.400159	-3.230081

129

Ba2*

H	1.471647	1.056599	-2.280624
H	2.126041	0.696146	-1.491118
Si	3.164342	1.619940	0.479570
Si	-2.825107	-1.535386	0.839953
N	-2.722101	-0.107107	-0.043977
N	2.652006	0.239988	-0.424074
C	4.069493	1.099979	2.061588
C	3.103254	0.539090	3.104963
C	4.992018	2.154488	2.668714
H	4.703477	0.269583	1.740095
C	-1.153836	-1.742896	1.786696
H	-0.391815	-1.909705	1.006072
C	-1.059672	-2.993836	2.656937
C	-0.742244	-0.514954	2.598609
Si	-3.637525	1.213658	-0.550112
Si	3.138632	-1.403866	-0.660705
C	4.989796	-1.703771	-1.007752
H	5.157792	-1.125643	-1.923593
C	6.022985	-1.216602	0.004251
C	5.259456	-3.171992	-1.348952
C	2.533876	-2.437750	0.843719
H	1.762384	-1.797820	1.293746
C	1.886140	-3.790435	0.556971
C	3.613514	-2.634221	1.909119
C	-5.295867	0.911464	-1.427128
C	-6.260358	0.108966	-0.557003
H	-5.720005	1.916273	-1.547218
C	-5.187502	0.296327	-2.820186
C	-4.014598	2.357975	0.930651
C	-2.736847	3.000807	1.463778
C	-5.112661	3.402847	0.750680
H	-4.364353	1.651826	1.692355
C	-2.512804	2.187374	-1.769288
H	-1.652584	2.506326	-1.159562

C	-1.991265	1.333711	-2.937795
C	-3.109275	3.490544	-2.296603
C	-2.857193	-3.033035	-0.341412
H	-2.684769	-3.939010	0.250498
C	-4.189733	-3.176193	-1.070112
C	-1.733520	-2.912186	-1.367952
C	2.243084	-1.856903	-2.307917
H	1.278823	-1.326428	-2.271681
C	2.999336	-1.201080	-3.469344
C	1.911025	-3.302617	-2.672105
C	-4.268665	-1.670064	2.067690
H	-5.118321	-1.284466	1.494883
C	-4.620065	-3.089845	2.512410
C	-4.097002	-0.766870	3.289025
C	1.583995	2.596889	0.982197
C	0.925945	3.285573	-0.218316
H	0.900253	1.834430	1.393893
C	1.738866	3.624653	2.104955
C	4.144603	2.742172	-0.694845
H	3.447077	2.823785	-1.540238
C	4.453657	4.157381	-0.211984
C	5.406967	2.075817	-1.233557
Ba	-0.181962	0.255379	-0.593434
H	-0.981625	1.618811	-3.255077
H	-2.006605	0.258315	-2.723341
H	-2.633495	1.449420	-3.809893
H	-4.037630	3.303249	-2.840532
H	-3.336728	4.185894	-1.489141
H	-2.422765	3.995755	-2.982778
H	-6.176289	0.040995	-3.214761
H	-4.723081	0.979883	-3.530864
H	-4.594761	-0.620407	-2.816752
H	-7.261231	0.080329	-0.999071
H	-5.923825	-0.923623	-0.456277
H	-6.355228	0.522760	0.449342
H	-2.898539	3.494135	2.427116
H	-1.944919	2.261555	1.612434
H	-2.358502	3.761166	0.775253
H	-5.280492	3.959126	1.678812
H	-4.860942	4.135197	-0.018852
H	-6.063106	2.949407	0.466996
H	-5.035810	-0.666999	3.842235
H	-3.363778	-1.184837	3.982578
H	-3.760711	0.235772	3.022382
H	-5.527809	-3.091228	3.124192
H	-4.791909	-3.761853	1.671491
H	-3.826162	-3.528393	3.119231
H	-1.698582	-3.767542	-2.048648
H	-1.886149	-2.021813	-1.987629

H	-0.745057	-2.852401	-0.901836
H	-4.188327	-4.037171	-1.746136
H	-5.029405	-3.297719	-0.384768
H	-4.390528	-2.289480	-1.672552
H	0.321865	-0.517906	2.849564
H	-0.985125	0.430942	2.103339
H	-1.290161	-0.480041	3.539717
H	-0.050232	-3.120229	3.059452
H	-1.740486	-2.925637	3.506430
H	-1.309457	-3.898408	2.101983
H	4.370335	-3.342193	1.567715
H	4.123739	-1.711496	2.174440
H	3.176480	-3.044760	2.824260
H	1.643298	-4.300864	1.493381
H	0.958690	-3.700411	-0.006171
H	2.549217	-4.446372	-0.009258
H	6.317088	-3.322276	-1.585796
H	5.022064	-3.833369	-0.512890
H	4.684544	-3.511013	-2.209241
H	7.031437	-1.348259	-0.400431
H	5.909386	-0.160758	0.236477
H	5.978413	-1.774811	0.938764
H	4.954595	4.729566	-0.999045
H	3.556614	4.710662	0.065226
H	5.116175	4.155748	0.654828
H	5.852444	2.675595	-2.032986
H	6.166224	1.966031	-0.455290
H	5.197875	1.086497	-1.639309
H	0.778335	4.101732	2.321476
H	2.101943	3.184153	3.030916
H	2.436049	4.414062	1.823738
H	-0.091099	3.617264	0.011075
H	1.491129	4.178878	-0.486605
H	0.922454	2.686186	-1.138516
H	5.480268	1.768808	3.569013
H	5.777115	2.451071	1.972375
H	4.450853	3.057431	2.956431
H	3.645695	0.066694	3.928968
H	2.474006	1.315841	3.539718
H	2.442502	-0.214769	2.677305
H	1.498954	-3.343720	-3.685078
H	2.791331	-3.945038	-2.660757
H	1.177577	-3.750212	-2.007268
H	2.391881	-1.218920	-4.378298
H	3.247836	-0.159951	-3.264780
H	3.922809	-1.743010	-3.682952

	129		
Ba3			
H	-1.058769	-0.376374	-2.397818
H	-2.294423	-0.320610	-1.242790
N	2.786758	-0.036345	0.055435
Si	3.167385	0.318240	1.657134
Ba	0.225630	-0.054862	-0.391436
Si	-3.314301	1.538347	-0.426159
Si	-3.485494	-1.600738	0.272985
N	-2.742371	-0.118323	-0.310682
Si	3.540423	-0.420192	-1.407098
C	3.827309	-2.305845	-1.465916
H	4.419512	-2.483231	-0.558886
C	4.617922	-2.868609	-2.643194
C	2.507471	-3.053127	-1.293401
C	5.176077	0.412771	-1.903837
C	5.090853	1.933038	-2.012345
H	5.349835	0.021430	-2.915010
C	6.375825	0.008836	-1.053695
C	4.102155	-1.033946	2.623246
C	3.481881	-2.424386	2.482812
H	4.053632	-0.738543	3.679887
C	5.574736	-1.119295	2.226180
C	4.167400	1.935376	1.750785
H	4.973894	1.739458	1.033599
C	4.826497	2.308437	3.076630
C	3.349220	3.098650	1.191983
C	2.281383	0.040446	-2.791783
H	1.430209	-0.658001	-2.703402
C	2.783544	-0.173203	-4.219447
C	1.713393	1.463221	-2.656268
C	1.500954	0.622320	2.592250
H	0.941562	1.317359	1.945051
C	1.640039	1.350759	3.927973
C	0.648064	-0.627731	2.825620
C	-3.014248	-1.879846	2.097944
C	-5.361427	-1.716952	-0.015540
C	-2.721244	-3.010575	-0.737722
H	-3.078196	-3.922187	-0.246568
C	-3.189936	-3.031363	-2.193330
C	-1.192241	-3.028889	-0.704188
C	-5.858680	-3.139195	0.262769
C	-6.278624	-0.721929	0.694332
H	-5.457738	-1.538357	-1.094041
C	-2.752463	-3.344953	2.453762
H	-2.063023	-1.345792	2.206553
C	-3.992660	-1.289084	3.112830
C	-4.723517	1.511315	-1.694753
C	-3.832534	2.138747	1.294752

C	-1.845882	2.558071	-1.093847
C	-5.647167	2.722266	-1.794419
C	-4.197994	1.092662	-3.072120
H	-5.333411	0.676358	-1.337874
C	-4.224501	3.617125	1.330005
H	-4.724481	1.554251	1.546153
C	-2.782978	1.858284	2.375294
C	-2.245659	3.801829	-1.889803
C	-0.806898	2.986507	-0.052327
H	-1.385901	1.862535	-1.815654
H	2.251556	2.159682	-3.298876
H	0.660469	1.500735	-2.962209
H	1.819208	1.872262	-1.644272
H	3.641183	0.468406	-4.432567
H	3.091777	-1.202716	-4.394947
H	2.003216	0.065144	-4.948053
H	6.050142	2.357880	-2.325655
H	4.341676	2.252739	-2.736392
H	4.832220	2.386635	-1.054666
H	7.305402	0.402561	-1.477316
H	6.292820	0.403151	-0.040711
H	6.484081	-1.074202	-0.973321
H	2.657208	-4.132480	-1.190836
H	1.982079	-2.720189	-0.393503
H	1.847217	-2.904076	-2.152832
H	4.863765	-3.923623	-2.484233
H	4.048119	-2.814495	-3.572522
H	5.555419	-2.334465	-2.804427
H	4.103109	-3.179292	2.975786
H	2.485846	-2.495046	2.915829
H	3.402593	-2.704876	1.431139
H	6.112737	-1.823274	2.868899
H	5.673404	-1.480491	1.201944
H	6.090000	-0.160188	2.282978
H	3.960794	3.996038	1.055093
H	2.912608	2.843440	0.224336
H	2.529792	3.369661	1.863495
H	5.517864	3.147223	2.946032
H	4.094614	2.614989	3.824386
H	5.394158	1.480960	3.504150
H	-4.275903	-3.079675	-2.279899
H	-2.841305	-2.139828	-2.719668
H	-2.779810	-3.897725	-2.720823
H	-0.790049	-3.997512	-1.009930
H	-0.827213	-2.300428	-1.439518
H	-0.780166	-2.823926	0.290724
H	-6.918102	-3.232758	0.007031
H	-5.316385	-3.894643	-0.305743
H	-5.762562	-3.389612	1.321788

H	-7.299731	-0.811402	0.311809
H	-6.320025	-0.910962	1.766274
H	-5.975709	0.314194	0.557119
H	-5.025132	0.783352	-3.717177
H	-3.688278	1.913329	-3.578498
H	-3.485794	0.266716	-3.019466
H	-6.394258	2.567262	-2.578732
H	-6.189676	2.897914	-0.864408
H	-5.107241	3.637715	-2.041459
H	0.140714	3.270534	-0.520116
H	-1.156143	3.861144	0.497874
H	-0.597358	2.250088	0.728683
H	-2.774280	4.521948	-1.261636
H	-1.358170	4.305055	-2.284620
H	-2.888499	3.563774	-2.733736
H	-3.223920	1.922023	3.373280
H	-2.337181	0.868376	2.277960
H	-1.978325	2.591978	2.340001
H	-4.569838	3.902278	2.327839
H	-3.370283	4.255138	1.093758
H	-5.017480	3.860087	0.624673
H	-3.558828	-1.294268	4.116983
H	-4.282377	-0.265261	2.883899
H	-4.905337	-1.886117	3.154946
H	-2.449359	-3.434397	3.501170
H	-3.651578	-3.951425	2.326140
H	-1.967720	-3.795143	1.846718
H	0.671653	1.437553	4.431853
H	2.310270	0.812259	4.601802
H	2.035490	2.356764	3.803898
H	-0.394166	-0.374356	3.043179
H	0.667429	-1.360760	2.011190
H	1.020263	-1.174321	3.692613

65
Ba4 (N*BaH)

H	-2.235228	-3.544243	0.289911
Ba	-0.211724	-2.484745	-0.015328
Si	-1.526440	0.733459	-0.139069
Si	1.635272	0.493519	0.143977
N	0.017351	0.046584	-0.005637
C	-2.659547	-0.271720	1.024577
H	-2.702629	-1.287905	0.590164
C	-2.099919	-0.438336	2.435741
H	-1.039129	-0.706168	2.428202
H	-2.642823	-1.208958	2.990320
H	-2.168251	0.490547	3.005107
C	-4.111041	0.199469	1.078661
H	-4.192288	1.159105	1.594008

H	-4.736654	-0.515168	1.621316
H	-4.542084	0.322030	0.083676
C	-2.233740	0.427803	-1.888609
H	-3.288505	0.723813	-1.856539
C	-2.182461	-1.047031	-2.281615
H	-1.139991	-1.342870	-2.466187
H	-2.702184	-1.237913	-3.225206
H	-2.630934	-1.718575	-1.539912
C	-1.530742	1.256939	-2.959370
H	-1.572115	2.327121	-2.752460
H	-1.970917	1.095142	-3.948810
H	-0.476457	0.977708	-3.023917
C	-1.621642	2.604362	0.175054
H	-0.806922	3.012210	-0.435087
C	-2.917951	3.263404	-0.296590
H	-3.130656	3.062427	-1.347074
H	-2.866682	4.350070	-0.174250
H	-3.776033	2.915910	0.280635
C	-1.340266	2.975131	1.629197
H	-2.163869	2.665652	2.276571
H	-1.227092	4.057363	1.748535
H	-0.434109	2.502651	2.007506
C	2.659888	-0.633052	-1.034746
H	2.595930	-1.639148	-0.583858
C	2.071621	-0.730240	-2.440048
H	0.992636	-0.905225	-2.424154
H	2.546223	-1.520738	-3.030628
H	2.208049	0.205064	-2.984909
C	4.149328	-0.304973	-1.098869
H	4.311043	0.651209	-1.599553
H	4.703602	-1.060922	-1.664162
H	4.600406	-0.236265	-0.107769
C	2.315551	0.075981	1.884528
H	3.395470	0.261477	1.853625
C	2.114791	-1.386125	2.275619
H	1.047745	-1.595822	2.425066
H	2.590411	-1.623367	3.231898
H	2.529697	-2.084383	1.540557
C	1.704426	0.966373	2.963475
H	1.866470	2.026132	2.764136
H	2.123479	0.749161	3.951291
H	0.625889	0.807817	3.023662
C	2.004394	2.333009	-0.152102
H	1.235917	2.834917	0.447307
C	1.795123	2.752905	-1.605114
H	2.580877	2.348142	-2.247633
H	1.825806	3.841816	-1.710496
H	0.839198	2.408758	-1.998510
C	3.362262	2.822141	0.351200

H	3.529161	2.585692	1.402525
H	3.443824	3.908351	0.243690
H	4.184697	2.383057	-0.215444

71
Ba5

Ba	1.571188	-1.548311	-0.270757
Si	-1.840663	-0.698745	-0.062459
Si	0.268953	1.670613	0.141062
N	-0.364306	0.117227	-0.010246
C	-1.686066	-2.224536	1.084095
H	-0.891385	-2.849406	0.637606
C	-1.211207	-1.876247	2.493028
H	-0.317644	-1.251942	2.477266
H	-0.970939	-2.776056	3.064746
H	-1.979315	-1.326923	3.039910
C	-2.927092	-3.112747	1.136594
H	-3.743978	-2.601388	1.649656
H	-2.730105	-4.036665	1.688456
H	-3.286227	-3.389339	0.143567
C	-2.155522	-1.436322	-1.804979
H	-3.069092	-2.036121	-1.725447
C	-1.044114	-2.367761	-2.282131
H	-0.139733	-1.788187	-2.511883
H	-1.304658	-2.873766	-3.216669
H	-0.807154	-3.153942	-1.557307
C	-2.382710	-0.352711	-2.854675
H	-3.211247	0.306548	-2.593453
H	-2.599609	-0.780018	-3.839368
H	-1.491870	0.270989	-2.957512
C	-3.372175	0.358958	0.325517
H	-3.213019	1.253487	-0.288936
C	-4.708123	-0.250556	-0.099051
H	-4.729946	-0.518428	-1.155844
H	-5.526691	0.454899	0.076151
H	-4.936542	-1.152922	0.470309
C	-3.441206	0.811082	1.782165
H	-3.698529	-0.023443	2.438148
H	-4.210294	1.577440	1.921404
H	-2.495195	1.221344	2.133304
C	1.743864	1.777568	-1.096502
H	2.493969	1.068268	-0.702228
C	1.389515	1.325148	-2.511222
H	0.808108	0.398926	-2.512450
H	2.280302	1.182374	-3.132326
H	0.765624	2.067289	-3.010885
C	2.457885	3.126395	-1.126566
H	1.812851	3.892150	-1.560990
H	3.366669	3.088280	-1.735830

H	2.741277	3.463753	-0.128595
C	1.077700	1.939886	1.848895
H	1.559751	2.924144	1.820341
C	2.156786	0.905721	2.155627
H	1.736014	-0.103663	2.240779
H	2.639160	1.096134	3.119129
H	2.953946	0.896642	1.407265
C	0.044183	1.946997	2.971984
H	-0.723136	2.708430	2.823281
H	0.510790	2.135050	3.944166
H	-0.459504	0.980786	3.034270
C	-0.967705	3.090410	-0.111509
H	-1.814696	2.784950	0.514219
C	-1.477335	3.199345	-1.546374
H	-0.702563	3.593041	-2.208855
H	-2.330174	3.882164	-1.612939
H	-1.792049	2.235403	-1.945252
C	-0.502612	4.455734	0.393419
H	-0.199154	4.430770	1.440354
H	-1.304864	5.194938	0.302367
H	0.344785	4.831789	-0.182434
C	4.882789	-0.859286	-0.144111
C	4.743048	-1.643766	0.923813
H	4.755877	0.216482	-0.083671
H	5.182472	-1.257334	-1.108469
H	4.469920	-1.251614	1.895179
H	4.896035	-2.714253	0.879805
H	2.209923	-2.872252	1.515245

71

Ba6*

Ba	1.683047	-1.425103	-0.316839
Si	-1.764715	-0.797393	-0.058996
Si	0.171608	1.714899	0.146309
N	-0.354065	0.122436	-0.012407
C	-1.481010	-2.332206	1.057667
H	-0.685942	-2.915339	0.557897
C	-0.955991	-1.979031	2.447616
H	-0.092685	-1.313638	2.403458
H	-0.657666	-2.872664	3.002346
H	-1.720188	-1.465259	3.033128
C	-2.677129	-3.276077	1.157249
H	-3.489440	-2.806929	1.715541
H	-2.414786	-4.197423	1.686065
H	-3.070956	-3.555643	0.178464
C	-2.063925	-1.532477	-1.805499
H	-2.931305	-2.196249	-1.715056
C	-0.902596	-2.375510	-2.326320
H	-0.043927	-1.732599	-2.560819

H	-1.150130	-2.878377	-3.266078
H	-0.597701	-3.159667	-1.624909
C	-2.392103	-0.451415	-2.831157
H	-3.256955	0.144985	-2.538636
H	-2.604402	-0.878251	-3.817000
H	-1.549233	0.233796	-2.945549
C	-3.362466	0.134049	0.381330
H	-3.280063	1.051306	-0.214588
C	-4.661005	-0.558929	-0.030680
H	-4.685786	-0.805951	-1.092509
H	-5.522742	0.084036	0.175195
H	-4.815052	-1.486431	0.522968
C	-3.428710	0.547674	1.849396
H	-3.600565	-0.318550	2.492431
H	-4.252354	1.246543	2.026307
H	-2.509012	1.025401	2.184539
C	1.612695	1.930639	-1.112476
H	2.413267	1.271228	-0.732436
C	1.269097	1.460572	-2.524236
H	0.749711	0.497982	-2.521302
H	2.159087	1.378134	-3.157059
H	0.590839	2.161068	-3.013315
C	2.231576	3.325530	-1.145600
H	1.528003	4.047649	-1.564023
H	3.130809	3.352900	-1.769137
H	2.508144	3.675423	-0.149989
C	0.982608	2.027572	1.845411
H	1.378122	3.049780	1.822364
C	2.151052	1.087348	2.123777
H	1.822640	0.042741	2.189213
H	2.620106	1.299601	3.089379
H	2.939920	1.161606	1.371709
C	-0.031338	1.933976	2.982132
H	-0.862859	2.628923	2.853904
H	0.431684	2.149354	3.950418
H	-0.450192	0.927539	3.038180
C	-1.169040	3.044702	-0.069541
H	-1.982858	2.674661	0.565663
C	-1.706384	3.130685	-1.495762
H	-0.967937	3.576673	-2.166518
H	-2.602035	3.758009	-1.545093
H	-1.964703	2.151057	-1.896963
C	-0.792980	4.434574	0.442576
H	-0.477317	4.421508	1.486157
H	-1.644581	5.118233	0.366212
H	0.020930	4.871887	-0.138328
C	4.607346	-0.890845	-0.119658
C	4.561763	-1.634360	1.010188
H	4.495304	0.187723	-0.094374

H	4.893259	-1.326796	-1.072210
H	4.438581	-1.181523	1.983370
H	4.850994	-2.674935	1.020045
H	2.694561	-2.478694	1.469919

71

Ba7

Ba	1.264519	-1.868568	0.116728
Si	-1.937687	-0.417856	-0.182163
Si	0.544644	1.540781	0.160366
N	-0.352793	0.113841	0.018548
C	-2.216271	-1.899549	1.015368
H	-1.593966	-2.711061	0.599871
C	-1.716904	-1.630152	2.432474
H	-0.719186	-1.183482	2.438181
H	-1.697291	-2.540072	3.041390
H	-2.361759	-0.913917	2.943978
C	-3.642692	-2.442211	1.043038
H	-4.319171	-1.724073	1.509913
H	-3.709323	-3.368968	1.621939
H	-4.025080	-2.649139	0.042215
C	-2.205818	-1.174270	-1.921471
H	-3.207541	-1.619612	-1.920671
C	-1.212472	-2.284526	-2.255207
H	-0.203845	-1.867365	-2.371087
H	-1.438810	-2.762379	-3.213139
H	-1.197180	-3.081288	-1.503974
C	-2.150762	-0.113577	-3.017913
H	-2.881358	0.680577	-2.860795
H	-2.339631	-0.544224	-4.006665
H	-1.164269	0.353478	-3.047569
C	-3.276763	0.913145	0.036338
H	-2.894077	1.746047	-0.565113
C	-4.658046	0.556014	-0.511891
H	-4.626357	0.247445	-1.557345
H	-5.333131	1.415117	-0.445140
H	-5.118396	-0.256002	0.052792
C	-3.389376	1.406467	1.477099
H	-3.841904	0.646072	2.118292
H	-4.022377	2.296980	1.542333
H	-2.418230	1.658343	1.902161
C	2.089917	1.316792	-0.941104
H	2.663928	0.509988	-0.454529
C	1.779944	0.825359	-2.352873
H	1.045855	0.014273	-2.353673
H	2.682741	0.471909	-2.860013
H	1.347473	1.618186	-2.966082
C	3.033481	2.515597	-0.988180
H	2.584687	3.345244	-1.538865

H	3.970946	2.260488	-1.490608
H	3.286745	2.879773	0.008756
C	1.245931	1.710907	1.930957
H	1.956934	2.544655	1.907695
C	2.012422	0.465801	2.372571
H	1.306723	-0.355435	2.559376
H	2.530452	0.618867	3.323893
H	2.771910	0.138908	1.653743
C	0.164342	2.025841	2.960125
H	-0.389512	2.933671	2.717366
H	0.585300	2.154438	3.962761
H	-0.557901	1.207841	3.013579
C	-0.396885	3.146620	-0.218591
H	-1.325854	3.046503	0.355045
C	-0.775082	3.269622	-1.692732
H	0.108591	3.454664	-2.307802
H	-1.462571	4.105160	-1.858757
H	-1.252236	2.366312	-2.071946
C	0.297865	4.422382	0.257342
H	0.539740	4.394583	1.320329
H	-0.339455	5.296437	0.088819
H	1.229522	4.596062	-0.283131
C	4.976066	-0.746207	-0.290178
C	3.893519	-1.793592	-0.065122
H	6.005407	-1.131254	-0.288266
H	4.836771	-0.236959	-1.248964
H	4.107119	-2.337110	0.877987
H	3.985826	-2.577220	-0.845132
H	4.938357	0.036745	0.475114

73

Ba8

Ba	2.092893	-0.449952	-0.617653
Si	-0.552022	1.705779	0.238652
Si	-1.258010	-1.375409	-0.080594
N	-0.368571	0.056784	-0.050244
C	0.485355	2.625587	-1.098473
H	1.533113	2.368339	-0.861704
C	0.210586	2.147809	-2.521634
H	0.200002	1.057207	-2.592906
H	0.946285	2.537341	-3.234075
H	-0.770648	2.481808	-2.861711
C	0.419558	4.148741	-1.022032
H	-0.571733	4.504862	-1.308078
H	1.138050	4.617423	-1.702209
H	0.624403	4.519962	-0.016900
C	0.266742	2.214512	1.887908
H	0.233611	3.309036	1.939487
C	1.730028	1.788272	1.964225

H	1.821579	0.695638	1.977835
H	2.206348	2.127479	2.888999
H	2.333943	2.191138	1.144806
C	-0.485105	1.654548	3.092585
H	-1.528838	1.971480	3.113742
H	-0.025549	1.970348	4.034673
H	-0.474804	0.563248	3.075512
C	-2.343822	2.336579	0.290386
H	-2.837623	1.608716	0.944663
C	-2.531990	3.714629	0.924017
H	-2.094657	3.778375	1.920824
H	-3.595899	3.955307	1.016971
H	-2.078541	4.501620	0.319185
C	-3.040616	2.276676	-1.066763
H	-2.656622	3.048437	-1.738343
H	-4.117283	2.447252	-0.967651
H	-2.900721	1.314429	-1.558912
C	-0.244960	-2.699408	0.864769
H	0.710252	-2.787263	0.316257
C	0.122188	-2.276908	2.285209
H	0.561118	-1.279703	2.307492
H	0.845667	-2.965605	2.729435
H	-0.758899	-2.260150	2.928416
C	-0.854525	-4.099276	0.858524
H	-1.767461	-4.123888	1.456630
H	-0.167725	-4.832831	1.291741
H	-1.112519	-4.439178	-0.146116
C	-1.391205	-2.057258	-1.867667
H	-1.884604	-3.032753	-1.794983
C	-0.033726	-2.283438	-2.528120
H	0.446170	-1.318822	-2.741594
H	-0.126012	-2.779686	-3.499001
H	0.634498	-2.904605	-1.921886
C	-2.238150	-1.155419	-2.760461
H	-3.246062	-1.017426	-2.367311
H	-2.332592	-1.559907	-3.773789
H	-1.783654	-0.165699	-2.843373
C	-3.048413	-1.238173	0.545850
H	-3.418784	-0.347673	0.023635
C	-3.152186	-0.964042	2.044307
H	-2.907473	-1.857731	2.622301
H	-4.169736	-0.671528	2.322251
H	-2.478103	-0.171920	2.368474
C	-3.961945	-2.401494	0.160381
H	-3.976371	-2.584626	-0.914538
H	-4.992440	-2.203145	0.472189
H	-3.654898	-3.330080	0.644529
C	4.643475	-0.290091	1.082680
C	3.680607	-1.449270	1.222755

H	5.694602	-0.562119	0.942287
H	4.443448	0.380242	0.193499
H	3.339919	-1.576167	2.250111
H	4.131147	-2.393343	0.897244
H	4.599620	0.397597	1.929932
H	3.954036	1.463770	-2.104682
H	3.359467	1.574009	-2.546567

73
Ba9*

Ba	-1.318200	-1.794032	-0.019408
Si	1.926175	-0.510756	0.142769
Si	-0.412786	1.621489	-0.143811
N	0.378594	0.133966	-0.010267
C	2.027874	-2.032924	-1.033367
H	1.365464	-2.793035	-0.580548
C	1.497050	-1.748219	-2.436644
H	0.533217	-1.233213	-2.421364
H	1.391594	-2.662832	-3.028220
H	2.174340	-1.087242	-2.979560
C	3.403610	-2.691322	-1.104527
H	4.116568	-2.035578	-1.607233
H	3.372103	-3.626602	-1.672061
H	3.806218	-2.916948	-0.115801
C	2.213355	-1.259338	1.883515
H	3.184279	-1.766980	1.848471
C	1.169049	-2.300688	2.278483
H	0.193293	-1.821344	2.428654
H	1.407049	-2.777403	3.234005
H	1.075214	-3.108839	1.544673
C	2.272177	-0.183025	2.964059
H	3.040460	0.563701	2.760771
H	2.481085	-0.610897	3.949942
H	1.317317	0.342425	3.031135
C	3.354332	0.706099	-0.152706
H	3.059518	1.573661	0.449729
C	4.724792	0.251685	0.348379
H	4.712421	-0.033027	1.400941
H	5.460850	1.053946	0.235745
H	5.097301	-0.604400	-0.216108
C	3.446437	1.170224	-1.604294
H	3.812085	0.368516	-2.250435
H	4.143665	2.007385	-1.708316
H	2.481405	1.491473	-1.994339
C	-1.945098	1.517347	1.002612
H	-2.580913	0.751618	0.529261
C	-1.628977	1.022735	2.411738
H	-0.960907	0.156742	2.402212
H	-2.537208	0.752404	2.959290

H	-1.114167	1.790176	2.992271
C	-2.805829	2.776611	1.056112
H	-2.286556	3.579603	1.583258
H	-3.744679	2.594430	1.587797
H	-3.057451	3.145070	0.060488
C	-1.146018	1.850776	-1.890621
H	-1.764929	2.754813	-1.855219
C	-2.045243	0.688470	-2.298437
H	-1.454139	-0.221513	-2.455442
H	-2.543476	0.870147	-3.255021
H	-2.836633	0.472874	-1.576056
C	-0.066533	2.048548	-2.951070
H	0.585009	2.895596	-2.731065
H	-0.501991	2.215709	-3.941489
H	0.564341	1.159611	-3.020384
C	0.653163	3.153736	0.211750
H	1.558639	2.978035	-0.381421
C	1.074739	3.260783	1.674990
H	0.222980	3.517957	2.309146
H	1.826438	4.044015	1.815578
H	1.492685	2.327966	2.052198
C	0.049526	4.475339	-0.263186
H	-0.205108	4.460140	-1.323273
H	0.752126	5.300088	-0.106338
H	-0.860520	4.721277	0.286268
C	-5.171605	-0.675299	-0.112181
C	-4.055006	-1.565803	0.407015
H	-6.027105	-0.589243	0.566443
H	-4.813279	0.341192	-0.298675
H	-4.453818	-2.544177	0.709921
H	-3.638785	-1.118989	1.330787
H	-5.553983	-1.050876	-1.066631
H	-3.006469	-2.391466	-1.712229
H	-3.434706	-2.052624	-0.948163

73

Ba10

Ba	2.030042	-0.118967	0.867636
Si	-0.963414	1.585622	0.058839
Si	-0.891622	-1.577001	-0.287181
N	-0.387415	0.003301	0.048862
C	0.372812	2.693113	-0.767968
H	1.248520	2.612041	-0.099230
C	0.809447	2.178619	-2.137034
H	1.018316	1.106958	-2.120064
H	1.698551	2.701247	-2.504686
H	0.022125	2.324132	-2.877755
C	0.033671	4.179584	-0.835413
H	-0.782429	4.356241	-1.538406

H	0.887560	4.770864	-1.181460
H	-0.275559	4.579188	0.131721
C	-1.103638	2.267973	1.843594
H	-1.361080	3.329353	1.752098
C	0.199289	2.183783	2.634742
H	0.427772	1.137885	2.877824
H	0.125204	2.691403	3.601174
H	1.047413	2.634540	2.108232
C	-2.210032	1.572358	2.632404
H	-3.181887	1.662730	2.146130
H	-2.307657	1.984803	3.641978
H	-1.995333	0.506478	2.733354
C	-2.675738	1.835683	-0.727543
H	-3.269701	1.033667	-0.273486
C	-3.365773	3.157382	-0.390999
H	-3.441768	3.329427	0.683115
H	-4.382045	3.176152	-0.797357
H	-2.835125	4.008952	-0.820028
C	-2.683981	1.607374	-2.237356
H	-2.197689	2.433922	-2.760781
H	-3.706808	1.544134	-2.621694
H	-2.166696	0.690685	-2.518824
C	-0.179233	-2.671027	1.110822
H	0.918124	-2.659163	0.971852
C	-0.439899	-2.126645	2.514041
H	-0.268044	-1.046989	2.579330
H	0.190578	-2.621901	3.258273
H	-1.480010	-2.272782	2.811300
C	-0.563253	-4.147113	1.035027
H	-1.621053	-4.289057	1.266974
H	0.010414	-4.740427	1.753068
H	-0.382856	-4.568356	0.044739
C	-0.042712	-2.215124	-1.875613
H	-0.238958	-3.292115	-1.929340
C	1.470987	-2.022599	-1.823422
H	1.709955	-0.951877	-1.876392
H	1.971407	-2.471839	-2.686581
H	1.940501	-2.458443	-0.933293
C	-0.590626	-1.559401	-3.139893
H	-1.667704	-1.694568	-3.247253
H	-0.116168	-1.962550	-4.040676
H	-0.397114	-0.484441	-3.125907
C	-2.762661	-1.813707	-0.512615
H	-3.043871	-1.013463	-1.207385
C	-3.541949	-1.596331	0.782379
H	-3.364918	-2.413367	1.485654
H	-4.620197	-1.560520	0.596809
H	-3.259891	-0.669866	1.282299
C	-3.165682	-3.139856	-1.157275

H	-2.660452	-3.312195	-2.108184
H	-4.243293	-3.166317	-1.348496
H	-2.934104	-3.986176	-0.509059
C	4.426675	1.023381	-1.112108
C	4.845313	-0.433669	-1.252952
H	3.373392	1.188612	-1.365271
H	4.616123	1.411888	-0.105314
H	4.371726	-1.106292	-0.525687
H	5.919844	-0.539736	-1.101615
H	4.607968	-0.811515	-2.247499
H	4.984719	1.666475	-1.793324
H	3.114161	-2.112170	1.309387

127

CaN^{*}_{2}

N	-2.409251	-0.024389	-0.018263
Si	-2.975720	-1.158106	1.108000
Ca	-0.106756	0.014860	-0.009609
Si	2.959065	-1.128625	-0.951124
Si	2.853379	1.304608	0.945786
N	2.165764	0.052749	0.000161
Si	-3.013064	1.111344	-1.122853
C	-3.901578	2.575673	-0.286858
H	-4.613134	2.084685	0.386811
C	-4.709786	3.491705	-1.205854
C	-2.958576	3.396645	0.586775
C	-4.192658	0.446145	-2.455388
C	-3.650195	-0.762430	-3.213824
H	-4.315257	1.270189	-3.170426
C	-5.571840	0.116509	-1.884066
C	-4.206864	-0.538767	2.417853
C	-3.751675	0.699369	3.185253
H	-4.288338	-1.367620	3.133468
C	-5.599062	-0.298113	1.832871
C	-3.791973	-2.685413	0.308690
H	-4.528504	-2.245950	-0.374166
C	-4.552800	-3.617226	1.252456
C	-2.817811	-3.485535	-0.548961
C	-1.434069	1.818174	-1.967300
H	-0.833023	2.170462	-1.111435
C	-1.621337	3.059115	-2.835660
C	-0.610457	0.787226	-2.749681
C	-1.373109	-1.758870	1.992560
H	-0.743542	-2.125569	1.163965
C	-1.520713	-2.959245	2.923940
C	-0.610443	-0.648576	2.727424
C	2.773640	0.913919	2.814458
C	4.615378	1.852714	0.436040
C	1.809334	2.906188	0.794611

H	2.387815	3.645500	1.358346
C	1.707709	3.405511	-0.642811
C	0.437021	2.871081	1.455222
C	4.988739	3.280112	0.843473
C	5.717895	0.896317	0.889331
H	4.594355	1.837975	-0.657741
C	3.292466	-0.460319	3.218840
C	3.380407	1.997452	3.704310
H	1.695440	0.908184	3.017227
C	4.132963	-0.312098	-2.222928
C	3.907246	-2.315555	0.205128
C	1.713571	-2.153360	-2.000798
C	4.785368	-1.182661	-3.296198
C	3.427716	0.858570	-2.907011
H	4.943527	0.097659	-1.611734
C	5.117956	-3.054926	-0.355712
H	4.284331	-1.624007	0.964619
C	2.974533	-3.278192	0.940768
C	2.372213	-3.413466	-2.576197
C	0.415947	-2.586627	-1.317646
H	1.446405	-1.512102	-2.849439
H	-0.945165	0.750621	-3.786346
H	0.456608	1.029324	-2.773909
H	-0.730911	-0.243530	-2.398661
H	-2.309219	2.855770	-3.659464
H	-2.021213	3.897138	-2.268110
H	-0.671247	3.381684	-3.272367
H	-4.383511	-1.133351	-3.936976
H	-2.737723	-0.536467	-3.764581
H	-3.427543	-1.581516	-2.528830
H	-6.260247	-0.192297	-2.676775
H	-5.512771	-0.707399	-1.171913
H	-6.026594	0.958427	-1.361731
H	-3.504467	4.142412	1.172872
H	-2.408645	2.767715	1.286651
H	-2.224130	3.937444	-0.015066
H	-5.273047	4.225343	-0.620472
H	-4.068526	4.053372	-1.886186
H	-5.425935	2.944198	-1.818432
H	-4.514025	1.015567	3.904429
H	-2.830742	0.532115	3.742690
H	-3.578487	1.536184	2.508499
H	-6.313460	-0.038455	2.620203
H	-5.589556	0.530133	1.123595
H	-5.992526	-1.166336	1.303991
H	-3.329346	-4.287301	-1.090501
H	-2.324297	-2.855076	-1.286779
H	-2.039357	-3.955603	0.057566
H	-5.084444	-4.387664	0.685085

H	-3.882408	-4.135706	1.938988
H	-5.290290	-3.091974	1.858797
H	2.690618	3.600524	-1.073431
H	1.227508	2.668831	-1.290977
H	1.124032	4.328844	-0.712965
H	-0.036298	3.856618	1.446103
H	-0.294835	2.240167	0.933015
H	0.473051	2.532687	2.490568
H	5.997414	3.520481	0.492458
H	4.315025	4.025857	0.421431
H	4.987536	3.411736	1.926200
H	6.687978	1.200681	0.483705
H	5.813029	0.891195	1.977078
H	5.542654	-0.131177	0.573528
H	4.125494	1.444429	-3.513607
H	2.647366	0.496318	-3.582953
H	2.954941	1.527796	-2.191360
H	5.534121	-0.604731	-3.847412
H	5.284777	-2.062827	-2.893389
H	4.050330	-1.520235	-4.029872
H	-0.024196	-3.450643	-1.821842
H	0.558433	-2.884986	-0.277900
H	-0.396393	-1.851827	-1.367316
H	2.546557	-4.154270	-1.795259
H	1.719562	-3.879382	-3.320858
H	3.327984	-3.215881	-3.052956
H	3.488810	-3.762240	1.776733
H	2.100769	-2.766475	1.351611
H	2.611352	-4.072231	0.286108
H	5.611889	-3.639728	0.427363
H	4.843935	-3.749920	-1.151109
H	5.859307	-2.364996	-0.761559
H	3.099854	-0.657097	4.278671
H	2.818850	-1.251443	2.640663
H	4.369925	-0.546816	3.066765
H	3.158720	1.806381	4.759155
H	4.467382	2.024234	3.607132
H	3.005992	2.994728	3.466456
H	-0.567452	-3.213081	3.397557
H	-2.236555	-2.746919	3.721111
H	-1.868397	-3.843766	2.393314
H	0.463204	-0.846579	2.782601
H	-0.769647	0.357524	2.317641
H	-0.964120	-0.567029	3.754956

129

Ca1

Ca	-0.039573	0.383022	-0.042932
Si	3.037174	1.366182	0.580629

Si	-2.599067	-1.263582	1.033827
Si	-3.280293	1.163973	-0.836426
Si	2.569648	-1.501172	-0.749607
N	-2.310952	0.005334	-0.056976
N	2.233622	-0.029123	0.038013
C	4.058131	1.119012	2.169953
C	3.205803	0.654003	3.347429
C	4.932301	2.298728	2.596505
C	-0.933640	-1.443670	1.988996
C	-0.845303	-2.642044	2.930651
C	-0.515722	-0.169834	2.733549
C	4.337062	-1.643437	-1.433186
C	5.351335	-1.852283	-0.307860
C	4.580606	-2.663440	-2.544459
C	2.171671	-2.996477	0.369427
C	2.695126	-4.347253	-0.116594
C	2.581822	-2.761443	1.818452
C	-4.776853	0.561995	-1.833454
C	-5.875418	-0.038366	-0.959910
C	-4.384556	-0.372473	-2.977157
C	-3.946853	2.417652	0.437166
C	-2.813641	3.156405	1.142697
C	-5.007078	3.405944	-0.043045
C	-2.138458	2.105851	-2.048443
C	-1.250597	1.200758	-2.900122
C	-2.850005	3.114786	-2.947441
C	-2.841235	-2.967029	0.217838
C	-4.201431	-3.225414	-0.423383
C	-1.739331	-3.220667	-0.804719
C	1.337202	-1.557696	-2.220045
C	1.709359	-0.486424	-3.243025
C	1.086462	-2.900928	-2.898725
C	-4.028859	-0.978720	2.258750
C	-4.512341	-2.232675	2.988931
C	-3.769124	0.130635	3.278105
C	1.582075	2.576387	0.977143
C	0.874625	3.068090	-0.290849
C	1.845687	3.772753	1.889168
C	4.144030	2.195728	-0.749403
C	4.255691	3.720133	-0.678364
C	5.563028	1.628036	-0.832280
H	1.871261	3.463634	-3.846305
H	2.430582	3.287307	-3.387357
H	4.731996	0.291897	1.913978
H	-0.168545	-1.656913	1.219502
H	4.513355	-0.654846	-1.870460
H	1.075824	-3.061640	0.355340
H	-5.186195	1.475686	-2.282424
H	-4.425481	1.768237	1.178970

H	-1.484363	2.691290	-1.392245
H	-2.722295	-3.690863	1.033065
H	0.349664	-1.288348	-1.796104
H	-4.845433	-0.641197	1.609139
H	0.883304	1.951945	1.561500
H	3.633912	1.939165	-1.687480
H	-0.362237	1.718512	-3.270228
H	-0.933477	0.285193	-2.388519
H	-1.792066	0.835967	-3.773839
H	-3.557219	2.614996	-3.613010
H	-3.407835	3.854090	-2.374630
H	-2.137890	3.656294	-3.578306
H	-5.265021	-0.833104	-3.436248
H	-3.851757	0.161993	-3.764623
H	-3.730528	-1.178020	-2.638669
H	-6.752768	-0.305031	-1.557710
H	-5.540472	-0.947232	-0.462983
H	-6.204355	0.655266	-0.183743
H	-3.166732	3.708370	2.019169
H	-2.034337	2.470489	1.487057
H	-2.345944	3.885189	0.474998
H	-5.435419	3.960468	0.798135
H	-4.588077	4.145925	-0.727411
H	-5.829532	2.912209	-0.561840
H	-4.697998	0.440487	3.766500
H	-3.100045	-0.219239	4.066932
H	-3.318804	1.017222	2.833539
H	-5.410819	-2.016943	3.575653
H	-4.751480	-3.051902	2.312218
H	-3.757990	-2.599958	3.687469
H	-1.840550	-4.204298	-1.273180
H	-1.773101	-2.472461	-1.600566
H	-0.744469	-3.180325	-0.359093
H	-4.278816	-4.264737	-0.757956
H	-5.034620	-3.038284	0.254833
H	-4.349029	-2.596854	-1.300165
H	0.566667	-0.092412	2.853891
H	-0.889732	0.753769	2.270476
H	-0.949339	-0.154242	3.731950
H	0.134071	-2.698537	3.411011
H	-1.594375	-2.566844	3.720074
H	-1.009043	-3.582720	2.405344
H	3.661176	-2.631631	1.916320
H	2.112461	-1.862451	2.211897
H	2.293547	-3.604221	2.454024
H	2.301927	-5.158425	0.504184
H	2.421196	-4.561873	-1.148937
H	3.783182	-4.396056	-0.050740
H	5.630184	-2.641784	-2.855675

H	4.362484	-3.682081	-2.222539
H	3.978872	-2.462014	-3.430334
H	6.376624	-1.715548	-0.666015
H	5.199112	-1.166015	0.525933
H	5.284558	-2.864737	0.093377
H	4.888964	4.093660	-1.489507
H	3.297690	4.229515	-0.761187
H	4.716069	4.042573	0.257333
H	6.058694	1.965122	-1.747884
H	6.172962	1.974966	0.002994
H	5.596208	0.543914	-0.821057
H	0.935777	4.364727	2.029653
H	2.187710	3.460143	2.873994
H	2.604219	4.432519	1.468261
H	-0.149116	3.394836	-0.093382
H	1.393230	3.931050	-0.703565
H	0.872432	2.353596	-1.126462
H	5.547763	2.026327	3.459678
H	5.606852	2.628412	1.808447
H	4.333607	3.160766	2.892581
H	3.826700	0.430681	4.220617
H	2.489311	1.420299	3.654003
H	2.646912	-0.247129	3.105822
H	0.341368	-2.802684	-3.694154
H	1.996335	-3.293925	-3.352100
H	0.717068	-3.646823	-2.196864
H	0.944799	-0.376387	-4.016321
H	1.861908	0.488485	-2.774308
H	2.643634	-0.745715	-3.744021

	129		
Ca2*			
H	1.056667	0.990126	-2.296835
H	1.748671	0.622956	-1.539035
Si	2.785280	1.673584	0.307882
Si	-2.386809	-0.881365	1.470260
N	-2.358381	0.012765	0.031670
N	2.247785	0.203521	-0.451827
C	3.817623	1.299945	1.845623
C	2.955509	0.726028	2.970391
C	4.701547	2.438799	2.348757
H	4.487831	0.502161	1.513118
C	-0.753831	-0.469683	2.410422
H	0.059644	-0.362433	1.667469
C	-0.255103	-1.554348	3.364021
C	-0.863163	0.864403	3.147338
Si	-3.413582	0.655153	-1.131812
Si	2.826181	-1.426003	-0.689872
C	4.632812	-1.573209	-1.274349

H	4.637249	-1.009030	-2.213791
C	5.732568	-0.979645	-0.398088
C	4.986268	-3.022657	-1.617773
C	2.542124	-2.454978	0.898021
H	1.679302	-1.967743	1.368725
C	2.201101	-3.935695	0.727736
C	3.707863	-2.366698	1.886124
C	-4.822923	-0.435247	-1.797979
C	-5.845235	-0.744393	-0.703720
H	-5.324340	0.204664	-2.534976
C	-4.400744	-1.713581	-2.520965
C	-4.284412	2.218464	-0.482413
C	-3.279264	3.274479	-0.037997
C	-5.374932	2.828221	-1.360373
H	-4.782511	1.848130	0.420345
C	-2.264552	1.116636	-2.603486
H	-1.482475	1.778581	-2.187541
C	-1.571343	-0.104057	-3.223748
C	-2.907584	1.948047	-3.711227
C	-2.281918	-2.754627	1.139010
H	-1.896659	-3.207080	2.058602
C	-3.597098	-3.449790	0.805739
C	-1.256556	-2.991535	0.036797
C	1.753846	-1.987772	-2.187645
H	0.778132	-1.498522	-2.042599
C	2.326110	-1.385976	-3.475741
C	1.431641	-3.462181	-2.420445
C	-3.886444	-0.598842	2.601559
H	-4.711297	-0.940477	1.964613
C	-3.883561	-1.479101	3.851920
C	-4.187971	0.850805	2.981656
C	1.182728	2.604285	0.818527
C	0.459977	3.252187	-0.368753
H	0.537232	1.826051	1.257570
C	1.317550	3.652763	1.923744
C	3.614821	2.761785	-1.006666
H	2.834773	2.786385	-1.780092
C	3.901669	4.205276	-0.595976
C	4.849495	2.145892	-1.655251
Ca	-0.207334	0.384982	-0.639838
H	-0.635815	0.166889	-3.716562
H	-1.361960	-0.907880	-2.505042
H	-2.219916	-0.568184	-3.965046
H	-3.786580	1.443778	-4.119040
H	-3.223845	2.926273	-3.352104
H	-2.208623	2.110312	-4.537111
H	-5.257221	-2.378590	-2.670767
H	-3.982288	-1.504658	-3.504527
H	-3.650203	-2.274880	-1.962899

H	-6.716473	-1.261397	-1.117512
H	-5.420489	-1.395456	0.060214
H	-6.203204	0.155676	-0.201450
H	-3.766866	4.109792	0.474254
H	-2.542993	2.849571	0.647894
H	-2.740134	3.693010	-0.892626
H	-5.879894	3.646280	-0.836621
H	-4.974556	3.242445	-2.286279
H	-6.137983	2.098577	-1.634606
H	-5.232922	0.968790	3.283466
H	-3.576128	1.171889	3.825188
H	-3.997119	1.544029	2.164551
H	-4.847069	-1.431244	4.368781
H	-3.681961	-2.527999	3.629295
H	-3.125472	-1.143825	4.563552
H	-1.074093	-4.056668	-0.132959
H	-1.601660	-2.564678	-0.907884
H	-0.287543	-2.540295	0.276351
H	-3.446938	-4.523132	0.650141
H	-4.335348	-3.335416	1.600825
H	-4.038790	-3.054035	-0.107604
H	0.110576	1.210053	3.501441
H	-1.299818	1.651355	2.529084
H	-1.502363	0.753993	4.023378
H	0.643598	-1.226984	3.895137
H	-1.008069	-1.793443	4.116249
H	-0.009540	-2.479468	2.842636
H	4.537652	-2.991132	1.550461
H	4.090665	-1.359700	2.022300
H	3.401021	-2.739641	2.867429
H	2.127685	-4.414672	1.709205
H	1.257930	-4.100033	0.217653
H	2.974857	-4.465198	0.169289
H	5.990755	-3.082075	-2.047566
H	4.981605	-3.654832	-0.727445
H	4.297848	-3.463208	-2.338227
H	6.687008	-0.990113	-0.933525
H	5.534606	0.052664	-0.121154
H	5.872487	-1.550906	0.518639
H	4.246663	4.785477	-1.457178
H	3.025243	4.713663	-0.195136
H	4.683828	4.261173	0.162178
H	5.178711	2.749048	-2.506753
H	5.687751	2.094912	-0.956615
H	4.656319	1.138189	-2.020413
H	0.340690	4.083061	2.162235
H	1.727523	3.242935	2.843639
H	1.965550	4.472596	1.612332
H	-0.558083	3.545800	-0.108406

H	0.985096	4.157118	-0.674850
H	0.441014	2.642431	-1.283009
H	5.252803	2.131154	3.242520
H	5.435265	2.740568	1.600709
H	4.120038	3.322818	2.615704
H	3.573540	0.255448	3.739662
H	2.374949	1.505026	3.463467
H	2.254208	-0.026314	2.608161
H	0.932128	-3.583988	-3.386378
H	2.326095	-4.084975	-2.440394
H	0.767984	-3.867793	-1.662111
H	1.605559	-1.483042	-4.292503
H	2.562239	-0.327134	-3.375020
H	3.232431	-1.914362	-3.777577

129

Ca3

H	-0.827665	1.209091	-2.181507
H	-1.993669	0.568881	-1.283832
N	2.387361	-0.042628	-0.029010
Si	2.628307	-0.896616	1.415737
Ca	0.212080	0.421840	-0.534780
Si	-3.030304	1.500887	0.502331
Si	-3.108102	-1.402208	-0.858513
N	-2.363954	0.132307	-0.391312
Si	3.389981	0.649688	-1.221923
C	4.022217	-0.695489	-2.413510
H	4.537625	-1.392308	-1.740734
C	5.032877	-0.280725	-3.480701
C	2.852757	-1.449919	-3.039008
C	4.930121	1.675236	-0.757971
C	4.717019	2.858887	0.181167
H	5.202145	2.086839	-1.740374
C	6.126044	0.859015	-0.272991
C	3.692913	-2.482094	1.319484
C	3.373223	-3.399191	0.138120
H	3.458837	-3.025382	2.246173
C	5.197401	-2.213345	1.313585
C	3.473878	0.239397	2.693022
H	4.395387	0.489410	2.151267
C	3.892570	-0.363708	4.033190
C	2.725101	1.552347	2.899279
C	2.219275	1.817158	-2.190731
H	1.382479	1.184933	-2.543015
C	2.780629	2.440821	-3.465720
C	1.596360	2.915934	-1.322698
C	0.892295	-1.466766	2.077279
H	0.120787	-0.793111	1.661760
C	0.735625	-1.361835	3.593151

C	0.507503	-2.885822	1.665758
C	-2.794237	-2.696829	0.492231
C	-4.941782	-1.291570	-1.344644
C	-2.181960	-1.945325	-2.418768
H	-2.481942	-2.987863	-2.566607
C	-2.563714	-1.154437	-3.671298
C	-0.661184	-1.916102	-2.247027
C	-5.383368	-2.605741	-1.999038
C	-5.964384	-0.902256	-0.277178
H	-4.959235	-0.514508	-2.119259
C	-2.426169	-4.077337	-0.053848
H	-1.910850	-2.307254	1.005879
C	-3.901762	-2.850609	1.533354
C	-4.355223	2.263689	-0.620141
C	-3.664234	0.909802	2.183643
C	-1.588715	2.724605	0.690138
C	-5.383674	3.192190	0.019700
C	-3.718171	2.898970	-1.860338
H	-4.899035	1.385035	-0.976749
C	-4.112146	2.048408	3.102447
H	-4.545757	0.298243	1.959930
C	-2.660188	0.022736	2.924177
C	-2.008548	4.169669	0.963850
C	-0.535241	2.330128	1.724313
H	-1.135766	2.720034	-0.314423
H	2.232476	3.800785	-1.302698
H	0.620160	3.217687	-1.712896
H	1.485314	2.629338	-0.268846
H	3.673318	3.035433	-3.257709
H	3.049400	1.686146	-4.202872
H	2.044591	3.103517	-3.930952
H	5.632634	3.453519	0.265393
H	3.925116	3.527165	-0.152451
H	4.460457	2.522752	1.185140
H	7.028551	1.477683	-0.237599
H	5.960350	0.481026	0.736342
H	6.341451	0.000930	-0.910348
H	3.191735	-2.315273	-3.617554
H	2.165492	-1.813172	-2.273986
H	2.285858	-0.809443	-3.720561
H	5.427040	-1.159000	-4.002176
H	4.582283	0.361160	-4.238377
H	5.883108	0.259789	-3.063715
H	3.982327	-4.308035	0.180698
H	2.331674	-3.707177	0.093561
H	3.601076	-2.902173	-0.803738
H	5.757370	-3.151147	1.385064
H	5.498380	-1.734202	0.381871
H	5.524920	-1.571679	2.130942

H	3.329352	2.277650	3.453730
H	2.456356	2.004185	1.944797
H	1.804279	1.406707	3.468447
H	4.621191	0.278020	4.538672
H	3.042138	-0.467835	4.706982
H	4.344699	-1.350916	3.928002
H	-3.632249	-1.201929	-3.882815
H	-2.279533	-0.105214	-3.563200
H	-2.038402	-1.542611	-4.549011
H	-0.154448	-2.586132	-2.944031
H	-0.305655	-0.908674	-2.497047
H	-0.325032	-2.206375	-1.244786
H	-6.410058	-2.525371	-2.367541
H	-4.753824	-2.890959	-2.841811
H	-5.365320	-3.428224	-1.280267
H	-6.940331	-0.722362	-0.737555
H	-6.096752	-1.698087	0.454513
H	-5.696970	0.000412	0.268613
H	-4.479188	3.090665	-2.621904
H	-3.249207	3.856634	-1.629474
H	-2.945019	2.269155	-2.307336
H	-6.081169	3.566687	-0.735441
H	-5.975237	2.680844	0.780787
H	-4.920743	4.061911	0.489437
H	0.372737	2.926561	1.620224
H	-0.908744	2.500674	2.734432
H	-0.241603	1.273572	1.727680
H	-2.502032	4.262586	1.932807
H	-1.133356	4.825458	0.980435
H	-2.690712	4.553010	0.208327
H	-3.143086	-0.500699	3.753328
H	-2.205945	-0.728149	2.280774
H	-1.853241	0.616305	3.351796
H	-4.552589	1.649741	4.020613
H	-3.262235	2.665985	3.399889
H	-4.848157	2.704148	2.640191
H	-3.554263	-3.458652	2.373687
H	-4.246319	-1.900063	1.936299
H	-4.767079	-3.359852	1.105573
H	-2.201197	-4.762916	0.768280
H	-3.249979	-4.514233	-0.622532
H	-1.553557	-4.055565	-0.705715
H	-0.264590	-1.681002	3.900992
H	1.451920	-2.011794	4.099256
H	0.890395	-0.350568	3.964108
H	-0.453480	-3.171518	2.100988
H	0.432599	-3.014100	0.585906
H	1.244780	-3.603191	2.027191

65
Ca4

Ca	0.217872	2.559807	-0.007430
Si	1.653568	-0.199330	-0.151941
Si	-1.555486	-0.183971	0.054796
N	0.058210	0.314324	-0.034394
C	2.704686	1.172261	0.722406
H	2.633934	2.080006	0.088456
C	2.225044	1.543300	2.127525
H	1.146223	1.719900	2.205389
H	2.730839	2.433752	2.508944
H	2.423644	0.726734	2.821154
C	4.201104	0.862353	0.747026
H	4.401282	0.013568	1.402529
H	4.779459	1.708965	1.127540
H	4.585484	0.612885	-0.242658
C	2.239419	-0.132175	-1.967071
H	3.323232	-0.291271	-1.988447
C	1.949630	1.241024	-2.571639
H	0.869964	1.440097	-2.548543
H	2.244653	1.298755	-3.623408
H	2.479930	2.050373	-2.058669
C	1.573385	-1.209609	-2.818538
H	1.766782	-2.214399	-2.440725
H	1.922262	-1.174179	-3.855249
H	0.490770	-1.071117	-2.832253
C	2.018253	-1.914580	0.565539
H	1.197335	-2.518790	0.159433
C	3.328263	-2.551995	0.105323
H	3.420980	-2.575412	-0.981086
H	3.404751	-3.583027	0.463818
H	4.194435	-2.013701	0.493660
C	1.898493	-1.961858	2.087991
H	2.752452	-1.473794	2.563422
H	1.880798	-2.994252	2.449448
H	0.993862	-1.469030	2.445079
C	-2.523222	1.474629	0.020828
H	-2.031674	2.114365	0.777892
C	-2.393345	2.163070	-1.344557
H	-1.414675	2.014050	-1.827302
H	-2.597646	3.236642	-1.289303
H	-3.098808	1.732302	-2.056301
C	-3.986257	1.457901	0.458273
H	-4.423509	2.459204	0.403216
H	-4.088289	1.114157	1.486918
H	-4.586546	0.799757	-0.170403
C	-1.877206	-1.103804	1.681129
H	-1.112263	-1.891243	1.670651
C	-1.578928	-0.193694	2.871503

H	-0.593682	0.267511	2.784780
H	-1.605887	-0.747334	3.815014
H	-2.311535	0.613425	2.951705
C	-3.232695	-1.788325	1.838241
H	-4.057311	-1.074317	1.807944
H	-3.295102	-2.310401	2.798408
H	-3.412339	-2.526007	1.055625
C	-2.071570	-1.244527	-1.436470
H	-1.592086	-0.740858	-2.286001
C	-1.470802	-2.645754	-1.335131
H	-0.395387	-2.616319	-1.160288
H	-1.637747	-3.217101	-2.253456
H	-1.919904	-3.210601	-0.515310
C	-3.566569	-1.324718	-1.738598
H	-4.130103	-1.744539	-0.903830
H	-3.752060	-1.965224	-2.606914
H	-3.995965	-0.347507	-1.961616
H	-0.354690	4.242789	0.987428

71

Ca5

Ca	-0.103886	2.056578	-1.026934
H	0.680489	3.380600	-2.380435
C	-1.047027	4.724620	0.008549
C	-1.722797	3.968477	0.870617
H	-1.278140	3.615932	1.794430
H	-2.758828	3.698512	0.700203
H	-1.491340	5.093000	-0.908396
H	-0.018282	5.017716	0.184286
Si	1.782833	-0.317324	-0.068061
Si	-1.391166	-0.731126	0.048779
N	0.123592	-0.042680	-0.217675
C	2.553865	1.444640	0.057211
H	2.344346	1.925239	-0.916922
C	1.942779	2.330523	1.147650
H	0.862395	2.182854	1.281203
H	2.131020	3.392973	0.965600
H	2.365023	2.088037	2.122680
C	4.077307	1.467482	0.165578
H	4.403934	1.063295	1.125662
H	4.467310	2.486777	0.090542
H	4.548432	0.876596	-0.620350
C	2.518689	-1.050546	-1.660242
H	3.610040	-1.004852	-1.565876
C	2.106190	-0.217684	-2.872618
H	1.018519	-0.244011	-2.995270
H	2.537703	-0.613151	-3.797117
H	2.407623	0.829847	-2.801511
C	2.121042	-2.507549	-1.881966

H	2.397032	-3.152589	-1.046193
H	2.593940	-2.913714	-2.781582
H	1.041658	-2.593970	-2.018019
C	2.277204	-1.402917	1.408690
H	1.591965	-2.256764	1.332391
C	3.702947	-1.949908	1.353653
H	3.909959	-2.478947	0.423062
H	3.886160	-2.647083	2.177358
H	4.439983	-1.149612	1.441885
C	2.015762	-0.731633	2.755719
H	2.749814	0.054461	2.946935
H	2.096196	-1.448590	3.578574
H	1.025440	-0.278287	2.806020
C	-2.612670	0.559202	-0.707901
H	-2.305816	1.523848	-0.259634
C	-2.463017	0.643095	-2.231771
H	-1.426355	0.583781	-2.588291
H	-2.921598	1.543001	-2.653317
H	-2.953172	-0.212279	-2.698461
C	-4.088641	0.447775	-0.333146
H	-4.680900	1.229814	-0.819157
H	-4.236871	0.542218	0.741939
H	-4.505090	-0.512060	-0.637678
C	-1.740132	-0.891371	1.909697
H	-0.862082	-1.441506	2.270906
C	-1.716108	0.484011	2.573339
H	-0.815607	1.038889	2.301341
H	-1.739949	0.406575	3.664609
H	-2.580714	1.084369	2.276547
C	-2.977492	-1.679311	2.332055
H	-3.899839	-1.216220	1.977610
H	-3.047415	-1.739423	3.422961
H	-2.961576	-2.700669	1.951798
C	-1.620853	-2.401685	-0.825308
H	-1.123416	-2.250141	-1.791718
C	-0.857699	-3.498960	-0.083067
H	0.175418	-3.215740	0.116203
H	-0.835868	-4.426574	-0.662912
H	-1.325808	-3.727414	0.876690
C	-3.053177	-2.848730	-1.109345
H	-3.642127	-2.939206	-0.194866
H	-3.062347	-3.829152	-1.596185
H	-3.579063	-2.158071	-1.769547

	71		
Ca6*			
C	-0.744739	4.265634	0.626541
C	0.301964	4.861628	-0.011647
H	-0.638257	3.863726	1.629116

H	-1.750861	4.317859	0.225091
H	1.252484	5.008676	0.480867
H	0.155136	5.462355	-0.897430
Ca	0.163532	2.146146	-0.600854
H	1.345155	3.714380	-1.228578
Si	1.683854	-0.559536	-0.113099
Si	-1.525064	-0.556636	0.064000
N	0.074388	-0.066430	-0.118474
C	2.684214	0.992677	0.435627
H	2.582864	1.728837	-0.384402
C	2.163885	1.667480	1.706793
H	1.070058	1.739554	1.750066
H	2.575886	2.672841	1.829307
H	2.434762	1.089800	2.590890
C	4.189594	0.758715	0.547990
H	4.417012	0.104155	1.391533
H	4.727862	1.697203	0.709905
H	4.598990	0.294540	-0.350126
C	2.268841	-0.889005	-1.896115
H	3.361425	-0.974488	-1.887618
C	1.887269	0.286575	-2.795056
H	0.795011	0.373641	-2.845782
H	2.231801	0.144641	-3.823658
H	2.301635	1.242527	-2.458304
C	1.685254	-2.176845	-2.471104
H	1.940447	-3.054333	-1.875151
H	2.039563	-2.356508	-3.491085
H	0.595793	-2.117261	-2.511588
C	2.067170	-2.063551	0.975451
H	1.278197	-2.775693	0.702611
C	3.411107	-2.736844	0.701082
H	3.539072	-2.997892	-0.350091
H	3.512330	-3.657867	1.283711
H	4.245107	-2.089773	0.977884
C	1.910663	-1.762174	2.465092
H	2.734457	-1.141660	2.825510
H	1.920442	-2.681280	3.058909
H	0.981250	-1.235010	2.682851
C	-2.526943	1.040963	-0.343703
H	-2.046964	1.828202	0.267566
C	-2.402331	1.415618	-1.826762
H	-1.418936	1.195640	-2.266506
H	-2.650388	2.464546	-2.022034
H	-3.090137	0.814100	-2.422144
C	-3.992490	1.097858	0.081073
H	-4.450504	2.051299	-0.201121
H	-4.099254	0.990795	1.159716
H	-4.574467	0.304068	-0.387365
C	-1.874023	-1.081461	1.854108

H	-1.097050	-1.834776	2.037663
C	-1.609115	0.082788	2.807316
H	-0.628071	0.526551	2.628961
H	-1.643847	-0.238483	3.852809
H	-2.355816	0.873125	2.690940
C	-3.220787	-1.737469	2.146862
H	-4.057161	-1.067193	1.941599
H	-3.292064	-2.026079	3.200523
H	-3.373969	-2.638918	1.553033
C	-2.023494	-1.927188	-1.155113
H	-1.538382	-1.621224	-2.091249
C	-1.407680	-3.262052	-0.737019
H	-0.334830	-3.178616	-0.563960
H	-1.557176	-4.026066	-1.506097
H	-1.860011	-3.636218	0.184082
C	-3.514133	-2.092383	-1.442741
H	-4.081604	-2.322831	-0.539530
H	-3.684111	-2.912644	-2.147612
H	-3.952663	-1.195513	-1.881975

	71		
Ca7			
Ca	0.438815	2.097120	-0.545214
C	0.122785	4.189801	0.496201
C	1.489148	4.592415	-0.061269
H	2.090829	5.265151	0.561776
H	2.191396	3.734187	-0.224586
H	0.150155	4.159314	1.590365
H	-0.630576	4.929802	0.218454
H	1.402717	5.073926	-1.041636
Si	1.543663	-0.781381	-0.118215
Si	-1.638727	-0.329113	0.067584
N	0.020546	-0.080352	-0.116031
C	2.765873	0.612788	0.437766
H	2.777771	1.363191	-0.378833
C	2.370844	1.336235	1.727015
H	1.325959	1.664356	1.762468
H	2.995384	2.215382	1.905474
H	2.483934	0.671405	2.582894
C	4.218440	0.146833	0.527113
H	4.342962	-0.551568	1.356073
H	4.897495	0.985570	0.705857
H	4.544901	-0.359099	-0.382300
C	2.093597	-1.176339	-1.902907
H	3.150012	-1.466553	-1.886053
C	1.954518	0.059029	-2.791486
H	0.907467	0.387996	-2.812729
H	2.230255	-0.147864	-3.829840
H	2.587372	0.889999	-2.462676

C	1.284124	-2.323932	-2.500568
H	1.370711	-3.240398	-1.915222
H	1.605092	-2.553160	-3.521594
H	0.224712	-2.063826	-2.540386
C	1.729253	-2.333569	0.953693
H	0.839307	-2.915297	0.682191
C	2.951752	-3.199003	0.653324
H	3.023175	-3.465772	-0.401850
H	2.918761	-4.130772	1.226393
H	3.879036	-2.690759	0.923103
C	1.630632	-2.034331	2.448684
H	2.541196	-1.551878	2.811652
H	1.505219	-2.953974	3.027983
H	0.792191	-1.377162	2.681593
C	-2.389320	1.389455	-0.347092
H	-1.805647	2.114637	0.251228
C	-2.198608	1.734265	-1.829880
H	-1.248016	1.372666	-2.251935
H	-2.281797	2.809608	-2.019018
H	-2.956256	1.239589	-2.439302
C	-3.833856	1.659254	0.067703
H	-4.139768	2.672070	-0.212204
H	-3.959205	1.569081	1.146136
H	-4.528020	0.963103	-0.404067
C	-2.040835	-0.808922	1.857555
H	-1.379947	-1.668465	2.030302
C	-1.602781	0.301622	2.811119
H	-0.570934	0.603754	2.624532
H	-1.674563	-0.016517	3.855812
H	-2.226952	1.192117	2.702078
C	-3.466638	-1.263060	2.158492
H	-4.196902	-0.476905	1.959694
H	-3.572367	-1.540302	3.212358
H	-3.753667	-2.131223	1.564440
C	-2.316752	-1.622563	-1.150755
H	-1.798157	-1.383401	-2.088481
C	-1.889621	-3.030088	-0.737415
H	-0.814412	-3.096283	-0.570748
H	-2.148154	-3.765923	-1.505296
H	-2.383332	-3.338665	0.186809
C	-3.816508	-1.579521	-1.435706
H	-4.408956	-1.730276	-0.531781
H	-4.099987	-2.367044	-2.141651
H	-4.125891	-0.629057	-1.871674

73

Ca8

C	-1.752055	4.441161	-0.349533
C	-0.257099	4.197976	-0.531500

H	-1.970590	5.258551	0.341830
H	-2.303230	4.644041	-1.272286
H	0.328825	5.028784	-0.136532
H	-0.003815	4.107354	-1.592244
H	-2.329073	3.577767	0.092980
Ca	-0.545466	2.068926	0.461705
H	-0.872043	3.522526	2.713941
H	-0.516049	3.931367	2.193072
Si	-1.501662	-0.859820	0.131688
Si	1.660273	-0.304082	-0.074507
N	-0.006373	-0.103404	0.098074
C	-2.767621	0.441193	-0.539831
H	-2.842022	1.239355	0.226066
C	-2.358548	1.104006	-1.856505
H	-1.327198	1.473912	-1.872497
H	-3.013350	1.941675	-2.108915
H	-2.408591	0.385250	-2.674241
C	-4.193928	-0.096179	-0.646787
H	-4.259738	-0.846370	-1.436272
H	-4.903538	0.698491	-0.894771
H	-4.527338	-0.563547	0.280631
C	-2.054116	-1.133295	1.939338
H	-3.103860	-1.447147	1.943302
C	-1.944642	0.169036	2.731512
H	-0.900768	0.508993	2.747050
H	-2.236832	0.041757	3.778034
H	-2.582379	0.965052	2.330450
C	-1.218624	-2.211122	2.624834
H	-1.284233	-3.172489	2.113602
H	-1.533507	-2.366615	3.661547
H	-0.165383	-1.923993	2.641096
C	-1.623735	-2.493458	-0.821328
H	-0.716287	-3.022115	-0.504416
C	-2.817915	-3.376426	-0.463191
H	-2.888965	-3.565141	0.608721
H	-2.747280	-4.347085	-0.963701
H	-3.759993	-2.923350	-0.776105
C	-1.525499	-2.301998	-2.334101
H	-2.446801	-1.873271	-2.735389
H	-1.371086	-3.257905	-2.843502
H	-0.704199	-1.640685	-2.611865
C	2.354051	1.457156	0.241137
H	1.727990	2.130243	-0.373484
C	2.177257	1.862894	1.709285
H	1.245680	1.492060	2.161886
H	2.221454	2.947755	1.844746
H	2.963011	1.424088	2.325971
C	3.779574	1.759167	-0.214528
H	4.052812	2.794454	0.011906

H	3.890844	1.621307	-1.289550
H	4.506720	1.111891	0.277157
C	2.077775	-0.878973	-1.833827
H	1.450897	-1.772591	-1.951138
C	1.595135	0.153126	-2.851613
H	0.550331	0.418854	-2.683400
H	1.684579	-0.221672	-3.876027
H	2.179739	1.074835	-2.793800
C	3.519562	-1.295136	-2.112595
H	4.219781	-0.472039	-1.960829
H	3.634916	-1.628079	-3.149257
H	3.839349	-2.116668	-1.470869
C	2.384642	-1.500255	1.215554
H	1.866501	-1.218218	2.141645
C	1.998334	-2.943299	0.896052
H	0.923403	-3.053857	0.753160
H	2.293446	-3.622253	1.702283
H	2.487251	-3.291846	-0.016549
C	3.884695	-1.394383	1.481969
H	4.473383	-1.592596	0.584875
H	4.196821	-2.121235	2.239084
H	4.170318	-0.406802	1.845374

	73		
Ca9*			
C	-2.132521	4.226875	-0.262266
C	-1.107046	4.027773	0.842252
H	-3.057010	3.660102	-0.132250
H	-2.413956	5.271585	-0.398584
H	-1.435305	3.285615	1.585091
H	-0.892051	4.941074	1.390756
H	-1.736441	3.947938	-1.273483
Ca	-0.218586	2.166876	-0.453625
H	0.737998	4.104681	-0.842816
H	0.219026	4.152639	-0.096676
Si	1.800074	-0.220979	-0.135908
Si	-1.336571	-0.858154	0.075508
N	0.126201	-0.037039	-0.095485
C	2.525807	1.442422	0.511327
H	2.269320	2.206500	-0.245983
C	1.944821	1.915362	1.845215
H	0.854220	1.839957	1.900470
H	2.212471	2.953769	2.055769
H	2.318040	1.302386	2.665789
C	4.052684	1.467757	0.567061
H	4.418970	0.800778	1.349510
H	4.425556	2.470415	0.795377
H	4.504895	1.152318	-0.374175
C	2.391125	-0.308188	-1.947706

H	3.482369	-0.209959	-1.959691
C	1.798856	0.852806	-2.745973
H	0.704458	0.770215	-2.763132
H	2.120930	0.840955	-3.791367
H	2.078864	1.834003	-2.348122
C	2.014599	-1.626427	-2.617942
H	2.430078	-2.490864	-2.098261
H	2.365492	-1.664673	-3.654073
H	0.929955	-1.749254	-2.636466
C	2.482996	-1.699232	0.834890
H	1.821158	-2.520661	0.535501
C	3.911655	-2.107247	0.477743
H	4.041035	-2.278613	-0.591577
H	4.193720	-3.029578	0.995044
H	4.631456	-1.341984	0.772674
C	2.334035	-1.529503	2.346240
H	3.055094	-0.804394	2.730039
H	2.519895	-2.472222	2.869700
H	1.338409	-1.183649	2.626298
C	-2.658376	0.499539	-0.281662
H	-2.353759	1.363578	0.336195
C	-2.635648	0.906404	-1.759249
H	-1.625311	1.002626	-2.182585
H	-3.179958	1.835613	-1.947869
H	-3.099970	0.129856	-2.368327
C	-4.094808	0.230553	0.161309
H	-4.750651	1.067750	-0.097907
H	-4.158353	0.086586	1.239135
H	-4.500692	-0.662096	-0.314193
C	-1.532887	-1.505087	1.847753
H	-0.612671	-2.085917	1.991776
C	-1.494043	-0.342294	2.838167
H	-0.628674	0.299377	2.664146
H	-1.442724	-0.697419	3.871943
H	-2.388772	0.281232	2.758673
C	-2.706985	-2.436865	2.135676
H	-3.668840	-1.952427	1.959553
H	-2.697872	-2.761283	3.181252
H	-2.677159	-3.334771	1.517823
C	-1.562902	-2.261901	-1.188549
H	-1.186264	-1.826970	-2.123458
C	-0.666787	-3.450637	-0.844229
H	0.374282	-3.153913	-0.722770
H	-0.700322	-4.211608	-1.630034
H	-0.982085	-3.929992	0.085257
C	-2.991753	-2.735856	-1.448494
H	-3.468812	-3.113645	-0.542847
H	-3.000211	-3.550347	-2.180003
H	-3.627513	-1.943231	-1.844004

73
Ca10

Ca	-0.443968	2.055369	-0.781241
H	-0.030166	3.433574	-2.225506
C	-2.111429	3.793710	0.852483
C	-3.081536	3.803012	-0.320922
H	-3.950027	3.173504	-0.127667
H	-2.622074	3.477219	-1.261579
H	-1.185063	4.326608	0.615080
H	-2.537948	4.286245	1.725902
H	-3.431859	4.816524	-0.514780
H	-1.872822	2.779648	1.202165
Si	1.868886	-0.012355	-0.095457
Si	-1.178046	-0.992454	0.060864
N	0.182228	-0.025429	-0.179257
C	2.323394	1.855173	0.067952
H	1.972367	2.318976	-0.873332
C	1.636567	2.579275	1.232097
H	0.627228	2.205840	1.455371
H	1.585772	3.661516	1.074601
H	2.183444	2.415424	2.160282
C	3.821576	2.151799	0.089908
H	4.277574	1.777340	1.008225
H	4.016059	3.227292	0.043139
H	4.337906	1.687233	-0.750210
C	2.679146	-0.559003	-1.724626
H	3.745430	-0.314475	-1.649437
C	2.091229	0.214386	-2.903447
H	1.022836	0.000385	-3.001652
H	2.563951	-0.075027	-3.846948
H	2.200598	1.296654	-2.807203
C	2.556270	-2.058913	-1.980590
H	2.961457	-2.661071	-1.165638
H	3.082392	-2.346815	-2.896092
H	1.510518	-2.343275	-2.107615
C	2.591402	-1.034303	1.332142
H	2.080817	-2.000302	1.228673
C	4.093824	-1.296427	1.239710
H	4.381629	-1.735247	0.283918
H	4.420775	-1.980855	2.028878
H	4.666633	-0.374798	1.358574
C	2.229825	-0.477480	2.707730
H	2.806727	0.424022	2.926135
H	2.454820	-1.198194	3.499881
H	1.172935	-0.220186	2.783183
C	-2.629267	0.079734	-0.625417
H	-2.516673	1.060529	-0.125932
C	-2.511386	0.277500	-2.139879
H	-1.496067	0.501211	-2.490304

H	-3.178645	1.060515	-2.511731
H	-2.779271	-0.645563	-2.655866
C	-4.052694	-0.334872	-0.259910
H	-4.786835	0.341516	-0.709302
H	-4.210204	-0.322722	0.817933
H	-4.278378	-1.340575	-0.612778
C	-1.464442	-1.288380	1.916699
H	-0.495939	-1.682796	2.249148
C	-1.681408	0.040766	2.637307
H	-0.894070	0.757652	2.391945
H	-1.683410	-0.083294	3.724493
H	-2.641471	0.489730	2.366254
C	-2.531571	-2.304625	2.314940
H	-3.528723	-2.000112	1.993208
H	-2.568403	-2.425700	3.402410
H	-2.339843	-3.287997	1.885749
C	-1.106453	-2.639970	-0.880723
H	-0.651565	-2.360537	-1.839390
C	-0.151979	-3.609501	-0.183297
H	0.812262	-3.150672	0.033286
H	0.038069	-4.490984	-0.802953
H	-0.566995	-3.961843	0.763291
C	-2.434886	-3.328890	-1.185006
H	-2.993297	-3.560101	-0.276054
H	-2.267246	-4.275274	-1.709009
H	-3.080282	-2.720734	-1.819735

55

CaN"$_2$

Ca	-0.000155	0.000813	-1.159943
Si	2.048185	-1.346234	1.124931
Si	-2.049042	1.347991	1.122786
Si	-2.986070	-0.838962	-0.908619
Si	2.986597	0.836719	-0.909479
N	1.962544	-0.291657	-0.198929
N	-1.963380	0.291434	-0.199393
C	4.434909	0.096132	-1.844413
C	3.631979	2.164990	0.245979
C	3.523547	-2.506577	1.045505
C	2.103343	-0.441560	2.770694
C	1.922758	1.750138	-2.228028
C	0.472709	-2.391196	1.093693
C	-3.630060	-2.166546	0.248395
C	-4.435199	-0.101094	-1.844452
C	-1.920994	-1.752563	-2.225989
C	-3.524189	2.508462	1.041561
C	-2.104069	0.446076	2.770039
C	-0.473397	2.392807	1.089563
H	5.096571	-0.431051	-1.153410

H	5.027105	0.855961	-2.360053
H	4.091909	-0.631031	-2.583909
H	2.810666	2.665117	0.762836
H	4.217048	2.921711	-0.282127
H	4.276425	1.719443	1.007472
H	3.532795	-3.059904	0.103795
H	3.517096	-3.229820	1.864622
H	4.458999	-1.944871	1.107422
H	2.999494	0.179194	2.840464
H	2.110535	-1.134831	3.615343
H	1.239415	0.216384	2.884378
H	1.558705	1.101002	-3.038166
H	2.539162	2.497443	-2.731261
H	1.081970	2.320437	-1.808850
H	-0.431108	-1.771821	1.105761
H	0.408592	-3.048794	1.963456
H	0.439362	-3.032722	0.207345
H	-2.536265	-2.501350	-2.728406
H	-1.557784	-1.103855	-3.036852
H	-1.079420	-2.321118	-1.806035
H	-5.026488	-0.862189	-2.359268
H	-5.097511	0.426073	-1.154054
H	-4.093035	0.625651	-2.584744
H	-4.214438	-2.924422	-0.278821
H	-2.808244	-2.665303	0.765765
H	-4.274888	-1.720741	1.009417
H	-3.517639	3.233002	1.859530
H	-3.533357	3.060288	0.098968
H	-4.459717	1.946982	1.104350
H	-2.111199	1.140720	3.613558
H	-3.000238	-0.174537	2.840858
H	-1.240160	-0.211716	2.884721
H	-0.409191	3.051946	1.958150
H	0.430501	1.773535	1.102775
H	-0.440099	3.032881	0.202142

28
N”H

Si	1.574548	0.008409	-0.087003
Si	-1.574542	-0.008385	-0.087007
N	0.000005	0.000105	-0.809741
C	-2.073031	1.699806	0.501064
C	-1.586096	-1.170824	1.381806
C	1.585986	1.170212	1.382307
C	2.073160	-1.700004	0.500336
C	-2.790962	-0.593134	-1.384803
C	2.790930	0.593833	-1.384531
H	-1.378652	2.071552	1.257056
H	-3.073837	1.694166	0.939824

H	-2.072157	2.411097	-0.327800
H	-1.342986	-2.189948	1.075678
H	-2.570484	-1.183862	1.855357
H	-0.862933	-0.868580	2.142191
H	1.342517	2.189395	1.076663
H	2.570468	1.183341	1.855661
H	0.863074	0.867422	2.142713
H	1.378649	-2.072216	1.255980
H	3.073865	-1.694453	0.939328
H	2.072576	-2.410890	-0.328877
H	-2.785237	0.065610	-2.256784
H	-3.809067	-0.603729	-0.989529
H	-2.551391	-1.603348	-1.723031
H	2.785182	-0.064457	-2.256855
H	3.809051	0.604237	-0.989293
H	2.551322	1.604216	-1.722229
H	0.000007	0.000042	-1.818792

	57		
Ca1'			
Ca	0.026002	0.903104	0.660068
Si	-1.999070	-1.745960	-0.151561
Si	2.151312	0.357765	-1.700552
Si	2.901481	-0.232032	1.327847
Si	-3.052268	1.122355	0.267619
N	-1.899878	-0.106187	0.286672
N	2.043856	0.289281	-0.019289
C	-4.358991	0.993880	1.607885
C	-3.907998	1.376703	-1.382883
C	-3.505543	-2.604852	0.574728
C	-2.050640	-1.976360	-2.018047
C	-2.104001	2.756930	0.622810
C	-0.452492	-2.613359	0.483737
C	3.420271	-2.032432	1.296661
C	4.410876	0.813189	1.722729
C	1.697660	-0.017173	2.804798
C	3.616601	1.355022	-2.320318
C	2.184992	-1.328827	-2.520552
C	0.572847	1.250404	-2.278005
H	-4.960506	0.094095	1.462630
H	-5.035185	1.852250	1.609000
H	-3.897683	0.920421	2.595396
H	-3.177227	1.508677	-2.183652
H	-4.570639	2.245412	-1.375905
H	-4.512491	0.501493	-1.632544
H	-3.530325	-2.495457	1.661189
H	-3.511474	-3.672387	0.341464
H	-4.428780	-2.175701	0.177085
H	-2.926557	-1.485208	-2.447602

H	-2.090178	-3.033017	-2.294103
H	-1.166575	-1.542628	-2.490548
H	-1.624407	2.794578	1.611603
H	-2.816774	3.583833	0.630321
H	-1.370012	3.026876	-0.148750
H	0.455038	-2.124741	0.116868
H	-0.411582	-3.655022	0.157386
H	-0.418804	-2.610810	1.576544
H	2.171763	-0.346027	3.731498
H	1.419020	1.030665	2.988932
H	0.787811	-0.626166	2.718123
H	4.902521	0.495322	2.645359
H	5.140806	0.738415	0.913206
H	4.146258	1.868078	1.826022
H	3.920143	-2.329362	2.221825
H	2.560517	-2.687399	1.146606
H	4.116172	-2.206930	0.472459
H	3.617139	1.450748	-3.408800
H	3.614976	2.359671	-1.891319
H	4.553959	0.875180	-2.028374
H	2.193803	-1.255011	-3.610659
H	3.076027	-1.882649	-2.215583
H	1.315648	-1.921221	-2.228871
H	0.535972	1.313913	-3.367479
H	-0.351340	0.736495	-1.983352
H	0.533567	2.285147	-1.915740
H	1.292624	3.347124	0.914057
H	1.757045	2.809033	0.664459

57

Ca2'*

Ca	-0.072228	-0.360724	1.217399
Si	-1.978125	1.536678	-0.813471
Si	3.077125	0.676935	1.039971
Si	2.067855	-1.130880	-1.351790
Si	-3.154849	-0.922792	0.655879
N	-1.978280	0.221767	0.253644
N	2.067164	-0.465882	0.244194
C	-3.747373	-1.957013	-0.795050
C	-4.658802	-0.209691	1.524894
C	-1.965225	1.015384	-2.620437
C	-3.424227	2.711050	-0.569109
C	-2.331947	-2.117187	1.892779
C	-0.390361	2.516139	-0.491793
C	2.070707	0.196464	-2.670007
C	3.529626	-2.268927	-1.618055
C	0.472865	-2.120374	-1.468086
C	4.579106	-0.138114	1.796368
C	3.605081	2.097579	-0.051222

C	2.014527	1.355018	2.460562
H	-4.265144	-1.329436	-1.524289
H	-4.440133	-2.741932	-0.482047
H	-2.906240	-2.429529	-1.306745
H	-4.363019	0.369791	2.402366
H	-5.348995	-0.991958	1.850342
H	-5.205674	0.461528	0.859386
H	-1.118892	0.361424	-2.839347
H	-1.906789	1.875951	-3.291502
H	-2.875080	0.462795	-2.865407
H	-4.367701	2.222688	-0.824113
H	-3.332047	3.599681	-1.198519
H	-3.491933	3.036388	0.471228
H	-1.460664	-2.657056	1.500195
H	-3.042080	-2.895105	2.181280
H	-2.040587	-1.629044	2.832163
H	-0.405676	2.978837	0.499446
H	-0.260619	3.321212	-1.218496
H	0.504640	1.889671	-0.564289
H	0.385996	-2.623227	-2.433279
H	0.439107	-2.893788	-0.695218
H	-0.416806	-1.488552	-1.366932
H	3.497223	-2.747192	-2.599676
H	4.469038	-1.715253	-1.548450
H	3.549484	-3.053311	-0.858555
H	1.953371	-0.256494	-3.657674
H	1.252362	0.903932	-2.531035
H	3.004699	0.760173	-2.673842
H	5.178098	0.564647	2.379855
H	4.278145	-0.958254	2.451622
H	5.217846	-0.557322	1.015879
H	4.137986	2.848490	0.537343
H	4.281431	1.755135	-0.836692
H	2.753338	2.583547	-0.528339
H	2.584244	2.048937	3.081392
H	1.145675	1.925050	2.108589
H	1.690188	0.552349	3.135247
H	1.256100	-1.973200	1.845462
H	1.799550	-1.379428	1.086499

	57		
Ca3'			
Ca	-0.073325	0.269692	-1.340751
Si	-1.970396	1.326640	1.017240
Si	1.977099	-1.547410	0.916980
Si	3.309847	0.848771	-0.713883
Si	-3.302793	-0.770585	-0.769042
N	-2.029876	0.246889	-0.279121
N	2.122501	-0.410375	-0.418420

C	-4.691363	0.180602	-1.610141
C	-4.058123	-1.740100	0.656270
C	-3.344436	2.606653	1.046446
C	-1.925265	0.501288	2.706054
C	-2.631980	-2.016841	-2.013279
C	-0.339767	2.304951	0.842411
C	3.792055	1.690308	0.877721
C	4.804783	0.132769	-1.567981
C	2.477300	2.081785	-1.863148
C	3.430480	-2.721204	0.943042
C	1.875369	-0.625569	2.535882
C	0.385734	-2.474291	0.601749
H	-5.134516	0.910596	-0.929339
H	-5.487880	-0.484596	-1.953046
H	-4.314647	0.727643	-2.477981
H	-3.291509	-2.298186	1.198432
H	-4.806640	-2.452781	0.301132
H	-4.549957	-1.074033	1.369377
H	-3.401610	3.142615	0.096636
H	-3.202854	3.338804	1.845354
H	-4.310922	2.123726	1.208794
H	-2.884098	0.023864	2.917753
H	-1.725121	1.216473	3.507815
H	-1.161684	-0.277453	2.746701
H	-2.138060	-1.544158	-2.868161
H	-3.440096	-2.632027	-2.416510
H	-1.907850	-2.693540	-1.553419
H	0.562427	1.682288	0.858872
H	-0.226471	2.998510	1.678297
H	-0.326131	2.922748	-0.063364
H	3.186269	2.836847	-2.208613
H	2.093891	1.572130	-2.753977
H	1.667622	2.628313	-1.368558
H	5.536431	0.906546	-1.810651
H	5.296440	-0.610553	-0.937397
H	4.511865	-0.358482	-2.498380
H	4.425431	2.551114	0.650374
H	2.925020	2.051749	1.431721
H	4.362017	1.027345	1.530231
H	3.325650	-3.463358	1.737659
H	3.518408	-3.254682	-0.005654
H	4.365872	-2.183103	1.112469
H	1.579055	-1.322033	3.323959
H	2.829753	-0.187170	2.826298
H	1.129675	0.169157	2.507684
H	0.243081	-3.265787	1.340331
H	-0.489713	-1.818826	0.662086
H	0.393533	-2.941902	-0.386184
H	0.835245	-0.835785	-2.847783

H	1.988847	-0.907362	-1.323407

29
Ca4'

Ca	0.000258	2.269259	0.084367
Si	1.583860	-0.505878	-0.021180
Si	-1.584003	-0.505690	-0.021163
N	-0.000033	0.067112	-0.049777
C	-1.951449	-1.675476	1.399750
C	-2.139182	-1.307773	-1.621363
C	1.951264	-1.675645	1.399757
C	2.138966	-1.308017	-1.621374
C	-2.674930	1.042747	0.240019
C	2.674981	1.042458	0.239982
H	-1.373495	-2.596345	1.293025
H	-3.008291	-1.949946	1.439983
H	-1.678847	-1.227745	2.358008
H	-1.993164	-0.630052	-2.465160
H	-3.192036	-1.598578	-1.594486
H	-1.549466	-2.206595	-1.817045
H	1.678633	-1.227899	2.357999
H	3.008103	-1.950124	1.440024
H	1.373305	-2.596510	1.293026
H	1.549233	-2.206839	-1.817003
H	3.191816	-1.598838	-1.594523
H	1.992927	-0.630319	-2.465187
H	-2.485008	1.542437	1.200765
H	-3.730526	0.764884	0.270092
H	-2.597953	1.781733	-0.569075
H	2.598237	1.781375	-0.569200
H	3.730523	0.764411	0.270192
H	2.485066	1.542273	1.200667
H	0.000113	3.839900	-1.198824

35
Ca5'

Ca	1.580104	-0.000382	-1.305856
Si	-0.882621	-1.574739	0.070290
Si	-0.881729	1.575188	0.070299
N	-0.360548	0.000080	-0.223606
C	-1.125060	1.975048	1.890736
C	-2.448875	2.047505	-0.844783
C	-1.126268	-1.974419	1.890726
C	-2.449998	-2.046163	-0.844858
C	0.508346	2.712401	-0.572425
C	0.506833	-2.712740	-0.572353
H	-1.935927	1.370976	2.304465
H	-1.382611	3.025644	2.045220
H	-0.229150	1.758019	2.476375

H	-2.336901	1.875087	-1.917349
H	-2.714676	3.096541	-0.693663
H	-3.287934	1.438049	-0.500794
H	-0.230265	-1.757876	2.476403
H	-1.384411	-3.024868	2.045221
H	-1.936820	-1.369888	2.304401
H	-3.288716	-1.436204	-0.500934
H	-2.716421	-3.095039	-0.693719
H	-2.337865	-1.873847	-1.917424
H	1.460161	2.567726	-0.044817
H	0.247767	3.761590	-0.418300
H	0.685569	2.613629	-1.651289
H	0.684180	-2.614044	-1.651202
H	0.245647	-3.761783	-0.418260
H	1.458695	-2.568602	-0.044686
H	2.319964	-0.000802	-3.209394
C	3.630933	-0.000988	0.894407
C	2.505143	-0.000675	1.604442
H	2.021601	-0.918745	1.914457
H	2.022134	0.917666	1.914486
H	4.126417	0.921590	0.609807
H	4.125885	-0.923842	0.609776

	35		
Ca6'*			
Si	0.763251	1.710911	0.010729
Si	1.356758	-1.371050	0.046486
N	0.474909	0.049483	-0.117903
C	2.095506	-1.649160	1.748872
C	2.703526	-1.614496	-1.236821
C	1.529438	2.211837	1.651333
C	1.845123	2.380542	-1.370911
C	0.098421	-2.800251	-0.222150
C	-0.927581	2.559400	-0.114710
H	2.849022	-0.887660	1.962020
H	2.576325	-2.626555	1.835127
H	1.329653	-1.574784	2.524040
H	2.302034	-1.518316	-2.247954
H	3.186753	-2.590712	-1.150920
H	3.475230	-0.850044	-1.119100
H	0.928531	1.849346	2.488323
H	1.619914	3.296992	1.742901
H	2.530284	1.786217	1.756150
H	2.837399	1.924438	-1.336119
H	1.973336	3.463608	-1.302646
H	1.412804	2.151518	-2.347621
H	-0.700902	-2.839119	0.532076
H	0.612859	-3.758609	-0.128425
H	-0.348339	-2.821997	-1.226512

H	-1.456527	2.318772	-1.045022
H	-0.828157	3.646996	-0.098631
H	-1.576755	2.296319	0.727464
C	-3.658666	-0.402150	1.054601
C	-4.307388	-0.059087	-0.097216
H	-3.240293	0.354756	1.710325
H	-3.695297	-1.417045	1.438210
H	-4.476899	0.975939	-0.358610
H	-4.933823	-0.764887	-0.623312
Ca	-1.620806	-0.558727	-0.523370
H	-3.235796	-0.104896	-1.718040

35

Ca7'

Si	0.671612	1.730996	0.029446
Si	1.474494	-1.337978	0.030542
N	0.543254	0.056548	-0.067856
C	2.225307	-1.653915	1.719689
C	2.832162	-1.452421	-1.261162
C	1.146922	2.366933	1.728709
C	1.833685	2.483782	-1.239035
C	0.264426	-2.781784	-0.316298
C	-1.078027	2.395824	-0.349377
H	2.939907	-0.865440	1.967849
H	2.753248	-2.609418	1.766739
H	1.453028	-1.650601	2.491831
H	2.426117	-1.329453	-2.267759
H	3.360917	-2.407800	-1.220417
H	3.566949	-0.658680	-1.106728
H	0.457442	1.989996	2.487157
H	1.145557	3.458533	1.777117
H	2.148825	2.022273	1.995757
H	2.858318	2.149240	-1.059847
H	1.829530	3.575914	-1.203336
H	1.561266	2.174722	-2.250723
H	-0.543412	-2.864628	0.423996
H	0.788205	-3.738896	-0.274710
H	-0.172645	-2.740832	-1.324705
H	-1.418891	2.138824	-1.361999
H	-1.097694	3.486814	-0.308960
H	-1.832507	2.065199	0.376739
Ca	-1.571139	-0.525842	-0.403623
C	-3.493005	-0.675166	0.945591
C	-4.321008	-0.281964	-0.284714
H	-5.297453	0.179834	-0.089732
H	-3.821731	0.472103	-0.937587
H	-3.575810	0.100503	1.715236
H	-3.895013	-1.591931	1.383676
H	-4.522395	-1.145258	-0.932388

	37		
Ca8'			
Si	-0.687904	1.730646	0.010247
Si	-1.464567	-1.276036	0.072653
N	-0.434488	0.052302	0.013828
C	-2.694462	-1.391236	-1.340235
C	-2.394540	-1.487842	1.688720
C	-1.444171	2.337511	-1.600912
C	-1.786791	2.312101	1.420078
C	-0.341095	-2.832682	-0.082212
C	0.998403	2.557081	0.200988
H	-3.406020	-0.563984	-1.285788
H	-3.266291	-2.321984	-1.316101
H	-2.189976	-1.323217	-2.306433
H	-1.706960	-1.495729	2.537428
H	-2.976736	-2.412263	1.711282
H	-3.085025	-0.655123	1.838566
H	-0.818699	2.057689	-2.451974
H	-1.560400	3.424068	-1.612092
H	-2.430336	1.894824	-1.758494
H	-2.798611	1.912995	1.314487
H	-1.863289	3.401820	1.448609
H	-1.397056	1.974502	2.382914
H	0.207277	-2.904180	-1.032677
H	-0.969482	-3.725195	-0.060637
H	0.359814	-2.969214	0.754291
H	1.458692	2.309358	1.162002
H	0.915750	3.645474	0.157129
H	1.696509	2.264851	-0.590523
Ca	1.602990	-0.804312	-0.197337
C	3.854418	-0.147723	-0.527110
C	3.967071	-0.235277	0.997411
H	4.662922	0.465564	1.472952
H	3.014446	-0.018420	1.547656
H	3.999662	0.886658	-0.855210
H	4.646165	-0.740791	-0.990283
H	4.264794	-1.236853	1.328887
H	1.336912	-0.641546	-2.709684
H	2.069138	-0.545318	-2.537341

	37		
Ca9'*			
Si	0.782118	1.712500	0.010914
Si	1.337471	-1.368662	0.063077
N	0.474476	0.056805	-0.153814
C	1.969400	-1.652731	1.807029
C	2.756110	-1.622826	-1.137469
C	1.285105	2.200269	1.754892
C	2.097159	2.340153	-1.174520

C	0.089135	-2.792741	-0.279467
C	-0.837014	2.608282	-0.388995
H	2.720693	-0.902217	2.062961
H	2.429539	-2.636827	1.924250
H	1.159768	-1.563951	2.534686
H	2.414391	-1.529809	-2.170577
H	3.229123	-2.600622	-1.018945
H	3.522565	-0.860766	-0.978427
H	0.539301	1.869411	2.481330
H	1.401131	3.282008	1.857606
H	2.236435	1.737103	2.027031
H	3.060494	1.868610	-0.965793
H	2.233018	3.421713	-1.097744
H	1.832781	2.103900	-2.207658
H	-0.748705	-2.834489	0.431754
H	0.597491	-3.751422	-0.160663
H	-0.303205	-2.812346	-1.306678
H	-1.188852	2.395181	-1.404637
H	-0.716197	3.692162	-0.327709
H	-1.637896	2.347964	0.311148
C	-3.360868	-0.419060	1.273356
C	-3.881929	0.093147	-0.068848
H	-2.344673	-0.057600	1.532641
H	-3.955030	-0.116459	2.141588
H	-4.012763	1.178873	-0.038858
H	-4.858841	-0.347084	-0.276663
H	-3.323861	-1.517707	1.314429
Ca	-1.586317	-0.576160	-0.667813
H	-2.900761	-0.388120	-2.384033
H	-3.357770	-0.192652	-1.599827

37

Ca10'

Si	1.325946	1.300637	0.132454
Si	0.625959	-1.741054	0.024174
N	0.433498	-0.078131	-0.236411
C	0.957003	-2.171828	1.824180
C	1.990194	-2.495500	-1.020910
C	1.524937	1.630001	1.970538
C	3.013279	1.387690	-0.679570
C	-1.008050	-2.561343	-0.465357
C	0.316802	2.783114	-0.553479
H	1.921011	-1.772246	2.147886
H	0.976955	-3.252636	1.984170
H	0.189859	-1.747616	2.476219
H	1.811472	-2.307505	-2.081936
H	2.066495	-3.575911	-0.876177
H	2.957475	-2.055367	-0.767411
H	0.554383	1.652164	2.471708

H	2.029858	2.579107	2.165934
H	2.113362	0.837238	2.437327
H	3.655790	0.590755	-0.297755
H	3.515429	2.339243	-0.489397
H	2.932090	1.252007	-1.760039
H	-1.825545	-2.262588	0.198551
H	-0.939506	-3.649548	-0.400024
H	-1.304616	-2.336484	-1.496638
H	0.170696	2.763638	-1.642072
H	0.864567	3.707797	-0.360925
H	-0.654791	2.930066	-0.061002
Ca	-1.427183	0.618780	-1.214904
H	-2.498310	0.321849	-2.922661
C	-2.874821	0.841992	1.233639
C	-3.910711	-0.079175	0.599778
H	-3.904851	-1.057960	1.078292
H	-3.767421	-0.247748	-0.475819
H	-2.860859	1.834161	0.765028
H	-3.102058	1.022429	2.283777
H	-4.911570	0.340315	0.699467
H	-1.859218	0.422303	1.230748

XYZ-Files for hydrogenation of benzene with 1-Ba

12
Benzene C_6H_6

C	0.000000	0.000000	1.388528
C	0.000000	1.202499	0.694261
C	0.000000	1.202504	-0.694262
C	0.000000	0.000000	-1.388526
C	0.000000	-1.202499	0.694261
C	0.000000	-1.202504	-0.694262
H	0.000000	2.140546	1.235847
H	0.000000	2.140550	-1.235847
H	0.000000	0.000000	-2.471690
H	0.000000	-2.140546	1.235847
H	0.000000	-2.140550	-1.235847
H	0.000000	0.000000	2.471693

14
1,3-Cyclohexadiene (1,3-C_6H_8)

C	-1.188650	-0.724182	-0.246127
C	-1.189005	0.723620	0.246142
C	0.107364	1.420935	-0.064636
C	1.257246	0.723844	-0.108182
C	0.108033	-1.420882	0.064641
C	1.257618	-0.723265	0.108211
H	-2.043641	1.271484	-0.180132
H	0.110195	2.508064	-0.188335
H	0.111328	-2.508042	0.188084
H	2.210545	-1.230888	0.282133
H	-2.043043	-1.272439	0.180108
H	-1.335242	-0.743656	-1.345660
H	2.209890	1.231992	-0.282150
H	-1.335668	0.743066	1.345666

14
1,4-Cyclohexadiene (1,4-C_6H_8)

C	1.489963	0.000000	0.000023
C	0.663809	1.245958	-0.000017
C	-0.663809	1.245958	-0.000005
C	-1.489963	0.000000	0.000016
C	0.663809	-1.245958	-0.000020
C	-0.663809	-1.245958	-0.000006
H	1.197770	2.191104	-0.000053
H	-1.197770	2.191105	-0.000024
H	-2.164186	-0.000001	0.867600
H	1.197770	-2.191104	-0.000049
H	-1.197771	-2.191104	-0.000016
H	2.164253	0.000002	-0.867508
H	2.164141	-0.000002	0.867645

H	-2.164209	0.000001	-0.867552

77
Ar1

H	1.362710	-2.319246	-2.294312
Si	-0.509628	1.811859	0.038437
Si	-2.164190	-0.864879	0.024800
Ba	1.335176	-1.200805	-0.274481
N	-0.802924	0.146165	0.059341
C	4.337641	-0.351263	1.502040
C	4.795882	-2.034787	-0.165503
H	4.973842	-3.068997	-0.431462
C	4.579982	-1.681435	1.163380
H	4.604773	-2.440109	1.936329
C	4.771888	-1.059265	-1.159128
C	-0.348699	2.475867	1.818964
H	-1.282130	2.142402	2.287683
C	-0.268018	3.986891	2.019286
C	0.796636	1.767141	2.539824
C	-1.825438	2.808002	-0.907849
H	-2.047135	2.177020	-1.776475
C	-1.412767	4.178312	-1.440632
C	-3.106091	2.925143	-0.080059
C	4.327158	0.625498	0.510482
H	4.147569	1.660691	0.770755
C	1.204054	2.025692	-0.818151
H	1.873127	1.350770	-0.259013
C	1.902093	3.378727	-0.704657
C	1.159340	1.568467	-2.275543
C	4.544378	0.271057	-0.818661
H	4.526714	1.031432	-1.588860
C	-1.491808	-2.662342	-0.134951
H	-0.760477	-2.628947	-0.962564
C	-2.496893	-3.720477	-0.590042
C	-0.793686	-3.152863	1.139768
C	-3.235177	-0.480040	-1.500320
H	-3.343550	0.607498	-1.438877
C	-4.649852	-1.049787	-1.561949
C	-2.456155	-0.773278	-2.781839
C	-3.222667	-0.725170	1.609620
C	-2.375177	-0.549067	2.869348
C	-4.246205	-1.837429	1.836891
H	-3.781209	0.208931	1.461393
H	4.167984	-0.074655	2.535101
H	4.918581	-1.338376	-2.194072
H	-3.928790	3.328664	-0.678431
H	-3.431907	1.963672	0.318143
H	-2.966663	3.594618	0.771418
H	-2.255375	4.666521	-1.941285

H	-1.081823	4.847303	-0.644098
H	-0.603663	4.108621	-2.168317
H	-0.240530	4.238109	3.084944
H	0.625757	4.417791	1.566526
H	-1.127093	4.500861	1.588046
H	2.856163	3.371242	-1.243851
H	1.302467	4.184266	-1.126430
H	2.113467	3.638882	0.332241
H	2.156892	1.525405	-2.724827
H	0.687115	0.591516	-2.419545
H	0.569568	2.265692	-2.873383
H	-4.900450	-1.599542	2.681988
H	-4.880722	-2.018082	0.970323
H	-3.749584	-2.779652	2.080029
H	-2.018451	-4.701684	-0.671908
H	-3.326890	-3.816490	0.110344
H	-2.912870	-3.481169	-1.567168
H	-2.985329	-0.219528	3.716354
H	-1.910925	-1.493531	3.162693
H	-1.575704	0.174018	2.722162
H	-5.153321	-0.729624	-2.480280
H	-4.662010	-2.140977	-1.555515
H	-5.259572	-0.708295	-0.724125
H	-2.980688	-0.386779	-3.661443
H	-1.463928	-0.319994	-2.759171
H	-2.312010	-1.844539	-2.937290
H	0.818926	2.009874	3.607006
H	0.704898	0.680680	2.453098
H	1.767111	2.056579	2.125205
H	-0.138211	-4.011262	0.949415
H	-0.221716	-2.378716	1.668427
H	-1.533397	-3.480232	1.871398

77

Ar2*

H	2.910751	-1.093715	2.097510
H	4.863335	-0.728151	2.215974
Ba	1.641928	-0.766012	0.156750
C	4.098813	0.082002	-1.528765
C	4.213715	0.774127	0.797456
H	4.160129	1.566917	1.532397
C	4.024932	1.072877	-0.540002
H	3.825764	2.099000	-0.826262
C	4.442767	-0.562820	1.238017
C	4.483181	-1.206439	-1.125437
H	4.648321	-1.971859	-1.875775
C	4.682749	-1.517984	0.205818
H	3.984338	0.329458	-2.574476
H	4.998138	-2.517607	0.478107

Si	-1.837636	-1.340876	0.107556
Si	-0.921293	1.691767	-0.026467
N	-0.835732	0.007892	0.053266
C	-3.481334	-1.137630	-0.823961
H	-3.844783	-0.173958	-0.452197
C	-4.558699	-2.172665	-0.506725
C	-3.291981	-0.993226	-2.332828
C	-2.226966	-1.867364	1.897029
C	-0.947176	-2.129404	2.686187
H	-2.804212	-2.798335	1.861559
C	-3.066499	-0.811928	2.610866
C	-0.827378	-2.826892	-0.608308
C	-0.222446	-2.575980	-1.991324
C	-1.578335	-4.156954	-0.602034
H	-0.001087	-2.975772	0.109121
C	0.486679	2.201824	-1.236231
H	1.381505	1.663041	-0.882239
C	0.927623	3.661853	-1.282530
C	0.186229	1.669938	-2.636917
C	-0.542553	2.430728	1.692572
H	-1.299017	1.966664	2.337389
C	-0.683060	3.943748	1.842217
C	0.820749	1.967404	2.200977
C	-2.590905	2.366559	-0.645734
H	-2.892356	1.641831	-1.409991
C	-2.574721	3.742392	-1.308973
C	-3.644005	2.332626	0.462834
H	1.706769	3.810671	-2.038108
H	0.103729	4.329555	-1.533305
H	1.333969	3.990388	-0.325922
H	-0.121258	0.622590	-2.609528
H	-0.630754	2.228085	-3.098614
H	1.053604	1.757577	-3.299041
H	-3.584382	4.032575	-1.618035
H	-2.212122	4.518360	-0.631964
H	-1.945580	3.764824	-2.199048
H	-4.645161	2.518516	0.061409
H	-3.668952	1.376456	0.984604
H	-3.451198	3.101044	1.214316
H	1.057212	2.393611	3.180721
H	0.858645	0.881892	2.338091
H	1.630811	2.269435	1.528605
H	-0.537964	4.248506	2.883875
H	0.052630	4.484403	1.245788
H	-1.667565	4.295833	1.534912
H	-1.159321	-2.385492	3.728786
H	-0.357393	-2.951859	2.273499
H	-0.315190	-1.235350	2.710054
H	-3.285045	-1.103621	3.643087

H	-2.531878	0.140070	2.647802
H	-4.020436	-0.634260	2.110876
H	0.577621	-3.284967	-2.231901
H	-0.983555	-2.687257	-2.763529
H	0.158624	-1.557560	-2.119897
H	-0.924989	-4.989882	-0.880721
H	-2.002851	-4.381521	0.376951
H	-2.398950	-4.137319	-1.320719
H	-5.505720	-1.909756	-0.989113
H	-4.284280	-3.166793	-0.863745
H	-4.748947	-2.253777	0.564367
H	-4.200857	-0.619367	-2.814599
H	-2.478583	-0.307885	-2.578945
H	-3.062624	-1.957060	-2.793459

77

Ar3

H	4.060010	-2.041709	-1.486245
Si	-1.436111	-1.629304	0.079394
Si	-1.313720	1.543735	-0.083123
Ba	1.710674	-0.201759	0.580900
N	-0.828769	-0.063470	0.092801
C	4.150844	1.393914	0.314638
C	4.708793	-0.987580	0.287955
H	5.072398	-1.859197	0.821499
C	4.667524	0.220114	0.925528
H	4.953139	0.261821	1.974426
C	4.526790	-1.087717	-1.212136
C	-2.979480	-1.876346	-0.998651
H	-3.609912	-1.030634	-0.704197
C	-3.784094	-3.147415	-0.734196
C	-2.683402	-1.718400	-2.489156
C	-1.842384	-2.215218	1.851074
C	-0.625899	-2.090334	2.764936
H	-2.131744	-3.271214	1.812525
C	-3.002888	-1.415800	2.436369
C	3.689286	1.270317	-1.019282
H	3.216688	2.128786	-1.488819
C	-0.011197	-2.806646	-0.486463
C	0.688058	-2.386332	-1.779617
C	-0.427877	-4.274051	-0.576466
H	0.736554	-2.782651	0.326715
C	3.687253	0.077491	-1.691529
H	3.264892	0.031444	-2.688722
C	0.061276	2.360119	-1.146793
H	1.012451	2.065603	-0.671394
C	0.136598	3.882393	-1.226329
C	0.066620	1.735597	-2.542309
C	-1.347107	2.378919	1.636872

H	-2.075455	1.776895	2.193953
C	-1.820713	3.829132	1.693678
C	-0.007502	2.221623	2.354398
C	-3.016747	1.762203	-0.902643
H	-3.040507	0.965446	-1.654126
C	-3.266459	3.080129	-1.632866
C	-4.145238	1.490929	0.093348
H	4.203091	2.355040	0.805550
H	5.506452	-1.105527	-1.726477
H	0.934871	4.195813	-1.907037
H	-0.792144	4.318616	-1.593767
H	0.346774	4.328915	-0.254690
H	0.023252	0.646286	-2.494899
H	-0.799118	2.067303	-3.119399
H	0.960969	2.016952	-3.106104
H	-4.272341	3.096548	-2.065070
H	-3.192025	3.939382	-0.963437
H	-2.560881	3.239474	-2.448267
H	-5.110299	1.410189	-0.416469
H	-3.991290	0.570605	0.656873
H	-4.233029	2.300966	0.820700
H	0.003513	2.733657	3.321394
H	0.190770	1.166282	2.569999
H	0.825503	2.639211	1.775254
H	-1.906802	4.173378	2.729541
H	-1.131912	4.505539	1.187240
H	-2.797587	3.955970	1.227369
H	-0.852812	-2.388072	3.793196
H	0.213796	-2.709474	2.435314
H	-0.300635	-1.043748	2.813481
H	-3.235612	-1.729403	3.459080
H	-2.753706	-0.352354	2.468378
H	-3.914312	-1.520131	1.845476
H	1.610074	-2.950295	-1.949392
H	0.039208	-2.562670	-2.637447
H	0.932522	-1.320387	-1.829740
H	0.433753	-4.924602	-0.754998
H	-0.921563	-4.618048	0.333312
H	-1.121634	-4.424756	-1.404868
H	-4.715860	-3.144017	-1.308767
H	-3.233765	-4.043880	-1.023753
H	-4.050138	-3.255932	0.318238
H	-3.605462	-1.610109	-3.068507
H	-2.057957	-0.846987	-2.692319
H	-2.163239	-2.595815	-2.880552

79
Ar4

H	3.677279	1.349653	2.357862
Si	-1.420164	1.637010	-0.074014
Si	-1.354863	-1.535776	0.082587
Ba	1.707791	0.223872	-0.585152
N	-0.840875	0.061802	-0.101931
C	4.386377	-1.066707	-0.602859
C	4.517242	1.167958	0.375642
H	4.741777	2.222261	0.255433
C	4.730602	0.309411	-0.666728
H	5.082439	0.716971	-1.611917
C	4.257851	0.634438	1.767147
C	-2.987169	1.902987	0.964494
H	-3.625467	1.072716	0.644401
C	-3.759335	3.192307	0.691080
C	-2.736429	1.725460	2.461060
C	-1.765686	2.261566	-1.846114
C	-0.530172	2.127130	-2.732475
H	-2.035502	3.322329	-1.797814
C	-2.924181	1.492417	-2.474973
C	3.768751	-1.517300	0.589141
H	3.374487	-2.530405	0.622483
C	0.013575	2.773182	0.555424
C	0.675300	2.295033	1.848423
C	-0.379629	4.244056	0.684887
H	0.780474	2.768421	-0.240457
C	3.524939	-0.682850	1.648994
H	2.985475	-1.057478	2.510909
C	-0.046965	-2.352725	1.223383
H	0.928380	-2.031007	0.822777
C	0.034018	-3.874892	1.289627
C	-0.143960	-1.742855	2.622253
C	-1.338320	-2.391927	-1.627836
H	-2.042773	-1.788945	-2.214434
C	-1.824421	-3.837901	-1.684406
C	0.023646	-2.253419	-2.304291
C	-3.095279	-1.722727	0.830240
H	-3.136693	-0.930307	1.585531
C	-3.399743	-3.040221	1.540964
C	-4.175983	-1.426788	-0.210329
H	4.642530	-1.752103	-1.398188
H	5.206373	0.511133	2.323530
H	0.785115	-4.192213	2.020413
H	-0.914924	-4.323116	1.585049
H	0.314200	-4.307681	0.328956
H	-0.164846	-0.652743	2.581408
H	-1.055160	-2.065149	3.130052
H	0.700551	-2.043186	3.249547

H	-4.420349	-3.036998	1.937582
H	-3.321542	-3.895878	0.867638
H	-2.727124	-3.223156	2.378719
H	-5.160942	-1.334497	0.257915
H	-3.983319	-0.505274	-0.759810
H	-4.244542	-2.230608	-0.946739
H	0.065199	-2.784788	-3.260029
H	0.231862	-1.203841	-2.534602
H	0.837264	-2.660130	-1.690761
H	-1.866034	-4.197337	-2.717942
H	-1.168177	-4.513567	-1.135490
H	-2.823610	-3.946334	-1.263099
H	-0.723719	2.454082	-3.758681
H	0.316645	2.717724	-2.369238
H	-0.230757	1.073635	-2.798798
H	-3.122697	1.824930	-3.498940
H	-2.692568	0.425111	-2.515290
H	-3.849786	1.604693	-1.908145
H	1.611645	2.823466	2.049768
H	0.015635	2.468314	2.698681
H	0.879509	1.219541	1.869970
H	0.488411	4.872705	0.905583
H	-0.844327	4.626018	-0.225059
H	-1.091274	4.379991	1.500435
H	-4.705581	3.201826	1.241388
H	-3.199554	4.075114	1.003489
H	-3.996026	3.315720	-0.366675
H	-3.676963	1.638025	3.013632
H	-2.141599	0.835410	2.674083
H	-2.203482	2.584847	2.874650
H	2.567333	-0.126979	-3.504208
H	3.086793	-0.451440	-3.070072

79

Ar5*

Si	-1.720584	-1.425879	0.000779
Si	-1.044086	1.673898	-0.030542
Ba	1.603436	-0.641359	0.683270
N	-0.833173	0.003892	0.087648
C	4.465538	1.034868	0.269231
C	4.410032	-1.440648	0.082521
H	5.184806	-2.201040	0.185050
C	4.685939	-0.187118	0.789161
H	5.024793	-0.259407	1.819933
C	3.922613	-1.241751	-1.285831
C	-3.260054	-1.344283	-1.108075
H	-3.747875	-0.420466	-0.780089
C	-4.270065	-2.474946	-0.920389
C	-2.908707	-1.170210	-2.584727

C	-2.262368	-1.988108	1.741687
C	-1.064335	-2.067784	2.684631
H	-2.698707	-2.989881	1.659670
C	-3.311209	-1.049117	2.329990
C	4.111613	1.251048	-1.177803
H	4.975552	1.677367	-1.712029
C	-0.513452	-2.803144	-0.596264
C	0.268170	-2.449785	-1.861820
C	-1.162257	-4.177426	-0.754104
H	0.218575	-2.942805	0.218546
C	3.691880	-0.035954	-1.834452
H	3.256659	0.024824	-2.825707
C	0.455862	2.293708	-1.057894
H	1.322874	1.798464	-0.591314
C	0.800147	3.779700	-1.059369
C	0.363210	1.746745	-2.481725
C	-0.959676	2.444421	1.719109
H	-1.786384	1.952258	2.246126
C	-1.186927	3.950403	1.826519
C	0.322017	2.042827	2.446794
C	-2.675868	2.214833	-0.847771
H	-2.829510	1.466189	-1.632375
C	-2.683323	3.584771	-1.523617
C	-3.847692	2.106293	0.128869
H	4.635843	1.914255	0.881585
H	1.645161	3.983769	-1.726192
H	-0.033960	4.392281	-1.401397
H	1.078784	4.132792	-0.066413
H	0.145661	0.677571	-2.485650
H	-0.435528	2.240514	-3.038656
H	1.291942	1.909895	-3.037859
H	-3.664908	3.791964	-1.962303
H	-2.472368	4.390715	-0.818011
H	-1.949191	3.653630	-2.326395
H	-4.804670	2.219744	-0.389890
H	-3.866978	1.150361	0.651819
H	-3.800803	2.887583	0.890619
H	0.405026	2.521729	3.427213
H	0.333974	0.965003	2.639434
H	1.222509	2.325878	1.887524
H	-1.225167	4.267376	2.873960
H	-0.390841	4.521020	1.347616
H	-2.124604	4.254189	1.361732
H	-1.353544	-2.396850	3.687361
H	-0.289209	-2.757432	2.337991
H	-0.622817	-1.070983	2.806598
H	-3.618296	-1.363753	3.332620
H	-2.908868	-0.036530	2.415959
H	-4.209776	-0.992780	1.713412

H	1.107632	-3.132654	-2.024215
H	-0.374066	-2.509279	-2.740775
H	0.655620	-1.425515	-1.865131
H	-0.411628	-4.951402	-0.940135
H	-1.724366	-4.469853	0.133650
H	-1.851927	-4.185016	-1.599881
H	-5.177296	-2.287143	-1.503673
H	-3.869555	-3.434026	-1.251881
H	-4.570081	-2.592016	0.121882
H	-3.785251	-0.877485	-3.171155
H	-2.137896	-0.412051	-2.736114
H	-2.538204	-2.105244	-3.011218
H	2.666732	-2.710124	1.347260
H	3.508584	-2.178413	0.780040
H	3.660848	-2.129149	-1.855574
H	3.331393	2.012479	-1.281538

79
Ar6*

H	-3.691266	2.094130	-0.720004
Si	1.511160	1.582883	0.058936
Si	1.262332	-1.580153	-0.051542
Ba	-1.693858	0.357660	0.579489
N	0.827800	0.044262	0.095660
C	-3.945340	-1.566991	-0.120074
C	-4.720682	0.693227	0.532987
H	-5.636982	1.097122	0.960005
C	-4.577926	-0.729252	0.750691
H	-4.953591	-1.129319	1.687758
C	-4.389542	1.234113	-0.865266
C	3.094143	1.731807	-0.979099
H	3.689164	0.876251	-0.642891
C	3.932009	2.985144	-0.732010
C	2.830456	1.540987	-2.471833
C	1.886296	2.192101	1.827544
C	0.646658	2.090626	2.713142
H	2.183090	3.245634	1.775008
C	3.025183	1.396752	2.459215
C	-3.563800	-1.065678	-1.423364
H	-3.122814	-1.751176	-2.136755
C	0.154540	2.792787	-0.584834
C	-0.540442	2.340446	-1.870231
C	0.627370	4.238283	-0.731908
H	-0.611470	2.838068	0.207975
C	-3.775751	0.216420	-1.778721
H	-3.529760	0.536598	-2.784869
C	-0.074341	-2.356298	-1.190134
H	-1.028631	-1.988507	-0.781555
C	-0.224027	-3.873482	-1.243504

C	0.048588	-1.762563	-2.593355
C	1.184755	-2.400301	1.675375
H	1.917789	-1.824359	2.253950
C	1.591246	-3.869063	1.763290
C	-0.170879	-2.177206	2.343271
C	2.998007	-1.874985	-0.776730
H	3.092598	-1.094638	-1.539549
C	3.232771	-3.214789	-1.471901
C	4.084679	-1.630537	0.270889
H	-3.823580	-2.617696	0.105824
H	-5.253232	1.692144	-1.357860
H	-0.991745	-4.163612	-1.969115
H	0.701580	-4.367562	-1.539277
H	-0.519661	-4.285220	-0.278290
H	0.126150	-0.674699	-2.560617
H	0.940605	-2.134345	-3.101514
H	-0.810244	-2.024387	-3.219995
H	4.256548	-3.274952	-1.855609
H	3.096375	-4.058480	-0.792660
H	2.560963	-3.365160	-2.316861
H	5.077710	-1.606809	-0.188763
H	3.944755	-0.690064	0.803536
H	4.096443	-2.425299	1.020170
H	-0.244046	-2.692583	3.305901
H	-0.321334	-1.114761	2.561956
H	-1.001897	-2.546531	1.729811
H	1.619398	-4.207190	2.804431
H	0.895265	-4.519573	1.233040
H	2.580055	-4.041857	1.339124
H	0.841382	2.442073	3.730930
H	-0.200944	2.670975	2.337112
H	0.344319	1.040252	2.806359
H	3.234595	1.732634	3.479964
H	2.762688	0.337020	2.512549
H	3.953165	1.475756	1.890503
H	-1.467168	2.894448	-2.049291
H	0.105055	2.500532	-2.734385
H	-0.769477	1.269229	-1.891178
H	-0.209834	4.910555	-0.942647
H	1.122117	4.600633	0.170141
H	1.334475	4.332861	-1.557773
H	4.880474	2.932228	-1.276219
H	3.419788	3.887400	-1.069086
H	4.168418	3.122198	0.324078
H	3.763103	1.395332	-3.025803
H	2.188919	0.679818	-2.667873
H	2.340594	2.419546	-2.898215
H	-3.043759	2.205197	1.390365
H	-3.897402	1.403843	1.222997

79

Ar7 (N*BaH·1,4-C_6H_8)

H	3.862420	-2.442792	0.120231
Si	-1.876000	-1.310030	-0.007357
Si	-0.906251	1.715510	-0.012075
Ba	1.476415	-0.881617	0.689926
N	-0.864420	0.035934	0.108863
C	4.309713	1.267639	-0.777856
C	4.862838	-0.645795	0.721633
H	5.206678	-1.015292	1.682366
C	4.744047	0.667273	0.524570
H	5.000379	1.354034	1.324743
C	4.530206	-1.670864	-0.316151
C	-3.422969	-1.066680	-1.082015
H	-3.834324	-0.114919	-0.729497
C	-4.511778	-2.123359	-0.901390
C	-3.077181	-0.895143	-2.560115
C	-2.423732	-1.863772	1.733161
C	-1.212715	-2.031930	2.648447
H	-2.922515	-2.835450	1.644377
C	-3.397652	-0.869759	2.358716
C	3.786346	0.241254	-1.735621
H	3.310820	0.604959	-2.639580
C	-0.791728	-2.751749	-0.664977
C	0.032116	-2.408930	-1.907465
C	-1.543509	-4.063622	-0.883900
H	-0.071749	-2.992588	0.139266
C	3.905322	-1.071928	-1.536970
H	3.523552	-1.760967	-2.283380
C	0.588081	2.178395	-1.129651
H	1.407376	1.549909	-0.744682
C	1.117338	3.608131	-1.113202
C	0.323674	1.700785	-2.556811
C	-0.666107	2.481583	1.726957
H	-1.514881	2.072602	2.289204
C	-0.745380	4.002609	1.830549
C	0.597005	1.964357	2.413067
C	-2.510864	2.424070	-0.752495
H	-2.762495	1.710884	-1.544889
C	-2.420416	3.804782	-1.400325
C	-3.649014	2.397057	0.268307
H	3.559842	2.049056	-0.611515
H	5.428684	-2.237754	-0.590496
H	1.938392	3.732652	-1.828509
H	0.346801	4.330874	-1.382801
H	1.495331	3.890169	-0.129688
H	-0.008239	0.661866	-2.572845
H	-0.455810	2.296837	-3.034611
H	1.217842	1.784009	-3.183566

H	-3.393063	4.104308	-1.804222
H	-2.121368	4.574378	-0.686336
H	-1.706973	3.830767	-2.223905
H	-4.610433	2.614638	-0.207320
H	-3.737524	1.431240	0.765181
H	-3.499715	3.147657	1.047871
H	0.771532	2.458086	3.374069
H	0.504170	0.897497	2.639955
H	1.497452	2.133917	1.809510
H	-0.692844	4.327677	2.874968
H	0.069767	4.494959	1.299426
H	-1.677263	4.387746	1.418205
H	-1.498345	-2.384637	3.644152
H	-0.471736	-2.741389	2.265021
H	-0.731283	-1.056957	2.799133
H	-3.705459	-1.182192	3.361913
H	-2.925647	0.111735	2.454997
H	-4.302275	-0.740567	1.762131
H	0.854232	-3.117735	-2.050551
H	-0.584013	-2.436391	-2.807135
H	0.446556	-1.394101	-1.884360
H	-0.850432	-4.884647	-1.089843
H	-2.134103	-4.345229	-0.011037
H	-2.223742	-3.988701	-1.734640
H	-5.408855	-1.859975	-1.471196
H	-4.184851	-3.103028	-1.252713
H	-4.808546	-2.236355	0.142232
H	-3.941911	-0.548745	-3.135008
H	-2.268119	-0.178173	-2.711513
H	-2.760253	-1.843797	-2.999394
H	1.994551	-3.115073	1.158391
H	5.153746	1.800877	-1.236703

79

Ar8 (N*BaH·1,3-C_6H_8)

Ba	1.594233	-0.355770	0.817685
Si	-1.526257	-1.600135	0.010312
Si	-1.303058	1.565831	-0.077152
N	-0.859389	-0.051614	0.102034
C	4.173059	1.572158	-0.182583
C	4.974806	-0.705187	0.393891
C	4.825172	0.757924	0.664310
C	4.844408	-1.076828	-1.088462
C	-3.010510	-1.764093	-1.162600
C	-3.838592	-3.036648	-0.991760
C	-2.613257	-1.568809	-2.624764
C	-2.047320	-2.198983	1.744073
C	-0.892703	-2.047326	2.732160
C	-3.264272	-1.440236	2.264202

C	3.609637	1.049519	-1.426364
C	-0.101956	-2.781430	-0.503696
C	0.697387	-2.319999	-1.723841
C	-0.518765	-4.240909	-0.676977
C	3.891087	-0.194266	-1.839340
C	0.106794	2.365169	-1.111184
C	0.244677	3.883920	-1.138616
C	0.082786	1.783960	-2.524355
C	-1.378806	2.394814	1.646859
C	-1.830002	3.852491	1.696576
C	-0.076362	2.212149	2.423976
C	-2.976967	1.842039	-0.942264
C	-3.175192	3.183775	-1.645681
C	-4.144835	1.573087	0.007496
H	4.534610	-2.121714	-1.166967
H	5.927930	-1.061136	0.788463
H	5.226202	1.150387	1.592794
H	-3.651611	-0.921814	-0.882913
H	-2.301781	-3.262751	1.676005
H	2.971455	1.691896	-2.020109
H	0.600618	-2.805952	0.349308
H	3.485345	-0.557442	-2.776664
H	1.031028	1.995440	-0.637922
H	-2.139103	1.800560	2.168829
H	-2.995837	1.066075	-1.715150
H	4.053541	2.625511	0.041726
H	5.817096	-1.014578	-1.593616
H	1.060864	4.191122	-1.802057
H	-0.661815	4.370889	-1.498331
H	0.460830	4.288521	-0.149523
H	0.043326	0.693848	-2.505790
H	-0.793464	2.128473	-3.076601
H	0.961564	2.085385	-3.104818
H	-4.164259	3.229662	-2.113199
H	-3.111295	4.023993	-0.951727
H	-2.438530	3.353622	-2.430879
H	-5.094342	1.540277	-0.535915
H	-4.037891	0.629613	0.541784
H	-4.232883	2.360256	0.759638
H	-0.096363	2.735421	3.384955
H	0.085384	1.156160	2.663512
H	0.790598	2.601668	1.876114
H	-1.935791	4.194923	2.731357
H	-1.119063	4.519921	1.208628
H	-2.793428	3.995922	1.208258
H	-1.155735	-2.416909	3.728015
H	0.013841	-2.580341	2.427852
H	-0.654808	-0.983236	2.855728
H	-3.557443	-1.782900	3.261902

H	-3.040715	-0.372920	2.341114
H	-4.132379	-1.546741	1.611468
H	1.673693	-2.813289	-1.774066
H	0.168010	-2.549951	-2.649430
H	0.853349	-1.235368	-1.753190
H	0.354969	-4.888296	-0.797510
H	-1.082782	-4.608972	0.181067
H	-1.143454	-4.367034	-1.563471
H	-4.732820	-3.007591	-1.622864
H	-3.274421	-3.926069	-1.275876
H	-4.169441	-3.178564	0.038038
H	-3.492806	-1.447988	-3.265103
H	-1.977638	-0.692078	-2.762993
H	-2.062337	-2.434959	-2.998500
H	2.571518	-2.373750	1.494732
H	4.225903	-1.290293	0.978715

XYZ-Files for hydride-ethylene insertion (dimeric catalysts $[HCaN(TRIP)_2]_2$, $[HBaN(TRIP)_2]_2$, and $[HCaN'']_2$)

	64		
Ca1D'			
Ca	1.897055	-1.487702	-0.541753
H	0.274669	-0.678102	-1.681348
Ca	-1.092970	-0.108465	-0.054564
H	0.350621	-1.461787	0.932797
H	-0.220527	0.279125	3.389319
C	-1.246405	0.248513	3.010655
C	-1.878284	1.341936	2.568484
H	-1.739530	-0.729359	3.023909
H	-2.904097	1.289230	2.190454
H	-1.390945	2.322589	2.568838
Si	-3.780186	1.250467	-1.058319
Si	-4.231806	-1.488553	0.337594
N	-3.298964	-0.186478	-0.272382
C	-2.181454	2.294857	-1.254961
C	-5.011014	2.303665	-0.085094
C	-4.470736	1.000414	-2.795332
C	-3.062134	-2.956920	0.613960
C	-5.608452	-2.045192	-0.830711
C	-5.037607	-1.076064	2.001212
Si	3.241709	1.539822	0.381355
Si	4.994264	-0.924138	-0.375211
N	3.538309	-0.071952	-0.119319
C	1.348010	1.772809	0.407432
C	3.953700	2.835657	-0.789904
C	3.861131	1.906250	2.126329
C	4.462213	-2.707320	-0.891875
C	6.076210	-1.125367	1.155166
C	6.058384	-0.269574	-1.786498
H	6.934115	-1.792290	0.971025
H	6.474423	-0.147907	1.471255
H	5.493045	-1.531092	1.996900
H	6.919997	-0.924750	-1.994115
H	5.466043	-0.169415	-2.709758
H	6.449620	0.729656	-1.537201
H	3.571593	2.915819	2.461193
H	3.454980	1.174235	2.842707
H	4.959912	1.840614	2.173517
H	3.660703	3.857268	-0.497626
H	5.054741	2.792583	-0.795878
H	3.608444	2.658742	-1.820938
H	1.049679	2.788278	0.709352
H	0.948755	1.587514	-0.605837
H	0.924157	1.062370	1.139940

H	3.903260	-3.255818	-0.106963
H	3.899741	-2.751771	-1.846530
H	5.361174	-3.317438	-1.071506
H	-3.583476	-3.812588	1.072582
H	-2.639613	-3.300509	-0.345520
H	-2.216008	-2.703498	1.277578
H	-6.133014	-2.935705	-0.447404
H	-6.357606	-1.246690	-0.959116
H	-5.202648	-2.286078	-1.826128
H	-5.634080	-1.918331	2.388901
H	-4.281640	-0.823462	2.762356
H	-5.707514	-0.206846	1.900646
H	-4.679441	1.958068	-3.299778
H	-3.758129	0.430953	-3.412973
H	-5.409478	0.425625	-2.757704
H	-5.234413	3.254126	-0.596672
H	-5.961536	1.758970	0.036820
H	-4.633912	2.538557	0.922894
H	-2.413347	3.251894	-1.747711
H	-1.710957	2.567873	-0.291075
H	-1.421965	1.813341	-1.902007

	64		
Ca2D'*			
Ca	-1.637061	0.570416	-1.073898
H	-0.000115	-0.028451	-2.336568
Ca	1.636790	0.570111	-1.073580
H	-0.000107	1.981033	-0.428332
H	-0.911182	1.686468	1.379909
C	-0.000454	1.149185	1.089793
C	-0.000033	-0.231945	0.986396
H	0.909701	1.687064	1.380545
H	0.931340	-0.799529	1.083871
H	-0.931132	-0.800067	1.083280
Si	3.867589	-1.721449	0.058893
Si	4.428971	1.366098	0.194317
N	3.557182	-0.050955	-0.186671
C	2.551515	-2.675796	-0.926752
C	3.719008	-2.226810	1.873542
C	5.563124	-2.273813	-0.560082
C	3.431335	2.809897	-0.593428
C	6.159296	1.457621	-0.547632
C	4.547671	1.725992	2.042802
Si	-3.867267	-1.721599	0.059007
Si	-4.429066	1.365898	0.194312
N	-3.557086	-0.051060	-0.186526
C	-2.551333	-2.675698	-0.927063
C	-5.562921	-2.274053	-0.559560
C	-3.718234	-2.226996	1.873606

C	-3.431708	2.809725	-0.593796
C	-4.547583	1.726184	2.042732
C	-6.159498	1.457127	-0.547422
H	-5.023604	2.698471	2.249673
H	-5.144124	0.946418	2.543201
H	-3.549787	1.723171	2.510237
H	-6.627444	2.440218	-0.376125
H	-6.130115	1.270506	-1.632563
H	-6.811600	0.691658	-0.098161
H	-3.864397	-3.310861	2.011244
H	-2.729910	-1.963034	2.283230
H	-4.474792	-1.705068	2.481573
H	-5.680649	-3.367932	-0.491679
H	-6.367366	-1.814056	0.036498
H	-5.713462	-1.976584	-1.609494
H	-2.656712	-3.763174	-0.784153
H	-2.643675	-2.484508	-2.010307
H	-1.517839	-2.424441	-0.627366
H	-2.402436	2.948971	-0.207434
H	-3.386968	2.737261	-1.698072
H	-3.936996	3.765962	-0.385812
H	3.936425	3.766201	-0.385270
H	3.386569	2.737605	-1.697709
H	2.402042	2.948833	-0.207026
H	6.627490	2.440460	-0.375559
H	6.811278	0.691609	-0.099129
H	6.129713	1.271948	-1.632932
H	5.023624	2.698280	2.249902
H	3.549930	1.722782	2.510425
H	5.144354	0.946174	2.543017
H	5.680870	-3.367697	-0.492319
H	5.713443	-1.976257	-1.610025
H	6.367692	-1.813863	0.035847
H	3.865311	-3.310658	2.011171
H	4.475639	-1.704794	2.481340
H	2.730741	-1.962942	2.283365
H	2.657197	-3.763249	-0.783894
H	1.518088	-2.424789	-0.626631
H	2.643408	-2.484586	-2.010037

64

Ca3D'

Ca	1.207940	0.319060	-0.312774
Ca	-1.592409	-1.320643	-0.327088
H	-0.097644	-0.697971	-1.727021
C	-0.014745	-0.773127	1.574886
C	-1.040428	-1.677820	2.261960
H	-0.036082	0.207533	2.099368
H	0.995899	-1.182151	1.787223

H	-0.907576	-1.809697	3.350279
H	-2.077253	-1.294393	2.151274
H	-1.026450	-2.714210	1.857678
N	-3.280784	0.103527	-0.216731
Si	-4.679710	-0.865129	-0.085689
Si	-3.093735	1.805082	-0.225060
C	-3.694988	2.629711	1.362868
H	-4.785867	2.517610	1.470867
H	-3.466883	3.707992	1.379568
H	-3.225232	2.163359	2.243681
C	-3.935364	2.643782	-1.690659
H	-3.590822	2.202743	-2.639613
H	-3.729251	3.726261	-1.717440
H	-5.027938	2.511712	-1.641398
C	-5.542865	-0.777223	1.588968
H	-4.832887	-0.975588	2.407614
H	-6.371921	-1.499273	1.666923
H	-5.958127	0.229965	1.752127
C	-5.963240	-0.602682	-1.440382
H	-6.418957	0.395948	-1.346405
H	-6.774902	-1.346474	-1.388895
H	-5.498512	-0.661080	-2.437172
C	-1.227152	2.189778	-0.366950
H	-0.731629	1.840332	0.555202
H	-1.026884	3.266917	-0.470169
H	-0.828808	1.678060	-1.262659
C	-4.074181	-2.689533	-0.270235
H	-3.606111	-2.905520	-1.251188
H	-4.943681	-3.362966	-0.216870
H	-3.406578	-3.034853	0.544249
N	3.391073	0.001826	-0.185665
Si	4.101447	1.492612	0.237467
Si	4.047942	-1.564924	-0.420467
C	5.120219	-2.150278	1.021607
H	6.031527	-1.536512	1.107100
H	5.435293	-3.199068	0.894813
H	4.572411	-2.067874	1.973950
C	5.078614	-1.691401	-1.999012
H	4.480006	-1.398270	-2.876499
H	5.455196	-2.713985	-2.165994
H	5.947592	-1.015542	-1.950324
C	4.918330	1.525044	1.938023
H	4.219694	1.167591	2.711173
H	5.255426	2.537245	2.215334
H	5.796915	0.861018	1.955993
C	5.326096	2.171250	-1.026718
H	6.210940	1.516971	-1.085411
H	5.674407	3.184270	-0.767088
H	4.874266	2.204549	-2.030797

C	2.594614	-2.780906	-0.585123
H	2.023213	-2.847332	0.356362
H	2.951828	-3.795071	-0.826233
H	1.900574	-2.480381	-1.390515
C	2.646281	2.750878	0.325877
H	2.107537	2.886185	-0.632472
H	3.033990	3.750461	0.577177
H	1.910241	2.523865	1.122034

	136		
Ca1D			
Ca	-1.632180	-0.906640	-1.233216
H	-0.077935	0.552200	-1.095511
Ca	1.273457	-0.344802	0.286903
H	-0.019352	-1.982628	-0.288493
H	-1.425847	-4.269522	-1.317289
C	-1.071072	-3.785517	-2.232378
C	-1.919786	-3.331779	-3.163827
H	0.013362	-3.700975	-2.341075
H	-1.561918	-2.868636	-4.089573
H	-3.003733	-3.442006	-3.054824
Si	4.215264	-1.467395	-0.212546
Si	3.818774	1.680480	0.425022
N	3.483014	0.030786	0.138268
C	2.998923	-2.404036	-1.387538
H	2.107006	-2.626647	-0.762641
C	2.491692	-1.572294	-2.567105
H	2.061230	-0.603093	-2.262062
H	1.708567	-2.109092	-3.132096
H	3.298510	-1.334482	-3.276032
C	3.519694	-3.763011	-1.856882
H	4.363596	-3.648283	-2.555124
H	2.739841	-4.336026	-2.389353
H	3.868956	-4.386627	-1.019716
C	4.225181	-2.518056	1.397094
H	4.490329	-3.557259	1.129823
C	2.829257	-2.530416	2.026445
H	2.563308	-1.510370	2.367013
H	2.780447	-3.165070	2.927609
H	2.042811	-2.899395	1.344021
C	5.238866	-1.995210	2.414370
H	6.267902	-2.002199	2.024947
H	5.234595	-2.592251	3.343273
H	5.003439	-0.955270	2.691516
C	5.974676	-1.395614	-0.950411
H	6.476871	-0.630887	-0.329880
C	6.778689	-2.691981	-0.826312
H	6.841986	-3.051134	0.211527
H	7.812347	-2.552867	-1.188567

H	6.336917	-3.503724	-1.424320
C	5.980181	-0.885728	-2.393346
H	5.576499	-1.643205	-3.084443
H	7.004371	-0.655185	-2.734263
H	5.376698	0.025289	-2.515898
C	2.070612	2.442040	0.768536
H	1.420557	2.035598	-0.040130
C	1.513815	1.988090	2.126222
H	1.723259	0.928416	2.376448
H	0.427305	2.156975	2.215369
H	1.993403	2.544387	2.945184
C	1.928094	3.957728	0.622888
H	0.892215	4.280874	0.822916
H	2.183141	4.288700	-0.393317
H	2.583480	4.500158	1.320233
C	4.604203	2.483705	-1.117280
H	5.468837	1.818724	-1.304277
C	3.663501	2.354429	-2.316617
H	3.296456	1.323449	-2.427739
H	4.162515	2.639341	-3.259231
H	2.778964	3.004315	-2.210786
C	5.149563	3.904153	-0.972317
H	4.361071	4.629666	-0.720242
H	5.613307	4.248069	-1.913718
H	5.918719	3.973311	-0.189056
C	4.916384	1.957827	1.970075
H	4.489096	1.247783	2.704468
C	6.359646	1.526662	1.704584
H	6.414633	0.504013	1.305941
H	6.966113	1.551633	2.626686
H	6.851813	2.189931	0.974761
C	4.872688	3.352570	2.596643
H	5.227605	4.128676	1.901469
H	5.516984	3.404310	3.492051
H	3.859102	3.639921	2.912249
Si	-3.883033	1.336429	-0.979919
Si	-3.789392	-0.877637	1.340507
N	-3.398909	-0.062702	-0.117777
C	-2.775273	1.318553	-2.579052
H	-1.735191	1.202785	-2.193684
C	-3.105701	0.155382	-3.525144
H	-3.357584	-0.795762	-3.015119
H	-2.299688	-0.042382	-4.255317
H	-4.009834	0.382684	-4.109106
C	-2.701838	2.625493	-3.371690
H	-3.687177	2.930128	-3.754098
H	-2.028428	2.525116	-4.240678
H	-2.314956	3.450293	-2.756999
C	-3.469923	3.004544	-0.134021

H	-3.402215	3.727001	-0.967581
C	-2.095294	2.931832	0.533689
H	-2.125116	2.252842	1.400712
H	-1.767652	3.920323	0.899705
H	-1.315542	2.546503	-0.143408
C	-4.528272	3.539339	0.830466
H	-5.511705	3.655980	0.350289
H	-4.239097	4.528314	1.226959
H	-4.664912	2.875378	1.696235
C	-5.735490	1.347636	-1.453907
H	-6.227829	1.530283	-0.480213
C	-6.141074	2.489484	-2.386634
H	-5.767527	3.467484	-2.045111
H	-7.238625	2.568575	-2.474408
H	-5.752825	2.329491	-3.405444
C	-6.245156	0.000526	-1.965467
H	-5.892352	-0.203154	-2.988772
H	-7.348048	-0.030732	-1.997540
H	-5.901358	-0.828050	-1.330515
C	-2.121273	-1.645002	1.927127
H	-1.727967	-2.168132	1.029936
C	-1.103656	-0.549948	2.241351
H	-1.048203	0.195702	1.426122
H	-0.102025	-0.984600	2.439377
H	-1.367159	0.020546	3.145768
C	-2.146668	-2.707961	3.023936
H	-1.127851	-3.079006	3.234754
H	-2.751474	-3.577396	2.731283
H	-2.557995	-2.327048	3.969802
C	-5.018456	-2.295418	0.956884
H	-5.869092	-1.753983	0.502466
C	-4.443256	-3.215509	-0.119566
H	-4.143486	-2.635687	-1.008742
H	-5.173714	-3.971241	-0.456849
H	-3.557763	-3.764607	0.244726
C	-5.571923	-3.087895	2.140969
H	-4.793592	-3.676611	2.648742
H	-6.351102	-3.799532	1.815695
H	-6.026842	-2.432943	2.898373
C	-4.516240	0.262588	2.690605
H	-3.942027	1.197489	2.554806
C	-5.988082	0.576468	2.413451
H	-6.156967	0.896393	1.375198
H	-6.361355	1.381644	3.069523
H	-6.625548	-0.304734	2.588132
C	-4.319857	-0.185987	4.139206
H	-4.793316	-1.160045	4.339818
H	-4.768359	0.539872	4.839985
H	-3.256979	-0.278223	4.406962

	136		
Ca2D*			
Ca	-1.743863	-0.816120	-1.473210
H	-0.113262	0.560564	-1.285549
Ca	1.296480	-0.360313	-0.003360
H	-0.020459	-2.017070	-0.848667
H	-0.508418	-3.925719	-0.848535
C	-0.482480	-3.314088	-1.755604
C	-1.633199	-3.156573	-2.513718
H	0.497113	-3.296343	-2.240014
H	-1.567173	-2.871966	-3.569763
H	-2.594900	-3.536598	-2.159539
Si	4.274536	-1.391838	-0.333859
Si	3.782610	1.683495	0.543373
N	3.491821	0.059914	0.093346
C	3.117950	-2.243488	-1.631019
H	2.207131	-2.526833	-1.057433
C	2.656767	-1.331449	-2.769801
H	2.221274	-0.376847	-2.426263
H	1.897530	-1.821745	-3.403394
H	3.496143	-1.051802	-3.422575
C	3.672706	-3.560575	-2.175685
H	4.551218	-3.383099	-2.815210
H	2.928483	-4.093543	-2.792642
H	3.984329	-4.244174	-1.371471
C	4.234938	-2.578275	1.176500
H	4.552993	-3.580568	0.836407
C	2.808918	-2.690273	1.721186
H	2.474459	-1.706819	2.104596
H	2.737830	-3.383760	2.576242
H	2.082386	-3.049312	0.970454
C	5.174099	-2.114406	2.289445
H	6.220476	-2.050555	1.955366
H	5.147189	-2.793902	3.159162
H	4.885017	-1.112980	2.645684
C	6.058273	-1.231872	-0.989921
H	6.516568	-0.509554	-0.289627
C	6.882425	-2.520074	-0.930253
H	6.912469	-2.954665	0.079860
H	7.926221	-2.335983	-1.238748
H	6.481130	-3.292176	-1.604519
C	6.114787	-0.607918	-2.386142
H	5.759557	-1.314554	-3.153498
H	7.147520	-0.331838	-2.660202
H	5.497814	0.299146	-2.462299
C	2.003263	2.380914	0.851280
H	1.403579	2.052479	-0.029118
C	1.383759	1.778993	2.120560

H	1.580430	0.696203	2.253405
H	0.294968	1.942814	2.176475
H	1.825723	2.235023	3.018858
C	1.843876	3.902029	0.853077
H	0.794782	4.191783	1.031302
H	2.144561	4.338623	-0.109449
H	2.454834	4.376214	1.634983
C	4.635057	2.628482	-0.876935
H	5.519806	1.994242	-1.076671
C	3.760772	2.598576	-2.131838
H	3.421231	1.577196	-2.359752
H	4.302337	2.977826	-3.015696
H	2.859553	3.222967	-2.014014
C	5.146031	4.036953	-0.574032
H	4.331392	4.726255	-0.304073
H	5.655631	4.470402	-1.452476
H	5.868338	4.044944	0.255482
C	4.790191	1.831848	2.164031
H	4.340194	1.049287	2.805072
C	6.253860	1.450078	1.935869
H	6.350953	0.472961	1.442266
H	6.809924	1.392159	2.887616
H	6.770465	2.189737	1.302950
C	4.686216	3.160151	2.915646
H	5.054520	4.005300	2.314219
H	5.287973	3.137985	3.841207
H	3.653163	3.397174	3.209284
Si	-4.011260	1.353407	-0.910293
Si	-3.743075	-1.009505	1.237785
N	-3.449585	-0.082256	-0.169308
C	-3.070089	1.410608	-2.614159
H	-1.992774	1.339875	-2.335292
C	-3.437275	0.248387	-3.550633
H	-3.604351	-0.724583	-3.047268
H	-2.695082	0.098564	-4.355677
H	-4.398431	0.452246	-4.044257
C	-3.145905	2.733360	-3.379621
H	-4.179258	2.975341	-3.668679
H	-2.547064	2.693279	-4.306027
H	-2.764503	3.572953	-2.781558
C	-3.484460	2.974196	-0.033739
H	-3.537478	3.749061	-0.820370
C	-2.030960	2.873084	0.430572
H	-1.935771	2.107035	1.217212
H	-1.671200	3.827868	0.851684
H	-1.342019	2.580593	-0.377708
C	-4.388726	3.434218	1.109299
H	-5.441623	3.529509	0.803540
H	-4.069489	4.418390	1.494615

H	-4.356401	2.734458	1.956489
C	-5.903360	1.395883	-1.172414
H	-6.276878	1.438315	-0.131307
C	-6.424927	2.638866	-1.894502
H	-6.015113	3.573354	-1.481105
H	-7.525139	2.706780	-1.835836
H	-6.166460	2.614999	-2.965186
C	-6.462261	0.115788	-1.793740
H	-6.241798	0.062196	-2.871514
H	-7.559818	0.060914	-1.690994
H	-6.034301	-0.782203	-1.326957
C	-2.067455	-1.929662	1.530435
H	-1.808467	-2.346155	0.534940
C	-0.976780	-0.922156	1.889145
H	-0.968337	-0.070197	1.181991
H	0.017879	-1.411710	1.938196
H	-1.136861	-0.474255	2.881579
C	-2.045707	-3.127920	2.476853
H	-1.032933	-3.564438	2.543465
H	-2.720652	-3.924079	2.133535
H	-2.351653	-2.856461	3.497595
C	-5.130068	-2.290159	0.934606
H	-5.979017	-1.647097	0.634295
C	-4.793771	-3.182538	-0.259352
H	-4.528406	-2.582610	-1.143961
H	-5.642396	-3.829549	-0.541208
H	-3.941436	-3.848517	-0.041278
C	-5.592236	-3.115321	2.136288
H	-4.801205	-3.780977	2.513135
H	-6.449938	-3.757429	1.869309
H	-5.911359	-2.483118	2.977520
C	-4.178502	0.042901	2.775526
H	-3.543271	0.939656	2.649327
C	-5.639067	0.498622	2.725750
H	-5.907641	0.929161	1.750500
H	-5.851248	1.264865	3.491176
H	-6.327238	-0.341274	2.909453
C	-3.851161	-0.564476	4.140594
H	-4.391050	-1.508468	4.313540
H	-4.136633	0.122795	4.956307
H	-2.778530	-0.777031	4.260809

136

Ca3D

Ca	1.319480	0.180512	0.250175
Ca	-1.666275	0.932934	-0.858005
H	-0.406337	1.367573	0.795978
C	0.403695	0.207830	-2.119235
C	-0.055355	1.354553	-3.031567

H	0.151889	-0.753578	-2.607341
H	1.512493	0.215070	-2.156556
H	0.354658	1.330810	-4.057023
H	-1.156318	1.371700	-3.210118
H	0.208845	2.348916	-2.622147
Si	-3.595516	-1.706015	-0.497486
Si	-4.539392	1.185917	0.546578
N	-3.578190	-0.029052	-0.179927
C	-1.765667	-2.313450	-0.368311
H	-1.247898	-1.777592	-1.188945
C	-1.044580	-1.922261	0.921256
H	-1.128043	-0.844457	1.156396
H	0.025805	-2.205185	0.872302
H	-1.446875	-2.443731	1.801060
C	-1.585333	-3.803401	-0.658302
H	-2.015008	-4.421159	0.145839
H	-0.518676	-4.073838	-0.738021
H	-2.072043	-4.103572	-1.598276
C	-4.059503	-2.000608	-2.336688
H	-3.923217	-3.076097	-2.552146
C	-3.129941	-1.209514	-3.257347
H	-3.227709	-0.125220	-3.057483
H	-3.385457	-1.345678	-4.322149
H	-2.070324	-1.487041	-3.143948
C	-5.513289	-1.627901	-2.623899
H	-6.224783	-2.199664	-2.009332
H	-5.777917	-1.803039	-3.681378
H	-5.686914	-0.560762	-2.414636
C	-4.740849	-2.750180	0.621178
H	-5.656812	-2.135642	0.692218
C	-5.139638	-4.106985	0.035949
H	-5.617447	-4.015881	-0.950485
H	-5.851676	-4.630091	0.697976
H	-4.268867	-4.769982	-0.084973
C	-4.193031	-2.911692	2.041021
H	-3.339110	-3.608207	2.060379
H	-4.956829	-3.326669	2.721130
H	-3.852055	-1.958067	2.469519
C	-3.482768	2.797211	0.298680
H	-2.466642	2.518383	0.663124
C	-3.407890	3.204354	-1.182940
H	-3.355947	2.354631	-1.894615
H	-2.572915	3.895478	-1.399997
H	-4.330355	3.723214	-1.482316
C	-3.842095	4.008577	1.161068
H	-3.173210	4.861398	0.951003
H	-3.749218	3.778201	2.231194
H	-4.872708	4.348295	0.984657
C	-4.767241	0.845570	2.409127

H	-5.174149	-0.183442	2.404414
C	-3.407726	0.795167	3.108988
H	-2.694859	0.161759	2.560200
H	-3.492695	0.399737	4.135993
H	-2.952357	1.796290	3.184994
C	-5.763581	1.722378	3.167635
H	-5.459897	2.780321	3.182648
H	-5.853032	1.399895	4.219849
H	-6.771363	1.679041	2.729111
C	-6.230633	1.426635	-0.318291
H	-5.974051	1.333413	-1.391023
C	-7.191306	0.285835	0.022466
H	-6.739148	-0.699451	-0.155072
H	-8.110728	0.337370	-0.586039
H	-7.499651	0.319436	1.079819
C	-6.912079	2.782532	-0.124015
H	-7.128607	2.989438	0.935379
H	-7.875638	2.819404	-0.661990
H	-6.302735	3.617204	-0.500511
Si	4.172715	1.692145	-0.234828
Si	4.020655	-1.483673	0.451184
N	3.575596	0.130485	0.109880
C	2.608879	2.841134	-0.186844
H	1.809136	2.296245	-0.739939
C	2.118738	3.090227	1.242840
H	2.073724	2.176029	1.862867
H	1.114318	3.541848	1.265986
H	2.808926	3.766139	1.769549
C	2.737850	4.165411	-0.942232
H	3.538748	4.791901	-0.522477
H	1.803206	4.750649	-0.883782
H	2.966250	4.012140	-2.006798
C	4.923855	1.908708	-1.980615
H	5.046548	3.000147	-2.111448
C	3.964668	1.408720	-3.059416
H	3.787580	0.327610	-2.950127
H	4.365459	1.576604	-4.074159
H	2.981810	1.902666	-3.009329
C	6.303270	1.269989	-2.137011
H	7.036595	1.677261	-1.424322
H	6.708149	1.431155	-3.151366
H	6.260633	0.181436	-1.976663
C	5.492160	2.322555	1.001441
H	6.384268	1.738375	0.707771
C	5.854748	3.798538	0.831959
H	6.095385	4.053977	-0.211899
H	6.730621	4.070631	1.446414
H	5.029189	4.455950	1.149117
C	5.200120	1.978129	2.460805

H	4.373474	2.581304	2.866253
H	6.078485	2.162456	3.103450
H	4.916421	0.922377	2.573738
C	2.707090	-2.574345	-0.471790
H	1.711910	-2.090606	-0.347458
C	2.981593	-2.545211	-1.977614
H	3.120946	-1.517227	-2.341543
H	2.153127	-2.995425	-2.549835
H	3.894744	-3.107800	-2.224751
C	2.501760	-4.000395	0.038896
H	1.807031	-4.559600	-0.611131
H	2.081988	-4.017062	1.055314
H	3.444743	-4.565352	0.066039
C	3.852121	-1.743454	2.347984
H	4.573936	-1.009443	2.750164
C	2.468888	-1.300131	2.829618
H	2.305060	-0.225012	2.632300
H	2.344989	-1.420714	3.919158
H	1.653836	-1.887912	2.367967
C	4.238108	-3.106686	2.920291
H	3.578584	-3.912960	2.568358
H	4.186103	-3.105058	4.023299
H	5.265889	-3.384828	2.645800
C	5.765772	-1.989932	-0.127331
H	5.817504	-1.563517	-1.146063
C	6.832444	-1.296259	0.721885
H	6.653143	-0.215476	0.807790
H	7.839968	-1.431041	0.292430
H	6.859143	-1.704951	1.745291
C	6.051153	-3.488172	-0.243957
H	5.967261	-4.003382	0.725318
H	7.077812	-3.662188	-0.610604
H	5.369033	-3.993336	-0.942754

136

Ba1D

Ba	-1.379471	-0.377291	-0.716064
Ba	1.846111	-0.514956	1.651761
Si	4.331842	-1.350968	-0.784826
Si	4.559054	1.558760	0.468795
Si	-4.372267	1.437415	-1.118893
Si	-4.429126	-0.609728	1.310513
N	3.943245	-0.027154	0.226313
N	-3.791194	0.270092	-0.014644
C	-1.149826	-3.836158	-1.170623
C	-1.797789	-3.479118	-2.284943
C	2.680363	-2.114587	-1.445939
C	1.750721	-1.112247	-2.127761
C	2.891343	-3.349830	-2.321903

C	5.107456	-2.763125	0.272480
C	4.160158	-3.243677	1.372710
C	6.434858	-2.338060	0.898291
C	5.506623	-0.996073	-2.263087
C	6.335456	-2.200454	-2.718865
C	4.809655	-0.351725	-3.464451
C	3.367717	2.376974	1.760201
C	3.580030	1.818358	3.176179
C	3.331355	3.906318	1.802986
C	4.517887	2.548383	-1.165390
C	3.084687	2.648252	-1.691646
C	5.229639	3.900678	-1.199782
C	6.325531	1.589017	1.224983
C	7.394064	1.151309	0.222959
C	6.742258	2.898284	1.900260
C	-3.458782	1.166360	-2.819271
C	-3.520305	-0.260122	-3.373846
C	-3.871089	2.178906	-3.889376
C	-3.830003	3.209109	-0.611875
C	-2.306076	3.337697	-0.575033
C	-4.416052	3.622915	0.736696
C	-6.266870	1.411944	-1.417898
C	-6.867319	2.743455	-1.873644
C	-6.720476	0.270171	-2.330242
C	-2.932375	-1.597108	2.041098
C	-1.954712	-0.674922	2.774841
C	-3.251806	-2.829145	2.888641
C	-5.758723	-1.833666	0.673148
C	-5.145404	-2.778941	-0.360235
C	-6.599648	-2.588327	1.701972
C	-5.153806	0.464271	2.728886
C	-6.482066	1.119184	2.349307
C	-5.281086	-0.224467	4.090413
H	0.290517	0.987783	0.525090
H	0.092093	-1.918273	0.627946
H	-1.684596	-4.084707	-0.248699
H	-0.059863	-3.895050	-1.121890
H	-1.262034	-3.248690	-3.212464
H	-2.890307	-3.434213	-2.323413
H	2.128719	-2.465934	-0.549008
H	1.559446	-0.232873	-1.487158
H	0.788212	-1.594628	-2.387989
H	2.170790	-0.724451	-3.065988
H	3.395330	-3.092167	-3.266310
H	1.932399	-3.827617	-2.591276
H	3.506717	-4.112620	-1.821482
H	5.294208	-3.616078	-0.404627
H	4.014944	-2.445038	2.125830
H	4.576482	-4.099914	1.931028

H	3.173923	-3.560325	0.992606
H	7.187474	-2.067978	0.143111
H	6.869763	-3.139415	1.521346
H	6.291960	-1.455460	1.542697
H	6.209757	-0.246996	-1.860127
H	6.954208	-2.617642	-1.911503
H	7.017486	-1.920133	-3.540710
H	5.699166	-3.016667	-3.095811
H	4.174864	-1.078032	-3.996843
H	5.547232	0.020762	-4.196340
H	4.173136	0.497910	-3.179555
H	2.354427	2.100458	1.393722
H	3.849300	0.745174	3.202793
H	2.706670	1.983654	3.835906
H	4.429373	2.319353	3.663877
H	2.610011	4.265950	2.557874
H	3.024547	4.327990	0.836241
H	4.311436	4.336770	2.055943
H	5.070835	1.877669	-1.848174
H	2.580303	1.671673	-1.662866
H	3.056869	3.018171	-2.731743
H	2.474794	3.338254	-1.086180
H	4.788109	4.624565	-0.498429
H	5.169867	4.351550	-2.206233
H	6.296902	3.812013	-0.949141
H	6.273966	0.811147	2.011633
H	7.176173	0.164509	-0.204572
H	8.388871	1.088032	0.697754
H	7.480602	1.862117	-0.615095
H	6.769198	3.738142	1.189560
H	7.754588	2.812031	2.333125
H	6.067186	3.188325	2.717680
H	-2.393540	1.404334	-2.607953
H	-3.420554	-1.034439	-2.594323
H	-2.754535	-0.445092	-4.149253
H	-4.495932	-0.457539	-3.839326
H	-4.921750	2.035358	-4.184787
H	-3.260825	2.077786	-4.803952
H	-3.768252	3.215499	-3.536065
H	-4.213818	3.902471	-1.382475
H	-1.856527	2.631593	0.146789
H	-1.990539	4.343731	-0.248517
H	-1.836397	3.168524	-1.557950
H	-5.516355	3.617479	0.737165
H	-4.093312	4.638974	1.023862
H	-4.085463	2.935318	1.530734
H	-6.671699	1.196085	-0.414213
H	-6.650679	3.561682	-1.171406
H	-7.965495	2.672288	-1.965112

H	-6.483194	3.051208	-2.858946
H	-6.460106	0.468849	-3.382273
H	-7.816318	0.142176	-2.294046
H	-6.267826	-0.693196	-2.052941
H	-2.392812	-2.014110	1.164660
H	-1.660128	0.213385	2.187558
H	-1.034578	-1.223691	3.050656
H	-2.394034	-0.287162	3.706692
H	-2.323207	-3.329154	3.217587
H	-3.838205	-3.569373	2.326081
H	-3.825614	-2.577036	3.792057
H	-6.443253	-1.159353	0.125912
H	-4.577927	-2.212379	-1.114448
H	-5.913116	-3.369051	-0.890629
H	-4.451909	-3.499047	0.106501
H	-5.990857	-3.249438	2.337093
H	-7.355378	-3.224358	1.207474
H	-7.142028	-1.904214	2.370809
H	-4.405534	1.272397	2.836508
H	-6.402032	1.711022	1.429051
H	-6.834244	1.800772	3.143310
H	-7.274775	0.369823	2.191311
H	-5.964877	-1.086379	4.056180
H	-5.683817	0.474039	4.845119
H	-4.317433	-0.589306	4.472957

	136		
Ba2D*			
Ba	-1.377934	-0.618711	-1.141855
Ba	1.754710	-1.055163	1.411163
Si	4.478562	-1.052514	-0.854276
Si	4.010238	1.662916	0.749918
Si	-4.108049	1.544934	-0.976485
Si	-4.232168	-0.748957	1.215671
N	3.785175	0.026220	0.274432
N	-3.599033	0.191529	-0.066556
C	-0.521420	-3.701934	-0.898994
C	-1.482947	-3.459825	-1.862086
C	3.035419	-2.072938	-1.652934
C	1.934521	-1.217109	-2.279270
C	3.511296	-3.152711	-2.625184
C	5.530149	-2.374531	0.071051
C	4.692291	-3.181324	1.063430
C	6.711432	-1.737247	0.800839
C	5.563717	-0.279074	-2.233811
C	6.631900	-1.217203	-2.802282
C	4.757180	0.357073	-3.368793
C	2.626232	1.996919	2.061807
C	2.908103	1.278759	3.390504

C	2.243983	3.455363	2.323020
C	3.782704	2.834568	-0.741686
C	2.375274	2.694849	-1.326102
C	4.171955	4.300951	-0.553242
C	5.703612	1.973010	1.599707
C	6.864342	1.934758	0.605403
C	5.795933	3.225356	2.475015
C	-3.476477	1.322468	-2.808033
C	-3.824530	-0.015057	-3.468621
C	-3.875037	2.491814	-3.711264
C	-3.220003	3.149917	-0.399374
C	-1.707217	3.087754	-0.622668
C	-3.508981	3.448736	1.070779
C	-6.002971	1.848402	-1.004008
C	-6.422149	3.304554	-1.216470
C	-6.756028	0.920082	-1.959646
C	-2.848726	-2.034711	1.657018
C	-1.695361	-1.382350	2.423057
C	-3.294686	-3.323608	2.347432
C	-5.801338	-1.670192	0.615709
C	-5.466821	-2.566457	-0.577651
C	-6.653555	-2.403665	1.650749
C	-4.618392	0.225636	2.827410
C	-5.834542	1.140821	2.684911
C	-4.746977	-0.614196	4.101605
H	0.239664	0.439051	0.361944
H	0.068457	-2.344707	-0.059837
H	-0.813986	-4.140247	0.059297
H	0.504261	-3.932953	-1.199963
H	-1.208902	-3.341928	-2.916513
H	-2.549508	-3.559297	-1.634865
H	2.559379	-2.624352	-0.812414
H	1.606865	-0.415336	-1.593501
H	1.064821	-1.841121	-2.556452
H	2.282526	-0.718781	-3.194551
H	3.969548	-2.704469	-3.520303
H	2.675453	-3.784928	-2.972746
H	4.262165	-3.817557	-2.172311
H	5.921140	-3.077329	-0.686484
H	4.312495	-2.523488	1.869163
H	5.289141	-3.957772	1.572420
H	3.839279	-3.697297	0.590309
H	7.393676	-1.213819	0.114905
H	7.309033	-2.487727	1.347547
H	6.356766	-0.994668	1.533582
H	6.088148	0.541051	-1.713042
H	7.313906	-1.598614	-2.028700
H	7.250331	-0.698635	-3.555619
H	6.184977	-2.091163	-3.302066

H	4.302854	-0.410758	-4.015379
H	5.403975	0.974077	-4.016145
H	3.947375	1.003564	-3.001697
H	1.714139	1.574095	1.582991
H	3.393792	0.290246	3.274686
H	2.000682	1.160921	4.013635
H	3.621770	1.855601	3.997647
H	1.435886	3.525782	3.072299
H	1.878665	3.943427	1.409182
H	3.091971	4.046323	2.699302
H	4.487597	2.405735	-1.477608
H	2.100031	1.638406	-1.456379
H	2.289516	3.197452	-2.305638
H	1.612127	3.136819	-0.665926
H	3.561005	4.799723	0.214148
H	4.035375	4.868355	-1.490793
H	5.225251	4.414914	-0.257641
H	5.810848	1.094252	2.265045
H	6.876839	1.003334	0.024847
H	7.838303	2.011006	1.119743
H	6.811491	2.769410	-0.112445
H	5.642134	4.147426	1.894140
H	6.792551	3.304301	2.944150
H	5.056853	3.228611	3.288708
H	-2.368343	1.392733	-2.745361
H	-3.677706	-0.885473	-2.805710
H	-3.245939	-0.186099	-4.394410
H	-4.886961	-0.049843	-3.746384
H	-4.965612	2.526406	-3.855230
H	-3.418909	2.407322	-4.713206
H	-3.569312	3.460615	-3.288955
H	-3.620408	3.981746	-1.007206
H	-1.242690	2.257402	-0.059330
H	-1.209050	4.005426	-0.264041
H	-1.432620	2.983149	-1.685507
H	-4.581769	3.594846	1.267385
H	-2.986748	4.360635	1.409759
H	-3.169618	2.616075	1.706988
H	-6.309115	1.566138	0.017708
H	-5.990938	3.977735	-0.460976
H	-7.519876	3.412173	-1.162402
H	-6.111414	3.682259	-2.203483
H	-6.596725	1.207857	-3.011138
H	-7.844487	0.963639	-1.780747
H	-6.447558	-0.130262	-1.853653
H	-2.454353	-2.367531	0.676138
H	-1.304207	-0.480110	1.917485
H	-0.866473	-2.105929	2.559954
H	-2.009289	-1.065108	3.429131

H	-2.435244	-3.995930	2.524410
H	-4.021993	-3.879985	1.740181
H	-3.762264	-3.131897	3.324264
H	-6.412874	-0.834159	0.228794
H	-4.881004	-2.019349	-1.331782
H	-6.376194	-2.949104	-1.073070
H	-4.874006	-3.445548	-0.273724
H	-6.106886	-3.224327	2.139718
H	-7.548575	-2.849804	1.181628
H	-7.007997	-1.729579	2.444195
H	-3.729897	0.874959	2.945787
H	-5.727483	1.839383	1.845949
H	-5.988731	1.747358	3.594619
H	-6.760089	0.565445	2.519390
H	-5.584134	-1.326130	4.044330
H	-4.935087	0.031183	4.977947
H	-3.841989	-1.197814	4.321939

	136		
Ba3D			
Ba	-1.315098	-0.092142	0.369447
Ba	2.028212	-1.616937	-0.866209
Si	3.438657	1.704991	-0.540312
Si	5.170336	-0.639784	0.694372
Si	-4.567432	-1.628733	0.048881
Si	-4.451456	1.561106	0.311467
N	3.847238	0.100595	-0.114100
N	-3.909121	-0.060625	0.231913
C	-0.412354	-0.849800	-2.256386
C	0.133624	-1.817245	-3.307683
C	1.546500	1.963564	-0.171189
C	1.182358	1.734549	1.298363
C	1.013059	3.308911	-0.664265
C	3.593028	1.932953	-2.444891
C	2.702120	0.957022	-3.210511
C	5.036339	1.774876	-2.921411
C	4.441875	3.127614	0.282190
C	4.515001	4.409406	-0.554479
C	4.040740	3.476787	1.717894
C	4.693778	-2.515577	0.849527
C	4.820579	-3.258959	-0.490268
C	5.381956	-3.318311	1.955796
C	5.399370	0.112576	2.435225
C	4.129599	-0.073703	3.267591
C	6.654071	-0.263724	3.222966
C	6.822715	-0.559668	-0.284668
C	7.418537	0.848410	-0.295372
C	7.893065	-1.577203	0.119556
C	-3.061161	-2.853448	0.172304

C	-2.540049	-2.985351	1.608606
C	-3.262992	-4.240202	-0.442724
C	-5.360539	-2.010778	-1.656782
C	-4.492542	-1.455352	-2.785321
C	-6.820575	-1.591916	-1.825442
C	-5.870393	-2.092320	1.376583
C	-6.315036	-3.554995	1.336029
C	-5.507446	-1.658377	2.796282
C	-3.038408	2.644238	-0.473281
C	-2.810622	2.250665	-1.935304
C	-3.129748	4.163131	-0.316500
C	-4.637752	2.082993	2.149584
C	-3.331804	1.847040	2.909157
C	-5.212140	3.469345	2.439165
C	-6.094401	1.871951	-0.616867
C	-7.278960	1.372084	0.213694
C	-6.365344	3.280550	-1.145026
H	0.528497	-1.708879	1.057178
H	-0.422920	0.169273	-2.697342
H	-1.490231	-1.103260	-2.157914
H	-0.394731	-1.802150	-4.279596
H	1.192008	-1.615178	-3.584177
H	0.085586	-2.872529	-2.967910
H	1.043083	1.183963	-0.789059
H	1.360730	0.695025	1.622677
H	0.128082	2.010624	1.517467
H	1.768509	2.373516	1.968951
H	1.465961	4.143099	-0.106977
H	-0.079751	3.393907	-0.528129
H	1.222933	3.472593	-1.731159
H	3.253732	2.957061	-2.682362
H	3.089439	-0.073858	-3.101214
H	2.707565	1.153792	-4.296291
H	1.648652	0.974120	-2.889445
H	5.715325	2.514300	-2.472460
H	5.116508	1.880902	-4.017511
H	5.421536	0.777076	-2.655780
H	5.462633	2.709990	0.329058
H	4.929662	4.240770	-1.557949
H	5.153547	5.162321	-0.060332
H	3.522855	4.869443	-0.685549
H	3.086823	4.027886	1.744103
H	4.794836	4.132390	2.186548
H	3.933171	2.592015	2.360796
H	3.626368	-2.485096	1.158544
H	4.584893	-2.641143	-1.377432
H	4.214780	-4.184677	-0.528017
H	5.861798	-3.572402	-0.655233
H	5.033232	-4.366196	1.965394

H	5.174191	-2.897539	2.948876
H	6.474121	-3.339368	1.828331
H	5.480981	1.189627	2.200781
H	3.231895	0.193942	2.691991
H	4.145173	0.544322	4.182290
H	4.005031	-1.120399	3.590573
H	6.697454	-1.338520	3.454706
H	6.688714	0.273749	4.187059
H	7.575306	-0.009198	2.678850
H	6.514652	-0.794473	-1.322292
H	6.708634	1.592152	-0.678076
H	8.318260	0.899368	-0.933081
H	7.719656	1.168727	0.715231
H	8.223594	-1.436009	1.159692
H	8.789446	-1.477323	-0.517523
H	7.549244	-2.616983	0.027945
H	-2.259214	-2.410112	-0.456903
H	-2.522775	-2.029452	2.164095
H	-1.524860	-3.413818	1.650374
H	-3.204309	-3.638061	2.195024
H	-4.102185	-4.776692	0.024593
H	-2.362578	-4.866432	-0.311507
H	-3.468284	-4.182919	-1.521572
H	-5.337631	-3.113683	-1.725231
H	-4.455027	-0.355199	-2.746456
H	-4.878551	-1.740787	-3.779691
H	-3.451183	-1.810617	-2.724281
H	-7.472770	-2.027373	-1.053237
H	-7.214374	-1.914934	-2.805285
H	-6.942134	-0.500306	-1.777266
H	-6.740521	-1.482518	1.069412
H	-6.598619	-3.880926	0.323037
H	-7.184620	-3.731229	1.993306
H	-5.514273	-4.227562	1.683705
H	-4.735080	-2.308142	3.235571
H	-6.381592	-1.701592	3.469273
H	-5.117909	-0.630937	2.814821
H	-2.117049	2.390688	0.096135
H	-2.749482	1.160060	-2.077690
H	-1.896606	2.706606	-2.354307
H	-3.653676	2.587228	-2.559192
H	-2.287902	4.662665	-0.827618
H	-3.096333	4.466860	0.738729
H	-4.055119	4.569833	-0.745588
H	-5.368295	1.344739	2.528120
H	-3.000463	0.799658	2.800284
H	-3.436187	2.033746	3.991930
H	-2.524161	2.513167	2.553306
H	-4.527001	4.275616	2.138539

H	-5.408978	3.602770	3.517544
H	-6.162071	3.640286	1.911938
H	-5.994717	1.207096	-1.494064
H	-7.111209	0.361818	0.612742
H	-8.206103	1.338816	-0.384086
H	-7.472124	2.032563	1.073890
H	-6.392615	4.030382	-0.338427
H	-7.343497	3.323724	-1.655631
H	-5.608346	3.606234	-1.873171

11 References

[S1] N. F. Chilton, C. A. P. Goodwin, D. P. Mills, R. E. P. Winpenny, *Chem. Commun.* **2015**, *51*, 101-103.

[S2] (a) I. C. Cai, M. I. Lipschutz, T. D. Tilley, *Chem. Commun.* **2014**, 50, 13062-13065; (b) B. Shen, L. Ying, J. Chen, Y. Luo, *Inorg. Chim. Acta* **2008**, *361*, 1255-1260.

[S3] S. Harder, S. Müller, E. Hübner, *Organometallics* **2004**, *23*, 178-183.

[S4] M. Westerhausen, *Inorg. Chem.* **1991**, *30*, 96-101.

[S5] M. R. Crimmin, A. G. M. Barrett, M. S. Hill, D. J. MacDougall, M. F. Mahon, P. A. Procopiou, *Chem. Eur. J.* **2008**, *14*, 11292-11295.

[S6] F. Feil, S. Harder, *Organometallics* **2001**, *20*, 4616-4622.

[S7] S. Kaiser, S. P. Smidt, A. Pfaltz, *Angew. Chem. Int. Ed.* **2006**, *45*, 5194-5197.

[S8] J.-D. Leng, C. A. P. Goodwin, I. J. Vitorica-Yrezabal, D. P. Mills, *Dalton Trans.* **2018**, *47*, 12526-12533.

[S9] I. C. Cai, M. S. Ziegler, P. C. Bunting, A. Nicolay, D. S. Levine, V. Kalendra, P. W. Smith, K. V. Lakshmi, T. D. Tilley, *Organometallics* **2019**, *38*, 1648-1663.

[S10] Rigaku OD, CrysAlis PRO, Rigaku Oxford Diffraction Ltd., Yarnton, England.

[S11] O. V. Dolomanov, L. J. Bourhis, R. J. Gildea, J. A. K. Howard, H. Puschmann, *J. Appl. Cryst.* **2009**, *42*, 339-341.

[S12] G. M. Sheldrick, *Acta Crystallogr. A* **2015**, *71*, 3-8.

[S13] G. M. Sheldrick, *Acta Crystallogr. C* **2015**, *71*, 3-8.

[S14] A. Thorn, B. Dittrich, G. M. Sheldrick, *Acta Crystallogr. A* **2015**, *68*, 448-451.

[S15] (a) J. M. Campelo, R. Chakraborty, J. M. Marinas, *Synth. Commun.* **1996**, *26*, 1639-1650; (b) K. Okamoto, R. Akiyama, S. Kobayashi, *J. Org. Chem.* **2004**, *69*, 2871-2873.

[S16] G. A. Gailyunas, G. V. Nurtdinova, F. G. Yusupova, L. M. Khalilov, V. K. Mavrodiev, S. R. Rafikov, Y. Yuriev, *J. Organomet. Chem.* **1981**, *209*, 139-146.

[S17] M. Klessinger, H. van Megen, K. Wilhelm, *Chem. Ber.* **1982**, *115*, 50-56.

[S18] (a) J. K. Whitesell, R. S. Matthews, *J. Org. Chem.* **1977**, *42*, 3878-3882; (b) K. B. Becker, *Helv. Chim. Acta* **1977**, *60*, 68-80.

[S19] Y. Wang, W. Chen, Z. Lu, Z. H. Li, H. Wang, *Angew. Chem. Int. Ed.* **2013**, *52*, 7496-7499.

[S20] (a) A. Krief, L. Hevesi, J. B. Nagy, E. G. Derouane, *Angew. Chem.* **1977**, *89*, 103-105; (b) L. Hevesi, J. B. Nagy, A. Krief, E. G. Derouane, *Org. Magn. Reson.* **1977**, *10*, 14-19.

[S21] N. G. Léonard, P. J. Chirik, *ACS Catal.* **2018**, *8*, 342-348.

[S22] (a) B. I. Yoo, Y. J. Kim, Y. You, J. W. Yang, S. W. Kim, *J. Org. Chem.* **2018**, *83*, 13847-13853; (b) M. Szostak, M. Spain, D. J. Procter, *J. Org. Chem.* **2014**, *79*, 2522-2537.

[S23] B. Han, P. Ma, X. Cong, H. Chen, X. Zeng, *J. Am. Chem. Soc.* **2019**, 141, 9018-9026.

[S24] M. Adamczyk, D. S. Watt, D. A. Netzel, *J. Org. Chem.* **1984**, *49*, 4226-4237.

[S25] H. Fuse, M. Kojima, H. Mitsunuma, M. Kanai, *Org. Lett.* **2018**, *20*, 2042-2045.

[S26] R. R. Cesati III, J. de Armas, A. H. Hoveyda, *Org. Lett.* **2002**, *4*, 395-398.

[S27] M. Yalpani, R. Köster, *Chem. Ber.* **1990**, *123*, 719-724.

[S28] R. L. Funk, K. P. C. Vollhardt, *J. Am. Chem. Soc.* **1980**, *102*, 5245-5253.

[S29] (a) J. P. Grealis, H. Müller-Bunz, Y. Ortin, M. Condell, M. Casey, M. J. McGlinchey, *Chem. Eur. J.* **2008**, *14*, 1552-1560; (b) H. Lund, Abstracts of Papers, 201st ACS National Meeting, Philadelphia, PA, 2002, http://www.electrochem.org/dl/ma/201/pdfs/1246.pdf.

[S30] (a) R. Neufeld, D. Stalke, *Chem. Sci.* **2015**, *6*, 3354-3364; (b) S. Bachmann, R. Neufeld, M. Dzemski, D. Stalke, *Chem. Eur. J.* **2016**, *22*, 8462-8465; (c) A.-K. Kreyenschmidt, S. Bachmann, T. Niklas, D. Stalke, *ChemistrySelect* **2017**, *2*, 6957-6960.

[S31] M. J. Frisch, G. W. Trucks, H. B. Schlegel, G. E. Scuseria, M. A. Robb, J. R. Cheeseman, G. Scalmani, V. Barone, G. A. Petersson, H. Nakatsuji, X. Li, M. Caricato, A. V. Marenich, J. Bloino, B. G. Janesko, R. Gomperts, B. Mennucci, H. P. Hratchian, J. V. Ortiz, A. F. Izmaylov, J. L.

Sonnenberg, D. Williams-Young, F. Ding, F. Lipparini, F. Egidi, J. Goings, B. Peng, A. Petrone, T. Henderson, D. Ranasinghe, V. G. Zakrzewski, J. Gao, N. Rega, G. Zheng, W. Liang, M. Hada, M. Ehara, K. Toyota, R. Fukuda, J. Hasegawa, M. Ishida, T. Nakajima, Y. Honda, O. Kitao, H. Nakai, T. Vreven, K. Throssell, J. A. Montgomery, J. E. Peralta, F. Ogliaro, M. J. Bearpark, J. J. Heyd, E. N. Brothers, K. N. Kudin, V. N. Staroverov, T. A. Keith, R. Kobayashi, J. Normand, K. Raghavachari, A. P. Rendell, J. C. Burant, S. S. Iyengar, J. Tomasi, M. Cossi, J. M. Millam, M. Klene, C. Adamo, R. Cammi, J. W. Ochterski, R. L. Martin, K. Morokuma, O. Farkas, J. B. Foresman, D. J. Fox, Gaussian 16 Rev. A.03, Wallingford CT, USA, 2016.

[S32] (a) A. D. Becke, *J. Chem. Phys.* **1993**, *98,* 5648-5652; (b) J. P. Perdew, J. A. Chevary, S. H. Vosko, K. A. Jackson, M. R. Pederson, D. J. Singh, C. Fiolhais, *Phys. Rev. B* **1992**, *46*, 6671-6687. (c) F. Weigend, R. Ahlrichs, *Phys. Chem. Chem. Phys.* **2005**, *7,* 3297-305; (d) F. Weigend, *Phys. Chem. Chem. Phys.* **2006**, *8,* 1057-1065.

[S33] S. Grimme, S. Ehrlich, L. Goerigk, *J. Comp. Chem.* **2011**, *32*, 1456-1465.

[S34] A. V. Marenich, C. J. Cramer, D. G. Truhlar, *J. Phys. Chem. B* **2009**, *113*, 6378-6396.

[S35] N. van Eikema Hommes, Molecule, Erlangen, Germany, 2018.
